# Human-SARS-CoV-2 interactome and human genetic diversity: *TMPRSS2*-rs2070788, associated with severe influenza, and its population genetics caveats in Native Americans

**DOI:** 10.1590/1678-4685-GMB-2020-0484

**Published:** 2021-08-25

**Authors:** Fernanda S.G. Kehdy, Murilo Pita-Oliveira, Mariana M. Scudeler, Sabrina Torres-Loureiro, Camila Zolini, Rennan Moreira, Lucas A. Michelin, Isabela Alvim, Carolina Silva-Carvalho, Vinicius C. Furlan, Marla M. Aquino, Meddly L. Santolalla, Victor Borda, Giordano B. Soares-Souza, Luis Jaramillo-Valverde, Andres Vasquez-Dominguez, Cesar Sanchez Neira, Renato S. Aguiar, Ricardo A. Verdugo, Timothy D. O`Connor, Heinner Guio, Eduardo Tarazona-Santos, Thiago P. Leal, Fernanda Rodrigues-Soares

**Affiliations:** 1 Instituto Oswaldo Cruz, Fundação Oswaldo Cruz, Laboratório de Hanseníase, Rio de Janeiro, RJ, Brazil.; 2 Universidade Federal do Triângulo Mineiro, Instituto de Ciências Biológicas e Naturais, Departamento de Patologia, Genética e Evolução, Uberaba, MG, Brazil.; 3 Universidade Federal de Minas Gerais, Instituto de Ciências Biológicas, Departamento de Genética, Ecologia e Evolução, Belo Horizonte, MG, Brazil.; 4 Mosaico Translational Genomics Initiative, Belo Horizonte, MG, Brazil.; 5 Universidad Peruana Cayetano Heredia, School of Public Health and Administration, Emerging Diseases and Climate Change Research Unit, Lima, Peru.; 6 Laboratório Nacional de Computação Científica (LNCC), Laboratório de Bioinformática, Petrópolis, RJ, Brazil.; 7 INBIOMEDIC Research and Technological Center, Lima, Peru.; 8 Instituto Nacional de Salud, Lima, Peru.; 9 Universidad de Chile, Facultad de Medicina, Instituto de Ciencias Biomédicas, Programa de Genética Humana, Santiago, Chile.; 10 Universidad de Chile, Facultad de Medicina, Departamento de Oncología Básico Clínica, Santiago, Chile.; 11 University of Maryland School of Medicine, Institute for Genome Sciences, Baltimore, United States.; 12 University of Maryland School of Medicine, Program in Personalized and Genomic Medicine Baltimore, United States.; 13 University of Maryland School of Medicine, Department of Medicine, Baltimore, United States.; 14 Universidad de Huánuco, Huanuco, Peru.

**Keywords:** TMPRSS2, ACE2, Native Americans, SARS-CoV-2, population genomics

## Abstract

For human/SARS-CoV-2 interactome genes *ACE2*, *TMPRSS2* and *BSG*, there is a convincing evidence of association in Asians with influenza-induced SARS for *TMPRSS2*-rs2070788, tag-SNP of the eQTL rs383510. This case illustrates the importance of population genetics and of sequencing data in the design of genetic association studies in different human populations: the high linkage disequilibrium (LD) between rs2070788 and rs383510 is Asian-specific. Leveraging on a combination of genotyping and sequencing data for Native Americans (neglected in genetic studies), we show that while their frequencies of the Asian tag-SNP rs2070788 is, surprisingly, the highest worldwide, it is not in LD with the eQTL rs383510, that therefore, should be directly genotyped in genetic association studies of SARS in populations with Native American ancestry.

In the context of a global interest in host genetic determinants of COVID-19 susceptibility (Casanova and Su, 2020) we established a three-step protocol to gain evidence about human genetic susceptibility to the SARS-CoV-2, the causative agent of the COVID-19 disease: 

(i) a systematic review of the literature about genes *ACE2* (angiotensin converting enzyme 2, Xp22.2), *TMPRSS2* (transmembrane serine protease 2, 21q22.3) and *BSG* (basigin, 19p13.3), which codify important proteins for severe acute respiratory syndrome coronavirus 2 (SARS-CoV-2) infection. SARS-CoV-2 spike S protein contains subunits S1 and S2, which bind the ACE2 cellular receptor, leading to an endosome formation around the virus. After this binding, TMPRSS2 host’s transmembrane serine protease cleaves S1/S2 subunits and induces a conformational change in S2, facilitating the endosome formation and allowing the entrance of virus cellular into the cytoplasm. CD147 (also called basigin - BSG) is a transmembrane glycoprotein, encoded by the *BSG* gene, discovered as a new SARS-CoV-2 cellular entry route (Wang et al. 2020). We performed a systematic review under the terms “[gene name] genetics infection]”, covering articles published until June 4th, 2020 in PubMed and in bioRxiv during 2020 (Figure 1A). For the ACE2 and BSG viral receptors, there was no solid and direct evidence of association between genetic polymorphisms and any respiratory viral infections.

**Figure 1 ‒:**
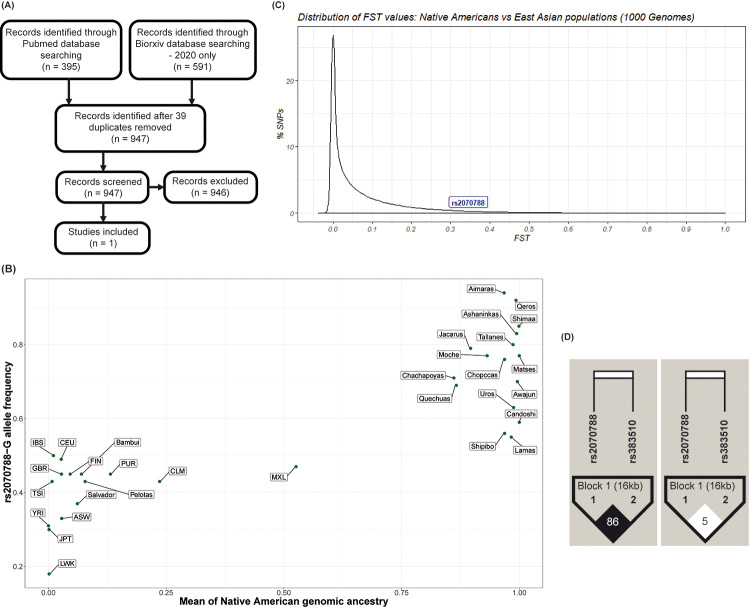
(A) PRISMA flowchart of the systematic review; (B) Frequencies of the rs2070788 SNP and Native American ancestry in different populations (Populations form 1000 Genomes Project: ASW, Americans of African Ancestry in SW USA; CEU, Utah Residents (CEPH) with Northern and Western European Ancestry; CLM, Colombians from Medellin, Colombia; FIN, Finnish in Finland; GBR, British in England and Scotland; IBS, Iberian Population in Spain; JPT, Japanese in Tokyo, Japan; LWK, Luhya in Webuye, Kenya; PUR, Puerto Ricans from Puerto Rico; TSI, Toscani in Italia; YRI, Yoruba in Ibadan, Nigeria); (C) Fst values distribution of Native Americans vs East Asian populations for 71 SNPs of *TMPRSS2* gene; (D) Linkage disequilibrium between rs2070788 and rs383510 in East Asian and Native American populations.

(ii) we annotated SNVs in *ACE2*, *TMPRSS2*, and *BSG* mining and integrating information from 24 biological and biomedical databases, using our bioinformatics tool (MASSA) [Multi-Agent System for SNP Annotation (Soares-Souza, 2014)], to identify functionally relevant variants (Table S1-A). MASSA integrates data with clinical findings from NCBI Databases like ClinVar and ClinGen. MASSA also includes approaches to distinguish between functional alleles, underlying clinical phenotypes and benign variants, cross-checking the data with multiple different databases. To ensure that collected variants are relevant for our analysis, MASSA performs some secondary filters, taking into account the frequency of alleles and SIFT and Polyphen predictions. The tool, in addition to performing the filters described above, searches for variants that have been cited in PubMed and also compares them to the OMIM database. From that, we’ve found 26 putatively functional variants for *ACE2*, 5 for *TMPRSS2* and 17 for *BSG* gene, resulting in a total of 48 genetic variants.

(iii) we performed a population genetics analysis of the 48 functionally relevant variants in the *ACE2*, *TMPRSS2* and *BSG* genes in human populations to detect particular patterns of between-population genetic differentiation and independently of evidence of genetic association between *ACE2*, *TMPRSS2* and *BSG* variants and infectious diseases, using published and unpublished data from different worldwide populations (Table S1-B), enriched for Latin Americans, who are mainly the product of admixture of Native Americans, Europeans and Africans. Unpublished data include the Peruvian Native Americans from the *Laboratório de Diversidade Genética Humana (UFMG)* and the whole genome sequenced Native Americans and admixed Peruvian populations from the Peruvian Genome Project. Detailed methodology is available on Text S1. 

*ACE2* and *BSG* allele frequencies and their regression analyses between population genomic ancestry (Native American, African, European and East Asian) and frequencies of functionally relevant SNPs are presented in Table S2 (A and B) and Table S3 (A and B), respectively. We did not observe a particular pattern of inter-population genetic diversity for most of our 48 analyzed SNPs. Our most illustrative result regards *TMPRSS2* (Table S4). In our systematic review, the only genotype/infection association was reported by Cheng et al. (2015), between rs2070788-G, a tag-SNP (i.e. in high linkage disequilibrium, r^2^>0.80) of the regulatory e-QTL rs383510. Both SNPs are located in intronic regions and were associated in Asiatic populations with severe pulmonary damage caused by influenza A(H7N9) in 2014 (OR 1.70 [1.13-2.55]) and rs2070788 was associated with severe pulmonary damage caused by the influenza A(H1N1) in 2009 (OR 1.54 [1.14-2.06]). The authors validated their finding by an *in-vitro* polymerase assay, showing that rs383510 maps on a region that regulates *TMPRSS2* expression (rs383510-T promotes a higher expression of TMPRSS2 than rs383510-C), and therefore is a functionally relevant SNP tagged by rs2070788-G. This result and the role of TMPRSS2 in SARS-CoV-2 infection suggest that there are shared elements in the pathogenesis of SARS caused by different viral infections. 

As in Cheng et al. (2015), the tag-SNP rs2070788 (https://www.ncbi.nlm.nih.gov/snp/rs2070788) is more commonly studied than the functional SNP rs383510 (https://www.ncbi.nlm.nih.gov/snp/rs383510), because the former is present in more SNP genome-wide arrays and has a TaqMan (Thermo Fisher, US) probe, while rs383510 does not. Irham et al. (2020) by analyzing variants that modify TMPRSS2 expression, have observed that rs2070788-G and rs383510-T were associated with the increase of protein expression in lung tissue. For this reason, there is a possibility of association to a higher susceptibility to COVID-19 development. Moreover, Latini et al. (2020), using complete exome sequencing, have evidenced that *TMPRSS2*-rs75603675 and rs12329760 were associated with COVID-19 protection. We examined our unpublished dataset of Native American and of admixed Latin Americans for the putative tag-SNP rs2070788 (genotyped with the Illumina Omni2.5 array) but not for rs383510 because there is no large dataset available for it. We realized that, interestingly, frequencies of the putative tag-SNP rs2070788-G are strongly correlated with population Native American ancestry (Figure 1B, Table S4), and its highest frequency worldwide are in Native Americans. Non-admixed Native American populations have frequencies between 76% and 94%, compared to around 50% in Europeans, 30-40% in Asians and 18-33% in Africans. Furthermore, the putative tag-SNP rs2070788-G is among the 5% most differentiated SNPs in Native Americans respect to Asians (the genetically closest continental group, Figure 1C). This result led us to hypothesize that Native Americans may have the highest frequencies of SARS-CoV-2 susceptibility alleles in *TMPRSS2* and to test this hypothesis we designed a further association study between rs2070788 and COVID-19 in Peru (a country inhabited by populations with predominant Native American ancestry). 

Mills and Rahal (2020) described that in 2020, 81,5% and 11,2% of the genome-wide association studies (GWAS) have analyzed, respectively, Europeans and Asians; in contrast, 0.38% have investigated Latin Americans. Recently, Ellinghaus et al. (2020) have published a GWAS (n=3,815 Europeans) and found a 3p21.31 gene cluster as a susceptibility locus in COVID-19 with respiratory failure and a possible contribution of the ABO blood-group system. However, none of recent COVID-19 GWAS have analyzed Native American populations.

Because Harris et al. (2018) have published whole genome sequencing data for 150 Peruvian individuals with high Native American ancestry, we used those data to test the linkage disequilibrium between the putative tag-SNP rs2070788 and the functional SNP rs383510. Surprisingly for us, in these Native Americans, the continental group that, on average, shows the highest linkage disequilibrium in the human genome (Bosch et al. 2009), there is no linkage disequilibrium between rs2070788 and rs383510 (r^2^=0.05, D’=0.61, Figure 1D). We verified that rs2070788 and rs383510 are in linkage disequilibrium only in Asian populations (Figure 1D) and therefore, the former is a tag-SNP of the latter functional SNP only in Asians. Thus, based on our current knowledge, there is no evidence that Native Americans have the highest frequency worldwide of *TMPRSS2* SARS susceptibility variants, as a superficial analysis would suggest, which was not the case of this study. In this context, as a previous example of distinct patterns of LD, Hünemeier et al. (2015) have demonstrated that two-SNP haplotypes, earlier suggested as proxies for 5-HTTLPR by Vinkhuyzen et al. (2011) in European descendants, could not be used in such way for Native Americans due to their absence of linkage disequilibrium at this locus. An association study in Native Americans should focus on the causative variant rs383510, to test its involvement in SARS induced by viral infection.

In summary, this case illustrates that, to properly design genetic association studies, it is compelling to: (i) consider the complexities of population genetics concepts such as differences not only in frequencies but also in linkage disequilibrium among different human populations, (ii) to have access to whole genome sequencing data for the broadest array of human populations, as we have in this case for Peruvians Native Americans, (iii) to perform genetic studies including neglected populations, such as Native American, aiming to create specific genetic knowledge for these populations. Moreover, if for any reason, including socioeconomic vulnerability, COVID-19 is more common in individuals with high Native American ancestries, the test of association between the rs383510 and COVID-19 phenotypes should be controlled for ancestry. Without considering differences in linkage disequilibrium (also for imputation in GWAS) and sequencing data, as well as ancestry, this is an example of how association studies may reach misleading conclusions in times when a search for susceptibility variants for SARS-CoV-2 is intense.

## Supplementary material

The following online material is available for this article:

Text S1 - Detailed Methods.

Table S1-A - Twenty six biological and biomedical databases integrated by MASSA.

Table S1-B - Sample datasets descriptions.

Table S2-A - *ACE2* allele frequencies.

Table S2-B - *ACE2* regression values.

Table S3-A - *BSG* allele frequencies.

Table S3-B - *BSG* regression values.

Table S4-A - *TMPRSS2* allele frequencies.

Table S4-B - *TMPRSS2* regression values.

## References

[B1] Bosch E, Laayouni H, Morcillo-Suarez C, Casals F, Moreno-Estrada A, Ferrer-Admetlla A, Gardner M, Rosa A, Navarro A, Comas D (2009). Decay of linkage disequilibrium within genes across HGDP-CEPH human samples: Most population isolates do not show increased LD. BMC Genomics.

[B2] Casanova J-L, Su HC, COVID Human Genetic Effort (2020). A global effort to define the human genetics of protective immunity to SARS-CoV-2 infection. Cell.

[B3] Cheng Z, Zhou J, To KK-W, Chu H, Li C, Wang D, Yang D, Zheng S, Hao K, Bossé Y (2015). Identification of TMPRSS2 as a susceptibility gene for severe 2009 pandemic A(H1N1) influenza and A(H7N9) influenza. J Infect Dis.

[B4] Ellinghaus D, Degenhardt F, Bujanda L, Buti M, Albillos A, Invernizzi P, Fernández J, Prati D, Baselli G, Asselta R (2020). Genomewide association study of severe Covid-19 with respiratory failure. N Engl J Med.

[B5] Harris DN, Song W, Shetty AC, Levano KS, Cáceres O, Padilla C, Borda V, Tarazona D, Trujillo O, Sanchez C (2018). Evolutionary genomic dynamics of Peruvians before, during, and after the Inca Empire. Proc Natl Acad Sci U S A.

[B6] Hünemeier T, Bisso-Machado R, Salzano FM, Bortolini MC (2015). Native American ancestry leads to complexity in 5-HTTLPR polymorphism association studies. Mol Psychiatry.

[B7] Irham LM, Chou W-H, Calkins MJ, Adikusuma W, Hsieh S-L, Chang W-C (2020). Genetic variants that influence SARS-CoV-2 receptor TMPRSS2 expression among population cohorts from multiple continents. Biochem Biophys Res Commun.

[B8] Latini A, Agolini E, Novelli A, Borgiani P, Giannini R, Gravina P, Smarrazzo A, Dauri M, Andreoni M, Rogliani P (2020). COVID‐19 and genetic variants of protein involved in the SARS‐CoV‐2 entry into the host cells. Genes (Basel).

[B9] Mills MC, Rahal C (2020). The GWAS diversity monitor tracks diversity by disease in real time. Nat Genet.

[B10] Soares-Souza GB (2014). New approaches for database integration and development of bioinformatics tools for population genetics studies.

[B11] Vinkhuyzen AAE, Dumenil T, Ryan L, Gordon SD, Henders AK, Madden PAF, Heath AC, Montgomery GW, Martin NG, Wray NR (2011). Identification of tag haplotypes for 5HTTLPR for different genome-wide SNP platforms. Mol Psychiatry.

[B12] Wang K, Chen W, Zhou Y-S, Lian J-Q, Zhang Z, Du P, Gong L, Zhang Y, Cui H-Y, Geng J-J (2020). SARS-CoV-2 invades host cells via a novel route: CD147-spike protein. bioRxiv.

