# Supplementary material for: Human-SARS-CoV-2 interactome and human genetic diversity: TMPRSS2-rs2070788, associated with severe influenza, and its population genetics caveats in Native Americans
Source: Genet Mol Biol. 2021 Aug 25;44(1 Suppl 1):e20200484. doi: 10.1590/1678-4685-GMB-2020-0484 (PMC8387978; doi:10.1590/1678-4685-GMB-2020-0484)
Supplement: Text S1 - [file 1415-4757-GMB-44-1-s1-e20200484-s1.pdf]

Genetics and Molecular Biology
https://doi.org/10.1590/1678-4685-GMB-2020-0484

Supplementary Material to "Human-SARS-CoV-2 interactome and human genetic diversity: *TMPRSS2*-rs2070788, associated with severe influenza, and its population genetics caveats in Native Americans"

Text S1 – Detailed Methods.

Human-SARS-CoV-2 interactome and human genetic diversity: *TMPRSS2*-rs2070788, associated with severe influenza, and its population genetics caveats in Native Americans
Kehdy et al.

Methods

Systematic Review

The search was performed in Pubmed, using the terms "ace2 genetics infection", "tmprss2 genetics infection", "cd147 genetics infection" and "bsg genetics infection" covering articles published until June 4th, 2020. Additionally, we searched the biorxiv database for studies of 2020. Studies included should have covered any type of infection and information about genetic polymorphisms in *ACE2*, *TMPRSS2* or *BSG*.

*SNPs annotation - MASSA (*Multi-Agent System for SNP Annotation*)*

Variation lists of each gene were downloaded from human genome assembly Feb.2009 (GRCH37/hg19), from the UCSC Genome Browser (http://genome.ucsc.edu/). We selected the option "dbSNP Reference SNP (rs) identifier", that results in a list of all variations inside the genes. For *TMPRSS2*, *ACE2* and *BSG* genes we obtained 11,453, 534 and 4,258 SNPs respectively.

Functional annotation for all variants was performed with MASSA, that is a SNP annotation tool that queries and aggregates information from 24 public databases and two additional tools (SIFT

and Polyphen) (Table S1-A). The population genetics analyses were performed on the functionally relevant SNPs.

Dataset Description

We studied 66 populations from five different datasets: i) 1000 Genomes Phase 3 (ftp://ftp.1000genomes.ebi.ac.uk/vol1/ftp/release/20130502/); ii) The EPIGEN-Brazil project dataset (Kehdy et al. 2015): three Brazilian population-based cohorts (Salvador, Bambuí and Pelotas) that consist of 265 individuals genotyped with the Illumina HumanOmni5-4v1 array; iii) One hundred twenty nine Peruvian Native Americans genotyped on a Illumina HumanOmni2.5-8v1 array from the Laboratório de Diversidade Genética Humana (LDGH, unpublished) and iv) Native Americans and admixed Peruvian populations from Peruvian Genome Project (Harris et al. 2018) that include 150 whole genome sequenced individuals and 712 unpublished individuals genotyped on a Illumina HumanOmni2.5-8v1 array and v) Chilean population from ChileGenomico Project (http://www.chilegenomico.cl/) and Patagonia DNA Project. This dataset includes 9 whole genome sequenced individuals recruited from the north of Chile with Aymara ancestry, 9 individuals from the Metropolitan Region (the capital, in the center of the country) with Mapuche ancestry and 17 patagonians consisting, by ethnicity, of 3 Pehuenche, 3 Huilliche, 3 Chilote (putative Chono descendants), 4 Kaweskar, and 4 Yamana (Verdugo et al. 2020). The datasets descriptions are summarised at Table S1-B. All allele frequencies necessary to reproduce the analyses are available as supplementary tables. The informed consent was obtained for all individuals in this study.

Fst and Linkage Disequilibrium

Differentiation of the *TMPRSS2* gene between Native Americans and Asians was tested by calculating Fst. To perform this analysis, we integrated data from 71 SNPS of the *TMPRSS2* gene genotyped in 218 Peruvians from the Dr. Tarazona's group and from the Peruvian Genome Project with data from East Asia populations from 1000 Genomes Project (Table S1-B). The Fst

for each *TMPRSS2* SNP was estimated in a pairwise fashion, between Peruvian Native-Americans and East Asians using the hierfstat R package (de Meeûs and Goudet 2007) (Figure 1C).

The linkage disequilibrium [(Hill and Robertson 1968) r2, D`, LD] between rs2070788 and rs383510 was calculated using genotyping data from East Asians populations from 1000 Genomes Project and sequencing data from Peruvian Native-Americans of Peruvian Genome Project (Table S1-B) using Haploview 4.2 (Barrett et al. 2005) (Figure 1D).

Genomic ancestry and regression analysis

For genomic ancestry estimation, datasets from 1000 Genomes Project (CEU, IBS, FIN, GBR, TSI, LWK, YRI, JPT, ASW, CLM, MXL and PUR), EPIGEN-Brazil project (Salvador, Bambuí and Pelotas) and Native Americans and Admixed Peruvian populations (Aymaras, Ashaninkas, Awajun, Candoshi, Chachapoyas, Chopccas, Jacarus, Lamas, Matses, Moche, Qeros, Shipibo, Quechuas, Shimaa, Tallanes and Uros) were integrated into a single database containing autosomal genomewide SNPs shared by all these populations (Table S1-B). African, European, Asian and Native American genomic ancestries were estimated by ADMIXTURE (Alexander et al. 2009) on unsupervised mode using k=4.

Linear regression coefficient beta was estimated for each allele frequency in each studied population for the functionally relevant variants on each continental ancestry (Native American, European, African and East Asian) in R environment, using *logit* function (Cribari-Neto and Zeileis 2010).

References

Alexander DH, Novembre J and Lange K (2009) Fast model-based estimation of ancestry in unrelated individuals. Genome Res 19:1655–64. doi: 10.1101/gr.094052.109

Barrett JC, Fry B, Maller J and Daly MJ (2005) Haploview : analysis and visualization of LD and haplotype maps. Bioinformatics 21:263–265. doi: 10.1093/bioinformatics/bth457

Cribari-Neto F and Zeileis A (2010) Beta regression in R. J Stat Softw 34:1–24. doi: 10.18637/jss.v034.i02
de Meeûs T and Goudet J (2007) A step-by-step tutorial to use HierFstat to analyse populations hierarchically structured at multiple levels. Infect Genet Evol 7:731–5. doi: 10.1016/j.meegid.2007.07.005
Hill WG and Robertson A (1968) Linkage disequilibrium in finite populations. Theor Appl Genet 38:226–231. doi: 10.1007/BF01245622
Kehdy FSG, Gouveia MH, Machado M, Magalhães WCS, Horimoto AR, Horta BL, Moreira RG, Leal TP, Scliar MO, Soares-Souza GB et al. (2015) Origin and dynamics of admixture in Brazilians and its effect on the pattern of deleterious mutations. Proc Natl Acad Sci 112:8696–8701. doi: 10.1073/pnas.1504447112
Verdugo RA, Di Genova A, Herrera L, Moraga M, Acuña M, Berríos S, Llop E, Valenzuela CY, Bustamante ML, Digman D et al. (2020) Development of a small panel of SNPs to infer ancestry in Chileans that distinguishes Aymara and Mapuche components. Biol Res 53:15. doi: 10.1186/s40659-020-00284-5
