# Supplementary material for: Human-SARS-CoV-2 interactome and human genetic diversity: TMPRSS2-rs2070788, associated with severe influenza, and its population genetics caveats in Native Americans
Source: Genet Mol Biol. 2021 Aug 25;44(1 Suppl 1):e20200484. doi: 10.1590/1678-4685-GMB-2020-0484 (PMC8387978; doi:10.1590/1678-4685-GMB-2020-0484)
Supplement: Table S1-A - [file 1415-4757-GMB-44-1-s1-e20200484-s2.pdf]

Genetics and Molecular Biology
https://doi.org/10.1590/1678-4685-GMB-2020-0484

Supplementary Material to "Human-SARS-CoV-2 interactome and human genetic diversity: *TMPRSS2*-rs2070788, associated with severe influenza, and its population genetics caveats in Native Americans"

Table S1-A – Twenty six biological and biomedical databases integrated by MASSA.

Category	Database	Link
**Genomic Mapping and additional annotations**	dbSNP	https://www.ncbi.nlm.nih.gov/snp/
**Gene/Protein consequences**	NCBI RefSeq	https://www.ncbi.nlm.nih.gov/refseq/
	UCSC Known Genes	https://www.ncbi.nlm.nih.gov/pubmed/16500937
	Ensembl	https://www.ensembl.org/
	Vega	http://vega.archive.ensembl.org/
	AceView	https://www.ncbi.nlm.nih.gov/IEB/Research/Acembly/
	CCDS	https://www.ncbi.nlm.nih.gov/CCDS/
**Effect on Protein Function for non-synonymous single amino acid substitution**	SIFT	https://sift.bii.a-star.edu.sg/
	PolyPhen	http://genetics.bwh.harvard.edu/pph/
**Population Data**	HapMap Consortium	https://www.genome.gov/10001688/international-hapmap-project
	1000 Genomes Project	http://www.internationalgenome.org/
	gnomAD	https://gnomad.broadinstitute.org/
**Regulatory Elements**	ENCODE	https://www.encodeproject.org/
	Roadmap Epigenomics	http://www.roadmapepigenomics.org/
	Ensembl Regulatory Build	http://www.ensembl.org/info/docs/funcgen/index.html
**Phenotype and Disease Association**	GAD	https://geneticassociationdb.nih.gov/
	COSMIC	https://cancer.sanger.ac.uk/cosmic/analyses
	GWAS Catalog	https://www.ebi.ac.uk/gwas/
	ClinVar	https://www.ncbi.nlm.nih.gov/clinvar/
	PharmGKB	https://www.pharmgkb.org/
**Non-coding Variation Scoring**	CADD	https://cadd.gs.washington.edu/
	FitCons	http://compgen.cshl.edu/fitCons/
	GWAVA	https://www.sanger.ac.uk/sanger/StatGen_Gwava
	ReMM	https://charite.github.io/software-remm-score.html
**Pathway analysis**	Reactome	https://reactome.org/
**Biological/Clinical Interpretation**	Cancer Genome Interpreter	https://www.cancergenomeinterpreter.org/
