# Supplementary material for: Human-SARS-CoV-2 interactome and human genetic diversity: TMPRSS2-rs2070788, associated with severe influenza, and its population genetics caveats in Native Americans
Source: Genet Mol Biol. 2021 Aug 25;44(1 Suppl 1):e20200484. doi: 10.1590/1678-4685-GMB-2020-0484 (PMC8387978; doi:10.1590/1678-4685-GMB-2020-0484)
Supplement: Table S1-B - [file 1415-4757-GMB-44-1-s1-e20200484-s3.pdf]

Genetics and Molecular Biology
https://doi.org/10.1590/1678-4685-GMB-2020-0484

Supplementary Material to "Human-SARS-CoV-2 interactome and human genetic diversity: *TMPRSS2*-rs2070788, associated with severe influenza, and its population genetics caveats in Native Americans"

Table S1-B – Sample datasets descriptions.

POP	Group	DataSet	N	Data Type	Analysis
ACB	AFR	1000 Genomes Project	96	WGS	Alelle frequencies
ASW	AFR	1000 Genomes Project	61	WGS	Alelle frequencies, ADMIXTURE
BEB	SAS	1000 Genomes Project	86	WGS	Alelle frequencies
CDX	EAS	1000 Genomes Project	93	WGS	Alelle frequencies, LD, FST
CEU	EUR	1000 Genomes Project	99	WGS	Alelle frequencies, ADMIXTURE
CHB	EAS	1000 Genomes Project	103	WGS	Alelle frequencies, LD, FST
CHS	EAS	1000 Genomes Project	105	WGS	Alelle frequencies, LD, FST
CLM	AMR	1000 Genomes Project	94	WGS	Alelle frequencies, ADMIXTURE
ESN	AFR	1000 Genomes Project	99	WGS	Alelle frequencies
FIN	EUR	1000 Genomes Project	99	WGS	Alelle frequencies, ADMIXTURE
GBR	EUR	1000 Genomes Project	91	WGS	Alelle frequencies, ADMIXTURE
GIH	SAS	1000 Genomes Project	103	WGS	Alelle frequencies
GWD	AFR	1000 Genomes Project	113	WGS	Alelle frequencies
IBS	EUR	1000 Genomes Project	107	WGS	Alelle frequencies, ADMIXTURE
ITU	SAS	1000 Genomes Project	102	WGS	Alelle frequencies
JPT	EAS	1000 Genomes Project	104	WGS	Alelle frequencies, ADMIXTURE, LD, FST
KHV	EAS	1000 Genomes Project	99	WGS	Alelle frequencies, LD, FST
LWK	AFR	1000 Genomes Project	99	WGS	Alelle frequencies, ADMIXTURE
MSL	AFR	1000 Genomes Project	85	WGS	Alelle frequencies
MXL	AMR	1000 Genomes Project	64	WGS	Alelle frequencies, ADMIXTURE
PEL	AMR	1000 Genomes Project	85	WGS	Alelle frequencies
PJL	SAS	1000 Genomes Project	96	WGS	Alelle frequencies
PUR	AMR	1000 Genomes	104	WGS	Alelle frequencies,

POP	Group	DataSet	N	Data Type	Analysis
		Project			ADMIXTURE
STU	SAS	1000 Genomes Project	102	WGS	Alelle frequencies
TSI	EUR	1000 Genomes Project	107	WGS	Alelle frequencies, ADMIXTURE
YRI	AFR	1000 Genomes Project	108	WGS	Alelle frequencies, ADMIXTURE
BAMBUI	Admixed Brazilian	EPIGEN-BRAZIL Project	88	Illumina HumanOmni5-4v1 array	Alelle frequencies, ADMIXTURE
PELOTAS	Admixed Brazilian	EPIGEN-BRAZIL Project	87	Illumina HumanOmni5-4v1 array	Alelle frequencies, ADMIXTURE
SALVADOR	Admixed Brazilian	EPIGEN-BRAZIL Project	90	Illumina HumanOmni5-4v1 array	Alelle frequencies, ADMIXTURE
Aimaras	Andean	LDGH	16	Illumina HumanOmni2.5-8v1 array	Alelle frequencies, ADMIXTURE, FST
Ashaninkas	Amazon	LDGH	44	Illumina HumanOmni2.5-8v1 array	Alelle frequencies, ADMIXTURE, FST
Quechuas	Andean	LDGH	24	Illumina HumanOmni2.5-8v1 array	Alelle frequencies, ADMIXTURE, FST
Shimaa	Amazon	LDGH	45	Illumina HumanOmni2.5-8v1 array	Alelle frequencies, ADMIXTURE, FST
Afro_des	Admixed Peruvian	Peruvian Genome Project	47	Illumina HumanOmni2.5-8v1 array	Alelle frequencies
Ancash	Admixed Peruvian	Peruvian Genome Project	36	Illumina HumanOmni2.5-8v1 array	Alelle frequencies
Arequipa	Admixed Peruvian	Peruvian Genome Project	36	Illumina HumanOmni2.5-8v1 array	Alelle frequencies
Awajun	Amazon	Peruvian Genome Project	23	Illumina HumanOmni2.5-8v1 array	Alelle frequencies, ADMIXTURE, FST
Ayacucho	Admixed Peruvian	Peruvian Genome Project	36	Illumina HumanOmni2.5-8v1 array	Alelle frequencies
Candoshi	Amazon	Peruvian Genome Project	17	Illumina HumanOmni2.5-8v1 array	Alelle frequencies, ADMIXTURE
Chachapoyas	Amazon	Peruvian Genome Project	46	Illumina HumanOmni2.5-8v1 array	Alelle frequencies, ADMIXTURE
Chopccas	Andean	Peruvian Genome Project	19	Illumina HumanOmni2.5-8v1 array	Alelle frequencies, ADMIXTURE, FST
Cusco	Admixed Peruvian	Peruvian Genome Project	37	Illumina HumanOmni2.5-8v1 array	Alelle frequencies
Iquitos	Admixed	Peruvian	31	Illumina	Alelle frequencies

POP	Group	DataSet	N	Data Type	Analysis
	Peruvian	Genome Project		HumanOmni2.5-8v1 array	
Jacarus	Andean	Peruvian Genome Project	21	Illumina HumanOmni2.5-8v1 array	Alelle frequencies, ADMIXTURE
Lamas	Amazon	Peruvian Genome Project	22	Illumina HumanOmni2.5-8v1 array	Alelle frequencies, ADMIXTURE, FST
Lambayeque	Admixed Peruvian	Peruvian Genome Project	12	Illumina HumanOmni2.5-8v1 array	Alelle frequencies
Lima	Admixed Peruvian	Peruvian Genome Project	29	Illumina HumanOmni2.5-8v1 array	Alelle frequencies
Matses	Amazon	Peruvian Genome Project	11	Illumina HumanOmni2.5-8v1 array	Alelle frequencies, ADMIXTURE, FST
Moche	Coast	Peruvian Genome Project	37	Illumina HumanOmni2.5-8v1 array	Alelle frequencies, ADMIXTURE, FST
Moquegua	Admixed Peruvian	Peruvian Genome Project	33	Illumina HumanOmni2.5-8v1 array	Alelle frequencies
Puno	Admixed Peruvian	Peruvian Genome Project	40	Illumina HumanOmni2.5-8v1 array	Alelle frequencies
Qeros	Andean	Peruvian Genome Project	12	Illumina HumanOmni2.5-8v1 array	Alelle frequencies, ADMIXTURE, FST
Shipibo	Amazon	Peruvian Genome Project	16	Illumina HumanOmni2.5-8v1 array	Alelle frequencies, ADMIXTURE
Tacna	Admixed Peruvian	Peruvian Genome Project	31	Illumina HumanOmni2.5-8v1 array	Alelle frequencies
Tallanes	Coast	Peruvian Genome Project	40	Illumina HumanOmni2.5-8v1 array	Alelle frequencies, ADMIXTURE, FST
Trujillo	Admixed Peruvian	Peruvian Genome Project	30	Illumina HumanOmni2.5-8v1 array	Alelle frequencies
Tumbes	Admixed Peruvian	Peruvian Genome Project	33	Illumina HumanOmni2.5-8v1 array	Alelle frequencies
Uros	Andean	Peruvian Genome Project	17	Illumina HumanOmni2.5-8v1 array	Alelle frequencies, ADMIXTURE, FST
Chopccas	Andean	Peruvian Genome Project	30	WGS	LD
Cusco	Admixed Peruvian	Peruvian Genome Project	16	WGS	LD
Iquitos	Admixed Peruvian	Peruvian Genome Project	16	WGS	LD
Matzes	Amazon	Peruvian Genome Project	12	WGS	LD
Moches	Coast	Peruvian	30	WGS	LD

POP	Group	DataSet	N	Data Type	Analysis
		Genome Project			
Trujillo	Admixed Peruvian	Peruvian Genome Project	16	WGS	LD
Uros	Andean	Peruvian Genome Project	30	WGS	LD
Arica y Tarapacá	Andean	ChileGenomico Project	9	WGS	Allele frequencies
Santiago	Mapuche	ChileGenomico Project	9	WGS	Allele frequencies
Southern Chile	Patagonian	PatogoniaDNA Project	17	WGS	Allele frequencies

ACB, African Caribbeans in Barbados; ASW, Americans of African Ancestry in SW USA; BEB, Bengali from Bangladesh; CDX, Chinese Dai in Xishuangbanna, China; CEU, Utah Residents(CEPH) with Northern and Western European Ancestry; CHB, Han Chinese in Beijing, China; CHS,Southern Han Chinese; CLM, Colombians from Medellin, Colombia; ESN, Esan in Nigeria; FIN, Finnish in Finland; GBR, British in England and Scotland; GIH, Gujarati Indian from Houston, Texas; GWD, Gambian in Western Divisions in the Gambia; IBS, Iberian Population in Spain; ITU, Indian Telugu from the UK; JPT,Japanese in Tokyo, Japan; KHV, Kinh in Ho Chi Minh City, Vietnam; LWK, Luhya in Webuye, Kenya; MSL, Mende in Sierra Leone; MXL, Mexican Ancestry from Los Angeles USA; PEL, Peruvians from Lima, Peru; PJL, Punjabi from Lahore, Pakistan; PUR, Puerto Ricans from Puerto Rico; STU, Sri Lankan Tamil from the UK; TSI, Toscani in Italia; YRI, Yoruba in Ibadan, Nigeria; AFR, African; AMR, Admixed American; EAS, East Asian; EUR, European; SAS, South Asia;

LDGH, Laboratory of Human Genomic Diversity;

WGS, Whole Genome Sequences;

LD, Linkage Disequilibrium berween rs2070788 and rs383510 ;
