# Supplementary material for: Human-SARS-CoV-2 interactome and human genetic diversity: TMPRSS2-rs2070788, associated with severe influenza, and its population genetics caveats in Native Americans
Source: Genet Mol Biol. 2021 Aug 25;44(1 Suppl 1):e20200484. doi: 10.1590/1678-4685-GMB-2020-0484 (PMC8387978; doi:10.1590/1678-4685-GMB-2020-0484)
Supplement: Table S2-A - [file 1415-4757-GMB-44-1-s1-e20200484-s4.pdf]

Genetics and Molecular Biology
https://doi.org/10.1590/1678-4685-GMB-2020-0484

Supplementary Material to "Human-SARS-CoV-2 interactome and human genetic diversity: *TMPRSS2*-rs2070788, associated with severe influenza, and its population genetics caveats in Native Americans"

Table S2-A – *ACE2* allele frequencies. Bold = functionally relevant SNPs found in our databases; NA = missing data.

SNP	Allele	ACB	Afro	Aimaras	Ancash	Arequipa	Ashaninkas	ASW	Awajun	Ayacucho	Bambui	BEB	Candoshi	CDX	CEU	Chachapoyas	CHB	Chileans	Chopccas	CHS	CLM
**rs4646123**	**T**	**0.08966**	**NA**	**NA**	**NA**	**NA**	**NA**	**0.05208**	**NA**	**NA**	**NA**	**0**	**NA**	**0**	**0**	**NA**	**0**	**NA**	**NA**	**0**	**0.01379**
**rs147311723**	**A**	**0.01379**	**NA**	**NA**	**NA**	**NA**	**NA**	**0**	**NA**	**NA**	**NA**	**0**	**NA**	**0**	**0**	**NA**	**0**	**NA**	**NA**	**0**	**0**
**rs4646188**	**G**	**0.006897**	**NA**	**NA**	**NA**	**NA**	**NA**	**0.01042**	**NA**	**NA**	**0.04965**	**0.05385**	**NA**	**0**	**0.08725**	**NA**	**0**	**NA**	**NA**	**0**	**0.03448**
**rs1514283**	**C**	**0.3379**	**0.3333**	**0**	**0**	**0.05263**	**NA**	**0.2396**	**0**	**0**	**0.06383**	**0.04615**	**0**	**0.0493**	**0**	**0**	**0.0375**	**NA**	**0**	**0.02532**	**0.02069**
**rs4646156**	**A**	**0.2**	**NA**	**NA**	**NA**	**NA**	**NA**	**0.1771**	**NA**	**NA**	**0.7535**	**0.1615**	**NA**	**0.02113**	**0.4027**	**NA**	**0.00625**	**0**	**NA**	**0**	**0.2966**
**rs714205**	**G**	**0.1241**	**NA**	**NA**	**NA**	**NA**	**NA**	**0.1771**	**NA**	**NA**	**0.2042**	**0.5308**	**NA**	**0.5634**	**0.255**	**NA**	**0.4938**	**0.6667**	**NA**	**0.5696**	**0.2897**
**rs233575**	**G**	**0.006897**	**NA**	**NA**	**NA**	**NA**	**NA**	**0.07292**	**NA**	**NA**	**0.1972**	**0.1538**	**NA**	**0.01408**	**0.3624**	**NA**	**0.00625**	**0**	**NA**	**0**	**0.3241**
**rs2074192**	**T**	**0.3517**	**0.2821**	**0.4783**	**0.3385**	**0.6316**	**0.3636**	**0.4062**	**0.5**	**0.4**	**0.4397**	**0.2462**	**0.5417**	**0.3732**	**0.349**	**0.5467**	**0.4625**	**0.7692**	**0.4483**	**0.4051**	**0.3379**
**rs4646174**	**G**	**0.4828**	**NA**	**NA**	**NA**	**NA**	**NA**	**0.5833**	**NA**	**NA**	**0.3262**	**0.7846**	**NA**	**0.93662**	**0.6174**	**NA**	**0.95625**	**1**	**NA**	**0.97468**	**0.6414**
**rs1514282**	**C**	**0.3379**	**NA**	**NA**	**NA**	**NA**	**NA**	**0.2292**	**NA**	**NA**	**NA**	**0.04615**	**NA**	**0.0493**	**0**	**NA**	**0.0375**	**NA**	**NA**	**0.02532**	**0.02069**
**rs4646181**	**T**	**0.01379**	**NA**	**NA**	**NA**	**NA**	**NA**	**0**	**NA**	**NA**	**NA**	**0**	**NA**	**0**	**0**	**NA**	**0**	**NA**	**NA**	**0**	**0**
**rs4646176**	**G**	**0.1862**	**NA**	**NA**	**NA**	**NA**	**NA**	**0.1146**	**NA**	**NA**	**NA**	**0.04615**	**NA**	**0.0493**	**0**	**NA**	**0.0375**	**NA**	**NA**	**0.02532**	**0.01379**
**rs1978124**	**T**	**0.06207**	**NA**	**NA**	**NA**	**NA**	**NA**	**0.1562**	**NA**	**NA**	**0.3732**	**0.1846**	**NA**	**0.02113**	**0.5168**	**NA**	**0.00625**	**0.02941**	**NA**	**0**	**0.3793**
**rs6632677**	**C**	**0**	**NA**	**NA**	**NA**	**NA**	**NA**	**0.01042**	**NA**	**NA**	**NA**	**0.02308**	**NA**	**0.03521**	**0.006711**	**NA**	**0.09375**	**NA**	**NA**	**0.08228**	**0.03448**
**rs4646171**	**C**	**0.1862**	**NA**	**NA**	**NA**	**NA**	**NA**	**0.1146**	**NA**	**NA**	**0.0493**	**0.04615**	**NA**	**0.0493**	**0**	**NA**	**0.0375**	**NA**	**NA**	**0.02532**	**0.01379**
**rs879922**	**G**	**0.4828**	**NA**	**NA**	**NA**	**NA**	**NA**	**0.5833**	**NA**	**NA**	**0.3239**	**0.7846**	**NA**	**0.93662**	**0.6107**	**NA**	**0.95625**	**1**	**NA**	**0.97468**	**0.6345**
**rs2106809**	**G**	**0.1241**	**NA**	**NA**	**NA**	**NA**	**NA**	**0.125**	**NA**	**NA**	**0.2113**	**0.5308**	**NA**	**0.507**	**0.2617**	**NA**	**0.5188**	**0.75**	**NA**	**0.557**	**0.3379**
**rs4646142**	**C**	**0.2414**	**NA**	**NA**	**NA**	**NA**	**NA**	**0.2604**	**NA**	**NA**	**NA**	**0.5538**	**NA**	**0.5493**	**0.2617**	**NA**	**0.5125**	**0.6667**	**NA**	**0.557**	**0.3655**
**rs4646155**	**T**	**0.1034**	**NA**	**NA**	**NA**	**NA**	**NA**	**0.1042**	**NA**	**NA**	**NA**	**0.04615**	**NA**	**0.0493**	**0**	**NA**	**0.0375**	**NA**	**NA**	**0.02532**	**0.01379**
**rs4240157**	**T**	**0.4828**	**0.5**	**0.7826**	**0.8806**	**0.8421**	**1**	**0.5833**	**0.7778**	**0.8727**	**0.6714**	**0.7769**	**0.95833**	**0.93662**	**0.6174**	**0.8933**	**0.95625**	**1**	**0.8667**	**0.97468**	**0.6345**
**rs2285666**	**T**	**0.2069**	**0.3333**	**NA**	**0.5224**	**0.2281**	**NA**	**0.2396**	**0.2778**	**0.4909**	**0.2057**	**0.5538**	**0.4583**	**0.5493**	**0.2617**	**0.3514**	**0.5125**	**NA**	**0.4**	**0.557**	**0.3655**
**rs2158083**	**C**	**0.1724**	**NA**	**NA**	**NA**	**NA**	**NA**	**0.1667**	**NA**	**NA**	**0.2465**	**0.1615**	**NA**	**0.02113**	**0.4094**	**NA**	**0.00625**	**0**	**NA**	**0**	**0.2966**
rs147417432	A	0	NA	NA	NA	NA	NA	0	NA	NA	NA	0	NA	0	0	NA	0	NA	NA	0	0
rs60122685	T	0.006897	NA	NA	NA	NA	NA	0.05208	NA	NA	NA	0	NA	0	0	NA	0	NA	NA	0	0
rs190342052	T	0.01379	NA	NA	NA	NA	NA	0	NA	NA	NA	0	NA	0	0	NA	0	NA	NA	0	0
rs188037336	T	0.00	NA	NA	NA	NA	NA	0	NA	NA	NA	0	NA	0	0	NA	0.00625	NA	NA	0	0
rs4646163	T	0.04828	NA	NA	NA	NA	NA	0	NA	NA	NA	0	NA	0	0	NA	0	NA	NA	0	0
rs191860450	C	0	NA	NA	NA	NA	NA	0	NA	NA	NA	0	NA	0	0	NA	0.00625	NA	NA	0	0
rs4646114	T	0.04828	NA	NA	NA	NA	NA	0.02083	NA	NA	NA	0	NA	0	0	NA	0	NA	NA	0	0.006897
rs4646145	G	0.1172	NA	NA	NA	NA	NA	0.07292	NA	NA	NA	0	NA	0	0	NA	0	NA	NA	0	0.006897

SNP	Allele	ACB	Afro	Aimaras	Ancash	Arequipa	Ashaninkas	ASW	Awajun	Ayacucho	Bambui	BEB	Candoshi	CDX	CEU	Chachapoyas	CHB	Chileans	Chopccas	CHS	CLM
rs73195520	T	0	NA	NA	NA	NA	NA	0.01042	NA	NA	NA	0	NA	0	0.01342	NA	0	NA	NA	0	0
rs188007597	C	0	NA	NA	NA	NA	NA	0	NA	NA	NA	0	NA	0	0	NA	0	NA	NA	0.006329	0
rs188895175	A	0	NA	NA	NA	NA	NA	0	NA	NA	NA	0	NA	0	0	NA	0	NA	NA	0	0
rs149683072	C	0	NA	NA	NA	NA	NA	0	NA	NA	NA	0.007692	NA	0	0	NA	0	NA	NA	0	0
rs201159862	C	0	NA	NA	NA	NA	NA	0	NA	NA	NA	0	NA	0	0	NA	0	NA	NA	0	0
rs141562322	A	0.01379	NA	NA	NA	NA	NA	0.02083	NA	NA	NA	0	NA	0	0	NA	0	NA	NA	0	0
rs182809041	C	0	NA	NA	NA	NA	NA	0	NA	NA	NA	0	NA	0	0	NA	0	NA	NA	0	0
rs190327760	T	0	NA	NA	NA	NA	NA	0	NA	NA	NA	0	NA	0	0	NA	0	NA	NA	0	0
rs4646165	T	0.04828	NA	NA	NA	NA	NA	0.03125	NA	NA	NA	0	NA	0	0	NA	0	NA	NA	0	0.006897
rs187045475	C	0	NA	NA	NA	NA	NA	0	NA	NA	NA	0	NA	0	0	NA	0	NA	NA	0	0
rs370596467	C	0	NA	NA	NA	NA	NA	0	NA	NA	NA	0	NA	0.007042	0	NA	0	NA	NA	0	0
rs4646138	T	0.06207	NA	NA	NA	NA	NA	0.04167	NA	NA	NA	0	NA	0	0	NA	0	NA	NA	0	0.006897
rs4646184	A	0.05517	0.03846	0	0	0	0	0.05208	0	0	0.02113	0	0	0	0	0	0	NA	0	0	0.01379
rs73195521	A	0	NA	NA	NA	NA	NA	0.01042	NA	NA	NA	0	NA	0	0.01342	NA	0	NA	NA	0	0
rs4646175	C	0.02759	NA	NA	NA	NA	NA	0.04167	NA	NA	NA	0	NA	0	0	NA	0	NA	NA	0	0.006897
rs4646119	G	0.131	NA	NA	NA	NA	NA	0.07292	NA	NA	NA	0	NA	0	0	NA	0	NA	NA	0	0.006897
rs183452730	T	0	NA	NA	NA	NA	NA	0	NA	NA	NA	0	NA	0	0	NA	0	NA	NA	0.006329	0
rs150550484	C	0	NA	NA	NA	NA	NA	0	NA	NA	NA	0	NA	0.007042	0	NA	0.00625	NA	NA	0.006329	0
rs187860487	A	0	NA	NA	NA	NA	NA	0	NA	NA	NA	0	NA	0	0	NA	0	NA	NA	0	0
rs114165600	A	0.01379	NA	NA	NA	NA	NA	0.03125	NA	NA	NA	0	NA	0	0	NA	0	NA	NA	0	0
rs137910448	C	0	NA	NA	NA	NA	NA	0	NA	NA	NA	0	NA	0.03521	0	NA	0.0625	NA	NA	0.03797	0
rs187504060	C	0	NA	NA	NA	NA	NA	0	NA	NA	NA	0	NA	0	0	NA	0	NA	NA	0	0
rs182246050	T	0	NA	NA	NA	NA	NA	0	NA	NA	NA	0	NA	0	0	NA	0	NA	NA	0	0
rs2316904	C	0.08276	NA	NA	NA	NA	NA	0.09375	NA	NA	NA	0.1615	NA	0.02113	0.4094	NA	0.00625	0	NA	0	0.2966
rs4646141	A	0.02759	NA	NA	NA	NA	NA	0.04167	NA	NA	NA	0	NA	0	0	NA	0	NA	NA	0	0.006897
rs4646154	T	0.006897	NA	NA	NA	NA	NA	0.01042	NA	NA	NA	0	NA	0	0	NA	0	NA	NA	0	0
rs149434207	T	0	0	0	0	0	0	0	0	0	0.007042	0	0	0	0.02013	0.01333	0	NA	0	0	0
rs183135788	C	0	NA	NA	NA	NA	NA	0	NA	NA	NA	0	NA	0	0	NA	0	NA	NA	0.01266	0
rs193237066	A	0	NA	NA	NA	NA	NA	0	NA	NA	NA	0	NA	0	0	NA	0	NA	NA	0	0.006897
rs199569050	A	0	NA	NA	NA	NA	NA	0	NA	NA	NA	0	NA	0	0	NA	0	NA	NA	0	0
rs4646159	T	0.09655	NA	NA	NA	NA	NA	0.0625	NA	NA	NA	0	NA	0	0	NA	0	NA	NA	0	0.006897
rs4646112	T	0.04138	NA	NA	NA	NA	NA	0.02083	NA	NA	NA	0	NA	0	0	NA	0	NA	NA	0	0.006897
rs190373279	T	0	NA	NA	NA	NA	NA	0	NA	NA	NA	0	NA	0	0	NA	0	NA	NA	0	0
rs2048684	A	0.05517	NA	NA	NA	NA	NA	0.09375	NA	NA	0.2357	0.1615	NA	0.02113	0.4094	NA	0.00625	0	NA	0	0.2966
rs190406475	A	0	NA	NA	NA	NA	NA	0	NA	NA	NA	0	NA	0	0	NA	0	NA	NA	0.006329	0
rs183156483	A	0	NA	NA	NA	NA	NA	0	NA	NA	NA	0	NA	0	0	NA	0	NA	NA	0	0
rs111572878	C	0.2276	NA	NA	NA	NA	NA	0.125	NA	NA	NA	0	NA	0	0	NA	0	NA	NA	0	0.02069
rs4646148	T	0.08276	NA	NA	NA	NA	NA	0.09375	NA	NA	NA	0.1615	NA	0.02113	0.4027	NA	0.00625	0	NA	0	0.2897
rs190384874	A	0.006897	NA	NA	NA	NA	NA	0	NA	NA	NA	0	NA	0	0	NA	0	NA	NA	0	0

SNP	Allele	ACB	Afro	Aimaras	Ancash	Arequipa	Ashaninkas	ASW	Awajun	Ayacucho	Bambui	BEB	Candoshi	CDX	CEU	Chachapoyas	CHB	Chileans	Chopccas	CHS	CLM
rs183576751	G	0	NA	NA	NA	NA	NA	0	NA	NA	NA	0	NA	0	0	NA	0	NA	NA	0	0
rs150909162	A	0	NA	NA	NA	NA	NA	0	NA	NA	NA	0	NA	0	0.006711	NA	0	NA	NA	0	0
rs201489317	G	0	NA	NA	NA	NA	NA	0	NA	NA	NA	0	NA	0.05634	0	NA	0.04375	NA	NA	0.01899	0
rs146750287	G	0.006897	NA	NA	NA	NA	NA	0.02083	NA	NA	NA	0	NA	0	0	NA	0	NA	NA	0	0
rs181161610	C	0	NA	NA	NA	NA	NA	0	NA	NA	NA	0	NA	0	0	NA	0.00625	NA	NA	0.006329	0
rs193159186	A	0	NA	NA	NA	NA	NA	0	NA	NA	NA	0	NA	0	0.006711	NA	0	NA	NA	0	0
rs4646144	A	0.1034	NA	NA	NA	NA	NA	0.1042	NA	NA	NA	0.04615	NA	0.0493	0	NA	0.0375	NA	NA	0.02532	0.01379
rs146122606	A	0.09655	NA	NA	NA	NA	NA	0.1562	NA	NA	NA	0.1615	NA	0.02113	0.4027	NA	0.00625	NA	NA	0.006329	0.2966
rs183655025	C	0	NA	NA	NA	NA	NA	0	NA	NA	NA	0	NA	0	0	NA	0	NA	NA	0	0
rs200973492	G	0	NA	NA	NA	NA	NA	0	NA	NA	NA	0	NA	0	0	NA	0	NA	NA	0	0
rs182858184	A	0	NA	NA	NA	NA	NA	0	NA	NA	NA	0	NA	0	0.006711	NA	0	NA	NA	0	0
rs181159881	G	0	NA	NA	NA	NA	NA	0	NA	NA	NA	0	NA	0	0	NA	0	NA	NA	0	0
rs233574	T	0.05517	NA	NA	NA	NA	NA	0.1042	NA	NA	NA	0.1538	NA	0.01408	0.3557	NA	0.00625	0	NA	0	0.3241
rs4646118	T	0.09655	NA	NA	NA	NA	NA	0.05208	NA	NA	NA	0	NA	0	0	NA	0	NA	NA	0	0.01379
rs2316903	G	0.08276	NA	NA	NA	NA	NA	0.09375	NA	NA	0.2286	0.1615	NA	0.02113	0.4027	NA	0.00625	0	NA	0	0.2966
rs187991938	G	0.01379	NA	NA	NA	NA	NA	0.02083	NA	NA	NA	0	NA	0	0	NA	0	NA	NA	0	0
rs192781231	C	0	NA	NA	NA	NA	NA	0	NA	NA	NA	0	NA	0	0	NA	0	NA	NA	0	0
rs4646185	T	0.03448	NA	NA	NA	NA	NA	0	NA	NA	NA	0	NA	0	0	NA	0	NA	NA	0	0
rs188959000	T	0.01379	NA	NA	NA	NA	NA	0.01042	NA	NA	NA	0	NA	0	0	NA	0	NA	NA	0	0
rs141554379	C	0.4897	NA	NA	NA	NA	NA	0.4271	NA	NA	NA	0.3	NA	0.1761	0.4497	NA	0.1438	0.1875	NA	0.1329	0.4276
rs147464721	A	0	NA	NA	NA	NA	NA	0	NA	NA	NA	0	NA	0	0	NA	0	NA	NA	0	0.006897
rs182777784	A	0	NA	NA	NA	NA	NA	0	NA	NA	NA	0	NA	0	0	NA	0.00625	NA	NA	0	0
rs191640680	T	0	NA	NA	NA	NA	NA	0.01042	NA	NA	NA	0	NA	0	0	NA	0	NA	NA	0	0
rs186597267	A	0	NA	NA	NA	NA	NA	0	NA	NA	NA	0	NA	0	0	NA	0	NA	NA	0	0
rs146598386	T	0	NA	NA	NA	NA	NA	0.01042	NA	NA	NA	0	NA	0	0.01342	NA	0	NA	NA	0	0
rs4646131	GA	0.2	NA	NA	NA	NA	NA	0.1667	NA	NA	NA	0.1615	NA	0.02113	0.4027	NA	0.00625	NA	NA	0	0.2966
rs145693360	T	0	NA	NA	NA	NA	NA	0	NA	NA	NA	0	NA	0.01408	0	NA	0.00625	NA	NA	0.006329	0
rs373114523	C	0	NA	NA	NA	NA	NA	0	NA	NA	NA	0	NA	0	0	NA	0	NA	NA	0	0
rs184680265	A	0	NA	NA	NA	NA	NA	0	NA	NA	NA	0	NA	0	0	NA	0	NA	NA	0	0
rs140473595	T	0	NA	NA	NA	NA	NA	0	NA	NA	NA	0	NA	0	0	NA	0.00625	NA	NA	0	0
rs191611107	A	0	NA	NA	NA	NA	NA	0	NA	NA	NA	0	NA	0	0	NA	0	NA	NA	0	0.01379
rs190147327	G	0	NA	NA	NA	NA	NA	0	NA	NA	NA	0	NA	0	0	NA	0	NA	NA	0	0
rs185202584	C	0	NA	NA	NA	NA	NA	0	NA	NA	NA	0	NA	0	0	NA	0	NA	NA	0	0.03448
rs4646115	C	0.01379	NA	NA	NA	NA	NA	0	NA	NA	NA	0	NA	0	0	NA	0	NA	NA	0	0
rs190614788	T	0	NA	NA	NA	NA	NA	0	NA	NA	NA	0	NA	0	0.02013	NA	0	NA	NA	0	0
rs367784090	A	0	NA	NA	NA	NA	NA	0	NA	NA	NA	0	NA	0	0	NA	0	NA	NA	0	0
rs59021449	A	0	NA	NA	NA	NA	NA	0	NA	NA	NA	0	NA	0	0	NA	0	NA	NA	0	0
rs143779699	T	0	NA	NA	NA	NA	NA	0	NA	NA	NA	0	NA	0	0	NA	0	NA	NA	0	0
rs141025655	T	0.02759	NA	NA	NA	NA	NA	0	NA	NA	NA	0	NA	0	0	NA	0	NA	NA	0	0

SNP	Allele	ACB	Afro	Aimaras	Ancash	Arequipa	Ashaninkas	ASW	Awajun	Ayacucho	Bambui	BEB	Candoshi	CDX	CEU	Chachapoyas	CHB	Chileans	Chopccas	CHS	CLM
rs4646122	G	0.2483	NA	NA	NA	NA	NA	0.1354	NA	NA	NA	0	NA	0	0	NA	0	NA	NA	0	0.01379
rs1514279	G	0.2	NA	NA	NA	NA	NA	0.1667	NA	NA	0.2465	0.1615	NA	0.02113	0.4094	NA	0.00625	0	NA	0	0.3034
rs191513620	C	0	NA	NA	NA	NA	NA	0	NA	NA	NA	0	NA	0	0	NA	0	NA	NA	0	0
rs201900069	T	0	NA	NA	NA	NA	NA	0	NA	NA	NA	0	NA	0	0	NA	0.00625	NA	NA	0	0
rs4646161	G	0.02759	NA	NA	NA	NA	NA	0.04167	NA	NA	NA	0	NA	0	0	NA	0	NA	NA	0	0.006897
rs4646170	C	0.1034	NA	NA	NA	NA	NA	0.0625	NA	NA	0.02857	0	NA	0	0	NA	0	NA	NA	0	0.006897
rs139797356	G	0	NA	NA	NA	NA	NA	0	NA	NA	NA	0	NA	0	0	NA	0	NA	NA	0	0
rs188512350	A	0	NA	NA	NA	NA	NA	0	NA	NA	NA	0	NA	0	0.006711	NA	0	NA	NA	0	0
rs183322022	T	0	NA	NA	NA	NA	NA	0	NA	NA	NA	0	NA	0	0	NA	0	NA	NA	0	0
rs4646166	T	0	NA	NA	NA	NA	NA	0.02083	NA	NA	NA	0	NA	0	0	NA	0	NA	NA	0	0
rs183411746	T	0	NA	NA	NA	NA	NA	0.01042	NA	NA	NA	0	NA	0	0	NA	0	NA	NA	0	0
rs4646152	A	0.05517	NA	NA	NA	NA	NA	0.09375	NA	NA	0.2324	0.1615	NA	0.02113	0.4094	NA	0.00625	0	NA	0	0.2966
rs188271923	C	0	NA	NA	NA	NA	NA	0	NA	NA	NA	0	NA	0	0	NA	0	NA	NA	0	0
rs182847340	G	0	NA	NA	NA	NA	NA	0	NA	NA	NA	0	NA	0	0.006711	NA	0	NA	NA	0	0
rs188556473	T	0	NA	NA	NA	NA	NA	0	NA	NA	NA	0	NA	0	0	NA	0	NA	NA	0	0
rs4646137	T	0.01379	NA	NA	NA	NA	NA	0.02083	NA	NA	NA	0	NA	0	0	NA	0	NA	NA	0	0.01379
rs193035703	T	0	NA	NA	NA	NA	NA	0	NA	NA	NA	0	NA	0	0	NA	0	NA	NA	0	0
rs372629764	C	0	NA	NA	NA	NA	NA	0	NA	NA	NA	0	NA	0	0	NA	0	NA	NA	0	0
rs187300691	T	0	NA	NA	NA	NA	NA	0	NA	NA	NA	0	NA	0	0	NA	0	NA	NA	0	0
rs141377908	G	0.006897	NA	NA	NA	NA	NA	0.02083	NA	NA	NA	0	NA	0	0	NA	0	NA	NA	0	0
rs192715876	A	0	NA	NA	NA	NA	NA	0	NA	NA	NA	0	NA	0	0	NA	0	NA	NA	0	0.006897
rs202137736	C	0	NA	NA	NA	NA	NA	0	NA	NA	NA	0	NA	0.007042	0	NA	0	NA	NA	0.006329	0
rs187846474	C	0	NA	NA	NA	NA	NA	0	NA	NA	NA	0	NA	0	0	NA	0	NA	NA	0	0
rs183880501	C	0	NA	NA	NA	NA	NA	0	NA	NA	NA	0	NA	0	0.006711	NA	0	NA	NA	0	0.01379
rs191961425	T	0.006897	NA	NA	NA	NA	NA	0	NA	NA	NA	0	NA	0	0	NA	0	NA	NA	0	0.006897
rs143459173	C	0	NA	NA	NA	NA	NA	0	NA	NA	NA	0	NA	0	0	NA	0	NA	NA	0	0
rs187467963	T	0	NA	NA	NA	NA	NA	0	NA	NA	NA	0	NA	0	0	NA	0	NA	NA	0	0
rs184459616	A	0	NA	NA	NA	NA	NA	0	NA	NA	NA	0	NA	0	0	NA	0	NA	NA	0	0
rs148805807	T	0.04138	NA	NA	NA	NA	NA	0.01042	NA	NA	NA	0	NA	0	0	NA	0	NA	NA	0	0.006897
rs199804629	T	0	NA	NA	NA	NA	NA	0	NA	NA	NA	0	NA	0	0	NA	0	NA	NA	0	0
rs138390800	C	0.006897	NA	NA	NA	NA	NA	0	NA	NA	NA	0	NA	0	0	NA	0	NA	NA	0	0
rs1514281	C	0.3379	NA	NA	NA	NA	NA	0.2292	NA	NA	NA	0.04615	NA	0.0493	0	NA	0.0375	NA	NA	0.02532	0.02069
rs148408803	T	0	NA	NA	NA	NA	NA	0	NA	NA	NA	0	NA	0	0.02013	NA	0	NA	NA	0	0
rs181799530	T	0.006897	NA	NA	NA	NA	NA	0	NA	NA	NA	0	NA	0	0	NA	0	NA	NA	0	0
rs192479337	C	0	NA	NA	NA	NA	NA	0	NA	NA	NA	0	NA	0	0	NA	0	NA	NA	0	0.02069
rs181964653	A	0	NA	NA	NA	NA	NA	0	NA	NA	NA	0	NA	0	0	NA	0	NA	NA	0	0
rs113691336	C	0.05517	NA	NA	NA	NA	NA	0.09375	NA	NA	NA	0.1615	NA	0.02113	0.4027	NA	0.00625	0	NA	0	0.2897
rs184756568	T	0	NA	NA	NA	NA	NA	0	NA	NA	NA	0	NA	0	0	NA	0	NA	NA	0	0
rs184453641	A	0	NA	NA	NA	NA	NA	0	NA	NA	NA	0	NA	0	0	NA	0	NA	NA	0	0

SNP	Allele	ACB	Afro	Aimaras	Ancash	Arequipa	Ashaninkas	ASW	Awajun	Ayacucho	Bambui	BEB	Candoshi	CDX	CEU	Chachapoyas	CHB	Chileans	Chopccas	CHS	CLM
rs188558468	A	0	NA	NA	NA	NA	NA	0	NA	NA	NA	0	NA	0	0	NA	0	NA	NA	0	0
rs2301692	C	0.1517	NA	NA	NA	NA	NA	0.1146	NA	NA	NA	0.04615	NA	0.0493	0	NA	0.0375	NA	NA	0.02532	0.01379
rs189821013	A	0	NA	NA	NA	NA	NA	0	NA	NA	NA	0	NA	0.03521	0	NA	0.00625	NA	NA	0	0
rs189699255	T	0	NA	NA	NA	NA	NA	0	NA	NA	NA	0	NA	0	0	NA	0	NA	NA	0	0
rs4646153	C	0.05517	NA	NA	NA	NA	NA	0.09375	NA	NA	0.2324	0.1615	NA	0.02113	0.4094	NA	0.00625	0	NA	0	0.2966
rs182802402	C	0	NA	NA	NA	NA	NA	0	NA	NA	NA	0	NA	0.04225	0	NA	0.00625	NA	NA	0.006329	0
rs186905240	T	0.006897	NA	NA	NA	NA	NA	0	NA	NA	NA	0	NA	0	0	NA	0	NA	NA	0	0
rs181989524	C	0	NA	NA	NA	NA	NA	0	NA	NA	NA	0	NA	0	0	NA	0	NA	NA	0	0
rs142060377	A	0	NA	NA	NA	NA	NA	0	NA	NA	0	0	NA	0	0	NA	0	NA	NA	0	0
rs79473368	T	0	NA	NA	NA	NA	NA	0	NA	NA	NA	0	NA	0	0	NA	0	NA	NA	0	0
rs73635825	G	0	NA	NA	NA	NA	NA	0	NA	NA	NA	0	NA	0	0	NA	0	NA	NA	0	0
rs141605946	T	0	NA	NA	NA	NA	NA	0	NA	NA	NA	0	NA	0	0.006711	NA	0	NA	NA	0	0
rs185220883	A	0	NA	NA	NA	NA	NA	0	NA	NA	NA	0	NA	0	0	NA	0	NA	NA	0	0
rs191099160	A	0	NA	NA	NA	NA	NA	0	NA	NA	NA	0	NA	0	0	NA	0	NA	NA	0	0
rs4646169	A	0.01379	NA	NA	NA	NA	NA	0	NA	NA	0	0	NA	0	0	NA	0	NA	NA	0	0
rs187738069	A	0	NA	NA	NA	NA	NA	0	NA	NA	NA	0	NA	0	0	NA	0	NA	NA	0	0.006897
rs184589032	T	0	NA	NA	NA	NA	NA	0	NA	NA	NA	0	NA	0	0.006711	NA	0	NA	NA	0	0
rs34481900	C	0.1724	NA	NA	NA	NA	NA	0.1667	NA	NA	NA	0.1615	NA	0.02113	0.4027	NA	0.00625	NA	NA	0	0.2966
rs188991352	C	0	NA	NA	NA	NA	NA	0	NA	NA	NA	0	NA	0	0.006711	NA	0	NA	NA	0	0
rs201835187	AC	0	NA	NA	NA	NA	NA	0	NA	NA	NA	0.007692	NA	0	0.01342	NA	0	NA	NA	0	0
rs187161283	G	0	NA	NA	NA	NA	NA	0.01042	NA	NA	NA	0	NA	0	0	NA	0	NA	NA	0	0
rs35803318	T	0.006897	0.07692	0.2174	0.1045	0.07018	0	0	0.2222	0.1273	0.04225	0	0.04167	0	0.04698	0.09333	0	NA	0.1333	0	0.06897
rs143079256	T	0.01379	NA	NA	NA	NA	NA	0.03125	NA	NA	NA	0	NA	0	0	NA	0	NA	NA	0	0.006897
rs189944937	G	0	NA	NA	NA	NA	NA	0	NA	NA	NA	0	NA	0	0	NA	0	NA	NA	0	0
rs4646147	T	0.08276	NA	NA	NA	NA	NA	0.09375	NA	NA	NA	0.1615	NA	0.02113	0.4094	NA	0.00625	0	NA	0	0.2966
rs190133379	T	0	NA	NA	NA	NA	NA	0	NA	NA	NA	0	NA	0	0	NA	0	NA	NA	0	0.006897
rs144523173	A	0	NA	NA	NA	NA	NA	0	NA	NA	NA	0	NA	0	0	NA	0.00625	NA	NA	0.006329	0
rs145502291	T	0	NA	NA	NA	NA	NA	0	NA	NA	NA	0	NA	0	0	NA	0	NA	NA	0	0
rs149958080	A	0	NA	NA	NA	NA	NA	0	NA	NA	NA	0	NA	0.01408	0	NA	0.00625	NA	NA	0.006329	0
rs4646186	A	0.04138	NA	NA	NA	NA	NA	0.03125	NA	NA	NA	0	NA	0	0	NA	0	NA	NA	0	0.006897
rs189142411	A	0	NA	NA	NA	NA	NA	0	NA	NA	NA	0	NA	0	0	NA	0	NA	NA	0	0
rs4646127	A	0.1724	NA	NA	NA	NA	NA	0.1667	NA	NA	NA	0.1615	NA	0.02113	0.4027	NA	0.00625	0	NA	0	0.2966
rs189140113	A	0	NA	NA	NA	NA	NA	0.01042	NA	NA	NA	0	NA	0	0.006711	NA	0	NA	NA	0	0
rs4646143	T	0.08276	NA	NA	NA	NA	NA	0.09375	NA	NA	NA	0.1615	NA	0.02113	0.4094	NA	0.00625	0	NA	0	0.2966
rs41303171	C	0	NA	NA	NA	NA	NA	0	NA	NA	0.02113	0	NA	0	0.02013	NA	0	NA	NA	0	0.006897
rs182808331	G	0	NA	NA	NA	NA	NA	0	NA	NA	NA	0	NA	0	0	NA	0	NA	NA	0	0
rs971249	T	0.2	NA	NA	NA	NA	NA	0.1667	NA	NA	0.2465	0.1615	NA	0.02113	0.4094	NA	0.00625	0	NA	0	0.3103
rs186029035	A	0	NA	NA	NA	NA	NA	0.01042	NA	NA	NA	0	NA	0	0	NA	0	NA	NA	0	0
rs184978092	G	0	NA	NA	NA	NA	NA	0	NA	NA	NA	0	NA	0	0	NA	0	NA	NA	0	0

SNP	Allele	ACB	Afro	Aimaras	Ancash	Arequipa	Ashaninkas	ASW	Awajun	Ayacucho	Bambui	BEB	Candoshi	CDX	CEU	Chachapoyas	CHB	Chileans	Chopccas	CHS	CLM
rs192180091	C	0	NA	NA	NA	NA	NA	0	NA	NA	NA	0	NA	0	0	NA	0	NA	NA	0	0
rs34161673	C	0	NA	NA	NA	NA	NA	0	NA	NA	NA	0	NA	0	0.006711	NA	0	NA	NA	0	0
rs188496534	C	0	NA	NA	NA	NA	NA	0.01042	NA	NA	NA	0	NA	0	0	NA	0	NA	NA	0	0
rs193005774	T	0.006897	NA	NA	NA	NA	NA	0.01042	NA	NA	NA	0	NA	0	0	NA	0	NA	NA	0	0
rs182372519	G	0	NA	NA	NA	NA	NA	0	NA	NA	NA	0	NA	0	0	NA	0	NA	NA	0	0
rs151205966	G	0	NA	NA	NA	NA	NA	0	NA	NA	NA	0	NA	0	0	NA	0.0125	NA	NA	0	0
rs182550682	A	0	NA	NA	NA	NA	NA	0	NA	NA	NA	0	NA	0	0	NA	0	NA	NA	0	0
rs191332175	G	0.006897	NA	NA	NA	NA	NA	0	NA	NA	NA	0	NA	0	0	NA	0	NA	NA	0	0.006897
rs144061872	T	0	NA	NA	NA	NA	NA	0	NA	NA	NA	0	NA	0	0.006711	NA	0	NA	NA	0	0
rs189571339	C	0	NA	NA	NA	NA	NA	0	NA	NA	NA	0	NA	0	0	NA	0	NA	NA	0	0
rs138940089	T	0.006897	NA	NA	NA	NA	NA	0	NA	NA	NA	0	NA	0	0	NA	0	NA	NA	0	0
rs190870013	C	0	NA	NA	NA	NA	NA	0.01042	NA	NA	NA	0	NA	0	0	NA	0	NA	NA	0	0
rs188290800	C	0	NA	NA	NA	NA	NA	0	NA	NA	NA	0	NA	0	0	NA	0	NA	NA	0	0
rs185622388	A	0	NA	NA	NA	NA	NA	0	NA	NA	NA	0	NA	0	0	NA	0	NA	NA	0	0
rs190055656	T	0	NA	NA	NA	NA	NA	0	NA	NA	NA	0	NA	0	0	NA	0	NA	NA	0	0
rs138854040	A	0	NA	NA	NA	NA	NA	0.01042	NA	NA	NA	0.02308	NA	0.03521	0.006711	NA	0.09375	NA	NA	0.08228	0.03448
rs4646182	C	0.05517	NA	NA	NA	NA	NA	0.05208	NA	NA	NA	0	NA	0	0	NA	0	NA	NA	0	0.01379
rs181796402	T	0	NA	NA	NA	NA	NA	0	NA	NA	NA	0	NA	0	0	NA	0	NA	NA	0	0
rs182366225	T	0	NA	NA	NA	NA	NA	0	NA	NA	NA	0	NA	0.02113	0	NA	0.00625	NA	NA	0.01899	0
rs4646151	A	0.04138	NA	NA	NA	NA	NA	0.02083	NA	NA	0.02113	0	NA	0	0	NA	0	NA	NA	0	0.006897
rs145890723	A	0.01379	NA	NA	NA	NA	NA	0.02083	NA	NA	NA	0	NA	0	0	NA	0	NA	NA	0	0
rs191536933	T	0	NA	NA	NA	NA	NA	0	NA	NA	NA	0	NA	0	0	NA	0	NA	NA	0.006329	0
rs151005246	A	0	NA	NA	NA	NA	NA	0	NA	NA	NA	0.01538	NA	0.01408	0	NA	0.01875	NA	NA	0.01266	0
rs182219706	T	0	NA	NA	NA	NA	NA	0	NA	NA	NA	0	NA	0	0	NA	0	NA	NA	0	0
rs200217737	CA	0.07586	NA	NA	NA	NA	NA	0.04167	NA	NA	NA	0.007692	NA	0	0.006711	NA	0	NA	NA	0.01266	0.006897
rs375635972	T	0	NA	NA	NA	NA	NA	0	NA	NA	NA	0	NA	0	0	NA	0	NA	NA	0	0
rs4646172	GA	0.03448	NA	NA	NA	NA	NA	0.05208	NA	NA	NA	0	NA	0	0	NA	0	NA	NA	0	0.006897
rs183661335	G	0	NA	NA	NA	NA	NA	0	NA	NA	NA	0	NA	0	0	NA	0	NA	NA	0	0
rs181695790	G	0	NA	NA	NA	NA	NA	0	NA	NA	NA	0	NA	0	0	NA	0.00625	NA	NA	0	0
rs188283144	C	0	NA	NA	NA	NA	NA	0	NA	NA	NA	0	NA	0	0	NA	0	NA	NA	0	0
rs199951323	C	0	NA	NA	NA	NA	NA	0	NA	NA	NA	0	NA	0	0	NA	0	NA	NA	0	0
rs181788868	C	0	NA	NA	NA	NA	NA	0	NA	NA	NA	0	NA	0	0	NA	0	NA	NA	0	0
rs72614595	T	0	NA	NA	NA	NA	NA	0.01042	NA	NA	NA	0	NA	0	0	NA	0	NA	NA	0	0
rs182287132	A	0	NA	NA	NA	NA	NA	0	NA	NA	NA	0	NA	0	0	NA	0	NA	NA	0	0
rs181522566	C	0	NA	NA	NA	NA	NA	0	NA	NA	NA	0	NA	0	0	NA	0	NA	NA	0.006329	0
rs4646120	G	0.2897	NA	NA	NA	NA	NA	0.2812	NA	NA	NA	0.1846	NA	0.02113	0.5235	NA	0.00625	0.03125	NA	0	0.4
rs4646177	T	0.08276	NA	NA	NA	NA	NA	0.0625	NA	NA	NA	0	NA	0	0	NA	0	NA	NA	0	0
rs371025504	G	0.006897	NA	NA	NA	NA	NA	0	NA	NA	NA	0	NA	0	0	NA	0	NA	NA	0	0
rs185525294	A	0	NA	NA	NA	NA	NA	0	NA	NA	NA	0	NA	0	0	NA	0	NA	NA	0	0

SNP	Allele	ACB	Afro	Aimaras	Ancash	Arequipa	Ashaninkas	ASW	Awajun	Ayacucho	Bambui	BEB	Candoshi	CDX	CEU	Chachapoyas	CHB	Chileans	Chopccas	CHS	CLM
rs189278377	A	0.006897	NA	NA	NA	NA	NA	0	NA	NA	NA	0	NA	0	0	NA	0	NA	NA	0	0
rs4646183	C	0	NA	NA	NA	NA	NA	0	NA	NA	NA	0	NA	0	0	NA	0	NA	NA	0	0
rs188292876	C	0	NA	NA	NA	NA	NA	0	NA	NA	NA	0	NA	0	0	NA	0	NA	NA	0	0
rs186957036	C	0.006897	NA	NA	NA	NA	NA	0.01042	NA	NA	NA	0	NA	0	0	NA	0	NA	NA	0	0
rs4646121	T	0.06897	NA	NA	NA	NA	NA	0.05208	NA	NA	NA	0	NA	0	0	NA	0	NA	NA	0	0.01379
rs143102000	A	0.03448	NA	NA	NA	NA	NA	0.01042	NA	NA	NA	0	NA	0	0	NA	0	NA	NA	0	0.006897
rs185005111	T	0	NA	NA	NA	NA	NA	0	NA	NA	NA	0	NA	0	0	NA	0	NA	NA	0	0
rs113208650	T	0.131	0.05128	0	0	0.03509	0	0.07292	0	0	0.01408	0	0	0	0	0	0	NA	0	0	0.006897
rs186914723	A	0	NA	NA	NA	NA	NA	0	NA	NA	NA	0	NA	0	0.006711	NA	0	NA	NA	0	0
rs4646150	T	0	NA	NA	NA	NA	NA	0	NA	NA	NA	0	NA	0	0	NA	0.00625	0.5	NA	0	0.1241
rs4646135	C	0.1448	NA	NA	NA	NA	NA	0.07292	NA	NA	0.01408	0	NA	0	0	NA	0	NA	NA	0	0.006897
rs184064003	A	0	NA	NA	NA	NA	NA	0	NA	NA	NA	0	NA	0	0	NA	0	NA	NA	0	0
rs4646187	A	0	NA	NA	NA	NA	NA	0	NA	NA	NA	0	NA	0	0.006711	NA	0	NA	NA	0	0
rs185723767	T	0	NA	NA	NA	NA	NA	0	NA	NA	NA	0	NA	0	0	NA	0	NA	NA	0.006329	0
rs376237761	G	0	NA	NA	NA	NA	NA	0	NA	NA	NA	0	NA	0.007042	0	NA	0	NA	NA	0	0
rs373723722	T	0	NA	NA	NA	NA	NA	0	NA	NA	NA	0	NA	0.02817	0	NA	0	NA	NA	0	0
rs61433707	T	0.01379	NA	NA	NA	NA	NA	0.01042	NA	NA	0	0	NA	0.01408	0	NA	0.025	NA	NA	0.01266	0
rs201424204	C	0	NA	NA	NA	NA	NA	0.02083	NA	NA	NA	0.01538	NA	0.08451	0	NA	0.075	NA	NA	0.08228	0
rs146087218	T	0	NA	NA	NA	NA	NA	0	NA	NA	NA	0	NA	0	0	NA	0	NA	NA	0	0
rs2301693	A	0.1517	NA	NA	NA	NA	NA	0.1146	NA	NA	NA	0.04615	NA	0.0493	0	NA	0.0375	NA	NA	0.02532	0.01379
rs186036909	T	0	NA	NA	NA	NA	NA	0	NA	NA	NA	0	NA	0	0	NA	0	NA	NA	0	0
rs191884180	G	0	NA	NA	NA	NA	NA	0	NA	NA	NA	0	NA	0	0	NA	0	NA	NA	0.006329	0
rs148977224	C	0	NA	NA	NA	NA	NA	0	NA	NA	NA	0	NA	0	0	NA	0	NA	NA	0.006329	0
rs190850356	A	0	NA	NA	NA	NA	NA	0	NA	NA	NA	0	NA	0	0.006711	NA	0	NA	NA	0	0
rs192474956	G	0	NA	NA	NA	NA	NA	0	NA	NA	NA	0	NA	0	0	NA	0.025	NA	NA	0	0
rs56700224	T	0.1172	NA	NA	NA	NA	NA	0.07292	NA	NA	NA	0	NA	0	0	NA	0	NA	NA	0	0.006897
rs142017934	C	0.02759	NA	NA	NA	NA	NA	0.01042	NA	NA	NA	0	NA	0	0	NA	0	NA	NA	0	0
rs41297301	T	0	0	NA	0	0.01754	0	0	0	0	0	0	0	0	0.02013	0	0	NA	0	0	0
rs4646168	G	0.1172	NA	NA	NA	NA	NA	0.0625	NA	NA	0.02817	0	NA	0	0	NA	0	NA	NA	0	0.006897
rs2048683	T	0.2	0.1667	0.2174	0.1045	0.1404	0	0.1667	0.2222	0.1273	0.2465	0.1615	0.04167	0.02113	0.4094	0.09589	0.00625	0	0.1333	0	0.3034
rs181786494	A	0	NA	NA	NA	NA	NA	0	NA	NA	NA	0	NA	0	0	NA	0	NA	NA	0	0
rs4646124	T	0.2138	NA	NA	NA	NA	NA	0.1667	NA	NA	0.2465	0.1615	NA	0.02113	0.4027	NA	0.00625	0	NA	0	0.3034
rs138529167	A	0.006897	NA	NA	NA	NA	NA	0.02083	NA	NA	NA	0	NA	0	0	NA	0	NA	NA	0	0
rs4646125	T	0.02759	NA	NA	NA	NA	NA	0	NA	NA	NA	0	NA	0	0	NA	0	NA	NA	0	0
rs4646162	G	0.04828	NA	NA	NA	NA	NA	0	NA	NA	NA	0	NA	0	0	NA	0	NA	NA	0	0
rs180864908	G	0	NA	NA	NA	NA	NA	0	NA	NA	NA	0	NA	0	0	NA	0	NA	NA	0	0.006897
rs184178697	T	0	NA	NA	NA	NA	NA	0.02083	NA	NA	NA	0	NA	0	0	NA	0	NA	NA	0	0
rs145954042	C	0	NA	NA	NA	NA	NA	0	NA	NA	NA	0	NA	0	0	NA	0	NA	NA	0	0
rs4646149	T	0.01379	NA	NA	NA	NA	NA	0	NA	NA	NA	0	NA	0	0	NA	0	NA	NA	0	0

SNP	Allele	ACB	Afro	Aimaras	Ancash	Arequipa	Ashaninkas	ASW	Awajun	Ayacucho	Bambui	BEB	Candoshi	CDX	CEU	Chachapoyas	CHB	Chileans	Chopccas	CHS	CLM
rs147312210	C	0.006897	NA	NA	NA	NA	NA	0.01042	NA	NA	NA	0	NA	0	0	NA	0	NA	NA	0	0
rs149039346	G	0.02759	NA	NA	NA	NA	NA	0	NA	NA	NA	0	NA	0	0	NA	0	NA	NA	0	0
rs192674337	G	0	NA	NA	NA	NA	NA	0	NA	NA	NA	0	NA	0	0	NA	0	NA	NA	0	0
rs193290621	A	0	NA	NA	NA	NA	NA	0	NA	NA	NA	0	NA	0	0	NA	0.00625	NA	NA	0.006329	0
rs187390194	G	0	NA	NA	NA	NA	NA	0	NA	NA	NA	0	NA	0	0	NA	0.0125	NA	NA	0	0
rs4646128	T	0.09655	NA	NA	NA	NA	NA	0.0625	NA	NA	NA	0	NA	0	0	NA	0	NA	NA	0	0.006897
rs4646133	G	0.02759	NA	NA	NA	NA	NA	0	NA	NA	NA	0	NA	0	0	NA	0	NA	NA	0	0
rs181620291	C	0.006897	NA	NA	NA	NA	NA	0.01042	NA	NA	NA	0	NA	0	0	NA	0	NA	NA	0	0
rs182501431	G	0	NA	NA	NA	NA	NA	0	NA	NA	NA	0	NA	0	0	NA	0	NA	NA	0	0
rs184503057	T	0	NA	NA	NA	NA	NA	0	NA	NA	NA	0	NA	0	0	NA	0	NA	NA	0	0
rs181169181	C	0	NA	NA	NA	NA	NA	0	NA	NA	NA	0	NA	0	0	NA	0	NA	NA	0	0
rs4646129	T	0.1172	NA	NA	NA	NA	NA	0.07292	NA	NA	NA	0	NA	0	0	NA	0	NA	NA	0	0.006897
rs185182583	A	0	NA	NA	NA	NA	NA	0	NA	NA	NA	0	NA	0	0	NA	0	NA	NA	0	0
rs140083333	A	0	NA	NA	NA	NA	NA	0	NA	NA	NA	0	NA	0	0	NA	0	NA	NA	0	0
rs4646146	C	0.09655	NA	NA	NA	NA	NA	0.0625	NA	NA	NA	0	NA	0	0	NA	0	NA	NA	0	0.006897
rs186271730	T	0	NA	NA	NA	NA	NA	0	NA	NA	NA	0	NA	0	0	NA	0	NA	NA	0	0
rs189283596	C	0	NA	NA	NA	NA	NA	0	NA	NA	NA	0	NA	0	0	NA	0	NA	NA	0	0
rs189944736	C	0	NA	NA	NA	NA	NA	0	NA	NA	NA	0	NA	0	0	NA	0	NA	NA	0	0
rs148006212	A	0.02759	NA	NA	NA	NA	NA	0	NA	NA	NA	0	NA	0	0	NA	0	NA	NA	0	0
rs372812138	T	0	NA	NA	NA	NA	NA	0	NA	NA	NA	0	NA	0.007042	0	NA	0	NA	NA	0.006329	0
rs4646173	A	0.03448	NA	NA	NA	NA	NA	0	NA	NA	NA	0	NA	0	0	NA	0	NA	NA	0	0
rs4646117	C	0	NA	NA	NA	NA	NA	0.01042	NA	NA	NA	0	NA	0	0	NA	0	NA	NA	0	0
rs187959864	T	0	NA	NA	NA	NA	NA	0	NA	NA	NA	0	NA	0	0	NA	0	NA	NA	0	0
rs191869625	A	0.006897	NA	NA	NA	NA	NA	0	NA	NA	NA	0	NA	0	0.006711	NA	0	NA	NA	0	0.01379
rs200885467	TA	0	NA	NA	NA	NA	NA	0	NA	NA	NA	0	NA	0	0.01342	NA	0	NA	NA	0	0
rs72614596	A	0	NA	NA	NA	NA	NA	0.02083	NA	NA	NA	0	NA	0.07042	0	NA	0.05625	0.6667	NA	0.06962	0
rs185721534	G	0.09655	NA	NA	NA	NA	NA	0.05208	NA	NA	NA	0	NA	0	0	NA	0	NA	NA	0	0.01379
rs183546232	G	0	NA	NA	NA	NA	NA	0	NA	NA	NA	0	NA	0	0.01342	NA	0	NA	NA	0	0
rs757066	C	0.006897	NA	NA	NA	NA	NA	0.07292	NA	NA	0.2042	0.1615	NA	0.02113	0.4094	NA	0.00625	0	NA	0	0.2897
rs186277162	C	0	NA	NA	NA	NA	NA	0	NA	NA	NA	0	NA	0	0	NA	0.00625	NA	NA	0.01266	0
rs4646157	C	0.08276	NA	NA	NA	NA	NA	0.07292	NA	NA	NA	0	NA	0	0	NA	0	NA	NA	0	0.006897
rs2023802	G	0.2	NA	NA	NA	NA	NA	0.1667	NA	NA	0.2465	0.1615	NA	0.02113	0.4027	NA	0.00625	0	NA	0	0.2966
rs200260858	C	0.006897	NA	NA	NA	NA	NA	0	NA	NA	NA	0	NA	0	0.006711	NA	0.0125	NA	NA	0	0
rs191336106	G	0.006897	NA	NA	NA	NA	NA	0	NA	NA	NA	0	NA	0	0	NA	0	NA	NA	0	0
rs150200247	A	0	NA	NA	NA	NA	NA	0	NA	NA	NA	0	NA	0.007042	0	NA	0	NA	NA	0	0
rs187474513	T	0.01379	NA	NA	NA	NA	NA	0.02083	NA	NA	NA	0	NA	0	0	NA	0	NA	NA	0	0
rs184874220	A	0	NA	NA	NA	NA	NA	0	NA	NA	NA	0	NA	0	0	NA	0.00625	NA	NA	0	0
rs189810201	C	0	NA	NA	NA	NA	NA	0	NA	NA	NA	0	NA	0	0	NA	0	NA	NA	0	0
rs184746393	C	0	NA	NA	NA	NA	NA	0	NA	NA	NA	0	NA	0	0	NA	0	NA	NA	0	0

SNP	Allele	ACB	Afro	Aimaras	Ancash	Arequipa	Ashaninkas	ASW	Awajun	Ayacucho	Bambui	BEB	Candoshi	CDX	CEU	Chachapoyas	CHB	Chileans	Chopccas	CHS	CLM
rs72614598	C	0	NA	NA	NA	NA	NA	0.02083	NA	NA	NA	0	NA	0.07042	0	NA	0.05625	0.6667	NA	0.06962	0
rs180878567	C	0.006897	NA	NA	NA	NA	NA	0	NA	NA	NA	0	NA	0	0	NA	0	NA	NA	0	0
rs200477770	G	0	NA	NA	NA	NA	NA	0	NA	NA	NA	0	NA	0	0	NA	0	NA	NA	0	0
rs199612962	G	0	NA	NA	NA	NA	NA	0	NA	NA	NA	0	NA	0	0	NA	0	NA	NA	0	0
rs200180615	T	0	NA	NA	NA	NA	NA	0	NA	NA	NA	0	NA	0	0	NA	0.00625	NA	NA	0	0
rs192914427	T	0	NA	NA	NA	NA	NA	0	NA	NA	NA	0	NA	0	0	NA	0	NA	NA	0	0
rs181390351	T	0	NA	NA	NA	NA	NA	0	NA	NA	NA	0	NA	0	0	NA	0	NA	NA	0.006329	0
rs4646178	G	0.08966	NA	NA	NA	NA	NA	0.0625	NA	NA	NA	0	NA	0	0	NA	0	NA	NA	0	0
rs187251131	C	0	NA	NA	NA	NA	NA	0	NA	NA	NA	0	NA	0	0	NA	0.00625	NA	NA	0	0
rs192692847	C	0	NA	NA	NA	NA	NA	0	NA	NA	NA	0	NA	0	0	NA	0	NA	NA	0.006329	0
rs4646167	T	0.04828	NA	NA	NA	NA	NA	0.03125	NA	NA	NA	0	NA	0	0	NA	0	NA	NA	0	0.006897
rs189691652	T	0.006897	NA	NA	NA	NA	NA	0	NA	NA	NA	0	NA	0	0	NA	0	NA	NA	0	0.006897
rs138763015	G	0	NA	NA	NA	NA	NA	0	NA	NA	NA	0	NA	0	0	NA	0.025	NA	NA	0.006329	0
rs372272603	A	0	NA	NA	NA	NA	NA	0	NA	NA	NA	0	NA	0	0	NA	0	NA	NA	0	0
rs184730109	A	0	NA	NA	NA	NA	NA	0	NA	NA	NA	0	NA	0	0	NA	0	NA	NA	0	0
rs186261546	C	0	NA	NA	NA	NA	NA	0	NA	NA	NA	0	NA	0	0	NA	0	NA	NA	0	0

Table S2-A – *ACE2* allele frequencies continuation. Bold = functionally relevant SNPs found in our databases; NA = missing data.

SNP	Allele	Cusco	ESN	FIN	GBR	GIH	GWD	IBS	Iquitos	ITU	Jacarus	JPT	KHV	Lamas	Lambayeque	Lima	LWK	Matses	Moche	Moquegua	MSL
**rs4646123**	**T**	**NA**	**0.07639**	**0**	**0**	**0**	**0.2105**	**0**	**NA**	**0**	**NA**	**0**	**0**	**NA**	**NA**	**NA**	**0.07843**	**NA**	**NA**	**NA**	**0.1562**
**rs147311723**	**A**	**NA**	**0.01389**	**0**	**0**	**0**	**0**	**0**	**NA**	**0**	**NA**	**0**	**0**	**NA**	**NA**	**NA**	**0.01307**	**NA**	**NA**	**NA**	**0.03125**
**rs4646188**	**G**	**NA**	**0**	**0.1312**	**0.2059**	**0.07333**	**0**	**0.1375**	**NA**	**0.05517**	**NA**	**0**	**0**	**NA**	**NA**	**NA**	**0**	**NA**	**NA**	**NA**	**0**
**rs1514283**	**C**	**0**	**0.3056**	**0**	**0.007353**	**0.09333**	**0.2515**	**0**	**0**	**0.1103**	**0**	**0.02632**	**0.02632**	**0**	**0**	**0.08511**	**0.3268**	**0**	**0**	**0**	**0.2656**
**rs4646156**	**A**	**NA**	**0.1667**	**0.3312**	**0.2868**	**0.2333**	**0.1871**	**0.3375**	**NA**	**0.1724**	**NA**	**0**	**0.006579**	**NA**	**NA**	**NA**	**0.2418**	**NA**	**NA**	**NA**	**0.1719**
**rs714205**	**G**	**NA**	**0.1042**	**0.2**	**0.1985**	**0.4067**	**0.1053**	**0.2125**	**NA**	**0.5103**	**NA**	**0.5461**	**0.5066**	**NA**	**NA**	**NA**	**0.08497**	**NA**	**NA**	**NA**	**0.07031**
**rs233575**	**G**	**NA**	**0**	**0.2938**	**0.2721**	**0.1933**	**0.005848**	**0.3438**	**NA**	**0.1724**	**NA**	**0**	**0**	**NA**	**NA**	**NA**	**0.01961**	**NA**	**NA**	**NA**	**0**
**rs2074192**	**T**	**0.4746**	**0.3194**	**0.4625**	**0.5**	**0.24**	**0.2924**	**0.4062**	**0.4068**	**0.1793**	**0.6053**	**0.4276**	**0.4671**	**0.5**	**0.5556**	**0.3617**	**0.4183**	**0.2308**	**0.2923**	**0.5**	**0.3594**
**rs4646174**	**G**	**NA**	**0.4375**	**0.6688**	**0.7206**	**0.68**	**0.4035**	**0.6438**	**NA**	**0.7103**	**NA**	**0.97368**	**0.97368**	**NA**	**NA**	**NA**	**0.5098**	**NA**	**NA**	**NA**	**0.4453**
**rs1514282**	**C**	**NA**	**0.3125**	**0**	**0.007353**	**0.09333**	**0.2632**	**0**	**NA**	**0.1103**	**NA**	**0.02632**	**0.02632**	**NA**	**NA**	**NA**	**0.3268**	**NA**	**NA**	**NA**	**0.2812**
**rs4646181**	**T**	**NA**	**0.01389**	**0**	**0**	**0**	**0**	**0.00625**	**NA**	**0**	**NA**	**0**	**0**	**NA**	**NA**	**NA**	**0.01307**	**NA**	**NA**	**NA**	**0.03125**
**rs4646176**	**G**	**NA**	**0.1806**	**0**	**0**	**0.09333**	**0.1813**	**0**	**NA**	**0.1103**	**NA**	**0.02632**	**0.02632**	**NA**	**NA**	**NA**	**0.09804**	**NA**	**NA**	**NA**	**0.1641**
**rs1978124**	**T**	**NA**	**0.1111**	**0.4812**	**0.4118**	**0.2733**	**0.08187**	**0.425**	**NA**	**0.1655**	**NA**	**0**	**0.006579**	**NA**	**NA**	**NA**	**0.1046**	**NA**	**NA**	**NA**	**0.07031**
**rs6632677**	**C**	**NA**	**0**	**0**	**0**	**0**	**0**	**0**	**NA**	**0**	**NA**	**0.09868**	**0.02632**	**NA**	**NA**	**NA**	**0**	**NA**	**NA**	**NA**	**0**
**rs4646171**	**C**	**NA**	**0.1806**	**0**	**0**	**0.09333**	**0.193**	**0**	**NA**	**0.1103**	**NA**	**0.02632**	**0.02632**	**NA**	**NA**	**NA**	**0.1046**	**NA**	**NA**	**NA**	**0.1641**
**rs879922**	**G**	**NA**	**0.4375**	**0.6625**	**0.7206**	**0.68**	**0.4035**	**0.6438**	**NA**	**0.7103**	**NA**	**0.97368**	**0.97368**	**NA**	**NA**	**NA**	**0.5098**	**NA**	**NA**	**NA**	**0.4453**
**rs2106809**	**G**	**NA**	**0.04167**	**0.2375**	**0.2574**	**0.3867**	**0.09357**	**0.275**	**NA**	**0.5379**	**NA**	**0.5132**	**0.5132**	**NA**	**NA**	**NA**	**0.07843**	**NA**	**NA**	**NA**	**0.1016**
**rs4646142**	**C**	**NA**	**0.2708**	**0.2562**	**0.2353**	**0.4**	**0.3099**	**0.25**	**NA**	**0.5172**	**NA**	**0.5461**	**0.5263**	**NA**	**NA**	**NA**	**0.1699**	**NA**	**NA**	**NA**	**0.2422**

SNP	Allele	Cusco	ESN	FIN	GBR	GIH	GWD	IBS	Iquitos	ITU	Jacarus	JPT	KHV	Lamas	Lambayeque	Lima	LWK	Matses	Moche	Moquegua	MSL
**rs4646155**	**T**	**NA**	**0.1667**	**0**	**0**	**0.09333**	**0.1579**	**0**	**NA**	**0.1103**	**NA**	**0.02632**	**0.02632**	**NA**	**NA**	**NA**	**0.0915**	**NA**	**NA**	**NA**	**0.1094**
**rs4240157**	**T**	**0.8305**	**0.4375**	**0.6438**	**0.7059**	**0.6867**	**0.4094**	**0.65**	**0.91525**	**0.7103**	**0.7895**	**0.97368**	**0.97368**	**0.97368**	**0.94444**	**0.7234**	**0.5163**	**0.8462**	**0.7692**	**0.8148**	**0.4453**
**rs2285666**	**T**	**0.4407**	**0.1806**	**0.2562**	**0.2206**	**0.4**	**0.3041**	**0.25**	**0.4915**	**0.5172**	**0.3684**	**0.5461**	**0.5197**	**0.5**	**0.4444**	**0.383**	**0.1634**	**0.6154**	**0.4769**	**0.3333**	**0.2109**
**rs2158083**	**C**	**NA**	**0.1667**	**0.3375**	**0.2868**	**0.2333**	**0.1579**	**0.3375**	**NA**	**0.1586**	**NA**	**0**	**0.006579**	**NA**	**NA**	**NA**	**0.2288**	**NA**	**NA**	**NA**	**0.125**
rs147417432	A	NA	0	0	0	0	0	0	NA	0	NA	0.01316	0	NA	NA	NA	0	NA	NA	NA	0
rs60122685	T	NA	0.01389	0	0	0	0.005848	0	NA	0	NA	0	0	NA	NA	NA	0.006536	NA	NA	NA	0.007812
rs190342052	T	NA	0	0	0	0	0	0	NA	0	NA	0	0	NA	NA	NA	0	NA	NA	NA	0
rs188037336	T	NA	0	0	0	0	0	0	NA	0	NA	0	0	NA	NA	NA	0	NA	NA	NA	0
rs4646163	T	NA	0.01389	0	0	0	0.02924	0	NA	0	NA	0	0	NA	NA	NA	0.02614	NA	NA	NA	0.07812
rs191860450	C	NA	0	0	0	0	0	0	NA	0	NA	0.006579	0	NA	NA	NA	0	NA	NA	NA	0
rs4646114	T	NA	0.09722	0	0	0	0.07018	0	NA	0	NA	0	0	NA	NA	NA	0.05882	NA	NA	NA	0.07031
rs4646145	G	NA	0.0625	0	0.007353	0	0.07018	0	NA	0	NA	0	0	NA	NA	NA	0.1503	NA	NA	NA	0.03906
rs73195520	T	NA	0	0.00625	0.007353	0	0	0	NA	0	NA	0	0	NA	NA	NA	0	NA	NA	NA	0
rs188007597	C	NA	0	0	0	0	0	0	NA	0	NA	0.01316	0.006579	NA	NA	NA	0	NA	NA	NA	0
rs188895175	A	NA	0.006944	0	0	0	0.0117	0	NA	0	NA	0	0	NA	NA	NA	0	NA	NA	NA	0.01562
rs149683072	C	NA	0	0.01875	0.01471	0	0	0	NA	0	NA	0	0	NA	NA	NA	0	NA	NA	NA	0
rs201159862	C	NA	0	0	0	0	0	0	NA	0	NA	0	0	NA	NA	NA	0	NA	NA	NA	0
rs141562322	A	NA	0.02083	0	0.007353	0	0.005848	0	NA	0	NA	0	0	NA	NA	NA	0.01307	NA	NA	NA	0.007812
rs182809041	C	NA	0	0	0	0	0	0	NA	0	NA	0	0	NA	NA	NA	0	NA	NA	NA	0
rs190327760	T	NA	0	0	0	0	0	0	NA	0	NA	0	0	NA	NA	NA	0.006536	NA	NA	NA	0
rs4646165	T	NA	0.09722	0	0	0	0.07018	0	NA	0	NA	0	0	NA	NA	NA	0.05882	NA	NA	NA	0.07031
rs187045475	C	NA	0	0	0	0	0	0	NA	0	NA	0	0	NA	NA	NA	0	NA	NA	NA	0
rs370596467	C	NA	0	0	0	0	0	0	NA	0.006897	NA	0	0	NA	NA	NA	0	NA	NA	NA	0
rs4646138	T	NA	0.03472	0	0	0	0.04094	0	NA	0	NA	0	0	NA	NA	NA	0.1046	NA	NA	NA	0.03125
rs4646184	A	0.01695	0.06944	0	0	0	0.2105	0	0	0	0	0	0	0	0	0	0.06536	0	0	0	0.1328
rs73195521	A	NA	0	0.00625	0.007353	0	0	0	NA	0	NA	0	0	NA	NA	NA	0	NA	NA	NA	0
rs4646175	C	NA	0.06944	0	0	0	0.09942	0	NA	0	NA	0	0	NA	NA	NA	0.02614	NA	NA	NA	0.08594
rs4646119	G	NA	0.05556	0	0.007353	0	0.09357	0	NA	0	NA	0	0	NA	NA	NA	0.1634	NA	NA	NA	0.08594
rs183452730	T	NA	0	0	0	0	0	0	NA	0	NA	0	0	NA	NA	NA	0	NA	NA	NA	0
rs150550484	C	NA	0	0	0	0	0	0	NA	0	NA	0	0.01316	NA	NA	NA	0	NA	NA	NA	0
rs187860487	A	NA	0	0	0.007353	0	0	0	NA	0	NA	0	0	NA	NA	NA	0	NA	NA	NA	0
rs114165600	A	NA	0.0625	0	0	0	0.01754	0	NA	0	NA	0	0	NA	NA	NA	0.01961	NA	NA	NA	0.02344
rs137910448	C	NA	0	0.0125	0	0.01333	0	0	NA	0	NA	0.06579	0.05921	NA	NA	NA	0	NA	NA	NA	0
rs187504060	C	NA	0	0	0	0	0	0	NA	0	NA	0	0	NA	NA	NA	0	NA	NA	NA	0
rs182246050	T	NA	0	0	0.02206	0	0	0.00625	NA	0	NA	0	0	NA	NA	NA	0	NA	NA	NA	0
rs2316904	C	NA	0.1042	0.3375	0.2794	0.2333	0.117	0.3375	NA	0.1586	NA	0	0.006579	NA	NA	NA	0.08497	NA	NA	NA	0.1328
rs4646141	A	NA	0.07639	0	0	0	0.09942	0	NA	0	NA	0	0	NA	NA	NA	0.02614	NA	NA	NA	0.07812
rs4646154	T	NA	0.01389	0	0	0	0	0	NA	0	NA	0	0	NA	NA	NA	0	NA	NA	NA	0.01562
rs149434207	T	0	0.006944	0	0	0	0.0117	0	0	0	0	0	0	0	0	0	0	0	0	0	0.01562

SNP	Allele	Cusco	ESN	FIN	GBR	GIH	GWD	IBS	Iquitos	ITU	Jacarus	JPT	KHV	Lamas	Lambayeque	Lima	LWK	Matses	Moche	Moquegua	MSL
rs183135788	C	NA	0	0	0	0	0	0	NA	0	NA	0	0	NA	NA	NA	0	NA	NA	NA	0
rs193237066	A	NA	0	0	0	0	0	0	NA	0	NA	0	0	NA	NA	NA	0	NA	NA	NA	0
rs199569050	A	NA	0	0	0	0	0	0	NA	0	NA	0	0	NA	NA	NA	0	NA	NA	NA	0
rs4646159	T	NA	0.04861	0	0	0	0.05848	0	NA	0	NA	0	0	NA	NA	NA	0.05882	NA	NA	NA	0.09375
rs4646112	T	NA	0.09722	0	0	0	0.07018	0	NA	0	NA	0	0	NA	NA	NA	0.05882	NA	NA	NA	0.07031
rs190373279	T	NA	0	0.00625	0	0	0	0	NA	0	NA	0	0	NA	NA	NA	0	NA	NA	NA	0
rs2048684	A	NA	0.1042	0.3375	0.2794	0.2333	0.08772	0.3375	NA	0.1586	NA	0	0.006579	NA	NA	NA	0.07843	NA	NA	NA	0.08594
rs190406475	A	NA	0	0	0	0	0	0	NA	0	NA	0	0	NA	NA	NA	0	NA	NA	NA	0
rs183156483	A	NA	0	0	0	0	0	0	NA	0	NA	0	0	NA	NA	NA	0	NA	NA	NA	0
rs111572878	C	NA	0.1319	0	0.007353	0	0.2924	0	NA	0	NA	0	0	NA	NA	NA	0.2288	NA	NA	NA	0.2266
rs4646148	T	NA	0.1042	0.3312	0.2794	0.2333	0.117	0.3375	NA	0.1586	NA	0	0.006579	NA	NA	NA	0.08497	NA	NA	NA	0.1328
rs190384874	A	NA	0.01389	0	0	0	0	0	NA	0	NA	0	0	NA	NA	NA	0.006536	NA	NA	NA	0.007812
rs183576751	G	NA	0	0	0	0	0	0	NA	0	NA	0	0	NA	NA	NA	0.006536	NA	NA	NA	0
rs150909162	A	NA	0	0	0	0	0	0	NA	0	NA	0	0	NA	NA	NA	0.006536	NA	NA	NA	0
rs201489317	G	NA	0	0	0	0	0	0	NA	0	NA	0.03289	0.05921	NA	NA	NA	0	NA	NA	NA	0
rs146750287	G	NA	0	0	0	0	0	0	NA	0	NA	0	0	NA	NA	NA	0.01961	NA	NA	NA	0
rs181161610	C	NA	0	0	0	0	0	0	NA	0	NA	0	0	NA	NA	NA	0	NA	NA	NA	0
rs193159186	A	NA	0	0	0	0	0	0	NA	0	NA	0	0	NA	NA	NA	0	NA	NA	NA	0
rs4646144	A	NA	0.1667	0	0	0.09333	0.1579	0	NA	0.1103	NA	0.02632	0.02632	NA	NA	NA	0.0915	NA	NA	NA	0.1172
rs146122606	A	NA	0.1528	0.3312	0.2868	0.2333	0.1345	0.3375	NA	0.1586	NA	0	0.006579	NA	NA	NA	0.1242	NA	NA	NA	0.1172
rs183655025	C	NA	0	0	0	0	0	0	NA	0	NA	0	0	NA	NA	NA	0	NA	NA	NA	0
rs200973492	G	NA	0	0	0	0	0	0	NA	0	NA	0	0	NA	NA	NA	0.006536	NA	NA	NA	0
rs182858184	A	NA	0	0	0	0	0	0	NA	0	NA	0	0	NA	NA	NA	0	NA	NA	NA	0
rs181159881	G	NA	0	0	0	0	0	0	NA	0	NA	0	0	NA	NA	NA	0	NA	NA	NA	0
rs233574	T	NA	0.09722	0.2875	0.2721	0.1933	0.07018	0.3438	NA	0.1724	NA	0	0	NA	NA	NA	0.07843	NA	NA	NA	0.07031
rs4646118	T	NA	0.06944	0	0	0	0.1871	0	NA	0	NA	0	0	NA	NA	NA	0.06536	NA	NA	NA	0.125
rs2316903	G	NA	0.1042	0.3375	0.2794	0.2333	0.117	0.3375	NA	0.1586	NA	0	0.006579	NA	NA	NA	0.08497	NA	NA	NA	0.1328
rs187991938	G	NA	0.02083	0	0.007353	0	0.005848	0	NA	0	NA	0	0	NA	NA	NA	0.02614	NA	NA	NA	0
rs192781231	C	NA	0.006944	0	0	0	0.0117	0	NA	0	NA	0	0	NA	NA	NA	0	NA	NA	NA	0.01562
rs4646185	T	NA	0	0	0	0	0.02924	0	NA	0	NA	0	0	NA	NA	NA	0.006536	NA	NA	NA	0.04688
rs188959000	T	NA	0.01389	0	0	0	0.005848	0	NA	0	NA	0	0	NA	NA	NA	0.01961	NA	NA	NA	0
rs141554379	C	NA	0.4792	0.3812	0.3382	0.4267	0.4444	0.4062	NA	0.3448	NA	0.1776	0.2039	NA	NA	NA	0.4706	NA	NA	NA	0.4062
rs147464721	A	NA	0	0	0	0	0.01754	0	NA	0	NA	0	0	NA	NA	NA	0	NA	NA	NA	0.007812
rs182777784	A	NA	0	0	0	0	0	0	NA	0	NA	0	0	NA	NA	NA	0	NA	NA	NA	0
rs191640680	T	NA	0	0	0	0	0	0	NA	0	NA	0	0	NA	NA	NA	0	NA	NA	NA	0
rs186597267	A	NA	0.006944	0	0	0	0.0117	0	NA	0	NA	0	0	NA	NA	NA	0	NA	NA	NA	0.01562
rs146598386	T	NA	0	0.00625	0.007353	0	0	0.0125	NA	0	NA	0	0	NA	NA	NA	0	NA	NA	NA	0
rs4646131	GA	NA	0.1667	0.3312	0.2868	0.2333	0.1871	0.3375	NA	0.1586	NA	0	0.006579	NA	NA	NA	0.2418	NA	NA	NA	0.1719
rs145693360	T	NA	0	0	0	0	0	0	NA	0	NA	0.006579	0	NA	NA	NA	0	NA	NA	NA	0

SNP	Allele	Cusco	ESN	FIN	GBR	GIH	GWD	IBS	Iquitos	ITU	Jacarus	JPT	KHV	Lamas	Lambayeque	Lima	LWK	Matses	Moche	Moquegua	MSL
rs373114523	C	NA	0	0	0	0	0	0	NA	0	NA	0	0.006579	NA	NA	NA	0	NA	NA	NA	0
rs184680265	A	NA	0	0	0	0	0	0	NA	0	NA	0.006579	0	NA	NA	NA	0	NA	NA	NA	0
rs140473595	T	NA	0	0	0	0	0	0	NA	0	NA	0	0	NA	NA	NA	0	NA	NA	NA	0
rs191611107	A	NA	0	0	0	0	0	0	NA	0	NA	0	0	NA	NA	NA	0	NA	NA	NA	0
rs190147327	G	NA	0	0	0	0	0.0117	0	NA	0	NA	0	0	NA	NA	NA	0	NA	NA	NA	0.01562
rs185202584	C	NA	0	0	0.007353	0	0	0.00625	NA	0	NA	0	0	NA	NA	NA	0	NA	NA	NA	0
rs4646115	C	NA	0	0	0	0	0.02339	0	NA	0	NA	0	0	NA	NA	NA	0.006536	NA	NA	NA	0.04688
rs190614788	T	NA	0	0	0.01471	0	0	0.01875	NA	0	NA	0	0	NA	NA	NA	0	NA	NA	NA	0
rs367784090	A	NA	0.006944	0	0	0	0	0	NA	0	NA	0	0	NA	NA	NA	0	NA	NA	NA	0
rs59021449	A	NA	0	0	0	0	0	0	NA	0	NA	0	0	NA	NA	NA	0	NA	NA	NA	0
rs143779699	T	NA	0	0	0	0	0	0	NA	0	NA	0.006579	0	NA	NA	NA	0	NA	NA	NA	0
rs141025655	T	NA	0	0	0	0	0.005848	0	NA	0	NA	0	0	NA	NA	NA	0.006536	NA	NA	NA	0
rs4646122	G	NA	0.09722	0	0.007353	0	0.1813	0	NA	0	NA	0	0	NA	NA	NA	0.2157	NA	NA	NA	0.1797
rs1514279	G	NA	0.1667	0.3375	0.2868	0.2333	0.1871	0.3375	NA	0.1586	NA	0	0.006579	NA	NA	NA	0.2353	NA	NA	NA	0.1719
rs191513620	C	NA	0	0	0	0	0	0	NA	0	NA	0	0	NA	NA	NA	0.006536	NA	NA	NA	0
rs201900069	T	NA	0	0	0	0	0	0	NA	0	NA	0	0	NA	NA	NA	0	NA	NA	NA	0
rs4646161	G	NA	0.07639	0	0	0	0.09942	0	NA	0	NA	0	0	NA	NA	NA	0.02614	NA	NA	NA	0.07812
rs4646170	C	NA	0.0625	0	0.007353	0	0.09942	0	NA	0	NA	0	0	NA	NA	NA	0.1699	NA	NA	NA	0.07812
rs139797356	G	NA	0	0	0	0	0	0	NA	0	NA	0	0	NA	NA	NA	0	NA	NA	NA	0
rs188512350	A	NA	0	0.0375	0	0.006667	0	0	NA	0.006897	NA	0	0	NA	NA	NA	0	NA	NA	NA	0
rs183322022	T	NA	0	0	0	0	0	0	NA	0	NA	0	0	NA	NA	NA	0	NA	NA	NA	0
rs4646166	T	NA	0.01389	0	0	0	0.0117	0	NA	0	NA	0	0	NA	NA	NA	0	NA	NA	NA	0.01562
rs183411746	T	NA	0	0	0	0	0	0	NA	0	NA	0	0	NA	NA	NA	0	NA	NA	NA	0
rs4646152	A	NA	0.1042	0.3312	0.2868	0.2333	0.08772	0.3375	NA	0.1586	NA	0	0.006579	NA	NA	NA	0.07843	NA	NA	NA	0.08594
rs188271923	C	NA	0	0	0	0	0	0	NA	0	NA	0	0	NA	NA	NA	0	NA	NA	NA	0
rs182847340	G	NA	0	0	0	0	0	0	NA	0	NA	0	0	NA	NA	NA	0	NA	NA	NA	0
rs188556473	T	NA	0	0	0	0	0	0	NA	0	NA	0	0	NA	NA	NA	0.006536	NA	NA	NA	0
rs4646137	T	NA	0	0	0	0	0.01754	0	NA	0	NA	0	0	NA	NA	NA	0	NA	NA	NA	0.02344
rs193035703	T	NA	0	0	0	0	0	0	NA	0	NA	0	0	NA	NA	NA	0.006536	NA	NA	NA	0
rs372629764	C	NA	0	0	0	0	0	0.00625	NA	0	NA	0	0	NA	NA	NA	0	NA	NA	NA	0
rs187300691	T	NA	0	0.00625	0.007353	0	0	0	NA	0	NA	0	0	NA	NA	NA	0	NA	NA	NA	0
rs141377908	G	NA	0	0	0	0	0	0	NA	0	NA	0	0	NA	NA	NA	0.01961	NA	NA	NA	0
rs192715876	A	NA	0	0	0	0	0	0	NA	0	NA	0	0	NA	NA	NA	0	NA	NA	NA	0
rs202137736	C	NA	0	0	0	0	0	0	NA	0	NA	0	0	NA	NA	NA	0	NA	NA	NA	0
rs187846474	C	NA	0	0	0	0	0	0	NA	0	NA	0	0	NA	NA	NA	0	NA	NA	NA	0
rs183880501	C	NA	0	0	0	0	0	0	NA	0	NA	0	0	NA	NA	NA	0	NA	NA	NA	0
rs191961425	T	NA	0.006944	0	0	0	0	0	NA	0	NA	0	0	NA	NA	NA	0.01961	NA	NA	NA	0
rs143459173	C	NA	0	0	0	0	0	0	NA	0	NA	0	0	NA	NA	NA	0.006536	NA	NA	NA	0
rs187467963	T	NA	0	0	0	0	0	0	NA	0	NA	0	0	NA	NA	NA	0.006536	NA	NA	NA	0

SNP	Allele	Cusco	ESN	FIN	GBR	GIH	GWD	IBS	Iquitos	ITU	Jacarus	JPT	KHV	Lamas	Lambayeque	Lima	LWK	Matses	Moche	Moquegua	MSL
rs184459616	A	NA	0	0	0	0	0	0	NA	0	NA	0	0	NA	NA	NA	0	NA	NA	NA	0
rs148805807	T	NA	0	0	0	0	0.1053	0	NA	0	NA	0	0	NA	NA	NA	0.02614	NA	NA	NA	0.03906
rs199804629	T	NA	0	0	0	0	0	0	NA	0	NA	0	0	NA	NA	NA	0	NA	NA	NA	0
rs138390800	C	NA	0.01389	0	0	0	0	0	NA	0	NA	0	0	NA	NA	NA	0	NA	NA	NA	0
rs1514281	C	NA	0.3056	0	0.007353	0.09333	0.2573	0	NA	0.1103	NA	0.02632	0.02632	NA	NA	NA	0.3268	NA	NA	NA	0.2656
rs148408803	T	NA	0	0	0.02941	0	0	0.00625	NA	0	NA	0	0	NA	NA	NA	0	NA	NA	NA	0.007812
rs181799530	T	NA	0.006944	0	0	0	0	0	NA	0	NA	0	0	NA	NA	NA	0.01961	NA	NA	NA	0
rs192479337	C	NA	0	0	0	0	0	0	NA	0	NA	0	0	NA	NA	NA	0	NA	NA	NA	0
rs181964653	A	NA	0	0	0	0	0	0	NA	0	NA	0.006579	0	NA	NA	NA	0	NA	NA	NA	0
rs113691336	C	NA	0.09722	0.3312	0.2868	0.2333	0.07602	0.3375	NA	0.1724	NA	0	0.006579	NA	NA	NA	0.07843	NA	NA	NA	0.07031
rs184756568	T	NA	0	0	0	0	0	0	NA	0	NA	0	0	NA	NA	NA	0.006536	NA	NA	NA	0
rs184453641	A	NA	0	0	0	0	0	0	NA	0	NA	0	0	NA	NA	NA	0	NA	NA	NA	0
rs188558468	A	NA	0	0	0	0	0	0	NA	0	NA	0.006579	0	NA	NA	NA	0	NA	NA	NA	0
rs2301692	C	NA	0.1806	0	0	0.09333	0.1579	0	NA	0.1103	NA	0.02632	0.02632	NA	NA	NA	0.09804	NA	NA	NA	0.1172
rs189821013	A	NA	0	0	0	0	0	0	NA	0	NA	0	0	NA	NA	NA	0	NA	NA	NA	0
rs189699255	T	NA	0	0	0	0	0	0	NA	0	NA	0	0	NA	NA	NA	0.006536	NA	NA	NA	0
rs4646153	C	NA	0.1042	0.3312	0.2868	0.2333	0.08772	0.3375	NA	0.1586	NA	0	0.006579	NA	NA	NA	0.07843	NA	NA	NA	0.08594
rs182802402	C	NA	0	0	0	0	0	0	NA	0	NA	0	0.006579	NA	NA	NA	0	NA	NA	NA	0
rs186905240	T	NA	0.01389	0	0	0	0	0	NA	0	NA	0	0	NA	NA	NA	0.006536	NA	NA	NA	0.007812
rs181989524	C	NA	0	0	0.007353	0	0	0	NA	0	NA	0	0	NA	NA	NA	0	NA	NA	NA	0
rs142060377	A	NA	0	0	0.02206	0	0	0.00625	NA	0	NA	0	0	NA	NA	NA	0	NA	NA	NA	0
rs79473368	T	NA	0	0	0	0	0	0	NA	0	NA	0	0.006579	NA	NA	NA	0	NA	NA	NA	0
rs73635825	G	NA	0.006944	0	0	0	0	0	NA	0	NA	0	0	NA	NA	NA	0	NA	NA	NA	0
rs141605946	T	NA	0	0	0	0	0	0	NA	0	NA	0	0	NA	NA	NA	0	NA	NA	NA	0
rs185220883	A	NA	0	0	0.007353	0	0	0	NA	0	NA	0	0	NA	NA	NA	0	NA	NA	NA	0
rs191099160	A	NA	0.006944	0	0	0	0.0117	0	NA	0	NA	0	0	NA	NA	NA	0	NA	NA	NA	0.01562
rs4646169	A	NA	0.01389	0	0	0	0	0	NA	0	NA	0	0	NA	NA	NA	0.01307	NA	NA	NA	0.03125
rs187738069	A	NA	0	0	0	0	0	0	NA	0	NA	0	0	NA	NA	NA	0	NA	NA	NA	0
rs184589032	T	NA	0	0	0	0	0	0	NA	0	NA	0	0	NA	NA	NA	0	NA	NA	NA	0
rs34481900	C	NA	0.1667	0.3312	0.2868	0.2333	0.1637	0.3375	NA	0.1586	NA	0	0.006579	NA	NA	NA	0.2288	NA	NA	NA	0.125
rs188991352	C	NA	0	0.0125	0	0	0	0	NA	0	NA	0	0	NA	NA	NA	0	NA	NA	NA	0
rs201835187	AC	NA	0	0	0.007353	0	0	0	NA	0	NA	0	0	NA	NA	NA	0	NA	NA	NA	0
rs187161283	G	NA	0	0	0	0	0	0	NA	0	NA	0	0	NA	NA	NA	0	NA	NA	NA	0
rs35803318	T	0.08475	0	0.025	0.04412	0	0	0.1	0.08475	0	0.1842	0	0	0.02632	0.05556	0.1915	0	0.1538	0.2	0.1852	0
rs143079256	T	NA	0.03472	0	0	0	0.04094	0	NA	0	NA	0	0	NA	NA	NA	0.0915	NA	NA	NA	0.03125
rs189944937	G	NA	0	0	0	0	0	0	NA	0	NA	0	0	NA	NA	NA	0	NA	NA	NA	0
rs4646147	T	NA	0.1042	0.3375	0.2794	0.2333	0.117	0.3375	NA	0.1586	NA	0	0.006579	NA	NA	NA	0.08497	NA	NA	NA	0.1328
rs190133379	T	NA	0	0	0	0	0	0	NA	0	NA	0	0	NA	NA	NA	0	NA	NA	NA	0
rs144523173	A	NA	0	0	0	0	0	0	NA	0	NA	0.01316	0.006579	NA	NA	NA	0	NA	NA	NA	0

SNP	Allele	Cusco	ESN	FIN	GBR	GIH	GWD	IBS	Iquitos	ITU	Jacarus	JPT	KHV	Lamas	Lambayeque	Lima	LWK	Matses	Moche	Moquegua	MSL
rs145502291	T	NA	0.006944	0	0	0	0	0	NA	0	NA	0	0	NA	NA	NA	0.006536	NA	NA	NA	0
rs149958080	A	NA	0	0	0	0	0	0	NA	0	NA	0.006579	0	NA	NA	NA	0	NA	NA	NA	0
rs4646186	A	NA	0.09722	0	0	0	0.07018	0	NA	0	NA	0	0	NA	NA	NA	0.05882	NA	NA	NA	0.07031
rs189142411	A	NA	0	0	0	0	0	0	NA	0	NA	0	0	NA	NA	NA	0	NA	NA	NA	0
rs4646127	A	NA	0.1667	0.3312	0.2868	0.2333	0.1579	0.3375	NA	0.1586	NA	0	0.006579	NA	NA	NA	0.2288	NA	NA	NA	0.125
rs189140113	A	NA	0	0	0	0	0	0	NA	0	NA	0	0	NA	NA	NA	0	NA	NA	NA	0
rs4646143	T	NA	0.1042	0.3375	0.2794	0.2333	0.117	0.3375	NA	0.1586	NA	0	0.006579	NA	NA	NA	0.08497	NA	NA	NA	0.1328
rs41303171	C	NA	0	0.00625	0.02941	0	0	0.025	NA	0	NA	0	0	NA	NA	NA	0	NA	NA	NA	0
rs182808331	G	NA	0	0	0.007353	0	0	0	NA	0	NA	0	0	NA	NA	NA	0	NA	NA	NA	0
rs971249	T	NA	0.1667	0.3375	0.2868	0.2333	0.1871	0.3375	NA	0.1586	NA	0	0.006579	NA	NA	NA	0.2353	NA	NA	NA	0.1719
rs186029035	A	NA	0	0	0	0	0	0	NA	0	NA	0	0	NA	NA	NA	0.006536	NA	NA	NA	0
rs184978092	G	NA	0	0	0	0	0	0	NA	0	NA	0	0	NA	NA	NA	0	NA	NA	NA	0
rs192180091	C	NA	0	0	0	0	0	0	NA	0	NA	0	0	NA	NA	NA	0.006536	NA	NA	NA	0
rs34161673	C	NA	0	0	0	0	0	0.00625	NA	0	NA	0	0	NA	NA	NA	0	NA	NA	NA	0
rs188496534	C	NA	0	0	0	0	0	0	NA	0	NA	0	0	NA	NA	NA	0.006536	NA	NA	NA	0
rs193005774	T	NA	0	0	0	0	0	0	NA	0	NA	0	0	NA	NA	NA	0	NA	NA	NA	0
rs182372519	G	NA	0	0	0	0	0	0	NA	0	NA	0	0	NA	NA	NA	0.006536	NA	NA	NA	0
rs151205966	G	NA	0	0	0	0	0	0	NA	0	NA	0	0	NA	NA	NA	0	NA	NA	NA	0
rs182550682	A	NA	0	0	0	0	0	0	NA	0	NA	0	0	NA	NA	NA	0.006536	NA	NA	NA	0
rs191332175	G	NA	0.006944	0	0	0	0.01754	0	NA	0	NA	0	0	NA	NA	NA	0	NA	NA	NA	0
rs144061872	T	NA	0	0.0125	0	0	0	0	NA	0	NA	0	0	NA	NA	NA	0	NA	NA	NA	0
rs189571339	C	NA	0	0	0	0	0	0	NA	0	NA	0.006579	0	NA	NA	NA	0	NA	NA	NA	0
rs138940089	T	NA	0.006944	0	0	0	0	0	NA	0	NA	0	0	NA	NA	NA	0.01961	NA	NA	NA	0
rs190870013	C	NA	0	0	0	0	0	0	NA	0	NA	0	0	NA	NA	NA	0	NA	NA	NA	0
rs188290800	C	NA	0	0	0	0	0	0	NA	0	NA	0	0	NA	NA	NA	0	NA	NA	NA	0
rs185622388	A	NA	0	0	0	0	0	0	NA	0	NA	0	0	NA	NA	NA	0	NA	NA	NA	0
rs190055656	T	NA	0	0	0	0	0	0	NA	0	NA	0	0	NA	NA	NA	0.006536	NA	NA	NA	0
rs138854040	A	NA	0	0	0	0	0	0	NA	0	NA	0.09868	0.02632	NA	NA	NA	0	NA	NA	NA	0
rs4646182	C	NA	0.06944	0	0	0	0.2105	0	NA	0	NA	0	0	NA	NA	NA	0.06536	NA	NA	NA	0.1328
rs181796402	T	NA	0.006944	0	0	0	0.0117	0	NA	0	NA	0	0	NA	NA	NA	0	NA	NA	NA	0.01562
rs182366225	T	NA	0	0	0	0	0	0	NA	0	NA	0	0.04605	NA	NA	NA	0	NA	NA	NA	0
rs4646151	A	NA	0.09722	0	0	0	0.07018	0	NA	0	NA	0	0	NA	NA	NA	0.05882	NA	NA	NA	0.07031
rs145890723	A	NA	0.02083	0	0.007353	0	0.005848	0	NA	0	NA	0	0	NA	NA	NA	0.01307	NA	NA	NA	0.007812
rs191536933	T	NA	0	0	0	0	0	0	NA	0	NA	0	0	NA	NA	NA	0	NA	NA	NA	0
rs151005246	A	NA	0	0	0	0	0	0	NA	0	NA	0.03947	0.01316	NA	NA	NA	0	NA	NA	NA	0
rs182219706	T	NA	0	0	0	0	0	0	NA	0	NA	0	0	NA	NA	NA	0	NA	NA	NA	0
rs200217737	CA	NA	0.04167	0.00625	0	0	0.07018	0	NA	0	NA	0.006579	0	NA	NA	NA	0.03922	NA	NA	NA	0.09375
rs375635972	T	NA	0	0	0	0	0	0	NA	0	NA	0	0	NA	NA	NA	0	NA	NA	NA	0
rs4646172	GA	NA	0.03472	0	0	0	0.08772	0	NA	0	NA	0	0	NA	NA	NA	0.1373	NA	NA	NA	0.05469

SNP	Allele	Cusco	ESN	FIN	GBR	GIH	GWD	IBS	Iquitos	ITU	Jacarus	JPT	KHV	Lamas	Lambayeque	Lima	LWK	Matses	Moche	Moquegua	MSL
rs183661335	G	NA	0.006944	0	0	0	0.0117	0	NA	0	NA	0	0	NA	NA	NA	0	NA	NA	NA	0.01562
rs181695790	G	NA	0	0	0	0	0	0	NA	0	NA	0	0	NA	NA	NA	0	NA	NA	NA	0
rs188283144	C	NA	0	0	0	0	0	0	NA	0	NA	0	0	NA	NA	NA	0	NA	NA	NA	0
rs199951323	C	NA	0	0	0	0	0	0	NA	0	NA	0.006579	0	NA	NA	NA	0	NA	NA	NA	0
rs181788868	C	NA	0.006944	0	0	0	0.0117	0	NA	0	NA	0	0	NA	NA	NA	0	NA	NA	NA	0.01562
rs72614595	T	NA	0	0	0	0	0	0	NA	0	NA	0	0.006579	NA	NA	NA	0	NA	NA	NA	0
rs182287132	A	NA	0	0	0	0	0	0	NA	0	NA	0	0	NA	NA	NA	0.01307	NA	NA	NA	0
rs181522566	C	NA	0	0	0	0	0	0	NA	0	NA	0	0	NA	NA	NA	0	NA	NA	NA	0
rs4646120	G	NA	0.2569	0.475	0.4191	0.2733	0.3743	0.425	NA	0.1655	NA	0	0.006579	NA	NA	NA	0.3464	NA	NA	NA	0.2969
rs4646177	T	NA	0.04861	0	0	0	0.02924	0	NA	0	NA	0	0	NA	NA	NA	0.05229	NA	NA	NA	0.08594
rs371025504	G	NA	0	0	0.007353	0	0	0	NA	0	NA	0	0	NA	NA	NA	0	NA	NA	NA	0
rs185525294	A	NA	0	0	0	0	0	0	NA	0	NA	0	0	NA	NA	NA	0	NA	NA	NA	0
rs189278377	A	NA	0	0	0.007353	0	0	0	NA	0	NA	0	0	NA	NA	NA	0	NA	NA	NA	0
rs4646183	C	NA	0	0	0	0	0	0	NA	0	NA	0	0	NA	NA	NA	0.006536	NA	NA	NA	0
rs188292876	C	NA	0	0	0	0	0	0	NA	0	NA	0	0	NA	NA	NA	0.01307	NA	NA	NA	0
rs186957036	C	NA	0	0	0	0	0	0	NA	0	NA	0	0	NA	NA	NA	0	NA	NA	NA	0
rs4646121	T	NA	0.07639	0	0	0	0.1813	0	NA	0	NA	0	0	NA	NA	NA	0.07843	NA	NA	NA	0.125
rs143102000	A	NA	0	0	0	0	0.1053	0	NA	0	NA	0	0	NA	NA	NA	0.02614	NA	NA	NA	0.03906
rs185005111	T	NA	0	0	0	0	0	0	NA	0	NA	0	0	NA	NA	NA	0.006536	NA	NA	NA	0
rs113208650	T	0	0.05556	0	0.007353	0	0.09357	0	0	0	0	0	0	0	0	0.06383	0.1634	0	0	0	0.08594
rs186914723	A	NA	0	0.0125	0	0	0	0	NA	0	NA	0	0	NA	NA	NA	0	NA	NA	NA	0
rs4646150	T	NA	0	0	0	0	0	0	NA	0	NA	0	0	NA	NA	NA	0	NA	NA	NA	0
rs4646135	C	NA	0.0625	0	0.007353	0	0.09942	0	NA	0	NA	0	0	NA	NA	NA	0.1569	NA	NA	NA	0.08594
rs184064003	A	NA	0	0	0.007353	0	0	0	NA	0	NA	0	0	NA	NA	NA	0	NA	NA	NA	0
rs4646187	A	NA	0	0.00625	0	0.006667	0	0	NA	0	NA	0	0	NA	NA	NA	0	NA	NA	NA	0
rs185723767	T	NA	0	0	0	0	0	0	NA	0	NA	0	0	NA	NA	NA	0	NA	NA	NA	0
rs376237761	G	NA	0	0	0	0	0	0	NA	0	NA	0	0	NA	NA	NA	0	NA	NA	NA	0
rs373723722	T	NA	0	0	0	0	0	0	NA	0	NA	0	0.01316	NA	NA	NA	0	NA	NA	NA	0
rs61433707	T	NA	0.02083	0	0	0	0	0	NA	0	NA	0	0.01316	NA	NA	NA	0.006536	NA	NA	NA	0.01562
rs201424204	C	NA	0	0.0125	0	0	0	0	NA	0	NA	0.125	0.09868	NA	NA	NA	0	NA	NA	NA	0
rs146087218	T	NA	0	0	0	0	0	0	NA	0	NA	0	0	NA	NA	NA	0.006536	NA	NA	NA	0
rs2301693	A	NA	0.1806	0	0	0.09333	0.1579	0	NA	0.1103	NA	0.02632	0.02632	NA	NA	NA	0.09804	NA	NA	NA	0.1172
rs186036909	T	NA	0	0	0	0	0	0	NA	0	NA	0.006579	0	NA	NA	NA	0	NA	NA	NA	0
rs191884180	G	NA	0	0	0	0	0	0	NA	0	NA	0	0	NA	NA	NA	0	NA	NA	NA	0
rs148977224	C	NA	0	0	0	0	0	0	NA	0	NA	0	0	NA	NA	NA	0	NA	NA	NA	0
rs190850356	A	NA	0	0	0	0	0	0	NA	0	NA	0	0	NA	NA	NA	0	NA	NA	NA	0
rs192474956	G	NA	0	0	0	0	0	0	NA	0	NA	0	0	NA	NA	NA	0	NA	NA	NA	0
rs56700224	T	NA	0.0625	0	0.007353	0	0.07018	0	NA	0	NA	0	0	NA	NA	NA	0.1503	NA	NA	NA	0.03906
rs142017934	C	NA	0.006944	0	0	0	0.01754	0	NA	0	NA	0	0	NA	NA	NA	0	NA	NA	NA	0

SNP	Allele	Cusco	ESN	FIN	GBR	GIH	GWD	IBS	Iquitos	ITU	Jacarus	JPT	KHV	Lamas	Lambayeque	Lima	LWK	Matses	Moche	Moquegua	MSL
rs41297301	T	0	0	0	0.02941	0	0	0.00625	0	0	0	0	0	0	0	0	0	0	0	0	0
rs4646168	G	NA	0.07639	0	0.007353	0	0.09942	0	NA	0	NA	0	0	NA	NA	NA	0.183	NA	NA	NA	0.1094
rs2048683	T	0.1525	0.1667	0.3375	0.2868	0.2333	0.1871	0.3375	0.05085	0.1586	0.1579	0	0.006579	0.05263	0.05556	0.234	0.2353	0.1538	0.2615	0.1667	0.1719
rs181786494	A	NA	0	0	0	0	0.005848	0	NA	0	NA	0	0	NA	NA	NA	0.006536	NA	NA	NA	0
rs4646124	T	NA	0.1667	0.3312	0.2868	0.2333	0.1871	0.3375	NA	0.1586	NA	0	0.006579	NA	NA	NA	0.2418	NA	NA	NA	0.1641
rs138529167	A	NA	0	0	0	0	0	0	NA	0	NA	0	0	NA	NA	NA	0.01961	NA	NA	NA	0
rs4646125	T	NA	0	0	0	0	0.02924	0	NA	0	NA	0	0	NA	NA	NA	0.006536	NA	NA	NA	0.04688
rs4646162	G	NA	0.01389	0	0	0	0.02924	0	NA	0	NA	0	0	NA	NA	NA	0.02614	NA	NA	NA	0.07031
rs180864908	G	NA	0	0	0	0	0	0	NA	0	NA	0	0	NA	NA	NA	0	NA	NA	NA	0
rs184178697	T	NA	0	0	0	0	0	0	NA	0	NA	0	0	NA	NA	NA	0	NA	NA	NA	0
rs145954042	C	NA	0	0	0	0	0	0	NA	0	NA	0	0	NA	NA	NA	0	NA	NA	NA	0
rs4646149	T	NA	0	0	0	0	0.02339	0	NA	0	NA	0	0	NA	NA	NA	0.006536	NA	NA	NA	0.04688
rs147312210	C	NA	0.006944	0	0	0	0	0	NA	0	NA	0	0	NA	NA	NA	0	NA	NA	NA	0
rs149039346	G	NA	0.006944	0	0	0	0	0	NA	0	NA	0	0	NA	NA	NA	0	NA	NA	NA	0
rs192674337	G	NA	0	0.01875	0.007353	0	0	0	NA	0	NA	0	0	NA	NA	NA	0	NA	NA	NA	0
rs193290621	A	NA	0	0	0	0	0	0	NA	0	NA	0	0	NA	NA	NA	0	NA	NA	NA	0
rs187390194	G	NA	0	0	0	0	0	0	NA	0	NA	0	0	NA	NA	NA	0	NA	NA	NA	0
rs4646128	T	NA	0.04861	0	0	0	0.06433	0	NA	0	NA	0	0	NA	NA	NA	0.05882	NA	NA	NA	0.1016
rs4646133	G	NA	0	0	0	0	0.02924	0	NA	0	NA	0	0	NA	NA	NA	0.006536	NA	NA	NA	0.04688
rs181620291	C	NA	0	0	0	0	0	0	NA	0	NA	0	0	NA	NA	NA	0	NA	NA	NA	0.007812
rs182501431	G	NA	0	0	0	0	0	0	NA	0	NA	0.01316	0	NA	NA	NA	0	NA	NA	NA	0
rs184503057	T	NA	0	0	0	0	0	0	NA	0	NA	0	0	NA	NA	NA	0.006536	NA	NA	NA	0
rs181169181	C	NA	0	0	0	0	0	0	NA	0	NA	0	0	NA	NA	NA	0	NA	NA	NA	0
rs4646129	T	NA	0.0625	0	0.007353	0	0.07018	0	NA	0	NA	0	0	NA	NA	NA	0.1503	NA	NA	NA	0.03906
rs185182583	A	NA	0.006944	0	0	0	0.0117	0	NA	0	NA	0	0	NA	NA	NA	0	NA	NA	NA	0.01562
rs140083333	A	NA	0	0	0	0	0	0	NA	0	NA	0	0	NA	NA	NA	0.006536	NA	NA	NA	0
rs4646146	C	NA	0.04861	0	0	0	0.05848	0	NA	0	NA	0	0	NA	NA	NA	0.05882	NA	NA	NA	0.09375
rs186271730	T	NA	0	0	0	0	0	0	NA	0	NA	0	0	NA	NA	NA	0.006536	NA	NA	NA	0
rs189283596	C	NA	0	0	0	0	0	0	NA	0	NA	0	0	NA	NA	NA	0	NA	NA	NA	0
rs189944736	C	NA	0	0	0	0	0	0	NA	0	NA	0	0	NA	NA	NA	0.006536	NA	NA	NA	0
rs148006212	A	NA	0	0	0	0	0.02924	0	NA	0	NA	0	0	NA	NA	NA	0.01307	NA	NA	NA	0.04688
rs372812138	T	NA	0	0	0	0	0	0	NA	0	NA	0	0.006579	NA	NA	NA	0	NA	NA	NA	0
rs4646173	A	NA	0	0	0	0	0.02924	0	NA	0	NA	0	0	NA	NA	NA	0.006536	NA	NA	NA	0.04688
rs4646117	C	NA	0.006944	0	0	0	0	0	NA	0	NA	0	0	NA	NA	NA	0	NA	NA	NA	0
rs187959864	T	NA	0	0	0	0	0	0	NA	0	NA	0	0	NA	NA	NA	0	NA	NA	NA	0
rs191869625	A	NA	0	0	0.007353	0	0	0.01875	NA	0	NA	0	0	NA	NA	NA	0	NA	NA	NA	0
rs200885467	TA	NA	0	0.03125	0.02206	0.01333	0	0.0125	NA	0	NA	0	0	NA	NA	NA	0.006536	NA	NA	NA	0
rs72614596	A	NA	0	0.0125	0	0	0	0	NA	0	NA	0.08553	0.08553	NA	NA	NA	0	NA	NA	NA	0
rs185721534	G	NA	0.06944	0	0	0	0.1871	0	NA	0	NA	0	0	NA	NA	NA	0.06536	NA	NA	NA	0.125

SNP	Allele	Cusco	ESN	FIN	GBR	GIH	GWD	IBS	Iquitos	ITU	Jacarus	JPT	KHV	Lamas	Lambayeque	Lima	LWK	Matses	Moche	Moquegua	MSL
rs183546232	G	NA	0	0.03125	0.02206	0.01333	0	0.0125	NA	0	NA	0	0	NA	NA	NA	0.006536	NA	NA	NA	0
rs757066	C	NA	0	0.3375	0.2794	0.2333	0.005848	0.3375	NA	0.1586	NA	0	0.006579	NA	NA	NA	0.01961	NA	NA	NA	0
rs186277162	C	NA	0	0	0	0	0	0	NA	0	NA	0	0	NA	NA	NA	0	NA	NA	NA	0
rs4646157	C	NA	0.09722	0	0	0	0.04678	0.00625	NA	0	NA	0	0	NA	NA	NA	0.04575	NA	NA	NA	0.03125
rs2023802	G	NA	0.1667	0.3312	0.2868	0.2333	0.1871	0.3375	NA	0.1586	NA	0	0.006579	NA	NA	NA	0.2353	NA	NA	NA	0.1719
rs200260858	C	NA	0.02083	0.00625	0.007353	0.006667	0.04094	0	NA	0.006897	NA	0	0	NA	NA	NA	0	NA	NA	NA	0.02344
rs191336106	G	NA	0	0	0	0	0.005848	0	NA	0	NA	0	0	NA	NA	NA	0	NA	NA	NA	0
rs150200247	A	NA	0	0	0	0	0	0	NA	0	NA	0.006579	0	NA	NA	NA	0	NA	NA	NA	0
rs187474513	T	NA	0.08333	0	0	0	0	0	NA	0	NA	0	0	NA	NA	NA	0.006536	NA	NA	NA	0.01562
rs184874220	A	NA	0	0	0	0	0	0	NA	0	NA	0	0	NA	NA	NA	0	NA	NA	NA	0
rs189810201	C	NA	0	0	0	0	0	0	NA	0	NA	0	0	NA	NA	NA	0.006536	NA	NA	NA	0
rs184746393	C	NA	0	0.00625	0	0	0	0	NA	0	NA	0	0	NA	NA	NA	0	NA	NA	NA	0
rs72614598	C	NA	0	0.0125	0	0	0	0	NA	0	NA	0.08553	0.08553	NA	NA	NA	0	NA	NA	NA	0
rs180878567	C	NA	0.01389	0	0	0	0	0	NA	0	NA	0	0	NA	NA	NA	0.01961	NA	NA	NA	0
rs200477770	G	NA	0	0	0	0	0	0	NA	0	NA	0.006579	0	NA	NA	NA	0	NA	NA	NA	0
rs199612962	G	NA	0	0	0	0	0	0	NA	0	NA	0.006579	0	NA	NA	NA	0	NA	NA	NA	0
rs200180615	T	NA	0	0	0	0.006667	0	0	NA	0	NA	0	0	NA	NA	NA	0	NA	NA	NA	0
rs192914427	T	NA	0	0	0	0	0	0	NA	0	NA	0	0	NA	NA	NA	0.006536	NA	NA	NA	0
rs181390351	T	NA	0	0	0	0	0	0	NA	0	NA	0	0	NA	NA	NA	0	NA	NA	NA	0
rs4646178	G	NA	0.04861	0	0	0	0.02924	0	NA	0	NA	0	0	NA	NA	NA	0.05882	NA	NA	NA	0.08594
rs187251131	C	NA	0	0	0	0	0	0	NA	0	NA	0	0	NA	NA	NA	0	NA	NA	NA	0
rs192692847	C	NA	0	0	0	0	0	0	NA	0	NA	0	0.006579	NA	NA	NA	0	NA	NA	NA	0
rs4646167	T	NA	0.09722	0	0	0	0.07018	0	NA	0	NA	0	0	NA	NA	NA	0.05882	NA	NA	NA	0.07031
rs189691652	T	NA	0	0	0	0	0	0	NA	0	NA	0	0	NA	NA	NA	0	NA	NA	NA	0
rs138763015	G	NA	0	0	0	0	0	0	NA	0	NA	0.01974	0.03947	NA	NA	NA	0	NA	NA	NA	0
rs372272603	A	NA	0	0	0	0.006667	0	0	NA	0	NA	0	0	NA	NA	NA	0	NA	NA	NA	0
rs184730109	A	NA	0	0	0	0	0	0	NA	0	NA	0	0.006579	NA	NA	NA	0.006536	NA	NA	NA	0
rs186261546	C	NA	0	0	0	0	0	0	NA	0	NA	0	0	NA	NA	NA	0	NA	NA	NA	0

Table S2-A – *ACE2* allele frequencies continuation. Bold = functionally relevant SNPs found in our databases; NA = missing data.

SNP	Allele	MXL	PEL	Pelotas	PJL	Puno	PUR	Qeros	Quechua	Salvador	Shimaa	Shipibo	STU	Tacna	Tallanes	Trujillo	TSI	Tumbes	Uros	YRI
**rs4646123**	**T**	**0.02083**	**0**	**NA**	**0**	**NA**	**0.006494**	**NA**	**NA**	**NA**	**NA**	**NA**	**0**	**NA**	**NA**	**NA**	**0**	**NA**	**NA**	**0.08537**
**rs147311723**	**A**	**0**	**0**	**NA**	**0**	**NA**	**0.006494**	**NA**	**NA**	**NA**	**NA**	**NA**	**0**	**NA**	**NA**	**NA**	**0**	**NA**	**NA**	**0.04268**
**rs4646188**	**G**	**0.03125**	**0.0155**	**0.08148**	**0.09028**	**NA**	**0.05195**	**NA**	**NA**	**0.0219**	**NA**	**NA**	**0.04027**	**NA**	**NA**	**NA**	**0.1006**	**NA**	**NA**	**0**
**rs1514283**	**C**	**0.01042**	**0.0155**	**0.03704**	**0.0625**	**0**	**0.05195**	**0**	**NA**	**0.2409**	**0**	**0**	**0.1074**	**0**	**0**	**0.08**	**0.006289**	**0.08929**	**0**	**0.4146**
**rs4646156**	**A**	**0.1667**	**0.124**	**0.7481**	**0.2778**	**NA**	**0.3571**	**NA**	**NA**	**0.7226**	**NA**	**NA**	**0.1678**	**NA**	**NA**	**NA**	**0.3774**	**NA**	**NA**	**0.2195**
**rs714205**	**G**	**0.3438**	**0.3256**	**0.2296**	**0.3819**	**NA**	**0.2727**	**NA**	**NA**	**0.1971**	**NA**	**NA**	**0.5302**	**NA**	**NA**	**NA**	**0.1572**	**NA**	**NA**	**0.06098**

SNP	Allele	MXL	PEL	Pelotas	PJL	Puno	PUR	Qeros	Quechua	Salvador	Shimaa	Shipibo	STU	Tacna	Tallanes	Trujillo	TSI	Tumbes	Uros	YRI
**rs233575**	**G**	**0.1771**	**0.1163**	**0.237**	**0.2569**	**NA**	**0.2143**	**NA**	**NA**	**0.1241**	**NA**	**NA**	**0.1409**	**NA**	**NA**	**NA**	**0.3962**	**NA**	**NA**	**0**
**rs2074192**	**T**	**0.4167**	**0.5271**	**0.4222**	**0.2847**	**0.6164**	**0.3377**	**0.375**	**NA**	**0.2993**	**0.5362**	**0.5**	**0.1812**	**0.4565**	**0.5873**	**0.4583**	**0.4214**	**0.3393**	**0.5**	**0.3049**
**rs4646174**	**G**	**0.7917**	**0.8605**	**0.3333**	**0.6736**	**NA**	**0.6364**	**NA**	**NA**	**0.4964**	**NA**	**NA**	**0.7315**	**NA**	**NA**	**NA**	**0.6038**	**NA**	**NA**	**0.3659**
**rs1514282**	**C**	**0.01042**	**0.02326**	**NA**	**0.0625**	**NA**	**0.06494**	**NA**	**NA**	**NA**	**NA**	**NA**	**0.1074**	**NA**	**NA**	**NA**	**0.006289**	**NA**	**NA**	**0.4268**
**rs4646181**	**T**	**0**	**0**	**NA**	**0.006944**	**NA**	**0.01299**	**NA**	**NA**	**NA**	**NA**	**NA**	**0**	**NA**	**NA**	**NA**	**0**	**NA**	**NA**	**0.04268**
**rs4646176**	**G**	**0.01042**	**0.007752**	**NA**	**0.0625**	**NA**	**0.03896**	**NA**	**NA**	**NA**	**NA**	**NA**	**0.1074**	**NA**	**NA**	**NA**	**0.006289**	**NA**	**NA**	**0.2073**
**rs1978124**	**T**	**0.2083**	**0.124**	**0.3259**	**0.2778**	**NA**	**0.3961**	**NA**	**NA**	**0.1752**	**NA**	**NA**	**0.1879**	**NA**	**NA**	**NA**	**0.5157**	**NA**	**NA**	**0.128**
**rs6632677**	**C**	**0.04167**	**0.06202**	**NA**	**0.006944**	**NA**	**0.01299**	**NA**	**NA**	**NA**	**NA**	**NA**	**0.006711**	**NA**	**NA**	**NA**	**0**	**NA**	**NA**	**0**
**rs4646171**	**C**	**0.01042**	**0.007752**	**0.007407**	**0.0625**	**NA**	**0.03896**	**NA**	**NA**	**0.1119**	**NA**	**NA**	**0.1074**	**NA**	**NA**	**NA**	**0.006289**	**NA**	**NA**	**0.2073**
**rs879922**	**G**	**0.7917**	**0.8605**	**0.3358**	**0.6736**	**NA**	**0.6364**	**NA**	**NA**	**0.5**	**NA**	**NA**	**0.7315**	**NA**	**NA**	**NA**	**0.6038**	**NA**	**NA**	**0.3659**
**rs2106809**	**G**	**0.3646**	**0.3566**	**0.2741**	**0.4028**	**NA**	**0.2597**	**NA**	**NA**	**0.2117**	**NA**	**NA**	**0.5638**	**NA**	**NA**	**NA**	**0.2013**	**NA**	**NA**	**0.07317**
**rs4646142**	**C**	**0.3646**	**0.3566**	**NA**	**0.4028**	**NA**	**0.2727**	**NA**	**NA**	**NA**	**NA**	**NA**	**0.5302**	**NA**	**NA**	**NA**	**0.1824**	**NA**	**NA**	**0.189**
**rs4646155**	**T**	**0.01042**	**0.007752**	**NA**	**0.0625**	**NA**	**0.03896**	**NA**	**NA**	**NA**	**NA**	**NA**	**0.1074**	**NA**	**NA**	**NA**	**0.006289**	**NA**	**NA**	**0.189**
**rs4240157**	**T**	**0.7917**	**0.8527**	**0.6667**	**0.6667**	**0.93151**	**0.6429**	**0.875**	**0.8293**	**0.5036**	**1**	**0.96667**	**0.7383**	**0.91304**	**0.8889**	**0.8**	**0.5975**	**0.6727**	**0.95833**	**0.372**
**rs2285666**	**T**	**0.3646**	**0.3566**	**0.2963**	**0.4028**	**0.3014**	**0.2727**	**0.5**	**0.3415**	**0.2628**	**NA**	**0.4667**	**0.5302**	**0.4783**	**0.3016**	**0.4167**	**0.1824**	**0.2857**	**0.4583**	**0.1707**
**rs2158083**	**C**	**0.1667**	**0.124**	**0.2444**	**0.2639**	**NA**	**0.3571**	**NA**	**NA**	**0.2628**	**NA**	**NA**	**0.1678**	**NA**	**NA**	**NA**	**0.3836**	**NA**	**NA**	**0.2073**
rs147417432	A	0	0	NA	0	NA	0	NA	NA	NA	NA	NA	0	NA	NA	NA	0	NA	NA	0
rs60122685	T	0	0	NA	0	NA	0	NA	NA	NA	NA	NA	0	NA	NA	NA	0	NA	NA	0.006098
rs190342052	T	0	0	NA	0	NA	0	NA	NA	NA	NA	NA	0	NA	NA	NA	0	NA	NA	0.006098
rs188037336	T	0	0	NA	0	NA	0	NA	NA	NA	NA	NA	0	NA	NA	NA	0	NA	NA	0
rs4646163	T	0	0	NA	0	NA	0.006494	NA	NA	NA	NA	NA	0	NA	NA	NA	0	NA	NA	0.04878
rs191860450	C	0	0	NA	0	NA	0	NA	NA	NA	NA	NA	0	NA	NA	NA	0	NA	NA	0
rs4646114	T	0	0	NA	0	NA	0.06494	NA	NA	NA	NA	NA	0	NA	NA	NA	0	NA	NA	0.1159
rs4646145	G	0	0.007752	NA	0	NA	0.006494	NA	NA	NA	NA	NA	0	NA	NA	NA	0	NA	NA	0.07927
rs73195520	T	0	0	NA	0	NA	0	NA	NA	NA	NA	NA	0	NA	NA	NA	0	NA	NA	0
rs188007597	C	0	0	NA	0	NA	0	NA	NA	NA	NA	NA	0	NA	NA	NA	0	NA	NA	0
rs188895175	A	0	0.007752	NA	0	NA	0.01299	NA	NA	NA	NA	NA	0	NA	NA	NA	0	NA	NA	0.006098
rs149683072	C	0	0	NA	0	NA	0	NA	NA	NA	NA	NA	0	NA	NA	NA	0	NA	NA	0
rs201159862	C	0	0	NA	0	NA	0	NA	NA	NA	NA	NA	0	NA	NA	NA	0.006289	NA	NA	0
rs141562322	A	0	0	NA	0	NA	0	NA	NA	NA	NA	NA	0	NA	NA	NA	0	NA	NA	0.03049
rs182809041	C	0	0	NA	0	NA	0	NA	NA	NA	NA	NA	0	NA	NA	NA	0	NA	NA	0.006098
rs190327760	T	0	0	NA	0	NA	0	NA	NA	NA	NA	NA	0	NA	NA	NA	0	NA	NA	0
rs4646165	T	0	0	NA	0	NA	0.06494	NA	NA	NA	NA	NA	0	NA	NA	NA	0	NA	NA	0.122
rs187045475	C	0	0	NA	0	NA	0.006494	NA	NA	NA	NA	NA	0	NA	NA	NA	0	NA	NA	0
rs370596467	C	0	0	NA	0	NA	0	NA	NA	NA	NA	NA	0	NA	NA	NA	0	NA	NA	0
rs4646138	T	0	0.007752	NA	0	NA	0.006494	NA	NA	NA	NA	NA	0.006711	NA	NA	NA	0	NA	NA	0.03659
rs4646184	A	0.02083	0	0.02963	0	0	0.006494	0	NA	0.07299	0	0	0	0	0	0	0	0	0	0.05488
rs73195521	A	0	0	NA	0	NA	0	NA	NA	NA	NA	NA	0	NA	NA	NA	0	NA	NA	0
rs4646175	C	0.02083	0	NA	0	NA	0	NA	NA	NA	NA	NA	0	NA	NA	NA	0	NA	NA	0.04878

SNP	Allele	MXL	PEL	Pelotas	PJL	Puno	PUR	Qeros	Quechua	Salvador	Shimaa	Shipibo	STU	Tacna	Tallanes	Trujillo	TSI	Tumbes	Uros	YRI
rs4646119	G	0	0.007752	NA	0	NA	0.006494	NA	NA	NA	NA	NA	0	NA	NA	NA	0	NA	NA	0.09146
rs183452730	T	0	0	NA	0	NA	0	NA	NA	NA	NA	NA	0	NA	NA	NA	0	NA	NA	0
rs150550484	C	0	0	NA	0	NA	0	NA	NA	NA	NA	NA	0	NA	NA	NA	0	NA	NA	0
rs187860487	A	0	0	NA	0	NA	0	NA	NA	NA	NA	NA	0	NA	NA	NA	0	NA	NA	0
rs114165600	A	0	0	NA	0	NA	0	NA	NA	NA	NA	NA	0	NA	NA	NA	0	NA	NA	0.02439
rs137910448	C	0	0	NA	0	NA	0	NA	NA	NA	NA	NA	0	NA	NA	NA	0	NA	NA	0
rs187504060	C	0.01042	0	NA	0	NA	0	NA	NA	NA	NA	NA	0	NA	NA	NA	0	NA	NA	0
rs182246050	T	0	0	NA	0	NA	0	NA	NA	NA	NA	NA	0	NA	NA	NA	0	NA	NA	0
rs2316904	C	0.1667	0.1163	NA	0.2639	NA	0.3506	NA	NA	NA	NA	NA	0.1678	NA	NA	NA	0.3774	NA	NA	0.1341
rs4646141	A	0.02083	0	NA	0	NA	0	NA	NA	NA	NA	NA	0	NA	NA	NA	0	NA	NA	0.04878
rs4646154	T	0	0	NA	0	NA	0	NA	NA	NA	NA	NA	0	NA	NA	NA	0	NA	NA	0.01829
rs149434207	T	0	0.007752	0.007407	0	0	0.01948	0	NA	0.007299	0	0	0	0	0	0	0	0	0	0.0122
rs183135788	C	0	0	NA	0	NA	0	NA	NA	NA	NA	NA	0	NA	NA	NA	0	NA	NA	0
rs193237066	A	0	0	NA	0	NA	0	NA	NA	NA	NA	NA	0	NA	NA	NA	0	NA	NA	0
rs199569050	A	0.01042	0	NA	0	NA	0	NA	NA	NA	NA	NA	0	NA	NA	NA	0	NA	NA	0
rs4646159	T	0	0	NA	0	NA	0.006494	NA	NA	NA	NA	NA	0	NA	NA	NA	0	NA	NA	0.07927
rs4646112	T	0	0	NA	0	NA	0.06494	NA	NA	NA	NA	NA	0	NA	NA	NA	0	NA	NA	0.1098
rs190373279	T	0	0	NA	0	NA	0	NA	NA	NA	NA	NA	0	NA	NA	NA	0	NA	NA	0
rs2048684	A	0.1667	0.1163	0.2296	0.2639	NA	0.3506	NA	NA	0.1533	NA	NA	0.1678	NA	NA	NA	0.3711	NA	NA	0.128
rs190406475	A	0	0	NA	0	NA	0	NA	NA	NA	NA	NA	0	NA	NA	NA	0	NA	NA	0
rs183156483	A	0	0	NA	0	NA	0	NA	NA	NA	NA	NA	0	NA	NA	NA	0.006289	NA	NA	0
rs111572878	C	0.02083	0.0155	NA	0	NA	0.02597	NA	NA	NA	NA	NA	0	NA	NA	NA	0	NA	NA	0.1768
rs4646148	T	0.1667	0.1163	NA	0.2639	NA	0.3506	NA	NA	NA	NA	NA	0.1678	NA	NA	NA	0.3774	NA	NA	0.1341
rs190384874	A	0	0	NA	0	NA	0.006494	NA	NA	NA	NA	NA	0	NA	NA	NA	0	NA	NA	0
rs183576751	G	0	0	NA	0	NA	0	NA	NA	NA	NA	NA	0	NA	NA	NA	0	NA	NA	0
rs150909162	A	0	0	NA	0	NA	0	NA	NA	NA	NA	NA	0	NA	NA	NA	0	NA	NA	0
rs201489317	G	0	0	NA	0	NA	0	NA	NA	NA	NA	NA	0	NA	NA	NA	0	NA	NA	0
rs146750287	G	0	0	NA	0	NA	0	NA	NA	NA	NA	NA	0	NA	NA	NA	0	NA	NA	0
rs181161610	C	0	0	NA	0	NA	0	NA	NA	NA	NA	NA	0	NA	NA	NA	0	NA	NA	0
rs193159186	A	0	0	NA	0	NA	0	NA	NA	NA	NA	NA	0	NA	NA	NA	0	NA	NA	0
rs4646144	A	0.01042	0.007752	NA	0.0625	NA	0.03896	NA	NA	NA	NA	NA	0.1074	NA	NA	NA	0.006289	NA	NA	0.189
rs146122606	A	0.1667	0.124	NA	0.2639	NA	0.3571	NA	NA	NA	NA	NA	0.1678	NA	NA	NA	0.3774	NA	NA	0.1524
rs183655025	C	0	0	NA	0	NA	0.006494	NA	NA	NA	NA	NA	0	NA	NA	NA	0	NA	NA	0
rs200973492	G	0	0	NA	0	NA	0	NA	NA	NA	NA	NA	0	NA	NA	NA	0	NA	NA	0
rs182858184	A	0	0	NA	0	NA	0.006494	NA	NA	NA	NA	NA	0	NA	NA	NA	0	NA	NA	0
rs181159881	G	0	0	NA	0	NA	0.006494	NA	NA	NA	NA	NA	0	NA	NA	NA	0	NA	NA	0
rs233574	T	0.1771	0.1163	NA	0.2569	NA	0.2792	NA	NA	NA	NA	NA	0.1409	NA	NA	NA	0.3836	NA	NA	0.122
rs4646118	T	0.02083	0	NA	0	NA	0.006494	NA	NA	NA	NA	NA	0	NA	NA	NA	0	NA	NA	0.07317
rs2316903	G	0.1667	0.1163	0.2296	0.2639	NA	0.3506	NA	NA	0.1679	NA	NA	0.1678	NA	NA	NA	0.3797	NA	NA	0.1341

SNP	Allele	MXL	PEL	Pelotas	PJL	Puno	PUR	Qeros	Quechua	Salvador	Shimaa	Shipibo	STU	Tacna	Tallanes	Trujillo	TSI	Tumbes	Uros	YRI
rs187991938	G	0	0	NA	0	NA	0	NA	NA	NA	NA	NA	0	NA	NA	NA	0	NA	NA	0.03049
rs192781231	C	0	0.007752	NA	0	NA	0.01299	NA	NA	NA	NA	NA	0	NA	NA	NA	0	NA	NA	0.0122
rs4646185	T	0	0	NA	0	NA	0	NA	NA	NA	NA	NA	0	NA	NA	NA	0	NA	NA	0.006098
rs188959000	T	0	0	NA	0	NA	0	NA	NA	NA	NA	NA	0	NA	NA	NA	0	NA	NA	0
rs141554379	C	0.2604	0.1938	NA	0.4167	NA	0.487	NA	NA	NA	NA	NA	0.3826	NA	NA	NA	0.443	NA	NA	0.4878
rs147464721	A	0	0	NA	0	NA	0	NA	NA	NA	NA	NA	0	NA	NA	NA	0	NA	NA	0.006098
rs182777784	A	0	0	NA	0	NA	0	NA	NA	NA	NA	NA	0	NA	NA	NA	0	NA	NA	0
rs191640680	T	0	0	NA	0	NA	0	NA	NA	NA	NA	NA	0	NA	NA	NA	0	NA	NA	0
rs186597267	A	0	0.007752	NA	0	NA	0.01299	NA	NA	NA	NA	NA	0	NA	NA	NA	0	NA	NA	0.006098
rs146598386	T	0	0	NA	0.006944	NA	0.006494	NA	NA	NA	NA	NA	0	NA	NA	NA	0.006289	NA	NA	0
rs4646131	GA	0.1667	0.124	NA	0.2639	NA	0.3571	NA	NA	NA	NA	NA	0.1678	NA	NA	NA	0.3899	NA	NA	0.2195
rs145693360	T	0	0	NA	0	NA	0	NA	NA	NA	NA	NA	0	NA	NA	NA	0	NA	NA	0
rs373114523	C	0	0	NA	0	NA	0	NA	NA	NA	NA	NA	0	NA	NA	NA	0	NA	NA	0
rs184680265	A	0	0	NA	0	NA	0	NA	NA	NA	NA	NA	0	NA	NA	NA	0	NA	NA	0
rs140473595	T	0	0	NA	0	NA	0	NA	NA	NA	NA	NA	0	NA	NA	NA	0	NA	NA	0
rs191611107	A	0	0	NA	0	NA	0	NA	NA	NA	NA	NA	0	NA	NA	NA	0	NA	NA	0
rs190147327	G	0	0.007752	NA	0	NA	0.01299	NA	NA	NA	NA	NA	0	NA	NA	NA	0	NA	NA	0.006098
rs185202584	C	0	0	NA	0	NA	0	NA	NA	NA	NA	NA	0	NA	NA	NA	0	NA	NA	0
rs4646115	C	0	0	NA	0	NA	0	NA	NA	NA	NA	NA	0	NA	NA	NA	0	NA	NA	0.006098
rs190614788	T	0.02083	0	NA	0	NA	0	NA	NA	NA	NA	NA	0	NA	NA	NA	0.006289	NA	NA	0
rs367784090	A	0	0	NA	0	NA	0	NA	NA	NA	NA	NA	0	NA	NA	NA	0	NA	NA	0
rs59021449	A	0	0	NA	0	NA	0	NA	NA	NA	NA	NA	0	NA	NA	NA	0	NA	NA	0.006098
rs143779699	T	0	0	NA	0	NA	0	NA	NA	NA	NA	NA	0	NA	NA	NA	0	NA	NA	0
rs141025655	T	0	0	NA	0	NA	0	NA	NA	NA	NA	NA	0	NA	NA	NA	0	NA	NA	0.006098
rs4646122	G	0	0.0155	NA	0	NA	0.01948	NA	NA	NA	NA	NA	0	NA	NA	NA	0	NA	NA	0.1768
rs1514279	G	0.1667	0.124	0.2444	0.2639	NA	0.3571	NA	NA	0.2774	NA	NA	0.1678	NA	NA	NA	0.3899	NA	NA	0.2134
rs191513620	C	0	0	NA	0	NA	0	NA	NA	NA	NA	NA	0	NA	NA	NA	0	NA	NA	0
rs201900069	T	0	0	NA	0	NA	0	NA	NA	NA	NA	NA	0	NA	NA	NA	0	NA	NA	0
rs4646161	G	0.02083	0	NA	0	NA	0	NA	NA	NA	NA	NA	0	NA	NA	NA	0	NA	NA	0.04878
rs4646170	C	0	0.007752	0.02222	0	NA	0.006494	NA	NA	0.1241	NA	NA	0	NA	NA	NA	0	NA	NA	0.09146
rs139797356	G	0	0	NA	0	NA	0	NA	NA	NA	NA	NA	0	NA	NA	NA	0	NA	NA	0.006098
rs188512350	A	0	0	NA	0.02083	NA	0	NA	NA	NA	NA	NA	0	NA	NA	NA	0	NA	NA	0
rs183322022	T	0	0	NA	0	NA	0	NA	NA	NA	NA	NA	0	NA	NA	NA	0	NA	NA	0.006098
rs4646166	T	0	0.007752	NA	0	NA	0.006494	NA	NA	NA	NA	NA	0	NA	NA	NA	0	NA	NA	0.0122
rs183411746	T	0	0	NA	0	NA	0	NA	NA	NA	NA	NA	0	NA	NA	NA	0	NA	NA	0
rs4646152	A	0.1667	0.1163	0.2273	0.2639	NA	0.3506	NA	NA	0.1533	NA	NA	0.1678	NA	NA	NA	0.3711	NA	NA	0.128
rs188271923	C	0	0	NA	0	NA	0.006494	NA	NA	NA	NA	NA	0	NA	NA	NA	0	NA	NA	0
rs182847340	G	0	0	NA	0	NA	0	NA	NA	NA	NA	NA	0	NA	NA	NA	0	NA	NA	0
rs188556473	T	0	0	NA	0	NA	0	NA	NA	NA	NA	NA	0	NA	NA	NA	0	NA	NA	0

SNP	Allele	MXL	PEL	Pelotas	PJL	Puno	PUR	Qeros	Quechua	Salvador	Shimaa	Shipibo	STU	Tacna	Tallanes	Trujillo	TSI	Tumbes	Uros	YRI
rs4646137	T	0	0	NA	0	NA	0	NA	NA	NA	NA	NA	0	NA	NA	NA	0	NA	NA	0.0122
rs193035703	T	0	0	NA	0	NA	0	NA	NA	NA	NA	NA	0	NA	NA	NA	0	NA	NA	0
rs372629764	C	0	0	NA	0	NA	0	NA	NA	NA	NA	NA	0	NA	NA	NA	0	NA	NA	0
rs187300691	T	0	0	NA	0	NA	0	NA	NA	NA	NA	NA	0	NA	NA	NA	0	NA	NA	0
rs141377908	G	0	0	NA	0	NA	0	NA	NA	NA	NA	NA	0	NA	NA	NA	0	NA	NA	0
rs192715876	A	0	0	NA	0	NA	0	NA	NA	NA	NA	NA	0	NA	NA	NA	0	NA	NA	0
rs202137736	C	0	0	NA	0	NA	0	NA	NA	NA	NA	NA	0	NA	NA	NA	0	NA	NA	0
rs187846474	C	0.01042	0	NA	0	NA	0	NA	NA	NA	NA	NA	0	NA	NA	NA	0	NA	NA	0
rs183880501	C	0	0	NA	0	NA	0.006494	NA	NA	NA	NA	NA	0	NA	NA	NA	0	NA	NA	0
rs191961425	T	0	0	NA	0	NA	0	NA	NA	NA	NA	NA	0	NA	NA	NA	0	NA	NA	0
rs143459173	C	0	0	NA	0	NA	0	NA	NA	NA	NA	NA	0	NA	NA	NA	0	NA	NA	0.006098
rs187467963	T	0	0	NA	0	NA	0	NA	NA	NA	NA	NA	0	NA	NA	NA	0	NA	NA	0
rs184459616	A	0	0	NA	0	NA	0.006494	NA	NA	NA	NA	NA	0	NA	NA	NA	0	NA	NA	0
rs148805807	T	0	0	NA	0	NA	0	NA	NA	NA	NA	NA	0	NA	NA	NA	0	NA	NA	0.02439
rs199804629	T	0	0.007752	NA	0	NA	0	NA	NA	NA	NA	NA	0	NA	NA	NA	0	NA	NA	0
rs138390800	C	0	0	NA	0	NA	0	NA	NA	NA	NA	NA	0	NA	NA	NA	0	NA	NA	0
rs1514281	C	0.01042	0.0155	NA	0.0625	NA	0.05195	NA	NA	NA	NA	NA	0.1074	NA	NA	NA	0.006289	NA	NA	0.4146
rs148408803	T	0.01042	0.007752	NA	0.006944	NA	0.006494	NA	NA	NA	NA	NA	0.01342	NA	NA	NA	0.006289	NA	NA	0
rs181799530	T	0	0	NA	0	NA	0	NA	NA	NA	NA	NA	0	NA	NA	NA	0	NA	NA	0
rs192479337	C	0	0	NA	0	NA	0	NA	NA	NA	NA	NA	0	NA	NA	NA	0	NA	NA	0
rs181964653	A	0	0	NA	0	NA	0	NA	NA	NA	NA	NA	0	NA	NA	NA	0	NA	NA	0
rs113691336	C	0.1667	0.1085	NA	0.2778	NA	0.3377	NA	NA	NA	NA	NA	0.1678	NA	NA	NA	0.3711	NA	NA	0.122
rs184756568	T	0	0	NA	0	NA	0	NA	NA	NA	NA	NA	0	NA	NA	NA	0	NA	NA	0
rs184453641	A	0	0	NA	0	NA	0.03247	NA	NA	NA	NA	NA	0	NA	NA	NA	0	NA	NA	0
rs188558468	A	0	0	NA	0	NA	0	NA	NA	NA	NA	NA	0	NA	NA	NA	0	NA	NA	0
rs2301692	C	0.01042	0.007752	NA	0.0625	NA	0.03896	NA	NA	NA	NA	NA	0.1074	NA	NA	NA	0.006289	NA	NA	0.2012
rs189821013	A	0	0	NA	0	NA	0	NA	NA	NA	NA	NA	0	NA	NA	NA	0	NA	NA	0
rs189699255	T	0	0	NA	0	NA	0	NA	NA	NA	NA	NA	0	NA	NA	NA	0	NA	NA	0
rs4646153	C	0.1667	0.1163	0.2296	0.2639	NA	0.3506	NA	NA	0.1533	NA	NA	0.1678	NA	NA	NA	0.3836	NA	NA	0.128
rs182802402	C	0	0	NA	0	NA	0	NA	NA	NA	NA	NA	0	NA	NA	NA	0	NA	NA	0
rs186905240	T	0	0	NA	0	NA	0.006494	NA	NA	NA	NA	NA	0	NA	NA	NA	0	NA	NA	0
rs181989524	C	0	0	NA	0	NA	0	NA	NA	NA	NA	NA	0	NA	NA	NA	0	NA	NA	0
rs142060377	A	0	0	0	0	NA	0	NA	NA	0	NA	NA	0	NA	NA	NA	0	NA	NA	0
rs79473368	T	0	0	NA	0	NA	0	NA	NA	NA	NA	NA	0	NA	NA	NA	0	NA	NA	0
rs73635825	G	0	0	NA	0	NA	0	NA	NA	NA	NA	NA	0	NA	NA	NA	0	NA	NA	0.0122
rs141605946	T	0	0.007752	NA	0	NA	0	NA	NA	NA	NA	NA	0	NA	NA	NA	0	NA	NA	0
rs185220883	A	0	0	NA	0	NA	0.006494	NA	NA	NA	NA	NA	0	NA	NA	NA	0.006289	NA	NA	0
rs191099160	A	0	0.007752	NA	0	NA	0.01299	NA	NA	NA	NA	NA	0	NA	NA	NA	0	NA	NA	0.006098
rs4646169	A	0	0	0	0	NA	0.006494	NA	NA	0	NA	NA	0	NA	NA	NA	0	NA	NA	0.04268

SNP	Allele	MXL	PEL	Pelotas	PJL	Puno	PUR	Qeros	Quechua	Salvador	Shimaa	Shipibo	STU	Tacna	Tallanes	Trujillo	TSI	Tumbes	Uros	YRI
rs187738069	A	0	0	NA	0	NA	0	NA	NA	NA	NA	NA	0	NA	NA	NA	0	NA	NA	0
rs184589032	T	0	0	NA	0	NA	0	NA	NA	NA	NA	NA	0	NA	NA	NA	0	NA	NA	0
rs34481900	C	0.1667	0.1163	NA	0.2639	NA	0.3571	NA	NA	NA	NA	NA	0.1678	NA	NA	NA	0.3774	NA	NA	0.2073
rs188991352	C	0	0	NA	0	NA	0	NA	NA	NA	NA	NA	0	NA	NA	NA	0	NA	NA	0
rs201835187	AC	0	0	NA	0.006944	NA	0	NA	NA	NA	NA	NA	0	NA	NA	NA	0.01258	NA	NA	0
rs187161283	G	0	0	NA	0	NA	0	NA	NA	NA	NA	NA	0	NA	NA	NA	0	NA	NA	0
rs35803318	T	0.08333	0.1008	0.05926	0	0.06849	0.03896	0.125	NA	0.0365	0	0.03333	0	0.08696	0.1111	0.1	0.05031	0.1071	0.04167	0
rs143079256	T	0	0.007752	NA	0	NA	0.006494	NA	NA	NA	NA	NA	0	NA	NA	NA	0	NA	NA	0.03659
rs189944937	G	0.01042	0	NA	0	NA	0	NA	NA	NA	NA	NA	0	NA	NA	NA	0	NA	NA	0
rs4646147	T	0.1667	0.1163	NA	0.2639	NA	0.3506	NA	NA	NA	NA	NA	0.1611	NA	NA	NA	0.3711	NA	NA	0.1341
rs190133379	T	0	0	NA	0	NA	0	NA	NA	NA	NA	NA	0	NA	NA	NA	0	NA	NA	0
rs144523173	A	0	0	NA	0	NA	0	NA	NA	NA	NA	NA	0	NA	NA	NA	0	NA	NA	0
rs145502291	T	0	0	NA	0	NA	0	NA	NA	NA	NA	NA	0	NA	NA	NA	0	NA	NA	0
rs149958080	A	0	0	NA	0	NA	0	NA	NA	NA	NA	NA	0	NA	NA	NA	0	NA	NA	0
rs4646186	A	0	0	NA	0	NA	0.06494	NA	NA	NA	NA	NA	0	NA	NA	NA	0	NA	NA	0.1159
rs189142411	A	0	0	NA	0	NA	0.01948	NA	NA	NA	NA	NA	0	NA	NA	NA	0	NA	NA	0
rs4646127	A	0.1667	0.1163	NA	0.2639	NA	0.3571	NA	NA	NA	NA	NA	0.1678	NA	NA	NA	0.3774	NA	NA	0.2073
rs189140113	A	0	0	NA	0	NA	0	NA	NA	NA	NA	NA	0	NA	NA	NA	0	NA	NA	0
rs4646143	T	0.1667	0.1163	NA	0.2639	NA	0.3506	NA	NA	NA	NA	NA	0.1678	NA	NA	NA	0.3774	NA	NA	0.1341
rs41303171	C	0	0	0	0	NA	0.006494	NA	NA	0	NA	NA	0.006711	NA	NA	NA	0.01258	NA	NA	0
rs182808331	G	0	0	NA	0	NA	0.006494	NA	NA	NA	NA	NA	0	NA	NA	NA	0.006289	NA	NA	0
rs971249	T	0.1667	0.124	0.2444	0.2639	NA	0.3571	NA	NA	0.2774	NA	NA	0.1678	NA	NA	NA	0.3836	NA	NA	0.2134
rs186029035	A	0	0	NA	0	NA	0	NA	NA	NA	NA	NA	0	NA	NA	NA	0	NA	NA	0
rs184978092	G	0.01042	0	NA	0	NA	0	NA	NA	NA	NA	NA	0	NA	NA	NA	0	NA	NA	0
rs192180091	C	0	0	NA	0	NA	0.006494	NA	NA	NA	NA	NA	0	NA	NA	NA	0	NA	NA	0
rs34161673	C	0.01042	0	NA	0.006944	NA	0	NA	NA	NA	NA	NA	0	NA	NA	NA	0.01887	NA	NA	0
rs188496534	C	0	0	NA	0	NA	0	NA	NA	NA	NA	NA	0	NA	NA	NA	0	NA	NA	0
rs193005774	T	0	0	NA	0	NA	0	NA	NA	NA	NA	NA	0	NA	NA	NA	0	NA	NA	0
rs182372519	G	0	0	NA	0	NA	0.006494	NA	NA	NA	NA	NA	0	NA	NA	NA	0	NA	NA	0
rs151205966	G	0	0	NA	0	NA	0	NA	NA	NA	NA	NA	0	NA	NA	NA	0	NA	NA	0
rs182550682	A	0	0	NA	0	NA	0	NA	NA	NA	NA	NA	0	NA	NA	NA	0	NA	NA	0.006098
rs191332175	G	0	0	NA	0	NA	0.006494	NA	NA	NA	NA	NA	0	NA	NA	NA	0	NA	NA	0.0122
rs144061872	T	0	0	NA	0	NA	0	NA	NA	NA	NA	NA	0	NA	NA	NA	0.01258	NA	NA	0
rs189571339	C	0	0	NA	0	NA	0	NA	NA	NA	NA	NA	0	NA	NA	NA	0	NA	NA	0
rs138940089	T	0	0	NA	0	NA	0	NA	NA	NA	NA	NA	0	NA	NA	NA	0	NA	NA	0
rs190870013	C	0	0	NA	0	NA	0	NA	NA	NA	NA	NA	0	NA	NA	NA	0	NA	NA	0
rs188290800	C	0	0	NA	0	NA	0	NA	NA	NA	NA	NA	0	NA	NA	NA	0	NA	NA	0.006098
rs185622388	A	0	0	NA	0	NA	0.006494	NA	NA	NA	NA	NA	0	NA	NA	NA	0	NA	NA	0
rs190055656	T	0	0	NA	0.006944	NA	0	NA	NA	NA	NA	NA	0	NA	NA	NA	0	NA	NA	0

SNP	Allele	MXL	PEL	Pelotas	PJL	Puno	PUR	Qeros	Quechua	Salvador	Shimaa	Shipibo	STU	Tacna	Tallanes	Trujillo	TSI	Tumbes	Uros	YRI
rs138854040	A	0.04167	0.06202	NA	0.006944	NA	0.01299	NA	NA	NA	NA	NA	0.006711	NA	NA	NA	0	NA	NA	0
rs4646182	C	0.02083	0	NA	0	NA	0.006494	NA	NA	NA	NA	NA	0	NA	NA	NA	0	NA	NA	0.05488
rs181796402	T	0	0.007752	NA	0	NA	0.01299	NA	NA	NA	NA	NA	0	NA	NA	NA	0	NA	NA	0.006098
rs182366225	T	0	0	NA	0	NA	0	NA	NA	NA	NA	NA	0	NA	NA	NA	0	NA	NA	0
rs4646151	A	0	0	0.02222	0	NA	0.06494	NA	NA	0.0219	NA	NA	0	NA	NA	NA	0	NA	NA	0.1159
rs145890723	A	0	0	NA	0	NA	0	NA	NA	NA	NA	NA	0	NA	NA	NA	0	NA	NA	0.03049
rs191536933	T	0	0	NA	0	NA	0	NA	NA	NA	NA	NA	0	NA	NA	NA	0	NA	NA	0
rs151005246	A	0	0	NA	0	NA	0	NA	NA	NA	NA	NA	0	NA	NA	NA	0	NA	NA	0
rs182219706	T	0	0	NA	0	NA	0	NA	NA	NA	NA	NA	0	NA	NA	NA	0	NA	NA	0.006098
rs200217737	CA	0	0.007752	NA	0.006944	NA	0.006494	NA	NA	NA	NA	NA	0.01342	NA	NA	NA	0.006289	NA	NA	0.07317
rs375635972	T	0	0	NA	0	NA	0	NA	NA	NA	NA	NA	0.006711	NA	NA	NA	0	NA	NA	0
rs4646172	GA	0	0.007752	NA	0.006944	NA	0	NA	NA	NA	NA	NA	0	NA	NA	NA	0	NA	NA	0.04268
rs183661335	G	0	0.007752	NA	0	NA	0.01299	NA	NA	NA	NA	NA	0	NA	NA	NA	0	NA	NA	0.006098
rs181695790	G	0	0	NA	0	NA	0	NA	NA	NA	NA	NA	0	NA	NA	NA	0	NA	NA	0
rs188283144	C	0	0	NA	0	NA	0	NA	NA	NA	NA	NA	0	NA	NA	NA	0	NA	NA	0.006098
rs199951323	C	0	0	NA	0	NA	0	NA	NA	NA	NA	NA	0	NA	NA	NA	0	NA	NA	0
rs181788868	C	0	0.007752	NA	0	NA	0.01299	NA	NA	NA	NA	NA	0	NA	NA	NA	0	NA	NA	0.006098
rs72614595	T	0	0	NA	0	NA	0	NA	NA	NA	NA	NA	0	NA	NA	NA	0	NA	NA	0
rs182287132	A	0	0	NA	0	NA	0	NA	NA	NA	NA	NA	0	NA	NA	NA	0	NA	NA	0
rs181522566	C	0	0	NA	0.006944	NA	0	NA	NA	NA	NA	NA	0	NA	NA	NA	0	NA	NA	0
rs4646120	G	0.2292	0.1395	NA	0.2847	NA	0.4221	NA	NA	NA	NA	NA	0.1812	NA	NA	NA	0.5157	NA	NA	0.311
rs4646177	T	0	0	NA	0	NA	0	NA	NA	NA	NA	NA	0	NA	NA	NA	0	NA	NA	0.07927
rs371025504	G	0	0	NA	0	NA	0	NA	NA	NA	NA	NA	0	NA	NA	NA	0	NA	NA	0
rs185525294	A	0	0	NA	0	NA	0	NA	NA	NA	NA	NA	0	NA	NA	NA	0	NA	NA	0.006098
rs189278377	A	0	0	NA	0	NA	0	NA	NA	NA	NA	NA	0	NA	NA	NA	0	NA	NA	0
rs4646183	C	0	0	NA	0	NA	0	NA	NA	NA	NA	NA	0	NA	NA	NA	0	NA	NA	0.006098
rs188292876	C	0	0	NA	0	NA	0	NA	NA	NA	NA	NA	0	NA	NA	NA	0	NA	NA	0
rs186957036	C	0	0	NA	0	NA	0	NA	NA	NA	NA	NA	0	NA	NA	NA	0	NA	NA	0
rs4646121	T	0.02083	0	NA	0	NA	0.006494	NA	NA	NA	NA	NA	0	NA	NA	NA	0	NA	NA	0.07317
rs143102000	A	0	0	NA	0	NA	0	NA	NA	NA	NA	NA	0	NA	NA	NA	0	NA	NA	0.02439
rs185005111	T	0	0	NA	0	NA	0	NA	NA	NA	NA	NA	0	NA	NA	NA	0	NA	NA	0
rs113208650	T	0	0.007752	0.01481	0	0	0.006494	0	NA	0.1241	0	0	0	0	0	0.02	0	0.03571	0	0.09756
rs186914723	A	0	0	NA	0	NA	0	NA	NA	NA	NA	NA	0	NA	NA	NA	0.01258	NA	NA	0
rs4646150	T	0.09375	0.06977	NA	0	NA	0.09091	NA	NA	NA	NA	NA	0	NA	NA	NA	0	NA	NA	0
rs4646135	C	0	0.007752	0.01481	0	NA	0.006494	NA	NA	0.1241	NA	NA	0	NA	NA	NA	0	NA	NA	0.09146
rs184064003	A	0	0	NA	0	NA	0	NA	NA	NA	NA	NA	0	NA	NA	NA	0	NA	NA	0
rs4646187	A	0	0	NA	0	NA	0.006494	NA	NA	NA	NA	NA	0	NA	NA	NA	0	NA	NA	0
rs185723767	T	0	0	NA	0	NA	0	NA	NA	NA	NA	NA	0	NA	NA	NA	0	NA	NA	0
rs376237761	G	0	0	NA	0	NA	0	NA	NA	NA	NA	NA	0	NA	NA	NA	0	NA	NA	0

SNP	Allele	MXL	PEL	Pelotas	PJL	Puno	PUR	Qeros	Quechua	Salvador	Shimaa	Shipibo	STU	Tacna	Tallanes	Trujillo	TSI	Tumbes	Uros	YRI
rs373723722	T	0	0	NA	0	NA	0	NA	NA	NA	NA	NA	0	NA	NA	NA	0	NA	NA	0
rs61433707	T	0	0	0	0	NA	0	NA	NA	0.0146	NA	NA	0	NA	NA	NA	0	NA	NA	0.01829
rs201424204	C	0.02083	0.155	NA	0	NA	0.02597	NA	NA	NA	NA	NA	0	NA	NA	NA	0	NA	NA	0
rs146087218	T	0	0	NA	0	NA	0	NA	NA	NA	NA	NA	0	NA	NA	NA	0	NA	NA	0.006098
rs2301693	A	0.01042	0.007752	NA	0.0625	NA	0.03896	NA	NA	NA	NA	NA	0.1074	NA	NA	NA	0.006289	NA	NA	0.2012
rs186036909	T	0	0	NA	0	NA	0	NA	NA	NA	NA	NA	0	NA	NA	NA	0	NA	NA	0
rs191884180	G	0	0	NA	0	NA	0	NA	NA	NA	NA	NA	0	NA	NA	NA	0	NA	NA	0
rs148977224	C	0	0	NA	0	NA	0	NA	NA	NA	NA	NA	0	NA	NA	NA	0	NA	NA	0
rs190850356	A	0	0	NA	0	NA	0	NA	NA	NA	NA	NA	0	NA	NA	NA	0	NA	NA	0
rs192474956	G	0	0	NA	0	NA	0	NA	NA	NA	NA	NA	0	NA	NA	NA	0	NA	NA	0
rs56700224	T	0	0.007752	NA	0	NA	0.006494	NA	NA	NA	NA	NA	0	NA	NA	NA	0	NA	NA	0.07927
rs142017934	C	0	0	NA	0	NA	0	NA	NA	NA	NA	NA	0	NA	NA	NA	0	NA	NA	0.02439
rs41297301	T	0.01042	0	0.01481	0.006944	0	0.006494	0	NA	0	0	0	0.01342	0	0	0	0.006289	0	0	0
rs4646168	G	0	0.007752	0.02222	0	NA	0.01299	NA	NA	0.1241	NA	NA	0	NA	NA	NA	0	NA	NA	0.1341
rs2048683	T	0.1667	0.124	0.2444	0.2639	0.08219	0.3571	0.125	0.1707	0.2774	0	0.03333	0.1678	0.08696	0.1111	0.14	0.3836	0.3036	0.04167	0.2134
rs181786494	A	0	0	NA	0	NA	0	NA	NA	NA	NA	NA	0	NA	NA	NA	0	NA	NA	0.006098
rs4646124	T	0.1667	0.1163	0.2593	0.2639	NA	0.3571	NA	NA	0.2774	NA	NA	0.1678	NA	NA	NA	0.3774	NA	NA	0.2195
rs138529167	A	0	0	NA	0	NA	0	NA	NA	NA	NA	NA	0	NA	NA	NA	0	NA	NA	0
rs4646125	T	0	0	NA	0	NA	0	NA	NA	NA	NA	NA	0	NA	NA	NA	0	NA	NA	0.006098
rs4646162	G	0	0	NA	0	NA	0.006494	NA	NA	NA	NA	NA	0	NA	NA	NA	0	NA	NA	0.04268
rs180864908	G	0.02083	0.05426	NA	0	NA	0.01948	NA	NA	NA	NA	NA	0	NA	NA	NA	0	NA	NA	0
rs184178697	T	0	0	NA	0	NA	0	NA	NA	NA	NA	NA	0	NA	NA	NA	0	NA	NA	0
rs145954042	C	0	0	NA	0	NA	0	NA	NA	NA	NA	NA	0	NA	NA	NA	0.006289	NA	NA	0
rs4646149	T	0	0	NA	0	NA	0	NA	NA	NA	NA	NA	0	NA	NA	NA	0	NA	NA	0.006098
rs147312210	C	0	0	NA	0	NA	0	NA	NA	NA	NA	NA	0	NA	NA	NA	0	NA	NA	0.006098
rs149039346	G	0	0	NA	0	NA	0	NA	NA	NA	NA	NA	0	NA	NA	NA	0	NA	NA	0
rs192674337	G	0	0	NA	0.006944	NA	0	NA	NA	NA	NA	NA	0	NA	NA	NA	0	NA	NA	0
rs193290621	A	0	0	NA	0	NA	0	NA	NA	NA	NA	NA	0	NA	NA	NA	0	NA	NA	0
rs187390194	G	0	0	NA	0	NA	0	NA	NA	NA	NA	NA	0	NA	NA	NA	0	NA	NA	0
rs4646128	T	0	0.007752	NA	0	NA	0.006494	NA	NA	NA	NA	NA	0	NA	NA	NA	0	NA	NA	0.07927
rs4646133	G	0	0	NA	0	NA	0	NA	NA	NA	NA	NA	0	NA	NA	NA	0	NA	NA	0.006098
rs181620291	C	0	0	NA	0	NA	0	NA	NA	NA	NA	NA	0	NA	NA	NA	0	NA	NA	0.006098
rs182501431	G	0	0	NA	0	NA	0	NA	NA	NA	NA	NA	0	NA	NA	NA	0	NA	NA	0
rs184503057	T	0	0	NA	0	NA	0	NA	NA	NA	NA	NA	0	NA	NA	NA	0	NA	NA	0
rs181169181	C	0.01042	0	NA	0	NA	0	NA	NA	NA	NA	NA	0	NA	NA	NA	0	NA	NA	0
rs4646129	T	0	0.007752	NA	0	NA	0.006494	NA	NA	NA	NA	NA	0	NA	NA	NA	0	NA	NA	0.08537
rs185182583	A	0	0.007752	NA	0	NA	0.01299	NA	NA	NA	NA	NA	0	NA	NA	NA	0	NA	NA	0.006098
rs140083333	A	0	0	NA	0	NA	0	NA	NA	NA	NA	NA	0	NA	NA	NA	0	NA	NA	0
rs4646146	C	0	0	NA	0	NA	0.006494	NA	NA	NA	NA	NA	0	NA	NA	NA	0	NA	NA	0.07927

SNP	Allele	MXL	PEL	Pelotas	PJL	Puno	PUR	Qeros	Quechua	Salvador	Shimaa	Shipibo	STU	Tacna	Tallanes	Trujillo	TSI	Tumbes	Uros	YRI
rs186271730	T	0	0	NA	0	NA	0	NA	NA	NA	NA	NA	0	NA	NA	NA	0	NA	NA	0
rs189283596	C	0.01042	0	NA	0	NA	0	NA	NA	NA	NA	NA	0	NA	NA	NA	0	NA	NA	0
rs189944736	C	0	0	NA	0	NA	0	NA	NA	NA	NA	NA	0	NA	NA	NA	0	NA	NA	0
rs148006212	A	0	0	NA	0	NA	0	NA	NA	NA	NA	NA	0	NA	NA	NA	0	NA	NA	0.0122
rs372812138	T	0	0	NA	0	NA	0	NA	NA	NA	NA	NA	0	NA	NA	NA	0	NA	NA	0
rs4646173	A	0	0	NA	0	NA	0	NA	NA	NA	NA	NA	0	NA	NA	NA	0	NA	NA	0.006098
rs4646117	C	0	0	NA	0	NA	0	NA	NA	NA	NA	NA	0	NA	NA	NA	0	NA	NA	0.04268
rs187959864	T	0	0	NA	0	NA	0	NA	NA	NA	NA	NA	0	NA	NA	NA	0.006289	NA	NA	0
rs191869625	A	0.05208	0.09302	NA	0	NA	0.02597	NA	NA	NA	NA	NA	0	NA	NA	NA	0.01258	NA	NA	0
rs200885467	TA	0	0	NA	0.006944	NA	0.01299	NA	NA	NA	NA	NA	0	NA	NA	NA	0.006289	NA	NA	0
rs72614596	A	0.02083	0.155	NA	0	NA	0.02597	NA	NA	NA	NA	NA	0	NA	NA	NA	0	NA	NA	0
rs185721534	G	0.02083	0	NA	0	NA	0.006494	NA	NA	NA	NA	NA	0	NA	NA	NA	0	NA	NA	0.07317
rs183546232	G	0	0	NA	0.006944	NA	0	NA	NA	NA	NA	NA	0	NA	NA	NA	0	NA	NA	0
rs757066	C	0.1667	0.1085	0.2074	0.2639	NA	0.2727	NA	NA	0.1176	NA	NA	0.1678	NA	NA	NA	0.3836	NA	NA	0
rs186277162	C	0	0	NA	0	NA	0	NA	NA	NA	NA	NA	0	NA	NA	NA	0	NA	NA	0
rs4646157	C	0	0	NA	0	NA	0.006494	NA	NA	NA	NA	NA	0	NA	NA	NA	0	NA	NA	0.04878
rs2023802	G	0.1667	0.124	0.2519	0.2639	NA	0.3571	NA	NA	0.2774	NA	NA	0.1678	NA	NA	NA	0.3774	NA	NA	0.2195
rs200260858	C	0	0	NA	0.01389	NA	0.006494	NA	NA	NA	NA	NA	0.01342	NA	NA	NA	0	NA	NA	0.0122
rs191336106	G	0	0	NA	0	NA	0.01299	NA	NA	NA	NA	NA	0	NA	NA	NA	0	NA	NA	0.0122
rs150200247	A	0	0	NA	0	NA	0	NA	NA	NA	NA	NA	0	NA	NA	NA	0	NA	NA	0
rs187474513	T	0	0	NA	0	NA	0	NA	NA	NA	NA	NA	0	NA	NA	NA	0	NA	NA	0.01829
rs184874220	A	0	0	NA	0	NA	0	NA	NA	NA	NA	NA	0	NA	NA	NA	0	NA	NA	0
rs189810201	C	0	0	NA	0	NA	0	NA	NA	NA	NA	NA	0	NA	NA	NA	0	NA	NA	0.006098
rs184746393	C	0	0	NA	0	NA	0	NA	NA	NA	NA	NA	0	NA	NA	NA	0	NA	NA	0
rs72614598	C	0.02083	0.155	NA	0	NA	0.02597	NA	NA	NA	NA	NA	0	NA	NA	NA	0	NA	NA	0
rs180878567	C	0	0	NA	0	NA	0	NA	NA	NA	NA	NA	0	NA	NA	NA	0	NA	NA	0
rs200477770	G	0	0	NA	0	NA	0	NA	NA	NA	NA	NA	0	NA	NA	NA	0	NA	NA	0
rs199612962	G	0	0	NA	0	NA	0	NA	NA	NA	NA	NA	0	NA	NA	NA	0	NA	NA	0
rs200180615	T	0	0	NA	0	NA	0	NA	NA	NA	NA	NA	0	NA	NA	NA	0	NA	NA	0
rs192914427	T	0	0	NA	0	NA	0	NA	NA	NA	NA	NA	0	NA	NA	NA	0	NA	NA	0
rs181390351	T	0	0	NA	0	NA	0	NA	NA	NA	NA	NA	0	NA	NA	NA	0	NA	NA	0
rs4646178	G	0	0	NA	0	NA	0	NA	NA	NA	NA	NA	0	NA	NA	NA	0	NA	NA	0.07317
rs187251131	C	0	0	NA	0	NA	0	NA	NA	NA	NA	NA	0	NA	NA	NA	0	NA	NA	0
rs192692847	C	0	0	NA	0	NA	0	NA	NA	NA	NA	NA	0	NA	NA	NA	0	NA	NA	0
rs4646167	T	0	0	NA	0	NA	0.06494	NA	NA	NA	NA	NA	0	NA	NA	NA	0	NA	NA	0.122
rs189691652	T	0	0	NA	0	NA	0	NA	NA	NA	NA	NA	0	NA	NA	NA	0	NA	NA	0
rs138763015	G	0	0	NA	0	NA	0	NA	NA	NA	NA	NA	0	NA	NA	NA	0	NA	NA	0
rs372272603	A	0	0	NA	0	NA	0	NA	NA	NA	NA	NA	0	NA	NA	NA	0	NA	NA	0
rs184730109	A	0	0	NA	0	NA	0	NA	NA	NA	NA	NA	0	NA	NA	NA	0	NA	NA	0

SNP	Allele	MXL	PEL	Pelotas	PJL	Puno	PUR	Qeros	Quechua	Salvador	Shimaa	Shipibo	STU	Tacna	Tallanes	Trujillo	TSI	Tumbes	Uros	YRI
rs186261546	C	0	0	NA	0	NA	0.006494	NA	NA	NA	NA	NA	0	NA	NA	NA	0	NA	NA	0
