# Supplementary material for: Human-SARS-CoV-2 interactome and human genetic diversity: TMPRSS2-rs2070788, associated with severe influenza, and its population genetics caveats in Native Americans
Source: Genet Mol Biol. 2021 Aug 25;44(1 Suppl 1):e20200484. doi: 10.1590/1678-4685-GMB-2020-0484 (PMC8387978; doi:10.1590/1678-4685-GMB-2020-0484)
Supplement: Table S2-B - [file 1415-4757-GMB-44-1-s1-e20200484-s5.pdf]

Genetics and Molecular Biology
https://doi.org/10.1590/1678-4685-GMB-2020-0484

Supplementary Material to "Human-SARS-CoV-2 interactome and human genetic diversity: *TMPRSS2*-rs2070788, associated with severe influenza, and its population genetics caveats in Native Americans"

Table S2-B – *ACE2* regression values

			rs4646123	rs147311723	rs4646188	rs1514283	rs4646156	rs714205	rs4646179	rs233575	rs2074192	rs4646174	rs1514282	rs4646181	rs4646176
AFR															
	Intercept		-<br>3.07613036	-3.13452999	-<br>2.04948926	-<br>3.03806069	-<br>0.50996702	-<br>0.87645404	-<br>3.14145764	-<br>0.95099225	-<br>0.28664794	-<br>0.62264202	-2.9373986	-<br>3.10398438	-<br>2.95418595
	P-Value		0	0	3.09E-46	5.00E-166	0.05737494	1.08E-12	5.71E-115	1.06E-07	2.52E-05	0.00190172	8.24E-305	0	1.42E-220
	Beta		1.06754	0.40452	-1.15529	2.69699	-0.06012	-1.0223	0.99124	-1.50698	-0.30593	-0.93272	2.53126	0.37575	1.50098
	P-Value		< 0.001	7.00E-05	0.00678	< 0.001	0.92224	0.00239	4.00E-05	0.004	0.0648	0.0397	< 0.001	0.00072	< 0.001
	R^2		0.93315	0.5734	0.47665	0.93022	0.00073	0.50463	0.4715	0.49477	0.17771	0.20296	0.96339	0.48538	0.89304
	AICc		-81.92453	-78.26686	-48.21422	-70.88349	0.68987	-28.20216	-63.29457	-21.31186	-39.85123	-7.39978	-59.35233	-75.84562	-57.78564
EUR															
	Intercept		-<br>2.33220995	-2.87171434	-3.4607587	-<br>1.46764014	-<br>1.20792554	-1.1780686	-<br>2.39855526	-<br>2.72729956	-<br>0.37085615	0.56697498	-<br>1.13585711	-<br>2.87173534	-<br>1.84810748
	P-Value		1.33E-70	3.58E-238	3.38E-83	1.90E-06	0.00209313	4.32E-06	1.49E-64	3.72E-45	0.00061616	0.08953239	4.81E-05	8.59E-212	8.05E-26
	Beta		-0.78125	-0.27866	1.77585	-1.10039	1.17194	0.11851	-0.95657	2.2509	0.02254	-0.32619	-1.61625	-0.23702	-1.18838
	P-Value		0.00031	0.03592	< 0.001	0.03026	0.03543	0.75029	0.00019	< 0.001	0.89102	0.50314	0.00101	0.09033	0.00011
	R^2		0.58125	0.29002	0.88515	0.34939	0.27135	0.00767	0.55759	0.8962	0.00115	0.02834	0.59258	0.20531	0.64121
	AICc		-55.29447	-72.65244	-67.35949	-32.83597	-3.69892	-19.73742	-63.4777	-45.57127	-36.74457	-4.07763	-23.80807	-70.94465	-42.03524
NAT															
	Intercept		-<br>2.72145492	-2.99701886	-<br>2.26528667	-<br>1.89873437	-<br>0.49866399	-<br>1.23882088	-<br>2.93689986	-<br>1.36800421	-<br>0.41773304	0.305285	-<br>1.80212181	-<br>2.97409732	-<br>2.35574465
	P-Value		4.88E-79	0	3.78E-34	1.22E-13	0.05173169	2.94E-16	8.28E-81	5.88E-09	7.21E-11	0.13896109	1.05E-09	0	3.28E-31
	Beta		-0.06581	-0.29672	-0.26782	-0.83484	-0.31787	1.43779	0.65118	1.03747	0.41027	0.8752	-1.15413	-0.31141	-0.80106
	P-Value		0.93649	0.48265	0.81807	0.34127	0.84551	0.09852	0.10264	0.44018	0.05734	0.51721	0.49637	0.46329	0.51726
	R^2		0.00054	0.04731	0.00486	0.10488	0.00322	0.14182	0.11874	0.03941	0.1774	0.03042	0.06285	0.05172	0.04773
	AICc		-46.58976	-69.54794	-40.89271	-29.54723	0.65678	-21.95662	-55.99376	-13.12396	-39.92862	-4.08485	-16.84819	-69.01689	-33.00005
EAS															
	Intercept		-<br>2.69389776	-3.00956849	-<br>2.23400945	-<br>1.97852757	-<br>0.35206182	-<br>1.27308772	-<br>2.79650989	-<br>1.18279638	-<br>0.36721705	0.19352553	-<br>1.86180948	-<br>2.98526108	-2.4095532
	P-Value		7.80E-96	0	7.16E-45	3.52E-15	0.06319871	1.43E-34	1.00E-81	6.32E-10	1.51E-08	0.13431551	5.85E-11	0	3.32E-35

			rs4646123	rs147311723	rs4646188	rs1514283	rs4646156	rs714205	rs4646179	rs233575	rs2074192	rs4646174	rs1514282	rs4646181	rs4646176
	**Beta**		-0.35563	-0.12461	-0.75737	-0.32716	-2.18495	1.48247	-0.3661	-1.31437	0.10627	2.4487	-0.33078	-0.15172	-0.10487
	**P-Value**		0.47058	0.59425	0.29879	0.72166	0.02685	1.00E-05	0.53349	0.15062	0.6786	0.00259	0.71895	0.52033	0.8715
	**R^2**		0.05982	0.02837	0.12902	0.0129	0.48488	0.46441	0.04172	0.24464	0.01051	0.6339	0.01723	0.04062	0.00275
	**AICc**		-47.17002	-69.3434	-42.299	-28.67568	-5.98211	-30.90981	-54.61748	-15.58358	-36.89549	-15.6033	-16.49924	-68.89585	-32.58669
**AFR+NAT**															
	**Intercept**		- 3.16780866	-3.13249907	- 1.92542538	- 3.00008411	- 0.46725873	- 0.96537804	- 3.44208496	- 0.95486419	- 0.34876454	0.59075757	- 2.95189259	- 3.09759837	-2.9751187
	**P-Value**		0	0	4.75E-34	6.99E-119	0.14982096	2.61E-11	1.45E-191	9.31E-06	4.50E-06	0.01511274	1.23E-206	0	5.60E-153
	**Beta**	AFR	1.15541	0.40259	-1.28549	2.65668	-0.10043	-0.94866	1.30113	-1.50359	-0.23736	-0.90324	2.54572	0.36966	1.52163
		NAT	0.74187	-0.01777	-1.22186	-0.21373	-0.38743	0.79724	1.19882	0.03565	0.31935	0.29105	0.11973	-0.05608	0.17551
	**P-Value**		< 0.001	0.00018	0.00169	< 0.001	0.87494	0.00462	< 0.001	0.00486	0.14296	0.05494	< 0.001	0.00152	< 0.001
			< 0.001	0.95397	0.19721	0.59612	0.81897	0.2635	< 0.001	0.97424	0.13141	0.81915	0.79233	0.86654	0.74054
	**R^2**		0.99446	0.57357	0.54742	0.93249	0.00515	0.54138	0.79958	0.49484	0.27569	0.20595	0.96363	0.48699	0.89456
	**AICc**		-104.61537	-73.5559	-46.3265	-67.57576	4.44818	-25.52148	-71.2102	-17.49482	-38.31377	-3.64018	-54.7094	-71.15999	-53.18072
**AFR+EUR**															
	**Intercept**		- 2.96255214	-3.13452307	- 3.42812556	- 3.03587601	-2.1865906	0.12600052	- 2.70068351	- 2.54089021	-0.1352936	2.11536669	- 2.68615557	- 3.13744634	- 2.66945874
	**P-Value**		3.62E-254	7.30E-129	8.26E-29	2.13E-34	0.00237945	0.34197901	2.70E-42	3.20E-16	0.32451429	1.70E-05	1.03E-58	6.35E-109	8.95E-44
	**Beta**	AFR	0.9548	0.40452	-0.05097	2.69485	1.50502	-2.06165	0.57056	-0.31097	-0.44631	-2.3915	2.28185	0.40876	1.2172
		EUR	-0.15397	-1.00E-05	1.74148	-0.00302	2.20274	-1.34535	-0.69476	2.05681	-0.21884	-1.93212	-0.34225	0.04499	-0.38931
	**P-Value**		< 0.001	0.00933	0.89721	< 0.001	0.07318	< 0.001	0.02343	0.44835	0.02116	2.00E-05	< 0.001	0.01599	< 0.001
			0.15591	0.99995	< 0.001	0.99224	0.00871	< 0.001	0.01184	< 0.001	0.21176	0.00071	0.10379	0.79555	0.11102
	**R^2**		0.944	0.5734	0.88556	0.93027	0.52044	0.87464	0.65241	0.89431	0.24856	0.70332	0.97317	0.48933	0.92556
	**AICc**		-79.11841	-73.55257	-63.55626	-67.24723	-3.65499	-49.03257	-63.82863	-42.39232	-37.68652	-15.62802	-56.85065	-71.1986	-55.17977
**AFR+EAS**															
	**Intercept**		- 3.07077603	-3.13528788	- 1.88846759	- 3.06586213	- 0.21617265	-1.053995	- 3.13235259	- 0.65407073	- 0.28856333	0.37370176	- 2.98678078	-3.099371	- 3.00996409
	**P-Value**		0	0	8.92E-64	1.61E-150	0.35241581	5.79E-37	3.91E-99	2.46E-19	8.28E-05	0.01194087	1.98E-301	0	2.16E-213
	**Beta**	AFR	1.0618	0.40536	-1.5341	2.72641	-0.47923	-0.8561	0.98051	-2.28239	-0.3035	-0.63295	2.58256	0.37064	1.55824
		EUR	-0.03842	0.00528	-1.43579	0.27424	-2.40275	1.24185	-0.07215	-2.72536	0.01676	2.26832	0.34001	-0.0324	0.37269
	**P-Value**		< 0.001	0.00012	1.00E-05	< 0.001	0.35477	5.00E-05	8.00E-05	< 0.001	0.07277	0.04817	< 0.001	0.0012	< 0.001
			0.7636	0.97475	0.0089	0.45601	0.01649	< 0.001	0.87656	< 0.001	0.94416	0.00291	0.11024	0.85874	0.13274
	**R^2**		0.93378	0.57341	0.7553	0.93723	0.52006	0.79053	0.47414	0.94291	0.17798	0.71151	0.97237	0.4873	0.92101
	**AICc**		-77.30371	-73.55356	-53.36051	-67.72441	-3.07232	-39.96083	-59.27474	-46.94559	-36.21977	-15.30496	-56.6546	-71.16313	-54.86302
**NAT+EUR**															

			rs4646123	rs147311723	rs4646188	rs1514283	rs4646156	rs714205	rs4646179	rs233575	rs2074192	rs4646174	rs1514282	rs4646181	rs4646176
	**Intercept**		-2.32179082	-2.84466965	-3.41120092	-1.00354591	-1.18081359	-1.32684651	-2.4552421	-3.01087169	-0.49853901	0.48758229	-1.02628119	-2.84320955	-1.78466442
	**P-Value**		1.23E-64	8.63E-229	4.98E-68	0.000124	0.00473682	4.11E-07	2.26E-49	1.39E-64	1.42E-05	0.16235247	0.00012167	1.08E-202	3.54E-26
	**Beta**	NAT	-0.14277	-0.32024	-0.35088	-1.84913	-0.28058	1.46451	0.21693	1.58682	0.47618	0.8497	-1.59039	-0.332	-0.97307
		EUR	-0.77741	-0.27884	1.74386	-1.66669	1.1667	0.14728	-0.91321	2.4539	0.13201	-0.31184	-1.59731	-0.23819	-1.16113
	**P-Value**		0.80237	0.36673	0.56841	0.0135	0.85114	0.09235	0.48835	0.00017	0.03483	0.52702	0.24029	0.37614	0.2433
			0.00031	0.0284	< 0.001	0.00011	0.03615	0.67834	0.00059	< 0.001	0.39856	0.51555	0.00049	0.07577	5.00E-05
	**R^2**		0.58381	0.34615	0.88533	0.65909	0.27274	0.15205	0.56764	0.9503	0.21252	0.05671	0.6699	0.26474	0.70227
	**AICc**		-50.64487	-68.77685	-63.83596	-36.74198	0.07948	-18.31057	-59.86589	-50.85057	-36.98557	-0.69905	-20.85007	-67.04428	-38.93955
NAT+EAS															
	**Intercept**		-2.67964296	-2.97973062	-2.19677074	-1.8557014	-0.27751149	-1.44201148	-2.9081381	-1.25665476	-0.43380085	0.06486976	-1.75251232	-2.95365708	-2.33634371
	**P-Value**		1.59E-72	0	4.22E-33	2.07E-12	0.209024	7.40E-51	4.40E-73	1.71E-08	1.04E-10	0.64438566	1.17E-08	0	1.40E-27
	**Beta**	NAT	-0.15563	-0.33064	-0.44145	-0.90725	-0.84524	1.72094	0.61722	0.82213	0.42973	1.48185	-1.28074	-0.35135	-0.84387
		EAS	-0.36456	-0.1446	-0.77456	-0.47063	-2.21716	1.60929	-0.28845	-1.30083	0.16517	2.49048	-0.41798	-0.17258	-0.15923
	**P-Value**		0.849	0.43037	0.69863	0.30406	0.54303	0.00065	0.12244	0.51299	0.04502	0.10686	0.45304	0.40107	0.49818
			0.45959	0.52631	0.28406	0.60469	0.02341	< 0.001	0.61272	0.15621	0.47675	0.00089	0.64501	0.45158	0.8026
	**R^2**		0.06241	0.08505	0.14054	0.12912	0.50142	0.66586	0.14389	0.26637	0.20211	0.7006	0.08866	0.10403	0.05375
	**AICc**		-42.48947	-65.251	-38.64748	-26.2025	-2.59016	-35.24318	-52.22413	-12.20128	-36.78381	-14.52571	-12.36335	-64.89426	-28.3501
EUR+EAS															
	**Intercept**		-2.04269864	-2.77190405	-3.51773285	-1.21774816	-0.77767591	-1.65545968	-2.17061618	-2.64249981	-0.39898868	-0.08465578	-0.55182142	-2.77242324	-1.5405615
	**P-Value**		0	1.79E-229	1.80E-66	7.25E-05	0.04403226	5.45E-17	2.78E-110	5.12E-37	0.00102265	0.73899591	0.00054962	1.58E-196	9.73E-34
	**Beta**	EUR	-1.17451	-0.39596	1.83776	-1.45365	0.67699	0.59513	-1.30685	2.15967	0.05419	0.44379	-2.69556	-0.35306	-1.63706
		EAS	-1.11232	-0.3714	0.24802	-1.24271	-1.83661	1.85934	-1.10455	-0.44421	0.13708	2.75864	-2.17276	-0.37181	-1.14476
	**P-Value**		< 0.001	0.00135	< 0.001	0.00388	0.20163	0.02477	< 0.001	< 0.001	0.75712	0.21176	< 0.001	0.00785	< 0.001
			< 0.001	0.05008	0.56554	0.15106	0.07362	< 0.001	0.00041	0.41743	0.61749	0.00079	2.00E-05	0.07021	0.00066
	**R^2**		0.97504	0.48228	0.88563	0.47126	0.54635	0.63488	0.80195	0.90675	0.01627	0.66871	0.84846	0.39453	0.82889
	**AICc**		-85.79195	-71.53637	-63.8411	-31.75707	-3.83067	-32.19494	-71.32232	-42.58802	-33.35342	-13.2847	-33.97905	-69.36316	-47.21904
AFR+NAT+EUR															
	**Intercept**		-3.13618057	-3.12956667	-3.259036	-2.7912029	-2.57335773	0.1609313	-3.27291511	-3.27630096	-0.27485344	2.5765384	-2.65262981	-3.12985677	-2.64463218
	**P-Value**		0	9.23E-94	1.60E-18	2.08E-16	0.00600362	0.29394441	3.35E-29	1.38E-15	0.18582975	0.00019926	2.73E-44	5.15E-79	2.57E-33
	**Beta**	AFR	1.12411	0.3997	-0.20667	2.44967	1.87747	-2.0945	1.13623	0.33662	-0.30833	-2.84182	2.2485	0.40139	1.19259
		NAT	0.71515	-0.02012	-0.50472	-0.41577	1.14742	-0.14981	1.03302	1.87404	0.24874	-1.24857	-0.13617	-0.03021	-0.10582
		EUR	-0.03842	-0.00359	1.59305	-0.24005	2.52698	-1.36919	-0.2035	2.71747	-0.08705	-2.3319	-0.36558	0.03941	-0.40607

			rs4646123	rs147311723	rs4646188	rs1514283	rs4646156	rs714205	rs4646179	rs233575	rs2074192	rs4646174	rs1514282	rs4646181	rs4646176
			< 0.001	0.02148	0.63342	< 0.001	0.06531	< 0.001	0.00032	0.4557	0.2101	0.00012	< 0.001	0.03431	< 0.001
	**P-Value**		< 0.001	0.95096	0.46456	0.39922	0.48243	0.66048	0.0027	0.0012	0.37488	0.2609	0.7505	0.93224	0.83299
			0.34719	0.98312	4.00E-05	0.51966	0.01047	< 0.001	0.53122	< 0.001	0.70258	0.00123	0.0914	0.83099	0.10706
	**R^2**		0.99492	0.57356	0.88613	0.93809	0.55778	0.8748	0.80357	0.95564	0.28211	0.74474	0.97353	0.48973	0.92611
	**AICc**		-99.16885	-67.27063	-59.38264	-63.58046	0.50235	-44.56161	-66.50077	-46.80198	-34.09419	-12.25202	-50.67188	-64.92014	-48.94054
**AFR+NAT+EAS**															
	**Intercept**		- 3.17459733	-3.13315625	-1.6659868	- 3.03124658	- 0.04636574	- 1.20825782	- 3.47641591	- 0.55882435	- 0.36190503	0.24463616	- 3.01821461	- 3.09044411	- 3.05070391
	**P-Value**		0	0	1.74E-72	1.21E-103	0.86692055	1.84E-56	1.22E-161	8.45E-14	1.36E-05	0.15782834	2.17E-202	0	7.87E-147
		AFR	1.16252	0.40329	-1.79973	2.68971	-0.64953	-0.72531	1.33973	-2.38087	-0.22128	-0.50991	2.61409	0.36197	1.59866
	**Beta**	NAT	0.75356	-0.01653	-2.09775	-0.17573	-1.37954	1.21936	1.23652	-0.8434	0.3358	1.08331	0.22941	-0.06963	0.30024
		EAS	0.03842	0.00359	-1.59305	0.24003	-2.52707	1.3692	0.20351	-2.71753	0.08706	2.33195	0.36559	-0.03942	0.40607
	**P-Value**		< 0.001	0.00033	< 0.001	< 0.001	0.21618	1.00E-05	< 0.001	< 0.001	0.18437	0.11013	< 0.001	0.00289	< 0.001
			< 0.001	0.95793	0.00044	0.66512	0.32586	0.00025	< 0.001	0.02986	0.1185	0.22162	0.58727	0.83736	0.54564
			0.34719	0.98312	4.00E-05	0.5197	0.01047	< 0.001	0.53119	< 0.001	0.70256	0.00123	0.0914	0.83098	0.10706
	**R^2**		0.99492	0.57356	0.88612	0.93809	0.55779	0.87479	0.80357	0.95564	0.28212	0.74475	0.97353	0.48973	0.92611
	**AICc**		-99.16887	-67.27063	-59.38228	-63.58039	0.50208	-44.56121	-66.50083	-46.80277	-34.09421	-12.25226	-50.67186	-64.92015	-48.94051
**AFR+EUR+EAS**															
	**Intercept**		- 2.42102282	-3.14968791	- 3.76380645	- 3.20695712	- 1.42584445	0.0111364	- 2.23989484	- 1.40221364	-0.0261118	1.32792145	- 2.78879262	- 3.16006427	- 2.75045033
	**P-Value**		0	1.55E-29	1.02E-11	3.18E-20	0.25979945	0.96982204	5.75E-38	7.20E-05	0.88550173	0.0967092	6.12E-14	1.57E-25	3.15E-10
		AFR	0.40895	0.41982	0.2981	2.86542	0.72995	-1.94471	0.10321	-1.53748	-0.55707	-1.5932	2.38467	0.43159	1.29841
	**Beta**	EUR	-0.75357	0.01653	2.09782	0.17571	1.37946	-1.21939	-1.23651	0.84338	-0.33579	-1.08328	-0.22942	0.06962	-0.30025
		EAS	-0.71516	0.02012	0.50479	0.41574	-1.14758	0.14979	-1.03302	-1.87413	-0.24874	1.24866	0.13616	0.03021	0.10582
	**P-Value**		< 0.001	0.15321	0.62998	< 0.001	0.58868	< 0.001	0.62309	0.00023	0.01361	0.06054	< 0.001	0.17593	0.00385
			< 0.001	0.95792	0.00044	0.66516	0.3259	0.00025	< 0.001	0.02986	0.11851	0.22164	0.58726	0.83738	0.54562
			< 0.001	0.95096	0.46451	0.39926	0.48237	0.66052	0.0027	0.0012	0.37489	0.26087	0.75051	0.93226	0.833
	**R^2**		0.99492	0.57356	0.88613	0.93809	0.55778	0.8748	0.80356	0.95564	0.28211	0.74475	0.97353	0.48973	0.92611
	**AICc**		-99.16857	-67.27063	-59.38276	-63.58033	0.50223	-44.56157	-66.50052	-46.80253	-34.09413	-12.25215	-50.67187	-64.92014	-48.94054
**NAT+EUR+EAS**															
	**Intercept**		- 2.01207599	-2.7298639	- 3.46571506	- 0.34153374	- 0.69588395	- 1.93356074	- 2.13668885	- 2.93967691	- 0.58318357	- 0.26527748	- 0.40412756	- 2.72846913	- 1.45204139
	**P-Value**		0	2.85E-241	7.53E-53	0.00128854	0.0874388	9.17E-48	3.66E-75	5.16E-53	7.36E-06	0.27627011	2.19E-10	3.29E-203	4.60E-55
		NAT	-0.40894	-0.41982	-0.29805	-2.86544	-0.73003	1.94465	-0.1032	1.53742	0.55707	1.59321	-2.38463	-0.4316	-1.2984
	**Beta**	EUR	-1.16252	-0.40329	1.79973	-2.68971	0.64951	0.7253	-1.33972	2.38085	0.22128	0.50991	-2.61408	-0.36197	-1.59866
		EAS	-1.12412	-0.39971	0.2067	-2.44971	-1.87755	2.09451	-1.13622	-0.33665	0.30834	2.84187	-2.24853	-0.40139	-1.1926

			rs4646123	rs147311723	rs4646188	rs1514283	rs4646156	rs714205	rs4646179	rs233575	rs2074192	rs4646174	rs1514282	rs4646181	rs4646176
	**P-Value**		< 0.001	0.15321	0.63004	< 0.001	0.58864	< 0.001	0.62311	0.00023	0.01361	0.06054	< 0.001	0.17592	0.00385
			< 0.001	0.00033	< 0.001	< 0.001	0.21619	1.00E-05	< 0.001	< 0.001	0.18436	0.11013	< 0.001	0.00289	< 0.001
			< 0.001	0.02148	0.63338	< 0.001	0.0653	< 0.001	0.00032	0.45567	0.21009	0.00012	< 0.001	0.03431	< 0.001
	**R^2**		0.99492	0.57356	0.88613	0.9381	0.55779	0.87479	0.80356	0.95564	0.28212	0.74475	0.97353	0.48973	0.92612
	**AICc**		-99.1684	-67.27062	-59.38269	-63.58125	0.50215	-44.56101	-66.50049	-46.80208	-34.09426	-12.25232	-50.67206	-64.9202	-48.94063
AFR+NAT+EUR+EAS															
	**Intercept**		-736.946424	-634.880634	-12679.7831	30152.7996	47674.7197	9769.43137	-16915.2453	10027.5408	-9479.94545	-17198.3112	4622.59751	5385.35684	6155.46707
	**P-Value**		0.69667965	0.93633022	0.19332552	0.00703368	0.07997813	0.20946731	0.13035612	0.20037026	0.27763342	0.3292824	0.67750491	0.51734244	0.63906286
	**Beta**	AFR	734.93467	632.15117	12676.3783	-30153.1683	-47675.5528	-9771.39825	16913.1445	-10030.5451	9479.38859	17198.0932	-4623.00299	-5388.08907	-6156.92129
		NAT	734.51442	631.72184	12675.8887	-30155.9438	-47675.5398	-9769.26769	16912.9573	-10028.7964	9479.88858	17199.4098	-4625.31582	-5388.44057	-6158.12454
		EUR	733.76993	631.74569	12678.0803	-30155.8287	-47674.6668	-9770.62391	16911.7053	-10028.0769	9479.55613	17198.5253	-4625.60566	-5388.43237	-6158.50335
		EAS	733.8173	631.75716	12676.6303	-30155.8857	-47677.8178	-9769.36459	16912.1129	-10030.903	9479.75688	17201.0503	-4625.29529	-5388.53909	-6158.17205
	**P-Value**		0.69747	0.9366	0.19345	0.00703	0.07997	0.20938	0.1304	0.20024	0.27766	0.32929	0.67748	0.51713	0.63898
			0.69763	0.93665	0.19346	0.00703	0.07997	0.20947	0.13041	0.20031	0.27763	0.32925	0.67732	0.5171	0.63891
			0.69792	0.93664	0.19338	0.00703	0.07998	0.20941	0.13044	0.20035	0.27765	0.32928	0.67731	0.5171	0.6389
			0.69791	0.93664	0.19344	0.00703	0.07996	0.20947	0.13043	0.20022	0.27765	0.32921	0.67733	0.5171	0.63892
	**R^2**		0.99496	0.57367	0.90259	0.94058	0.63289	0.88678	0.82666	0.96008	0.33347	0.76122	0.974	0.51228	0.92878
	**AICc**		-90.51714	-58.4769	-55.05798	-64.04645	3.6318	-40.21135	-62.21748	-42.52008	-29.90558	-7.36408	-42.05499	-56.52243	-40.37059

Table S2-B – *ACE2* regression continuation.

			rs1978124	rs6632677	rs4646171	rs35803318	rs879922	rs41303171	rs2106809	rs4646142	rs4646155	rs4646140	rs4240157	rs2285666	rs2158083
AFR															
	**Intercept**		-0.54867	-2.73484	-3.07594	-2.4993	0.616272	-3.06421	-0.75024	-0.73556	-2.95573	-2.84322	1.006163	-0.77744	-0.93058
	**P-Value**		0.007334	1.07E-99	7.36E-186	1.78E-102	0.002117	0	5.38E-15	4.16E-09	1.16E-244	1.54E-56	3.16E-07	4.20E-12	1.92E-07
	**Beta**		-0.99395	-0.27059	1.66198	-0.72061	-0.92272	-0.34106	-1.26909	-0.48759	1.41984	1.12992	-1.29593	-0.54509	-0.17501
	**P-Value**		0.05776	0.36542	< 0.001	0.02858	0.04196	0.02708	< 0.001	0.09591	< 0.001	0.00036	0.00314	0.0505	0.67482
	**R^2**		0.21298	0.08973	0.84364	0.33441	0.19914	0.27967	0.71281	0.22301	0.88572	0.36453	0.2768	0.24481	0.00983
	**AICc**		-9.47251	-50.8084	-71.3425	-66.6676	-7.34955	-95.3844	-34.5433	-18.6209	-59.2294	-52.2656	-11.8735	-28.0601	-15.602
EUR															
	**Intercept**		-2.13165	-2.60196	-1.8427	-3.19248	0.569183	-3.44942	-1.26073	-0.72704	-1.90555	-2.06606	1.044783	-0.80094	-1.74988
	**P-Value**		2.52E-37	2.05E-52	3.83E-23	1.83E-82	0.087621	0	5.40E-07	0.000238	1.19E-30	1.73E-17	0.001168	2.48E-05	4.33E-17
	**Beta**		2.16961	-0.36693	-1.27642	0.91853	-0.33626	0.50304	0.38757	-0.22417	-1.14282	-0.7784	-0.68409	-0.18952	1.27217

	**P-Value**		< 0.001	0.16076	7.00E-05	5.00E-05	0.48907	1.00E-05	0.28179	0.4443	7.00E-05	0.04351	0.14802	0.50157	1.00E-05
	**R^2**		0.83214	0.1812	0.59387	0.61454	0.03014	0.597	0.07837	0.05151	0.64765	0.20219	0.10809	0.03245	0.50062
	**AICc**		-39.0973	-51.8084	-54.7734	-74.2708	-4.13261	-103.961	-20.0649	-16.5177	-44.1292	-47.9617	-7.06255	-24.8167	-29.3481
NAT															
	**Intercept**		-0.8102	-2.89441	-2.41714	-2.6333	0.302116	-3.11496	-1.16845	-0.94187	-2.39745	-2.41004	0.423582	-1.00721	-0.96643
	**P-Value**		0.000297	4.64E-117	8.99E-35	8.61E-86	0.142494	0	2.55E-15	4.78E-13	2.07E-35	6.85E-37	0.011681	2.59E-18	2.30E-08
	**Beta**		0.3038	0.98852	-0.89974	-0.10515	0.86936	-0.3704	1.505	0.99447	-0.75486	-0.90701	1.82828	1.11544	-0.09572
	**P-Value**		0.82632	0.10991	0.49272	0.81779	0.51923	0.41396	0.07794	0.16339	0.52248	0.47765	0.01298	0.10453	0.93033
	**R^2**		0.00343	0.1688	0.04464	0.00548	0.03009	0.0522	0.15302	0.13801	0.04562	0.05301	0.46166	0.1461	5.00E-04
	**AICc**		-5.83919	-51.9326	-44.7004	-61.4111	-4.10514	-91.6051	-21.5289	-17.6543	-34.7343	-45.3206	-13.2449	-26.658	-15.4288
EAS															
	**Intercept**		-0.63863	-2.96314	-2.47459	-2.60924	0.189313	-3.12639	-1.16494	-0.99761	-2.44978	-2.46571	0.545998	-1.03609	-0.82527
	**P-Value**		5.77E-05	0	1.02E-41	3.34E-107	0.140679	0	3.74E-24	1.98E-37	5.54E-40	3.06E-44	0.001261	4.35E-48	2.55E-21
	**Beta**		-1.96417	1.10255	-0.1522	-0.56742	2.45388	-0.2338	1.25836	1.19682	-0.08458	-0.18316	1.69367	1.24122	-2.2927
	**P-Value**		0.02765	< 0.001	0.82339	0.30941	0.00244	0.35471	0.0011	< 0.001	0.89099	0.7844	0.05743	< 0.001	0.00046
	**R^2**		0.50911	0.66615	0.00414	0.10274	0.63731	0.06588	0.33878	0.629	0.00191	0.00584	0.29991	0.5838	0.78527
	**AICc**		-13.2348	-64.977	-44.2406	-62.7299	-15.7607	-91.8107	-26.285	-28.5966	-34.3241	-44.7988	-10.0391	-38.5164	-32.9918
AFR+NAT															
	**Intercept**		-0.505	-2.84003	-3.09895	-2.42576	0.58435	-2.99433	-0.82963	-0.81792	-2.97738	-2.81334	0.722489	-0.86625	-0.9057
	**P-Value**		0.04048	2.67E-81	3.32E-124	4.83E-78	0.016254	0	8.03E-14	1.83E-08	5.27E-170	7.70E-39	0.000102	4.29E-11	2.72E-05
	**Beta**	AFR	-1.03527	-0.18326	1.68473	-0.81482	-0.8932	-0.40842	-1.20498	-0.41573	1.44115	1.10032	-0.98021	-0.4668	-0.19845
		NAT	-0.39948	0.86823	0.19998	-0.3953	0.29134	-0.66997	0.71781	0.71331	0.18176	-0.26928	1.51993	0.79406	-0.22759
	**P-Value**		0.05388	0.5342	< 0.001	0.01273	0.0578	0.00401	1.00E-05	0.15436	< 0.001	0.00108	0.00823	0.09173	0.64542
			0.75765	0.1717	0.76173	0.31852	0.81898	0.07453	0.19359	0.29718	0.71774	0.8115	0.02294	0.22376	0.84027
	**R^2**		0.21768	0.207	0.84509	0.39688	0.20215	0.42834	0.74152	0.28703	0.88756	0.36911	0.57289	0.31147	0.01243
	**AICc**		-5.75965	-47.6149	-67.6199	-64.3535	-3.59006	-94.7749	-32.2593	-14.9106	-54.6454	-48.5126	-15.4535	-25.5936	-11.8297
AFR+EUR															
	**Intercept**		-2.52778	-1.88092	-2.79291	-3.05478	2.112533	-3.48279	0.037173	0.140451	-2.67009	-2.77926	2.573605	0.150373	-2.61359
	**P-Value**		1.54E-18	5.81E-193	2.10E-28	3.06E-40	1.63E-05	3.66E-135	0.722807	0.220684	1.55E-49	1.67E-09	1.12E-22	0.2246	7.69E-18
	**Beta**	AFR	0.64961	-1.19215	1.38146	-0.26732	-2.38461	0.05033	-2.07362	-1.39259	1.13506	1.06768	-2.77689	-1.47763	1.35328
		EUR	2.58036	-1.2279	-0.36992	0.77787	-1.93742	0.53854	-1.04834	-1.20665	-0.3907	-0.08385	-2.1638	-1.23755	2.16103
	**P-Value**		0.05942	< 0.001	< 0.001	0.42224	2.00E-05	0.77417	< 0.001	< 0.001	< 0.001	0.04089	< 0.001	< 0.001	1.00E-04
			< 0.001	< 0.001	0.2359	0.00479	0.00066	0.00113	< 0.001	< 0.001	0.0883	0.88034	< 0.001	< 0.001	< 0.001
	**R^2**		0.90229	0.92305	0.86868	0.63446	0.70395	0.59828	0.93397	0.87121	0.92182	0.36494	0.90231	0.83914	0.84567
	**AICc**		-39.1514	-77.7283	-68.7551	-71.3139	-15.6883	-100.221	-55.3735	-36.5688	-56.9004	-48.469	-38.9643	-48.8942	-38.861

AFR+EAS															
	**Intercept**		-0.19415	-2.93214	-3.11859	-2.41227	0.366178	-3.01936	-0.88069	-0.90949	-3.01164	-2.86673	0.8565	-0.93349	-0.651
	**P-Value**		0.027254	8.45E-274	1.58E-171	2.62E-115	0.013468	0	2.42E-39	4.02E-28	1.03E-240	1.37E-49	2.19E-06	9.06E-34	1.79E-15
	**Beta**	AFR	-1.71326	-0.11394	1.70751	-0.86882	-0.62139	-0.40671	-1.13464	-0.32475	1.47722	1.15749	-1.1142	-0.37906	-0.63211
		EUR	-3.15667	1.06955	0.35459	-0.86612	2.27523	-0.37475	0.9434	1.09943	0.37249	0.18495	1.4665	1.12786	-2.70048
	**P-Value**		< 0.001	0.51635	< 0.001	0.0033	0.05173	0.00417	< 0.001	0.06835	< 0.001	4.00E-04	0.00414	0.03336	0.00093
			< 0.001	< 0.001	0.26456	0.06019	0.00276	0.07216	< 0.001	< 0.001	0.11028	0.74233	0.06964	< 0.001	< 0.001
	**R^2**		0.91639	0.67976	0.86481	0.53556	0.71223	0.42824	0.87448	0.71862	0.91642	0.3682	0.47686	0.6936	0.87903
	**AICc**		-34.2667	-60.7028	-68.5447	-67.7955	-15.3654	-94.8913	-44.7728	-26.9942	-56.5192	-48.544	-13.0151	-38.9586	-37.7383
NAT+EUR															
	**Intercept**		-2.22176	-2.68927	-1.7736	-3.32616	0.490343	-3.41209	-1.4323	-0.82028	-1.84544	-1.9803	0.555958	-0.90315	-1.75119
	**P-Value**		2.81E-34	1.28E-55	4.66E-23	5.56E-63	0.159185	0	1.79E-08	3.87E-05	3.09E-31	1.19E-16	0.063706	2.22E-06	9.51E-15
	**Beta**	NAT	0.60409	0.91644	-1.08621	0.43312	0.84287	-0.36232	1.58842	0.96474	-0.91411	-1.07792	1.73298	1.08792	0.01114
		EUR	2.22436	-0.37066	-1.25028	1.04254	-0.32195	0.48965	0.43483	-0.21479	-1.11752	-0.78741	-0.21798	-0.17762	1.27275
	**P-Value**		0.24691	0.10418	0.24148	0.22111	0.5293	0.2289	0.05292	0.16565	0.23992	0.34391	0.02173	0.10793	0.98822
			< 0.001	0.13356	3.00E-05	2.00E-05	0.50113	< 0.001	0.19823	0.43846	3.00E-05	0.03176	0.5937	0.50446	1.00E-05
	**R^2**		0.84049	0.33785	0.65094	0.65502	0.05812	0.63729	0.23955	0.18373	0.7064	0.26278	0.47269	0.17383	0.50063
	**AICc**		-36.5391	-49.222	-52.721	-72.1589	-0.74928	-101.553	-19.3056	-13.5209	-41.0415	-45.3134	-9.90078	-23.2735	-25.5301
NAT+EAS															
	**Intercept**		-0.62879	-3.09797	-2.39614	-2.58271	0.061183	-3.09038	-1.33436	-1.1246	-2.38092	-2.38704	0.203517	-1.1647	-0.77796
	**P-Value**		0.00077	0	2.06E-31	1.59E-83	0.661327	0	3.09E-32	3.02E-79	3.11E-31	2.45E-33	0.032586	1.60E-86	5.33E-15
	**Beta**	NAT	-0.11321	1.22209	-0.95413	-0.17857	1.47481	-0.42514	1.74552	1.25978	-0.79052	-0.96031	2.39994	1.35597	-0.56495
		EAS	-1.96615	1.21315	-0.20699	-0.59689	2.49481	-0.25487	1.3822	1.29101	-0.13536	-0.23386	2.39799	1.33376	-2.27949
	**P-Value**		0.92133	< 0.001	0.47051	0.68682	0.10606	0.33564	0.00371	2.00E-05	0.50606	0.45533	< 0.001	3.00E-05	0.38022
			0.0273	< 0.001	0.75737	0.28494	0.00082	0.29617	1.00E-05	< 0.001	0.82342	0.72108	3.00E-05	< 0.001	0.00029
	**R^2**		0.5093	0.92196	0.05216	0.11604	0.70363	0.13165	0.54436	0.84425	0.0503	0.06313	0.86753	0.79873	0.79845
	**AICc**		-9.42715	-77.5783	-40.9819	-59.3052	-14.6955	-88.9743	-28.8129	-34.4903	-30.0709	-41.637	-30.5938	-45.8313	-30.0163
EUR+EAS															
	**Intercept**		-1.86406	-2.90904	-1.50408	-3.169	-0.08162	-3.4712	-1.72027	-1.07796	-1.6193	-1.82866	0.742573	-1.14845	-1.33409
	**P-Value**		2.35E-51	1.19E-110	1.18E-26	5.75E-67	0.747118	0	2.11E-18	4.95E-13	1.10E-40	1.52E-13	0.018832	4.94E-15	2.84E-28
	**Beta**	EUR	1.87176	-0.08753	-1.78415	0.89364	0.43221	0.52742	0.85781	0.12835	-1.55167	-1.10426	-0.33397	0.17758	0.79176
		EAS	-1.29497	1.04882	-1.32379	-0.13478	2.75474	0.08932	1.80511	1.27617	-1.06538	-0.89452	1.52303	1.35222	-1.9501
	**P-Value**		< 0.001	0.62635	< 0.001	2.00E-04	0.22212	2.00E-05	0.001	0.52618	< 0.001	0.00411	0.45855	0.38004	< 0.001
			0.00204	< 0.001	0.00091	0.76554	0.00078	0.63654	< 0.001	< 0.001	0.00071	0.1476	0.09828	< 0.001	3.00E-05
	**R^2**		0.96125	0.67381	0.7799	0.61556	0.67035	0.60305	0.67487	0.64358	0.82875	0.28905	0.31962	0.60878	0.91945

	**AICc**		-46.4759	-60.5202	-61.7124	-70.7329	-13.3787	-100.352	-31.9574	-24.306	-49.119	-46.4561	-6.96234	-35.5151	-43.7946
**AFR+NAT+EUR**															
	**Intercept**		-3.20647	-1.88463	-2.77203	-3.31562	2.575687	-3.37091	0.037982	0.1644	-2.64666	-2.67579	2.615641	0.166638	-3.23033
	**P-Value**		3.39E-16	2.33E-156	1.25E-21	3.75E-17	0.000192	5.01E-105	0.755541	0.217128	1.11E-37	2.51E-07	3.26E-09	0.248113	4.90E-14
		AFR	1.28596	-1.1887	1.36081	-0.01404	-2.83688	-0.05609	-2.07437	-1.41549	1.11185	0.96632	-2.81877	-1.49308	1.94629
	**Beta**	NAT	1.64173	0.02086	-0.08453	0.42257	-1.25319	-0.40533	-0.00346	-0.10127	-0.10006	-0.38632	-0.06462	-0.06868	1.53385
		EUR	3.19417	-1.22625	-0.38458	1.03204	-2.33895	0.44919	-1.0489	-1.22313	-0.40651	-0.16351	-2.205	-1.24878	2.7141
			0.00197	< 0.001	1.00E-05	0.97543	0.00011	0.7576	< 0.001	< 0.001	< 0.001	0.09005	< 0.001	< 0.001	1.00E-05
	**P-Value**		0.00285	0.90856	0.89748	0.39312	0.25747	0.21995	0.98976	0.7295	0.83175	0.74255	0.91023	0.82858	0.01226
			< 0.001	< 0.001	0.23523	0.01299	0.00115	0.00699	< 0.001	< 0.001	0.08561	0.77467	< 0.001	< 0.001	< 0.001
	**R^2**		0.96319	0.92352	0.86898	0.6552	0.74575	0.64001	0.93396	0.87156	0.92235	0.37151	0.90105	0.83901	0.92624
	**AICc**		-42.7026	-71.4563	-64.1063	-67.7962	-12.333	-96.9787	-50.707	-30.4035	-50.6619	-43.9213	-34.6158	-44.275	-40.1698
**AFR+NAT+EAS**															
	**Intercept**		-0.0123	-3.11087	-3.1566	-2.28357	0.236729	-2.92172	-1.01092	-1.05873	-3.05317	-2.83931	0.410634	-1.08214	-0.51623
	**P-Value**		0.859553	0	2.68E-113	6.68E-99	0.170078	0	9.99E-67	2.06E-53	1.55E-166	3.27E-33	3.03E-05	4.90E-55	4.82E-11
		AFR	-1.90822	0.03755	1.74538	-1.0461	-0.49792	-0.50529	-1.02546	-0.19235	1.51837	1.12984	-0.61375	-0.24429	-0.76782
	**Beta**	NAT	-1.5524	1.24709	0.30004	-0.60949	1.08573	-0.85452	1.04543	1.12184	0.30644	-0.2228	2.14039	1.18008	-1.18022
		EAS	-3.19416	1.22626	0.38457	-1.03208	2.33901	-0.4492	1.04891	1.22314	0.40651	0.16354	2.20503	1.24879	-2.71412
	**P-Value**		< 0.001	0.68826	< 0.001	0.00011	0.11744	2.00E-05	< 0.001	0.12749	< 0.001	0.00136	0.00098	0.07061	< 0.001
			2.00E-05	< 0.001	0.64112	0.06038	0.21866	0.0057	6.00E-05	9.00E-05	0.5111	0.84548	< 0.001	0.00013	0.00508
			< 0.001	< 0.001	0.23525	0.01299	0.00115	0.00699	< 0.001	< 0.001	0.08561	0.77464	< 0.001	< 0.001	< 0.001
	**R^2**		0.96318	0.92352	0.86898	0.6552	0.74575	0.64001	0.93396	0.87156	0.92235	0.37151	0.90105	0.83901	0.92624
	**AICc**		-42.7014	-71.4558	-64.1062	-67.7965	-12.3332	-96.9786	-50.7063	-30.4032	-50.6618	-43.9213	-34.6158	-44.2746	-40.1697
**AFR+EUR+EAS**															
	**Intercept**		-1.56474	-1.86376	-2.85652	-2.89304	1.322444	-3.77625	0.034541	0.063138	-2.74672	-3.06218	2.551026	0.097972	-1.69646
	**P-Value**		2.51E-06	3.44E-30	5.38E-07	1.96E-24	0.09668	4.65E-41	0.881659	0.802032	2.18E-11	0.002824	7.90E-14	0.719397	9.34E-06
		AFR	-0.35577	-1.20956	1.4453	-0.43662	-1.58364	0.34924	-2.07093	-1.31422	1.21191	1.35271	-2.75415	-1.42441	0.41243
	**Beta**	EUR	1.55244	-1.24711	-0.30009	0.60947	-1.08571	0.85453	-1.04546	-1.12187	-0.30645	0.22288	-2.14039	-1.18012	1.18023
		EAS	-1.64171	-0.02087	0.08448	-0.4226	1.25328	0.40534	0.00344	0.10126	0.10006	0.38641	0.06462	0.06866	-1.53387
	**P-Value**		0.33306	< 0.001	0.01338	0.23885	0.06102	0.2492	< 0.001	< 0.001	0.00409	0.20213	< 0.001	< 0.001	0.31476
			2.00E-05	< 0.001	0.64107	0.06039	0.21868	0.0057	6.00E-05	9.00E-05	0.51109	0.84543	< 0.001	0.00013	0.00508
			0.00285	0.90853	0.89754	0.39309	0.25744	0.21995	0.98983	0.72952	0.83177	0.74249	0.91023	0.82862	0.01226
	**R^2**		0.96319	0.92352	0.86898	0.6552	0.74575	0.64001	0.93396	0.87156	0.92235	0.37151	0.90105	0.83901	0.92624
	**AICc**		-42.702	-71.4563	-64.1063	-67.7963	-12.3331	-96.9787	-50.707	-30.4034	-50.6619	-43.9213	-34.6158	-44.275	-40.1697
**NAT+EUR+EAS**															

	**Intercept**		-1.92052	-3.07332	-1.41122	-3.32965	-0.26119	-3.427	-2.03638	-1.25108	-1.53481	-1.70947	-0.20312	-1.32643	-1.28404
	**P-Value**		1.68E-46	0	9.05E-36	1.54E-47	0.282084	1.81E-287	8.61E-78	1.21E-35	2.52E-66	2.93E-13	0.153731	3.17E-36	8.58E-25
		NAT	0.35579	1.20954	-1.44532	0.4366	1.58365	-0.34923	2.07087	1.3142	-1.2119	-1.35262	2.75414	1.42437	-0.41242
	**Beta**	EUR	1.90822	-0.03756	-1.74538	1.04608	0.49792	0.50529	1.02545	0.19235	-1.51836	-1.12983	0.61375	0.24429	0.76781
		EAS	-1.28593	1.1887	-1.36083	0.01402	2.83693	0.0561	2.07437	1.4155	-1.11187	-0.9663	2.81878	1.49308	-1.9463
	**P-Value**		0.33302	< 0.001	0.01338	0.23887	0.06102	0.24921	< 0.001	< 0.001	0.00409	0.20215	< 0.001	< 0.001	0.31476
			< 0.001	0.68825	< 0.001	0.00011	0.11744	2.00E-05	< 0.001	0.12749	< 0.001	0.00136	0.00098	0.07062	< 0.001
			0.00197	< 0.001	1.00E-05	0.97548	0.00011	0.75759	< 0.001	< 0.001	< 0.001	0.09006	< 0.001	< 0.001	1.00E-05
	**R^2**		0.96319	0.92352	0.86899	0.6552	0.74575	0.64001	0.93395	0.87156	0.92235	0.37151	0.90105	0.83901	0.92624
	**AICc**		-42.7021	-71.4558	-64.1066	-67.7962	-12.3333	-96.9787	-50.7057	-30.4032	-50.662	-43.9211	-34.6158	-44.2745	-40.1697
**AFR+NAT+EUR+EAS**															
	**Intercept**		-10438	6777.721	22246.27	19839.64	-16331.9	-1554.32	13254.49	5154.396	6323.023	-31617.4	151.2778	9384.93	-884.962
	**P-Value**		0.136395	0.221551	0.062136	0.067729	0.353697	0.79519	0.012873	0.499119	0.607344	0.157718	0.988672	0.175106	0.91519
		AFR	10436.16	-6780.8	-22247.7	-19843.1	16331.7	1550.903	-13256.6	-5155.65	-6324.56	31615.78	-151.481	-9386.28	883.681
		NAT	10436.32	-6779.47	-22248.8	-19842.5	16333.02	1550.528	-13254.3	-5154.25	-6325.67	31613.83	-148.726	-9384.69	883.2538
	**Beta**	EUR	10438	-6780.82	-22249.4	-19841.9	16332.13	1551.399	-13255.5	-5155.44	-6326.06	31614.51	-150.867	-9386	884.4443
		EAS	10434.89	-6779.67	-22249.3	-19843.1	16334.65	1550.967	-13254.6	-5154.28	-6325.73	31615.01	-148.663	-9384.85	881.7385
			0.13647	0.22134	0.06212	0.06768	0.35371	0.79563	0.01286	0.49902	0.60726	0.15774	0.98866	0.17504	0.91531
			0.13645	0.22142	0.0621	0.06769	0.35366	0.79568	0.01287	0.49912	0.60719	0.15776	0.98886	0.17511	0.91535
	**P-Value**		0.1364	0.22134	0.0621	0.0677	0.35369	0.79557	0.01287	0.49903	0.60717	0.15776	0.9887	0.17506	0.91524
			0.13652	0.22142	0.0621	0.06768	0.35362	0.79562	0.01287	0.49913	0.60719	0.15775	0.98887	0.17511	0.9155
	**R^2**		0.96936	0.93002	0.88682	0.68176	0.76064	0.64303	0.95447	0.87531	0.92554	0.44771	0.90113	0.85559	0.92629
	**AICc**		-38.9692	-64.0958	-61.3191	-65.1957	-7.35571	-91.2103	-49.9393	-22.0452	-42.1376	-40.2824	-29.2827	-40.1434	-34.3476
