# Supplementary material for: Human-SARS-CoV-2 interactome and human genetic diversity: TMPRSS2-rs2070788, associated with severe influenza, and its population genetics caveats in Native Americans
Source: Genet Mol Biol. 2021 Aug 25;44(1 Suppl 1):e20200484. doi: 10.1590/1678-4685-GMB-2020-0484 (PMC8387978; doi:10.1590/1678-4685-GMB-2020-0484)
Supplement: Table S3-A - [file 1415-4757-GMB-44-1-s1-e20200484-s6.pdf]

Genetics and Molecular Biology
https://doi.org/10.1590/1678-4685-GMB-2020-0484

Supplementary Material to "Human-SARS-CoV-2 interactome and human genetic diversity: *TMPRSS2*-rs2070788, associated with severe influenza, and its population genetics caveats in Native Americans"

Table S3-A – BSG allele frequencies. Bold = functionally relevant SNPs found in our databases; NA = missing data

SNP	Allele	ACB	Afro	Aimaras	Ancash	Arequipa	Ashaninkas	ASW	Awajun	Ayacucho	Bambui	BEB	Candoshi	CDX	CEU	Chachapoyas	CHB	Chileans	Chopccas	CHS	CLM	Cusco	ESN	FIN	GBR	GIH	GWD	IBS	Iquitos
**rs8259**	**T**	**NA**	**NA**	**NA**	**NA**	**NA**	**NA**	**NA**	**NA**	**NA**	**0.460**	**NA**	**NA**	**NA**	**NA**	**NA**	**NA**	**0.35**	**NA**	**NA**	**NA**	**NA**	**NA**	**NA**	**NA**	**NA**	**NA**	**NA**	**NA**
**rs6757**	**C**	**0.141**	**0.160**	**0.031**	**0.014**	**0.000**	**0.029**	**0.246**	**0.022**	**0.029**	**0.108**	**0.314**	**0.094**	**0.253**	**0.045**	**0.013**	**0.233**	**NA**	**0.000**	**0.243**	**0.096**	**0.028**	**0.248**	**0.162**	**0.066**	**0.228**	**0.270**	**0.084**	**0.038**
**rs8637**	**A**	**0.057**	**NA**	**NA**	**NA**	**NA**	**NA**	**0.098**	**NA**	**NA**	**NA**	**0.157**	**NA**	**0.231**	**0.606**	**NA**	**0.301**	**0.2**	**NA**	**0.229**	**0.372**	**NA**	**0.000**	**0.546**	**0.560**	**0.141**	**0.031**	**0.481**	**NA**
**rs12608994**	**T**	**0.167**	**NA**	**NA**	**NA**	**NA**	**NA**	**0.254**	**NA**	**NA**	**0.114**	**0.320**	**NA**	**0.258**	**0.066**	**NA**	**0.233**	**NA**	**NA**	**0.243**	**0.106**	**NA**	**0.242**	**0.162**	**0.082**	**0.257**	**0.270**	**0.108**	**NA**
**rs1803202**	**T**	**0.141**	**NA**	**NA**	**NA**	**NA**	**NA**	**0.238**	**NA**	**NA**	**0.105**	**0.320**	**NA**	**0.258**	**0.066**	**NA**	**0.233**	**NA**	**NA**	**0.243**	**0.106**	**NA**	**0.242**	**0.162**	**0.082**	**0.252**	**0.270**	**0.108**	**NA**
**rs11879069**	**A**	**0.333**	**0.367**	**0.406**	**0.375**	**0.296**	**0.279**	**0.262**	**0.348**	**0.441**	**0.337**	**0.361**	**0.219**	**0.457**	**0.167**	**0.288**	**0.398**	**0.65**	**0.412**	**0.462**	**0.213**	**0.414**	**0.313**	**0.147**	**0.198**	**0.369**	**0.376**	**0.220**	**0.135**
**rs4919862**	**T**	**0.026**	**NA**	**NA**	**NA**	**NA**	**NA**	**0.033**	**NA**	**NA**	**NA**	**0.093**	**NA**	**0.231**	**0.278**	**NA**	**0.252**	**0.1562**	**NA**	**0.219**	**0.197**	**NA**	**0.000**	**0.222**	**0.192**	**0.068**	**0.031**	**0.266**	**NA**
**rs6758**	**A**	**0.141**	**NA**	**NA**	**NA**	**NA**	**NA**	**0.230**	**NA**	**NA**	**NA**	**0.314**	**NA**	**0.231**	**0.045**	**NA**	**0.223**	**NA**	**NA**	**0.219**	**0.096**	**NA**	**0.242**	**0.162**	**0.066**	**0.223**	**0.270**	**0.084**	**NA**
**rs28992474**	**T**	**0.141**	**NA**	**NA**	**NA**	**NA**	**NA**	**0.238**	**NA**	**NA**	**NA**	**0.320**	**NA**	**0.258**	**0.066**	**NA**	**0.233**	**NA**	**NA**	**0.243**	**0.106**	**NA**	**0.242**	**0.162**	**0.082**	**0.252**	**0.270**	**0.108**	**NA**
**rs2072310**	**T**	**0.333**	**NA**	**NA**	**NA**	**NA**	**NA**	**0.254**	**NA**	**NA**	**NA**	**0.349**	**NA**	**0.457**	**0.162**	**NA**	**0.398**	**0.55**	**NA**	**0.457**	**0.213**	**NA**	**0.313**	**0.147**	**0.192**	**0.364**	**0.376**	**0.210**	**NA**
**rs3764937**	**A**	**0.318**	**NA**	**NA**	**NA**	**NA**	**NA**	**0.336**	**NA**	**NA**	**NA**	**0.337**	**NA**	**0.258**	**0.066**	**NA**	**0.238**	**NA**	**NA**	**0.243**	**0.128**	**NA**	**0.389**	**0.157**	**0.082**	**0.311**	**0.305**	**0.112**	**NA**
**rs4682**	**C**	**0.333**	**NA**	**NA**	**NA**	**NA**	**NA**	**0.262**	**NA**	**NA**	**NA**	**0.361**	**NA**	**0.457**	**0.167**	**NA**	**0.398**	**0.65**	**NA**	**0.462**	**0.213**	**NA**	**0.313**	**0.147**	**0.198**	**0.369**	**0.376**	**0.220**	**NA**
**rs2072309**	**A**	**0.323**	**0.330**	**0.438**	**0.431**	**0.304**	**0.271**	**0.221**	**0.348**	**0.443**	**0.339**	**0.355**	**0.219**	**0.452**	**0.167**	**0.288**	**0.388**	**0.65**	**0.412**	**0.462**	**0.218**	**0.431**	**0.288**	**0.147**	**0.198**	**0.364**	**0.367**	**0.220**	**0.135**
**rs2074962**	**A**	**0.162**	**NA**	**NA**	**NA**	**NA**	**NA**	**0.205**	**NA**	**NA**	**NA**	**0.238**	**NA**	**0.231**	**0.657**	**NA**	**0.306**	**0.1786**	**NA**	**0.229**	**0.399**	**NA**	**0.066**	**0.586**	**0.599**	**0.209**	**0.097**	**0.519**	**NA**
rs568500243	T	0.010	NA	NA	NA	NA	NA	0.008	NA	NA	NA	0.000	NA	0.000	0.000	NA	0.000	NA	NA	0.000	0.000	NA	0.010	0.000	0.000	0.000	0.000	0.000	NA
rs565860293	A	0.000	NA	NA	NA	NA	NA	0.000	NA	NA	NA	0.000	NA	0.000	0.000	NA	0.000	NA	NA	0.005	0.000	NA	0.000	0.000	0.000	0.000	0.000	0.000	NA
rs369456038	T	0.000	NA	NA	NA	NA	NA	0.000	NA	NA	NA	0.000	NA	0.005	0.000	NA	0.000	NA	NA	0.000	0.000	NA	0.000	0.000	0.000	0.000	0.000	0.000	NA
rs2108832	C	NA	NA	NA	NA	NA	NA	NA	NA	NA	0.341	NA	NA	NA	NA	NA	NA	0.65	NA	NA	NA	NA	NA	NA	NA	NA	NA	NA	NA
rs193268910	T	0.016	NA	NA	NA	NA	NA	0.008	NA	NA	NA	0.000	NA	0.000	0.000	NA	0.000	NA	NA	0.000	0.005	NA	0.000	0.000	0.000	0.000	0.004	0.000	NA
rs576903171	A	0.000	NA	NA	NA	NA	NA	0.000	NA	NA	NA	0.000	NA	0.000	0.000	NA	0.000	NA	NA	0.000	0.000	NA	0.000	0.005	0.000	0.000	0.000	0.000	NA
rs376295294	A	0.000	NA	NA	NA	NA	NA	0.000	NA	NA	NA	0.000	NA	0.000	0.000	NA	0.000	NA	NA	0.000	0.000	NA	0.005	0.000	0.000	0.000	0.000	0.000	NA
rs188261956	A	0.000	NA	NA	NA	NA	NA	0.000	NA	NA	NA	0.000	NA	0.000	0.000	NA	0.000	NA	NA	0.000	0.000	NA	0.005	0.000	0.000	0.000	0.000	0.000	NA
rs140055452	T	0.010	NA	NA	NA	NA	NA	0.008	NA	NA	NA	0.000	NA	0.000	0.000	NA	0.000	NA	NA	0.000	0.000	NA	0.005	0.000	0.000	0.000	0.000	0.005	NA
rs552833814	T	0.000	NA	NA	NA	NA	NA	0.000	NA	NA	NA	0.000	NA	0.000	0.000	NA	0.000	NA	NA	0.000	0.000	NA	0.000	0.000	0.000	0.000	0.000	0.000	NA
rs531651313	A	0.000	NA	NA	NA	NA	NA	0.000	NA	NA	NA	0.000	NA	0.000	0.000	NA	0.000	NA	NA	0.000	0.000	NA	0.000	0.000	0.000	0.000	0.000	0.000	NA
rs551644758	G	0.000	NA	NA	NA	NA	NA	0.000	NA	NA	NA	0.006	NA	0.000	0.000	NA	0.000	NA	NA	0.000	0.000	NA	0.000	0.000	0.000	0.000	0.000	0.000	NA
rs2238540	T	0.141	NA	NA	NA	NA	NA	0.238	NA	NA	0.114	0.320	NA	0.258	0.066	NA	0.233	NA	NA	0.243	0.106	NA	0.242	0.162	0.082	0.252	0.270	0.108	NA
rs189460104	A	0.010	NA	NA	NA	NA	NA	0.008	NA	NA	NA	0.000	NA	0.000	0.000	NA	0.000	NA	NA	0.005	0.000	NA	0.005	0.000	0.000	0.000	0.022	0.000	NA
rs118187381	A	0.005	NA	NA	NA	NA	NA	0.000	NA	NA	0.000	0.000	NA	0.000	0.025	NA	0.000	NA	NA	0.000	0.000	NA	0.000	0.005	0.016	0.000	0.000	0.000	NA
rs183979701	G	0.000	NA	NA	NA	NA	NA	0.008	NA	NA	NA	0.000	NA	0.000	0.000	NA	0.000	0.5	NA	0.000	0.048	NA	0.005	0.005	0.000	0.000	0.000	0.000	NA
rs10593023	C	0.333	NA	NA	NA	NA	NA	0.262	NA	NA	NA	0.361	NA	0.457	0.167	NA	0.398	0.5909	NA	0.462	0.202	NA	0.313	0.147	0.198	0.369	0.376	0.220	NA
rs561149009	A	0.005	NA	NA	NA	NA	NA	0.000	NA	NA	NA	0.000	NA	0.000	0.000	NA	0.000	NA	NA	0.000	0.011	NA	0.000	0.000	0.000	0.000	0.000	0.000	NA
rs543884178	T	0.000	NA	NA	NA	NA	NA	0.000	NA	NA	NA	0.000	NA	0.000	0.000	NA	0.000	NA	NA	0.000	0.000	NA	0.000	0.000	0.000	0.000	0.000	0.000	NA
rs544074414	A	0.005	NA	NA	NA	NA	NA	0.000	NA	NA	NA	0.000	NA	0.000	0.000	NA	0.000	NA	NA	0.000	0.000	NA	0.000	0.000	0.000	0.000	0.000	0.000	NA
rs551225501	G	0.021	NA	NA	NA	NA	NA	0.025	NA	NA	NA	0.000	NA	0.000	0.000	NA	0.000	NA	NA	0.000	0.000	NA	0.045	0.000	0.000	0.000	0.031	0.000	NA
rs28915407	T	0.016	NA	NA	NA	NA	NA	0.000	NA	NA	NA	0.000	NA	0.000	0.040	NA	0.000	NA	NA	0.000	0.000	NA	0.000	0.025	0.066	0.000	0.000	0.047	NA

SNP	Allele	ACB	Afro	Aimaras	Ancash	Arequipa	Ashaninkas	ASW	Awajun	Ayacucho	Bambui	BEB	Candoshi	CDX	CEU	Chachapoyas	CHB	Chileans	Chopccas	CHS	CLM	Cusco	ESN	FIN	GBR	GIH	GWD	IBS	Iquitos
rs28992486	G	0.177	NA	NA	NA	NA	NA	0.098	NA	NA	NA	0.000	NA	0.000	0.000	NA	0.000	NA	NA	0.000	0.021	NA	0.147	0.000	0.000	0.000	0.040	0.005	NA
rs375283966	C	0.000	NA	NA	NA	NA	NA	0.000	NA	NA	NA	0.000	NA	0.000	0.000	NA	0.000	NA	NA	0.000	0.000	NA	0.000	0.000	0.000	0.000	0.004	0.000	NA
rs2283572	T	0.333	NA	NA	NA	NA	NA	0.262	NA	NA	NA	0.361	NA	0.457	0.167	NA	0.398	0.65	NA	0.462	0.213	NA	0.313	0.147	0.198	0.369	0.381	0.220	NA
rs12609063	A	0.141	NA	NA	NA	NA	NA	0.238	NA	NA	NA	0.320	NA	0.258	0.066	NA	0.233	NA	NA	0.243	0.106	NA	0.242	0.162	0.082	0.252	0.270	0.108	NA
rs528163900	A	0.000	NA	NA	NA	NA	NA	0.000	NA	NA	NA	0.000	NA	0.000	0.000	NA	0.000	NA	NA	0.000	0.000	NA	0.000	0.000	0.000	0.000	0.000	0.000	NA
rs188930171	C	0.000	NA	NA	NA	NA	NA	0.000	NA	NA	NA	0.000	NA	0.000	0.000	NA	0.000	NA	NA	0.005	0.000	NA	0.000	0.000	0.000	0.000	0.000	0.000	NA
rs149894645	C	0.000	NA	NA	NA	NA	NA	0.000	NA	NA	NA	0.000	NA	0.000	0.000	NA	0.000	NA	NA	0.000	0.000	NA	0.000	0.000	0.000	0.000	0.000	0.000	NA
rs558467385	A	0.000	NA	NA	NA	NA	NA	0.000	NA	NA	NA	0.000	NA	0.000	0.000	NA	0.000	NA	NA	0.000	0.000	NA	0.000	0.000	0.000	0.000	0.004	0.000	NA
rs542497048	T	0.000	NA	NA	NA	NA	NA	0.000	NA	NA	NA	0.000	NA	0.000	0.000	NA	0.000	NA	NA	0.000	0.000	NA	0.000	0.000	0.000	0.005	0.000	0.000	NA
rs547801807	C	0.042	NA	NA	NA	NA	NA	0.041	NA	NA	NA	0.000	NA	0.000	0.000	NA	0.000	NA	NA	0.000	0.000	NA	0.071	0.000	0.000	0.000	0.004	0.000	NA
rs371927933	T	0.000	NA	NA	NA	NA	NA	0.000	NA	NA	NA	0.000	NA	0.000	0.000	NA	0.000	NA	NA	0.000	0.000	NA	0.000	0.000	0.000	0.000	0.000	0.000	NA
rs556218809	A	0.000	NA	NA	NA	NA	NA	0.000	NA	NA	NA	0.000	NA	0.000	0.000	NA	0.005	NA	NA	0.000	0.000	NA	0.000	0.000	0.000	0.000	0.000	0.000	NA
rs1138150	T	0.287	NA	NA	NA	NA	NA	0.402	NA	NA	NA	0.564	NA	0.489	0.727	NA	0.534	0.1786	NA	0.471	0.532	NA	0.258	0.748	0.687	0.471	0.332	0.631	NA
rs28921974	C	0.068	NA	NA	NA	NA	NA	0.041	NA	NA	0.109	0.151	NA	0.022	0.030	NA	0.039	0.5	NA	0.043	0.027	NA	0.071	0.015	0.066	0.078	0.031	0.056	NA
rs568364520	A	0.000	NA	NA	NA	NA	NA	0.000	NA	NA	NA	0.000	NA	0.000	0.000	NA	0.005	NA	NA	0.000	0.000	NA	0.000	0.000	0.000	0.000	0.000	0.000	NA
rs2283573	T	0.141	NA	NA	NA	NA	NA	0.238	NA	NA	NA	0.320	NA	0.258	0.066	NA	0.233	NA	NA	0.243	0.106	NA	0.242	0.162	0.082	0.252	0.270	0.108	NA
rs562293196	A	0.000	NA	NA	NA	NA	NA	0.000	NA	NA	NA	0.000	NA	0.000	0.000	NA	0.000	NA	NA	0.000	0.000	NA	0.000	0.000	0.000	0.000	0.000	0.000	NA
rs11458913	C	0.167	NA	NA	NA	NA	NA	0.205	NA	NA	NA	0.250	NA	0.237	0.662	NA	0.301	0.1786	NA	0.229	0.431	NA	0.071	0.586	0.610	0.223	0.102	0.523	NA
rs114359237	T	0.021	NA	NA	NA	NA	NA	0.000	NA	NA	NA	0.000	NA	0.000	0.000	NA	0.000	NA	NA	0.000	0.000	NA	0.010	0.000	0.000	0.000	0.004	0.000	NA
rs28989774	A	0.120	NA	NA	NA	NA	NA	0.074	NA	NA	NA	0.000	NA	0.000	0.000	NA	0.000	NA	NA	0.000	0.021	NA	0.111	0.000	0.000	0.000	0.035	0.005	NA
rs189315508	A	0.000	NA	NA	NA	NA	NA	0.000	NA	NA	NA	0.000	NA	0.000	0.000	NA	0.000	NA	NA	0.000	0.000	NA	0.000	0.000	0.005	0.000	0.000	0.000	NA
rs138409636	T	0.000	NA	NA	NA	NA	NA	0.000	NA	NA	NA	0.000	NA	0.000	0.000	NA	0.000	NA	NA	0.000	0.000	NA	0.000	0.000	0.000	0.000	0.000	0.000	NA
rs375925694	T	0.000	NA	NA	NA	NA	NA	0.000	NA	NA	NA	0.006	NA	0.032	0.000	NA	0.015	NA	NA	0.024	0.000	NA	0.000	0.000	0.000	0.000	0.000	0.000	NA
rs567062140	A	0.000	NA	NA	NA	NA	NA	0.000	NA	NA	NA	0.000	NA	0.000	0.000	NA	0.000	NA	NA	0.000	0.000	NA	0.000	0.000	0.000	0.000	0.000	0.000	NA
rs574441118	A	0.000	NA	NA	NA	NA	NA	0.000	NA	NA	NA	0.000	NA	0.000	0.000	NA	0.000	NA	NA	0.000	0.000	NA	0.000	0.000	0.000	0.000	0.000	0.000	NA
rs1049676	T	0.047	NA	NA	NA	NA	NA	0.057	NA	NA	0.006	0.070	NA	0.000	0.056	NA	0.000	0.5	NA	0.000	0.043	NA	0.025	0.040	0.049	0.044	0.013	0.037	NA
rs183656586	A	0.000	NA	NA	NA	NA	NA	0.000	NA	NA	NA	0.000	NA	0.000	0.000	NA	0.005	NA	NA	0.010	0.000	NA	0.000	0.000	0.000	0.000	0.000	0.000	NA
rs574480082	G	0.000	NA	NA	NA	NA	NA	0.000	NA	NA	NA	0.000	NA	0.000	0.000	NA	0.000	NA	NA	0.000	0.000	NA	0.000	0.000	0.000	0.000	0.009	0.000	NA
rs565755933	T	0.000	NA	NA	NA	NA	NA	0.000	NA	NA	NA	0.000	NA	0.000	0.000	NA	0.000	NA	NA	0.000	0.000	NA	0.000	0.005	0.005	0.000	0.000	0.009	NA
rs554979181	A	0.000	NA	NA	NA	NA	NA	0.000	NA	NA	NA	0.000	NA	0.000	0.000	NA	0.000	NA	NA	0.000	0.000	NA	0.000	0.000	0.000	0.005	0.000	0.000	NA
rs146784114	A	NA	NA	NA	NA	NA	NA	NA	NA	NA	NA	NA	NA	NA	NA	NA	NA	NA	NA	NA	NA	NA	NA	NA	NA	NA	NA	NA	NA
rs564593406	A	0.005	NA	NA	NA	NA	NA	0.000	NA	NA	NA	0.000	NA	0.000	0.000	NA	0.000	NA	NA	0.000	0.000	NA	0.000	0.000	0.000	0.000	0.000	0.000	NA
rs535528596	T	0.000	NA	NA	NA	NA	NA	0.000	NA	NA	NA	0.000	NA	0.000	0.000	NA	0.000	NA	NA	0.000	0.000	NA	0.000	0.000	0.000	0.000	0.000	0.000	NA
rs185996213	C	0.016	NA	NA	NA	NA	NA	0.000	NA	NA	NA	0.017	NA	0.000	0.000	NA	0.000	NA	NA	0.000	0.005	NA	0.005	0.000	0.000	0.034	0.013	0.000	NA
rs185382187	A	0.000	NA	NA	NA	NA	NA	0.000	NA	NA	NA	0.000	NA	0.000	0.000	NA	0.000	NA	NA	0.000	0.000	NA	0.000	0.000	0.005	0.000	0.000	0.000	NA
rs2072308	C	0.151	NA	NA	NA	NA	NA	0.254	NA	NA	NA	0.314	NA	0.199	0.066	NA	0.155	NA	NA	0.195	0.106	NA	0.253	0.162	0.077	0.243	0.279	0.108	NA
rs141043764	A	0.010	NA	NA	NA	NA	NA	0.008	NA	NA	NA	0.000	NA	0.000	0.005	NA	0.000	NA	NA	0.000	0.005	NA	0.005	0.000	0.000	0.000	0.000	0.000	NA
rs185250119	A	0.000	NA	NA	NA	NA	NA	0.000	NA	NA	NA	0.000	NA	0.016	0.000	NA	0.010	NA	NA	0.010	0.000	NA	0.000	0.000	0.000	0.000	0.000	0.000	NA
rs147511542	C	0.000	NA	NA	NA	NA	NA	0.000	NA	NA	NA	0.000	NA	0.000	0.000	NA	0.005	NA	NA	0.000	0.000	NA	0.000	0.000	0.000	0.000	0.000	0.000	NA
rs573330257	G	0.000	NA	NA	NA	NA	NA	0.000	NA	NA	NA	0.000	NA	0.000	0.000	NA	0.000	NA	NA	0.000	0.000	NA	0.005	0.000	0.000	0.000	0.000	0.000	NA
rs542924300	T	0.000	NA	NA	NA	NA	NA	0.000	NA	NA	NA	0.000	NA	0.000	0.000	NA	0.000	NA	NA	0.000	0.000	NA	0.000	0.000	0.000	0.000	0.000	0.000	NA
rs543561666	A	0.016	NA	NA	NA	NA	NA	0.000	NA	NA	NA	0.000	NA	0.000	0.000	NA	0.000	NA	NA	0.000	0.000	NA	0.000	0.000	0.000	0.000	0.000	0.000	NA
rs8110980	T	0.333	0.362	0.433	0.431	0.273	0.271	0.262	0.348	0.427	0.345	0.361	0.219	0.457	0.167	0.263	0.398	0.65	0.412	0.462	0.202	0.429	0.313	0.147	0.198	0.369	0.376	0.220	0.135
rs200045153	G	0.010	NA	NA	NA	NA	NA	0.008	NA	NA	NA	0.006	NA	0.000	0.061	NA	0.000	NA	NA	0.000	0.027	NA	0.000	0.066	0.077	0.000	0.000	0.028	NA
rs10422916	T	0.068	NA	NA	NA	NA	NA	0.115	NA	NA	NA	0.157	NA	0.231	0.616	NA	0.301	0	NA	0.229	0.378	NA	0.015	0.546	0.560	0.141	0.044	0.491	NA

SNP	Allele	ACB	Afro	Aimaras	Ancash	Arequipa	Ashaninkas	ASW	Awajun	Ayacucho	Bambui	BEB	Candoshi	CDX	CEU	Chachapoyas	CHB	Chileans	Chopccas	CHS	CLM	Cusco	ESN	FIN	GBR	GIH	GWD	IBS	Iquitos
rs568226979	T	0.005	NA	NA	NA	NA	NA	0.000	NA	NA	NA	0.000	NA	0.000	0.000	NA	0.000	NA	NA	0.000	0.000	NA	0.000	0.000	0.000	0.000	0.000	0.000	NA
rs550149267	T	0.005	NA	NA	NA	NA	NA	0.000	NA	NA	NA	0.000	NA	0.000	0.000	NA	0.000	NA	NA	0.000	0.000	NA	0.000	0.000	0.000	0.000	0.000	0.000	NA
rs182848936	T	0.000	NA	NA	NA	NA	NA	0.008	NA	NA	NA	0.000	NA	0.000	0.000	NA	0.000	NA	NA	0.000	0.000	NA	0.000	0.000	0.000	0.000	0.000	0.000	NA
rs535528658	T	0.000	NA	NA	NA	NA	NA	0.008	NA	NA	NA	0.000	NA	0.000	0.000	NA	0.000	NA	NA	0.000	0.000	NA	0.005	0.000	0.000	0.000	0.000	0.000	NA
rs537007790	T	0.000	NA	NA	NA	NA	NA	0.000	NA	NA	NA	0.000	NA	0.000	0.000	NA	0.000	NA	NA	0.000	0.000	NA	0.000	0.005	0.000	0.000	0.000	0.000	NA
rs571095325	A	0.010	NA	NA	NA	NA	NA	0.016	NA	NA	NA	0.000	NA	0.000	0.000	NA	0.000	NA	NA	0.000	0.000	NA	0.025	0.000	0.000	0.000	0.009	0.000	NA
rs548874736	T	0.000	NA	NA	NA	NA	NA	0.008	NA	NA	NA	0.000	NA	0.000	0.000	NA	0.000	NA	NA	0.000	0.000	NA	0.015	0.000	0.000	0.000	0.031	0.000	NA
rs546527955	T	0.000	NA	NA	NA	NA	NA	0.000	NA	NA	NA	0.000	NA	0.000	0.000	NA	0.000	NA	NA	0.000	0.000	NA	0.000	0.000	0.000	0.000	0.004	0.000	NA
rs533122473	T	0.000	NA	NA	NA	NA	NA	0.000	NA	NA	NA	0.000	NA	0.000	0.000	NA	0.000	NA	NA	0.000	0.000	NA	0.000	0.000	0.000	0.000	0.004	0.000	NA
rs28992481	C	0.026	NA	NA	NA	NA	NA	0.016	NA	NA	NA	0.000	NA	0.000	0.000	NA	0.000	NA	NA	0.000	0.000	NA	0.000	0.000	0.000	0.000	0.000	0.000	NA
rs547511336	A	0.000	NA	NA	NA	NA	NA	0.000	NA	NA	NA	0.000	NA	0.000	0.000	NA	0.000	NA	NA	0.000	0.000	NA	0.000	0.000	0.000	0.000	0.000	0.000	NA
rs117305685	T	0.000	NA	NA	NA	NA	NA	0.000	NA	NA	0.000	0.000	NA	0.000	0.005	NA	0.000	NA	NA	0.000	0.000	NA	0.000	0.010	0.000	0.000	0.000	0.009	NA
rs28921987	A	0.000	NA	NA	NA	NA	NA	0.000	NA	NA	NA	0.000	NA	0.000	0.005	NA	0.000	NA	NA	0.000	0.000	NA	0.000	0.000	0.000	0.000	0.000	0.009	NA
rs60851035	A	0.026	NA	NA	NA	NA	NA	0.041	NA	NA	NA	0.000	NA	0.000	0.000	NA	0.000	NA	NA	0.000	0.000	NA	0.051	0.000	0.000	0.000	0.044	0.000	NA
rs570887990	T	0.000	NA	NA	NA	NA	NA	0.000	NA	NA	NA	0.000	NA	0.000	0.000	NA	0.000	NA	NA	0.000	0.000	NA	0.000	0.000	0.000	0.000	0.000	0.000	NA
rs556084263	T	0.000	NA	NA	NA	NA	NA	0.000	NA	NA	NA	0.000	NA	0.000	0.000	NA	0.000	NA	NA	0.000	0.000	NA	0.000	0.000	0.000	0.000	0.000	0.000	NA
rs142283391	T	0.000	NA	NA	NA	NA	NA	0.000	NA	NA	NA	0.000	NA	0.000	0.000	NA	0.000	NA	NA	0.000	0.000	NA	0.005	0.000	0.000	0.000	0.000	0.000	NA
rs375240640	A	0.026	NA	NA	NA	NA	NA	0.041	NA	NA	NA	0.070	NA	0.032	0.066	NA	0.024	0.625	NA	0.024	0.154	NA	0.040	0.066	0.055	0.112	0.035	0.079	NA
rs114264435	T	0.000	NA	NA	NA	NA	NA	0.016	NA	NA	NA	0.000	NA	0.000	0.000	NA	0.000	NA	NA	0.000	0.005	NA	0.020	0.000	0.000	0.005	0.013	0.000	NA
rs553170407	C	0.000	NA	NA	NA	NA	NA	0.000	NA	NA	NA	0.000	NA	0.000	0.000	NA	0.000	NA	NA	0.000	0.000	NA	0.000	0.000	0.000	0.015	0.000	0.000	NA
rs541302606	A	0.000	NA	NA	NA	NA	NA	0.000	NA	NA	NA	0.000	NA	0.000	0.000	NA	0.000	NA	NA	0.000	0.000	NA	0.000	0.000	0.000	0.000	0.000	0.000	NA
rs529379861	A	0.000	NA	NA	NA	NA	NA	0.000	NA	NA	NA	0.000	NA	0.000	0.000	NA	0.000	NA	NA	0.000	0.000	NA	0.000	0.000	0.000	0.000	0.000	0.005	NA
rs112152563	T	NA	NA	NA	NA	NA	NA	NA	NA	NA	NA	NA	NA	NA	NA	NA	NA	NA	NA	NA	NA	NA	NA	NA	NA	NA	NA	NA	NA
rs557135170	A	0.000	NA	NA	NA	NA	NA	0.000	NA	NA	NA	0.000	NA	0.000	0.000	NA	0.000	NA	NA	0.000	0.005	NA	0.000	0.000	0.000	0.000	0.000	0.000	NA
rs557199346	A	0.000	NA	NA	NA	NA	NA	0.000	NA	NA	NA	0.000	NA	0.000	0.000	NA	0.000	NA	NA	0.000	0.000	NA	0.000	0.000	0.000	0.000	0.000	0.000	NA
rs14173	G	0.010	NA	NA	NA	NA	NA	0.008	NA	NA	NA	0.000	NA	0.005	0.000	NA	0.000	NA	NA	0.000	0.005	NA	0.000	0.000	0.000	0.000	0.000	0.000	NA
rs531691645	A	0.000	NA	NA	NA	NA	NA	0.000	NA	NA	NA	0.000	NA	0.000	0.005	NA	0.000	NA	NA	0.000	0.000	NA	0.000	0.010	0.000	0.000	0.000	0.000	NA
rs376756373	A	0.000	NA	NA	NA	NA	NA	0.000	NA	NA	NA	0.000	NA	0.000	0.000	NA	0.000	NA	NA	0.000	0.000	NA	0.000	0.000	0.000	0.000	0.000	0.000	NA
rs7253615	G	0.167	NA	NA	NA	NA	NA	0.205	NA	NA	NA	0.244	NA	0.231	0.662	NA	0.301	0.1786	NA	0.229	0.426	NA	0.066	0.586	0.610	0.218	0.102	0.523	NA
rs566889863	C	0.000	NA	NA	NA	NA	NA	0.000	NA	NA	NA	0.000	NA	0.005	0.000	NA	0.000	NA	NA	0.000	0.000	NA	0.000	0.000	0.000	0.000	0.000	0.000	NA
rs369996586	C	0.000	NA	NA	NA	NA	NA	0.000	NA	NA	NA	0.000	NA	0.000	0.000	NA	0.000	NA	NA	0.000	0.000	NA	0.000	0.000	0.000	0.019	0.000	0.000	NA
rs200704727	CA	0.141	NA	NA	NA	NA	NA	0.238	NA	NA	NA	0.320	NA	0.258	0.066	NA	0.233	NA	NA	0.243	0.106	NA	0.242	0.162	0.082	0.252	0.270	0.108	NA
rs539709135	G	0.224	NA	NA	NA	NA	NA	0.205	NA	NA	NA	0.384	NA	0.043	0.071	NA	0.005	NA	NA	0.048	0.149	NA	0.369	0.040	0.022	0.209	0.460	0.051	NA
rs28921981	A	0.073	NA	NA	NA	NA	NA	0.115	NA	NA	NA	0.140	NA	0.075	0.035	NA	0.053	NA	NA	0.043	0.021	NA	0.111	0.035	0.022	0.165	0.204	0.033	NA
rs7252521	T	0.010	NA	NA	NA	NA	NA	0.000	NA	NA	NA	0.000	NA	0.000	0.000	NA	0.000	NA	NA	0.000	0.000	NA	0.000	0.000	0.000	0.000	0.000	0.000	NA
rs562167455	G	0.000	NA	NA	NA	NA	NA	0.000	NA	NA	NA	0.000	NA	0.000	0.000	NA	0.000	NA	NA	0.000	0.000	NA	0.000	0.000	0.000	0.005	0.000	0.000	NA
rs539126301	A	0.000	NA	NA	NA	NA	NA	0.000	NA	NA	NA	0.000	NA	0.000	0.000	NA	0.000	NA	NA	0.000	0.000	NA	0.000	0.000	0.000	0.000	0.000	0.000	NA
rs570527862	G	0.000	NA	NA	NA	NA	NA	0.000	NA	NA	NA	0.000	NA	0.000	0.000	NA	0.000	NA	NA	0.000	0.000	NA	0.005	0.000	0.000	0.000	0.000	0.000	NA
rs577273577	T	0.000	NA	NA	NA	NA	NA	0.000	NA	NA	NA	0.000	NA	0.000	0.000	NA	0.000	NA	NA	0.000	0.000	NA	0.000	0.000	0.000	0.000	0.000	0.000	NA
rs187186896	G	0.000	NA	NA	NA	NA	NA	0.000	NA	NA	NA	0.000	NA	0.000	0.000	NA	0.000	NA	NA	0.000	0.000	NA	0.000	0.000	0.000	0.000	0.000	0.000	NA
rs11551906	G	0.005	NA	NA	NA	NA	NA	0.000	NA	NA	NA	0.000	NA	0.000	0.030	NA	0.000	NA	NA	0.000	0.016	NA	0.000	0.010	0.033	0.000	0.000	0.014	NA
rs576653835	A	0.000	NA	NA	NA	NA	NA	0.000	NA	NA	NA	0.000	NA	0.000	0.000	NA	0.000	NA	NA	0.000	0.000	NA	0.000	0.000	0.000	0.000	0.000	0.000	NA
rs141694906	A	0.000	NA	NA	NA	NA	NA	0.000	NA	NA	NA	0.000	NA	0.000	0.000	NA	0.000	NA	NA	0.000	0.000	NA	0.000	0.000	0.000	0.000	0.000	0.000	NA
rs140396239	A	0.000	NA	NA	NA	NA	NA	0.000	NA	NA	NA	0.000	NA	0.000	0.000	NA	0.000	NA	NA	0.000	0.000	NA	0.000	0.000	0.000	0.000	0.000	0.000	NA
rs142446858	T	NA	NA	NA	NA	NA	NA	NA	NA	NA	NA	NA	NA	NA	NA	NA	NA	NA	NA	NA	NA	NA	NA	NA	NA	NA	NA	NA	NA

SNP	Allele	ACB	Afro	Aimaras	Ancash	Arequipa	Ashaninkas	ASW	Awajun	Ayacucho	Bambui	BEB	Candoshi	CDX	CEU	Chachapoyas	CHB	Chileans	Chopccas	CHS	CLM	Cusco	ESN	FIN	GBR	GIH	GWD	IBS	Iquitos
rs561158080	T	0.000	NA	NA	NA	NA	NA	0.000	NA	NA	NA	0.000	NA	0.000	0.000	NA	0.000	NA	NA	0.000	0.000	NA	0.005	0.000	0.000	0.000	0.000	0.000	NA
rs2238543	T	0.068	NA	NA	NA	NA	NA	0.115	NA	NA	NA	0.157	NA	0.231	0.606	NA	0.301	0.2	NA	0.219	0.383	NA	0.015	0.546	0.560	0.141	0.044	0.486	NA
rs554135380	T	0.000	NA	NA	NA	NA	NA	0.000	NA	NA	NA	0.000	NA	0.000	0.000	NA	0.005	NA	NA	0.000	0.000	NA	0.000	0.000	0.000	0.000	0.000	0.000	NA
rs543916508	A	0.000	NA	NA	NA	NA	NA	0.000	NA	NA	NA	0.000	NA	0.000	0.000	NA	0.000	NA	NA	0.000	0.000	NA	0.000	0.000	0.000	0.000	0.000	0.000	NA
rs551307834	T	0.000	NA	NA	NA	NA	NA	0.000	NA	NA	NA	0.000	NA	0.000	0.000	NA	0.000	NA	NA	0.000	0.000	NA	0.000	0.000	0.000	0.000	0.000	0.000	NA
rs146061693	A	0.010	NA	NA	NA	NA	NA	0.016	NA	NA	NA	0.000	NA	0.000	0.000	NA	0.000	NA	NA	0.000	0.000	NA	0.035	0.000	0.000	0.000	0.004	0.000	NA
rs559328258	T	0.000	NA	NA	NA	NA	NA	0.000	NA	NA	NA	0.000	NA	0.000	0.000	NA	0.000	NA	NA	0.000	0.000	NA	0.000	0.000	0.000	0.000	0.000	0.000	NA
rs545139311	A	0.000	NA	NA	NA	NA	NA	0.000	NA	NA	NA	0.000	NA	0.000	0.000	NA	0.000	NA	NA	0.000	0.000	NA	0.000	0.000	0.000	0.000	0.004	0.000	NA
rs572647656	T	0.000	NA	NA	NA	NA	NA	0.000	NA	NA	NA	0.000	NA	0.000	0.000	NA	0.000	NA	NA	0.000	0.000	NA	0.000	0.000	0.000	0.000	0.004	0.000	NA
rs372445442	A	0.000	NA	NA	NA	NA	NA	0.000	NA	NA	NA	0.000	NA	0.000	0.000	NA	0.000	NA	NA	0.000	0.000	NA	0.000	0.000	0.000	0.000	0.000	0.000	NA
rs190259313	A	0.000	NA	NA	NA	NA	NA	0.000	NA	NA	NA	0.000	NA	0.016	0.000	NA	0.005	NA	NA	0.014	0.000	NA	0.000	0.000	0.000	0.000	0.000	0.000	NA
rs561243171	T	0.120	NA	NA	NA	NA	NA	0.057	NA	NA	NA	0.000	NA	0.000	0.000	NA	0.005	NA	NA	0.000	0.000	NA	0.096	0.000	0.000	0.000	0.102	0.000	NA
rs138600800	C	0.000	NA	NA	NA	NA	NA	0.000	NA	NA	0.006	0.006	NA	0.000	0.015	NA	0.000	0.5	NA	0.000	0.016	NA	0.000	0.005	0.005	0.000	0.000	0.023	NA
rs28921977	G	0.021	NA	NA	NA	NA	NA	0.025	NA	NA	NA	0.000	NA	0.000	0.035	NA	0.005	NA	NA	0.000	0.053	NA	0.051	0.040	0.044	0.005	0.022	0.061	NA
rs571072379	T	0.000	NA	NA	NA	NA	NA	0.000	NA	NA	NA	0.000	NA	0.000	0.000	NA	0.000	NA	NA	0.000	0.000	NA	0.000	0.000	0.000	0.000	0.000	0.000	NA
rs375819042	T	0.000	NA	NA	NA	NA	NA	0.000	NA	NA	NA	0.000	NA	0.016	0.000	NA	0.005	NA	NA	0.000	0.000	NA	0.000	0.000	0.000	0.000	0.000	0.000	NA
rs41276870	T	0.000	NA	NA	NA	NA	NA	0.000	NA	NA	NA	0.000	NA	0.000	0.005	NA	0.000	NA	NA	0.000	0.000	NA	0.000	0.010	0.000	0.000	0.000	0.000	NA
rs567156454	C	0.000	NA	NA	NA	NA	NA	0.000	NA	NA	NA	0.000	NA	0.005	0.000	NA	0.000	NA	NA	0.000	0.000	NA	0.000	0.000	0.000	0.000	0.000	0.000	NA
rs539842435	T	0.005	NA	NA	NA	NA	NA	0.000	NA	NA	NA	0.000	NA	0.000	0.005	NA	0.000	NA	NA	0.000	0.011	NA	0.000	0.000	0.000	0.000	0.000	0.009	NA
rs529739382	G	0.000	NA	NA	NA	NA	NA	0.008	NA	NA	NA	0.000	NA	0.000	0.000	NA	0.000	NA	NA	0.000	0.000	NA	0.010	0.000	0.000	0.000	0.000	0.000	NA
rs2041192	A	0.151	0.085	0.188	0.300	0.283	0.214	0.197	0.087	0.243	NA	0.244	0.125	0.231	0.662	0.238	0.301	0.1786	0.118	0.229	0.426	0.306	0.066	0.586	0.610	0.218	0.097	0.523	0.269
rs565127256	A	0.000	NA	NA	NA	NA	NA	0.000	NA	NA	NA	0.000	NA	0.000	0.000	NA	0.000	NA	NA	0.000	0.000	NA	0.000	0.000	0.000	0.005	0.000	0.000	NA
rs561885264	T	0.000	NA	NA	NA	NA	NA	0.000	NA	NA	NA	0.000	NA	0.000	0.000	NA	0.000	NA	NA	0.000	0.000	NA	0.000	0.000	0.000	0.000	0.000	0.005	NA
rs111866858	A	0.068	NA	NA	NA	NA	NA	0.049	NA	NA	NA	0.000	NA	0.000	0.000	NA	0.000	NA	NA	0.000	0.005	NA	0.091	0.000	0.000	0.000	0.159	0.000	NA
rs141476449	T	0.146	NA	NA	NA	NA	NA	0.238	NA	NA	NA	0.320	NA	0.204	0.066	NA	0.155	NA	NA	0.195	0.112	NA	0.242	0.167	0.082	0.243	0.274	0.108	NA
rs144233497	A	0.000	NA	NA	NA	NA	NA	0.000	NA	NA	NA	0.000	NA	0.000	0.000	NA	0.000	NA	NA	0.000	0.000	NA	0.000	0.000	0.000	0.000	0.000	0.000	NA
rs183270957	T	0.000	NA	NA	NA	NA	NA	0.000	NA	NA	NA	0.000	NA	0.000	0.000	NA	0.000	NA	NA	0.000	0.000	NA	0.000	0.000	0.000	0.000	0.000	0.000	NA
rs554359587	T	0.000	NA	NA	NA	NA	NA	0.000	NA	NA	NA	0.000	NA	0.000	0.000	NA	0.000	NA	NA	0.000	0.000	NA	0.000	0.000	0.000	0.000	0.000	0.000	NA
rs55713331	T	0.000	NA	NA	NA	NA	NA	0.000	NA	NA	NA	0.029	NA	0.000	0.000	NA	0.000	NA	NA	0.000	0.000	NA	0.005	0.000	0.000	0.039	0.000	0.000	NA
rs367873477	C	0.000	NA	NA	NA	NA	NA	0.000	NA	NA	NA	0.029	NA	0.000	0.000	NA	0.000	NA	NA	0.000	0.000	NA	0.000	0.000	0.000	0.039	0.000	0.000	NA
rs576495399	T	0.000	NA	NA	NA	NA	NA	0.000	NA	NA	NA	0.000	NA	0.000	0.000	NA	0.000	NA	NA	0.000	0.000	NA	0.000	0.000	0.000	0.000	0.000	0.005	NA
rs564880752	A	0.000	NA	NA	NA	NA	NA	0.000	NA	NA	NA	0.006	NA	0.000	0.000	NA	0.000	NA	NA	0.000	0.000	NA	0.000	0.000	0.000	0.000	0.000	0.000	NA
rs549998110	T	0.000	NA	NA	NA	NA	NA	0.000	NA	NA	NA	0.000	NA	0.000	0.000	NA	0.005	NA	NA	0.000	0.000	NA	0.000	0.000	0.000	0.000	0.000	0.000	NA
rs370592787	T	0.010	NA	NA	NA	NA	NA	0.008	NA	NA	NA	0.000	NA	0.000	0.000	NA	0.000	NA	NA	0.000	0.000	NA	0.005	0.000	0.000	0.000	0.022	0.000	NA
rs12609912	T	0.141	NA	NA	NA	NA	NA	0.238	NA	NA	0.109	0.320	NA	0.258	0.066	NA	0.233	NA	NA	0.243	0.106	NA	0.242	0.162	0.082	0.243	0.270	0.108	NA
rs138950962	A	0.000	NA	NA	NA	NA	NA	0.000	NA	NA	NA	0.000	NA	0.000	0.000	NA	0.000	NA	NA	0.000	0.000	NA	0.000	0.000	0.000	0.000	0.000	0.000	NA
rs28915406	G	0.000	NA	NA	NA	NA	NA	0.025	NA	NA	NA	0.000	NA	0.000	0.000	NA	0.000	NA	NA	0.000	0.000	NA	0.015	0.000	0.000	0.000	0.000	0.000	NA
rs560808777	A	0.010	NA	NA	NA	NA	NA	0.000	NA	NA	NA	0.000	NA	0.000	0.000	NA	0.000	NA	NA	0.000	0.000	NA	0.000	0.000	0.000	0.000	0.004	0.000	NA
rs553790377	A	0.000	NA	NA	NA	NA	NA	0.000	NA	NA	NA	0.006	NA	0.000	0.000	NA	0.000	NA	NA	0.000	0.000	NA	0.000	0.000	0.000	0.000	0.000	0.000	NA
rs576854854	T	0.000	NA	NA	NA	NA	NA	0.000	NA	NA	NA	0.000	NA	0.000	0.000	NA	0.000	NA	NA	0.000	0.000	NA	0.000	0.000	0.000	0.000	0.000	0.000	NA
rs558986305	A	0.005	NA	NA	NA	NA	NA	0.000	NA	NA	NA	0.000	NA	0.000	0.000	NA	0.000	NA	NA	0.000	0.000	NA	0.000	0.000	0.000	0.000	0.000	0.000	NA
rs185398568	A	0.000	NA	NA	NA	NA	NA	0.000	NA	NA	NA	0.000	NA	0.000	0.000	NA	0.005	NA	NA	0.000	0.000	NA	0.000	0.000	0.000	0.000	0.000	0.000	NA
rs555055894	A	0.000	NA	NA	NA	NA	NA	0.000	NA	NA	NA	0.000	NA	0.000	0.000	NA	0.000	NA	NA	0.000	0.000	NA	0.000	0.000	0.000	0.000	0.000	0.000	NA
rs181812598	T	0.000	NA	NA	NA	NA	NA	0.000	NA	NA	NA	0.000	NA	0.000	0.000	NA	0.000	NA	NA	0.000	0.000	NA	0.000	0.000	0.000	0.000	0.000	0.000	NA
rs534560674	G	0.000	NA	NA	NA	NA	NA	0.000	NA	NA	NA	0.000	NA	0.000	0.000	NA	0.000	NA	NA	0.000	0.005	NA	0.000	0.000	0.000	0.000	0.000	0.000	NA

SNP	Allele	ACB	Afro	Aimaras	Ancash	Arequipa	Ashaninkas	ASW	Awajun	Ayacucho	Bambui	BEB	Candoshi	CDX	CEU	Chachapoyas	CHB	Chileans	Chopccas	CHS	CLM	Cusco	ESN	FIN	GBR	GIH	GWD	IBS	Iquitos
rs2238541	T	0.005	NA	NA	NA	NA	NA	0.000	NA	NA	NA	0.000	NA	0.016	0.000	NA	0.058	0.5	NA	0.029	0.027	NA	0.000	0.010	0.000	0.000	0.000	0.000	NA
rs148868153	T	0.000	NA	NA	NA	NA	NA	0.000	NA	NA	NA	0.000	NA	0.000	0.005	NA	0.000	NA	NA	0.000	0.000	NA	0.000	0.000	0.000	0.000	0.000	0.000	NA
rs10422922	G	0.172	NA	NA	NA	NA	NA	0.139	NA	NA	NA	0.000	NA	0.022	0.040	NA	0.039	NA	NA	0.043	0.064	NA	0.202	0.040	0.044	0.010	0.181	0.065	NA
rs529166895	A	0.000	NA	NA	NA	NA	NA	0.000	NA	NA	NA	0.012	NA	0.000	0.000	NA	0.000	NA	NA	0.000	0.000	NA	0.000	0.000	0.000	0.000	0.000	0.000	NA
rs570767821	A	0.000	NA	NA	NA	NA	NA	0.000	NA	NA	NA	0.000	NA	0.000	0.000	NA	0.000	NA	NA	0.000	0.000	NA	0.000	0.000	0.000	0.000	0.000	0.000	NA
rs572997523	A	0.000	NA	NA	NA	NA	NA	0.000	NA	NA	NA	0.000	NA	0.000	0.000	NA	0.000	NA	NA	0.000	0.000	NA	0.000	0.000	0.000	0.000	0.000	0.000	NA
rs527567567	T	0.000	NA	NA	NA	NA	NA	0.000	NA	NA	NA	0.000	NA	0.000	0.000	NA	0.000	NA	NA	0.000	0.000	NA	0.000	0.000	0.000	0.000	0.000	0.000	NA
rs187875345	A	0.000	NA	NA	NA	NA	NA	0.000	NA	NA	NA	0.000	NA	0.000	0.000	NA	0.000	NA	NA	0.000	0.000	NA	0.000	0.000	0.000	0.000	0.000	0.000	NA
rs10412522	A	0.146	0.053	0.000	0.029	0.022	0.000	0.107	0.000	0.000	0.045	0.000	0.000	0.022	0.040	0.000	0.039	NA	0.000	0.043	0.064	0.000	0.152	0.040	0.044	0.010	0.133	0.065	0.000
rs199856669	G	0.000	NA	NA	NA	NA	NA	0.008	NA	NA	NA	0.000	NA	0.048	0.000	NA	0.029	0.5	NA	0.076	0.000	NA	0.000	0.000	0.000	0.000	0.000	0.000	NA
rs113431687	A	NA	NA	NA	NA	NA	NA	NA	NA	NA	NA	NA	NA	NA	NA	NA	NA	NA	NA	NA	NA	NA	NA	NA	NA	NA	NA	NA	NA
rs575490602	T	0.000	NA	NA	NA	NA	NA	0.000	NA	NA	NA	0.000	NA	0.000	0.000	NA	0.000	NA	NA	0.000	0.000	NA	0.000	0.000	0.005	0.000	0.000	0.000	NA
rs142683396	A	0.000	NA	NA	NA	NA	NA	0.000	NA	NA	NA	0.000	NA	0.000	0.000	NA	0.000	NA	NA	0.000	0.000	NA	0.000	0.000	0.011	0.000	0.000	0.000	NA
rs543921498	A	0.000	NA	NA	NA	NA	NA	0.008	NA	NA	NA	0.000	NA	0.000	0.000	NA	0.000	NA	NA	0.000	0.000	NA	0.000	0.000	0.000	0.005	0.000	0.000	NA
rs199564958	A	0.000	NA	NA	NA	NA	NA	0.000	NA	NA	NA	0.000	NA	0.000	0.000	NA	0.000	NA	NA	0.000	0.000	NA	0.000	0.005	0.000	0.000	0.000	0.000	NA
rs528839538	G	0.000	NA	NA	NA	NA	NA	0.000	NA	NA	NA	0.000	NA	0.000	0.000	NA	0.000	NA	NA	0.000	0.000	NA	0.000	0.000	0.005	0.010	0.000	0.000	NA
rs541648314	G	0.000	NA	NA	NA	NA	NA	0.000	NA	NA	NA	0.000	NA	0.000	0.000	NA	0.000	NA	NA	0.000	0.000	NA	0.000	0.000	0.000	0.000	0.000	0.000	NA
rs182434553	G	0.000	NA	NA	NA	NA	NA	0.000	NA	NA	NA	0.000	NA	0.000	0.000	NA	0.000	NA	NA	0.000	0.000	NA	0.000	0.000	0.000	0.000	0.000	0.000	NA
rs577301914	A	0.000	NA	NA	NA	NA	NA	0.000	NA	NA	NA	0.000	NA	0.000	0.000	NA	0.000	NA	NA	0.000	0.000	NA	0.000	0.000	0.000	0.000	0.004	0.000	NA
rs111517369	T	0.005	NA	NA	NA	NA	NA	0.016	NA	NA	NA	0.000	NA	0.000	0.000	NA	0.000	NA	NA	0.000	0.000	NA	0.010	0.000	0.000	0.000	0.009	0.000	NA
rs193000162	A	0.000	NA	NA	NA	NA	NA	0.000	NA	NA	NA	0.000	NA	0.000	0.000	NA	0.000	NA	NA	0.000	0.000	NA	0.000	0.000	0.000	0.000	0.000	0.000	NA
rs185020271	G	0.000	NA	NA	NA	NA	NA	0.000	NA	NA	NA	0.000	NA	0.000	0.000	NA	0.000	NA	NA	0.000	0.000	NA	0.000	0.000	0.000	0.000	0.000	0.000	NA
rs560734336	T	0.005	NA	NA	NA	NA	NA	0.000	NA	NA	NA	0.000	NA	0.000	0.000	NA	0.000	NA	NA	0.000	0.000	NA	0.000	0.000	0.000	0.000	0.000	0.000	NA
rs527804308	T	0.000	NA	NA	NA	NA	NA	0.000	NA	NA	NA	0.000	NA	0.000	0.010	NA	0.000	NA	NA	0.000	0.000	NA	0.000	0.000	0.005	0.010	0.000	0.000	NA
rs374000063	A	0.021	NA	NA	NA	NA	NA	0.008	NA	NA	NA	0.000	NA	0.000	0.000	NA	0.000	NA	NA	0.000	0.005	NA	0.000	0.000	0.000	0.000	0.027	0.000	NA
rs574157191	A	0.000	NA	NA	NA	NA	NA	0.000	NA	NA	NA	0.000	NA	0.005	0.000	NA	0.000	NA	NA	0.000	0.000	NA	0.000	0.000	0.000	0.000	0.000	0.000	NA
rs144058382	C	0.000	NA	NA	NA	NA	NA	0.000	NA	NA	NA	0.000	NA	0.000	0.000	NA	0.005	NA	NA	0.000	0.000	NA	0.000	0.000	0.000	0.000	0.000	0.000	NA
rs10422066	A	0.203	NA	NA	NA	NA	NA	0.262	NA	NA	NA	0.535	NA	0.500	0.818	NA	0.515	NA	NA	0.491	0.590	NA	0.081	0.788	0.808	0.452	0.168	0.687	NA
rs61729580	A	0.010	NA	NA	NA	NA	NA	0.000	NA	NA	NA	0.000	NA	0.000	0.010	NA	0.000	NA	NA	0.000	0.005	NA	0.000	0.005	0.016	0.000	0.000	0.005	NA
rs565778867	A	0.000	NA	NA	NA	NA	NA	0.000	NA	NA	NA	0.012	NA	0.000	0.000	NA	0.000	NA	NA	0.000	0.000	NA	0.000	0.000	0.000	0.000	0.000	0.000	NA
rs1803535	A	0.000	0.011	0.000	0.000	0.022	0.000	0.000	0.000	0.014	0.006	0.012	0.000	0.000	0.035	0.013	0.000	NA	0.000	0.000	0.016	0.000	0.000	0.020	0.027	0.000	0.000	0.061	0.000
rs539526884	A	0.000	NA	NA	NA	NA	NA	0.000	NA	NA	NA	0.000	NA	0.000	0.000	NA	0.005	NA	NA	0.000	0.000	NA	0.000	0.000	0.000	0.000	0.000	0.000	NA
rs536177717	A	0.000	NA	NA	NA	NA	NA	0.000	NA	NA	NA	0.012	NA	0.000	0.000	NA	0.000	NA	NA	0.000	0.000	NA	0.000	0.000	0.000	0.005	0.000	0.000	NA
rs530649173	C	0.000	NA	NA	NA	NA	NA	0.000	NA	NA	NA	0.000	NA	0.000	0.000	NA	0.000	NA	NA	0.000	0.000	NA	0.000	0.000	0.000	0.000	0.004	0.000	NA
rs181996046	T	0.000	NA	NA	NA	NA	NA	0.000	NA	NA	NA	0.000	NA	0.000	0.000	NA	0.000	NA	NA	0.000	0.005	NA	0.000	0.000	0.000	0.000	0.000	0.000	NA
rs546352239	T	0.000	NA	NA	NA	NA	NA	0.000	NA	NA	NA	0.000	NA	0.000	0.000	NA	0.000	NA	NA	0.000	0.000	NA	0.000	0.000	0.000	0.000	0.000	0.005	NA
rs531528464	A	0.000	NA	NA	NA	NA	NA	0.000	NA	NA	NA	0.000	NA	0.000	0.000	NA	0.000	NA	NA	0.000	0.000	NA	0.000	0.000	0.000	0.000	0.000	0.000	NA
rs537327096	T	0.000	NA	NA	NA	NA	NA	0.000	NA	NA	NA	0.000	NA	0.000	0.000	NA	0.000	NA	NA	0.000	0.000	NA	0.000	0.000	0.000	0.005	0.000	0.000	NA
rs28921978	T	0.005	NA	NA	NA	NA	NA	0.016	NA	NA	NA	0.070	NA	0.032	0.066	NA	0.024	0.7143	NA	0.024	0.160	NA	0.000	0.066	0.066	0.117	0.009	0.079	NA
rs541295754	T	0.000	NA	NA	NA	NA	NA	0.000	NA	NA	NA	0.000	NA	0.000	0.000	NA	0.000	NA	NA	0.000	0.000	NA	0.000	0.000	0.000	0.010	0.000	0.000	NA
rs201497117	A	0.000	NA	NA	NA	NA	NA	0.000	NA	NA	NA	0.000	NA	0.000	0.000	NA	0.005	NA	NA	0.000	0.000	NA	0.000	0.000	0.000	0.000	0.000	0.000	NA
rs564731463	A	0.000	NA	NA	NA	NA	NA	0.000	NA	NA	NA	0.000	NA	0.000	0.000	NA	0.000	NA	NA	0.000	0.000	NA	0.005	0.000	0.000	0.000	0.000	0.000	NA
rs542324120	T	0.000	NA	NA	NA	NA	NA	0.000	NA	NA	NA	0.000	NA	0.000	0.000	NA	0.000	NA	NA	0.000	0.000	NA	0.000	0.000	0.000	0.000	0.004	0.000	NA
rs112540819	A	0.000	NA	NA	NA	NA	NA	0.000	NA	NA	NA	0.000	NA	0.000	0.000	NA	0.000	NA	NA	0.000	0.000	NA	0.000	0.000	0.000	0.000	0.000	0.000	NA
rs145586063	T	0.000	NA	NA	NA	NA	NA	0.000	NA	NA	NA	0.000	NA	0.000	0.000	NA	0.000	NA	NA	0.000	0.000	NA	0.000	0.000	0.000	0.000	0.000	0.000	NA

SNP	Allele	ACB	Afro	Aimaras	Ancash	Arequipa	Ashaninkas	ASW	Awajun	Ayacucho	Bambui	BEB	Candoshi	CDX	CEU	Chachapoyas	CHB	Chileans	Chopccas	CHS	CLM	Cusco	ESN	FIN	GBR	GIH	GWD	IBS	Iquitos
rs556111109	T	0.000	NA	NA	NA	NA	NA	0.000	NA	NA	NA	0.000	NA	0.000	0.000	NA	0.000	NA	NA	0.000	0.000	NA	0.000	0.000	0.000	0.000	0.000	0.000	NA
rs544240790	A	0.000	NA	NA	NA	NA	NA	0.000	NA	NA	NA	0.000	NA	0.000	0.000	NA	0.000	NA	NA	0.000	0.000	NA	0.005	0.000	0.000	0.000	0.000	0.000	NA
rs527846123	A	0.000	NA	NA	NA	NA	NA	0.000	NA	NA	NA	0.000	NA	0.000	0.000	NA	0.000	NA	NA	0.000	0.000	NA	0.000	0.000	0.000	0.000	0.013	0.005	NA
rs200823690	AC	0.000	NA	NA	NA	NA	NA	0.008	NA	NA	NA	0.000	NA	0.048	0.000	NA	0.029	0.5	NA	0.076	0.000	NA	0.000	0.000	0.000	0.000	0.000	0.000	NA
rs547374902	A	0.000	NA	NA	NA	NA	NA	0.000	NA	NA	NA	0.000	NA	0.000	0.000	NA	0.000	NA	NA	0.000	0.000	NA	0.000	0.000	0.000	0.000	0.000	0.000	NA
rs534410335	T	0.000	NA	NA	NA	NA	NA	0.000	NA	NA	NA	0.000	NA	0.000	0.000	NA	0.000	NA	NA	0.000	0.000	NA	0.000	0.000	0.000	0.000	0.000	0.000	NA
rs11551900	G	0.042	NA	NA	NA	NA	NA	0.008	NA	NA	NA	0.000	NA	0.000	0.000	NA	0.000	NA	NA	0.000	0.000	NA	0.020	0.000	0.000	0.000	0.000	0.000	NA
rs188657384	T	0.005	NA	NA	NA	NA	NA	0.016	NA	NA	NA	0.000	NA	0.000	0.000	NA	0.000	NA	NA	0.000	0.000	NA	0.010	0.000	0.000	0.000	0.013	0.000	NA
rs7260603	A	0.177	NA	NA	NA	NA	NA	0.156	NA	NA	NA	0.070	NA	0.054	0.106	NA	0.063	0.7143	NA	0.067	0.223	NA	0.202	0.106	0.110	0.126	0.190	0.145	NA
rs537898048	A	0.005	NA	NA	NA	NA	NA	0.000	NA	NA	NA	0.000	NA	0.000	0.000	NA	0.000	NA	NA	0.000	0.000	NA	0.000	0.000	0.000	0.000	0.000	0.000	NA
rs575502711	A	0.000	NA	NA	NA	NA	NA	0.000	NA	NA	NA	0.017	NA	0.000	0.000	NA	0.000	NA	NA	0.000	0.000	NA	0.000	0.000	0.000	0.010	0.000	0.000	NA
rs532039913	A	0.000	NA	NA	NA	NA	NA	0.000	NA	NA	NA	0.000	NA	0.000	0.000	NA	0.000	NA	NA	0.000	0.000	NA	0.000	0.000	0.000	0.000	0.004	0.000	NA
rs558360796	C	0.000	NA	NA	NA	NA	NA	0.000	NA	NA	NA	0.000	NA	0.000	0.000	NA	0.000	NA	NA	0.000	0.000	NA	0.000	0.000	0.000	0.005	0.000	0.000	NA
rs547687363	G	0.000	NA	NA	NA	NA	NA	0.000	NA	NA	NA	0.000	NA	0.000	0.000	NA	0.000	NA	NA	0.000	0.000	NA	0.000	0.000	0.000	0.000	0.000	0.000	NA
rs144193084	T	0.000	NA	NA	NA	NA	NA	0.000	NA	NA	NA	0.000	NA	0.000	0.000	NA	0.000	NA	NA	0.000	0.000	NA	0.000	0.000	0.000	0.000	0.000	0.000	NA
rs28992473	G	0.005	NA	NA	NA	NA	NA	0.025	NA	NA	0.017	0.000	NA	0.000	0.000	NA	0.000	NA	NA	0.000	0.005	NA	0.025	0.000	0.000	0.000	0.013	0.000	NA
rs564064006	G	0.000	NA	NA	NA	NA	NA	0.000	NA	NA	NA	0.000	NA	0.000	0.000	NA	0.000	NA	NA	0.000	0.000	NA	0.000	0.000	0.000	0.000	0.000	0.000	NA
rs73918141	T	0.000	NA	NA	NA	NA	NA	0.008	NA	NA	NA	0.000	NA	0.000	0.000	NA	0.000	NA	NA	0.000	0.000	NA	0.015	0.000	0.000	0.000	0.035	0.000	NA
rs537163143	A	0.000	NA	NA	NA	NA	NA	0.016	NA	NA	NA	0.000	NA	0.000	0.000	NA	0.000	NA	NA	0.000	0.000	NA	0.005	0.000	0.000	0.000	0.000	0.000	NA
rs559463709	A	0.000	NA	NA	NA	NA	NA	0.000	NA	NA	NA	0.000	NA	0.000	0.000	NA	0.000	NA	NA	0.000	0.000	NA	0.000	0.000	0.000	0.010	0.000	0.000	NA
rs28921986	T	0.042	NA	NA	NA	NA	NA	0.041	NA	NA	NA	0.000	NA	0.000	0.000	NA	0.000	NA	NA	0.000	0.000	NA	0.071	0.005	0.000	0.000	0.009	0.000	NA
rs7252138	T	0.057	0.064	0.188	0.278	0.261	0.214	0.098	0.087	0.243	0.392	0.151	0.125	0.231	0.606	0.213	0.301	0.2	0.118	0.214	0.356	0.278	0.000	0.546	0.550	0.131	0.031	0.481	0.250
rs537290646	G	0.010	NA	NA	NA	NA	NA	0.000	NA	NA	NA	0.000	NA	0.000	0.000	NA	0.000	NA	NA	0.000	0.000	NA	0.000	0.000	0.000	0.000	0.000	0.000	NA
rs12609013	T	0.141	NA	NA	NA	NA	NA	0.238	NA	NA	NA	0.320	NA	0.258	0.066	NA	0.233	NA	NA	0.243	0.106	NA	0.242	0.162	0.082	0.257	0.270	0.108	NA
rs144824657	T	0.000	NA	NA	NA	NA	NA	0.000	NA	NA	NA	0.000	NA	0.000	0.005	NA	0.000	NA	NA	0.000	0.021	NA	0.000	0.000	0.011	0.000	0.000	0.019	NA
rs2283569	C	0.141	NA	NA	NA	NA	NA	0.238	NA	NA	NA	0.326	NA	0.258	0.071	NA	0.233	NA	NA	0.243	0.128	NA	0.242	0.162	0.093	0.262	0.270	0.112	NA
rs532689650	A	0.021	NA	NA	NA	NA	NA	0.008	NA	NA	NA	0.000	NA	0.000	0.000	NA	0.000	NA	NA	0.000	0.005	NA	0.000	0.000	0.000	0.000	0.027	0.000	NA
rs549863661	T	0.000	NA	NA	NA	NA	NA	0.000	NA	NA	NA	0.000	NA	0.000	0.000	NA	0.000	NA	NA	0.000	0.000	NA	0.000	0.000	0.000	0.010	0.000	0.000	NA
rs199862501	T	0.000	NA	NA	NA	NA	NA	0.000	NA	NA	NA	0.000	NA	0.000	0.000	NA	0.000	NA	NA	0.000	0.000	NA	0.000	0.005	0.000	0.000	0.000	0.005	NA
rs575727796	G	0.000	NA	NA	NA	NA	NA	0.008	NA	NA	NA	0.000	NA	0.000	0.000	NA	0.000	NA	NA	0.000	0.000	NA	0.005	0.000	0.000	0.000	0.004	0.000	NA
rs539913003	A	0.000	NA	NA	NA	NA	NA	0.000	NA	NA	NA	0.000	NA	0.005	0.000	NA	0.000	NA	NA	0.005	0.000	NA	0.000	0.000	0.000	0.000	0.000	0.000	NA
rs532703227	A	0.000	NA	NA	NA	NA	NA	0.000	NA	NA	NA	0.017	NA	0.000	0.000	NA	0.000	NA	NA	0.000	0.005	NA	0.000	0.000	0.000	0.034	0.000	0.000	NA
rs1138151	A	0.063	NA	NA	NA	NA	NA	0.098	NA	NA	NA	0.157	NA	0.231	0.606	NA	0.301	0.2	NA	0.229	0.378	NA	0.000	0.546	0.555	0.141	0.040	0.486	NA
rs537512067	T	0.000	NA	NA	NA	NA	NA	0.000	NA	NA	NA	0.000	NA	0.000	0.000	NA	0.000	NA	NA	0.000	0.000	NA	0.000	0.000	0.000	0.000	0.000	0.000	NA
rs569562559	A	0.000	NA	NA	NA	NA	NA	0.000	NA	NA	NA	0.000	NA	0.000	0.000	NA	0.000	NA	NA	0.000	0.000	NA	0.000	0.000	0.000	0.000	0.004	0.000	NA
rs28541371	T	0.005	NA	NA	NA	NA	NA	0.016	NA	NA	NA	0.000	NA	0.000	0.000	NA	0.000	NA	NA	0.000	0.000	NA	0.010	0.000	0.000	0.000	0.009	0.000	NA
rs547640504	T	0.005	NA	NA	NA	NA	NA	0.000	NA	NA	NA	0.000	NA	0.000	0.000	NA	0.000	NA	NA	0.000	0.000	NA	0.000	0.000	0.000	0.000	0.000	0.000	NA
rs368481272	T	0.000	NA	NA	NA	NA	NA	0.000	NA	NA	NA	0.000	NA	0.000	0.000	NA	0.000	NA	NA	0.005	0.000	NA	0.000	0.000	0.000	0.000	0.000	0.000	NA
rs138672257	A	0.000	NA	NA	NA	NA	NA	0.000	NA	NA	NA	0.000	NA	0.000	0.000	NA	0.000	NA	NA	0.000	0.000	NA	0.000	0.000	0.000	0.000	0.000	0.000	NA
rs150718850	A	0.000	NA	NA	NA	NA	NA	0.000	NA	NA	0.000	0.000	NA	0.000	0.010	NA	0.000	NA	NA	0.000	0.000	NA	0.000	0.015	0.022	0.015	0.000	0.019	NA
rs28921976	C	0.000	NA	NA	NA	NA	NA	0.000	NA	NA	NA	0.000	NA	0.000	0.015	NA	0.000	NA	NA	0.000	0.005	NA	0.000	0.015	0.027	0.000	0.000	0.005	NA
rs28921989	T	0.000	NA	NA	NA	NA	NA	0.000	NA	NA	NA	0.006	NA	0.000	0.020	NA	0.005	NA	NA	0.000	0.000	NA	0.000	0.005	0.005	0.049	0.000	0.014	NA
rs541886354	C	0.000	NA	NA	NA	NA	NA	0.000	NA	NA	NA	0.000	NA	0.000	0.000	NA	0.000	NA	NA	0.000	0.000	NA	0.000	0.000	0.000	0.000	0.000	0.000	NA
rs373097790	C	0.000	NA	NA	NA	NA	NA	0.000	NA	NA	NA	0.000	NA	0.000	0.000	NA	0.005	NA	NA	0.000	0.000	NA	0.000	0.000	0.000	0.000	0.000	0.000	NA
rs546562394	A	0.000	NA	NA	NA	NA	NA	0.000	NA	NA	NA	0.000	NA	0.000	0.000	NA	0.000	NA	NA	0.000	0.000	NA	0.000	0.000	0.000	0.000	0.000	0.000	NA

SNP	Allele	ACB	Afro	Aimaras	Ancash	Arequipa	Ashaninkas	ASW	Awajun	Ayacucho	Bambui	BEB	Candoshi	CDX	CEU	Chachapoyas	CHB	Chileans	Chopccas	CHS	CLM	Cusco	ESN	FIN	GBR	GIH	GWD	IBS	Iquitos
rs555516404	A	0.000	NA	NA	NA	NA	NA	0.000	NA	NA	NA	0.000	NA	0.000	0.000	NA	0.000	NA	NA	0.000	0.000	NA	0.000	0.000	0.000	0.000	0.000	0.000	NA
rs572599699	T	0.000	NA	NA	NA	NA	NA	0.000	NA	NA	NA	0.000	NA	0.000	0.000	NA	0.000	NA	NA	0.000	0.000	NA	0.000	0.000	0.000	0.000	0.000	0.000	NA
rs560691399	T	0.000	NA	NA	NA	NA	NA	0.000	NA	NA	NA	0.000	NA	0.000	0.000	NA	0.000	NA	NA	0.000	0.000	NA	0.000	0.000	0.000	0.000	0.000	0.000	NA
rs192403165	T	0.000	NA	NA	NA	NA	NA	0.008	NA	NA	NA	0.000	NA	0.000	0.000	NA	0.000	NA	NA	0.000	0.000	NA	0.000	0.000	0.000	0.000	0.000	0.000	NA
rs529959881	C	0.000	NA	NA	NA	NA	NA	0.000	NA	NA	NA	0.000	NA	0.000	0.000	NA	0.000	NA	NA	0.000	0.000	NA	0.000	0.000	0.000	0.000	0.000	0.005	NA
rs563525909	T	0.010	NA	NA	NA	NA	NA	0.008	NA	NA	NA	0.000	NA	0.000	0.000	NA	0.000	NA	NA	0.000	0.005	NA	0.000	0.000	0.000	0.000	0.000	0.000	NA
rs552948152	A	0.000	NA	NA	NA	NA	NA	0.000	NA	NA	NA	0.000	NA	0.005	0.000	NA	0.000	NA	NA	0.000	0.000	NA	0.000	0.000	0.000	0.000	0.000	0.000	NA
rs5826703	GA	0.083	NA	NA	NA	NA	NA	0.115	NA	NA	NA	0.180	NA	0.231	0.601	NA	0.301	NA	NA	0.229	0.383	NA	0.020	0.546	0.560	0.175	0.058	0.486	NA
rs571034655	T	0.000	NA	NA	NA	NA	NA	0.000	NA	NA	NA	0.000	NA	0.000	0.000	NA	0.000	NA	NA	0.000	0.000	NA	0.000	0.000	0.000	0.000	0.000	0.000	NA
rs570927174	G	0.000	NA	NA	NA	NA	NA	0.000	NA	NA	NA	0.000	NA	0.000	0.000	NA	0.000	NA	NA	0.000	0.000	NA	0.000	0.000	0.000	0.000	0.000	0.000	NA
rs552366872	T	0.000	NA	NA	NA	NA	NA	0.000	NA	NA	NA	0.000	NA	0.000	0.000	NA	0.000	NA	NA	0.000	0.000	NA	0.000	0.000	0.005	0.000	0.000	0.000	NA
rs559051628	A	0.000	NA	NA	NA	NA	NA	0.000	NA	NA	NA	0.000	NA	0.005	0.000	NA	0.000	NA	NA	0.000	0.000	NA	0.000	0.000	0.000	0.000	0.000	0.000	NA
rs556312871	A	0.000	NA	NA	NA	NA	NA	0.000	NA	NA	NA	0.000	NA	0.000	0.000	NA	0.000	NA	NA	0.000	0.000	NA	0.000	0.000	0.000	0.000	0.000	0.000	NA
rs567001549	G	0.000	NA	NA	NA	NA	NA	0.000	NA	NA	NA	0.000	NA	0.000	0.000	NA	0.000	NA	NA	0.000	0.000	NA	0.000	0.000	0.000	0.000	0.000	0.000	NA
rs533301686	A	0.000	NA	NA	NA	NA	NA	0.000	NA	NA	NA	0.000	NA	0.000	0.000	NA	0.000	NA	NA	0.000	0.000	NA	0.000	0.000	0.000	0.000	0.000	0.000	NA
rs13676	A	0.005	0.149	0.375	0.208	0.370	0.486	0.016	0.544	0.286	0.046	0.070	0.563	0.032	0.066	0.463	0.024	0.7143	0.471	0.024	0.160	0.222	0.000	0.066	0.066	0.117	0.009	0.084	0.519
rs563356581	A	0.000	NA	NA	NA	NA	NA	0.000	NA	NA	NA	0.000	NA	0.000	0.000	NA	0.000	NA	NA	0.000	0.000	NA	0.000	0.000	0.000	0.000	0.000	0.000	NA
rs111929936	A	0.000	0.000	0.000	0.000	0.000	0.000	0.025	0.000	0.000	0.017	0.000	0.000	0.000	0.000	0.000	0.000	NA	0.000	0.000	0.005	0.000	0.020	0.000	0.000	0.000	0.013	0.000	0.000
rs151017812	T	0.000	NA	NA	NA	NA	NA	0.000	NA	NA	NA	0.000	NA	0.000	0.000	NA	0.000	NA	NA	0.000	0.000	NA	0.000	0.000	0.000	0.000	0.000	0.000	NA
rs28992485	T	0.141	NA	NA	NA	NA	NA	0.238	NA	NA	NA	0.320	NA	0.258	0.066	NA	0.233	NA	NA	0.243	0.106	NA	0.242	0.162	0.082	0.257	0.270	0.108	NA
rs578018107	A	0.000	NA	NA	NA	NA	NA	0.000	NA	NA	NA	0.000	NA	0.000	0.000	NA	0.000	NA	NA	0.000	0.000	NA	0.000	0.000	0.000	0.000	0.000	0.000	NA
rs543483161	T	0.000	NA	NA	NA	NA	NA	0.000	NA	NA	NA	0.000	NA	0.000	0.000	NA	0.000	NA	NA	0.000	0.000	NA	0.000	0.000	0.000	0.000	0.000	0.000	NA
rs368686315	C	0.000	NA	NA	NA	NA	NA	0.000	NA	NA	NA	0.000	NA	0.011	0.000	NA	0.005	NA	NA	0.000	0.000	NA	0.000	0.000	0.000	0.000	0.000	0.000	NA
rs535212456	T	0.000	NA	NA	NA	NA	NA	0.000	NA	NA	NA	0.000	NA	0.000	0.000	NA	0.000	NA	NA	0.000	0.000	NA	0.000	0.000	0.000	0.000	0.004	0.000	NA
rs202212562	T	0.000	NA	NA	NA	NA	NA	0.000	NA	NA	NA	0.000	NA	0.000	0.000	NA	0.000	NA	NA	0.005	0.000	NA	0.000	0.000	0.000	0.000	0.000	0.000	NA
rs2229662	T	NA	NA	NA	NA	NA	NA	NA	NA	NA	0.000	NA	NA	NA	NA	NA	NA	NA	NA	NA	NA	NA	NA	NA	NA	NA	NA	NA	NA
rs150860707	T	0.000	NA	NA	NA	NA	NA	0.000	NA	NA	NA	0.000	NA	0.000	0.000	NA	0.000	NA	NA	0.000	0.000	NA	0.010	0.000	0.000	0.000	0.000	0.000	NA
rs543519383	A	0.000	NA	NA	NA	NA	NA	0.000	NA	NA	NA	0.000	NA	0.000	0.000	NA	0.000	NA	NA	0.000	0.005	NA	0.000	0.000	0.000	0.000	0.000	0.000	NA
rs199725376	T	0.000	NA	NA	NA	NA	NA	0.000	NA	NA	NA	0.006	NA	0.000	0.000	NA	0.005	NA	NA	0.000	0.000	NA	0.000	0.000	0.000	0.034	0.000	0.000	NA
rs10402344	T	0.260	NA	NA	NA	NA	NA	0.385	NA	NA	NA	0.773	NA	0.887	0.889	NA	0.869	NA	NA	0.881	0.692	NA	0.197	0.849	0.857	0.718	0.283	0.790	NA
rs200189683	A	0.000	NA	NA	NA	NA	NA	0.000	NA	NA	NA	0.000	NA	0.000	0.000	NA	0.000	NA	NA	0.000	0.000	NA	0.000	0.000	0.000	0.000	0.000	0.000	NA
rs113445094	T	NA	NA	NA	NA	NA	NA	NA	NA	NA	NA	NA	NA	NA	NA	NA	NA	NA	NA	NA	NA	NA	NA	NA	NA	NA	NA	NA	NA
rs527594490	G	0.000	NA	NA	NA	NA	NA	0.000	NA	NA	NA	0.000	NA	0.000	0.000	NA	0.000	NA	NA	0.000	0.000	NA	0.000	0.000	0.000	0.000	0.000	0.000	NA
rs545107557	T	0.000	NA	NA	NA	NA	NA	0.000	NA	NA	NA	0.000	NA	0.000	0.000	NA	0.000	NA	NA	0.000	0.000	NA	0.000	0.000	0.000	0.000	0.000	0.000	NA
rs7259828	T	0.177	NA	NA	NA	NA	NA	0.156	NA	NA	NA	0.076	NA	0.054	0.106	NA	0.063	0.7143	NA	0.067	0.223	NA	0.202	0.106	0.110	0.126	0.190	0.145	NA
rs112251245	T	0.031	NA	NA	NA	NA	NA	0.049	NA	NA	NA	0.000	NA	0.000	0.000	NA	0.000	NA	NA	0.000	0.000	NA	0.061	0.000	0.000	0.000	0.053	0.000	NA
rs557528051	G	0.005	NA	NA	NA	NA	NA	0.000	NA	NA	NA	0.000	NA	0.000	0.000	NA	0.000	NA	NA	0.000	0.000	NA	0.000	0.000	0.000	0.000	0.000	0.000	NA
rs540128503	T	0.000	NA	NA	NA	NA	NA	0.000	NA	NA	NA	0.000	NA	0.000	0.000	NA	0.000	NA	NA	0.000	0.000	NA	0.000	0.000	0.000	0.000	0.000	0.000	NA
rs529018328	T	0.000	NA	NA	NA	NA	NA	0.000	NA	NA	NA	0.000	NA	0.011	0.000	NA	0.000	NA	NA	0.000	0.000	NA	0.000	0.000	0.000	0.000	0.000	0.000	NA
rs183742660	A	0.000	NA	NA	NA	NA	NA	0.000	NA	NA	NA	0.000	NA	0.000	0.000	NA	0.000	NA	NA	0.000	0.000	NA	0.000	0.000	0.000	0.000	0.000	0.000	NA
rs572465381	GGGC	0.000	NA	NA	NA	NA	NA	0.016	NA	NA	NA	0.000	NA	0.000	0.000	NA	0.000	NA	NA	0.000	0.000	NA	0.010	0.000	0.000	0.000	0.004	0.000	NA
rs549282195	GAGAT	0.010	NA	NA	NA	NA	NA	0.000	NA	NA	NA	0.000	NA	0.000	0.000	NA	0.000	NA	NA	0.000	0.000	NA	0.005	0.000	0.000	0.000	0.000	0.000	NA
rs545279552	C	0.000	NA	NA	NA	NA	NA	0.000	NA	NA	NA	0.000	NA	0.000	0.000	NA	0.000	NA	NA	0.000	0.000	NA	0.000	0.000	0.000	0.000	0.000	0.000	NA
rs201531347	A	0.000	NA	NA	NA	NA	NA	0.000	NA	NA	NA	0.000	NA	0.000	0.000	NA	0.000	NA	NA	0.000	0.000	NA	0.000	0.000	0.000	0.000	0.000	0.000	NA
rs566474268	A	0.000	NA	NA	NA	NA	NA	0.000	NA	NA	NA	0.000	NA	0.000	0.000	NA	0.000	NA	NA	0.000	0.000	NA	0.000	0.000	0.000	0.000	0.000	0.000	NA

SNP	Allele	ACB	Afro	Aimaras	Ancash	Arequipa	Ashaninkas	ASW	Awajun	Ayacucho	Bambui	BEB	Candoshi	CDX	CEU	Chachapoyas	CHB	Chileans	Chopccas	CHS	CLM	Cusco	ESN	FIN	GBR	GIH	GWD	IBS	Iquitos
rs560075963	C	0.000	NA	NA	NA	NA	NA	0.000	NA	NA	NA	0.000	NA	0.000	0.000	NA	0.000	NA	NA	0.000	0.000	NA	0.000	0.000	0.000	0.000	0.004	0.000	NA
rs555375779	T	0.000	NA	NA	NA	NA	NA	0.000	NA	NA	NA	0.000	NA	0.000	0.000	NA	0.000	NA	NA	0.000	0.000	NA	0.000	0.000	0.000	0.015	0.000	0.000	NA
rs551356579	T	0.000	NA	NA	NA	NA	NA	0.008	NA	NA	NA	0.000	NA	0.000	0.000	NA	0.000	NA	NA	0.000	0.000	NA	0.000	0.000	0.000	0.000	0.000	0.000	NA
rs541386550	T	0.000	NA	NA	NA	NA	NA	0.000	NA	NA	NA	0.000	NA	0.000	0.005	NA	0.000	NA	NA	0.000	0.000	NA	0.000	0.000	0.000	0.000	0.000	0.005	NA
rs184596705	A	0.000	NA	NA	NA	NA	NA	0.000	NA	NA	NA	0.000	NA	0.000	0.000	NA	0.000	NA	NA	0.000	0.000	NA	0.000	0.000	0.000	0.000	0.000	0.000	NA
rs139397975	A	0.000	NA	NA	NA	NA	NA	0.000	NA	NA	NA	0.000	NA	0.000	0.000	NA	0.000	NA	NA	0.000	0.000	NA	0.000	0.000	0.000	0.000	0.000	0.000	NA
rs567401469	T	0.000	NA	NA	NA	NA	NA	0.000	NA	NA	NA	0.000	NA	0.000	0.000	NA	0.000	NA	NA	0.000	0.000	NA	0.000	0.000	0.000	0.000	0.000	0.000	NA
rs11473	T	0.078	NA	NA	NA	NA	NA	0.148	NA	NA	0.006	0.000	NA	0.000	0.000	NA	0.000	NA	NA	0.000	0.011	NA	0.106	0.000	0.000	0.000	0.208	0.000	NA
rs192885278	A	0.000	NA	NA	NA	NA	NA	0.000	NA	NA	NA	0.000	NA	0.000	0.000	NA	0.000	NA	NA	0.000	0.000	NA	0.000	0.000	0.000	0.000	0.000	0.000	NA
rs572733540	G	0.224	NA	NA	NA	NA	NA	0.205	NA	NA	NA	0.384	NA	0.043	0.071	NA	0.005	NA	NA	0.048	0.149	NA	0.369	0.040	0.022	0.209	0.460	0.051	NA
rs145675941	A	0.000	NA	NA	NA	NA	NA	0.000	NA	NA	NA	0.000	NA	0.000	0.000	NA	0.000	NA	NA	0.000	0.000	NA	0.010	0.000	0.000	0.000	0.000	0.000	NA
rs547384835	T	0.000	NA	NA	NA	NA	NA	0.000	NA	NA	NA	0.000	NA	0.005	0.000	NA	0.000	NA	NA	0.000	0.000	NA	0.000	0.000	0.000	0.000	0.000	0.000	NA
rs140385832	C	0.000	NA	NA	NA	NA	NA	0.000	NA	NA	NA	0.000	NA	0.000	0.000	NA	0.000	NA	NA	0.000	0.000	NA	0.000	0.000	0.000	0.000	0.000	0.000	NA
rs13235	T	0.016	NA	NA	NA	NA	NA	0.008	NA	NA	0.000	0.000	NA	0.000	0.000	NA	0.000	NA	NA	0.000	0.005	NA	0.000	0.000	0.000	0.000	0.004	0.000	NA
rs573279565	A	0.000	NA	NA	NA	NA	NA	0.000	NA	NA	NA	0.000	NA	0.000	0.000	NA	0.000	NA	NA	0.000	0.000	NA	0.000	0.000	0.000	0.000	0.004	0.000	NA
rs117933091	A	0.000	NA	NA	NA	NA	NA	0.000	NA	NA	NA	0.006	NA	0.000	0.000	NA	0.005	NA	NA	0.010	0.000	NA	0.000	0.000	0.000	0.034	0.000	0.000	NA
rs2238544	A	0.151	NA	NA	NA	NA	NA	0.197	NA	NA	NA	0.244	NA	0.231	0.662	NA	0.301	0.1786	NA	0.219	0.436	NA	0.066	0.586	0.610	0.218	0.097	0.523	NA
rs372040583	A	0.000	NA	NA	NA	NA	NA	0.000	NA	NA	NA	0.000	NA	0.000	0.000	NA	0.005	NA	NA	0.000	0.000	NA	0.000	0.000	0.000	0.000	0.000	0.000	NA
rs530681399	T	0.000	NA	NA	NA	NA	NA	0.000	NA	NA	NA	0.000	NA	0.000	0.000	NA	0.000	NA	NA	0.000	0.000	NA	0.000	0.000	0.000	0.000	0.000	0.000	NA
rs191110653	A	0.016	NA	NA	NA	NA	NA	0.000	NA	NA	NA	0.017	NA	0.000	0.000	NA	0.000	NA	NA	0.000	0.005	NA	0.005	0.000	0.000	0.034	0.013	0.000	NA
rs3764938	T	0.396	NA	NA	NA	NA	NA	0.443	NA	NA	NA	0.407	NA	0.317	0.177	NA	0.301	0.7143	NA	0.310	0.356	NA	0.530	0.268	0.203	0.456	0.615	0.257	NA
rs139776734	A	0.000	NA	NA	NA	NA	NA	0.000	NA	NA	NA	0.000	NA	0.000	0.000	NA	0.000	NA	NA	0.000	0.000	NA	0.000	0.000	0.000	0.000	0.000	0.000	NA
rs575050179	A	0.000	NA	NA	NA	NA	NA	0.000	NA	NA	NA	0.000	NA	0.005	0.000	NA	0.000	NA	NA	0.000	0.000	NA	0.000	0.000	0.000	0.000	0.000	0.000	NA
rs572043068	A	0.000	NA	NA	NA	NA	NA	0.000	NA	NA	NA	0.000	NA	0.000	0.000	NA	0.000	NA	NA	0.000	0.005	NA	0.000	0.000	0.000	0.000	0.000	0.000	NA
rs2238545	C	0.141	NA	NA	NA	NA	NA	0.238	NA	NA	NA	0.320	NA	0.258	0.066	NA	0.233	NA	NA	0.243	0.106	NA	0.242	0.162	0.082	0.252	0.270	0.108	NA
rs533373164	T	0.000	NA	NA	NA	NA	NA	0.000	NA	NA	NA	0.000	NA	0.000	0.005	NA	0.000	NA	NA	0.005	0.011	NA	0.000	0.000	0.016	0.000	0.000	0.009	NA
rs201635915	A	0.000	NA	NA	NA	NA	NA	0.000	NA	NA	NA	0.000	NA	0.000	0.000	NA	0.000	NA	NA	0.000	0.000	NA	0.000	0.000	0.000	0.000	0.000	0.000	NA
rs541344713	G	0.000	NA	NA	NA	NA	NA	0.000	NA	NA	NA	0.000	NA	0.000	0.000	NA	0.000	NA	NA	0.000	0.000	NA	0.000	0.000	0.000	0.000	0.004	0.000	NA
rs567649209	T	0.000	NA	NA	NA	NA	NA	0.000	NA	NA	NA	0.000	NA	0.005	0.000	NA	0.000	NA	NA	0.000	0.000	NA	0.000	0.000	0.000	0.000	0.000	0.000	NA
rs529458335	T	0.000	NA	NA	NA	NA	NA	0.000	NA	NA	NA	0.000	NA	0.000	0.000	NA	0.000	NA	NA	0.000	0.000	NA	0.000	0.000	0.000	0.005	0.000	0.000	NA
rs186158048	T	0.000	NA	NA	NA	NA	NA	0.000	NA	NA	NA	0.000	NA	0.000	0.000	NA	0.000	NA	NA	0.000	0.000	NA	0.005	0.000	0.000	0.000	0.000	0.000	NA
rs560636050	A	0.000	NA	NA	NA	NA	NA	0.000	NA	NA	NA	0.000	NA	0.000	0.000	NA	0.000	NA	NA	0.000	0.000	NA	0.000	0.000	0.000	0.000	0.000	0.000	NA
rs111841168	C	NA	NA	NA	NA	NA	NA	NA	NA	NA	NA	NA	NA	NA	NA	NA	NA	NA	NA	NA	NA	NA	NA	NA	NA	NA	NA	NA	NA
rs137922704	T	0.016	NA	NA	NA	NA	NA	0.008	NA	NA	NA	0.000	NA	0.000	0.000	NA	0.000	NA	NA	0.000	0.005	NA	0.000	0.000	0.000	0.000	0.004	0.000	NA
rs28992470	A	0.000	NA	NA	NA	NA	NA	0.008	NA	NA	0.017	0.006	NA	0.000	0.010	NA	0.000	NA	NA	0.000	0.005	NA	0.000	0.010	0.005	0.005	0.000	0.028	NA
rs190631157	T	0.016	NA	NA	NA	NA	NA	0.016	NA	NA	NA	0.000	NA	0.000	0.000	NA	0.000	NA	NA	0.000	0.000	NA	0.035	0.000	0.000	0.000	0.009	0.000	NA
rs28921991	A	0.021	NA	NA	NA	NA	NA	0.041	NA	NA	NA	0.000	NA	0.000	0.000	NA	0.000	NA	NA	0.005	0.005	NA	0.056	0.000	0.000	0.000	0.044	0.000	NA
rs577780415	G	0.000	NA	NA	NA	NA	NA	0.000	NA	NA	NA	0.006	NA	0.000	0.000	NA	0.000	NA	NA	0.000	0.000	NA	0.000	0.000	0.000	0.000	0.000	0.000	NA
rs1063378	C	NA	NA	NA	NA	NA	NA	NA	NA	NA	NA	NA	NA	NA	NA	NA	NA	NA	NA	NA	NA	NA	NA	NA	NA	NA	NA	NA	NA
rs140499328	T	0.141	NA	NA	NA	NA	NA	0.238	NA	NA	NA	0.320	NA	0.258	0.066	NA	0.233	NA	NA	0.243	0.106	NA	0.242	0.162	0.082	0.252	0.266	0.108	NA
rs28921972	A	0.000	NA	NA	NA	NA	NA	0.008	NA	NA	0.000	0.000	NA	0.000	0.000	NA	0.000	NA	NA	0.000	0.000	NA	0.000	0.000	0.000	0.000	0.004	0.000	NA
rs191842753	T	0.000	NA	NA	NA	NA	NA	0.000	NA	NA	NA	0.000	NA	0.000	0.000	NA	0.000	NA	NA	0.000	0.005	NA	0.000	0.000	0.016	0.000	0.000	0.005	NA
rs150369286	T	0.000	NA	NA	NA	NA	NA	0.000	NA	NA	NA	0.000	NA	0.000	0.000	NA	0.000	NA	NA	0.000	0.000	NA	0.010	0.000	0.000	0.000	0.000	0.000	NA
rs28992484	T	0.141	NA	NA	NA	NA	NA	0.238	NA	NA	NA	0.320	NA	0.258	0.066	NA	0.233	NA	NA	0.243	0.106	NA	0.242	0.162	0.082	0.262	0.270	0.108	NA
rs140722203	A	0.000	NA	NA	NA	NA	NA	0.000	NA	NA	NA	0.000	NA	0.000	0.000	NA	0.000	NA	NA	0.000	0.000	NA	0.000	0.000	0.005	0.000	0.000	0.000	NA

SNP	Allele	ACB	Afro	Aimaras	Ancash	Arequipa	Ashaninkas	ASW	Awajun	Ayacucho	Bambui	BEB	Candoshi	CDX	CEU	Chachapoyas	CHB	Chileans	Chopccas	CHS	CLM	Cusco	ESN	FIN	GBR	GIH	GWD	IBS	Iquitos
rs546300301	G	0.000	NA	NA	NA	NA	NA	0.000	NA	NA	NA	0.000	NA	0.000	0.000	NA	0.000	NA	NA	0.000	0.000	NA	0.000	0.000	0.000	0.000	0.004	0.000	NA
rs531900250	A	0.000	NA	NA	NA	NA	NA	0.000	NA	NA	NA	0.000	NA	0.000	0.000	NA	0.000	NA	NA	0.000	0.000	NA	0.000	0.000	0.000	0.000	0.000	0.000	NA
rs577921461	T	0.000	NA	NA	NA	NA	NA	0.000	NA	NA	NA	0.000	NA	0.000	0.000	NA	0.000	NA	NA	0.000	0.000	NA	0.000	0.000	0.000	0.000	0.004	0.000	NA
rs201403607	A	0.000	NA	NA	NA	NA	NA	0.000	NA	NA	NA	0.000	NA	0.000	0.000	NA	0.000	NA	NA	0.000	0.000	NA	0.000	0.000	0.000	0.000	0.009	0.000	NA
rs7255181	G	0.021	NA	NA	NA	NA	NA	0.025	NA	NA	NA	0.000	NA	0.000	0.000	NA	0.000	NA	NA	0.000	0.000	NA	0.010	0.000	0.000	0.000	0.040	0.000	NA
rs141583892	T	0.000	NA	NA	NA	NA	NA	0.008	NA	NA	NA	0.000	NA	0.000	0.000	NA	0.000	NA	NA	0.000	0.000	NA	0.015	0.000	0.000	0.000	0.035	0.000	NA
rs113790346	G	NA	NA	NA	NA	NA	NA	NA	NA	NA	NA	NA	NA	NA	NA	NA	NA	NA	NA	NA	NA	NA	NA	NA	NA	NA	NA	NA	NA
rs551388840	T	0.000	NA	NA	NA	NA	NA	0.000	NA	NA	NA	0.000	NA	0.000	0.000	NA	0.000	NA	NA	0.000	0.000	NA	0.000	0.000	0.000	0.000	0.000	0.000	NA
rs542552024	A	0.000	NA	NA	NA	NA	NA	0.000	NA	NA	NA	0.006	NA	0.000	0.000	NA	0.000	NA	NA	0.000	0.000	NA	0.000	0.000	0.000	0.000	0.000	0.000	NA
rs552594392	A	0.000	NA	NA	NA	NA	NA	0.000	NA	NA	NA	0.000	NA	0.000	0.000	NA	0.000	NA	NA	0.000	0.000	NA	0.000	0.000	0.000	0.000	0.000	0.000	NA
rs182826947	A	0.000	NA	NA	NA	NA	NA	0.000	NA	NA	NA	0.000	NA	0.000	0.000	NA	0.000	NA	NA	0.000	0.000	NA	0.000	0.000	0.000	0.000	0.000	0.000	NA
rs188799878	A	0.000	NA	NA	NA	NA	NA	0.000	NA	NA	NA	0.000	NA	0.000	0.000	NA	0.000	NA	NA	0.000	0.000	NA	0.000	0.000	0.000	0.000	0.000	0.000	NA
rs11879178	A	0.333	NA	NA	NA	NA	NA	0.262	NA	NA	NA	0.361	NA	0.457	0.167	NA	0.398	NA	NA	0.462	0.213	NA	0.313	0.147	0.198	0.369	0.376	0.220	NA
rs28993669	C	NA	NA	NA	NA	NA	NA	NA	NA	NA	0.000	NA	NA	NA	NA	NA	NA	NA	NA	NA	NA	NA	NA	NA	NA	NA	NA	NA	NA
rs191246732	A	0.000	NA	NA	NA	NA	NA	0.000	NA	NA	NA	0.000	NA	0.000	0.000	NA	0.000	NA	NA	0.000	0.000	NA	0.000	0.000	0.000	0.000	0.000	0.000	NA
rs551261955	T	0.000	NA	NA	NA	NA	NA	0.000	NA	NA	NA	0.000	NA	0.000	0.000	NA	0.000	NA	NA	0.000	0.000	NA	0.000	0.000	0.000	0.000	0.000	0.000	NA
rs146186991	A	0.000	NA	NA	NA	NA	NA	0.000	NA	NA	0.017	0.000	NA	0.000	0.005	NA	0.000	NA	NA	0.000	0.000	NA	0.000	0.005	0.027	0.000	0.000	0.019	NA
rs544039773	T	0.000	NA	NA	NA	NA	NA	0.000	NA	NA	NA	0.000	NA	0.000	0.000	NA	0.000	NA	NA	0.000	0.000	NA	0.000	0.000	0.000	0.000	0.000	0.000	NA
rs554365832	A	0.000	NA	NA	NA	NA	NA	0.000	NA	NA	NA	0.000	NA	0.000	0.000	NA	0.000	NA	NA	0.000	0.000	NA	0.000	0.000	0.000	0.005	0.000	0.000	NA
rs138039913	T	0.000	NA	NA	NA	NA	NA	0.000	NA	NA	NA	0.000	NA	0.011	0.000	NA	0.019	NA	NA	0.033	0.000	NA	0.000	0.000	0.000	0.000	0.000	0.000	NA
rs541089761	A	0.000	NA	NA	NA	NA	NA	0.000	NA	NA	NA	0.000	NA	0.000	0.000	NA	0.000	NA	NA	0.000	0.000	NA	0.000	0.000	0.000	0.000	0.000	0.000	NA
rs28921985	A	0.000	NA	NA	NA	NA	NA	0.016	NA	NA	0.000	0.000	NA	0.000	0.000	NA	0.000	NA	NA	0.000	0.005	NA	0.020	0.000	0.000	0.000	0.013	0.000	NA
rs529207268	G	0.000	NA	NA	NA	NA	NA	0.000	NA	NA	NA	0.000	NA	0.000	0.000	NA	0.000	NA	NA	0.000	0.005	NA	0.000	0.000	0.000	0.000	0.000	0.000	NA
rs190217060	A	0.000	NA	NA	NA	NA	NA	0.000	NA	NA	NA	0.000	NA	0.000	0.000	NA	0.000	NA	NA	0.000	0.000	NA	0.000	0.000	0.000	0.000	0.000	0.000	NA
rs552965493	T	0.005	NA	NA	NA	NA	NA	0.000	NA	NA	NA	0.000	NA	0.000	0.005	NA	0.000	NA	NA	0.000	0.000	NA	0.000	0.000	0.000	0.000	0.000	0.000	NA
rs545068935	G	0.000	NA	NA	NA	NA	NA	0.000	NA	NA	NA	0.000	NA	0.000	0.000	NA	0.000	NA	NA	0.000	0.000	NA	0.000	0.000	0.000	0.000	0.004	0.000	NA
rs540123195	A	0.000	NA	NA	NA	NA	NA	0.000	NA	NA	NA	0.000	NA	0.000	0.000	NA	0.000	NA	NA	0.000	0.000	NA	0.005	0.000	0.000	0.000	0.000	0.000	NA
rs12611131	T	0.141	NA	NA	NA	NA	NA	0.238	NA	NA	NA	0.326	NA	0.258	0.071	NA	0.233	NA	NA	0.243	0.128	NA	0.242	0.162	0.093	0.262	0.270	0.112	NA
rs200467897	T	0.000	NA	NA	NA	NA	NA	0.000	NA	NA	NA	0.000	NA	0.000	0.005	NA	0.000	NA	NA	0.000	0.000	NA	0.000	0.000	0.000	0.000	0.000	0.000	NA
rs28992469	T	0.000	NA	NA	NA	NA	NA	0.000	NA	NA	0.000	0.000	NA	0.000	0.000	NA	0.000	NA	NA	0.000	0.000	NA	0.000	0.000	0.000	0.000	0.000	0.000	NA
rs2072307	C	0.151	NA	NA	NA	NA	NA	0.254	NA	NA	NA	0.314	NA	0.199	0.066	NA	0.155	NA	NA	0.195	0.106	NA	0.253	0.162	0.077	0.238	0.279	0.108	NA
rs535566277	A	0.000	NA	NA	NA	NA	NA	0.000	NA	NA	NA	0.000	NA	0.005	0.000	NA	0.000	NA	NA	0.000	0.000	NA	0.000	0.000	0.000	0.000	0.000	0.000	NA
rs145461969	T	0.000	NA	NA	NA	NA	NA	0.000	NA	NA	NA	0.000	NA	0.000	0.000	NA	0.000	NA	NA	0.000	0.000	NA	0.000	0.000	0.000	0.000	0.000	0.000	NA
rs568389785	T	0.000	NA	NA	NA	NA	NA	0.000	NA	NA	NA	0.000	NA	0.000	0.000	NA	0.000	NA	NA	0.000	0.000	NA	0.000	0.000	0.000	0.000	0.000	0.000	NA
rs56043551	T	NA	NA	NA	NA	NA	NA	NA	NA	NA	NA	NA	NA	NA	NA	NA	NA	NA	NA	NA	NA	NA	NA	NA	NA	NA	NA	NA	NA
rs191693960	T	0.000	NA	NA	NA	NA	NA	0.000	NA	NA	NA	0.000	NA	0.000	0.000	NA	0.000	NA	NA	0.000	0.000	NA	0.010	0.000	0.000	0.000	0.000	0.000	NA
rs28921968	G	NA	NA	NA	NA	NA	NA	NA	NA	NA	0.006	NA	NA	NA	NA	NA	NA	NA	NA	NA	NA	NA	NA	NA	NA	NA	NA	NA	NA
rs549983564	T	0.000	NA	NA	NA	NA	NA	0.000	NA	NA	NA	0.000	NA	0.000	0.000	NA	0.000	NA	NA	0.000	0.000	NA	0.000	0.000	0.000	0.000	0.000	0.000	NA
rs200630157	T	0.000	NA	NA	NA	NA	NA	0.008	NA	NA	NA	0.000	NA	0.000	0.000	NA	0.000	NA	NA	0.000	0.005	NA	0.000	0.000	0.000	0.000	0.000	0.000	NA
rs550013368	A	0.021	NA	NA	NA	NA	NA	0.025	NA	NA	NA	0.000	NA	0.000	0.000	NA	0.000	NA	NA	0.000	0.000	NA	0.020	0.000	0.000	0.000	0.018	0.000	NA
rs191389346	T	0.000	NA	NA	NA	NA	NA	0.000	NA	NA	NA	0.000	NA	0.000	0.000	NA	0.000	NA	NA	0.000	0.000	NA	0.000	0.000	0.005	0.000	0.000	0.000	NA
rs28921983	G	NA	NA	NA	NA	NA	NA	NA	NA	NA	0.000	NA	NA	NA	NA	NA	NA	NA	NA	NA	NA	NA	NA	NA	NA	NA	NA	NA	NA
rs564546624	T	0.000	NA	NA	NA	NA	NA	0.000	NA	NA	NA	0.000	NA	0.000	0.000	NA	0.000	NA	NA	0.000	0.000	NA	0.000	0.000	0.000	0.005	0.000	0.000	NA
rs150791055	T	0.000	NA	NA	NA	NA	NA	0.000	NA	NA	NA	0.000	NA	0.000	0.000	NA	0.000	NA	NA	0.000	0.000	NA	0.000	0.000	0.000	0.000	0.004	0.000	NA
rs202135216	T	0.021	NA	NA	NA	NA	NA	0.008	NA	NA	NA	0.000	NA	0.000	0.000	NA	0.000	NA	NA	0.000	0.005	NA	0.000	0.000	0.000	0.000	0.027	0.000	NA

SNP	Allele	ACB	Afro	Aimaras	Ancash	Arequipa	Ashaninkas	ASW	Awajun	Ayacucho	Bambui	BEB	Candoshi	CDX	CEU	Chachapoyas	CHB	Chileans	Chopccas	CHS	CLM	Cusco	ESN	FIN	GBR	GIH	GWD	IBS	Iquitos
rs575212330	T	0.000	NA	NA	NA	NA	NA	0.000	NA	NA	NA	0.000	NA	0.000	0.000	NA	0.000	NA	NA	0.000	0.000	NA	0.000	0.000	0.000	0.000	0.000	0.000	NA
rs559570766	T	0.000	NA	NA	NA	NA	NA	0.000	NA	NA	NA	0.000	NA	0.000	0.000	NA	0.000	NA	NA	0.000	0.000	NA	0.000	0.000	0.000	0.000	0.004	0.000	NA
rs151237303	T	0.141	NA	NA	NA	NA	NA	0.238	NA	NA	NA	0.320	NA	0.258	0.066	NA	0.233	NA	NA	0.243	0.106	NA	0.242	0.162	0.082	0.252	0.266	0.108	NA
rs369186206	A	0.005	NA	NA	NA	NA	NA	0.000	NA	NA	NA	0.000	NA	0.000	0.000	NA	0.000	NA	NA	0.000	0.000	NA	0.000	0.000	0.000	0.000	0.000	0.000	NA
rs28921975	A	0.000	NA	NA	NA	NA	NA	0.025	NA	NA	NA	0.000	NA	0.000	0.000	NA	0.000	NA	NA	0.000	0.000	NA	0.015	0.000	0.000	0.000	0.000	0.000	NA
rs538891893	G	0.000	NA	NA	NA	NA	NA	0.000	NA	NA	NA	0.000	NA	0.000	0.000	NA	0.000	NA	NA	0.000	0.000	NA	0.000	0.000	0.000	0.000	0.000	0.000	NA
rs566302789	T	0.000	NA	NA	NA	NA	NA	0.000	NA	NA	NA	0.000	NA	0.000	0.000	NA	0.000	NA	NA	0.000	0.000	NA	0.000	0.005	0.005	0.000	0.000	0.009	NA
rs189865097	A	0.000	NA	NA	NA	NA	NA	0.000	NA	NA	NA	0.000	NA	0.000	0.000	NA	0.000	NA	NA	0.000	0.000	NA	0.000	0.000	0.000	0.000	0.000	0.000	NA
rs2283570	T	0.141	NA	NA	NA	NA	NA	0.230	NA	NA	NA	0.320	NA	0.258	0.081	NA	0.238	NA	NA	0.257	0.122	NA	0.242	0.187	0.104	0.252	0.270	0.117	NA
rs181004617	A	0.000	NA	NA	NA	NA	NA	0.000	NA	NA	NA	0.000	NA	0.000	0.000	NA	0.000	NA	NA	0.000	0.005	NA	0.000	0.000	0.005	0.000	0.000	0.000	NA
rs2283574	A	0.141	NA	NA	NA	NA	NA	0.238	NA	NA	NA	0.320	NA	0.258	0.066	NA	0.233	NA	NA	0.243	0.106	NA	0.242	0.162	0.082	0.252	0.270	0.108	NA
rs553131488	A	0.000	NA	NA	NA	NA	NA	0.000	NA	NA	NA	0.000	NA	0.000	0.000	NA	0.000	NA	NA	0.000	0.000	NA	0.000	0.000	0.005	0.000	0.000	0.000	NA
rs200432051	T	0.000	NA	NA	NA	NA	NA	0.000	NA	NA	NA	0.000	NA	0.000	0.000	NA	0.000	NA	NA	0.010	0.000	NA	0.000	0.000	0.000	0.000	0.000	0.000	NA
rs569279461	T	0.000	NA	NA	NA	NA	NA	0.000	NA	NA	NA	0.000	NA	0.000	0.000	NA	0.000	NA	NA	0.005	0.000	NA	0.000	0.000	0.000	0.000	0.000	0.000	NA
rs112785861	A	0.083	NA	NA	NA	NA	NA	0.066	NA	NA	NA	0.000	NA	0.000	0.000	NA	0.000	NA	NA	0.000	0.021	NA	0.076	0.000	0.000	0.000	0.027	0.005	NA
rs529813357	T	0.000	NA	NA	NA	NA	NA	0.000	NA	NA	NA	0.000	NA	0.000	0.000	NA	0.000	NA	NA	0.000	0.000	NA	0.000	0.005	0.022	0.000	0.000	0.000	NA
rs563512179	A	0.000	NA	NA	NA	NA	NA	0.000	NA	NA	NA	0.000	NA	0.000	0.000	NA	0.000	NA	NA	0.000	0.000	NA	0.000	0.000	0.000	0.010	0.000	0.000	NA
rs201881530	G	0.000	NA	NA	NA	NA	NA	0.000	NA	NA	NA	0.000	NA	0.000	0.000	NA	0.000	NA	NA	0.000	0.000	NA	0.000	0.005	0.000	0.000	0.000	0.000	NA
rs2283571	C	0.307	NA	NA	NA	NA	NA	0.426	NA	NA	NA	0.558	NA	0.489	0.702	NA	0.534	0.1786	NA	0.471	0.543	NA	0.313	0.722	0.643	0.432	0.376	0.603	NA
rs116073835	A	0.026	NA	NA	NA	NA	NA	0.033	NA	NA	NA	0.000	NA	0.000	0.000	NA	0.000	NA	NA	0.000	0.000	NA	0.051	0.000	0.000	0.000	0.044	0.000	NA
rs368973405	T	0.005	NA	NA	NA	NA	NA	0.000	NA	NA	NA	0.000	NA	0.000	0.000	NA	0.000	NA	NA	0.000	0.005	NA	0.010	0.000	0.000	0.000	0.004	0.000	NA
rs553337242	T	0.000	NA	NA	NA	NA	NA	0.000	NA	NA	NA	0.006	NA	0.000	0.000	NA	0.000	NA	NA	0.000	0.000	NA	0.000	0.000	0.000	0.000	0.000	0.000	NA
rs577310345	C	0.000	NA	NA	NA	NA	NA	0.000	NA	NA	NA	0.000	NA	0.000	0.000	NA	0.000	NA	NA	0.000	0.005	NA	0.000	0.000	0.000	0.000	0.000	0.000	NA
rs538315599	A	0.000	NA	NA	NA	NA	NA	0.000	NA	NA	NA	0.000	NA	0.000	0.000	NA	0.000	NA	NA	0.000	0.000	NA	0.000	0.000	0.000	0.000	0.004	0.000	NA
rs556900373	A	0.000	NA	NA	NA	NA	NA	0.000	NA	NA	NA	0.000	NA	0.000	0.000	NA	0.000	NA	NA	0.000	0.000	NA	0.000	0.000	0.000	0.000	0.004	0.000	NA
rs534224219	T	0.000	NA	NA	NA	NA	NA	0.000	NA	NA	NA	0.000	NA	0.000	0.000	NA	0.000	NA	NA	0.000	0.000	NA	0.000	0.000	0.000	0.000	0.000	0.000	NA
rs28915408	A	0.000	NA	NA	NA	NA	NA	0.000	NA	NA	NA	0.000	NA	0.000	0.025	NA	0.000	NA	NA	0.000	0.011	NA	0.000	0.040	0.044	0.010	0.000	0.014	NA
rs538902991	T	0.000	NA	NA	NA	NA	NA	0.000	NA	NA	NA	0.000	NA	0.000	0.000	NA	0.000	NA	NA	0.000	0.000	NA	0.000	0.000	0.000	0.000	0.000	0.000	NA
rs200237260	T	0.000	NA	NA	NA	NA	NA	0.000	NA	NA	NA	0.006	NA	0.032	0.000	NA	0.015	NA	NA	0.024	0.000	NA	0.000	0.000	0.000	0.000	0.000	0.000	NA
rs28992480	A	0.010	NA	NA	NA	NA	NA	0.025	NA	NA	0.000	0.000	NA	0.000	0.000	NA	0.000	NA	NA	0.000	0.000	NA	0.025	0.000	0.000	0.000	0.009	0.000	NA
rs12979852	T	0.266	NA	NA	NA	NA	NA	0.156	NA	NA	NA	0.151	NA	0.011	0.030	NA	0.019	0.5	NA	0.010	0.037	NA	0.258	0.015	0.071	0.078	0.323	0.056	NA
rs200853058	T	0.000	NA	NA	NA	NA	NA	0.000	NA	NA	NA	0.000	NA	0.000	0.000	NA	0.005	NA	NA	0.000	0.000	NA	0.000	0.000	0.000	0.000	0.000	0.000	NA
rs560866175	A	0.000	NA	NA	NA	NA	NA	0.000	NA	NA	NA	0.000	NA	0.000	0.000	NA	0.000	NA	NA	0.000	0.000	NA	0.000	0.000	0.000	0.000	0.000	0.000	NA
rs186833702	A	0.000	NA	NA	NA	NA	NA	0.000	NA	NA	NA	0.000	NA	0.000	0.000	NA	0.000	NA	NA	0.000	0.000	NA	0.000	0.000	0.000	0.000	0.000	0.000	NA
rs113907190	G	NA	NA	NA	NA	NA	NA	NA	NA	NA	NA	NA	NA	NA	NA	NA	NA	NA	NA	NA	NA	NA	NA	NA	NA	NA	NA	NA	NA
rs28921990	C	0.141	NA	NA	NA	NA	NA	0.238	NA	NA	NA	0.320	NA	0.258	0.066	NA	0.233	NA	NA	0.243	0.106	NA	0.242	0.162	0.082	0.252	0.270	0.108	NA
rs577273590	G	0.000	NA	NA	NA	NA	NA	0.000	NA	NA	NA	0.000	NA	0.000	0.000	NA	0.000	NA	NA	0.000	0.000	NA	0.000	0.000	0.000	0.000	0.004	0.000	NA
rs536730099	A	0.000	NA	NA	NA	NA	NA	0.000	NA	NA	NA	0.000	NA	0.000	0.000	NA	0.005	NA	NA	0.005	0.000	NA	0.000	0.000	0.000	0.000	0.000	0.000	NA
rs143543245	T	0.005	NA	NA	NA	NA	NA	0.016	NA	NA	NA	0.000	NA	0.000	0.000	NA	0.000	NA	NA	0.000	0.000	NA	0.015	0.000	0.000	0.000	0.000	0.000	NA
rs202113312	C	NA	NA	NA	NA	NA	NA	NA	NA	NA	NA	NA	NA	NA	NA	NA	NA	NA	NA	NA	NA	NA	NA	NA	NA	NA	NA	NA	NA
rs565922179	T	0.005	NA	NA	NA	NA	NA	0.000	NA	NA	NA	0.000	NA	0.000	0.000	NA	0.000	NA	NA	0.000	0.000	NA	0.000	0.000	0.000	0.000	0.004	0.000	NA
rs113033881	C	0.260	NA	NA	NA	NA	NA	0.303	NA	NA	NA	0.320	NA	0.258	0.066	NA	0.238	NA	NA	0.243	0.112	NA	0.338	0.162	0.082	0.252	0.363	0.112	NA
rs565509602	T	0.000	NA	NA	NA	NA	NA	0.000	NA	NA	NA	0.000	NA	0.000	0.000	NA	0.000	NA	NA	0.000	0.000	NA	0.000	0.000	0.000	0.000	0.000	0.000	NA
rs552797010	A	0.000	NA	NA	NA	NA	NA	0.000	NA	NA	NA	0.000	NA	0.000	0.000	NA	0.000	NA	NA	0.000	0.000	NA	0.000	0.000	0.000	0.000	0.000	0.000	NA
rs534935683	T	0.000	NA	NA	NA	NA	NA	0.000	NA	NA	NA	0.000	NA	0.005	0.000	NA	0.000	NA	NA	0.000	0.000	NA	0.000	0.000	0.000	0.000	0.000	0.000	NA

SNP	Allele	ACB	Afro	Aimaras	Ancash	Arequipa	Ashaninkas	ASW	Awajun	Ayacucho	Bambui	BEB	Candoshi	CDX	CEU	Chachapoyas	CHB	Chileans	Chopccas	CHS	CLM	Cusco	ESN	FIN	GBR	GIH	GWD	IBS	Iquitos
rs367579969	T	0.000	NA	NA	NA	NA	NA	0.000	NA	NA	NA	0.000	NA	0.032	0.000	NA	0.005	NA	NA	0.005	0.000	NA	0.000	0.000	0.000	0.000	0.000	0.000	NA
rs568509871	C	0.005	NA	NA	NA	NA	NA	0.000	NA	NA	NA	0.000	NA	0.000	0.000	NA	0.000	NA	NA	0.000	0.000	NA	0.000	0.000	0.000	0.000	0.000	0.000	NA
rs565826251	A	0.000	NA	NA	NA	NA	NA	0.000	NA	NA	NA	0.000	NA	0.000	0.000	NA	0.000	NA	NA	0.000	0.000	NA	0.000	0.000	0.000	0.000	0.004	0.000	NA
rs542552123	A	0.000	NA	NA	NA	NA	NA	0.000	NA	NA	NA	0.000	NA	0.000	0.000	NA	0.010	NA	NA	0.000	0.000	NA	0.000	0.000	0.000	0.000	0.000	0.000	NA
rs28992471	A	0.141	NA	NA	NA	NA	NA	0.238	NA	NA	NA	0.320	NA	0.258	0.066	NA	0.233	NA	NA	0.243	0.106	NA	0.242	0.162	0.082	0.252	0.270	0.108	NA
rs563724259	T	0.000	NA	NA	NA	NA	NA	0.000	NA	NA	NA	0.000	NA	0.011	0.000	NA	0.000	NA	NA	0.000	0.000	NA	0.000	0.000	0.000	0.000	0.000	0.000	NA
rs28921988	T	0.005	NA	NA	NA	NA	NA	0.025	NA	NA	NA	0.000	NA	0.000	0.000	NA	0.000	NA	NA	0.000	0.000	NA	0.005	0.000	0.000	0.000	0.027	0.000	NA
rs560442683	T	0.000	NA	NA	NA	NA	NA	0.000	NA	NA	NA	0.000	NA	0.005	0.000	NA	0.000	NA	NA	0.000	0.000	NA	0.000	0.000	0.000	0.000	0.004	0.000	NA
rs537692667	A	0.000	NA	NA	NA	NA	NA	0.000	NA	NA	NA	0.000	NA	0.000	0.000	NA	0.000	NA	NA	0.000	0.000	NA	0.000	0.000	0.000	0.000	0.000	0.000	NA
rs28921971	T	0.005	NA	NA	NA	NA	NA	0.016	NA	NA	0.041	0.070	NA	0.032	0.066	NA	0.024	0.7143	NA	0.024	0.160	NA	0.000	0.066	0.066	0.117	0.009	0.079	NA
rs572811560	A	0.000	NA	NA	NA	NA	NA	0.000	NA	NA	NA	0.000	NA	0.000	0.000	NA	0.005	NA	NA	0.000	0.000	NA	0.000	0.000	0.000	0.000	0.000	0.000	NA
rs28992483	T	0.141	NA	NA	NA	NA	NA	0.238	NA	NA	NA	0.320	NA	0.258	0.066	NA	0.228	NA	NA	0.243	0.106	NA	0.242	0.162	0.082	0.257	0.270	0.108	NA
rs547376405	G	0.000	NA	NA	NA	NA	NA	0.008	NA	NA	NA	0.000	NA	0.005	0.000	NA	0.000	NA	NA	0.000	0.005	NA	0.000	0.000	0.000	0.000	0.000	0.005	NA
rs138748626	T	0.000	NA	NA	NA	NA	NA	0.000	NA	NA	NA	0.000	NA	0.048	0.000	NA	0.005	NA	NA	0.000	0.000	NA	0.000	0.000	0.000	0.000	0.000	0.000	NA
rs528453463	A	0.000	NA	NA	NA	NA	NA	0.000	NA	NA	NA	0.000	NA	0.000	0.000	NA	0.000	NA	NA	0.000	0.000	NA	0.000	0.000	0.000	0.000	0.000	0.000	NA
rs552838011	G	0.000	NA	NA	NA	NA	NA	0.000	NA	NA	NA	0.000	NA	0.000	0.000	NA	0.000	NA	NA	0.000	0.005	NA	0.000	0.000	0.000	0.000	0.000	0.000	NA
rs544905487	T	0.000	NA	NA	NA	NA	NA	0.000	NA	NA	NA	0.000	NA	0.000	0.000	NA	0.000	NA	NA	0.000	0.000	NA	0.000	0.000	0.000	0.000	0.004	0.000	NA
rs139995866	A	0.000	NA	NA	NA	NA	NA	0.000	NA	NA	NA	0.000	NA	0.016	0.000	NA	0.015	NA	NA	0.014	0.000	NA	0.000	0.000	0.000	0.000	0.000	0.000	NA
rs556660637	A	0.000	NA	NA	NA	NA	NA	0.000	NA	NA	NA	0.000	NA	0.000	0.000	NA	0.000	NA	NA	0.000	0.000	NA	0.000	0.000	0.000	0.000	0.000	0.000	NA
rs559190146	A	0.000	NA	NA	NA	NA	NA	0.000	NA	NA	NA	0.000	NA	0.000	0.000	NA	0.000	NA	NA	0.000	0.000	NA	0.000	0.000	0.000	0.015	0.000	0.000	NA
rs544661215	T	0.010	NA	NA	NA	NA	NA	0.008	NA	NA	NA	0.000	NA	0.000	0.000	NA	0.000	NA	NA	0.000	0.000	NA	0.010	0.000	0.000	0.000	0.004	0.000	NA
rs2238542	T	0.141	NA	NA	NA	NA	NA	0.238	NA	NA	0.114	0.320	NA	0.258	0.066	NA	0.233	NA	NA	0.243	0.106	NA	0.242	0.162	0.082	0.252	0.270	0.108	NA
rs571648795	T	0.000	NA	NA	NA	NA	NA	0.000	NA	NA	NA	0.000	NA	0.005	0.000	NA	0.000	NA	NA	0.000	0.000	NA	0.000	0.000	0.000	0.000	0.000	0.000	NA
rs567013917	A	0.000	NA	NA	NA	NA	NA	0.000	NA	NA	NA	0.000	NA	0.000	0.000	NA	0.000	NA	NA	0.000	0.000	NA	0.000	0.000	0.000	0.005	0.000	0.000	NA
rs187005996	T	0.016	NA	NA	NA	NA	NA	0.016	NA	NA	NA	0.000	NA	0.000	0.000	NA	0.000	NA	NA	0.000	0.000	NA	0.005	0.000	0.000	0.000	0.004	0.000	NA
rs28473389	T	0.008	NA	NA	NA	NA	NA	0.021	NA	NA	NA	0.071	NA	0.032	0.066	NA	0.025	0.7143	NA	0.024	0.167	NA	0.000	0.066	0.066	0.124	0.010	0.080	NA
rs71334826	C	0.021	NA	NA	NA	NA	NA	0.025	NA	NA	NA	0.000	NA	0.000	0.000	NA	0.000	NA	NA	0.000	0.000	NA	0.040	0.000	0.000	0.000	0.027	0.000	NA
rs543090783	A	0.000	NA	NA	NA	NA	NA	0.000	NA	NA	NA	0.006	NA	0.000	0.000	NA	0.000	NA	NA	0.000	0.000	NA	0.000	0.000	0.000	0.000	0.000	0.000	NA
rs540356623	A	0.000	NA	NA	NA	NA	NA	0.000	NA	NA	NA	0.000	NA	0.000	0.000	NA	0.000	NA	NA	0.000	0.000	NA	0.000	0.000	0.000	0.000	0.009	0.000	NA
rs564651283	T	0.000	NA	NA	NA	NA	NA	0.000	NA	NA	NA	0.000	NA	0.000	0.000	NA	0.000	NA	NA	0.000	0.000	NA	0.005	0.000	0.000	0.000	0.000	0.000	NA
rs12609610	G	0.000	0.000	0.000	0.000	0.000	0.000	0.000	0.000	0.000	0.000	0.012	0.031	0.022	0.000	0.000	0.039	NA	0.000	0.029	0.000	0.000	0.000	0.000	0.000	0.000	0.000	0.000	0.000
rs572636722	T	0.000	NA	NA	NA	NA	NA	0.000	NA	NA	NA	0.000	NA	0.000	0.000	NA	0.000	NA	NA	0.000	0.000	NA	0.000	0.000	0.000	0.000	0.000	0.000	NA
rs10416339	A	0.307	NA	NA	NA	NA	NA	0.443	NA	NA	NA	0.571	NA	0.495	0.737	NA	0.534	0	NA	0.476	0.537	NA	0.308	0.748	0.692	0.471	0.376	0.636	NA
rs56067110	A	0.031	NA	NA	NA	NA	NA	0.008	NA	NA	NA	0.000	NA	0.000	0.000	NA	0.000	NA	NA	0.000	0.000	NA	0.030	0.000	0.000	0.000	0.022	0.000	NA
rs552690398	A	0.000	NA	NA	NA	NA	NA	0.000	NA	NA	NA	0.000	NA	0.000	0.000	NA	0.000	NA	NA	0.000	0.000	NA	0.000	0.000	0.000	0.000	0.000	0.000	NA
rs72972182	C	0.005	NA	NA	NA	NA	NA	0.016	NA	NA	NA	0.070	NA	0.032	0.066	NA	0.024	0.7143	NA	0.024	0.160	NA	0.000	0.066	0.066	0.117	0.009	0.079	NA
rs143454800	G	0.000	NA	NA	NA	NA	NA	0.000	NA	NA	NA	0.000	NA	0.011	0.000	NA	0.019	NA	NA	0.033	0.000	NA	0.000	0.000	0.000	0.000	0.000	0.000	NA
rs539563939	A	0.000	NA	NA	NA	NA	NA	0.000	NA	NA	NA	0.000	NA	0.000	0.000	NA	0.000	NA	NA	0.000	0.000	NA	0.000	0.000	0.000	0.000	0.000	0.000	NA
rs368584754	A	0.016	NA	NA	NA	NA	NA	0.008	NA	NA	NA	0.000	NA	0.000	0.000	NA	0.000	NA	NA	0.000	0.005	NA	0.000	0.000	0.000	0.000	0.004	0.000	NA
rs529198553	A	0.005	NA	NA	NA	NA	NA	0.000	NA	NA	NA	0.000	NA	0.000	0.000	NA	0.000	NA	NA	0.000	0.000	NA	0.000	0.000	0.000	0.000	0.000	0.000	NA
rs557853268	A	0.000	NA	NA	NA	NA	NA	0.000	NA	NA	NA	0.000	NA	0.000	0.000	NA	0.000	NA	NA	0.000	0.000	NA	0.000	0.000	0.000	0.000	0.000	0.000	NA
rs533240475	A	0.000	NA	NA	NA	NA	NA	0.000	NA	NA	NA	0.000	NA	0.000	0.000	NA	0.000	NA	NA	0.000	0.000	NA	0.000	0.000	0.000	0.000	0.000	0.000	NA
rs202082105	A	0.000	NA	NA	NA	NA	NA	0.000	NA	NA	NA	0.000	NA	0.000	0.000	NA	0.000	NA	NA	0.000	0.000	NA	0.000	0.000	0.000	0.000	0.000	0.000	NA

bold: functionally relevant SNPs found in our databases

Table S3-A – BSG allele frequencies continuation. Bold = functionally relevant SNPs found in our databases; NA = missing data

SNP	Allele	ITU	Jacarus	JPT	KHV	Lamas	Lambayeque	Lima	LWK	Matses	Moche	Moquegua	MSL	MXL	PEL	Pelotas	PJL	Puno	PUR	Qeros	Quechuas	Salvador	Shimaa
**rs8259**	**T**	**NA**	**NA**	**NA**	**NA**	**NA**	**NA**	**NA**	**NA**	**NA**	**NA**	**NA**	**NA**	**NA**	**NA**	**0.401**	**NA**	**NA**	**NA**	**NA**	**NA**	**0.483**	**NA**
**rs6757**	**C**	**0.284**	**0.000**	**0.125**	**0.288**	**0.071**	**0.083**	**0.071**	**0.167**	**0.045**	**0.050**	**0.034**	**0.247**	**0.078**	**0.041**	**0.082**	**0.182**	**0.025**	**0.087**	**0.000**	**0.042**	**0.178**	**0.152**
**rs8637**	**A**	**0.196**	**NA**	**0.284**	**0.177**	**NA**	**NA**	**NA**	**0.025**	**NA**	**NA**	**NA**	**0.000**	**0.281**	**0.235**	**NA**	**0.156**	**NA**	**0.423**	**NA**	**NA**	**NA**	**NA**
**rs12608994**	**T**	**0.284**	**NA**	**0.125**	**0.293**	**NA**	**NA**	**NA**	**0.177**	**NA**	**NA**	**NA**	**0.259**	**0.094**	**0.041**	**0.099**	**0.203**	**NA**	**0.115**	**NA**	**NA**	**0.189**	**NA**
**rs1803202**	**T**	**0.284**	**NA**	**0.125**	**0.288**	**NA**	**NA**	**NA**	**0.167**	**NA**	**NA**	**NA**	**0.253**	**0.094**	**0.041**	**0.090**	**0.203**	**NA**	**0.115**	**NA**	**NA**	**0.189**	**NA**
**rs11879069**	**A**	**0.358**	**0.353**	**0.500**	**0.465**	**0.381**	**0.364**	**0.393**	**0.374**	**0.273**	**0.466**	**0.345**	**0.300**	**0.406**	**0.353**	**0.291**	**0.396**	**0.388**	**0.226**	**0.542**	**0.326**	**0.275**	**0.217**
**rs4919862**	**T**	**0.078**	**NA**	**0.240**	**0.152**	**NA**	**NA**	**NA**	**0.015**	**NA**	**NA**	**NA**	**0.000**	**0.172**	**0.153**	**NA**	**0.068**	**NA**	**0.284**	**NA**	**NA**	**NA**	**NA**
**rs6758**	**A**	**0.279**	**NA**	**0.125**	**0.242**	**NA**	**NA**	**NA**	**0.172**	**NA**	**NA**	**NA**	**0.247**	**0.078**	**0.041**	**NA**	**0.182**	**NA**	**0.087**	**NA**	**NA**	**NA**	**NA**
**rs28992474**	**T**	**0.284**	**NA**	**0.125**	**0.298**	**NA**	**NA**	**NA**	**0.172**	**NA**	**NA**	**NA**	**0.247**	**0.094**	**0.041**	**NA**	**0.203**	**NA**	**0.115**	**NA**	**NA**	**NA**	**NA**
**rs2072310**	**T**	**0.358**	**NA**	**0.500**	**0.460**	**NA**	**NA**	**NA**	**0.374**	**NA**	**NA**	**NA**	**0.294**	**0.375**	**0.347**	**NA**	**0.391**	**NA**	**0.216**	**NA**	**NA**	**NA**	**NA**
**rs3764937**	**A**	**0.304**	**NA**	**0.130**	**0.308**	**NA**	**NA**	**NA**	**0.308**	**NA**	**NA**	**NA**	**0.412**	**0.094**	**0.041**	**NA**	**0.245**	**NA**	**0.130**	**NA**	**NA**	**NA**	**NA**
**rs4682**	**C**	**0.358**	**NA**	**0.500**	**0.465**	**NA**	**NA**	**NA**	**0.374**	**NA**	**NA**	**NA**	**0.300**	**0.406**	**0.353**	**NA**	**0.396**	**NA**	**0.221**	**NA**	**NA**	**NA**	**NA**
**rs2072309**	**A**	**0.333**	**0.353**	**0.495**	**0.450**	**0.381**	**0.375**	**0.393**	**0.318**	**0.273**	**0.467**	**0.345**	**0.288**	**0.398**	**0.359**	**0.297**	**0.354**	**0.397**	**0.212**	**0.542**	**0.370**	**0.272**	**0.217**
**rs2074962**	**A**	**0.206**	**NA**	**0.293**	**0.182**	**NA**	**NA**	**NA**	**0.101**	**NA**	**NA**	**NA**	**0.012**	**0.289**	**0.253**	**NA**	**0.245**	**NA**	**0.457**	**NA**	**NA**	**NA**	**NA**
rs568500243	T	0.000	NA	0.000	0.000	NA	NA	NA	0.000	NA	NA	NA	0.000	0.000	0.000	NA	0.000	NA	0.000	NA	NA	NA	NA
rs565860293	A	0.000	NA	0.000	0.000	NA	NA	NA	0.000	NA	NA	NA	0.000	0.000	0.000	NA	0.000	NA	0.000	NA	NA	NA	NA
rs369456038	T	0.000	NA	0.000	0.000	NA	NA	NA	0.000	NA	NA	NA	0.000	0.000	0.000	NA	0.000	NA	0.000	NA	NA	NA	NA
rs2108832	C	NA	NA	NA	NA	NA	NA	NA	NA	NA	NA	NA	NA	NA	NA	0.293	NA	NA	NA	NA	NA	0.283	NA
rs193268910	T	0.000	NA	0.000	0.000	NA	NA	NA	0.010	NA	NA	NA	0.006	0.000	0.000	NA	0.000	NA	0.000	NA	NA	NA	NA
rs576903171	A	0.000	NA	0.000	0.000	NA	NA	NA	0.000	NA	NA	NA	0.000	0.000	0.000	NA	0.000	NA	0.000	NA	NA	NA	NA
rs376295294	A	0.000	NA	0.000	0.000	NA	NA	NA	0.000	NA	NA	NA	0.000	0.000	0.000	NA	0.000	NA	0.000	NA	NA	NA	NA
rs188261956	A	0.000	NA	0.000	0.000	NA	NA	NA	0.015	NA	NA	NA	0.000	0.000	0.006	NA	0.000	NA	0.005	NA	NA	NA	NA
rs140055452	T	0.000	NA	0.000	0.000	NA	NA	NA	0.015	NA	NA	NA	0.006	0.000	0.000	NA	0.000	NA	0.000	NA	NA	NA	NA
rs552833814	T	0.000	NA	0.000	0.000	NA	NA	NA	0.000	NA	NA	NA	0.000	0.000	0.000	NA	0.000	NA	0.000	NA	NA	NA	NA
rs531651313	A	0.000	NA	0.000	0.000	NA	NA	NA	0.000	NA	NA	NA	0.000	0.000	0.000	NA	0.000	NA	0.000	NA	NA	NA	NA
rs551644758	G	0.000	NA	0.000	0.000	NA	NA	NA	0.000	NA	NA	NA	0.000	0.000	0.000	NA	0.000	NA	0.000	NA	NA	NA	NA
rs2238540	T	0.284	NA	0.125	0.298	NA	NA	NA	0.167	NA	NA	NA	0.247	0.094	0.041	0.098	0.203	NA	0.115	NA	NA	0.189	NA
rs189460104	A	0.000	NA	0.000	0.000	NA	NA	NA	0.015	NA	NA	NA	0.041	0.000	0.000	NA	0.000	NA	0.000	NA	NA	NA	NA
rs118187381	A	0.000	NA	0.000	0.000	NA	NA	NA	0.000	NA	NA	NA	0.000	0.008	0.000	0.006	0.000	NA	0.000	NA	NA	0.000	NA
rs183979701	G	0.000	NA	0.000	0.000	NA	NA	NA	0.000	NA	NA	NA	0.000	0.008	0.124	NA	0.000	NA	0.014	NA	NA	NA	NA
rs10593023	C	0.358	NA	0.500	0.460	NA	NA	NA	0.374	NA	NA	NA	0.300	0.391	0.347	NA	0.396	NA	0.221	NA	NA	NA	NA
rs561149009	A	0.000	NA	0.000	0.000	NA	NA	NA	0.000	NA	NA	NA	0.000	0.000	0.000	NA	0.000	NA	0.005	NA	NA	NA	NA
rs543884178	T	0.000	NA	0.000	0.000	NA	NA	NA	0.000	NA	NA	NA	0.000	0.000	0.000	NA	0.005	NA	0.000	NA	NA	NA	NA
rs544074414	A	0.000	NA	0.000	0.000	NA	NA	NA	0.000	NA	NA	NA	0.000	0.000	0.000	NA	0.000	NA	0.000	NA	NA	NA	NA
rs551225501	G	0.000	NA	0.000	0.000	NA	NA	NA	0.035	NA	NA	NA	0.076	0.000	0.000	NA	0.000	NA	0.000	NA	NA	NA	NA
rs28915407	T	0.005	NA	0.000	0.000	NA	NA	NA	0.000	NA	NA	NA	0.000	0.008	0.000	NA	0.005	NA	0.019	NA	NA	NA	NA
rs28992486	G	0.000	NA	0.000	0.000	NA	NA	NA	0.121	NA	NA	NA	0.165	0.000	0.000	NA	0.000	NA	0.019	NA	NA	NA	NA
rs375283966	C	0.000	NA	0.000	0.000	NA	NA	NA	0.000	NA	NA	NA	0.000	0.000	0.000	NA	0.000	NA	0.000	NA	NA	NA	NA
rs2283572	T	0.358	NA	0.500	0.460	NA	NA	NA	0.379	NA	NA	NA	0.300	0.406	0.353	NA	0.396	NA	0.226	NA	NA	NA	NA
rs12609063	A	0.284	NA	0.125	0.298	NA	NA	NA	0.172	NA	NA	NA	0.247	0.094	0.041	NA	0.203	NA	0.115	NA	NA	NA	NA
rs528163900	A	0.000	NA	0.000	0.000	NA	NA	NA	0.005	NA	NA	NA	0.000	0.000	0.000	NA	0.000	NA	0.000	NA	NA	NA	NA
rs188930171	C	0.000	NA	0.014	0.010	NA	NA	NA	0.000	NA	NA	NA	0.000	0.000	0.000	NA	0.000	NA	0.000	NA	NA	NA	NA

SNP	Allele	ITU	Jacarus	JPT	KHV	Lamas	Lambayeque	Lima	LWK	Matses	Moche	Moquegua	MSL	MXL	PEL	Pelotas	PJL	Puno	PUR	Qeros	Quechuas	Salvador	Shimaa
rs149894645	C	0.000	NA	0.000	0.000	NA	NA	NA	0.005	NA	NA	NA	0.000	0.000	0.000	NA	0.000	NA	0.000	NA	NA	NA	NA
rs558467385	A	0.000	NA	0.000	0.000	NA	NA	NA	0.000	NA	NA	NA	0.000	0.000	0.000	NA	0.000	NA	0.000	NA	NA	NA	NA
rs542497048	T	0.000	NA	0.000	0.000	NA	NA	NA	0.000	NA	NA	NA	0.000	0.000	0.000	NA	0.000	NA	0.000	NA	NA	NA	NA
rs547801807	C	0.000	NA	0.000	0.000	NA	NA	NA	0.015	NA	NA	NA	0.012	0.000	0.000	NA	0.000	NA	0.000	NA	NA	NA	NA
rs371927933	T	0.000	NA	0.000	0.005	NA	NA	NA	0.000	NA	NA	NA	0.000	0.000	0.000	NA	0.000	NA	0.000	NA	NA	NA	NA
rs556218809	A	0.000	NA	0.000	0.000	NA	NA	NA	0.000	NA	NA	NA	0.000	0.000	0.000	NA	0.000	NA	0.000	NA	NA	NA	NA
rs1138150	T	0.529	NA	0.418	0.480	NA	NA	NA	0.232	NA	NA	NA	0.235	0.383	0.294	NA	0.453	NA	0.567	NA	NA	NA	NA
rs28921974	C	0.093	NA	0.014	0.025	NA	NA	NA	0.121	NA	NA	NA	0.035	0.031	0.012	0.052	0.146	NA	0.063	NA	NA	0.050	NA
rs568364520	A	0.005	NA	0.000	0.000	NA	NA	NA	0.000	NA	NA	NA	0.000	0.000	0.000	NA	0.000	NA	0.000	NA	NA	NA	NA
rs2283573	T	0.284	NA	0.125	0.298	NA	NA	NA	0.172	NA	NA	NA	0.247	0.094	0.041	NA	0.203	NA	0.115	NA	NA	NA	NA
rs562293196	A	0.000	NA	0.000	0.000	NA	NA	NA	0.005	NA	NA	NA	0.000	0.000	0.000	NA	0.000	NA	0.000	NA	NA	NA	NA
rs11458913	C	0.245	NA	0.293	0.182	NA	NA	NA	0.096	NA	NA	NA	0.024	0.289	0.253	NA	0.250	NA	0.447	NA	NA	NA	NA
rs114359237	T	0.000	NA	0.000	0.000	NA	NA	NA	0.000	NA	NA	NA	0.012	0.000	0.000	NA	0.000	NA	0.010	NA	NA	NA	NA
rs28989774	A	0.000	NA	0.000	0.000	NA	NA	NA	0.091	NA	NA	NA	0.106	0.000	0.000	NA	0.000	NA	0.014	NA	NA	NA	NA
rs189315508	A	0.000	NA	0.000	0.000	NA	NA	NA	0.000	NA	NA	NA	0.000	0.000	0.000	NA	0.000	NA	0.000	NA	NA	NA	NA
rs138409636	T	0.000	NA	0.000	0.000	NA	NA	NA	0.005	NA	NA	NA	0.000	0.000	0.000	NA	0.000	NA	0.000	NA	NA	NA	NA
rs375925694	T	0.000	NA	0.000	0.015	NA	NA	NA	0.000	NA	NA	NA	0.000	0.000	0.000	NA	0.000	NA	0.000	NA	NA	NA	NA
rs567062140	A	0.000	NA	0.000	0.000	NA	NA	NA	0.000	NA	NA	NA	0.000	0.008	0.000	NA	0.000	NA	0.000	NA	NA	NA	NA
rs574441118	A	0.000	NA	0.000	0.000	NA	NA	NA	0.000	NA	NA	NA	0.000	0.008	0.000	NA	0.000	NA	0.000	NA	NA	NA	NA
rs1049676	T	0.039	NA	0.000	0.000	NA	NA	NA	0.040	NA	NA	NA	0.000	0.008	0.012	0.029	0.078	NA	0.014	NA	NA	0.039	NA
rs183656586	A	0.000	NA	0.000	0.000	NA	NA	NA	0.000	NA	NA	NA	0.000	0.000	0.000	NA	0.000	NA	0.000	NA	NA	NA	NA
rs574480082	G	0.000	NA	0.000	0.000	NA	NA	NA	0.000	NA	NA	NA	0.000	0.000	0.000	NA	0.000	NA	0.000	NA	NA	NA	NA
rs565755933	T	0.000	NA	0.000	0.000	NA	NA	NA	0.000	NA	NA	NA	0.000	0.016	0.000	NA	0.000	NA	0.014	NA	NA	NA	NA
rs554979181	A	0.000	NA	0.000	0.000	NA	NA	NA	0.000	NA	NA	NA	0.000	0.000	0.000	NA	0.000	NA	0.000	NA	NA	NA	NA
rs146784114	A	NA	NA	NA	NA	NA	NA	NA	NA	NA	NA	NA	NA	NA	NA	NA	NA	NA	NA	NA	NA	NA	NA
rs564593406	A	0.000	NA	0.000	0.000	NA	NA	NA	0.000	NA	NA	NA	0.000	0.000	0.000	NA	0.000	NA	0.000	NA	NA	NA	NA
rs535528596	T	0.000	NA	0.000	0.000	NA	NA	NA	0.000	NA	NA	NA	0.000	0.000	0.000	NA	0.000	NA	0.000	NA	NA	NA	NA
rs185996213	C	0.010	NA	0.000	0.005	NA	NA	NA	0.005	NA	NA	NA	0.000	0.000	0.000	NA	0.010	NA	0.010	NA	NA	NA	NA
rs185382187	A	0.000	NA	0.000	0.000	NA	NA	NA	0.000	NA	NA	NA	0.000	0.000	0.000	NA	0.000	NA	0.000	NA	NA	NA	NA
rs2072308	C	0.284	NA	0.082	0.222	NA	NA	NA	0.187	NA	NA	NA	0.247	0.086	0.041	NA	0.203	NA	0.120	NA	NA	NA	NA
rs141043764	A	0.000	NA	0.000	0.000	NA	NA	NA	0.010	NA	NA	NA	0.006	0.000	0.000	NA	0.000	NA	0.000	NA	NA	NA	NA
rs185250119	A	0.000	NA	0.000	0.035	NA	NA	NA	0.000	NA	NA	NA	0.000	0.000	0.000	NA	0.000	NA	0.000	NA	NA	NA	NA
rs147511542	C	0.000	NA	0.014	0.000	NA	NA	NA	0.000	NA	NA	NA	0.000	0.000	0.000	NA	0.000	NA	0.000	NA	NA	NA	NA
rs573330257	G	0.000	NA	0.000	0.000	NA	NA	NA	0.000	NA	NA	NA	0.000	0.000	0.000	NA	0.000	NA	0.000	NA	NA	NA	NA
rs542924300	T	0.005	NA	0.000	0.000	NA	NA	NA	0.000	NA	NA	NA	0.000	0.000	0.000	NA	0.000	NA	0.000	NA	NA	NA	NA
rs543561666	A	0.000	NA	0.000	0.005	NA	NA	NA	0.005	NA	NA	NA	0.000	0.000	0.000	NA	0.000	NA	0.000	NA	NA	NA	NA
rs8110980	T	0.358	0.324	0.500	0.460	0.194	0.375	0.389	0.374	0.273	0.467	0.328	0.300	0.391	0.347	0.292	0.396	0.375	0.221	0.458	0.370	0.283	0.217
rs200045153	G	0.005	NA	0.000	0.000	NA	NA	NA	0.000	NA	NA	NA	0.000	0.000	0.006	NA	0.005	NA	0.010	NA	NA	NA	NA
rs10422916	T	0.196	NA	0.293	0.177	NA	NA	NA	0.035	NA	NA	NA	0.006	0.281	0.241	NA	0.162	NA	0.418	NA	NA	NA	NA
rs568226979	T	0.000	NA	0.000	0.000	NA	NA	NA	0.000	NA	NA	NA	0.000	0.000	0.000	NA	0.000	NA	0.000	NA	NA	NA	NA
rs550149267	T	0.000	NA	0.000	0.000	NA	NA	NA	0.000	NA	NA	NA	0.000	0.000	0.000	NA	0.000	NA	0.000	NA	NA	NA	NA
rs182848936	T	0.000	NA	0.000	0.000	NA	NA	NA	0.000	NA	NA	NA	0.000	0.000	0.000	NA	0.000	NA	0.000	NA	NA	NA	NA
rs535528658	T	0.000	NA	0.000	0.000	NA	NA	NA	0.000	NA	NA	NA	0.000	0.000	0.000	NA	0.000	NA	0.000	NA	NA	NA	NA

SNP	Allele	ITU	Jacarus	JPT	KHV	Lamas	Lambayeque	Lima	LWK	Matses	Moche	Moquegua	MSL	MXL	PEL	Pelotas	PJL	Puno	PUR	Qeros	Quechuas	Salvador	Shimaa
rs537007790	T	0.000	NA	0.000	0.000	NA	NA	NA	0.000	NA	NA	NA	0.000	0.000	0.000	NA	0.000	NA	0.000	NA	NA	NA	NA
rs571095325	A	0.000	NA	0.000	0.000	NA	NA	NA	0.000	NA	NA	NA	0.024	0.008	0.000	NA	0.000	NA	0.000	NA	NA	NA	NA
rs548874736	T	0.000	NA	0.005	0.000	NA	NA	NA	0.015	NA	NA	NA	0.000	0.000	0.000	NA	0.000	NA	0.000	NA	NA	NA	NA
rs546527955	T	0.000	NA	0.000	0.000	NA	NA	NA	0.000	NA	NA	NA	0.000	0.000	0.000	NA	0.000	NA	0.000	NA	NA	NA	NA
rs533122473	T	0.000	NA	0.000	0.000	NA	NA	NA	0.000	NA	NA	NA	0.000	0.000	0.000	NA	0.000	NA	0.000	NA	NA	NA	NA
rs28992481	C	0.000	NA	0.000	0.000	NA	NA	NA	0.005	NA	NA	NA	0.012	0.000	0.000	NA	0.000	NA	0.000	NA	NA	NA	NA
rs547511336	A	0.000	NA	0.000	0.000	NA	NA	NA	0.000	NA	NA	NA	0.000	0.000	0.000	NA	0.005	NA	0.000	NA	NA	NA	NA
rs117305685	T	0.005	NA	0.000	0.000	NA	NA	NA	0.000	NA	NA	NA	0.000	0.000	0.000	0.000	0.000	NA	0.000	NA	NA	0.000	NA
rs28921987	A	0.000	NA	0.000	0.000	NA	NA	NA	0.000	NA	NA	NA	0.000	0.000	0.000	NA	0.000	NA	0.010	NA	NA	NA	NA
rs60851035	A	0.000	NA	0.000	0.000	NA	NA	NA	0.040	NA	NA	NA	0.076	0.000	0.000	NA	0.000	NA	0.005	NA	NA	NA	NA
rs570887990	T	0.000	NA	0.000	0.005	NA	NA	NA	0.000	NA	NA	NA	0.000	0.000	0.000	NA	0.000	NA	0.000	NA	NA	NA	NA
rs556084263	T	0.000	NA	0.000	0.000	NA	NA	NA	0.000	NA	NA	NA	0.006	0.000	0.000	NA	0.000	NA	0.000	NA	NA	NA	NA
rs142283391	T	0.000	NA	0.000	0.000	NA	NA	NA	0.005	NA	NA	NA	0.000	0.000	0.000	NA	0.000	NA	0.000	NA	NA	NA	NA
rs375240640	A	0.074	NA	0.005	0.015	NA	NA	NA	0.040	NA	NA	NA	0.071	0.133	0.312	NA	0.125	NA	0.101	NA	NA	NA	NA
rs114264435	T	0.000	NA	0.000	0.000	NA	NA	NA	0.030	NA	NA	NA	0.041	0.000	0.000	NA	0.000	NA	0.000	NA	NA	NA	NA
rs553170407	C	0.000	NA	0.000	0.000	NA	NA	NA	0.000	NA	NA	NA	0.000	0.000	0.000	NA	0.005	NA	0.000	NA	NA	NA	NA
rs541302606	A	0.005	NA	0.000	0.000	NA	NA	NA	0.000	NA	NA	NA	0.000	0.000	0.000	NA	0.000	NA	0.000	NA	NA	NA	NA
rs529379861	A	0.000	NA	0.005	0.010	NA	NA	NA	0.000	NA	NA	NA	0.000	0.000	0.000	NA	0.000	NA	0.000	NA	NA	NA	NA
rs112152563	T	NA	NA	NA	NA	NA	NA	NA	NA	NA	NA	NA	NA	NA	NA	NA	NA	NA	NA	NA	NA	NA	NA
rs557135170	A	0.000	NA	0.000	0.000	NA	NA	NA	0.000	NA	NA	NA	0.000	0.000	0.000	NA	0.000	NA	0.000	NA	NA	NA	NA
rs557199346	A	0.000	NA	0.000	0.000	NA	NA	NA	0.000	NA	NA	NA	0.006	0.000	0.000	NA	0.000	NA	0.000	NA	NA	NA	NA
rs14173	G	0.000	NA	0.000	0.005	NA	NA	NA	0.015	NA	NA	NA	0.006	0.000	0.000	NA	0.000	NA	0.000	NA	NA	NA	NA
rs531691645	A	0.000	NA	0.000	0.000	NA	NA	NA	0.000	NA	NA	NA	0.000	0.000	0.000	NA	0.000	NA	0.000	NA	NA	NA	NA
rs376756373	A	0.000	NA	0.000	0.000	NA	NA	NA	0.000	NA	NA	NA	0.000	0.000	0.024	NA	0.000	NA	0.000	NA	NA	NA	NA
rs7253615	G	0.245	NA	0.293	0.182	NA	NA	NA	0.101	NA	NA	NA	0.012	0.289	0.253	NA	0.250	NA	0.447	NA	NA	NA	NA
rs566889863	C	0.000	NA	0.000	0.000	NA	NA	NA	0.000	NA	NA	NA	0.000	0.000	0.000	NA	0.000	NA	0.000	NA	NA	NA	NA
rs369996586	C	0.000	NA	0.000	0.000	NA	NA	NA	0.000	NA	NA	NA	0.000	0.000	0.000	NA	0.000	NA	0.000	NA	NA	NA	NA
rs200704727	CA	0.284	NA	0.125	0.298	NA	NA	NA	0.167	NA	NA	NA	0.247	0.094	0.041	NA	0.203	NA	0.115	NA	NA	NA	NA
rs539709135	G	0.363	NA	0.034	0.040	NA	NA	NA	0.131	NA	NA	NA	0.429	0.094	0.082	NA	0.406	NA	0.082	NA	NA	NA	NA
rs28921981	A	0.142	NA	0.053	0.081	NA	NA	NA	0.056	NA	NA	NA	0.129	0.031	0.035	NA	0.115	NA	0.024	NA	NA	NA	NA
rs7252521	T	0.000	NA	0.000	0.000	NA	NA	NA	0.000	NA	NA	NA	0.006	0.000	0.000	NA	0.000	NA	0.000	NA	NA	NA	NA
rs562167455	G	0.000	NA	0.000	0.000	NA	NA	NA	0.000	NA	NA	NA	0.000	0.000	0.000	NA	0.000	NA	0.000	NA	NA	NA	NA
rs539126301	A	0.000	NA	0.005	0.000	NA	NA	NA	0.000	NA	NA	NA	0.000	0.000	0.000	NA	0.000	NA	0.000	NA	NA	NA	NA
rs570527862	G	0.000	NA	0.000	0.000	NA	NA	NA	0.000	NA	NA	NA	0.012	0.000	0.000	NA	0.000	NA	0.000	NA	NA	NA	NA
rs577273577	T	0.000	NA	0.000	0.000	NA	NA	NA	0.000	NA	NA	NA	0.000	0.000	0.000	NA	0.000	NA	0.000	NA	NA	NA	NA
rs187186896	G	0.000	NA	0.000	0.000	NA	NA	NA	0.000	NA	NA	NA	0.000	0.000	0.000	NA	0.000	NA	0.005	NA	NA	NA	NA
rs11551906	G	0.000	NA	0.000	0.000	NA	NA	NA	0.000	NA	NA	NA	0.000	0.008	0.000	NA	0.010	NA	0.005	NA	NA	NA	NA
rs576653835	A	0.000	NA	0.000	0.000	NA	NA	NA	0.000	NA	NA	NA	0.006	0.000	0.000	NA	0.000	NA	0.000	NA	NA	NA	NA
rs141694906	A	0.000	NA	0.005	0.000	NA	NA	NA	0.000	NA	NA	NA	0.000	0.000	0.000	NA	0.000	NA	0.000	NA	NA	NA	NA
rs140396239	A	0.000	NA	0.000	0.000	NA	NA	NA	0.010	NA	NA	NA	0.000	0.000	0.000	NA	0.000	NA	0.000	NA	NA	NA	NA
rs142446858	T	NA	NA	NA	NA	NA	NA	NA	NA	NA	NA	NA	NA	NA	NA	NA	NA	NA	NA	NA	NA	NA	NA
rs561158080	T	0.000	NA	0.000	0.000	NA	NA	NA	0.000	NA	NA	NA	0.000	0.000	0.000	NA	0.000	NA	0.000	NA	NA	NA	NA
rs2238543	T	0.196	NA	0.284	0.172	NA	NA	NA	0.035	NA	NA	NA	0.006	0.297	0.247	NA	0.162	NA	0.423	NA	NA	NA	NA

SNP	Allele	ITU	Jacarus	JPT	KHV	Lamas	Lambayeque	Lima	LWK	Matses	Moche	Moquegua	MSL	MXL	PEL	Pelotas	PJL	Puno	PUR	Qeros	Quechuas	Salvador	Shimaa
rs554135380	T	0.000	NA	0.010	0.000	NA	NA	NA	0.000	NA	NA	NA	0.000	0.000	0.000	NA	0.000	NA	0.000	NA	NA	NA	NA
rs543916508	A	0.000	NA	0.010	0.000	NA	NA	NA	0.000	NA	NA	NA	0.000	0.000	0.000	NA	0.000	NA	0.000	NA	NA	NA	NA
rs551307834	T	0.000	NA	0.000	0.000	NA	NA	NA	0.000	NA	NA	NA	0.006	0.000	0.000	NA	0.000	NA	0.000	NA	NA	NA	NA
rs146061693	A	0.000	NA	0.000	0.000	NA	NA	NA	0.020	NA	NA	NA	0.029	0.000	0.000	NA	0.000	NA	0.000	NA	NA	NA	NA
rs559328258	T	0.000	NA	0.000	0.000	NA	NA	NA	0.000	NA	NA	NA	0.000	0.000	0.000	NA	0.000	NA	0.014	NA	NA	NA	NA
rs545139311	A	0.000	NA	0.000	0.000	NA	NA	NA	0.000	NA	NA	NA	0.000	0.000	0.000	NA	0.000	NA	0.000	NA	NA	NA	NA
rs572647656	T	0.000	NA	0.000	0.000	NA	NA	NA	0.000	NA	NA	NA	0.000	0.000	0.000	NA	0.000	NA	0.000	NA	NA	NA	NA
rs372445442	A	0.000	NA	0.005	0.000	NA	NA	NA	0.000	NA	NA	NA	0.000	0.000	0.000	NA	0.000	NA	0.000	NA	NA	NA	NA
rs190259313	A	0.000	NA	0.014	0.040	NA	NA	NA	0.000	NA	NA	NA	0.000	0.000	0.000	NA	0.000	NA	0.000	NA	NA	NA	NA
rs561243171	T	0.000	NA	0.005	0.000	NA	NA	NA	0.091	NA	NA	NA	0.053	0.000	0.012	NA	0.000	NA	0.010	NA	NA	NA	NA
rs138600800	C	0.000	NA	0.000	0.000	NA	NA	NA	0.000	NA	NA	NA	0.000	0.000	0.000	0.006	0.000	NA	0.010	NA	NA	0.006	NA
rs28921977	G	0.005	NA	0.000	0.000	NA	NA	NA	0.020	NA	NA	NA	0.012	0.047	0.024	NA	0.010	NA	0.067	NA	NA	NA	NA
rs571072379	T	0.000	NA	0.000	0.000	NA	NA	NA	0.000	NA	NA	NA	0.000	0.000	0.000	NA	0.000	NA	0.005	NA	NA	NA	NA
rs375819042	T	0.000	NA	0.000	0.010	NA	NA	NA	0.000	NA	NA	NA	0.000	0.000	0.000	NA	0.000	NA	0.000	NA	NA	NA	NA
rs41276870	T	0.000	NA	0.000	0.000	NA	NA	NA	0.000	NA	NA	NA	0.000	0.000	0.000	NA	0.000	NA	0.000	NA	NA	NA	NA
rs567156454	C	0.000	NA	0.005	0.000	NA	NA	NA	0.000	NA	NA	NA	0.000	0.000	0.000	NA	0.000	NA	0.000	NA	NA	NA	NA
rs539842435	T	0.000	NA	0.000	0.000	NA	NA	NA	0.000	NA	NA	NA	0.000	0.000	0.000	NA	0.005	NA	0.005	NA	NA	NA	NA
rs529739382	G	0.000	NA	0.000	0.000	NA	NA	NA	0.000	NA	NA	NA	0.018	0.000	0.000	NA	0.000	NA	0.000	NA	NA	NA	NA
rs2041192	A	0.245	0.235	0.293	0.182	0.050	0.208	0.232	0.091	0.300	0.100	0.328	0.006	0.289	0.253	NA	0.250	0.300	0.447	0.042	0.313	NA	0.239
rs565127256	A	0.005	NA	0.000	0.000	NA	NA	NA	0.000	NA	NA	NA	0.000	0.000	0.000	NA	0.005	NA	0.000	NA	NA	NA	NA
rs561885264	T	0.000	NA	0.000	0.000	NA	NA	NA	0.000	NA	NA	NA	0.000	0.000	0.000	NA	0.000	NA	0.000	NA	NA	NA	NA
rs111866858	A	0.000	NA	0.000	0.000	NA	NA	NA	0.051	NA	NA	NA	0.129	0.008	0.000	NA	0.000	NA	0.019	NA	NA	NA	NA
rs141476449	T	0.284	NA	0.082	0.237	NA	NA	NA	0.172	NA	NA	NA	0.241	0.094	0.041	NA	0.203	NA	0.115	NA	NA	NA	NA
rs144233497	A	0.000	NA	0.005	0.000	NA	NA	NA	0.000	NA	NA	NA	0.000	0.000	0.000	NA	0.000	NA	0.000	NA	NA	NA	NA
rs183270957	T	0.000	NA	0.000	0.000	NA	NA	NA	0.005	NA	NA	NA	0.000	0.000	0.000	NA	0.000	NA	0.000	NA	NA	NA	NA
rs554359587	T	0.000	NA	0.000	0.000	NA	NA	NA	0.000	NA	NA	NA	0.000	0.000	0.000	NA	0.000	NA	0.000	NA	NA	NA	NA
rs55713331	T	0.039	NA	0.000	0.000	NA	NA	NA	0.000	NA	NA	NA	0.000	0.000	0.000	NA	0.036	NA	0.000	NA	NA	NA	NA
rs367873477	C	0.039	NA	0.000	0.000	NA	NA	NA	0.000	NA	NA	NA	0.000	0.000	0.000	NA	0.036	NA	0.000	NA	NA	NA	NA
rs576495399	T	0.000	NA	0.000	0.000	NA	NA	NA	0.000	NA	NA	NA	0.000	0.000	0.000	NA	0.000	NA	0.000	NA	NA	NA	NA
rs564880752	A	0.000	NA	0.000	0.000	NA	NA	NA	0.000	NA	NA	NA	0.000	0.000	0.000	NA	0.005	NA	0.000	NA	NA	NA	NA
rs549998110	T	0.000	NA	0.000	0.000	NA	NA	NA	0.010	NA	NA	NA	0.000	0.000	0.000	NA	0.000	NA	0.000	NA	NA	NA	NA
rs370592787	T	0.000	NA	0.000	0.000	NA	NA	NA	0.015	NA	NA	NA	0.041	0.000	0.000	NA	0.000	NA	0.000	NA	NA	NA	NA
rs12609912	T	0.284	NA	0.125	0.298	NA	NA	NA	0.172	NA	NA	NA	0.247	0.094	0.041	0.098	0.203	NA	0.115	NA	NA	0.189	NA
rs138950962	A	0.000	NA	0.005	0.000	NA	NA	NA	0.000	NA	NA	NA	0.000	0.000	0.000	NA	0.000	NA	0.000	NA	NA	NA	NA
rs28915406	G	0.000	NA	0.000	0.000	NA	NA	NA	0.020	NA	NA	NA	0.006	0.000	0.000	NA	0.000	NA	0.000	NA	NA	NA	NA
rs560808777	A	0.000	NA	0.000	0.000	NA	NA	NA	0.000	NA	NA	NA	0.018	0.000	0.000	NA	0.000	NA	0.000	NA	NA	NA	NA
rs553790377	A	0.005	NA	0.000	0.000	NA	NA	NA	0.000	NA	NA	NA	0.000	0.000	0.000	NA	0.000	NA	0.000	NA	NA	NA	NA
rs576854854	T	0.000	NA	0.000	0.000	NA	NA	NA	0.000	NA	NA	NA	0.000	0.000	0.006	NA	0.000	NA	0.000	NA	NA	NA	NA
rs558986305	A	0.000	NA	0.000	0.000	NA	NA	NA	0.000	NA	NA	NA	0.000	0.000	0.000	NA	0.000	NA	0.000	NA	NA	NA	NA
rs185398568	A	0.000	NA	0.000	0.000	NA	NA	NA	0.000	NA	NA	NA	0.000	0.000	0.000	NA	0.000	NA	0.000	NA	NA	NA	NA
rs555055894	A	0.000	NA	0.000	0.000	NA	NA	NA	0.000	NA	NA	NA	0.006	0.000	0.000	NA	0.000	NA	0.000	NA	NA	NA	NA
rs181812598	T	0.000	NA	0.000	0.000	NA	NA	NA	0.000	NA	NA	NA	0.000	0.000	0.000	NA	0.000	NA	0.000	NA	NA	NA	NA
rs534560674	G	0.000	NA	0.000	0.000	NA	NA	NA	0.000	NA	NA	NA	0.000	0.000	0.000	NA	0.000	NA	0.000	NA	NA	NA	NA

SNP	Allele	ITU	Jacarus	JPT	KHV	Lamas	Lambayeque	Lima	LWK	Matses	Moche	Moquegua	MSL	MXL	PEL	Pelotas	PJL	Puno	PUR	Qeros	Quechuas	Salvador	Shimaa
rs2238541	T	0.000	NA	0.010	0.020	NA	NA	NA	0.000	NA	NA	NA	0.000	0.031	0.035	NA	0.000	NA	0.024	NA	NA	NA	NA
rs148868153	T	0.000	NA	0.000	0.000	NA	NA	NA	0.000	NA	NA	NA	0.000	0.000	0.000	NA	0.000	NA	0.000	NA	NA	NA	NA
rs10422922	G	0.029	NA	0.072	0.030	NA	NA	NA	0.157	NA	NA	NA	0.229	0.078	0.024	NA	0.016	NA	0.091	NA	NA	NA	NA
rs529166895	A	0.005	NA	0.000	0.000	NA	NA	NA	0.000	NA	NA	NA	0.000	0.000	0.000	NA	0.005	NA	0.000	NA	NA	NA	NA
rs570767821	A	0.000	NA	0.000	0.010	NA	NA	NA	0.000	NA	NA	NA	0.000	0.000	0.000	NA	0.000	NA	0.000	NA	NA	NA	NA
rs572997523	A	0.000	NA	0.000	0.000	NA	NA	NA	0.000	NA	NA	NA	0.000	0.000	0.000	NA	0.005	NA	0.000	NA	NA	NA	NA
rs527567567	T	0.000	NA	0.000	0.000	NA	NA	NA	0.000	NA	NA	NA	0.000	0.000	0.000	NA	0.000	NA	0.010	NA	NA	NA	NA
rs187875345	A	0.000	NA	0.000	0.000	NA	NA	NA	0.000	NA	NA	NA	0.000	0.000	0.000	NA	0.000	NA	0.000	NA	NA	NA	NA
rs10412522	A	0.029	0.059	0.072	0.030	0.000	0.000	0.018	0.121	0.000	0.017	0.017	0.153	0.078	0.024	0.086	0.016	0.000	0.087	0.000	0.000	0.089	0.000
rs199856669	G	0.000	NA	0.087	0.086	NA	NA	NA	0.000	NA	NA	NA	0.000	0.055	0.024	NA	0.000	NA	0.000	NA	NA	NA	NA
rs113431687	A	NA	NA	NA	NA	NA	NA	NA	NA	NA	NA	NA	NA	NA	NA	NA	NA	NA	NA	NA	NA	NA	NA
rs575490602	T	0.000	NA	0.000	0.000	NA	NA	NA	0.000	NA	NA	NA	0.000	0.000	0.000	NA	0.000	NA	0.000	NA	NA	NA	NA
rs142683396	A	0.000	NA	0.000	0.000	NA	NA	NA	0.000	NA	NA	NA	0.000	0.000	0.000	NA	0.000	NA	0.000	NA	NA	NA	NA
rs543921498	A	0.000	NA	0.000	0.000	NA	NA	NA	0.000	NA	NA	NA	0.000	0.000	0.000	NA	0.000	NA	0.000	NA	NA	NA	NA
rs199564958	A	0.000	NA	0.000	0.000	NA	NA	NA	0.000	NA	NA	NA	0.000	0.000	0.000	NA	0.000	NA	0.000	NA	NA	NA	NA
rs528839538	G	0.000	NA	0.000	0.000	NA	NA	NA	0.000	NA	NA	NA	0.000	0.000	0.000	NA	0.000	NA	0.000	NA	NA	NA	NA
rs541648314	G	0.000	NA	0.000	0.000	NA	NA	NA	0.000	NA	NA	NA	0.000	0.000	0.000	NA	0.000	NA	0.005	NA	NA	NA	NA
rs182434553	G	0.000	NA	0.000	0.000	NA	NA	NA	0.005	NA	NA	NA	0.000	0.000	0.000	NA	0.000	NA	0.000	NA	NA	NA	NA
rs577301914	A	0.000	NA	0.000	0.000	NA	NA	NA	0.000	NA	NA	NA	0.000	0.000	0.000	NA	0.000	NA	0.000	NA	NA	NA	NA
rs111517369	T	0.000	NA	0.000	0.000	NA	NA	NA	0.020	NA	NA	NA	0.000	0.000	0.000	NA	0.000	NA	0.000	NA	NA	NA	NA
rs193000162	A	0.000	NA	0.000	0.000	NA	NA	NA	0.000	NA	NA	NA	0.000	0.008	0.000	NA	0.000	NA	0.000	NA	NA	NA	NA
rs185020271	G	0.000	NA	0.010	0.000	NA	NA	NA	0.000	NA	NA	NA	0.000	0.000	0.000	NA	0.000	NA	0.000	NA	NA	NA	NA
rs560734336	T	0.000	NA	0.000	0.000	NA	NA	NA	0.000	NA	NA	NA	0.000	0.000	0.000	NA	0.000	NA	0.000	NA	NA	NA	NA
rs527804308	T	0.000	NA	0.000	0.000	NA	NA	NA	0.000	NA	NA	NA	0.000	0.000	0.000	NA	0.000	NA	0.000	NA	NA	NA	NA
rs374000063	A	0.000	NA	0.000	0.000	NA	NA	NA	0.010	NA	NA	NA	0.047	0.008	0.006	NA	0.000	NA	0.000	NA	NA	NA	NA
rs574157191	A	0.000	NA	0.000	0.000	NA	NA	NA	0.000	NA	NA	NA	0.000	0.000	0.000	NA	0.000	NA	0.000	NA	NA	NA	NA
rs144058382	C	0.000	NA	0.019	0.000	NA	NA	NA	0.000	NA	NA	NA	0.000	0.000	0.000	NA	0.000	NA	0.000	NA	NA	NA	NA
rs10422066	A	0.539	NA	0.563	0.450	NA	NA	NA	0.167	NA	NA	NA	0.029	0.508	0.359	NA	0.495	NA	0.635	NA	NA	NA	NA
rs61729580	A	0.005	NA	0.000	0.000	NA	NA	NA	0.000	NA	NA	NA	0.000	0.008	0.006	NA	0.000	NA	0.005	NA	NA	NA	NA
rs565778867	A	0.010	NA	0.000	0.000	NA	NA	NA	0.000	NA	NA	NA	0.000	0.000	0.000	NA	0.000	NA	0.000	NA	NA	NA	NA
rs1803535	A	0.005	0.029	0.000	0.000	0.000	0.000	0.000	0.000	0.136	0.000	0.000	0.000	0.023	0.000	0.017	0.005	0.000	0.014	0.000	0.000	0.006	0.000
rs539526884	A	0.000	NA	0.005	0.000	NA	NA	NA	0.000	NA	NA	NA	0.000	0.000	0.000	NA	0.000	NA	0.000	NA	NA	NA	NA
rs536177717	A	0.000	NA	0.000	0.000	NA	NA	NA	0.000	NA	NA	NA	0.000	0.000	0.000	NA	0.005	NA	0.000	NA	NA	NA	NA
rs530649173	C	0.000	NA	0.000	0.000	NA	NA	NA	0.000	NA	NA	NA	0.000	0.000	0.000	NA	0.000	NA	0.000	NA	NA	NA	NA
rs181996046	T	0.000	NA	0.000	0.000	NA	NA	NA	0.000	NA	NA	NA	0.000	0.000	0.000	NA	0.000	NA	0.000	NA	NA	NA	NA
rs546352239	T	0.000	NA	0.000	0.000	NA	NA	NA	0.005	NA	NA	NA	0.000	0.008	0.000	NA	0.000	NA	0.000	NA	NA	NA	NA
rs531528464	A	0.000	NA	0.000	0.000	NA	NA	NA	0.000	NA	NA	NA	0.000	0.000	0.000	NA	0.000	NA	0.000	NA	NA	NA	NA
rs537327096	T	0.000	NA	0.000	0.000	NA	NA	NA	0.000	NA	NA	NA	0.000	0.000	0.000	NA	0.000	NA	0.000	NA	NA	NA	NA
rs28921978	T	0.074	NA	0.005	0.015	NA	NA	NA	0.005	NA	NA	NA	0.000	0.133	0.329	NA	0.125	NA	0.101	NA	NA	NA	NA
rs541295754	T	0.000	NA	0.000	0.000	NA	NA	NA	0.000	NA	NA	NA	0.000	0.000	0.000	NA	0.000	NA	0.000	NA	NA	NA	NA
rs201497117	A	0.000	NA	0.000	0.000	NA	NA	NA	0.000	NA	NA	NA	0.000	0.000	0.000	NA	0.000	NA	0.000	NA	NA	NA	NA
rs564731463	A	0.000	NA	0.000	0.000	NA	NA	NA	0.000	NA	NA	NA	0.000	0.000	0.000	NA	0.000	NA	0.000	NA	NA	NA	NA
rs542324120	T	0.000	NA	0.000	0.000	NA	NA	NA	0.000	NA	NA	NA	0.000	0.000	0.000	NA	0.000	NA	0.000	NA	NA	NA	NA

SNP	Allele	ITU	Jacarus	JPT	KHV	Lamas	Lambayeque	Lima	LWK	Matses	Moche	Moquegua	MSL	MXL	PEL	Pelotas	PJL	Puno	PUR	Qeros	Quechuas	Salvador	Shimaa
rs112540819	A	0.000	NA	0.000	0.000	NA	NA	NA	0.005	NA	NA	NA	0.000	0.000	0.000	NA	0.000	NA	0.000	NA	NA	NA	NA
rs145586063	T	0.000	NA	0.000	0.000	NA	NA	NA	0.005	NA	NA	NA	0.000	0.000	0.000	NA	0.000	NA	0.000	NA	NA	NA	NA
rs556111109	T	0.000	NA	0.005	0.000	NA	NA	NA	0.000	NA	NA	NA	0.000	0.000	0.000	NA	0.000	NA	0.000	NA	NA	NA	NA
rs544240790	A	0.000	NA	0.000	0.000	NA	NA	NA	0.000	NA	NA	NA	0.000	0.000	0.000	NA	0.000	NA	0.000	NA	NA	NA	NA
rs527846123	A	0.000	NA	0.000	0.000	NA	NA	NA	0.000	NA	NA	NA	0.000	0.000	0.000	NA	0.000	NA	0.000	NA	NA	NA	NA
rs200823690	AC	0.000	NA	0.087	0.086	NA	NA	NA	0.000	NA	NA	NA	0.000	0.055	0.024	NA	0.000	NA	0.000	NA	NA	NA	NA
rs547374902	A	0.000	NA	0.000	0.010	NA	NA	NA	0.000	NA	NA	NA	0.000	0.000	0.000	NA	0.000	NA	0.000	NA	NA	NA	NA
rs534410335	T	0.000	NA	0.000	0.005	NA	NA	NA	0.000	NA	NA	NA	0.000	0.000	0.000	NA	0.000	NA	0.000	NA	NA	NA	NA
rs11551900	G	0.000	NA	0.000	0.000	NA	NA	NA	0.005	NA	NA	NA	0.018	0.000	0.000	NA	0.000	NA	0.000	NA	NA	NA	NA
rs188657384	T	0.000	NA	0.000	0.000	NA	NA	NA	0.015	NA	NA	NA	0.000	0.000	0.000	NA	0.000	NA	0.000	NA	NA	NA	NA
rs7260603	A	0.103	NA	0.077	0.045	NA	NA	NA	0.162	NA	NA	NA	0.229	0.211	0.353	NA	0.141	NA	0.192	NA	NA	NA	NA
rs537898048	A	0.000	NA	0.000	0.000	NA	NA	NA	0.000	NA	NA	NA	0.000	0.000	0.000	NA	0.000	NA	0.000	NA	NA	NA	NA
rs575502711	A	0.025	NA	0.000	0.000	NA	NA	NA	0.000	NA	NA	NA	0.000	0.000	0.000	NA	0.010	NA	0.000	NA	NA	NA	NA
rs532039913	A	0.000	NA	0.000	0.000	NA	NA	NA	0.000	NA	NA	NA	0.000	0.000	0.000	NA	0.000	NA	0.000	NA	NA	NA	NA
rs558360796	C	0.000	NA	0.000	0.000	NA	NA	NA	0.000	NA	NA	NA	0.000	0.000	0.000	NA	0.000	NA	0.000	NA	NA	NA	NA
rs547687363	G	0.000	NA	0.000	0.000	NA	NA	NA	0.000	NA	NA	NA	0.006	0.000	0.000	NA	0.000	NA	0.000	NA	NA	NA	NA
rs144193084	T	0.000	NA	0.005	0.000	NA	NA	NA	0.000	NA	NA	NA	0.000	0.000	0.000	NA	0.000	NA	0.000	NA	NA	NA	NA
rs28992473	G	0.000	NA	0.000	0.000	NA	NA	NA	0.056	NA	NA	NA	0.047	0.000	0.000	0.000	0.000	NA	0.000	NA	NA	0.022	NA
rs564064006	G	0.000	NA	0.000	0.000	NA	NA	NA	0.000	NA	NA	NA	0.006	0.000	0.000	NA	0.000	NA	0.000	NA	NA	NA	NA
rs73918141	T	0.000	NA	0.000	0.000	NA	NA	NA	0.015	NA	NA	NA	0.000	0.000	0.000	NA	0.000	NA	0.000	NA	NA	NA	NA
rs537163143	A	0.000	NA	0.000	0.000	NA	NA	NA	0.010	NA	NA	NA	0.000	0.000	0.000	NA	0.000	NA	0.000	NA	NA	NA	NA
rs559463709	A	0.000	NA	0.000	0.000	NA	NA	NA	0.000	NA	NA	NA	0.000	0.000	0.000	NA	0.000	NA	0.000	NA	NA	NA	NA
rs28921986	T	0.000	NA	0.005	0.000	NA	NA	NA	0.025	NA	NA	NA	0.012	0.000	0.000	NA	0.000	NA	0.000	NA	NA	NA	NA
rs7252138	T	0.157	0.206	0.284	0.172	0.071	0.208	0.232	0.025	0.318	0.100	0.310	0.000	0.281	0.235	0.374	0.156	0.300	0.414	0.042	0.292	0.194	0.239
rs537290646	G	0.000	NA	0.000	0.000	NA	NA	NA	0.000	NA	NA	NA	0.000	0.000	0.000	NA	0.000	NA	0.000	NA	NA	NA	NA
rs12609013	T	0.284	NA	0.125	0.293	NA	NA	NA	0.172	NA	NA	NA	0.247	0.094	0.041	NA	0.203	NA	0.115	NA	NA	NA	NA
rs144824657	T	0.000	NA	0.000	0.000	NA	NA	NA	0.000	NA	NA	NA	0.000	0.000	0.000	NA	0.000	NA	0.005	NA	NA	NA	NA
rs2283569	C	0.324	NA	0.125	0.298	NA	NA	NA	0.172	NA	NA	NA	0.247	0.094	0.047	NA	0.208	NA	0.115	NA	NA	NA	NA
rs532689650	A	0.000	NA	0.000	0.000	NA	NA	NA	0.010	NA	NA	NA	0.047	0.008	0.000	NA	0.000	NA	0.000	NA	NA	NA	NA
rs549863661	T	0.000	NA	0.005	0.000	NA	NA	NA	0.000	NA	NA	NA	0.000	0.000	0.000	NA	0.005	NA	0.000	NA	NA	NA	NA
rs199862501	T	0.000	NA	0.000	0.000	NA	NA	NA	0.000	NA	NA	NA	0.000	0.000	0.000	NA	0.000	NA	0.000	NA	NA	NA	NA
rs575727796	G	0.000	NA	0.000	0.000	NA	NA	NA	0.005	NA	NA	NA	0.000	0.000	0.000	NA	0.000	NA	0.000	NA	NA	NA	NA
rs539913003	A	0.000	NA	0.005	0.000	NA	NA	NA	0.000	NA	NA	NA	0.000	0.000	0.000	NA	0.000	NA	0.000	NA	NA	NA	NA
rs532703227	A	0.010	NA	0.000	0.005	NA	NA	NA	0.000	NA	NA	NA	0.000	0.000	0.000	NA	0.010	NA	0.010	NA	NA	NA	NA
rs1138151	A	0.196	NA	0.293	0.177	NA	NA	NA	0.025	NA	NA	NA	0.000	0.281	0.235	NA	0.162	NA	0.414	NA	NA	NA	NA
rs537512067	T	0.000	NA	0.005	0.000	NA	NA	NA	0.000	NA	NA	NA	0.000	0.000	0.000	NA	0.000	NA	0.000	NA	NA	NA	NA
rs569562559	A	0.000	NA	0.000	0.000	NA	NA	NA	0.000	NA	NA	NA	0.000	0.000	0.000	NA	0.000	NA	0.000	NA	NA	NA	NA
rs28541371	T	0.000	NA	0.000	0.000	NA	NA	NA	0.020	NA	NA	NA	0.000	0.000	0.000	NA	0.000	NA	0.000	NA	NA	NA	NA
rs547640504	T	0.000	NA	0.000	0.000	NA	NA	NA	0.000	NA	NA	NA	0.000	0.000	0.000	NA	0.000	NA	0.000	NA	NA	NA	NA
rs368481272	T	0.000	NA	0.000	0.000	NA	NA	NA	0.000	NA	NA	NA	0.000	0.000	0.000	NA	0.000	NA	0.000	NA	NA	NA	NA
rs138672257	A	0.000	NA	0.000	0.000	NA	NA	NA	0.000	NA	NA	NA	0.000	0.000	0.000	NA	0.000	NA	0.000	NA	NA	NA	NA
rs150718850	A	0.010	NA	0.000	0.000	NA	NA	NA	0.000	NA	NA	NA	0.000	0.008	0.012	0.000	0.000	NA	0.005	NA	NA	0.006	NA
rs28921976	C	0.000	NA	0.000	0.000	NA	NA	NA	0.000	NA	NA	NA	0.000	0.000	0.000	NA	0.000	NA	0.005	NA	NA	NA	NA

SNP	Allele	ITU	Jacarus	JPT	KHV	Lamas	Lambayeque	Lima	LWK	Matses	Moche	Moquegua	MSL	MXL	PEL	Pelotas	PJL	Puno	PUR	Qeros	Quechuas	Salvador	Shimaa
rs28921989	T	0.000	NA	0.000	0.000	NA	NA	NA	0.000	NA	NA	NA	0.000	0.008	0.000	NA	0.026	NA	0.000	NA	NA	NA	NA
rs541886354	C	0.000	NA	0.000	0.005	NA	NA	NA	0.000	NA	NA	NA	0.000	0.000	0.000	NA	0.000	NA	0.000	NA	NA	NA	NA
rs373097790	C	0.000	NA	0.005	0.000	NA	NA	NA	0.000	NA	NA	NA	0.000	0.000	0.000	NA	0.000	NA	0.000	NA	NA	NA	NA
rs546562394	A	0.000	NA	0.000	0.000	NA	NA	NA	0.000	NA	NA	NA	0.000	0.000	0.000	NA	0.005	NA	0.000	NA	NA	NA	NA
rs555516404	A	0.000	NA	0.000	0.005	NA	NA	NA	0.000	NA	NA	NA	0.000	0.000	0.000	NA	0.000	NA	0.000	NA	NA	NA	NA
rs572599699	T	0.000	NA	0.000	0.000	NA	NA	NA	0.000	NA	NA	NA	0.000	0.000	0.000	NA	0.000	NA	0.000	NA	NA	NA	NA
rs560691399	T	0.000	NA	0.000	0.000	NA	NA	NA	0.005	NA	NA	NA	0.000	0.000	0.000	NA	0.000	NA	0.000	NA	NA	NA	NA
rs192403165	T	0.000	NA	0.000	0.000	NA	NA	NA	0.000	NA	NA	NA	0.000	0.000	0.000	NA	0.000	NA	0.000	NA	NA	NA	NA
rs529959881	C	0.000	NA	0.000	0.000	NA	NA	NA	0.000	NA	NA	NA	0.000	0.000	0.000	NA	0.000	NA	0.000	NA	NA	NA	NA
rs563525909	T	0.000	NA	0.000	0.000	NA	NA	NA	0.010	NA	NA	NA	0.006	0.000	0.000	NA	0.000	NA	0.000	NA	NA	NA	NA
rs552948152	A	0.000	NA	0.000	0.000	NA	NA	NA	0.000	NA	NA	NA	0.000	0.000	0.000	NA	0.000	NA	0.000	NA	NA	NA	NA
rs5826703	GA	0.206	NA	0.293	0.182	NA	NA	NA	0.040	NA	NA	NA	0.006	0.281	0.241	NA	0.172	NA	0.428	NA	NA	NA	NA
rs571034655	T	0.000	NA	0.000	0.000	NA	NA	NA	0.000	NA	NA	NA	0.000	0.000	0.000	NA	0.000	NA	0.000	NA	NA	NA	NA
rs570927174	G	0.000	NA	0.000	0.000	NA	NA	NA	0.000	NA	NA	NA	0.000	0.000	0.000	NA	0.000	NA	0.000	NA	NA	NA	NA
rs552366872	T	0.000	NA	0.000	0.000	NA	NA	NA	0.000	NA	NA	NA	0.000	0.000	0.000	NA	0.000	NA	0.000	NA	NA	NA	NA
rs559051628	A	0.000	NA	0.000	0.000	NA	NA	NA	0.000	NA	NA	NA	0.000	0.000	0.000	NA	0.000	NA	0.000	NA	NA	NA	NA
rs556312871	A	0.000	NA	0.005	0.000	NA	NA	NA	0.000	NA	NA	NA	0.000	0.000	0.000	NA	0.000	NA	0.000	NA	NA	NA	NA
rs567001549	G	0.000	NA	0.000	0.005	NA	NA	NA	0.000	NA	NA	NA	0.000	0.000	0.000	NA	0.000	NA	0.000	NA	NA	NA	NA
rs533301686	A	0.000	NA	0.000	0.000	NA	NA	NA	0.000	NA	NA	NA	0.000	0.000	0.018	NA	0.000	NA	0.000	NA	NA	NA	NA
rs13676	A	0.074	0.353	0.005	0.015	0.476	0.292	0.286	0.005	0.364	0.300	0.259	0.000	0.133	0.324	0.071	0.125	0.288	0.101	0.417	0.292	0.089	0.391
rs563356581	A	0.005	NA	0.000	0.000	NA	NA	NA	0.000	NA	NA	NA	0.000	0.000	0.000	NA	0.000	NA	0.000	NA	NA	NA	NA
rs111929936	A	0.000	0.000	0.000	0.000	0.000	0.000	0.000	0.030	0.000	0.000	0.000	0.047	0.000	0.000	0.000	0.000	0.000	0.000	0.000	0.000	0.017	0.000
rs151017812	T	0.000	NA	0.000	0.000	NA	NA	NA	0.005	NA	NA	NA	0.000	0.000	0.000	NA	0.000	NA	0.000	NA	NA	NA	NA
rs28992485	T	0.279	NA	0.130	0.293	NA	NA	NA	0.172	NA	NA	NA	0.247	0.094	0.041	NA	0.208	NA	0.115	NA	NA	NA	NA
rs578018107	A	0.000	NA	0.000	0.000	NA	NA	NA	0.000	NA	NA	NA	0.006	0.000	0.000	NA	0.000	NA	0.000	NA	NA	NA	NA
rs543483161	T	0.000	NA	0.000	0.005	NA	NA	NA	0.000	NA	NA	NA	0.000	0.000	0.000	NA	0.000	NA	0.000	NA	NA	NA	NA
rs368686315	C	0.000	NA	0.000	0.000	NA	NA	NA	0.000	NA	NA	NA	0.000	0.000	0.000	NA	0.000	NA	0.000	NA	NA	NA	NA
rs535212456	T	0.000	NA	0.000	0.005	NA	NA	NA	0.000	NA	NA	NA	0.006	0.000	0.000	NA	0.000	NA	0.000	NA	NA	NA	NA
rs202212562	T	0.000	NA	0.000	0.000	NA	NA	NA	0.000	NA	NA	NA	0.000	0.000	0.000	NA	0.000	NA	0.000	NA	NA	NA	NA
rs2229662	T	NA	NA	NA	NA	NA	NA	NA	NA	NA	NA	NA	NA	NA	NA	0.000	NA	NA	NA	NA	NA	0.000	NA
rs150860707	T	0.000	NA	0.000	0.000	NA	NA	NA	0.000	NA	NA	NA	0.000	0.000	0.000	NA	0.000	NA	0.000	NA	NA	NA	NA
rs543519383	A	0.000	NA	0.000	0.000	NA	NA	NA	0.000	NA	NA	NA	0.000	0.000	0.000	NA	0.000	NA	0.000	NA	NA	NA	NA
rs199725376	T	0.010	NA	0.005	0.015	NA	NA	NA	0.015	NA	NA	NA	0.000	0.000	0.000	NA	0.010	NA	0.000	NA	NA	NA	NA
rs10402344	T	0.755	NA	0.889	0.864	NA	NA	NA	0.268	NA	NA	NA	0.076	0.727	0.553	NA	0.708	NA	0.702	NA	NA	NA	NA
rs200189683	A	0.000	NA	0.000	0.000	NA	NA	NA	0.010	NA	NA	NA	0.000	0.000	0.000	NA	0.000	NA	0.000	NA	NA	NA	NA
rs113445094	T	NA	NA	NA	NA	NA	NA	NA	NA	NA	NA	NA	NA	NA	NA	NA	NA	NA	NA	NA	NA	NA	NA
rs527594490	G	0.000	NA	0.000	0.005	NA	NA	NA	0.000	NA	NA	NA	0.000	0.000	0.000	NA	0.000	NA	0.000	NA	NA	NA	NA
rs545107557	T	0.005	NA	0.000	0.000	NA	NA	NA	0.000	NA	NA	NA	0.000	0.000	0.000	NA	0.000	NA	0.000	NA	NA	NA	NA
rs7259828	T	0.103	NA	0.077	0.045	NA	NA	NA	0.157	NA	NA	NA	0.229	0.211	0.353	NA	0.141	NA	0.192	NA	NA	NA	NA
rs112251245	T	0.000	NA	0.000	0.000	NA	NA	NA	0.061	NA	NA	NA	0.076	0.000	0.000	NA	0.000	NA	0.005	NA	NA	NA	NA
rs557528051	G	0.000	NA	0.000	0.000	NA	NA	NA	0.000	NA	NA	NA	0.000	0.000	0.000	NA	0.000	NA	0.000	NA	NA	NA	NA
rs540128503	T	0.000	NA	0.000	0.000	NA	NA	NA	0.000	NA	NA	NA	0.000	0.000	0.000	NA	0.005	NA	0.000	NA	NA	NA	NA
rs529018328	T	0.000	NA	0.000	0.005	NA	NA	NA	0.000	NA	NA	NA	0.000	0.000	0.000	NA	0.000	NA	0.000	NA	NA	NA	NA

SNP	Allele	ITU	Jacarus	JPT	KHV	Lamas	Lambayeque	Lima	LWK	Matses	Moche	Moquegua	MSL	MXL	PEL	Pelotas	PJL	Puno	PUR	Qeros	Quechuas	Salvador	Shimaa
rs183742660	A	0.000	NA	0.000	0.000	NA	NA	NA	0.000	NA	NA	NA	0.000	0.000	0.000	NA	0.000	NA	0.000	NA	NA	NA	NA
rs572465381	GGGC	0.000	NA	0.000	0.000	NA	NA	NA	0.020	NA	NA	NA	0.047	0.000	0.000	NA	0.000	NA	0.000	NA	NA	NA	NA
rs549282195	GAGAT	0.000	NA	0.000	0.000	NA	NA	NA	0.005	NA	NA	NA	0.012	0.000	0.000	NA	0.000	NA	0.000	NA	NA	NA	NA
rs545279552	C	0.000	NA	0.000	0.005	NA	NA	NA	0.000	NA	NA	NA	0.000	0.000	0.000	NA	0.000	NA	0.000	NA	NA	NA	NA
rs201531347	A	0.000	NA	0.000	0.000	NA	NA	NA	0.000	NA	NA	NA	0.000	0.000	0.000	NA	0.000	NA	0.005	NA	NA	NA	NA
rs566474268	A	0.000	NA	0.000	0.000	NA	NA	NA	0.005	NA	NA	NA	0.000	0.000	0.000	NA	0.000	NA	0.000	NA	NA	NA	NA
rs560075963	C	0.000	NA	0.000	0.000	NA	NA	NA	0.000	NA	NA	NA	0.000	0.000	0.000	NA	0.000	NA	0.000	NA	NA	NA	NA
rs555375779	T	0.010	NA	0.000	0.000	NA	NA	NA	0.000	NA	NA	NA	0.000	0.000	0.000	NA	0.000	NA	0.000	NA	NA	NA	NA
rs551356579	T	0.000	NA	0.000	0.000	NA	NA	NA	0.000	NA	NA	NA	0.000	0.000	0.000	NA	0.000	NA	0.005	NA	NA	NA	NA
rs541386550	T	0.000	NA	0.000	0.000	NA	NA	NA	0.000	NA	NA	NA	0.000	0.000	0.000	NA	0.000	NA	0.019	NA	NA	NA	NA
rs184596705	A	0.000	NA	0.000	0.000	NA	NA	NA	0.000	NA	NA	NA	0.000	0.000	0.000	NA	0.000	NA	0.000	NA	NA	NA	NA
rs139397975	A	0.000	NA	0.000	0.000	NA	NA	NA	0.025	NA	NA	NA	0.000	0.000	0.000	NA	0.000	NA	0.005	NA	NA	NA	NA
rs567401469	T	0.000	NA	0.005	0.000	NA	NA	NA	0.000	NA	NA	NA	0.000	0.000	0.000	NA	0.000	NA	0.000	NA	NA	NA	NA
rs11473	T	0.000	NA	0.000	0.000	NA	NA	NA	0.081	NA	NA	NA	0.206	0.000	0.006	0.011	0.000	NA	0.010	NA	NA	0.078	NA
rs192885278	A	0.000	NA	0.000	0.000	NA	NA	NA	0.000	NA	NA	NA	0.000	0.000	0.000	NA	0.000	NA	0.000	NA	NA	NA	NA
rs572733540	G	0.363	NA	0.034	0.040	NA	NA	NA	0.131	NA	NA	NA	0.429	0.094	0.082	NA	0.406	NA	0.082	NA	NA	NA	NA
rs145675941	A	0.000	NA	0.000	0.000	NA	NA	NA	0.000	NA	NA	NA	0.000	0.000	0.000	NA	0.000	NA	0.000	NA	NA	NA	NA
rs547384835	T	0.000	NA	0.000	0.000	NA	NA	NA	0.000	NA	NA	NA	0.000	0.000	0.000	NA	0.000	NA	0.000	NA	NA	NA	NA
rs140385832	C	0.000	NA	0.005	0.000	NA	NA	NA	0.000	NA	NA	NA	0.000	0.000	0.000	NA	0.000	NA	0.000	NA	NA	NA	NA
rs13235	T	0.000	NA	0.000	0.000	NA	NA	NA	0.010	NA	NA	NA	0.006	0.008	0.000	0.000	0.000	NA	0.005	NA	NA	0.000	NA
rs573279565	A	0.000	NA	0.000	0.000	NA	NA	NA	0.000	NA	NA	NA	0.000	0.000	0.000	NA	0.000	NA	0.000	NA	NA	NA	NA
rs117933091	A	0.010	NA	0.014	0.020	NA	NA	NA	0.015	NA	NA	NA	0.006	0.000	0.000	NA	0.010	NA	0.000	NA	NA	NA	NA
rs2238544	A	0.245	NA	0.284	0.177	NA	NA	NA	0.091	NA	NA	NA	0.006	0.305	0.259	NA	0.250	NA	0.452	NA	NA	NA	NA
rs372040583	A	0.000	NA	0.000	0.000	NA	NA	NA	0.000	NA	NA	NA	0.000	0.000	0.000	NA	0.000	NA	0.000	NA	NA	NA	NA
rs530681399	T	0.000	NA	0.000	0.000	NA	NA	NA	0.000	NA	NA	NA	0.000	0.000	0.000	NA	0.000	NA	0.010	NA	NA	NA	NA
rs191110653	A	0.010	NA	0.000	0.005	NA	NA	NA	0.005	NA	NA	NA	0.000	0.000	0.000	NA	0.010	NA	0.010	NA	NA	NA	NA
rs3764938	T	0.446	NA	0.207	0.359	NA	NA	NA	0.409	NA	NA	NA	0.594	0.313	0.406	NA	0.370	NA	0.332	NA	NA	NA	NA
rs139776734	A	0.000	NA	0.000	0.000	NA	NA	NA	0.000	NA	NA	NA	0.000	0.000	0.000	NA	0.005	NA	0.000	NA	NA	NA	NA
rs575050179	A	0.000	NA	0.000	0.005	NA	NA	NA	0.000	NA	NA	NA	0.000	0.000	0.000	NA	0.000	NA	0.000	NA	NA	NA	NA
rs572043068	A	0.000	NA	0.000	0.000	NA	NA	NA	0.000	NA	NA	NA	0.000	0.000	0.000	NA	0.000	NA	0.000	NA	NA	NA	NA
rs2238545	C	0.284	NA	0.125	0.298	NA	NA	NA	0.177	NA	NA	NA	0.247	0.094	0.041	NA	0.203	NA	0.115	NA	NA	NA	NA
rs533373164	T	0.000	NA	0.000	0.000	NA	NA	NA	0.000	NA	NA	NA	0.000	0.000	0.006	NA	0.000	NA	0.010	NA	NA	NA	NA
rs201635915	A	0.005	NA	0.000	0.000	NA	NA	NA	0.000	NA	NA	NA	0.000	0.000	0.000	NA	0.000	NA	0.000	NA	NA	NA	NA
rs541344713	G	0.000	NA	0.000	0.000	NA	NA	NA	0.000	NA	NA	NA	0.000	0.000	0.000	NA	0.000	NA	0.000	NA	NA	NA	NA
rs567649209	T	0.000	NA	0.000	0.000	NA	NA	NA	0.000	NA	NA	NA	0.000	0.000	0.000	NA	0.000	NA	0.000	NA	NA	NA	NA
rs529458335	T	0.000	NA	0.000	0.000	NA	NA	NA	0.000	NA	NA	NA	0.000	0.000	0.000	NA	0.000	NA	0.000	NA	NA	NA	NA
rs186158048	T	0.000	NA	0.000	0.000	NA	NA	NA	0.010	NA	NA	NA	0.000	0.008	0.000	NA	0.000	NA	0.000	NA	NA	NA	NA
rs560636050	A	0.000	NA	0.000	0.005	NA	NA	NA	0.000	NA	NA	NA	0.000	0.000	0.000	NA	0.000	NA	0.000	NA	NA	NA	NA
rs111841168	C	NA	NA	NA	NA	NA	NA	NA	NA	NA	NA	NA	NA	NA	NA	NA	NA	NA	NA	NA	NA	NA	NA
rs137922704	T	0.000	NA	0.000	0.000	NA	NA	NA	0.010	NA	NA	NA	0.006	0.000	0.000	NA	0.000	NA	0.000	NA	NA	NA	NA
rs28992470	A	0.000	NA	0.000	0.005	NA	NA	NA	0.000	NA	NA	NA	0.000	0.016	0.012	0.006	0.016	NA	0.010	NA	NA	0.000	NA
rs190631157	T	0.000	NA	0.000	0.000	NA	NA	NA	0.015	NA	NA	NA	0.000	0.008	0.000	NA	0.000	NA	0.000	NA	NA	NA	NA
rs28921991	A	0.000	NA	0.000	0.000	NA	NA	NA	0.040	NA	NA	NA	0.024	0.000	0.000	NA	0.000	NA	0.000	NA	NA	NA	NA

SNP	Allele	ITU	Jacarus	JPT	KHV	Lamas	Lambayeque	Lima	LWK	Matses	Moche	Moquegua	MSL	MXL	PEL	Pelotas	PJL	Puno	PUR	Qeros	Quechuas	Salvador	Shimaa
rs577780415	G	0.000	NA	0.000	0.000	NA	NA	NA	0.000	NA	NA	NA	0.000	0.000	0.000	NA	0.000	NA	0.000	NA	NA	NA	NA
rs1063378	C	NA	NA	NA	NA	NA	NA	NA	NA	NA	NA	NA	NA	NA	NA	NA	NA	NA	NA	NA	NA	NA	NA
rs140499328	T	0.284	NA	0.125	0.298	NA	NA	NA	0.172	NA	NA	NA	0.247	0.094	0.041	NA	0.203	NA	0.115	NA	NA	NA	NA
rs28921972	A	0.000	NA	0.000	0.000	NA	NA	NA	0.000	NA	NA	NA	0.000	0.000	0.000	0.000	0.000	NA	0.000	NA	NA	0.000	NA
rs191842753	T	0.000	NA	0.000	0.000	NA	NA	NA	0.000	NA	NA	NA	0.000	0.008	0.006	NA	0.000	NA	0.010	NA	NA	NA	NA
rs150369286	T	0.000	NA	0.000	0.000	NA	NA	NA	0.010	NA	NA	NA	0.012	0.000	0.000	NA	0.000	NA	0.005	NA	NA	NA	NA
rs28992484	T	0.324	NA	0.130	0.293	NA	NA	NA	0.172	NA	NA	NA	0.247	0.094	0.041	NA	0.214	NA	0.115	NA	NA	NA	NA
rs140722203	A	0.000	NA	0.000	0.000	NA	NA	NA	0.000	NA	NA	NA	0.000	0.000	0.000	NA	0.000	NA	0.000	NA	NA	NA	NA
rs546300301	G	0.000	NA	0.000	0.000	NA	NA	NA	0.000	NA	NA	NA	0.000	0.000	0.000	NA	0.000	NA	0.000	NA	NA	NA	NA
rs531900250	A	0.000	NA	0.000	0.000	NA	NA	NA	0.000	NA	NA	NA	0.006	0.000	0.000	NA	0.000	NA	0.000	NA	NA	NA	NA
rs577921461	T	0.000	NA	0.000	0.000	NA	NA	NA	0.000	NA	NA	NA	0.000	0.000	0.000	NA	0.000	NA	0.000	NA	NA	NA	NA
rs201403607	A	0.000	NA	0.000	0.000	NA	NA	NA	0.000	NA	NA	NA	0.006	0.000	0.000	NA	0.000	NA	0.000	NA	NA	NA	NA
rs7255181	G	0.000	NA	0.000	0.000	NA	NA	NA	0.020	NA	NA	NA	0.018	0.000	0.000	NA	0.000	NA	0.005	NA	NA	NA	NA
rs141583892	T	0.000	NA	0.000	0.000	NA	NA	NA	0.015	NA	NA	NA	0.000	0.000	0.000	NA	0.000	NA	0.000	NA	NA	NA	NA
rs113790346	G	NA	NA	NA	NA	NA	NA	NA	NA	NA	NA	NA	NA	NA	NA	NA	NA	NA	NA	NA	NA	NA	NA
rs551388840	T	0.000	NA	0.000	0.000	NA	NA	NA	0.000	NA	NA	NA	0.000	0.000	0.000	NA	0.005	NA	0.000	NA	NA	NA	NA
rs542552024	A	0.000	NA	0.000	0.000	NA	NA	NA	0.000	NA	NA	NA	0.000	0.000	0.000	NA	0.000	NA	0.000	NA	NA	NA	NA
rs552594392	A	0.000	NA	0.000	0.000	NA	NA	NA	0.000	NA	NA	NA	0.000	0.000	0.000	NA	0.005	NA	0.000	NA	NA	NA	NA
rs182826947	A	0.000	NA	0.000	0.000	NA	NA	NA	0.000	NA	NA	NA	0.000	0.008	0.000	NA	0.000	NA	0.000	NA	NA	NA	NA
rs188799878	A	0.000	NA	0.005	0.000	NA	NA	NA	0.000	NA	NA	NA	0.000	0.000	0.000	NA	0.000	NA	0.000	NA	NA	NA	NA
rs11879178	A	0.358	NA	0.500	0.465	NA	NA	NA	0.374	NA	NA	NA	0.300	0.406	0.353	NA	0.396	NA	0.226	NA	NA	NA	NA
rs28993669	C	NA	NA	NA	NA	NA	NA	NA	NA	NA	NA	NA	NA	NA	NA	0.000	NA	NA	NA	NA	NA	0.000	NA
rs191246732	A	0.000	NA	0.000	0.000	NA	NA	NA	0.000	NA	NA	NA	0.000	0.008	0.000	NA	0.000	NA	0.000	NA	NA	NA	NA
rs551261955	T	0.000	NA	0.000	0.005	NA	NA	NA	0.000	NA	NA	NA	0.000	0.000	0.000	NA	0.000	NA	0.000	NA	NA	NA	NA
rs146186991	A	0.000	NA	0.000	0.000	NA	NA	NA	0.000	NA	NA	NA	0.000	0.000	0.000	0.042	0.000	NA	0.005	NA	NA	0.000	NA
rs544039773	T	0.000	NA	0.000	0.000	NA	NA	NA	0.000	NA	NA	NA	0.000	0.000	0.006	NA	0.000	NA	0.000	NA	NA	NA	NA
rs554365832	A	0.000	NA	0.000	0.000	NA	NA	NA	0.000	NA	NA	NA	0.000	0.000	0.000	NA	0.010	NA	0.000	NA	NA	NA	NA
rs138039913	T	0.000	NA	0.005	0.010	NA	NA	NA	0.000	NA	NA	NA	0.000	0.000	0.000	NA	0.000	NA	0.000	NA	NA	NA	NA
rs541089761	A	0.000	NA	0.000	0.000	NA	NA	NA	0.005	NA	NA	NA	0.006	0.000	0.000	NA	0.000	NA	0.000	NA	NA	NA	NA
rs28921985	A	0.000	NA	0.000	0.000	NA	NA	NA	0.030	NA	NA	NA	0.047	0.000	0.000	0.000	0.000	NA	0.000	NA	NA	0.000	NA
rs529207268	G	0.000	NA	0.000	0.000	NA	NA	NA	0.000	NA	NA	NA	0.000	0.000	0.000	NA	0.000	NA	0.000	NA	NA	NA	NA
rs190217060	A	0.000	NA	0.000	0.000	NA	NA	NA	0.005	NA	NA	NA	0.000	0.000	0.000	NA	0.000	NA	0.000	NA	NA	NA	NA
rs552965493	T	0.000	NA	0.000	0.000	NA	NA	NA	0.000	NA	NA	NA	0.000	0.000	0.000	NA	0.000	NA	0.000	NA	NA	NA	NA
rs545068935	G	0.000	NA	0.000	0.000	NA	NA	NA	0.000	NA	NA	NA	0.006	0.000	0.000	NA	0.000	NA	0.005	NA	NA	NA	NA
rs540123195	A	0.000	NA	0.000	0.000	NA	NA	NA	0.000	NA	NA	NA	0.000	0.000	0.000	NA	0.000	NA	0.000	NA	NA	NA	NA
rs12611131	T	0.324	NA	0.125	0.298	NA	NA	NA	0.177	NA	NA	NA	0.247	0.094	0.041	NA	0.208	NA	0.115	NA	NA	NA	NA
rs200467897	T	0.000	NA	0.000	0.000	NA	NA	NA	0.000	NA	NA	NA	0.000	0.000	0.000	NA	0.000	NA	0.000	NA	NA	NA	NA
rs28992469	T	0.000	NA	0.000	0.000	NA	NA	NA	0.000	NA	NA	NA	0.000	0.000	0.000	0.006	0.000	NA	0.000	NA	NA	0.000	NA
rs2072307	C	0.284	NA	0.082	0.222	NA	NA	NA	0.192	NA	NA	NA	0.247	0.086	0.041	NA	0.203	NA	0.120	NA	NA	NA	NA
rs535566277	A	0.000	NA	0.000	0.000	NA	NA	NA	0.000	NA	NA	NA	0.000	0.000	0.000	NA	0.000	NA	0.000	NA	NA	NA	NA
rs145461969	T	0.000	NA	0.000	0.000	NA	NA	NA	0.000	NA	NA	NA	0.000	0.000	0.000	NA	0.000	NA	0.000	NA	NA	NA	NA
rs568389785	T	0.000	NA	0.000	0.000	NA	NA	NA	0.000	NA	NA	NA	0.000	0.000	0.006	NA	0.000	NA	0.000	NA	NA	NA	NA
rs56043551	T	NA	NA	NA	NA	NA	NA	NA	NA	NA	NA	NA	NA	NA	NA	NA	NA	NA	NA	NA	NA	NA	NA

SNP	Allele	ITU	Jacarus	JPT	KHV	Lamas	Lambayeque	Lima	LWK	Matses	Moche	Moquegua	MSL	MXL	PEL	Pelotas	PJL	Puno	PUR	Qeros	Quechuas	Salvador	Shimaa
rs191693960	T	0.000	NA	0.000	0.000	NA	NA	NA	0.010	NA	NA	NA	0.006	0.000	0.000	NA	0.000	NA	0.005	NA	NA	NA	NA
rs28921968	G	NA	NA	NA	NA	NA	NA	NA	NA	NA	NA	NA	NA	NA	NA	0.017	NA	NA	NA	NA	NA	0.044	NA
rs549983564	T	0.000	NA	0.000	0.000	NA	NA	NA	0.005	NA	NA	NA	0.000	0.000	0.000	NA	0.000	NA	0.000	NA	NA	NA	NA
rs200630157	T	0.000	NA	0.000	0.000	NA	NA	NA	0.000	NA	NA	NA	0.000	0.000	0.000	NA	0.000	NA	0.000	NA	NA	NA	NA
rs550013368	A	0.000	NA	0.000	0.000	NA	NA	NA	0.010	NA	NA	NA	0.000	0.000	0.000	NA	0.000	NA	0.005	NA	NA	NA	NA
rs191389346	T	0.020	NA	0.000	0.000	NA	NA	NA	0.000	NA	NA	NA	0.000	0.000	0.000	NA	0.000	NA	0.000	NA	NA	NA	NA
rs28921983	G	NA	NA	NA	NA	NA	NA	NA	NA	NA	NA	NA	NA	NA	NA	0.000	NA	NA	NA	NA	NA	0.000	NA
rs564546624	T	0.000	NA	0.000	0.000	NA	NA	NA	0.000	NA	NA	NA	0.000	0.000	0.000	NA	0.000	NA	0.000	NA	NA	NA	NA
rs150791055	T	0.000	NA	0.000	0.000	NA	NA	NA	0.005	NA	NA	NA	0.000	0.000	0.000	NA	0.000	NA	0.000	NA	NA	NA	NA
rs202135216	T	0.000	NA	0.000	0.000	NA	NA	NA	0.005	NA	NA	NA	0.047	0.008	0.006	NA	0.000	NA	0.000	NA	NA	NA	NA
rs575212330	T	0.010	NA	0.000	0.000	NA	NA	NA	0.000	NA	NA	NA	0.000	0.000	0.000	NA	0.000	NA	0.000	NA	NA	NA	NA
rs559570766	T	0.000	NA	0.000	0.000	NA	NA	NA	0.000	NA	NA	NA	0.000	0.000	0.000	NA	0.000	NA	0.000	NA	NA	NA	NA
rs151237303	T	0.284	NA	0.125	0.298	NA	NA	NA	0.172	NA	NA	NA	0.247	0.094	0.041	NA	0.203	NA	0.115	NA	NA	NA	NA
rs369186206	A	0.000	NA	0.000	0.000	NA	NA	NA	0.000	NA	NA	NA	0.000	0.000	0.000	NA	0.000	NA	0.000	NA	NA	NA	NA
rs28921975	A	0.000	NA	0.000	0.000	NA	NA	NA	0.020	NA	NA	NA	0.000	0.000	0.000	NA	0.000	NA	0.000	NA	NA	NA	NA
rs538891893	G	0.000	NA	0.000	0.000	NA	NA	NA	0.000	NA	NA	NA	0.000	0.000	0.000	NA	0.000	NA	0.000	NA	NA	NA	NA
rs566302789	T	0.000	NA	0.000	0.000	NA	NA	NA	0.000	NA	NA	NA	0.000	0.016	0.018	NA	0.000	NA	0.014	NA	NA	NA	NA
rs189865097	A	0.000	NA	0.000	0.000	NA	NA	NA	0.000	NA	NA	NA	0.000	0.000	0.000	NA	0.000	NA	0.005	NA	NA	NA	NA
rs2283570	T	0.328	NA	0.164	0.298	NA	NA	NA	0.172	NA	NA	NA	0.247	0.102	0.041	NA	0.214	NA	0.130	NA	NA	NA	NA
rs181004617	A	0.000	NA	0.000	0.000	NA	NA	NA	0.000	NA	NA	NA	0.000	0.000	0.000	NA	0.000	NA	0.005	NA	NA	NA	NA
rs2283574	A	0.284	NA	0.125	0.293	NA	NA	NA	0.172	NA	NA	NA	0.247	0.094	0.041	NA	0.203	NA	0.115	NA	NA	NA	NA
rs553131488	A	0.000	NA	0.000	0.000	NA	NA	NA	0.000	NA	NA	NA	0.000	0.000	0.000	NA	0.000	NA	0.000	NA	NA	NA	NA
rs200432051	T	0.000	NA	0.000	0.000	NA	NA	NA	0.000	NA	NA	NA	0.000	0.000	0.000	NA	0.000	NA	0.000	NA	NA	NA	NA
rs569279461	T	0.000	NA	0.000	0.000	NA	NA	NA	0.000	NA	NA	NA	0.000	0.000	0.000	NA	0.000	NA	0.000	NA	NA	NA	NA
rs112785861	A	0.000	NA	0.000	0.000	NA	NA	NA	0.056	NA	NA	NA	0.076	0.000	0.000	NA	0.000	NA	0.005	NA	NA	NA	NA
rs529813357	T	0.000	NA	0.000	0.000	NA	NA	NA	0.000	NA	NA	NA	0.000	0.000	0.000	NA	0.000	NA	0.000	NA	NA	NA	NA
rs563512179	A	0.000	NA	0.000	0.000	NA	NA	NA	0.000	NA	NA	NA	0.000	0.000	0.000	NA	0.000	NA	0.000	NA	NA	NA	NA
rs201881530	G	0.000	NA	0.000	0.000	NA	NA	NA	0.000	NA	NA	NA	0.000	0.000	0.000	NA	0.000	NA	0.000	NA	NA	NA	NA
rs2283571	C	0.510	NA	0.389	0.480	NA	NA	NA	0.278	NA	NA	NA	0.259	0.391	0.288	NA	0.432	NA	0.563	NA	NA	NA	NA
rs116073835	A	0.000	NA	0.000	0.000	NA	NA	NA	0.040	NA	NA	NA	0.076	0.000	0.000	NA	0.000	NA	0.005	NA	NA	NA	NA
rs368973405	T	0.000	NA	0.000	0.000	NA	NA	NA	0.005	NA	NA	NA	0.000	0.000	0.000	NA	0.000	NA	0.000	NA	NA	NA	NA
rs553337242	T	0.000	NA	0.000	0.000	NA	NA	NA	0.000	NA	NA	NA	0.000	0.000	0.000	NA	0.000	NA	0.000	NA	NA	NA	NA
rs577310345	C	0.000	NA	0.000	0.000	NA	NA	NA	0.000	NA	NA	NA	0.000	0.000	0.000	NA	0.000	NA	0.000	NA	NA	NA	NA
rs538315599	A	0.000	NA	0.000	0.000	NA	NA	NA	0.000	NA	NA	NA	0.000	0.000	0.000	NA	0.000	NA	0.000	NA	NA	NA	NA
rs556900373	A	0.000	NA	0.000	0.000	NA	NA	NA	0.000	NA	NA	NA	0.000	0.000	0.000	NA	0.000	NA	0.000	NA	NA	NA	NA
rs534224219	T	0.005	NA	0.000	0.000	NA	NA	NA	0.000	NA	NA	NA	0.000	0.000	0.000	NA	0.000	NA	0.000	NA	NA	NA	NA
rs28915408	A	0.020	NA	0.000	0.000	NA	NA	NA	0.000	NA	NA	NA	0.000	0.000	0.000	NA	0.021	NA	0.005	NA	NA	NA	NA
rs538902991	T	0.000	NA	0.000	0.005	NA	NA	NA	0.000	NA	NA	NA	0.000	0.000	0.000	NA	0.000	NA	0.000	NA	NA	NA	NA
rs200237260	T	0.000	NA	0.000	0.015	NA	NA	NA	0.000	NA	NA	NA	0.000	0.000	0.000	NA	0.000	NA	0.000	NA	NA	NA	NA
rs28992480	A	0.000	NA	0.000	0.000	NA	NA	NA	0.010	NA	NA	NA	0.035	0.008	0.000	0.000	0.000	NA	0.000	NA	NA	0.000	NA
rs12979852	T	0.093	NA	0.010	0.015	NA	NA	NA	0.273	NA	NA	NA	0.277	0.047	0.035	NA	0.146	NA	0.091	NA	NA	NA	NA
rs200853058	T	0.000	NA	0.000	0.000	NA	NA	NA	0.000	NA	NA	NA	0.000	0.000	0.000	NA	0.000	NA	0.000	NA	NA	NA	NA
rs560866175	A	0.000	NA	0.000	0.000	NA	NA	NA	0.000	NA	NA	NA	0.000	0.000	0.000	NA	0.005	NA	0.000	NA	NA	NA	NA

SNP	Allele	ITU	Jacarus	JPT	KHV	Lamas	Lambayeque	Lima	LWK	Matses	Moche	Moquegua	MSL	MXL	PEL	Pelotas	PJL	Puno	PUR	Qeros	Quechuas	Salvador	Shimaa
rs186833702	A	0.000	NA	0.005	0.000	NA	NA	NA	0.000	NA	NA	NA	0.000	0.000	0.000	NA	0.000	NA	0.000	NA	NA	NA	NA
rs113907190	G	NA	NA	NA	NA	NA	NA	NA	NA	NA	NA	NA	NA	NA	NA	NA	NA	NA	NA	NA	NA	NA	NA
rs28921990	C	0.284	NA	0.125	0.298	NA	NA	NA	0.172	NA	NA	NA	0.247	0.094	0.041	NA	0.203	NA	0.115	NA	NA	NA	NA
rs577273590	G	0.000	NA	0.000	0.000	NA	NA	NA	0.000	NA	NA	NA	0.000	0.000	0.000	NA	0.000	NA	0.000	NA	NA	NA	NA
rs536730099	A	0.000	NA	0.005	0.000	NA	NA	NA	0.000	NA	NA	NA	0.000	0.000	0.000	NA	0.000	NA	0.000	NA	NA	NA	NA
rs143543245	T	0.000	NA	0.000	0.000	NA	NA	NA	0.010	NA	NA	NA	0.006	0.000	0.000	NA	0.000	NA	0.005	NA	NA	NA	NA
rs202113312	C	NA	NA	NA	NA	NA	NA	NA	NA	NA	NA	NA	NA	NA	NA	NA	NA	NA	NA	NA	NA	NA	NA
rs565922179	T	0.000	NA	0.000	0.000	NA	NA	NA	0.000	NA	NA	NA	0.000	0.000	0.000	NA	0.000	NA	0.000	NA	NA	NA	NA
rs113033881	C	0.284	NA	0.130	0.293	NA	NA	NA	0.263	NA	NA	NA	0.294	0.094	0.053	NA	0.203	NA	0.125	NA	NA	NA	NA
rs565509602	T	0.000	NA	0.000	0.000	NA	NA	NA	0.000	NA	NA	NA	0.006	0.000	0.000	NA	0.000	NA	0.000	NA	NA	NA	NA
rs552797010	A	0.000	NA	0.000	0.000	NA	NA	NA	0.000	NA	NA	NA	0.000	0.000	0.000	NA	0.000	NA	0.000	NA	NA	NA	NA
rs534935683	T	0.000	NA	0.000	0.005	NA	NA	NA	0.000	NA	NA	NA	0.000	0.000	0.000	NA	0.000	NA	0.000	NA	NA	NA	NA
rs367579969	T	0.000	NA	0.005	0.056	NA	NA	NA	0.000	NA	NA	NA	0.000	0.000	0.000	NA	0.000	NA	0.000	NA	NA	NA	NA
rs568509871	C	0.000	NA	0.000	0.000	NA	NA	NA	0.000	NA	NA	NA	0.000	0.000	0.000	NA	0.000	NA	0.000	NA	NA	NA	NA
rs565826251	A	0.000	NA	0.000	0.000	NA	NA	NA	0.000	NA	NA	NA	0.000	0.000	0.000	NA	0.000	NA	0.000	NA	NA	NA	NA
rs542552123	A	0.000	NA	0.000	0.000	NA	NA	NA	0.000	NA	NA	NA	0.000	0.000	0.000	NA	0.000	NA	0.000	NA	NA	NA	NA
rs28992471	A	0.284	NA	0.125	0.293	NA	NA	NA	0.172	NA	NA	NA	0.247	0.094	0.041	NA	0.203	NA	0.115	NA	NA	NA	NA
rs563724259	T	0.000	NA	0.000	0.000	NA	NA	NA	0.000	NA	NA	NA	0.000	0.000	0.000	NA	0.000	NA	0.000	NA	NA	NA	NA
rs28921988	T	0.000	NA	0.000	0.000	NA	NA	NA	0.010	NA	NA	NA	0.018	0.008	0.000	NA	0.000	NA	0.000	NA	NA	NA	NA
rs560442683	T	0.000	NA	0.000	0.000	NA	NA	NA	0.000	NA	NA	NA	0.000	0.000	0.000	NA	0.000	NA	0.000	NA	NA	NA	NA
rs537692667	A	0.000	NA	0.000	0.000	NA	NA	NA	0.000	NA	NA	NA	0.000	0.000	0.000	NA	0.000	NA	0.005	NA	NA	NA	NA
rs28921971	T	0.074	NA	0.005	0.015	NA	NA	NA	0.005	NA	NA	NA	0.000	0.133	0.329	0.082	0.125	NA	0.101	NA	NA	0.090	NA
rs572811560	A	0.000	NA	0.000	0.000	NA	NA	NA	0.000	NA	NA	NA	0.000	0.000	0.000	NA	0.000	NA	0.000	NA	NA	NA	NA
rs28992483	T	0.284	NA	0.130	0.293	NA	NA	NA	0.172	NA	NA	NA	0.247	0.094	0.041	NA	0.203	NA	0.115	NA	NA	NA	NA
rs547376405	G	0.000	NA	0.000	0.000	NA	NA	NA	0.010	NA	NA	NA	0.000	0.008	0.000	NA	0.000	NA	0.000	NA	NA	NA	NA
rs138748626	T	0.000	NA	0.019	0.015	NA	NA	NA	0.000	NA	NA	NA	0.000	0.000	0.000	NA	0.000	NA	0.000	NA	NA	NA	NA
rs528453463	A	0.005	NA	0.000	0.000	NA	NA	NA	0.000	NA	NA	NA	0.000	0.000	0.000	NA	0.005	NA	0.000	NA	NA	NA	NA
rs552838011	G	0.000	NA	0.000	0.000	NA	NA	NA	0.000	NA	NA	NA	0.000	0.000	0.000	NA	0.000	NA	0.000	NA	NA	NA	NA
rs544905487	T	0.000	NA	0.000	0.000	NA	NA	NA	0.000	NA	NA	NA	0.000	0.000	0.000	NA	0.000	NA	0.000	NA	NA	NA	NA
rs139995866	A	0.000	NA	0.014	0.010	NA	NA	NA	0.000	NA	NA	NA	0.000	0.000	0.000	NA	0.000	NA	0.000	NA	NA	NA	NA
rs556660637	A	0.000	NA	0.000	0.000	NA	NA	NA	0.000	NA	NA	NA	0.000	0.000	0.000	NA	0.000	NA	0.000	NA	NA	NA	NA
rs559190146	A	0.000	NA	0.000	0.000	NA	NA	NA	0.000	NA	NA	NA	0.000	0.000	0.000	NA	0.000	NA	0.000	NA	NA	NA	NA
rs544661215	T	0.000	NA	0.000	0.000	NA	NA	NA	0.000	NA	NA	NA	0.000	0.000	0.000	NA	0.000	NA	0.000	NA	NA	NA	NA
rs2238542	T	0.284	NA	0.125	0.298	NA	NA	NA	0.172	NA	NA	NA	0.247	0.094	0.041	0.099	0.203	NA	0.115	NA	NA	0.189	NA
rs571648795	T	0.000	NA	0.000	0.000	NA	NA	NA	0.000	NA	NA	NA	0.006	0.000	0.000	NA	0.000	NA	0.000	NA	NA	NA	NA
rs567013917	A	0.000	NA	0.000	0.000	NA	NA	NA	0.000	NA	NA	NA	0.000	0.000	0.000	NA	0.000	NA	0.000	NA	NA	NA	NA
rs187005996	T	0.000	NA	0.000	0.000	NA	NA	NA	0.025	NA	NA	NA	0.006	0.000	0.000	NA	0.000	NA	0.010	NA	NA	NA	NA
rs28473389	T	0.080	NA	0.005	0.016	NA	NA	NA	0.007	NA	NA	NA	0.000	0.133	0.329	NA	0.122	NA	0.105	NA	NA	NA	NA
rs71334826	C	0.000	NA	0.000	0.000	NA	NA	NA	0.030	NA	NA	NA	0.071	0.000	0.000	NA	0.000	NA	0.000	NA	NA	NA	NA
rs543090783	A	0.000	NA	0.000	0.000	NA	NA	NA	0.000	NA	NA	NA	0.000	0.000	0.000	NA	0.000	NA	0.000	NA	NA	NA	NA
rs540356623	A	0.000	NA	0.000	0.000	NA	NA	NA	0.000	NA	NA	NA	0.000	0.000	0.000	NA	0.000	NA	0.000	NA	NA	NA	NA
rs564651283	T	0.000	NA	0.000	0.000	NA	NA	NA	0.000	NA	NA	NA	0.000	0.000	0.000	NA	0.000	NA	0.000	NA	NA	NA	NA
rs12609610	G	0.005	0.000	0.029	0.010	0.000	0.000	0.000	0.000	0.000	0.033	0.000	0.000	0.000	0.006	0.000	0.000	0.000	0.000	0.000	0.000	0.000	0.000

SNP	Allele	ITU	Jacarus	JPT	KHV	Lamas	Lambayeque	Lima	LWK	Matses	Moche	Moquegua	MSL	MXL	PEL	Pelotas	PJL	Puno	PUR	Qeros	Quechuas	Salvador	Shimaa
rs572636722	T	0.000	NA	0.000	0.005	NA	NA	NA	0.000	NA	NA	NA	0.000	0.000	0.000	NA	0.000	NA	0.000	NA	NA	NA	NA
rs10416339	A	0.529	NA	0.418	0.475	NA	NA	NA	0.278	NA	NA	NA	0.259	0.383	0.294	NA	0.453	NA	0.563	NA	NA	NA	NA
rs56067110	A	0.000	NA	0.000	0.000	NA	NA	NA	0.035	NA	NA	NA	0.018	0.008	0.000	NA	0.000	NA	0.000	NA	NA	NA	NA
rs552690398	A	0.000	NA	0.000	0.000	NA	NA	NA	0.000	NA	NA	NA	0.000	0.000	0.006	NA	0.000	NA	0.000	NA	NA	NA	NA
rs72972182	C	0.074	NA	0.005	0.015	NA	NA	NA	0.005	NA	NA	NA	0.000	0.133	0.329	NA	0.125	NA	0.101	NA	NA	NA	NA
rs143454800	G	0.000	NA	0.005	0.010	NA	NA	NA	0.000	NA	NA	NA	0.000	0.000	0.000	NA	0.000	NA	0.000	NA	NA	NA	NA
rs539563939	A	0.000	NA	0.000	0.000	NA	NA	NA	0.005	NA	NA	NA	0.000	0.000	0.000	NA	0.000	NA	0.000	NA	NA	NA	NA
rs368584754	A	0.000	NA	0.000	0.000	NA	NA	NA	0.010	NA	NA	NA	0.006	0.000	0.000	NA	0.000	NA	0.000	NA	NA	NA	NA
rs529198553	A	0.000	NA	0.000	0.000	NA	NA	NA	0.000	NA	NA	NA	0.000	0.000	0.000	NA	0.000	NA	0.000	NA	NA	NA	NA
rs557853268	A	0.000	NA	0.000	0.000	NA	NA	NA	0.000	NA	NA	NA	0.000	0.008	0.000	NA	0.000	NA	0.000	NA	NA	NA	NA
rs533240475	A	0.000	NA	0.000	0.000	NA	NA	NA	0.000	NA	NA	NA	0.006	0.000	0.000	NA	0.000	NA	0.000	NA	NA	NA	NA
rs202082105	A	0.000	NA	0.000	0.000	NA	NA	NA	0.000	NA	NA	NA	0.000	0.000	0.000	NA	0.000	NA	0.000	NA	NA	NA	NA

bold: functionally relevant SNPs found in our databases

Table S3-A – BSG allele frequencies continuation. Bold = functionally relevant SNPs found in our databases; NA = missing data

SNP	Allele	Shipibo	STU	Tacna	Tallanes	Trujillo	TSI	Tumbes	Uros	YRI
**rs8259**	**T**	**NA**	**NA**	**NA**	**NA**	**NA**	**NA**	**NA**	**NA**	**NA**
**rs6757**	**C**	**0.063**	**0.275**	**0.045**	**0.100**	**0.146**	**0.098**	**0.061**	**0.063**	**0.171**
**rs8637**	**A**	**NA**	**0.201**	**NA**	**NA**	**NA**	**0.472**	**NA**	**NA**	**0.009**
**rs12608994**	**T**	**NA**	**0.289**	**NA**	**NA**	**NA**	**0.131**	**NA**	**NA**	**0.190**
**rs1803202**	**T**	**NA**	**0.294**	**NA**	**NA**	**NA**	**0.131**	**NA**	**NA**	**0.167**
**rs11879069**	**A**	**0.125**	**0.324**	**0.432**	**0.257**	**0.396**	**0.210**	**0.258**	**0.375**	**0.301**
**rs4919862**	**T**	**NA**	**0.088**	**NA**	**NA**	**NA**	**0.290**	**NA**	**NA**	**0.009**
**rs6758**	**A**	**NA**	**0.275**	**NA**	**NA**	**NA**	**0.098**	**NA**	**NA**	**0.167**
**rs28992474**	**T**	**NA**	**0.289**	**NA**	**NA**	**NA**	**0.131**	**NA**	**NA**	**0.167**
**rs2072310**	**T**	**NA**	**0.319**	**NA**	**NA**	**NA**	**0.220**	**NA**	**NA**	**0.301**
**rs3764937**	**A**	**NA**	**0.304**	**NA**	**NA**	**NA**	**0.126**	**NA**	**NA**	**0.403**
**rs4682**	**C**	**NA**	**0.324**	**NA**	**NA**	**NA**	**0.215**	**NA**	**NA**	**0.301**
**rs2072309**	**A**	**0.125**	**0.289**	**0.455**	**0.258**	**0.375**	**0.224**	**0.273**	**0.375**	**0.296**
**rs2074962**	**A**	**NA**	**0.211**	**NA**	**NA**	**NA**	**0.519**	**NA**	**NA**	**0.060**
rs568500243	T	NA	0.000	NA	NA	NA	0.000	NA	NA	0.000
rs565860293	A	NA	0.000	NA	NA	NA	0.000	NA	NA	0.000
rs369456038	T	NA	0.000	NA	NA	NA	0.000	NA	NA	0.000
rs2108832	C	NA	NA	NA	NA	NA	NA	NA	NA	NA
rs193268910	T	NA	0.000	NA	NA	NA	0.000	NA	NA	0.005
rs576903171	A	NA	0.000	NA	NA	NA	0.009	NA	NA	0.000
rs376295294	A	NA	0.000	NA	NA	NA	0.000	NA	NA	0.000
rs188261956	A	NA	0.000	NA	NA	NA	0.000	NA	NA	0.000
rs140055452	T	NA	0.000	NA	NA	NA	0.000	NA	NA	0.009
rs552833814	T	NA	0.000	NA	NA	NA	0.005	NA	NA	0.000
rs531651313	A	NA	0.005	NA	NA	NA	0.000	NA	NA	0.000
rs551644758	G	NA	0.000	NA	NA	NA	0.000	NA	NA	0.000
rs2238540	T	NA	0.289	NA	NA	NA	0.131	NA	NA	0.167

SNP	Allele	Shipibo	STU	Tacna	Tallanes	Trujillo	TSI	Tumbes	Uros	YRI
rs189460104	A	NA	0.000	NA	NA	NA	0.000	NA	NA	0.005
rs118187381	A	NA	0.000	NA	NA	NA	0.014	NA	NA	0.009
rs183979701	G	NA	0.005	NA	NA	NA	0.000	NA	NA	0.000
rs10593023	C	NA	0.324	NA	NA	NA	0.210	NA	NA	0.296
rs561149009	A	NA	0.000	NA	NA	NA	0.000	NA	NA	0.000
rs543884178	T	NA	0.000	NA	NA	NA	0.000	NA	NA	0.000
rs544074414	A	NA	0.000	NA	NA	NA	0.000	NA	NA	0.000
rs551225501	G	NA	0.000	NA	NA	NA	0.000	NA	NA	0.014
rs28915407	T	NA	0.005	NA	NA	NA	0.014	NA	NA	0.000
rs28992486	G	NA	0.000	NA	NA	NA	0.000	NA	NA	0.241
rs375283966	C	NA	0.000	NA	NA	NA	0.000	NA	NA	0.000
rs2283572	T	NA	0.324	NA	NA	NA	0.210	NA	NA	0.296
rs12609063	A	NA	0.289	NA	NA	NA	0.131	NA	NA	0.167
rs528163900	A	NA	0.000	NA	NA	NA	0.000	NA	NA	0.000
rs188930171	C	NA	0.000	NA	NA	NA	0.000	NA	NA	0.000
rs149894645	C	NA	0.000	NA	NA	NA	0.000	NA	NA	0.000
rs558467385	A	NA	0.000	NA	NA	NA	0.000	NA	NA	0.000
rs542497048	T	NA	0.000	NA	NA	NA	0.000	NA	NA	0.000
rs547801807	C	NA	0.000	NA	NA	NA	0.000	NA	NA	0.056
rs371927933	T	NA	0.000	NA	NA	NA	0.000	NA	NA	0.000
rs556218809	A	NA	0.000	NA	NA	NA	0.000	NA	NA	0.000
rs1138150	T	NA	0.534	NA	NA	NA	0.664	NA	NA	0.208
rs28921974	C	NA	0.142	NA	NA	NA	0.042	NA	NA	0.028
rs568364520	A	NA	0.000	NA	NA	NA	0.000	NA	NA	0.000
rs2283573	T	NA	0.289	NA	NA	NA	0.131	NA	NA	0.167
rs562293196	A	NA	0.000	NA	NA	NA	0.000	NA	NA	0.000
rs11458913	C	NA	0.245	NA	NA	NA	0.537	NA	NA	0.065
rs114359237	T	NA	0.000	NA	NA	NA	0.000	NA	NA	0.014
rs28989774	A	NA	0.000	NA	NA	NA	0.000	NA	NA	0.157
rs189315508	A	NA	0.000	NA	NA	NA	0.000	NA	NA	0.000
rs138409636	T	NA	0.000	NA	NA	NA	0.000	NA	NA	0.000
rs375925694	T	NA	0.000	NA	NA	NA	0.000	NA	NA	0.000
rs567062140	A	NA	0.000	NA	NA	NA	0.000	NA	NA	0.000
rs574441118	A	NA	0.000	NA	NA	NA	0.000	NA	NA	0.000
rs1049676	T	NA	0.020	NA	NA	NA	0.042	NA	NA	0.019
rs183656586	A	NA	0.000	NA	NA	NA	0.000	NA	NA	0.000
rs574480082	G	NA	0.000	NA	NA	NA	0.000	NA	NA	0.000
rs565755933	T	NA	0.000	NA	NA	NA	0.000	NA	NA	0.000
rs554979181	A	NA	0.000	NA	NA	NA	0.000	NA	NA	0.000
rs146784114	A	NA	NA	NA	NA	NA	NA	NA	NA	NA
rs564593406	A	NA	0.000	NA	NA	NA	0.000	NA	NA	0.000
rs535528596	T	NA	0.015	NA	NA	NA	0.000	NA	NA	0.000
rs185996213	C	NA	0.020	NA	NA	NA	0.005	NA	NA	0.019

SNP	Allele	Shipibo	STU	Tacna	Tallanes	Trujillo	TSI	Tumbes	Uros	YRI
rs185382187	A	NA	0.000	NA	NA	NA	0.000	NA	NA	0.000
rs2072308	C	NA	0.294	NA	NA	NA	0.131	NA	NA	0.181
rs141043764	A	NA	0.000	NA	NA	NA	0.000	NA	NA	0.005
rs185250119	A	NA	0.000	NA	NA	NA	0.000	NA	NA	0.000
rs147511542	C	NA	0.000	NA	NA	NA	0.000	NA	NA	0.000
rs573330257	G	NA	0.000	NA	NA	NA	0.000	NA	NA	0.000
rs542924300	T	NA	0.000	NA	NA	NA	0.000	NA	NA	0.000
rs543561666	A	NA	0.000	NA	NA	NA	0.000	NA	NA	0.009
rs8110980	T	0.125	0.324	0.409	0.257	0.370	0.210	0.273	0.375	0.296
rs200045153	G	NA	0.000	NA	NA	NA	0.019	NA	NA	0.000
rs10422916	T	NA	0.206	NA	NA	NA	0.486	NA	NA	0.009
rs568226979	T	NA	0.000	NA	NA	NA	0.000	NA	NA	0.000
rs550149267	T	NA	0.000	NA	NA	NA	0.000	NA	NA	0.000
rs182848936	T	NA	0.000	NA	NA	NA	0.000	NA	NA	0.000
rs535528658	T	NA	0.000	NA	NA	NA	0.000	NA	NA	0.000
rs537007790	T	NA	0.000	NA	NA	NA	0.000	NA	NA	0.000
rs571095325	A	NA	0.000	NA	NA	NA	0.000	NA	NA	0.032
rs548874736	T	NA	0.000	NA	NA	NA	0.000	NA	NA	0.014
rs546527955	T	NA	0.000	NA	NA	NA	0.000	NA	NA	0.000
rs533122473	T	NA	0.000	NA	NA	NA	0.000	NA	NA	0.000
rs28992481	C	NA	0.000	NA	NA	NA	0.000	NA	NA	0.023
rs547511336	A	NA	0.000	NA	NA	NA	0.000	NA	NA	0.000
rs117305685	T	NA	0.000	NA	NA	NA	0.005	NA	NA	0.000
rs28921987	A	NA	0.000	NA	NA	NA	0.000	NA	NA	0.000
rs60851035	A	NA	0.000	NA	NA	NA	0.000	NA	NA	0.037
rs570887990	T	NA	0.000	NA	NA	NA	0.000	NA	NA	0.000
rs556084263	T	NA	0.000	NA	NA	NA	0.000	NA	NA	0.000
rs142283391	T	NA	0.000	NA	NA	NA	0.000	NA	NA	0.005
rs375240640	A	NA	0.074	NA	NA	NA	0.056	NA	NA	0.014
rs114264435	T	NA	0.000	NA	NA	NA	0.000	NA	NA	0.023
rs553170407	C	NA	0.015	NA	NA	NA	0.000	NA	NA	0.000
rs541302606	A	NA	0.000	NA	NA	NA	0.000	NA	NA	0.000
rs529379861	A	NA	0.000	NA	NA	NA	0.000	NA	NA	0.000
rs112152563	T	NA	NA	NA	NA	NA	NA	NA	NA	NA
rs557135170	A	NA	0.000	NA	NA	NA	0.000	NA	NA	0.000
rs557199346	A	NA	0.000	NA	NA	NA	0.000	NA	NA	0.000
rs14173	G	NA	0.000	NA	NA	NA	0.000	NA	NA	0.005
rs531691645	A	NA	0.000	NA	NA	NA	0.000	NA	NA	0.000
rs376756373	A	NA	0.000	NA	NA	NA	0.000	NA	NA	0.000
rs7253615	G	NA	0.245	NA	NA	NA	0.528	NA	NA	0.065
rs566889863	C	NA	0.000	NA	NA	NA	0.000	NA	NA	0.000
rs369996586	C	NA	0.000	NA	NA	NA	0.000	NA	NA	0.000
rs200704727	CA	NA	0.289	NA	NA	NA	0.131	NA	NA	0.167

SNP	Allele	Shipibo	STU	Tacna	Tallanes	Trujillo	TSI	Tumbes	Uros	YRI
rs539709135	G	NA	0.353	NA	NA	NA	0.136	NA	NA	0.153
rs28921981	A	NA	0.157	NA	NA	NA	0.079	NA	NA	0.088
rs7252521	T	NA	0.000	NA	NA	NA	0.000	NA	NA	0.005
rs562167455	G	NA	0.000	NA	NA	NA	0.000	NA	NA	0.000
rs539126301	A	NA	0.000	NA	NA	NA	0.000	NA	NA	0.000
rs570527862	G	NA	0.000	NA	NA	NA	0.000	NA	NA	0.009
rs577273577	T	NA	0.000	NA	NA	NA	0.005	NA	NA	0.000
rs187186896	G	NA	0.000	NA	NA	NA	0.000	NA	NA	0.000
rs11551906	G	NA	0.000	NA	NA	NA	0.019	NA	NA	0.000
rs576653835	A	NA	0.000	NA	NA	NA	0.000	NA	NA	0.000
rs141694906	A	NA	0.000	NA	NA	NA	0.000	NA	NA	0.000
rs140396239	A	NA	0.000	NA	NA	NA	0.000	NA	NA	0.000
rs142446858	T	NA	NA	NA	NA	NA	NA	NA	NA	NA
rs561158080	T	NA	0.000	NA	NA	NA	0.000	NA	NA	0.000
rs2238543	T	NA	0.206	NA	NA	NA	0.481	NA	NA	0.009
rs554135380	T	NA	0.000	NA	NA	NA	0.000	NA	NA	0.000
rs543916508	A	NA	0.000	NA	NA	NA	0.000	NA	NA	0.000
rs551307834	T	NA	0.000	NA	NA	NA	0.000	NA	NA	0.000
rs146061693	A	NA	0.000	NA	NA	NA	0.000	NA	NA	0.009
rs559328258	T	NA	0.000	NA	NA	NA	0.000	NA	NA	0.000
rs545139311	A	NA	0.000	NA	NA	NA	0.000	NA	NA	0.000
rs572647656	T	NA	0.000	NA	NA	NA	0.000	NA	NA	0.000
rs372445442	A	NA	0.000	NA	NA	NA	0.000	NA	NA	0.000
rs190259313	A	NA	0.000	NA	NA	NA	0.000	NA	NA	0.000
rs561243171	T	NA	0.000	NA	NA	NA	0.000	NA	NA	0.097
rs138600800	C	NA	0.000	NA	NA	NA	0.005	NA	NA	0.000
rs28921977	G	NA	0.010	NA	NA	NA	0.065	NA	NA	0.005
rs571072379	T	NA	0.000	NA	NA	NA	0.000	NA	NA	0.000
rs375819042	T	NA	0.005	NA	NA	NA	0.000	NA	NA	0.000
rs41276870	T	NA	0.000	NA	NA	NA	0.009	NA	NA	0.000
rs567156454	C	NA	0.000	NA	NA	NA	0.000	NA	NA	0.000
rs539842435	T	NA	0.000	NA	NA	NA	0.000	NA	NA	0.000
rs529739382	G	NA	0.000	NA	NA	NA	0.000	NA	NA	0.005
rs2041192	A	0.125	0.245	0.191	0.132	0.229	0.523	0.258	0.219	0.056
rs565127256	A	NA	0.000	NA	NA	NA	0.000	NA	NA	0.000
rs561885264	T	NA	0.000	NA	NA	NA	0.005	NA	NA	0.000
rs111866858	A	NA	0.000	NA	NA	NA	0.000	NA	NA	0.116
rs141476449	T	NA	0.289	NA	NA	NA	0.131	NA	NA	0.171
rs144233497	A	NA	0.000	NA	NA	NA	0.000	NA	NA	0.000
rs183270957	T	NA	0.000	NA	NA	NA	0.005	NA	NA	0.000
rs554359587	T	NA	0.000	NA	NA	NA	0.000	NA	NA	0.005
rs55713331	T	NA	0.029	NA	NA	NA	0.000	NA	NA	0.000
rs367873477	C	NA	0.029	NA	NA	NA	0.000	NA	NA	0.000

SNP	Allele	Shipibo	STU	Tacna	Tallanes	Trujillo	TSI	Tumbes	Uros	YRI
rs576495399	T	NA	0.000	NA	NA	NA	0.000	NA	NA	0.000
rs564880752	A	NA	0.000	NA	NA	NA	0.000	NA	NA	0.000
rs549998110	T	NA	0.000	NA	NA	NA	0.000	NA	NA	0.000
rs370592787	T	NA	0.000	NA	NA	NA	0.000	NA	NA	0.005
rs12609912	T	NA	0.289	NA	NA	NA	0.131	NA	NA	0.167
rs138950962	A	NA	0.000	NA	NA	NA	0.000	NA	NA	0.000
rs28915406	G	NA	0.000	NA	NA	NA	0.000	NA	NA	0.019
rs560808777	A	NA	0.000	NA	NA	NA	0.000	NA	NA	0.000
rs553790377	A	NA	0.000	NA	NA	NA	0.000	NA	NA	0.000
rs576854854	T	NA	0.000	NA	NA	NA	0.000	NA	NA	0.000
rs558986305	A	NA	0.000	NA	NA	NA	0.000	NA	NA	0.000
rs185398568	A	NA	0.005	NA	NA	NA	0.000	NA	NA	0.000
rs555055894	A	NA	0.000	NA	NA	NA	0.000	NA	NA	0.000
rs181812598	T	NA	0.000	NA	NA	NA	0.000	NA	NA	0.009
rs534560674	G	NA	0.000	NA	NA	NA	0.000	NA	NA	0.000
rs2238541	T	NA	0.000	NA	NA	NA	0.000	NA	NA	0.000
rs148868153	T	NA	0.000	NA	NA	NA	0.000	NA	NA	0.000
rs10422922	G	NA	0.059	NA	NA	NA	0.070	NA	NA	0.199
rs529166895	A	NA	0.000	NA	NA	NA	0.000	NA	NA	0.000
rs570767821	A	NA	0.000	NA	NA	NA	0.000	NA	NA	0.000
rs572997523	A	NA	0.000	NA	NA	NA	0.000	NA	NA	0.000
rs527567567	T	NA	0.000	NA	NA	NA	0.000	NA	NA	0.000
rs187875345	A	NA	0.000	NA	NA	NA	0.005	NA	NA	0.000
rs10412522	A	0.000	0.059	0.023	0.000	0.000	0.070	0.045	0.000	0.167
rs199856669	G	NA	0.000	NA	NA	NA	0.000	NA	NA	0.000
rs113431687	A	NA	NA	NA	NA	NA	NA	NA	NA	NA
rs575490602	T	NA	0.000	NA	NA	NA	0.000	NA	NA	0.000
rs142683396	A	NA	0.000	NA	NA	NA	0.000	NA	NA	0.000
rs543921498	A	NA	0.000	NA	NA	NA	0.000	NA	NA	0.005
rs199564958	A	NA	0.000	NA	NA	NA	0.000	NA	NA	0.000
rs528839538	G	NA	0.000	NA	NA	NA	0.000	NA	NA	0.000
rs541648314	G	NA	0.000	NA	NA	NA	0.000	NA	NA	0.000
rs182434553	G	NA	0.000	NA	NA	NA	0.000	NA	NA	0.000
rs577301914	A	NA	0.000	NA	NA	NA	0.000	NA	NA	0.000
rs111517369	T	NA	0.000	NA	NA	NA	0.000	NA	NA	0.009
rs193000162	A	NA	0.000	NA	NA	NA	0.000	NA	NA	0.000
rs185020271	G	NA	0.000	NA	NA	NA	0.000	NA	NA	0.000
rs560734336	T	NA	0.000	NA	NA	NA	0.000	NA	NA	0.000
rs527804308	T	NA	0.000	NA	NA	NA	0.000	NA	NA	0.000
rs374000063	A	NA	0.000	NA	NA	NA	0.000	NA	NA	0.000
rs574157191	A	NA	0.000	NA	NA	NA	0.000	NA	NA	0.000
rs144058382	C	NA	0.000	NA	NA	NA	0.000	NA	NA	0.000
rs10422066	A	NA	0.456	NA	NA	NA	0.710	NA	NA	0.083

SNP	Allele	Shipibo	STU	Tacna	Tallanes	Trujillo	TSI	Tumbes	Uros	YRI
rs61729580	A	NA	0.000	NA	NA	NA	0.019	NA	NA	0.000
rs565778867	A	NA	0.000	NA	NA	NA	0.000	NA	NA	0.000
rs1803535	A	0.000	0.000	0.000	0.000	0.000	0.070	0.000	0.000	0.000
rs539526884	A	NA	0.000	NA	NA	NA	0.000	NA	NA	0.000
rs536177717	A	NA	0.010	NA	NA	NA	0.000	NA	NA	0.000
rs530649173	C	NA	0.000	NA	NA	NA	0.000	NA	NA	0.009
rs181996046	T	NA	0.000	NA	NA	NA	0.005	NA	NA	0.000
rs546352239	T	NA	0.000	NA	NA	NA	0.000	NA	NA	0.000
rs531528464	A	NA	0.005	NA	NA	NA	0.000	NA	NA	0.000
rs537327096	T	NA	0.000	NA	NA	NA	0.000	NA	NA	0.000
rs28921978	T	NA	0.074	NA	NA	NA	0.056	NA	NA	0.000
rs541295754	T	NA	0.000	NA	NA	NA	0.000	NA	NA	0.000
rs201497117	A	NA	0.000	NA	NA	NA	0.000	NA	NA	0.000
rs564731463	A	NA	0.000	NA	NA	NA	0.000	NA	NA	0.000
rs542324120	T	NA	0.000	NA	NA	NA	0.000	NA	NA	0.005
rs112540819	A	NA	0.000	NA	NA	NA	0.000	NA	NA	0.000
rs145586063	T	NA	0.000	NA	NA	NA	0.000	NA	NA	0.000
rs556111109	T	NA	0.000	NA	NA	NA	0.000	NA	NA	0.000
rs544240790	A	NA	0.000	NA	NA	NA	0.000	NA	NA	0.005
rs527846123	A	NA	0.000	NA	NA	NA	0.000	NA	NA	0.000
rs200823690	AC	NA	0.000	NA	NA	NA	0.000	NA	NA	0.000
rs547374902	A	NA	0.000	NA	NA	NA	0.000	NA	NA	0.000
rs534410335	T	NA	0.000	NA	NA	NA	0.000	NA	NA	0.000
rs11551900	G	NA	0.000	NA	NA	NA	0.000	NA	NA	0.051
rs188657384	T	NA	0.000	NA	NA	NA	0.000	NA	NA	0.009
rs7260603	A	NA	0.132	NA	NA	NA	0.126	NA	NA	0.204
rs537898048	A	NA	0.000	NA	NA	NA	0.000	NA	NA	0.000
rs575502711	A	NA	0.020	NA	NA	NA	0.000	NA	NA	0.000
rs532039913	A	NA	0.000	NA	NA	NA	0.000	NA	NA	0.000
rs558360796	C	NA	0.010	NA	NA	NA	0.000	NA	NA	0.000
rs547687363	G	NA	0.000	NA	NA	NA	0.000	NA	NA	0.000
rs144193084	T	NA	0.000	NA	NA	NA	0.000	NA	NA	0.000
rs28992473	G	NA	0.000	NA	NA	NA	0.000	NA	NA	0.032
rs564064006	G	NA	0.000	NA	NA	NA	0.000	NA	NA	0.000
rs73918141	T	NA	0.000	NA	NA	NA	0.000	NA	NA	0.014
rs537163143	A	NA	0.000	NA	NA	NA	0.000	NA	NA	0.009
rs559463709	A	NA	0.000	NA	NA	NA	0.000	NA	NA	0.000
rs28921986	T	NA	0.000	NA	NA	NA	0.000	NA	NA	0.056
rs7252138	T	0.125	0.167	0.205	0.200	0.188	0.477	0.258	0.219	0.009
rs537290646	G	NA	0.000	NA	NA	NA	0.000	NA	NA	0.000
rs12609013	T	NA	0.289	NA	NA	NA	0.131	NA	NA	0.167
rs144824657	T	NA	0.000	NA	NA	NA	0.009	NA	NA	0.000
rs2283569	C	NA	0.324	NA	NA	NA	0.136	NA	NA	0.167

SNP	Allele	Shipibo	STU	Tacna	Tallanes	Trujillo	TSI	Tumbes	Uros	YRI
rs532689650	A	NA	0.000	NA	NA	NA	0.000	NA	NA	0.000
rs549863661	T	NA	0.000	NA	NA	NA	0.000	NA	NA	0.000
rs199862501	T	NA	0.000	NA	NA	NA	0.000	NA	NA	0.000
rs575727796	G	NA	0.000	NA	NA	NA	0.000	NA	NA	0.005
rs539913003	A	NA	0.000	NA	NA	NA	0.000	NA	NA	0.000
rs532703227	A	NA	0.020	NA	NA	NA	0.005	NA	NA	0.000
rs1138151	A	NA	0.201	NA	NA	NA	0.481	NA	NA	0.009
rs537512067	T	NA	0.000	NA	NA	NA	0.000	NA	NA	0.000
rs569562559	A	NA	0.000	NA	NA	NA	0.000	NA	NA	0.000
rs28541371	T	NA	0.000	NA	NA	NA	0.000	NA	NA	0.009
rs547640504	T	NA	0.000	NA	NA	NA	0.000	NA	NA	0.000
rs368481272	T	NA	0.000	NA	NA	NA	0.000	NA	NA	0.000
rs138672257	A	NA	0.000	NA	NA	NA	0.000	NA	NA	0.005
rs150718850	A	NA	0.000	NA	NA	NA	0.005	NA	NA	0.000
rs28921976	C	NA	0.000	NA	NA	NA	0.009	NA	NA	0.000
rs28921989	T	NA	0.015	NA	NA	NA	0.005	NA	NA	0.000
rs541886354	C	NA	0.000	NA	NA	NA	0.000	NA	NA	0.000
rs373097790	C	NA	0.000	NA	NA	NA	0.000	NA	NA	0.000
rs546562394	A	NA	0.000	NA	NA	NA	0.000	NA	NA	0.000
rs555516404	A	NA	0.000	NA	NA	NA	0.000	NA	NA	0.000
rs572599699	T	NA	0.005	NA	NA	NA	0.000	NA	NA	0.000
rs560691399	T	NA	0.000	NA	NA	NA	0.000	NA	NA	0.000
rs192403165	T	NA	0.000	NA	NA	NA	0.000	NA	NA	0.000
rs529959881	C	NA	0.000	NA	NA	NA	0.000	NA	NA	0.000
rs563525909	T	NA	0.000	NA	NA	NA	0.000	NA	NA	0.005
rs552948152	A	NA	0.010	NA	NA	NA	0.000	NA	NA	0.000
rs5826703	GA	NA	0.226	NA	NA	NA	0.481	NA	NA	0.028
rs571034655	T	NA	0.005	NA	NA	NA	0.000	NA	NA	0.000
rs570927174	G	NA	0.000	NA	NA	NA	0.000	NA	NA	0.005
rs552366872	T	NA	0.000	NA	NA	NA	0.000	NA	NA	0.000
rs559051628	A	NA	0.000	NA	NA	NA	0.000	NA	NA	0.000
rs556312871	A	NA	0.000	NA	NA	NA	0.000	NA	NA	0.000
rs567001549	G	NA	0.000	NA	NA	NA	0.000	NA	NA	0.000
rs533301686	A	NA	0.000	NA	NA	NA	0.000	NA	NA	0.000
rs13676	A	0.688	0.069	0.273	0.429	0.208	0.051	0.349	0.344	0.005
rs563356581	A	NA	0.000	NA	NA	NA	0.000	NA	NA	0.000
rs111929936	A	0.000	0.000	0.000	0.000	0.000	0.000	0.000	0.000	0.023
rs151017812	T	NA	0.000	NA	NA	NA	0.000	NA	NA	0.000
rs28992485	T	NA	0.299	NA	NA	NA	0.131	NA	NA	0.167
rs578018107	A	NA	0.000	NA	NA	NA	0.000	NA	NA	0.000
rs543483161	T	NA	0.000	NA	NA	NA	0.000	NA	NA	0.000
rs368686315	C	NA	0.000	NA	NA	NA	0.000	NA	NA	0.000
rs535212456	T	NA	0.000	NA	NA	NA	0.000	NA	NA	0.000

SNP	Allele	Shipibo	STU	Tacna	Tallanes	Trujillo	TSI	Tumbes	Uros	YRI
rs202212562	T	NA	0.005	NA	NA	NA	0.000	NA	NA	0.000
rs2229662	T	NA	NA	NA	NA	NA	NA	NA	NA	NA
rs150860707	T	NA	0.000	NA	NA	NA	0.005	NA	NA	0.000
rs543519383	A	NA	0.000	NA	NA	NA	0.000	NA	NA	0.000
rs199725376	T	NA	0.010	NA	NA	NA	0.005	NA	NA	0.000
rs10402344	T	NA	0.755	NA	NA	NA	0.827	NA	NA	0.125
rs200189683	A	NA	0.000	NA	NA	NA	0.000	NA	NA	0.000
rs113445094	T	NA	NA	NA	NA	NA	NA	NA	NA	NA
rs527594490	G	NA	0.000	NA	NA	NA	0.000	NA	NA	0.000
rs545107557	T	NA	0.000	NA	NA	NA	0.000	NA	NA	0.000
rs7259828	T	NA	0.128	NA	NA	NA	0.122	NA	NA	0.204
rs112251245	T	NA	0.000	NA	NA	NA	0.000	NA	NA	0.046
rs557528051	G	NA	0.000	NA	NA	NA	0.000	NA	NA	0.000
rs540128503	T	NA	0.000	NA	NA	NA	0.000	NA	NA	0.000
rs529018328	T	NA	0.000	NA	NA	NA	0.000	NA	NA	0.000
rs183742660	A	NA	0.000	NA	NA	NA	0.000	NA	NA	0.000
rs572465381	GGGC	NA	0.000	NA	NA	NA	0.000	NA	NA	0.014
rs549282195	GAGAT	NA	0.000	NA	NA	NA	0.000	NA	NA	0.000
rs545279552	C	NA	0.000	NA	NA	NA	0.000	NA	NA	0.000
rs201531347	A	NA	0.000	NA	NA	NA	0.000	NA	NA	0.000
rs566474268	A	NA	0.000	NA	NA	NA	0.000	NA	NA	0.000
rs560075963	C	NA	0.000	NA	NA	NA	0.000	NA	NA	0.000
rs555375779	T	NA	0.015	NA	NA	NA	0.000	NA	NA	0.000
rs551356579	T	NA	0.000	NA	NA	NA	0.000	NA	NA	0.000
rs541386550	T	NA	0.000	NA	NA	NA	0.000	NA	NA	0.000
rs184596705	A	NA	0.000	NA	NA	NA	0.000	NA	NA	0.005
rs139397975	A	NA	0.000	NA	NA	NA	0.000	NA	NA	0.000
rs567401469	T	NA	0.000	NA	NA	NA	0.000	NA	NA	0.000
rs11473	T	NA	0.000	NA	NA	NA	0.000	NA	NA	0.088
rs192885278	A	NA	0.000	NA	NA	NA	0.005	NA	NA	0.000
rs572733540	G	NA	0.353	NA	NA	NA	0.136	NA	NA	0.153
rs145675941	A	NA	0.000	NA	NA	NA	0.000	NA	NA	0.005
rs547384835	T	NA	0.000	NA	NA	NA	0.000	NA	NA	0.000
rs140385832	C	NA	0.000	NA	NA	NA	0.000	NA	NA	0.000
rs13235	T	NA	0.000	NA	NA	NA	0.000	NA	NA	0.005
rs573279565	A	NA	0.000	NA	NA	NA	0.000	NA	NA	0.005
rs117933091	A	NA	0.005	NA	NA	NA	0.000	NA	NA	0.000
rs2238544	A	NA	0.245	NA	NA	NA	0.533	NA	NA	0.056
rs372040583	A	NA	0.000	NA	NA	NA	0.000	NA	NA	0.000
rs530681399	T	NA	0.000	NA	NA	NA	0.000	NA	NA	0.000
rs191110653	A	NA	0.020	NA	NA	NA	0.005	NA	NA	0.014
rs3764938	T	NA	0.480	NA	NA	NA	0.257	NA	NA	0.500
rs139776734	A	NA	0.000	NA	NA	NA	0.000	NA	NA	0.000

SNP	Allele	Shipibo	STU	Tacna	Tallanes	Trujillo	TSI	Tumbes	Uros	YRI
rs575050179	A	NA	0.005	NA	NA	NA	0.000	NA	NA	0.000
rs572043068	A	NA	0.000	NA	NA	NA	0.000	NA	NA	0.000
rs2238545	C	NA	0.289	NA	NA	NA	0.131	NA	NA	0.167
rs533373164	T	NA	0.000	NA	NA	NA	0.009	NA	NA	0.000
rs201635915	A	NA	0.000	NA	NA	NA	0.000	NA	NA	0.000
rs541344713	G	NA	0.000	NA	NA	NA	0.000	NA	NA	0.000
rs567649209	T	NA	0.000	NA	NA	NA	0.000	NA	NA	0.000
rs529458335	T	NA	0.000	NA	NA	NA	0.000	NA	NA	0.000
rs186158048	T	NA	0.000	NA	NA	NA	0.000	NA	NA	0.000
rs560636050	A	NA	0.000	NA	NA	NA	0.000	NA	NA	0.000
rs111841168	C	NA	NA	NA	NA	NA	NA	NA	NA	NA
rs137922704	T	NA	0.000	NA	NA	NA	0.000	NA	NA	0.005
rs28992470	A	NA	0.005	NA	NA	NA	0.014	NA	NA	0.000
rs190631157	T	NA	0.000	NA	NA	NA	0.000	NA	NA	0.000
rs28921991	A	NA	0.000	NA	NA	NA	0.000	NA	NA	0.023
rs577780415	G	NA	0.000	NA	NA	NA	0.000	NA	NA	0.000
rs1063378	C	NA	NA	NA	NA	NA	NA	NA	NA	NA
rs140499328	T	NA	0.284	NA	NA	NA	0.131	NA	NA	0.167
rs28921972	A	NA	0.000	NA	NA	NA	0.000	NA	NA	0.005
rs191842753	T	NA	0.000	NA	NA	NA	0.009	NA	NA	0.000
rs150369286	T	NA	0.000	NA	NA	NA	0.000	NA	NA	0.005
rs28992484	T	NA	0.319	NA	NA	NA	0.131	NA	NA	0.167
rs140722203	A	NA	0.000	NA	NA	NA	0.000	NA	NA	0.000
rs546300301	G	NA	0.000	NA	NA	NA	0.000	NA	NA	0.000
rs531900250	A	NA	0.000	NA	NA	NA	0.000	NA	NA	0.000
rs577921461	T	NA	0.000	NA	NA	NA	0.000	NA	NA	0.000
rs201403607	A	NA	0.000	NA	NA	NA	0.000	NA	NA	0.000
rs7255181	G	NA	0.000	NA	NA	NA	0.000	NA	NA	0.032
rs141583892	T	NA	0.000	NA	NA	NA	0.000	NA	NA	0.014
rs113790346	G	NA	NA	NA	NA	NA	NA	NA	NA	NA
rs551388840	T	NA	0.000	NA	NA	NA	0.000	NA	NA	0.000
rs542552024	A	NA	0.000	NA	NA	NA	0.000	NA	NA	0.000
rs552594392	A	NA	0.000	NA	NA	NA	0.000	NA	NA	0.000
rs182826947	A	NA	0.000	NA	NA	NA	0.000	NA	NA	0.005
rs188799878	A	NA	0.000	NA	NA	NA	0.000	NA	NA	0.000
rs11879178	A	NA	0.324	NA	NA	NA	0.215	NA	NA	0.301
rs28993669	C	NA	NA	NA	NA	NA	NA	NA	NA	NA
rs191246732	A	NA	0.000	NA	NA	NA	0.000	NA	NA	0.000
rs551261955	T	NA	0.000	NA	NA	NA	0.000	NA	NA	0.000
rs146186991	A	NA	0.000	NA	NA	NA	0.009	NA	NA	0.000
rs544039773	T	NA	0.000	NA	NA	NA	0.000	NA	NA	0.000
rs554365832	A	NA	0.000	NA	NA	NA	0.000	NA	NA	0.000
rs138039913	T	NA	0.000	NA	NA	NA	0.000	NA	NA	0.000

SNP	Allele	Shipibo	STU	Tacna	Tallanes	Trujillo	TSI	Tumbes	Uros	YRI
rs541089761	A	NA	0.000	NA	NA	NA	0.000	NA	NA	0.000
rs28921985	A	NA	0.000	NA	NA	NA	0.000	NA	NA	0.023
rs529207268	G	NA	0.000	NA	NA	NA	0.000	NA	NA	0.000
rs190217060	A	NA	0.000	NA	NA	NA	0.000	NA	NA	0.000
rs552965493	T	NA	0.000	NA	NA	NA	0.000	NA	NA	0.000
rs545068935	G	NA	0.000	NA	NA	NA	0.000	NA	NA	0.005
rs540123195	A	NA	0.000	NA	NA	NA	0.000	NA	NA	0.000
rs12611131	T	NA	0.324	NA	NA	NA	0.136	NA	NA	0.167
rs200467897	T	NA	0.000	NA	NA	NA	0.000	NA	NA	0.000
rs28992469	T	NA	0.000	NA	NA	NA	0.000	NA	NA	0.005
rs2072307	C	NA	0.294	NA	NA	NA	0.131	NA	NA	0.181
rs535566277	A	NA	0.000	NA	NA	NA	0.000	NA	NA	0.000
rs145461969	T	NA	0.000	NA	NA	NA	0.005	NA	NA	0.000
rs568389785	T	NA	0.000	NA	NA	NA	0.000	NA	NA	0.000
rs56043551	T	NA	NA	NA	NA	NA	NA	NA	NA	NA
rs191693960	T	NA	0.000	NA	NA	NA	0.000	NA	NA	0.005
rs28921968	G	NA	NA	NA	NA	NA	NA	NA	NA	NA
rs549983564	T	NA	0.000	NA	NA	NA	0.000	NA	NA	0.000
rs200630157	T	NA	0.000	NA	NA	NA	0.000	NA	NA	0.005
rs550013368	A	NA	0.000	NA	NA	NA	0.000	NA	NA	0.014
rs191389346	T	NA	0.010	NA	NA	NA	0.000	NA	NA	0.000
rs28921983	G	NA	NA	NA	NA	NA	NA	NA	NA	NA
rs564546624	T	NA	0.000	NA	NA	NA	0.000	NA	NA	0.000
rs150791055	T	NA	0.000	NA	NA	NA	0.000	NA	NA	0.005
rs202135216	T	NA	0.000	NA	NA	NA	0.000	NA	NA	0.000
rs575212330	T	NA	0.005	NA	NA	NA	0.000	NA	NA	0.000
rs559570766	T	NA	0.000	NA	NA	NA	0.000	NA	NA	0.000
rs151237303	T	NA	0.284	NA	NA	NA	0.131	NA	NA	0.167
rs369186206	A	NA	0.000	NA	NA	NA	0.000	NA	NA	0.000
rs28921975	A	NA	0.000	NA	NA	NA	0.000	NA	NA	0.019
rs538891893	G	NA	0.000	NA	NA	NA	0.005	NA	NA	0.000
rs566302789	T	NA	0.000	NA	NA	NA	0.000	NA	NA	0.000
rs189865097	A	NA	0.000	NA	NA	NA	0.000	NA	NA	0.000
rs2283570	T	NA	0.309	NA	NA	NA	0.131	NA	NA	0.162
rs181004617	A	NA	0.000	NA	NA	NA	0.005	NA	NA	0.000
rs2283574	A	NA	0.289	NA	NA	NA	0.131	NA	NA	0.167
rs553131488	A	NA	0.000	NA	NA	NA	0.000	NA	NA	0.000
rs200432051	T	NA	0.000	NA	NA	NA	0.000	NA	NA	0.000
rs569279461	T	NA	0.000	NA	NA	NA	0.000	NA	NA	0.000
rs112785861	A	NA	0.000	NA	NA	NA	0.000	NA	NA	0.111
rs529813357	T	NA	0.000	NA	NA	NA	0.000	NA	NA	0.000
rs563512179	A	NA	0.000	NA	NA	NA	0.000	NA	NA	0.000
rs201881530	G	NA	0.000	NA	NA	NA	0.000	NA	NA	0.000

SNP	Allele	Shipibo	STU	Tacna	Tallanes	Trujillo	TSI	Tumbes	Uros	YRI
rs2283571	C	NA	0.534	NA	NA	NA	0.654	NA	NA	0.232
rs116073835	A	NA	0.000	NA	NA	NA	0.000	NA	NA	0.042
rs368973405	T	NA	0.000	NA	NA	NA	0.000	NA	NA	0.009
rs553337242	T	NA	0.000	NA	NA	NA	0.000	NA	NA	0.000
rs577310345	C	NA	0.000	NA	NA	NA	0.000	NA	NA	0.000
rs538315599	A	NA	0.000	NA	NA	NA	0.000	NA	NA	0.000
rs556900373	A	NA	0.000	NA	NA	NA	0.000	NA	NA	0.000
rs534224219	T	NA	0.000	NA	NA	NA	0.000	NA	NA	0.000
rs28915408	A	NA	0.000	NA	NA	NA	0.009	NA	NA	0.000
rs538902991	T	NA	0.000	NA	NA	NA	0.000	NA	NA	0.000
rs200237260	T	NA	0.000	NA	NA	NA	0.000	NA	NA	0.000
rs28992480	A	NA	0.000	NA	NA	NA	0.000	NA	NA	0.032
rs12979852	T	NA	0.142	NA	NA	NA	0.042	NA	NA	0.245
rs200853058	T	NA	0.000	NA	NA	NA	0.000	NA	NA	0.000
rs560866175	A	NA	0.000	NA	NA	NA	0.000	NA	NA	0.000
rs186833702	A	NA	0.000	NA	NA	NA	0.000	NA	NA	0.000
rs113907190	G	NA	NA	NA	NA	NA	NA	NA	NA	NA
rs28921990	C	NA	0.289	NA	NA	NA	0.131	NA	NA	0.167
rs577273590	G	NA	0.000	NA	NA	NA	0.000	NA	NA	0.000
rs536730099	A	NA	0.000	NA	NA	NA	0.000	NA	NA	0.000
rs143543245	T	NA	0.000	NA	NA	NA	0.000	NA	NA	0.000
rs202113312	C	NA	NA	NA	NA	NA	NA	NA	NA	NA
rs565922179	T	NA	0.000	NA	NA	NA	0.000	NA	NA	0.000
rs113033881	C	NA	0.289	NA	NA	NA	0.131	NA	NA	0.264
rs565509602	T	NA	0.000	NA	NA	NA	0.000	NA	NA	0.000
rs552797010	A	NA	0.000	NA	NA	NA	0.005	NA	NA	0.000
rs534935683	T	NA	0.000	NA	NA	NA	0.000	NA	NA	0.000
rs367579969	T	NA	0.000	NA	NA	NA	0.000	NA	NA	0.000
rs568509871	C	NA	0.000	NA	NA	NA	0.000	NA	NA	0.000
rs565826251	A	NA	0.000	NA	NA	NA	0.000	NA	NA	0.000
rs542552123	A	NA	0.000	NA	NA	NA	0.000	NA	NA	0.000
rs28992471	A	NA	0.289	NA	NA	NA	0.131	NA	NA	0.167
rs563724259	T	NA	0.000	NA	NA	NA	0.000	NA	NA	0.000
rs28921988	T	NA	0.000	NA	NA	NA	0.000	NA	NA	0.037
rs560442683	T	NA	0.000	NA	NA	NA	0.000	NA	NA	0.000
rs537692667	A	NA	0.000	NA	NA	NA	0.000	NA	NA	0.000
rs28921971	T	NA	0.069	NA	NA	NA	0.051	NA	NA	0.000
rs572811560	A	NA	0.000	NA	NA	NA	0.000	NA	NA	0.000
rs28992483	T	NA	0.289	NA	NA	NA	0.131	NA	NA	0.167
rs547376405	G	NA	0.000	NA	NA	NA	0.000	NA	NA	0.000
rs138748626	T	NA	0.000	NA	NA	NA	0.000	NA	NA	0.000
rs528453463	A	NA	0.000	NA	NA	NA	0.000	NA	NA	0.000
rs552838011	G	NA	0.000	NA	NA	NA	0.000	NA	NA	0.000

SNP	Allele	Shipibo	STU	Tacna	Tallanes	Trujillo	TSI	Tumbes	Uros	YRI
rs544905487	T	NA	0.000	NA	NA	NA	0.000	NA	NA	0.000
rs139995866	A	NA	0.000	NA	NA	NA	0.000	NA	NA	0.000
rs556660637	A	NA	0.000	NA	NA	NA	0.005	NA	NA	0.000
rs559190146	A	NA	0.000	NA	NA	NA	0.000	NA	NA	0.000
rs544661215	T	NA	0.000	NA	NA	NA	0.000	NA	NA	0.000
rs2238542	T	NA	0.289	NA	NA	NA	0.131	NA	NA	0.167
rs571648795	T	NA	0.000	NA	NA	NA	0.000	NA	NA	0.000
rs567013917	A	NA	0.000	NA	NA	NA	0.000	NA	NA	0.000
rs187005996	T	NA	0.000	NA	NA	NA	0.000	NA	NA	0.005
rs28473389	T	NA	0.075	NA	NA	NA	0.056	NA	NA	0.000
rs71334826	C	NA	0.000	NA	NA	NA	0.000	NA	NA	0.014
rs543090783	A	NA	0.000	NA	NA	NA	0.000	NA	NA	0.000
rs540356623	A	NA	0.000	NA	NA	NA	0.000	NA	NA	0.000
rs564651283	T	NA	0.000	NA	NA	NA	0.000	NA	NA	0.000
rs12609610	G	0.000	0.000	0.000	0.015	0.000	0.000	0.015	0.000	0.000
rs572636722	T	NA	0.000	NA	NA	NA	0.000	NA	NA	0.000
rs10416339	A	NA	0.534	NA	NA	NA	0.664	NA	NA	0.232
rs56067110	A	NA	0.000	NA	NA	NA	0.000	NA	NA	0.023
rs552690398	A	NA	0.000	NA	NA	NA	0.000	NA	NA	0.000
rs72972182	C	NA	0.074	NA	NA	NA	0.056	NA	NA	0.000
rs143454800	G	NA	0.000	NA	NA	NA	0.000	NA	NA	0.000
rs539563939	A	NA	0.000	NA	NA	NA	0.000	NA	NA	0.000
rs368584754	A	NA	0.000	NA	NA	NA	0.000	NA	NA	0.005
rs529198553	A	NA	0.000	NA	NA	NA	0.000	NA	NA	0.000
rs557853268	A	NA	0.000	NA	NA	NA	0.000	NA	NA	0.000
rs533240475	A	NA	0.000	NA	NA	NA	0.000	NA	NA	0.000
rs202082105	A	NA	0.000	NA	NA	NA	0.000	NA	NA	0.005

bold: functionally relevant SNPs found in our databases
