# Supplementary material for: Human-SARS-CoV-2 interactome and human genetic diversity: TMPRSS2-rs2070788, associated with severe influenza, and its population genetics caveats in Native Americans
Source: Genet Mol Biol. 2021 Aug 25;44(1 Suppl 1):e20200484. doi: 10.1590/1678-4685-GMB-2020-0484 (PMC8387978; doi:10.1590/1678-4685-GMB-2020-0484)
Supplement: Table S3-B - [file 1415-4757-GMB-44-1-s1-e20200484-s7.pdf]

Genetics and Molecular Biology
https://doi.org/10.1590/1678-4685-GMB-2020-0484

Supplementary Material to "Human-SARS-CoV-2 interactome and human genetic diversity: *TMPRSS2*-rs2070788, associated with severe influenza, and its population genetics caveats in Native Americans"

Table S3-B – BSG regression.

			rs8259	rs6757	rs8637	rs12608994	rs1803202	rs11879069	rs4919862	rs6758	rs28992474	rs2072310	rs3764937	rs4682	rs2072309	rs2074962
AFR																
	Intercept		-0.25112	-2.03318605	-0.11681515	-1.86519313	-1.86945698	-0.98852653	-0.99769382	-1.93158039	-1.79807617	-1.01266943	-1.8049257	-0.9852905	-0.98520353	-0.01217407
	P-Value		0.000571	4.04E-98	0.32872643	7.47E-154	4.72E-145	5.73E-19	6.04E-49	1.15E-101	4.51E-127	1.38E-12	1.13E-152	1.16E-11	2.30E-19	0.92802478
	Beta		0.4074112	0.79771	-2.44588	0.69111	0.60217	0.31647	-1.86104	0.67407	0.54076	0.34366	1.35674	0.32542	0.17298	-1.8393
	P-Value		7.32E-02	7.00E-05	< 0.001	< 0.001	6.00E-05	0.2272	< 0.001	8.00E-05	0.00023	0.24936	< 0.001	0.28458	0.50949	< 0.001
	R^2		0.5156219	0.42783	0.88812	0.58732	0.50186	0.07823	0.93247	0.5273	0.5107	0.09834	0.88543	0.08632	0.02538	0.77281
	AICc		-10.29285	-56.73331	-20.07187	-57.06921	-56.47303	-31.42851	-39.54234	-40.62605	-42.99926	-17.01147	-43.87621	-16.35926	-32.14916	-15.17225
EUR																
	Intercept		0.1296	-1.6429864	-1.99303475	-1.3720319	-1.43352424	-0.61744519	-2.15806062	-1.42567486	-1.42150275	-0.43912547	-0.8754012	-0.41912268	-0.69236222	-1.59034609
	P-Value		0.4195	4.04E-28	1.26E-15	2.66E-31	1.39E-35	3.49E-07	3.72E-17	3.64E-29	1.97E-34	0.00013956	1.09E-06	0.00051942	2.60E-08	8.64E-21
	Beta		-0.4167799	-0.37389	2.26154	-0.53637	-0.48073	-0.60638	1.32748	-0.5944	-0.42183	-0.91636	-0.97946	-0.90989	-0.51384	1.96975
	P-Value		8.53E-02	0.12462	< 0.001	0.0033	0.00672	0.00224	8.00E-05	0.00287	0.01781	< 0.001	0.00071	< 0.001	0.0111	< 0.001
	R^2		0.4953616	0.13135	0.77384	0.37304	0.33553	0.37809	0.55708	0.42692	0.32326	0.69689	0.51387	0.67585	0.29467	0.8456
	AICc		-10.16994	-48.76643	-19.07696	-50.46218	-51.95304	-37.62904	-23.21953	-37.8514	-38.91449	-29.46701	-24.0379	-28.17619	-37.26594	-24.08629
NAT																
	Intercept		0.7867	-1.75228379	-0.67257989	-1.60893605	-1.64695782	-0.91785887	-1.40206575	-1.68090385	-1.59630108	-0.97171535	-1.28925444	-0.96597386	-0.95211001	-0.4279221
	P-Value		5.48E-07	1.04E-55	0.01694098	4.72E-65	3.58E-75	1.18E-16	7.64E-11	2.28E-48	8.09E-65	7.20E-12	8.56E-14	9.92E-12	5.42E-19	0.08072911
	Beta		-13.2271543	-0.41673	0.50996	-0.74768	-0.66644	0.01552	0.63963	-0.70573	-0.62558	0.54014	-1.10589	0.7164	0.0342	-0.0272
	P-Value		3.25E-09	0.20292	0.7481	0.2527	0.2794	0.95811	0.57935	0.32946	0.28632	0.4945	0.31697	0.36111	0.90426	0.98471
	R^2		0.9214034	0.09937	0.00873	0.09379	0.08328	0.00017	0.02384	0.08502	0.09916	0.03219	0.09953	0.05516	0.00091	3.00E-05
	AICc		-15.73816	-48.17859	1.99085	-45.1156	-47.27126	-30.07765	-12.46395	-32.30425	-35.53481	-16.21489	-17.2544	-16.02642	-31.74283	0.7035
EAS																

			rs8259	rs6757	rs8637	rs12608994	rs1803202	rs11879069	rs4919862	rs6758	rs28992474	rs2072310	rs3764937	rs4682	rs2072309	rs2074962
	**Intercept**		0.6422	-1.83718787	-0.62852742	-1.66499065	-1.69976756	-1.00328191	-1.39880431	-1.75019003	-1.64676527	-1.05202412	-1.35664478	-1.0259914	-1.03591219	-0.4056112
	**P-Value**		0.1142	4.05E-65	0.01583997	5.54E-78	1.89E-90	1.23E-35	1.79E-12	4.90E-55	8.30E-72	2.70E-26	5.89E-16	2.26E-23	2.61E-43	0.07178472
	**Beta**		-75.8291343	0.14436	0.00794	-0.03952	-0.00462	1.00467	0.4795	0.10711	-0.01216	1.05554	-0.21685	1.03313	1.01988	-0.24066
	**P-Value**		5.38E-02	0.72791	0.99281	0.90842	0.98856	0.00071	0.4282	0.77185	0.96923	0.00055	0.71476	0.0012	0.00021	0.7598
	**R^2**		0.5523719	0.00666	1.00E-05	0.00094	1.00E-05	0.35514	0.03825	0.00685	0.00013	0.45017	0.01332	0.42263	0.39499	0.00818
	**AICc**		-10.52944	-46.60591	2.09724	-43.76854	-46.05849	-38.17358	-12.71313	-31.39606	-34.36907	-23.50853	-16.33798	-22.33044	-41.02601	0.60713
AFR+NAT																
	**Intercept**		-	-1.9940442	0.02317563	-1.83744586	-1.8438326	-1.02423823	-0.93414928	-1.90401253	-1.76919465	-1.11438415	-1.77809998	-1.10905858	-1.01222132	0.15669533
	**P-Value**		-	5.51E-66	0.85122583	2.18E-103	1.90E-97	5.95E-14	5.52E-38	7.85E-71	4.83E-89	2.38E-11	4.93E-106	3.00E-11	5.42E-14	0.25457124
	**Beta**	AFR	-	0.75288	-2.59793	0.66425	0.57739	0.35793	-1.91912	0.6475	0.51306	0.43673	1.33072	0.43812	0.20447	-2.01712
		NAT	-	-0.15685	-1.23812	-0.2528	-0.23341	0.13643	-0.5862	-0.24388	-0.25632	0.85771	-0.23421	1.03505	0.1033	-1.47782
	**P-Value**		-	4.00E-04	< 0.001	1.00E-05	0.00021	0.19602	< 0.001	0.00029	0.00071	0.14586	< 0.001	0.14515	0.46068	< 0.001
			-	0.5642	0.0485	0.57908	0.61943	0.64923	0.1184	0.64863	0.56346	0.26279	0.56351	0.17344	0.72786	0.03365
	**R^2**		-	0.43953	0.91631	0.59796	0.51207	0.09036	0.94485	0.53702	0.52659	0.17365	0.88939	0.19289	0.03291	0.83695
	**AICc**		-	-53.58695	-18.97193	-53.56873	-52.90894	-28.14768	-37.24374	-36.12649	-38.6286	-13.3587	-39.50733	-13.17548	-28.78355	-14.53983
			-													
AFR+EUR																
	**Intercept**		-	-2.00379389	-0.9873799	-1.78400256	-1.77882978	-0.59986328	-1.16517388	-1.74781318	-1.73413278	-0.01414263	-1.71208174	0.02643636	-0.62118702	-0.99620388
	**P-Value**		-	1.02E-28	3.64E-12	3.74E-22	5.38E-21	0.00023769	7.32E-13	1.01E-17	9.35E-22	0.90751116	1.50E-25	0.83636635	0.00015831	3.06E-09
	**Beta**	AFR	-	0.77049	-1.73312	0.61164	0.5135	-0.03956	-1.70154	0.49087	0.47721	-0.66448	1.26465	-0.69633	-0.16062	-0.96013
		EUR	-	-0.04569	1.17087	-0.10601	-0.11843	-0.62348	0.22476	-0.24943	-0.08624	-1.39751	-0.12535	-1.41412	-0.58369	1.3226
	**P-Value**		-	0.00159	< 0.001	0.00506	0.02275	0.8733	< 0.001	0.04561	0.02903	2.00E-05	< 0.001	2.00E-05	0.52467	5.00E-05
			-	0.84833	< 0.001	0.6365	0.60725	0.00552	0.25628	0.32797	0.70015	< 0.001	0.53773	< 0.001	0.00978	< 0.001
	**R^2**		-	0.42971	0.97545	0.59472	0.51198	0.37893	0.93914	0.56453	0.51769	0.85915	0.88961	0.85067	0.31064	0.94658
	**AICc**		-	-53.28285	-35.09863	-53.46584	-52.90821	-34.16744	-36.06201	-36.78119	-38.42883	-35.68093	-39.52369	-34.34545	-34.18284	-29.27239
			-													
AFR+EAS																
	**Intercept**		-	-2.07326279	0.01059357	-1.88848245	-1.89380403	-1.12226322	-0.9878676	-1.98226826	-1.82258344	-1.21718689	-1.83475558	-1.18313206	-1.11549734	0.12567015
	**P-Value**		-	3.12E-94	0.91533425	8.79E-139	5.53E-131	7.24E-38	2.73E-40	1.53E-98	7.68E-112	1.63E-36	1.02E-139	2.34E-29	6.81E-38	0.27838248
	**Beta**	AFR	-	0.84199	-2.68344	0.71815	0.63048	0.46386	-1.87398	0.72699	0.56686	0.55073	1.38856	0.52626	0.31485	-2.04841

			rs8259	rs6757	rs8637	rs12608994	rs1803202	rs11879069	rs4919862	rs6758	rs28992474	rs2072310	rs3764937	rs4682	rs2072309	rs2074962
		EUR	-	0.37167	-0.88044	0.18698	0.19453	1.1346	-0.06682	0.33701	0.16776	1.23665	0.20564	1.2056	1.10668	-0.95735
	**P-Value**		-	2.00E-05	< 0.001	< 0.001	3.00E-05	0.01615	< 0.001	1.00E-05	0.00013	0.00275	< 0.001	0.00898	0.10568	< 0.001
			-	0.22965	0.00433	0.40582	0.40025	2.00E-05	0.75454	0.17288	0.45375	< 0.001	0.30659	1.00E-05	2.00E-05	0.00745
	**R^2**		-	0.47126	0.93554	0.60846	0.52732	0.51431	0.93309	0.59227	0.53489	0.67967	0.89575	0.62862	0.47487	0.8619
	**AICc**		-	-54.48559	-22.06645	-53.89376	-53.31166	-39.501	-34.92555	-37.49883	-38.81193	-25.22328	-40.11035	-22.81087	-39.91451	-16.52775
			-													
**NAT+EUR**																
	**Intercept**		-	-1.3635362	-2.12664335	-1.31299709	-1.38036038	-0.46006483	-2.28434277	-1.36695441	-1.36827596	-0.47849388	-0.78238398	-0.47376162	-0.56909085	-1.6088833
	**P-Value**		-	3.63E-21	6.22E-15	2.13E-31	4.81E-35	0.00109598	2.29E-16	1.02E-29	1.09E-34	4.35E-05	2.45E-06	6.44E-05	0.00015203	5.61E-18
	**Beta**	NAT	-	-0.78007	0.85485	-0.76691	-0.68357	-0.41265	0.88497	-0.74748	-0.64985	0.47387	-1.2111	0.65315	-0.32232	0.14238
		EUR	-	-0.66665	2.34075	-0.53243	-0.47777	-0.76825	1.39691	-0.58924	-0.4206	-0.92489	-0.96982	-0.92229	-0.6398	1.97807
	**P-Value**		-	0.00497	0.2498	0.12101	0.15741	0.08593	0.25715	0.15138	0.1611	0.27778	0.10852	0.13595	0.20563	0.80445
			-	0.00165	< 0.001	0.00133	0.0035	0.00014	3.00E-05	0.00107	0.00938	< 0.001	0.00015	< 0.001	0.00269	< 0.001
	**R^2**		-	0.42114	0.78944	0.48088	0.43136	0.46717	0.58991	0.52623	0.43577	0.71919	0.63037	0.718	0.35352	0.84633
	**AICc**		-	-52.47818	-15.54511	-49.2	-50.26024	-36.96917	-19.63299	-35.30951	-36.22933	-25.81558	-22.12792	-25.38073	-35.36464	-19.4325
			-													
**NAT+EAS**																
	**Intercept**		-	-1.75846932	-0.67711099	-1.6003313	-1.64246935	-1.03028105	-1.46964876	-1.68883573	-1.5898958	-1.13195497	-1.25405307	-1.12469208	-1.06738842	-0.39856689
	**P-Value**		-	6.01E-50	0.02484631	7.00E-58	6.78E-67	9.04E-28	1.16E-10	1.61E-42	2.52E-56	2.05E-25	5.47E-12	1.24E-24	3.85E-34	0.12731794
	**Beta**	NAT	-	-0.4096	0.51687	-0.768	-0.6767	0.12832	0.72481	-0.69048	-0.63806	0.79579	-1.18702	0.97243	0.1492	-0.0755
		EAS	-	0.06311	0.03786	-0.08311	-0.04323	1.03223	0.52616	0.06276	-0.05196	1.11685	-0.2911	1.10892	1.05202	-0.24512
	**P-Value**		-	0.21491	0.74636	0.24278	0.2751	0.58687	0.52256	0.34227	0.27977	0.1498	0.28342	0.07916	0.49492	0.95771
			-	0.87499	0.9659	0.79989	0.88889	0.00054	0.384	0.86017	0.86275	1.00E-04	0.60695	0.00014	0.00013	0.75681
	**R^2**		-	0.10061	0.00891	0.09772	0.08443	0.36568	0.06963	0.08753	0.1014	0.516	0.12264	0.51872	0.41055	0.00843
	**AICc**		-	-44.71573	6.70329	-41.36405	-43.4731	-34.97906	-8.41082	-27.62071	-30.8512	-20.51877	-12.82405	-20.08272	-37.99785	5.31847
			-													
**EUR+EAS**																
	**Intercept**		-	-1.63263422	-2.41188807	-1.25068207	-1.33514594	-0.75255469	-2.58300628	-1.33545186	-1.32911185	-0.6102673	-0.58357126	-0.58359775	-0.83991705	-1.87862862
	**P-Value**		-	3.31E-23	1.70E-37	9.06E-25	2.46E-27	2.13E-11	2.08E-34	1.34E-21	3.55E-26	3.61E-09	5.24E-05	3.67E-07	7.08E-14	1.44E-49
	**Beta**	EUR	-	-0.38547	2.70488	-0.68537	-0.60019	-0.46918	1.74864	-0.7004	-0.52871	-0.736	-1.35967	-0.73633	-0.36492	2.2807
		EAS	-	-0.06026	1.61857	-0.47387	-0.38415	0.76697	1.57685	-0.33011	-0.34262	0.62045	-1.08652	0.59793	0.83374	1.10294
	**P-Value**		-	0.131	< 0.001	0.00013	0.00088	0.00681	< 0.001	0.00066	0.00345	< 0.001	< 0.001	1.00E-05	0.03376	< 0.001
			-	0.88289	< 0.001	0.0808	0.15501	0.00346	< 0.001	0.26075	0.19739	0.0016	0.00149	0.00631	0.00128	< 0.001
	**R^2**		-	0.13247	0.946	0.47511	0.41122	0.55847	0.87729	0.47304	0.40113	0.81916	0.7287	0.78646	0.52775	0.95524
	**AICc**		-	-45.30152	-28.02172	-49.68189	-50.18378	-40.89399	-30.48391	-34.41173	-35.85619	-31.95563	-27.74421	-29.23316	-41.61982	-32.03777
			-													

			rs8259	rs6757	rs8637	rs12608994	rs1803202	rs11879069	rs4919862	rs6758	rs28992474	rs2072310	rs3764937	rs4682	rs2072309	rs2074962
AFR+NAT+EUR																
	**Intercept**		-	-1.70180688	-0.83006706	-1.6965144	-1.69487398	0.0164236	-1.03890807	-1.64218751	-1.64979389	-0.00622723	-1.6248738	-0.00677857	-0.0052512	-0.78771013
	**P-Value**		-	2.61E-09	1.02E-08	1.48E-16	1.11E-15	0.94236369	2.14E-09	1.36E-13	2.36E-16	0.96470898	2.65E-19	0.96320463	0.98172101	1.56E-06
	**Beta**	AFR	-	0.46669	-1.8809	0.52649	0.43183	-0.6602	-1.81983	0.38734	0.39481	-0.6721	1.17908	-0.66443	-0.78223	-1.16196
		NAT	-	-0.44386	-0.57927	-0.36448	-0.35157	-0.89629	-0.50288	-0.45564	-0.35151	-0.03602	-0.35779	0.15016	-0.89573	-0.76314
		EUR	-	-0.34344	1.04945	-0.16805	-0.17775	-1.24061	0.12855	-0.32297	-0.14661	-1.40229	-0.18783	-1.39417	-1.20038	1.16298
	**P-Value**		-	0.15297	< 0.001	0.02406	0.07374	0.01503	< 0.001	0.13158	0.08949	8.00E-05	< 0.001	0.00019	0.00455	< 0.001
			-	0.22376	0.06003	0.4355	0.46517	0.00161	0.19781	0.38148	0.44086	0.90949	0.38215	0.64708	0.0018	0.0291
			-	0.27927	< 0.001	0.45969	0.44779	< 0.001	0.50553	0.20033	0.51661	< 0.001	0.35615	< 0.001	< 0.001	< 0.001
	**R^2**		-	0.47515	0.98069	0.61468	0.53283	0.58497	0.94703	0.59497	0.54464	0.85953	0.89775	0.85149	0.53449	0.96084
	**AICc**		-	-50.50499	-31.98117	-49.42178	-48.78828	-37.8858	-31.40278	-31.28289	-32.74703	-29.40796	-34.01396	-28.26338	-37.75102	-27.07103
			-													
AFR+NAT+EAS																
	**Intercept**		-	-2.04524745	0.21938194	-1.8645632	-1.87261908	-1.22418863	-0.91035171	-1.9651579	-1.79640023	-1.40852194	-1.81270219	-1.40095559	-1.20563804	0.37527236
	**P-Value**		-	4.38E-60	0.00042006	1.40E-90	1.42E-85	5.93E-32	1.63E-30	2.97E-66	1.40E-75	4.24E-71	2.19E-93	3.08E-65	1.34E-30	1.42E-07
	**Beta**	AFR	-	0.81013	-2.93035	0.69454	0.60958	0.58042	-1.94838	0.71031	0.54141	0.73019	1.3669	0.72975	0.41816	-2.32495
		NAT	-	-0.10042	-1.6287	-0.19643	-0.17383	0.34432	-0.63144	-0.13267	-0.20491	1.36626	-0.16997	1.54431	0.30465	-1.92611
		EAS	-	0.34344	-1.04945	0.16805	0.17774	1.24062	-0.12856	0.32297	0.14661	1.40231	0.18783	1.39419	1.2004	-1.16299
	**P-Value**		-	0.00017	< 0.001	1.00E-05	0.00014	0.00289	< 0.001	6.00E-05	0.00053	< 0.001	< 0.001	< 0.001	0.03581	< 0.001
			-	0.71122	< 0.001	0.66532	0.71021	0.0998	0.09253	0.79593	0.64428	1.00E-05	0.67025	< 0.001	0.14981	< 0.001
			-	0.27928	< 0.001	0.4597	0.4478	< 0.001	0.50551	0.20033	0.51662	< 0.001	0.35616	< 0.001	< 0.001	< 0.001
	**R^2**		-	0.47515	0.98069	0.61468	0.53283	0.58497	0.94703	0.59497	0.54464	0.85953	0.89775	0.85148	0.53449	0.96084
	**AICc**		-	-50.50496	-31.98051	-49.42175	-48.78824	-37.88574	-31.40283	-31.28288	-32.74702	-29.40764	-34.01393	-28.26309	-37.75093	-27.07037
			-													
AFR+EUR+EAS																
	**Intercept**		-	-2.14566474	-1.40936775	-2.06097892	-2.04643495	-0.87986946	-1.54177312	-2.09782977	-2.0013029	-0.04223764	-1.98266256	0.1433885	-0.90098283	-1.55088882
	**P-Value**		-	2.36E-22	7.25E-08	4.05E-07	1.04E-06	1.03E-07	5.25E-06	4.51E-06	4.47E-07	0.87777272	2.49E-08	0.61298285	7.44E-08	1.87E-07
	**Beta**	AFR	-	0.91055	-1.3016	0.89096	0.78339	0.2361	-1.31696	0.84298	0.74632	-0.63609	1.53686	-0.8146	0.1135	-0.39878
		EUR	-	0.10042	1.62875	0.19641	0.17381	-0.34432	0.63142	0.13267	0.2049	-1.36628	0.16996	-1.54434	-0.30465	1.92616
		EAS	-	0.44386	0.57931	0.36446	0.35156	0.8963	0.50286	0.45565	0.35151	0.03601	0.35779	-0.15017	0.89574	0.76319
	**P-Value**		-	8.00E-04	4.00E-05	0.03654	0.07483	0.28926	0.0011	0.07863	0.07353	0.03039	3.00E-05	0.0073	0.6178	0.23421
			-	0.71123	< 0.001	0.66535	0.71024	0.0998	0.09255	0.79593	0.64429	1.00E-05	0.67027	< 0.001	0.14981	< 0.001
			-	0.22376	0.06001	0.43553	0.4652	0.00161	0.19784	0.38148	0.44087	0.90952	0.38217	0.64706	0.0018	0.02909
	**R^2**		-	0.47515	0.98069	0.61468	0.53283	0.58497	0.94703	0.59497	0.54464	0.85953	0.89775	0.85149	0.53449	0.96085
	**AICc**		-	-50.50495	-31.98153	-49.42171	-48.78821	-37.88576	-31.40259	-31.28288	-32.747	-29.40795	-34.01391	-28.26341	-37.75095	-27.07146

			rs8259	rs6757	rs8637	rs12608994	rs1803202	rs11879069	rs4919862	rs6758	rs28992474	rs2072310	rs3764937	rs4682	rs2072309	rs2074962
			-													
NAT+EUR+EAS																
	**Intercept**		-	-1.23511993	-2.71097657	-1.17002222	-1.26304168	-0.64376969	-2.85872365	-1.25484836	-1.25498718	-0.67832803	-0.4457988	-0.67120979	-0.78747972	-1.94967738
	**P-Value**		-	1.96E-15	7.34E-67	9.39E-27	5.56E-28	1.16E-05	4.65E-48	7.41E-23	8.66E-28	1.07E-12	5.68E-07	1.39E-11	1.97E-07	8.56E-46
	**Beta**	NAT	-	-0.91055	1.30161	-0.89096	-0.7834	-0.2361	1.31691	-0.84297	-0.74631	0.63607	-1.53685	0.81458	-0.1135	0.39881
		EUR	-	-0.81013	2.93035	-0.69454	-0.60958	-0.58042	1.94836	-0.71031	-0.54141	-0.73019	-1.3669	-0.72974	-0.41816	2.32494
		EAS	-	-0.46669	1.88093	-0.5265	-0.43184	0.6602	1.81983	-0.38734	-0.39481	0.67211	-1.17909	0.66444	0.78223	1.16199
	**P-Value**		-	8.00E-04	4.00E-05	0.03654	0.07483	0.28925	0.0011	0.07863	0.07353	0.0304	3.00E-05	0.0073	0.61779	0.23417
			-	0.00017	< 0.001	1.00E-05	0.00014	0.00289	< 0.001	6.00E-05	0.00053	< 0.001	< 0.001	< 0.001	0.03581	< 0.001
			-	0.15297	< 0.001	0.02406	0.07373	0.01503	< 0.001	0.13158	0.08949	8.00E-05	< 0.001	0.00019	0.00455	< 0.001
	**R^2**		-	0.47515	0.9807	0.61468	0.53283	0.58497	0.94703	0.59497	0.54465	0.85953	0.89775	0.85149	0.53449	0.96085
	**AICc**		-	-50.50503	-31.98235	-49.42188	-48.78836	-37.88577	-31.4021	-31.2829	-32.74706	-29.4078	-34.01412	-28.26324	-37.75096	-27.07169
			-													
AFR+NAT+EUR+EAS																
	**Intercept**		-	7693.35292	-15569.0733	8478.22676	9411.40239	2564.48481	14599.9277	1269.77386	5039.32565	9223.82296	6846.45053	9076.84239	4788.37205	-18388.7674
	**P-Value**		-	0.55639153	0.00410234	0.35429416	0.31933294	0.81744555	0.06114996	0.92138644	0.64287168	0.27708806	0.48140594	0.30621462	0.66818902	0.00244587
	**Beta**	AFR	-	-7694.60203	15566.3775	-8479.41483	-9412.68546	-2565.13486	-14602.8201	-1271.02933	-5040.58341	-9224.50779	-6846.89959	-9077.52004	-4789.17145	18386.8304
		NAT	-	-7695.4846	15567.437	-8480.17374	-9413.32093	-2565.36169	-14601.2557	-1271.85351	-5041.25622	-9223.73265	-6848.33593	-9076.56911	-4789.26747	18386.9521
		EUR	-	-7695.38004	15569.2472	-8480.08018	-9413.26292	-2565.70068	-14600.7955	-1271.73551	-5041.10815	-9225.20554	-6848.2453	-9078.21759	-4789.56222	18389.09
		EAS	-	-7695.12792	15568.3909	-8480.0065	-9413.18981	-2564.49233	-14601.1092	-1271.4286	-5041.02509	-9223.91889	-6848.14296	-9076.93738	-4788.42201	18388.154
	**P-Value**		-	0.55633	0.00411	0.35423	0.31927	0.8174	0.0611	0.92131	0.64279	0.27705	0.48138	0.30618	0.66814	0.00245
			-	0.55628	0.00411	0.35418	0.31923	0.81738	0.06112	0.92126	0.64274	0.27709	0.48128	0.30622	0.66813	0.00245
			-	0.55629	0.0041	0.35419	0.31924	0.81736	0.06113	0.92127	0.64275	0.27701	0.48129	0.30614	0.66811	0.00245
			-	0.5563	0.0041	0.3542	0.31925	0.81745	0.06113	0.92129	0.64276	0.27709	0.4813	0.30621	0.66819	0.00245
	**R^2**		-	0.48179	0.98635	0.63224	0.55655	0.58759	0.95955	0.59523	0.55325	0.87611	0.90231	0.86764	0.54182	0.97405
	**AICc**		-	-45.89524	-29.46958	-44.41116	-43.89088	-32.99316	-25.72462	-22.49274	-24.16408	-21.72115	-25.70354	-20.44556	-32.9873	-25.09583
