# Supplementary material for: Human-SARS-CoV-2 interactome and human genetic diversity: TMPRSS2-rs2070788, associated with severe influenza, and its population genetics caveats in Native Americans
Source: Genet Mol Biol. 2021 Aug 25;44(1 Suppl 1):e20200484. doi: 10.1590/1678-4685-GMB-2020-0484 (PMC8387978; doi:10.1590/1678-4685-GMB-2020-0484)
Supplement: Table S4-A - [file 1415-4757-GMB-44-1-s1-e20200484-s8.pdf]

Genetics and Molecular Biology
https://doi.org/10.1590/1678-4685-GMB-2020-0484

Supplementary Material to "Human-SARS-CoV-2 interactome and human genetic diversity: *TMPRSS2*-rs2070788, associated with severe influenza, and its population genetics caveats in Native Americans"

Table S4-A – TMPRSS2 allele frequencies. Bold = functionally relevant SNPs found in our databases; NA = missing data.

SNP	Allele	ACB	Afro	Aimaras	Ancash	Arequipa	Ashaninkas	ASW	Awajun	Ayacucho	Bambui	BEB	Candoshi	CDX	CEU	Chachapoyas	CHB
**rs456298**	**T**	**0.36**	**NA**	**NA**	**NA**	**NA**	**NA**	**0.34**	**NA**	**NA**	**0.18**	**0.38**	**NA**	**0.73**	**0.16**	**NA**	**0.53**
**rs12329760**	**T**	**0.2812**	**0.2021**	**0**	**0.1111**	**0.1087**	**0**	**0.3115**	**0.1304**	**0.05714**	**0.1665**	**0.2907**	**0.09375**	**0.2957**	**0.2323**	**0.1**	**0.4126**
**rs2276205**	**G**	**0.125**	**0.1064**	**0**	**0**	**0.04348**	**0**	**0.09016**	**0.04348**	**0.01429**	**0.06736**	**0.05814**	**0.0625**	**0.1022**	**0.06061**	**0.025**	**0.2282**
**rs383510**	**T**	**0.3594**	**NA**	**NA**	**NA**	**NA**	**NA**	**0.3852**	**NA**	**NA**	**NA**	**0.4535**	**NA**	**0.4194**	**0.5051**	**NA**	**0.2718**
**rs2070788**	**G**	**0.3229**	**0.383**	**0.9375**	**0.6944**	**0.6957**	**0.8286**	**0.3279**	**0.6957**	**0.7143**	**0.4463**	**0.4186**	**0.5938**	**0.4086**	**0.4899**	**0.7125**	**0.2767**
rs149270377	A	0	NA	NA	NA	NA	NA	0	NA	NA	NA	0	NA	0	0	NA	0
rs75833467	A	0.005208	NA	NA	NA	NA	NA	0.01639	NA	NA	NA	0.005814	NA	0	0.09091	NA	0
rs569429288	A	0	NA	NA	NA	NA	NA	0	NA	NA	NA	0	NA	0	0	NA	0
rs554852594	C	0	NA	NA	NA	NA	NA	0	NA	NA	NA	0	NA	0	0	NA	0
rs537997349	G	0.01562	NA	NA	NA	NA	NA	0.008197	NA	NA	NA	0	NA	0	0	NA	0
rs569432639	C	0	NA	NA	NA	NA	NA	0	NA	NA	NA	0	NA	0	0	NA	0
rs535019926	C	0	NA	NA	NA	NA	NA	0	NA	NA	NA	0	NA	0	0	NA	0
rs368646465	T	0	NA	NA	NA	NA	NA	0	NA	NA	NA	0	NA	0	0	NA	0
rs9305744	A	0.3906	NA	NA	NA	NA	NA	0.4262	NA	NA	0.1978	0.3256	NA	0.3495	0.2121	NA	0.4272
rs148910500	T	0	NA	NA	NA	NA	NA	0	NA	NA	NA	0	NA	0	0	NA	0
rs142710225	T	0.01042	NA	NA	NA	NA	NA	0	NA	NA	NA	0	NA	0	0	NA	0
rs368735421	T	0	NA	NA	NA	NA	NA	0	NA	NA	NA	0	NA	0	0	NA	0
rs140458174	A	0	NA	NA	NA	NA	NA	0	NA	NA	NA	0	NA	0.02151	0	NA	0.004854
rs532232636	T	0	NA	NA	NA	NA	NA	0	NA	NA	NA	0	NA	0	0	NA	0
rs381179	T	0	NA	NA	NA	NA	NA	0	NA	NA	NA	0	NA	0.01075	0	NA	0
rs572410855	A	0	NA	NA	NA	NA	NA	0	NA	NA	NA	0	NA	0	0	NA	0
rs111620846	A	0.005208	NA	NA	NA	NA	NA	0.008197	NA	NA	NA	0	NA	0	0	NA	0
rs377496737	T	0	NA	NA	NA	NA	NA	0	NA	NA	NA	0	NA	0	0	NA	0
rs533552237	A	0.005208	NA	NA	NA	NA	NA	0	NA	NA	NA	0	NA	0	0	NA	0
rs76315847	A	0.005208	0.02128	0	0	0	0	0.01639	0	0	0	0	0	0	0	0	0
rs377348479	A	0	NA	NA	NA	NA	NA	0	NA	NA	NA	0	NA	0	0	NA	0
rs180818774	C	0.005208	NA	NA	NA	NA	NA	0	NA	NA	NA	0	NA	0	0	NA	0
rs462471	A	0.3646	0.3936	0.7812	0.6	0.587	0.8088	0.3443	0.6304	0.6	0.1907	0.3779	0.625	0.7312	0.1616	0.6375	0.534
rs554357472	A	0	NA	NA	NA	NA	NA	0	NA	NA	NA	0	NA	0	0	NA	0.004854
rs117652812	T	0	NA	NA	NA	NA	NA	0	NA	NA	NA	0.005814	NA	0.04301	0	NA	0.004854
rs56097233	CAG	0.4323	NA	NA	NA	NA	NA	0.4508	NA	NA	NA	0.3837	NA	0.6828	0.2778	NA	0.6699
rs573178525	A	0	NA	NA	NA	NA	NA	0	NA	NA	NA	0	NA	0	0	NA	0
rs186168224	T	0	NA	NA	NA	NA	NA	0	NA	NA	NA	0	NA	0	0	NA	0
rs551103429	T	0	NA	NA	NA	NA	NA	0	NA	NA	NA	0	NA	0	0	NA	0
rs462574	A	0.1458	0.266	0.7812	0.5278	0.5435	0.8143	0.1639	0.5	0.5714	0.1085	0.2849	0.5312	0.7097	0.0303	0.55	0.4709
rs543681861	C	0.005208	NA	NA	NA	NA	NA	0	NA	NA	NA	0	NA	0	0	NA	0
rs557632131	T	0.005208	NA	NA	NA	NA	NA	0	NA	NA	NA	0	NA	0.01613	0	NA	0
rs186425543	G	0	NA	NA	NA	NA	NA	0	NA	NA	NA	0	NA	0	0	NA	0

SNP	Allele	ACB	Afro	Aimaras	Ancash	Arequipa	Ashaninkas	ASW	Awajun	Ayacucho	Bambui	BEB	Candoshi	CDX	CEU	Chachapoyas	CHB
rs542228195	C	0	NA	NA	NA	NA	NA	0	NA	NA	NA	0	NA	0	0	NA	0
rs144052153	G	0	NA	NA	NA	NA	NA	0	NA	NA	NA	0	NA	0	0	NA	0
rs374377315	T	0	NA	NA	NA	NA	NA	0	NA	NA	NA	0.01163	NA	0	0	NA	0
rs73357664	A	0.07292	NA	NA	NA	NA	NA	0.02459	NA	NA	NA	0	NA	0	0	NA	0
rs373196115	A	0	NA	NA	NA	NA	NA	0	NA	NA	NA	0	NA	0	0	NA	0
rs181107522	A	0.005208	NA	NA	NA	NA	NA	0	NA	NA	NA	0	NA	0	0	NA	0
rs569716669	C	0	NA	NA	NA	NA	NA	0	NA	NA	NA	0	NA	0	0	NA	0
rs535834505	G	0	NA	NA	NA	NA	NA	0.008197	NA	NA	NA	0	NA	0	0	NA	0
rs28707508	A	0.3021	NA	NA	NA	NA	NA	0.2541	NA	NA	NA	0.2035	NA	0.03763	0.3889	NA	0.009709
rs458280	T	0.005208	NA	NA	NA	NA	NA	0.02459	NA	NA	NA	0.08721	NA	0.4032	0.02525	NA	0.267
rs567208488	CAG	0	NA	NA	NA	NA	NA	0	NA	NA	NA	0.005814	NA	0	0	NA	0
rs186510586	C	0	NA	NA	NA	NA	NA	0	NA	NA	NA	0	NA	0	0	NA	0
rs140532244	C	0	NA	NA	NA	NA	NA	0.008197	NA	NA	NA	0	NA	0	0	NA	0
rs193019598	A	0	NA	NA	NA	NA	NA	0	NA	NA	NA	0	NA	0	0	NA	0
rs533708336	A	0	NA	NA	NA	NA	NA	0	NA	NA	NA	0	NA	0	0	NA	0
rs188198121	A	0	NA	NA	NA	NA	NA	0	NA	NA	NA	0	NA	0	0.005051	NA	0
rs142119028	T	0	NA	NA	NA	NA	NA	0.008197	NA	NA	NA	0	NA	0	0	NA	0
rs141128014	G	0	NA	NA	NA	NA	NA	0	NA	NA	NA	0	NA	0	0	NA	0
rs2838039	C	0.4271	NA	NA	NA	NA	NA	0.4098	NA	NA	NA	0.3023	NA	0.2849	0.2424	NA	0.4029
rs558683527	C	0	NA	NA	NA	NA	NA	0	NA	NA	NA	0	NA	0	0	NA	0
rs545307151	G	0.005208	NA	NA	NA	NA	NA	0.008197	NA	NA	NA	0	NA	0	0	NA	0
rs9976780	C	0.4375	NA	NA	NA	NA	NA	0.418	NA	NA	0.2874	0.3721	NA	0.7419	0.2778	NA	0.6845
rs553788077	A	0	NA	NA	NA	NA	NA	0	NA	NA	NA	0	NA	0	0	NA	0
rs539639766	C	0	NA	NA	NA	NA	NA	0	NA	NA	NA	0	NA	0	0	NA	0
rs543253134	A	0.01562	NA	NA	NA	NA	NA	0	NA	NA	NA	0	NA	0	0	NA	0
rs79971314	A	0.2083	NA	NA	NA	NA	NA	0.1066	NA	NA	NA	0.005814	NA	0	0	NA	0
rs148719900	T	0.01042	NA	NA	NA	NA	NA	0	NA	NA	NA	0	NA	0	0	NA	0
rs144948620	A	0	NA	NA	NA	NA	NA	0	NA	NA	NA	0	NA	0	0.0202	NA	0
rs536525396	A	0	NA	NA	NA	NA	NA	0	NA	NA	NA	0	NA	0	0	NA	0
rs548341299	C	0	NA	NA	NA	NA	NA	0	NA	NA	NA	0	NA	0	0	NA	0
rs139816990	A	0.005208	NA	NA	NA	NA	NA	0.008197	NA	NA	NA	0	NA	0	0	NA	0
rs536147878	A	0	NA	NA	NA	NA	NA	0	NA	NA	NA	0.005814	NA	0	0	NA	0
rs564793560	G	0.02083	NA	NA	NA	NA	NA	0.008197	NA	NA	NA	0.005814	NA	0	0	NA	0
rs192011400	T	0	NA	NA	NA	NA	NA	0	NA	NA	NA	0	NA	0	0	NA	0
rs531024324	A	0	NA	NA	NA	NA	NA	0	NA	NA	NA	0	NA	0	0.005051	NA	0
rs192666663	A	0	NA	NA	NA	NA	NA	0	NA	NA	NA	0	NA	0	0	NA	0.004854
rs138282462	T	0	NA	NA	NA	NA	NA	0	NA	NA	NA	0	NA	0	0	NA	0.004854
rs143109187	G	0	NA	NA	NA	NA	NA	0	NA	NA	NA	0	NA	0	0	NA	0.004854
rs150014829	A	0	NA	NA	NA	NA	NA	0	NA	NA	NA	0	NA	0	0	NA	0
rs34561135	A	0	NA	NA	NA	NA	NA	0.01639	NA	NA	NA	0.02326	NA	0	0.0404	NA	0
rs11088551	G	0.3021	NA	NA	NA	NA	NA	0.3033	NA	NA	NA	0.2326	NA	0.03763	0.4091	NA	0.009709
rs200169208	A	0	NA	NA	NA	NA	NA	0	NA	NA	NA	0	NA	0	0	NA	0
rs566903241	C	0	NA	NA	NA	NA	NA	0	NA	NA	NA	0	NA	0	0	NA	0
rs146132415	A	0	NA	NA	NA	NA	NA	0	NA	NA	NA	0	NA	0	0	NA	0
rs61735791	T	0	NA	NA	NA	NA	NA	0	NA	NA	NA	0	NA	0	0.005051	NA	0

SNP	Allele	ACB	Afro	Aimaras	Ancash	Arequipa	Ashaninkas	ASW	Awajun	Ayacucho	Bambui	BEB	Candoshi	CDX	CEU	Chachapoyas	CHB
rs571587918	C	0	NA	NA	NA	NA	NA	0	NA	NA	NA	0	NA	0	0	NA	0
rs78459594	C	NA	0	0	0	0	0	NA	0	0	NA	NA	0	NA	NA	0	NA
rs389001	G	0.3073	NA	NA	NA	NA	NA	0.2377	NA	NA	NA	0.2442	NA	0.371	0.01515	NA	0.2767
rs200164183	T	0	NA	NA	NA	NA	NA	0	NA	NA	NA	0	NA	0.01075	0	NA	0
rs548354821	C	0	NA	NA	NA	NA	NA	0	NA	NA	NA	0	NA	0	0	NA	0.004854
rs2187238	C	0.04688	0.234	0.1562	0.2083	0.1739	0.05714	0.08197	0.1304	0.2429	0.1921	0.06395	0.1875	0.01613	0.2172	0.1625	0.06796
rs556456416	C	0	NA	NA	NA	NA	NA	0	NA	NA	NA	0	NA	0	0	NA	0
rs181414852	G	0	NA	NA	NA	NA	NA	0	NA	NA	NA	0	NA	0	0	NA	0
rs553787982	A	0.005208	NA	NA	NA	NA	NA	0	NA	NA	NA	0	NA	0	0	NA	0
rs417888	A	0.1823	NA	NA	NA	NA	NA	0.2049	NA	NA	NA	0.4535	NA	0.5	0.5101	NA	0.3204
rs189793246	G	0	NA	NA	NA	NA	NA	0	NA	NA	NA	0.005814	NA	0.01613	0	NA	0.009709
rs556371948	T	0	NA	NA	NA	NA	NA	0	NA	NA	NA	0	NA	0	0	NA	0
rs546273967	T	0	NA	NA	NA	NA	NA	0	NA	NA	NA	0	NA	0	0	NA	0
rs76000363	A	0.09896	NA	NA	NA	NA	NA	0.123	NA	NA	NA	0.08721	NA	0.02151	0.1313	NA	0.06311
rs557639119	A	0	NA	NA	NA	NA	NA	0	NA	NA	NA	0	NA	0	0.0101	NA	0
rs563507800	A	0	NA	NA	NA	NA	NA	0	NA	NA	NA	0	NA	0	0	NA	0
rs148125094	T	NA	NA	NA	NA	NA	NA	NA	NA	NA	NA	NA	NA	NA	NA	NA	NA
rs73372161	A	0.1406	NA	NA	NA	NA	NA	0.08197	NA	NA	0.01739	0.005814	NA	0	0	NA	0
rs555995855	C	0	NA	NA	NA	NA	NA	0	NA	NA	NA	0.06977	NA	0	0	NA	0
rs11281229	TCCAGG	0.3281	NA	NA	NA	NA	NA	0.2541	NA	NA	NA	0.2151	NA	0.03763	0.3838	NA	0.009709
rs559098267	T	0	NA	NA	NA	NA	NA	0	NA	NA	NA	0	NA	0	0	NA	0
rs191693032	G	0	NA	NA	NA	NA	NA	0	NA	NA	NA	0	NA	0	0	NA	0
rs187648498	C	0	NA	NA	NA	NA	NA	0	NA	NA	NA	0	NA	0	0	NA	0
rs541109823	T	0	NA	NA	NA	NA	NA	0	NA	NA	NA	0	NA	0	0	NA	0
rs559668949	G	0	NA	NA	NA	NA	NA	0	NA	NA	NA	0	NA	0	0	NA	0
rs113670863	T	0	NA	NA	NA	NA	NA	0	NA	NA	NA	0	NA	0	0	NA	0
rs545494331	C	0	NA	NA	NA	NA	NA	0	NA	NA	NA	0	NA	0.01075	0	NA	0
rs528691645	A	0	NA	NA	NA	NA	NA	0	NA	NA	NA	0	NA	0	0	NA	0
rs558543141	T	0	NA	NA	NA	NA	NA	0	NA	NA	NA	0	NA	0	0	NA	0
rs145877432	A	0	NA	NA	NA	NA	NA	0	NA	NA	NA	0	NA	0	0	NA	0
rs531615786	C	0	NA	NA	NA	NA	NA	0	NA	NA	NA	0	NA	0	0	NA	0
rs570264808	A	0.005208	NA	NA	NA	NA	NA	0	NA	NA	NA	0	NA	0	0	NA	0
rs371012018	A	0	NA	NA	NA	NA	NA	0	NA	NA	NA	0	NA	0	0	NA	0
rs534326511	T	0	NA	NA	NA	NA	NA	0	NA	NA	NA	0	NA	0	0	NA	0.004854
rs200744510	C	NA	NA	NA	NA	NA	NA	NA	NA	NA	NA	NA	NA	NA	NA	NA	NA
rs77996454	A	0.03125	NA	NA	NA	NA	NA	0.04098	NA	NA	0.006298	0	NA	0	0	NA	0
rs140259165	T	0	NA	NA	NA	NA	NA	0	NA	NA	NA	0	NA	0	0	NA	0
rs58978895	T	0.1979	NA	NA	NA	NA	NA	0.1639	NA	NA	NA	0.005814	NA	0	0	NA	0
rs564981706	T	0	NA	NA	NA	NA	NA	0	NA	NA	NA	0	NA	0	0	NA	0
rs149173609	T	0	NA	NA	NA	NA	NA	0	NA	NA	NA	0	NA	0	0	NA	0.009709
rs559934709	T	0	NA	NA	NA	NA	NA	0	NA	NA	NA	0	NA	0	0	NA	0
rs569899862	A	0	NA	NA	NA	NA	NA	0	NA	NA	NA	0	NA	0	0	NA	0
rs540844961	G	0	NA	NA	NA	NA	NA	0	NA	NA	NA	0	NA	0	0	NA	0
rs189582211	A	0	NA	NA	NA	NA	NA	0.008197	NA	NA	NA	0	NA	0	0	NA	0
rs553097317	T	0	NA	NA	NA	NA	NA	0	NA	NA	NA	0.005814	NA	0	0	NA	0

SNP	Allele	ACB	Afro	Aimaras	Ancash	Arequipa	Ashaninkas	ASW	Awajun	Ayacucho	Bambui	BEB	Candoshi	CDX	CEU	Chachapoyas	CHB
rs563693995	G	0	NA	NA	NA	NA	NA	0	NA	NA	NA	0	NA	0.005376	0	NA	0
rs2298658	T	0	NA	NA	NA	NA	NA	0	NA	NA	NA	0	NA	0	0	NA	0.004854
rs532882572	T	0	NA	NA	NA	NA	NA	0	NA	NA	NA	0	NA	0	0	NA	0
rs145347702	C	0	NA	NA	NA	NA	NA	0	NA	NA	NA	0	NA	0	0	NA	0
rs148988435	A	0.005208	NA	NA	NA	NA	NA	0	NA	NA	NA	0	NA	0	0	NA	0
rs561557938	A	0	NA	NA	NA	NA	NA	0	NA	NA	NA	0.005814	NA	0	0	NA	0
rs140230703	G	0.01042	NA	NA	NA	NA	NA	0.01639	NA	NA	NA	0	NA	0	0	NA	0
rs141232947	G	0	NA	NA	NA	NA	NA	0	NA	NA	NA	0	NA	0	0	NA	0
rs61735794	T	0	NA	NA	NA	NA	NA	0	NA	NA	NA	0	NA	0	0.03535	NA	0
rs117888036	A	0	NA	NA	NA	NA	NA	0	NA	NA	NA	0	NA	0	0	NA	0.004854
rs538864203	A	0.005208	NA	NA	NA	NA	NA	0	NA	NA	NA	0	NA	0	0	NA	0
rs540165785	A	0	NA	NA	NA	NA	NA	0	NA	NA	NA	0	NA	0	0	NA	0
rs79517809	C	0	NA	NA	NA	NA	NA	0	NA	NA	NA	0	NA	0	0	NA	0
rs144988776	T	0	NA	NA	NA	NA	NA	0	NA	NA	NA	0	NA	0	0.0202	NA	0
rs565946159	C	0	NA	NA	NA	NA	NA	0	NA	NA	NA	0	NA	0	0	NA	0
rs574894406	G	0	NA	NA	NA	NA	NA	0	NA	NA	NA	0	NA	0	0	NA	0
rs145570856	A	0.04688	NA	NA	NA	NA	NA	0.008197	NA	NA	NA	0	NA	0	0	NA	0
rs146142989	A	0	NA	NA	NA	NA	NA	0	NA	NA	NA	0	NA	0	0	NA	0
rs556966925	C	0.01562	NA	NA	NA	NA	NA	0	NA	NA	NA	0	NA	0	0	NA	0
rs573704321	T	0	NA	NA	NA	NA	NA	0	NA	NA	NA	0	NA	0	0	NA	0
rs376074355	T	0	NA	NA	NA	NA	NA	0	NA	NA	NA	0.005814	NA	0	0	NA	0
rs61325328	G	0	NA	NA	NA	NA	NA	0	NA	NA	NA	0	NA	0	0	NA	0
rs9979311	G	0.4062	NA	NA	NA	NA	NA	0.3934	NA	NA	NA	0.3023	NA	0.2903	0.2424	NA	0.4078
rs538655114	A	0	NA	NA	NA	NA	NA	0	NA	NA	NA	0	NA	0	0	NA	0
rs150066796	A	0	NA	NA	NA	NA	NA	0	NA	NA	NA	0	NA	0	0	NA	0
rs201093031	G	0	NA	NA	NA	NA	NA	0	NA	NA	NA	0	NA	0.005376	0	NA	0
rs378616	C	0.2708	NA	NA	NA	NA	NA	0.3279	NA	NA	0.2593	0.2558	NA	0.3333	0.2677	NA	0.335
rs573152591	A	0.005208	NA	NA	NA	NA	NA	0	NA	NA	NA	0	NA	0	0	NA	0
rs4818241	A	0.01562	NA	NA	NA	NA	NA	0.02459	NA	NA	NA	0.07558	NA	0.4516	0.0303	NA	0.2767
rs189181802	T	0	NA	NA	NA	NA	NA	0.008197	NA	NA	NA	0	NA	0	0	NA	0
rs566323147	A	0	NA	NA	NA	NA	NA	0	NA	NA	NA	0	NA	0	0	NA	0
rs184164037	C	0.02604	NA	NA	NA	NA	NA	0.03279	NA	NA	NA	0	NA	0	0	NA	0
rs77675406	A	0.07292	0.06383	0	0.1111	0.04348	0	0.09016	0.1304	0.02857	0.06623	0.08721	0.09375	0.02151	0.1313	0.075	0.06311
rs528276471	G	0	NA	NA	NA	NA	NA	0.008197	NA	NA	NA	0	NA	0	0	NA	0
rs552912416	T	0	NA	NA	NA	NA	NA	0	NA	NA	NA	0	NA	0	0	NA	0
rs572279833	A	0	NA	NA	NA	NA	NA	0	NA	NA	NA	0	NA	0	0	NA	0
rs559830930	G	0	NA	NA	NA	NA	NA	0	NA	NA	NA	0	NA	0	0	NA	0
rs2298660	T	0.4062	NA	NA	NA	NA	NA	0.3443	NA	NA	NA	0.25	NA	0.172	0.1818	NA	0.2961
rs527684898	G	0	NA	NA	NA	NA	NA	0	NA	NA	NA	0	NA	0	0	NA	0
rs181332845	G	0.03125	NA	NA	NA	NA	NA	0.008197	NA	NA	NA	0	NA	0	0	NA	0
rs182175876	G	0	NA	NA	NA	NA	NA	0	NA	NA	NA	0	NA	0	0	NA	0
rs539218228	A	0	NA	NA	NA	NA	NA	0	NA	NA	NA	0	NA	0	0	NA	0
rs116865960	A	0.02083	NA	NA	NA	NA	NA	0.02459	NA	NA	NA	0	NA	0	0.03535	NA	0
rs183398094	C	0	NA	NA	NA	NA	NA	0	NA	NA	NA	0	NA	0	0	NA	0
rs567340892	A	0.005208	NA	NA	NA	NA	NA	0	NA	NA	NA	0	NA	0	0	NA	0

SNP	Allele	ACB	Afro	Aimaras	Ancash	Arequipa	Ashaninkas	ASW	Awajun	Ayacucho	Bambui	BEB	Candoshi	CDX	CEU	Chachapoyas	CHB
rs190125027	T	0	NA	NA	NA	NA	NA	0.008197	NA	NA	NA	0	NA	0	0	NA	0
rs75430506	A	0	NA	NA	NA	NA	NA	0	NA	NA	NA	0	NA	0.005376	0	NA	0
rs138812485	A	0	NA	NA	NA	NA	NA	0	NA	NA	NA	0	NA	0	0	NA	0.004854
rs56066678	T	0.3594	0.4043	0.0625	0.1111	0.2174	0.05714	0.3115	0.1304	0.2571	0.3022	0.2267	0.25	0.2473	0.2222	0.1625	0.3107
rs561063944	A	0	NA	NA	NA	NA	NA	0	NA	NA	NA	0	NA	0	0	NA	0
rs17854725	G	0.3698	NA	NA	NA	NA	NA	0.4016	NA	NA	NA	0.3895	NA	0.03226	0.5455	NA	0.1748
rs533050705	A	0	NA	NA	NA	NA	NA	0	NA	NA	NA	0	NA	0	0	NA	0
rs545910743	C	0	NA	NA	NA	NA	NA	0.008197	NA	NA	NA	0	NA	0	0	NA	0
rs149021153	G	0.01562	NA	NA	NA	NA	NA	0	NA	NA	NA	0	NA	0	0	NA	0
rs371583288	A	0	NA	NA	NA	NA	NA	0	NA	NA	NA	0	NA	0	0	NA	0
rs552174617	C	0	NA	NA	NA	NA	NA	0	NA	NA	NA	0	NA	0	0	NA	0
rs144157441	A	0.01562	NA	NA	NA	NA	NA	0.008197	NA	NA	NA	0	NA	0	0	NA	0
rs559811756	A	0.02604	NA	NA	NA	NA	NA	0.03279	NA	NA	NA	0	NA	0	0	NA	0
rs536642840	A	0	NA	NA	NA	NA	NA	0	NA	NA	NA	0	NA	0	0	NA	0.004854
rs572530227	T	0	NA	NA	NA	NA	NA	0	NA	NA	NA	0	NA	0	0	NA	0
rs551045243	A	0	NA	NA	NA	NA	NA	0	NA	NA	NA	0	NA	0	0	NA	0
rs374886738	C	0	NA	NA	NA	NA	NA	0	NA	NA	NA	0	NA	0	0	NA	0
rs557714443	A	0	NA	NA	NA	NA	NA	0	NA	NA	NA	0	NA	0	0	NA	0
rs402303	C	0.3906	0.4894	0.2188	0.2639	0.3261	0.1765	0.377	0.3478	0.4143	0.656	0.6163	0.3438	0.328	0.7323	0.375	0.3738
rs544822322	C	0	NA	NA	NA	NA	NA	0	NA	NA	NA	0	NA	0	0	NA	0
rs186429734	A	0	NA	NA	NA	NA	NA	0	NA	NA	NA	0	NA	0	0	NA	0
rs138056981	T	0	NA	NA	NA	NA	NA	0	NA	NA	NA	0	NA	0	0	NA	0
rs79468500	G	0	NA	NA	NA	NA	NA	0.02459	NA	NA	NA	0.005814	NA	0	0.03535	NA	0
rs2156301	T	0.01562	NA	NA	NA	NA	NA	0.02459	NA	NA	NA	0.06977	NA	0.4516	0.0303	NA	0.2767
rs191228684	T	0	NA	NA	NA	NA	NA	0	NA	NA	NA	0	NA	0	0	NA	0.004854
rs563812695	A	0	NA	NA	NA	NA	NA	0	NA	NA	NA	0	NA	0.01075	0	NA	0
rs8128074	T	0.03646	0.01064	0.4062	0.1111	0.1136	0.3571	0.05738	0.04348	0.1429	0.0911	0.1221	0.2188	0.2419	0.09091	0.3	0.2379
rs560788815	C	0	NA	NA	NA	NA	NA	0	NA	NA	NA	0.005814	NA	0	0	NA	0
rs183851466	T	0	NA	NA	NA	NA	NA	0	NA	NA	NA	0	NA	0	0	NA	0
rs151152524	C	0	NA	NA	NA	NA	NA	0	NA	NA	NA	0	NA	0	0	NA	0
rs113506821	T	0.005208	0.02128	0.09375	0.01389	0	0	0.008197	0.1957	0.08571	0.03505	0.01163	0.0625	0	0.07071	0.0875	0.004854
rs530527676	A	0	NA	NA	NA	NA	NA	0	NA	NA	NA	0	NA	0	0	NA	0
rs550252022	C	0	NA	NA	NA	NA	NA	0	NA	NA	NA	0	NA	0	0	NA	0
rs386638	C	0.02083	NA	NA	NA	NA	NA	0.04098	NA	NA	NA	0.09302	NA	0.5269	0.0303	NA	0.4709
rs148136016	G	0.005208	NA	NA	NA	NA	NA	0	NA	NA	NA	0	NA	0	0	NA	0
rs114848359	T	0.005208	NA	NA	NA	NA	NA	0	NA	NA	NA	0	NA	0	0	NA	0
rs530009764	C	0	NA	NA	NA	NA	NA	0	NA	NA	NA	0	NA	0	0.005051	NA	0
rs371965055	T	0	NA	NA	NA	NA	NA	0	NA	NA	NA	0.02907	NA	0	0	NA	0
rs183984610	A	0	NA	NA	NA	NA	NA	0	NA	NA	NA	0	NA	0	0	NA	0
rs527305819	A	0	NA	NA	NA	NA	NA	0	NA	NA	NA	0.005814	NA	0	0	NA	0
rs181461974	T	0	NA	NA	NA	NA	NA	0	NA	NA	NA	0	NA	0	0	NA	0
rs566457884	T	0	NA	NA	NA	NA	NA	0	NA	NA	NA	0	NA	0	0	NA	0
rs540070564	A	0	NA	NA	NA	NA	NA	0	NA	NA	NA	0	NA	0	0	NA	0
rs563555305	T	0	NA	NA	NA	NA	NA	0	NA	NA	NA	0	NA	0	0	NA	0.004854
rs151189718	T	0	NA	NA	NA	NA	NA	0	NA	NA	NA	0	NA	0	0	NA	0

SNP	Allele	ACB	Afro	Aimaras	Ancash	Arequipa	Ashaninkas	ASW	Awajun	Ayacucho	Bambui	BEB	Candoshi	CDX	CEU	Chachapoyas	CHB
rs569681994	C	0	NA	NA	NA	NA	NA	0	NA	NA	NA	0	NA	0	0	NA	0
rs143097165	A	0.02604	NA	NA	NA	NA	NA	0.03279	NA	NA	NA	0	NA	0	0	NA	0
rs541200538	G	0	NA	NA	NA	NA	NA	0	NA	NA	NA	0	NA	0	0	NA	0
rs11701576	G	0.1094	NA	NA	NA	NA	NA	0.09836	NA	NA	NA	0.2093	NA	0.2473	0.101	NA	0.3301
rs17001042	A	0.1198	NA	NA	NA	NA	NA	0.05738	NA	NA	NA	0.005814	NA	0	0	NA	0
rs558449333	T	0	NA	NA	NA	NA	NA	0	NA	NA	NA	0	NA	0	0	NA	0
rs569866330	A	0	NA	NA	NA	NA	NA	0	NA	NA	NA	0	NA	0	0	NA	0
rs573234046	T	0	NA	NA	NA	NA	NA	0	NA	NA	NA	0	NA	0	0	NA	0
rs114363287	T	0.01042	NA	NA	NA	NA	NA	0	NA	NA	NA	0	NA	0	0	NA	0
rs55896064	A	0.07292	NA	NA	NA	NA	NA	0.09016	NA	NA	NA	0.08721	NA	0.02151	0.1313	NA	0.06311
rs547961708	T	0	NA	NA	NA	NA	NA	0	NA	NA	NA	0	NA	0	0	NA	0
rs542712985	G	0	NA	NA	NA	NA	NA	0	NA	NA	NA	0	NA	0	0	NA	0
rs117941520	A	0.005208	NA	NA	NA	NA	NA	0	NA	NA	NA	0	NA	0	0.0202	NA	0
rs73903404	G	0.0625	NA	NA	NA	NA	NA	0.04918	NA	NA	NA	0	NA	0	0	NA	0
rs551817968	T	0	NA	NA	NA	NA	NA	0	NA	NA	NA	0	NA	0	0	NA	0
rs550208798	T	0	NA	NA	NA	NA	NA	0	NA	NA	NA	0	NA	0	0	NA	0
rs542471574	A	0	NA	NA	NA	NA	NA	0	NA	NA	NA	0	NA	0	0	NA	0
rs145841350	T	0	NA	NA	NA	NA	NA	0	NA	NA	NA	0	NA	0	0	NA	0
rs7278627	A	0.1354	0.06383	0	0	0	0	0.08197	0	0	0.017	0.005814	0	0	0	0	0
rs185555838	A	0	NA	NA	NA	NA	NA	0.008197	NA	NA	NA	0	NA	0	0	NA	0
rs374666536	T	0	NA	NA	NA	NA	NA	0	NA	NA	NA	0	NA	0	0	NA	0
rs7278739	A	0.2292	NA	NA	NA	NA	NA	0.1475	NA	NA	NA	0.01163	NA	0.005376	0	NA	0
rs555667242	C	0	NA	NA	NA	NA	NA	0	NA	NA	NA	0	NA	0	0	NA	0
rs7364083	G	0.1979	NA	NA	NA	NA	NA	0.2541	NA	NA	NA	0.4186	NA	0.4086	0.4848	NA	0.2718
rs527284833	G	0.02604	NA	NA	NA	NA	NA	0.03279	NA	NA	NA	0	NA	0	0	NA	0
rs139458208	C	0	NA	NA	NA	NA	NA	0	NA	NA	NA	0	NA	0	0	NA	0
rs139001727	G	0	NA	NA	NA	NA	NA	0	NA	NA	NA	0	NA	0	0.005051	NA	0
rs4290734	G	0.08854	NA	NA	NA	NA	NA	0.1885	NA	NA	NA	0.4012	NA	0.01075	0.5051	NA	0.004854
rs2156300	G	0.01562	NA	NA	NA	NA	NA	0.02459	NA	NA	NA	0.06977	NA	0.4516	0.0303	NA	0.2767
rs555328523	T	0	NA	NA	NA	NA	NA	0	NA	NA	NA	0	NA	0.005376	0	NA	0.004854
rs575168295	G	0	NA	NA	NA	NA	NA	0	NA	NA	NA	0	NA	0	0	NA	0
rs145355824	A	0	NA	NA	NA	NA	NA	0	NA	NA	NA	0	NA	0	0	NA	0
rs375827195	G	0	NA	NA	NA	NA	NA	0	NA	NA	NA	0	NA	0	0	NA	0
rs533805020	T	0	NA	NA	NA	NA	NA	0	NA	NA	NA	0	NA	0.005376	0	NA	0
rs536311940	G	0	NA	NA	NA	NA	NA	0	NA	NA	NA	0	NA	0	0	NA	0
rs371046741	A	0	NA	NA	NA	NA	NA	0	NA	NA	NA	0	NA	0	0	NA	0
rs2838042	C	0.1927	0.1915	0	0.09722	0.08696	0.02857	0.1885	0.1304	0.04286	0.1834	0.2674	0.09375	0.3226	0.2424	0.075	0.2184
rs192334131	A	0	NA	NA	NA	NA	NA	0	NA	NA	NA	0	NA	0	0	NA	0
rs557822084	T	0	NA	NA	NA	NA	NA	0	NA	NA	NA	0	NA	0	0	NA	0
rs566208761	C	0	NA	NA	NA	NA	NA	0	NA	NA	NA	0	NA	0	0	NA	0
rs546335233	A	0	NA	NA	NA	NA	NA	0	NA	NA	NA	0	NA	0	0	NA	0
rs184767756	C	0	NA	NA	NA	NA	NA	0	NA	NA	NA	0	NA	0	0	NA	0
rs528259213	A	0	NA	NA	NA	NA	NA	0	NA	NA	NA	0	NA	0	0	NA	0
rs115265507	A	0.02083	NA	NA	NA	NA	NA	0.008197	NA	NA	NA	0	NA	0	0	NA	0
rs200072801	G	0	NA	NA	NA	NA	NA	0	NA	NA	NA	0	NA	0	0	NA	0.004854

SNP	Allele	ACB	Afro	Aimaras	Ancash	Arequipa	Ashaninkas	ASW	Awajun	Ayacucho	Bambui	BEB	Candoshi	CDX	CEU	Chachapoyas	CHB
rs138765307	T	0	NA	NA	NA	NA	NA	0	NA	NA	NA	0	NA	0	0	NA	0
rs532005855	C	0	NA	NA	NA	NA	NA	0	NA	NA	NA	0	NA	0	0	NA	0
rs2838043	T	0.05208	NA	NA	NA	NA	NA	0.08197	NA	NA	NA	0.06395	NA	0.01613	0.2172	NA	0.06796
rs115596471	A	0.05208	NA	NA	NA	NA	NA	0.08197	NA	NA	NA	0	NA	0	0	NA	0
rs574538362	T	0	NA	NA	NA	NA	NA	0.008197	NA	NA	NA	0	NA	0	0	NA	0
rs190618812	A	0	NA	NA	NA	NA	NA	0	NA	NA	NA	0	NA	0.03226	0	NA	0.004854
rs141583878	A	0	NA	NA	NA	NA	NA	0	NA	NA	NA	0	NA	0	0.005051	NA	0
rs571805265	T	0	NA	NA	NA	NA	NA	0	NA	NA	NA	0.005814	NA	0	0	NA	0
rs371497524	A	0	NA	NA	NA	NA	NA	0	NA	NA	NA	0	NA	0	0.005051	NA	0
rs540908584	T	0	NA	NA	NA	NA	NA	0	NA	NA	NA	0	NA	0	0	NA	0
rs73357642	A	0.224	NA	NA	NA	NA	NA	0.1803	NA	NA	NA	0.005814	NA	0	0	NA	0
rs192854367	A	0	NA	NA	NA	NA	NA	0	NA	NA	NA	0	NA	0	0	NA	0
rs371180501	G	0.005208	NA	NA	NA	NA	NA	0.008197	NA	NA	NA	0	NA	0	0	NA	0
rs467375	A	0.1406	0.1489	0.1562	0.05714	0.1522	0.01429	0.1885	0	0.1286	0.3478	0.314	0.0625	0.005376	0.4646	0.1375	0.004854
rs562045100	A	0	NA	NA	NA	NA	NA	0	NA	NA	NA	0	NA	0	0	NA	0
rs34205539	AT	0.02083	NA	NA	NA	NA	NA	0.04098	NA	NA	NA	0.05233	NA	0	0.101	NA	0
rs186418926	A	0	NA	NA	NA	NA	NA	0	NA	NA	NA	0	NA	0	0	NA	0
rs539528622	T	0	NA	NA	NA	NA	NA	0	NA	NA	NA	0.005814	NA	0	0	NA	0
rs145292327	G	0	NA	NA	NA	NA	NA	0	NA	NA	NA	0	NA	0	0	NA	0
rs372405355	C	0	NA	NA	NA	NA	NA	0	NA	NA	NA	0	NA	0	0	NA	0
rs544417878	T	0	NA	NA	NA	NA	NA	0	NA	NA	NA	0.005814	NA	0	0	NA	0
rs559363556	A	0	NA	NA	NA	NA	NA	0	NA	NA	NA	0.005814	NA	0	0	NA	0
rs570467504	C	0	NA	NA	NA	NA	NA	0	NA	NA	NA	0.005814	NA	0	0	NA	0
rs148701953	C	0	NA	NA	NA	NA	NA	0	NA	NA	NA	0	NA	0	0	NA	0
rs569988060	A	0	NA	NA	NA	NA	NA	0	NA	NA	NA	0	NA	0	0	NA	0
rs182254633	G	0	NA	NA	NA	NA	NA	0	NA	NA	NA	0	NA	0	0	NA	0
rs563305848	C	0	NA	NA	NA	NA	NA	0	NA	NA	NA	0	NA	0	0	NA	0
rs528477904	C	0	NA	NA	NA	NA	NA	0	NA	NA	NA	0	NA	0	0	NA	0
rs550874821	G	0.005208	NA	NA	NA	NA	NA	0	NA	NA	NA	0	NA	0	0	NA	0
rs73372193	C	0.2083	NA	NA	NA	NA	NA	0.1639	NA	NA	NA	0.005814	NA	0	0	NA	0
rs552607028	A	0	NA	NA	NA	NA	NA	0	NA	NA	NA	0	NA	0	0	NA	0
rs553861191	T	0	NA	NA	NA	NA	NA	0	NA	NA	NA	0	NA	0	0	NA	0
rs147977027	C	0	NA	NA	NA	NA	NA	0	NA	NA	NA	0	NA	0	0	NA	0
rs533445403	T	0	NA	NA	NA	NA	NA	0	NA	NA	NA	0	NA	0	0	NA	0
rs578005531	T	0	NA	NA	NA	NA	NA	0	NA	NA	NA	0	NA	0	0	NA	0
rs140605461	A	0	NA	NA	NA	NA	NA	0	NA	NA	NA	0	NA	0	0	NA	0
rs544023509	A	0	NA	NA	NA	NA	NA	0	NA	NA	NA	0	NA	0	0	NA	0
rs61735792	A	0	NA	NA	NA	NA	NA	0	NA	NA	NA	0	NA	0	0.01515	NA	0
rs568256706	G	0.005208	NA	NA	NA	NA	NA	0	NA	NA	NA	0	NA	0	0	NA	0
rs8131648	T	0.4844	NA	NA	NA	NA	NA	0.4672	NA	NA	NA	0.407	NA	0.7527	0.2525	NA	0.6893
rs527964326	C	0	NA	NA	NA	NA	NA	0	NA	NA	NA	0	NA	0	0	NA	0
rs191697229	A	0.01042	NA	NA	NA	NA	NA	0	NA	NA	NA	0	NA	0	0	NA	0
rs147359020	A	0	NA	NA	NA	NA	NA	0	NA	NA	NA	0.03488	NA	0	0	NA	0
rs532085624	T	0	NA	NA	NA	NA	NA	0	NA	NA	NA	0.005814	NA	0	0	NA	0
rs577753477	A	0	NA	NA	NA	NA	NA	0	NA	NA	NA	0.005814	NA	0	0	NA	0

SNP	Allele	ACB	Afro	Aimaras	Ancash	Arequipa	Ashaninkas	ASW	Awajun	Ayacucho	Bambui	BEB	Candoshi	CDX	CEU	Chachapoyas	CHB
rs181091055	C	0	NA	NA	NA	NA	NA	0	NA	NA	NA	0	NA	0	0	NA	0.004854
rs546447704	T	0	NA	NA	NA	NA	NA	0	NA	NA	NA	0.005814	NA	0	0	NA	0
rs575335056	CA	0	NA	NA	NA	NA	NA	0	NA	NA	NA	0.005814	NA	0	0	NA	0
rs536763630	A	0	NA	NA	NA	NA	NA	0.008197	NA	NA	NA	0	NA	0	0	NA	0
rs184500277	T	0	NA	NA	NA	NA	NA	0	NA	NA	NA	0	NA	0.01075	0	NA	0
rs569292495	G	0	NA	NA	NA	NA	NA	0	NA	NA	NA	0.005814	NA	0	0	NA	0
rs73372163	A	0.25	NA	NA	NA	NA	NA	0.1885	NA	NA	NA	0.09302	NA	0.02151	0.1313	NA	0.06311
rs554443322	C	0	NA	NA	NA	NA	NA	0	NA	NA	NA	0	NA	0	0	NA	0
rs568346732	T	0	NA	NA	NA	NA	NA	0	NA	NA	NA	0	NA	0	0	NA	0
rs114641598	C	0	NA	NA	NA	NA	NA	0	NA	NA	NA	0	NA	0	0	NA	0
rs549459413	A	0	NA	NA	NA	NA	NA	0	NA	NA	NA	0	NA	0	0	NA	0
rs184380117	A	0	NA	NA	NA	NA	NA	0	NA	NA	NA	0	NA	0	0	NA	0.009709
rs576181035	G	0	NA	NA	NA	NA	NA	0	NA	NA	NA	0	NA	0	0	NA	0
rs201679623	C	0	NA	NA	NA	NA	NA	0	NA	NA	NA	0	NA	0	0	NA	0.004854
rs568846685	A	0	NA	NA	NA	NA	NA	0	NA	NA	NA	0	NA	0	0	NA	0
rs548718178	C	0	NA	NA	NA	NA	NA	0	NA	NA	NA	0	NA	0	0	NA	0
rs537584838	G	0	NA	NA	NA	NA	NA	0	NA	NA	NA	0	NA	0	0	NA	0
rs569259137	A	0	NA	NA	NA	NA	NA	0	NA	NA	NA	0	NA	0	0	NA	0
rs367885466	T	0.01042	NA	NA	NA	NA	NA	0	NA	NA	NA	0	NA	0	0	NA	0
rs2410430	A	0.03646	NA	NA	NA	NA	NA	0.04098	NA	NA	NA	0.06977	NA	0.4516	0.0303	NA	0.2767
rs559637785	A	0	NA	NA	NA	NA	NA	0	NA	NA	NA	0	NA	0	0	NA	0
rs573343651	C	0	NA	NA	NA	NA	NA	0	NA	NA	NA	0.005814	NA	0	0	NA	0
rs34769294	CA	0.1927	NA	NA	NA	NA	NA	0.1803	NA	NA	NA	0.2267	NA	0.3011	0.2374	NA	0.335
rs7364088	A	0.349	NA	NA	NA	NA	NA	0.3852	NA	NA	NA	0.3314	NA	0.3495	0.2576	NA	0.4272
rs551432766	T	0	NA	NA	NA	NA	NA	0	NA	NA	NA	0	NA	0	0	NA	0
rs565299100	G	0	NA	NA	NA	NA	NA	0	NA	NA	NA	0	NA	0	0	NA	0
rs538252674	C	0	NA	NA	NA	NA	NA	0	NA	NA	NA	0	NA	0	0	NA	0
rs368994585	A	0	NA	NA	NA	NA	NA	0	NA	NA	NA	0	NA	0	0	NA	0
rs192709500	A	0	NA	NA	NA	NA	NA	0.008197	NA	NA	NA	0	NA	0	0	NA	0
rs192955773	C	0.02604	NA	NA	NA	NA	NA	0.03279	NA	NA	NA	0	NA	0	0	NA	0
rs371020678	T	0	NA	NA	NA	NA	NA	0	NA	NA	NA	0	NA	0	0	NA	0
rs556435793	A	0	NA	NA	NA	NA	NA	0	NA	NA	NA	0	NA	0	0	NA	0
rs549990870	G	0	NA	NA	NA	NA	NA	0	NA	NA	NA	0	NA	0	0	NA	0
rs146723217	T	0.03125	NA	NA	NA	NA	NA	0.04098	NA	NA	NA	0	NA	0	0	NA	0
rs145738510	C	0	NA	NA	NA	NA	NA	0	NA	NA	NA	0	NA	0	0	NA	0
rs114844880	C	0.005208	NA	NA	NA	NA	NA	0.01639	NA	NA	NA	0	NA	0	0	NA	0
rs370641046	T	0	NA	NA	NA	NA	NA	0	NA	NA	NA	0.005814	NA	0.005376	0	NA	0
rs528452128	GTGTTT	0.005208	NA	NA	NA	NA	NA	0	NA	NA	NA	0	NA	0	0	NA	0
rs11910678	C	0.1198	0.07447	0	0	0	0	0.1148	0	0	0.03086	0.01744	0	0.1237	0	0	0.06311
rs140625413	C	0	NA	NA	NA	NA	NA	0.008197	NA	NA	NA	0	NA	0	0	NA	0
rs142444476	T	0	NA	NA	NA	NA	NA	0.008197	NA	NA	NA	0	NA	0	0	NA	0
rs545149163	A	0.005208	NA	NA	NA	NA	NA	0	NA	NA	NA	0	NA	0	0	NA	0
rs149798653	C	0.01562	NA	NA	NA	NA	NA	0	NA	NA	NA	0	NA	0	0	NA	0
rs527416684	A	0	NA	NA	NA	NA	NA	0	NA	NA	NA	0.01744	NA	0	0	NA	0
rs189067157	A	0	NA	NA	NA	NA	NA	0	NA	NA	NA	0	NA	0	0	NA	0

SNP	Allele	ACB	Afro	Aimaras	Ancash	Arequipa	Ashaninkas	ASW	Awajun	Ayacucho	Bambui	BEB	Candoshi	CDX	CEU	Chachapoyas	CHB
rs527489879	C	0	NA	NA	NA	NA	NA	0	NA	NA	NA	0	NA	0	0	NA	0
rs558824762	T	0.005208	NA	NA	NA	NA	NA	0.03279	NA	NA	NA	0	NA	0	0	NA	0
rs370927492	T	0	NA	NA	NA	NA	NA	0	NA	NA	NA	0.005814	NA	0	0	NA	0
rs1003030	G	0.1094	NA	NA	NA	NA	NA	0.09836	NA	NA	NA	0.2093	NA	0.2473	0.101	NA	0.3301
rs543908797	A	0	NA	NA	NA	NA	NA	0	NA	NA	NA	0	NA	0	0	NA	0
rs567103273	G	0	NA	NA	NA	NA	NA	0	NA	NA	NA	0	NA	0	0	NA	0
rs565820920	T	0	NA	NA	NA	NA	NA	0	NA	NA	NA	0	NA	0	0	NA	0
rs548463596	G	0.3385	NA	NA	NA	NA	NA	0.3934	NA	NA	NA	0.2965	NA	0.1774	0.2879	NA	0.2816
rs561570726	T	0	NA	NA	NA	NA	NA	0	NA	NA	NA	0	NA	0	0	NA	0
rs555911696	A	0	NA	NA	NA	NA	NA	0	NA	NA	NA	0	NA	0	0	NA	0
rs561179495	T	0	NA	NA	NA	NA	NA	0	NA	NA	NA	0	NA	0	0	NA	0.004854
rs181972300	A	0	NA	NA	NA	NA	NA	0	NA	NA	NA	0	NA	0	0	NA	0
rs138498737	A	0	NA	NA	NA	NA	NA	0	NA	NA	NA	0.005814	NA	0	0.01515	NA	0
rs565468881	T	0	NA	NA	NA	NA	NA	0	NA	NA	NA	0	NA	0	0	NA	0
rs186734573	T	0	NA	NA	NA	NA	NA	0	NA	NA	NA	0	NA	0	0	NA	0
rs146132480	A	0.005208	NA	NA	NA	NA	NA	0	NA	NA	NA	0	NA	0	0.03535	NA	0
rs559125514	C	0	NA	NA	NA	NA	NA	0	NA	NA	NA	0	NA	0	0	NA	0
rs527499027	T	0	NA	NA	NA	NA	NA	0	NA	NA	NA	0	NA	0	0	NA	0
rs573736906	A	0	NA	NA	NA	NA	NA	0	NA	NA	NA	0.01163	NA	0.005376	0	NA	0
rs573613070	G	0	NA	NA	NA	NA	NA	0	NA	NA	NA	0	NA	0	0	NA	0
rs542898542	T	0.005208	NA	NA	NA	NA	NA	0	NA	NA	NA	0	NA	0	0	NA	0
rs422471	C	0.4219	0.4468	0.7812	0.7639	0.6739	0.8286	0.4754	0.6739	0.6143	0.3212	0.3837	0.6875	0.6828	0.2929	0.6625	0.6699
rs181058683	A	0	NA	NA	NA	NA	NA	0	NA	NA	NA	0	NA	0	0	NA	0
rs149855493	C	0.02604	NA	NA	NA	NA	NA	0.03279	NA	NA	NA	0	NA	0	0	NA	0
rs191457025	T	0	NA	NA	NA	NA	NA	0	NA	NA	NA	0	NA	0.005376	0	NA	0.004854
rs375760	T	0.151	NA	NA	NA	NA	NA	0.1393	NA	NA	NA	0.2267	NA	0.3011	0.2273	NA	0.3252
rs148038688	T	0.02604	NA	NA	NA	NA	NA	0.03279	NA	NA	NA	0	NA	0	0	NA	0
rs145728087	A	0	NA	NA	NA	NA	NA	0	NA	NA	NA	0	NA	0	0	NA	0
rs569947342	T	0	NA	NA	NA	NA	NA	0	NA	NA	NA	0	NA	0	0	NA	0
rs562599408	G	0	NA	NA	NA	NA	NA	0	NA	NA	NA	0	NA	0	0	NA	0
rs146252393	A	0	NA	NA	NA	NA	NA	0	NA	NA	NA	0	NA	0	0	NA	0
rs73372182	A	0.1979	NA	NA	NA	NA	NA	0.1311	NA	NA	NA	0.005814	NA	0	0	NA	0
rs566012796	C	0.005208	NA	NA	NA	NA	NA	0	NA	NA	NA	0	NA	0	0.005051	NA	0
rs74423429	A	0	NA	NA	NA	NA	NA	0.008197	NA	NA	NA	0.01163	NA	0	0.02525	NA	0
rs193067129	A	0	NA	NA	NA	NA	NA	0	NA	NA	NA	0	NA	0	0	NA	0
rs572143040	C	0.01042	NA	NA	NA	NA	NA	0	NA	NA	NA	0	NA	0	0	NA	0
rs9984012	T	0.01562	NA	NA	NA	NA	NA	0.09016	NA	NA	NA	0.1163	NA	0.1129	0.1768	NA	0.06311
rs141898436	C	0.005208	NA	NA	NA	NA	NA	0.008197	NA	NA	NA	0	NA	0	0	NA	0
rs558272207	A	0	NA	NA	NA	NA	NA	0	NA	NA	NA	0	NA	0	0	NA	0
rs146681599	A	0	NA	NA	NA	NA	NA	0	NA	NA	NA	0	NA	0	0	NA	0
rs743542	A	0.125	0.1596	0.0625	0.1944	0.1087	0.1714	0.123	0.1304	0.2286	0.06979	0.2151	0.25	0.2097	0.0404	0.175	0.301
rs532511763	T	0	NA	NA	NA	NA	NA	0	NA	NA	NA	0	NA	0	0	NA	0
rs149424945	T	0	NA	NA	NA	NA	NA	0	NA	NA	NA	0	NA	0.005376	0	NA	0.004854
rs568605816	A	0	NA	NA	NA	NA	NA	0.008197	NA	NA	NA	0	NA	0	0	NA	0
rs184859933	T	0	NA	NA	NA	NA	NA	0	NA	NA	NA	0	NA	0	0	NA	0

SNP	Allele	ACB	Afro	Aimaras	Ancash	Arequipa	Ashaninkas	ASW	Awajun	Ayacucho	Bambui	BEB	Candoshi	CDX	CEU	Chachapoyas	CHB
rs530918384	GGTGA	0.005208	NA	NA	NA	NA	NA	0	NA	NA	NA	0	NA	0	0	NA	0
rs28369457	T	0	NA	NA	NA	NA	NA	0	NA	NA	NA	0	NA	0	0	NA	0
rs556192760	A	0	NA	NA	NA	NA	NA	0	NA	NA	NA	0	NA	0	0	NA	0
rs576447507	C	0	NA	NA	NA	NA	NA	0	NA	NA	NA	0	NA	0	0	NA	0
rs546512831	A	0	NA	NA	NA	NA	NA	0	NA	NA	NA	0	NA	0	0	NA	0.004854
rs543066660	A	0	NA	NA	NA	NA	NA	0	NA	NA	NA	0	NA	0	0	NA	0
rs543404937	T	0	NA	NA	NA	NA	NA	0	NA	NA	NA	0	NA	0	0	NA	0
rs180826598	C	0	NA	NA	NA	NA	NA	0	NA	NA	NA	0	NA	0	0	NA	0
rs368878191	T	0	NA	NA	NA	NA	NA	0	NA	NA	NA	0	NA	0	0	NA	0
rs369619813	G	0	NA	NA	NA	NA	NA	0	NA	NA	NA	0	NA	0	0	NA	0
rs112213575	A	0.005208	NA	NA	NA	NA	NA	0.008197	NA	NA	NA	0	NA	0	0	NA	0
rs548725701	A	0	NA	NA	NA	NA	NA	0	NA	NA	NA	0	NA	0	0.005051	NA	0
rs569959837	A	0	NA	NA	NA	NA	NA	0	NA	NA	NA	0	NA	0	0	NA	0
rs554986094	A	0	NA	NA	NA	NA	NA	0	NA	NA	NA	0	NA	0	0	NA	0
rs456142	T	0.3646	NA	NA	NA	NA	NA	0.3443	NA	NA	0.1887	0.3779	NA	0.7312	0.1616	NA	0.534
rs73357644	T	0.224	NA	NA	NA	NA	NA	0.1803	NA	NA	NA	0.005814	NA	0	0	NA	0
rs576259678	C	0	NA	NA	NA	NA	NA	0	NA	NA	NA	0.005814	NA	0	0	NA	0
rs140141551	A	0	NA	NA	NA	NA	NA	0.008197	NA	NA	NA	0	NA	0	0	NA	0
rs146564124	T	0	NA	NA	NA	NA	NA	0.008197	NA	NA	NA	0	NA	0	0.0101	NA	0
rs558715322	T	0	NA	NA	NA	NA	NA	0	NA	NA	NA	0	NA	0	0	NA	0
rs116020930	A	0	NA	NA	NA	NA	NA	0	NA	NA	NA	0	NA	0	0	NA	0
rs539333191	T	0	NA	NA	NA	NA	NA	0	NA	NA	NA	0	NA	0	0	NA	0
rs192259532	T	0	NA	NA	NA	NA	NA	0	NA	NA	NA	0	NA	0	0	NA	0
rs531920590	T	0	NA	NA	NA	NA	NA	0	NA	NA	NA	0	NA	0	0	NA	0
rs531300154	C	0	NA	NA	NA	NA	NA	0	NA	NA	NA	0	NA	0	0	NA	0
rs147945120	T	0	NA	NA	NA	NA	NA	0	NA	NA	NA	0	NA	0	0.005051	NA	0
rs147934552	A	0	NA	NA	NA	NA	NA	0	NA	NA	NA	0	NA	0	0	NA	0
rs112980967	A	0.01042	NA	NA	NA	NA	NA	0.01639	NA	NA	NA	0	NA	0	0	NA	0
rs149601802	T	0	NA	NA	NA	NA	NA	0	NA	NA	NA	0	NA	0	0	NA	0.004854
rs3761373	T	0.1146	NA	NA	NA	NA	NA	0.09836	NA	NA	0.07719	0.2093	NA	0.2473	0.101	NA	0.3252
rs545333396	C	0	NA	NA	NA	NA	NA	0	NA	NA	NA	0	NA	0	0	NA	0
rs574683527	A	0	NA	NA	NA	NA	NA	0	NA	NA	NA	0	NA	0	0	NA	0
rs189431131	C	0	NA	NA	NA	NA	NA	0	NA	NA	NA	0	NA	0	0	NA	0
rs183147330	A	0	NA	NA	NA	NA	NA	0	NA	NA	NA	0	NA	0	0	NA	0
rs417443	A	0.01562	NA	NA	NA	NA	NA	0.02459	NA	NA	NA	0.0814	NA	0.4892	0.0303	NA	0.3155
rs564403172	T	0	NA	NA	NA	NA	NA	0	NA	NA	NA	0	NA	0	0	NA	0
rs8134216	T	0.4688	0.4574	0.1562	0.1528	0.2609	0.1143	0.5	0.2391	0.1571	0.7247	0.593	0.125	0.2473	0.7475	0.2125	0.3058
rs191441931	T	0	NA	NA	NA	NA	NA	0.008197	NA	NA	NA	0	NA	0	0	NA	0
rs567188556	T	0	NA	NA	NA	NA	NA	0.008197	NA	NA	NA	0	NA	0	0	NA	0
rs187662898	T	0	NA	NA	NA	NA	NA	0	NA	NA	NA	0	NA	0	0	NA	0
rs114911304	A	0.03646	NA	NA	NA	NA	NA	0.008197	NA	NA	NA	0	NA	0	0	NA	0
rs558858943	C	0.005208	NA	NA	NA	NA	NA	0	NA	NA	NA	0	NA	0	0	NA	0
rs541396507	C	0	NA	NA	NA	NA	NA	0	NA	NA	NA	0	NA	0	0	NA	0.004854
rs565142599	T	0.005208	NA	NA	NA	NA	NA	0	NA	NA	NA	0	NA	0	0	NA	0
rs146605032	A	0	NA	NA	NA	NA	NA	0	NA	NA	NA	0	NA	0	0	NA	0

SNP	Allele	ACB	Afro	Aimaras	Ancash	Arequipa	Ashaninkas	ASW	Awajun	Ayacucho	Bambui	BEB	Candoshi	CDX	CEU	Chachapoyas	CHB
rs553673225	G	0	NA	NA	NA	NA	NA	0	NA	NA	NA	0	NA	0.005376	0	NA	0
rs113034290	T	0.005208	NA	NA	NA	NA	NA	0	NA	NA	NA	0	NA	0	0	NA	0
rs150554820	T	NA	NA	NA	NA	NA	NA	NA	NA	NA	NA	NA	NA	NA	NA	NA	NA
rs544880781	A	0.005208	NA	NA	NA	NA	NA	0	NA	NA	NA	0	NA	0	0	NA	0
rs6517669	A	0.3958	0.6489	1	0.8889	0.8696	1	0.377	0.8261	0.94286	0.724	0.6919	0.8438	0.8065	0.7424	0.9	0.6456
rs550238535	A	0	NA	NA	NA	NA	NA	0	NA	NA	NA	0	NA	0	0	NA	0
rs455922	T	0.005208	NA	NA	NA	NA	NA	0.02459	NA	NA	NA	0.0814	NA	0.4032	0.02525	NA	0.267
rs374203194	A	0	NA	NA	NA	NA	NA	0	NA	NA	NA	0	NA	0	0.0101	NA	0
rs111671182	G	0.01042	NA	NA	NA	NA	NA	0	NA	NA	NA	0	NA	0	0	NA	0
rs181044447	A	0	NA	NA	NA	NA	NA	0	NA	NA	NA	0	NA	0	0.005051	NA	0
rs573737830	A	0	NA	NA	NA	NA	NA	0	NA	NA	NA	0	NA	0	0	NA	0
rs181778800	A	0	NA	NA	NA	NA	NA	0	NA	NA	NA	0	NA	0	0	NA	0
rs577217013	C	0	NA	NA	NA	NA	NA	0	NA	NA	NA	0	NA	0	0	NA	0
rs563948676	A	0	NA	NA	NA	NA	NA	0	NA	NA	NA	0	NA	0	0	NA	0
rs141888586	T	0.09896	NA	NA	NA	NA	NA	0.06557	NA	NA	NA	0	NA	0	0	NA	0
rs146797606	C	0.02604	NA	NA	NA	NA	NA	0.03279	NA	NA	NA	0	NA	0	0	NA	0
rs544037190	C	0	NA	NA	NA	NA	NA	0	NA	NA	NA	0	NA	0	0	NA	0
rs535041396	T	0	NA	NA	NA	NA	NA	0	NA	NA	NA	0	NA	0	0	NA	0
rs534591508	T	0	NA	NA	NA	NA	NA	0	NA	NA	NA	0	NA	0	0	NA	0
rs547758146	A	0	NA	NA	NA	NA	NA	0	NA	NA	NA	0	NA	0	0	NA	0
rs529424146	A	0	NA	NA	NA	NA	NA	0	NA	NA	NA	0	NA	0	0	NA	0
rs563946938	T	0	NA	NA	NA	NA	NA	0	NA	NA	NA	0	NA	0	0	NA	0
rs545056304	A	0	NA	NA	NA	NA	NA	0	NA	NA	NA	0	NA	0	0	NA	0
rs12481984	C	0.2865	NA	NA	NA	NA	NA	0.2705	NA	NA	NA	0.2151	NA	0.03763	0.399	NA	0.009709
rs538226704	T	0	NA	NA	NA	NA	NA	0.01639	NA	NA	NA	0	NA	0	0	NA	0
rs548678297	G	0	NA	NA	NA	NA	NA	0	NA	NA	NA	0	NA	0	0	NA	0
rs149676870	T	0	NA	NA	NA	NA	NA	0	NA	NA	NA	0	NA	0	0	NA	0
rs78217567	C	0	NA	NA	NA	NA	NA	0.04098	NA	NA	NA	0.005814	NA	0	0.06061	NA	0
rs558791980	A	0	NA	NA	NA	NA	NA	0	NA	NA	NA	0	NA	0	0.005051	NA	0
rs34256269	A	0.01562	NA	NA	NA	NA	NA	0.08197	NA	NA	NA	0.1221	NA	0.1075	0.1768	NA	0.06311
rs74749793	T	0.1094	0.07447	0	0	0	0	0.09836	0	0	0.07772	0.2093	0	0.2258	0.101	0.0125	0.3204
rs193253965	C	0	NA	NA	NA	NA	NA	0	NA	NA	NA	0	NA	0	0.005051	NA	0
rs373622147	C	0	NA	NA	NA	NA	NA	0	NA	NA	NA	0.01163	NA	0	0	NA	0
rs559691670	A	0	NA	NA	NA	NA	NA	0	NA	NA	NA	0	NA	0.005376	0	NA	0
rs534317621	A	0	NA	NA	NA	NA	NA	0	NA	NA	NA	0	NA	0	0	NA	0
rs539981354	G	0	NA	NA	NA	NA	NA	0	NA	NA	NA	0	NA	0	0	NA	0
rs115266855	G	0.02604	NA	NA	NA	NA	NA	0.01639	NA	NA	NA	0	NA	0	0	NA	0
rs149109132	A	0.03125	NA	NA	NA	NA	NA	0.03279	NA	NA	NA	0	NA	0	0	NA	0
rs557656729	A	0	NA	NA	NA	NA	NA	0	NA	NA	NA	0	NA	0	0	NA	0
rs57161767	A	0.03646	NA	NA	NA	NA	NA	0.04098	NA	NA	NA	0	NA	0	0	NA	0
rs574708698	T	0.005208	NA	NA	NA	NA	NA	0	NA	NA	NA	0	NA	0	0	NA	0
rs4303795	G	0.3021	NA	NA	NA	NA	NA	0.3033	NA	NA	NA	0.2326	NA	0.03763	0.4091	NA	0.009709
rs145662876	T	0	NA	NA	NA	NA	NA	0	NA	NA	NA	0.005814	NA	0	0	NA	0.009709
rs566146941	A	0	NA	NA	NA	NA	NA	0	NA	NA	NA	0.005814	NA	0	0	NA	0
rs563595766	T	0	NA	NA	NA	NA	NA	0	NA	NA	NA	0	NA	0	0	NA	0

SNP	Allele	ACB	Afro	Aimaras	Ancash	Arequipa	Ashaninkas	ASW	Awajun	Ayacucho	Bambui	BEB	Candoshi	CDX	CEU	Chachapoyas	CHB
rs572507505	T	0	NA	NA	NA	NA	NA	0	NA	NA	NA	0	NA	0	0	NA	0
rs557606231	G	0.005208	NA	NA	NA	NA	NA	0.008197	NA	NA	NA	0	NA	0	0	NA	0
rs11911394	T	0.3958	0.6489	1	0.8889	0.8696	1	0.377	0.8261	0.94286	0.7242	0.6919	0.8438	0.8065	0.7374	0.9	0.6456
rs76973757	A	0.03646	NA	NA	NA	NA	NA	0.05738	NA	NA	NA	0	NA	0	0	NA	0
rs573484758	G	0	NA	NA	NA	NA	NA	0	NA	NA	NA	0.005814	NA	0.005376	0	NA	0
rs530471976	T	0	NA	NA	NA	NA	NA	0	NA	NA	NA	0	NA	0	0.005051	NA	0
rs372286621	A	0	NA	NA	NA	NA	NA	0	NA	NA	NA	0	NA	0	0	NA	0
rs4818239	C	0.276	NA	NA	NA	NA	NA	0.2705	NA	NA	NA	0.407	NA	0.01613	0.5051	NA	0.004854
rs186484871	C	0	NA	NA	NA	NA	NA	0	NA	NA	NA	0	NA	0	0	NA	0
rs542575868	A	0	NA	NA	NA	NA	NA	0	NA	NA	NA	0	NA	0	0	NA	0
rs111572592	A	0	NA	NA	NA	NA	NA	0.01639	NA	NA	NA	0.005814	NA	0	0.0202	NA	0
rs144576889	G	0.02604	NA	NA	NA	NA	NA	0.03279	NA	NA	NA	0	NA	0	0	NA	0
rs572265353	A	0	NA	NA	NA	NA	NA	0	NA	NA	NA	0	NA	0	0	NA	0
rs191368876	C	0	NA	NA	NA	NA	NA	0	NA	NA	NA	0	NA	0.01075	0	NA	0
rs535472251	CAAAAAAAAAA	0.276	NA	NA	NA	NA	NA	0.2705	NA	NA	NA	0.2791	NA	0.3118	0.3636	NA	0.3204
rs141788162	A	0	NA	NA	NA	NA	NA	0	NA	NA	NA	0	NA	0	0	NA	0
rs56695953	A	0.05208	NA	NA	NA	NA	NA	0.08197	NA	NA	NA	0.06395	NA	0.01613	0.2172	NA	0.06796
rs548680244	T	0	NA	NA	NA	NA	NA	0	NA	NA	NA	0	NA	0	0	NA	0
rs430915	A	0.1875	0.2979	0.9375	0.7639	0.6739	0.94286	0.2049	0.6957	0.6571	0.4573	0.4651	0.5938	0.5161	0.5101	0.7375	0.3398
rs564795317	A	0	NA	NA	NA	NA	NA	0	NA	NA	NA	0	NA	0	0	NA	0
rs577731955	C	0	NA	NA	NA	NA	NA	0	NA	NA	NA	0	NA	0	0	NA	0
rs369342013	A	0	NA	NA	NA	NA	NA	0	NA	NA	NA	0	NA	0	0	NA	0
rs542402063	A	0	NA	NA	NA	NA	NA	0	NA	NA	NA	0	NA	0	0	NA	0
rs556381293	C	0.01042	NA	NA	NA	NA	NA	0	NA	NA	NA	0	NA	0	0	NA	0
rs534924497	T	0	NA	NA	NA	NA	NA	0	NA	NA	NA	0	NA	0	0	NA	0
rs554462605	A	0	NA	NA	NA	NA	NA	0	NA	NA	NA	0	NA	0	0	NA	0.004854
rs80027429	A	0	NA	NA	NA	NA	NA	0	NA	NA	NA	0.01163	NA	0	0.005051	NA	0
rs561192574	T	0	NA	NA	NA	NA	NA	0	NA	NA	NA	0	NA	0.005376	0	NA	0
rs455281	G	0.2188	NA	NA	NA	NA	NA	0.1885	NA	NA	NA	0.2907	NA	0.7043	0.0303	NA	0.466
rs9975782	A	0	NA	NA	NA	NA	NA	0	NA	NA	NA	0	NA	0	0	NA	0
rs569362310	G	0	NA	NA	NA	NA	NA	0	NA	NA	NA	0	NA	0	0	NA	0
rs536278410	C	0	NA	NA	NA	NA	NA	0	NA	NA	NA	0.005814	NA	0.005376	0	NA	0
rs415918	C	0.4219	NA	NA	NA	NA	NA	0.459	NA	NA	NA	0.3837	NA	0.6828	0.2828	NA	0.6699
rs554890599	A	0	NA	NA	NA	NA	NA	0	NA	NA	NA	0	NA	0	0	NA	0
rs530721239	G	0	NA	NA	NA	NA	NA	0	NA	NA	NA	0	NA	0	0.005051	NA	0
rs552589240	G	0	NA	NA	NA	NA	NA	0	NA	NA	NA	0	NA	0	0	NA	0
rs545504807	C	0	NA	NA	NA	NA	NA	0	NA	NA	NA	0	NA	0	0	NA	0
rs28403625	A	0	NA	NA	NA	NA	NA	0	NA	NA	NA	0	NA	0	0	NA	0.004854
rs532159311	C	0	NA	NA	NA	NA	NA	0	NA	NA	NA	0	NA	0	0	NA	0
rs543578752	G	0	NA	NA	NA	NA	NA	0	NA	NA	NA	0	NA	0	0	NA	0
rs576215861	A	0	NA	NA	NA	NA	NA	0	NA	NA	NA	0	NA	0	0	NA	0
rs199824558	A	0	NA	NA	NA	NA	NA	0	NA	NA	NA	0	NA	0	0	NA	0
rs562000328	C	0	NA	NA	NA	NA	NA	0	NA	NA	NA	0	NA	0	0	NA	0
rs559698510	T	0	NA	NA	NA	NA	NA	0	NA	NA	NA	0	NA	0	0	NA	0
rs75168613	A	0.09896	NA	NA	NA	NA	NA	0.09016	NA	NA	NA	0.005814	NA	0.005376	0	NA	0

SNP	Allele	ACB	Afro	Aimaras	Ancash	Arequipa	Ashaninkas	ASW	Awajun	Ayacucho	Bambui	BEB	Candoshi	CDX	CEU	Chachapoyas	CHB
rs543344542	T	0	NA	NA	NA	NA	NA	0	NA	NA	NA	0	NA	0	0	NA	0
rs570889046	A	0.03125	NA	NA	NA	NA	NA	0.008197	NA	NA	NA	0	NA	0	0	NA	0
rs548267325	A	0	NA	NA	NA	NA	NA	0	NA	NA	NA	0	NA	0	0	NA	0
rs35899679	A	0.1667	NA	NA	NA	NA	NA	0.1803	NA	NA	NA	0.3721	NA	0.01075	0.4798	NA	0.004854
rs561575371	A	0	NA	NA	NA	NA	NA	0	NA	NA	NA	0	NA	0	0	NA	0
rs577554253	A	0	NA	NA	NA	NA	NA	0	NA	NA	NA	0	NA	0	0	NA	0
rs535407146	A	0	NA	NA	NA	NA	NA	0	NA	NA	NA	0	NA	0	0	NA	0
rs149024920	G	0.01042	NA	NA	NA	NA	NA	0	NA	NA	NA	0	NA	0	0	NA	0
rs112209215	A	0.005208	NA	NA	NA	NA	NA	0.008197	NA	NA	NA	0	NA	0	0	NA	0
rs183405398	G	0	NA	NA	NA	NA	NA	0.008197	NA	NA	NA	0	NA	0	0.005051	NA	0
rs374510753	T	0	NA	NA	NA	NA	NA	0	NA	NA	NA	0	NA	0	0	NA	0
rs75200570	C	0.1094	NA	NA	NA	NA	NA	0.05738	NA	NA	NA	0.02326	NA	0.04301	0	NA	0.07282
rs149275684	T	0	NA	NA	NA	NA	NA	0	NA	NA	NA	0	NA	0	0	NA	0
rs547544037	A	0	NA	NA	NA	NA	NA	0	NA	NA	NA	0	NA	0	0	NA	0
rs553755767	A	0	NA	NA	NA	NA	NA	0	NA	NA	NA	0	NA	0	0	NA	0
rs76855393	T	0	NA	NA	NA	NA	NA	0	NA	NA	NA	0	NA	0	0	NA	0.004854
rs118028230	C	NA	NA	NA	NA	NA	NA	NA	NA	NA	NA	NA	NA	NA	NA	NA	NA
rs184764113	A	0	NA	NA	NA	NA	NA	0	NA	NA	NA	0	NA	0	0	NA	0
rs541351488	A	0	NA	NA	NA	NA	NA	0	NA	NA	NA	0	NA	0	0	NA	0
rs185078457	T	0	NA	NA	NA	NA	NA	0	NA	NA	NA	0	NA	0	0	NA	0.004854
rs542961770	T	0	NA	NA	NA	NA	NA	0	NA	NA	NA	0	NA	0	0	NA	0
rs562131885	A	0.005208	NA	NA	NA	NA	NA	0.03279	NA	NA	NA	0	NA	0	0	NA	0
rs536399456	A	0	NA	NA	NA	NA	NA	0	NA	NA	NA	0	NA	0	0	NA	0
rs547523412	A	0	NA	NA	NA	NA	NA	0	NA	NA	NA	0	NA	0	0	NA	0.004854
rs534034788	A	0	NA	NA	NA	NA	NA	0	NA	NA	NA	0.005814	NA	0	0	NA	0
rs544784789	G	0	NA	NA	NA	NA	NA	0	NA	NA	NA	0.005814	NA	0	0	NA	0
rs540987630	T	0	NA	NA	NA	NA	NA	0	NA	NA	NA	0	NA	0	0	NA	0
rs141478137	G	0	NA	NA	NA	NA	NA	0	NA	NA	NA	0	NA	0.005376	0	NA	0.009709
rs5844077	G	0.1406	NA	NA	NA	NA	NA	0.1721	NA	NA	NA	0.2384	NA	0.3441	0.2677	NA	0.2961
rs551681725	G	0	NA	NA	NA	NA	NA	0	NA	NA	NA	0	NA	0	0	NA	0
rs548592256	C	0	NA	NA	NA	NA	NA	0	NA	NA	NA	0	NA	0	0	NA	0
rs568689321	A	0	NA	NA	NA	NA	NA	0	NA	NA	NA	0.005814	NA	0	0	NA	0
rs555302570	A	0	NA	NA	NA	NA	NA	0	NA	NA	NA	0	NA	0	0	NA	0
rs537212420	G	0	NA	NA	NA	NA	NA	0	NA	NA	NA	0	NA	0	0	NA	0
rs547186777	C	0	NA	NA	NA	NA	NA	0	NA	NA	NA	0	NA	0	0	NA	0
rs532627816	A	0	NA	NA	NA	NA	NA	0	NA	NA	NA	0	NA	0	0	NA	0
rs150314077	A	0.04167	NA	NA	NA	NA	NA	0.01639	NA	NA	NA	0	NA	0	0	NA	0
rs544983081	A	0	NA	NA	NA	NA	NA	0	NA	NA	NA	0.005814	NA	0	0	NA	0
rs140121827	T	0	NA	NA	NA	NA	NA	0	NA	NA	NA	0	NA	0.005376	0	NA	0
rs74605993	T	0.005208	NA	NA	NA	NA	NA	0.01639	NA	NA	NA	0	NA	0	0	NA	0
rs7279603	C	0.4479	0.3298	0	0	0.1522	0	0.3525	0.04348	0	0.3773	0.2616	0.0625	0.2419	0.298	0.0375	0.2961
rs535611212	G	0	NA	NA	NA	NA	NA	0	NA	NA	NA	0	NA	0	0	NA	0
rs180792570	T	0	NA	NA	NA	NA	NA	0.008197	NA	NA	NA	0	NA	0	0	NA	0
rs180784757	A	0.02604	NA	NA	NA	NA	NA	0.03279	NA	NA	NA	0	NA	0	0	NA	0
rs532756922	A	0	NA	NA	NA	NA	NA	0	NA	NA	NA	0	NA	0	0	NA	0

SNP	Allele	ACB	Afro	Aimaras	Ancash	Arequipa	Ashaninkas	ASW	Awajun	Ayacucho	Bambui	BEB	Candoshi	CDX	CEU	Chachapoyas	CHB
rs138651919	A	0	NA	NA	NA	NA	NA	0	NA	NA	NA	0	NA	0	0	NA	0
rs549212787	G	0	NA	NA	NA	NA	NA	0	NA	NA	NA	0.005814	NA	0	0	NA	0
rs538863731	C	0	NA	NA	NA	NA	NA	0	NA	NA	NA	0	NA	0	0	NA	0
rs538803792	T	0	NA	NA	NA	NA	NA	0	NA	NA	NA	0	NA	0	0	NA	0
rs574890845	A	0.005208	NA	NA	NA	NA	NA	0	NA	NA	NA	0	NA	0	0	NA	0
rs546165791	T	0	NA	NA	NA	NA	NA	0	NA	NA	NA	0	NA	0	0	NA	0
rs554530868	G	0	NA	NA	NA	NA	NA	0.008197	NA	NA	NA	0.01744	NA	0	0.04545	NA	0
rs79512425	C	0	NA	NA	NA	NA	NA	0	NA	NA	NA	0	NA	0	0	NA	0
rs542395007	T	0	NA	NA	NA	NA	NA	0	NA	NA	NA	0	NA	0	0.005051	NA	0
rs556249043	A	0	NA	NA	NA	NA	NA	0	NA	NA	NA	0.005814	NA	0	0	NA	0
rs529550811	A	0.01562	NA	NA	NA	NA	NA	0.008197	NA	NA	NA	0	NA	0	0	NA	0
rs533931558	A	0	NA	NA	NA	NA	NA	0	NA	NA	NA	0	NA	0	0	NA	0
rs565902164	AACC	0	NA	NA	NA	NA	NA	0	NA	NA	NA	0.02326	NA	0	0	NA	0
rs530084541	G	0.005208	NA	NA	NA	NA	NA	0	NA	NA	NA	0	NA	0	0	NA	0
rs549593213	T	0	NA	NA	NA	NA	NA	0	NA	NA	NA	0.005814	NA	0	0	NA	0
rs189570078	C	0	NA	NA	NA	NA	NA	0	NA	NA	NA	0	NA	0	0	NA	0
rs190682490	T	0	NA	NA	NA	NA	NA	0	NA	NA	NA	0	NA	0	0.005051	NA	0
rs577570888	T	0	NA	NA	NA	NA	NA	0	NA	NA	NA	0	NA	0	0	NA	0
rs376403654	A	0	NA	NA	NA	NA	NA	0	NA	NA	NA	0	NA	0	0	NA	0
rs576016640	T	0.005208	NA	NA	NA	NA	NA	0	NA	NA	NA	0	NA	0	0.005051	NA	0
rs567552202	C	0	NA	NA	NA	NA	NA	0	NA	NA	NA	0	NA	0	0	NA	0
rs140793040	A	0	NA	NA	NA	NA	NA	0	NA	NA	NA	0	NA	0	0.005051	NA	0
rs146445857	TGCC	0	NA	NA	NA	NA	NA	0	NA	NA	NA	0	NA	0	0	NA	0.05825
rs547401659	T	0	NA	NA	NA	NA	NA	0	NA	NA	NA	0	NA	0	0	NA	0
rs571396442	T	0	NA	NA	NA	NA	NA	0	NA	NA	NA	0	NA	0	0	NA	0
rs185406661	G	0	NA	NA	NA	NA	NA	0	NA	NA	NA	0	NA	0	0	NA	0
rs2070789	T	0.3958	NA	NA	NA	NA	NA	0.4344	NA	NA	NA	0.3256	NA	0.3548	0.2121	NA	0.4272
rs7277080	T	0.2969	NA	NA	NA	NA	NA	0.2869	NA	NA	NA	0.2151	NA	0.03763	0.3687	NA	0.009709
rs183385443	A	0	NA	NA	NA	NA	NA	0	NA	NA	NA	0	NA	0	0	NA	0
rs181592444	T	0	NA	NA	NA	NA	NA	0	NA	NA	NA	0	NA	0.01075	0	NA	0.01456
rs189832305	T	0	NA	NA	NA	NA	NA	0	NA	NA	NA	0	NA	0.01613	0	NA	0.009709
rs734056	A	0.2448	0.1809	0.1562	0.1528	0.1087	0.1143	0.2787	0.1957	0.1286	0.4185	0.407	0.0625	0.01613	0.5051	0.175	0.004854
rs561819256	G	0	NA	NA	NA	NA	NA	0	NA	NA	NA	0	NA	0	0	NA	0
rs2070786	C	0.3177	0.2826	0	0	0.1522	0	0.2787	0.04348	0	0.3608	0.2558	0.0625	0.2419	0.298	0.0375	0.2961
rs541155775	C	0	NA	NA	NA	NA	NA	0	NA	NA	NA	0	NA	0	0	NA	0
rs544315388	A	0	NA	NA	NA	NA	NA	0	NA	NA	NA	0	NA	0	0	NA	0
rs191763249	G	0	NA	NA	NA	NA	NA	0	NA	NA	NA	0	NA	0	0	NA	0
rs137962614	T	0	NA	NA	NA	NA	NA	0	NA	NA	NA	0	NA	0	0.005051	NA	0
rs464431	A	0.01562	NA	NA	NA	NA	NA	0.02459	NA	NA	NA	0.08721	NA	0.4032	0.02525	NA	0.267
rs147054075	A	0	NA	NA	NA	NA	NA	0.01639	NA	NA	NA	0	NA	0	0	NA	0
rs190516010	A	0	NA	NA	NA	NA	NA	0.008197	NA	NA	NA	0	NA	0	0	NA	0
rs542265380	T	0	NA	NA	NA	NA	NA	0	NA	NA	NA	0	NA	0	0	NA	0
rs188263047	A	0	NA	NA	NA	NA	NA	0	NA	NA	NA	0	NA	0	0	NA	0
rs143148203	G	0	NA	NA	NA	NA	NA	0.01639	NA	NA	NA	0	NA	0	0	NA	0
rs557853844	A	0	NA	NA	NA	NA	NA	0	NA	NA	NA	0	NA	0	0	NA	0

SNP	Allele	ACB	Afro	Aimaras	Ancash	Arequipa	Ashaninkas	ASW	Awajun	Ayacucho	Bambui	BEB	Candoshi	CDX	CEU	Chachapoyas	CHB
rs571275590	G	0	NA	NA	NA	NA	NA	0	NA	NA	NA	0.005814	NA	0	0	NA	0
rs562173027	C	0	NA	NA	NA	NA	NA	0	NA	NA	NA	0	NA	0	0	NA	0
rs530253044	T	0	NA	NA	NA	NA	NA	0	NA	NA	NA	0	NA	0	0	NA	0
rs532694622	C	0	NA	NA	NA	NA	NA	0	NA	NA	NA	0.005814	NA	0	0	NA	0
rs75756279	T	0	NA	NA	NA	NA	NA	0	NA	NA	NA	0.01163	NA	0	0.005051	NA	0
rs570835062	T	0	NA	NA	NA	NA	NA	0	NA	NA	NA	0	NA	0	0	NA	0
rs530942388	G	0	NA	NA	NA	NA	NA	0	NA	NA	NA	0	NA	0	0	NA	0
rs575313753	G	0	NA	NA	NA	NA	NA	0.008197	NA	NA	NA	0	NA	0	0	NA	0
rs537370123	T	0	NA	NA	NA	NA	NA	0	NA	NA	NA	0	NA	0	0	NA	0
rs185946013	A	0	NA	NA	NA	NA	NA	0	NA	NA	NA	0	NA	0	0	NA	0
rs142518005	T	0	NA	NA	NA	NA	NA	0.008197	NA	NA	NA	0	NA	0	0	NA	0
rs558104085	A	0	NA	NA	NA	NA	NA	0	NA	NA	NA	0	NA	0	0	NA	0
rs144800717	T	0	NA	NA	NA	NA	NA	0	NA	NA	NA	0	NA	0	0	NA	0
rs57474639	T	0.08854	0.08511	0	0.1111	0.04348	0	0.09836	0.1304	0.02857	0.06727	0.08721	0.09375	0.02151	0.1313	0.075	0.06311
rs538139969	T	0	NA	NA	NA	NA	NA	0	NA	NA	NA	0	NA	0	0	NA	0
rs567302726	A	0	NA	NA	NA	NA	NA	0	NA	NA	NA	0.02326	NA	0	0	NA	0
rs187052796	T	0.02604	NA	NA	NA	NA	NA	0.03279	NA	NA	NA	0	NA	0	0	NA	0
rs551164492	G	0.01562	NA	NA	NA	NA	NA	0	NA	NA	NA	0.005814	NA	0	0.005051	NA	0
rs143562195	A	0.02083	NA	NA	NA	NA	NA	0.02459	NA	NA	NA	0	NA	0	0	NA	0
rs11088550	A	0.005208	NA	NA	NA	NA	NA	0.04098	NA	NA	NA	0.01163	NA	0	0.1061	NA	0
rs151338439	A	0	NA	NA	NA	NA	NA	0	NA	NA	NA	0	NA	0	0.005051	NA	0
rs185726773	A	0.005208	NA	NA	NA	NA	NA	0	NA	NA	NA	0	NA	0	0	NA	0
rs531410451	A	0	NA	NA	NA	NA	NA	0	NA	NA	NA	0	NA	0	0	NA	0
rs2257202	C	0.1198	NA	NA	NA	NA	NA	0.123	NA	NA	NA	0.2151	NA	0.2527	0.2222	NA	0.3155
rs117827300	A	0	NA	NA	NA	NA	NA	0	NA	NA	NA	0	NA	0	0	NA	0
rs187208295	C	0	NA	NA	NA	NA	NA	0	NA	NA	NA	0	NA	0.01075	0	NA	0.01456
rs61735793	A	0	0	0	0	0.02174	0	0	0	0	0.00694	0.005814	0	0	0.0202	0	0
rs143712818	T	0.02604	NA	NA	NA	NA	NA	0.03279	NA	NA	NA	0	NA	0	0	NA	0
rs542575245	T	0	NA	NA	NA	NA	NA	0	NA	NA	NA	0	NA	0	0	NA	0
rs551797163	C	0	NA	NA	NA	NA	NA	0	NA	NA	NA	0	NA	0	0	NA	0
rs571050655	C	0	NA	NA	NA	NA	NA	0	NA	NA	NA	0	NA	0	0	NA	0
rs34624090	AC	0.1406	NA	NA	NA	NA	NA	0.1885	NA	NA	NA	0.3256	NA	0.005376	0.4646	NA	0.004854
rs147099383	C	0.05208	NA	NA	NA	NA	NA	0.02459	NA	NA	NA	0.1395	NA	0.1505	0.0404	NA	0.1068
rs58146697	C	0.2552	0.1702	0	0	0.02174	0	0.1557	0	0	0.05791	0.03488	0	0.05376	0.005051	0	0.05825
rs139700775	C	0.005208	NA	NA	NA	NA	NA	0	NA	NA	NA	0	NA	0	0	NA	0
rs560183935	C	0.005208	NA	NA	NA	NA	NA	0.008197	NA	NA	NA	0	NA	0	0	NA	0
rs182330267	A	0.01042	NA	NA	NA	NA	NA	0	NA	NA	NA	0	NA	0	0	NA	0
rs565879862	C	0	NA	NA	NA	NA	NA	0	NA	NA	NA	0	NA	0.005376	0	NA	0.004854
rs188500480	T	0	NA	NA	NA	NA	NA	0	NA	NA	NA	0	NA	0	0	NA	0.004854
rs533556786	G	0	NA	NA	NA	NA	NA	0	NA	NA	NA	0.01163	NA	0	0	NA	0
rs551106657	T	0	NA	NA	NA	NA	NA	0	NA	NA	NA	0.005814	NA	0	0	NA	0
rs116606027	A	0.02604	NA	NA	NA	NA	NA	0.03279	NA	NA	NA	0	NA	0	0	NA	0
rs573715927	A	0	NA	NA	NA	NA	NA	0	NA	NA	NA	0	NA	0	0	NA	0
rs145026350	T	0	NA	NA	NA	NA	NA	0	NA	NA	NA	0	NA	0	0	NA	0
rs915823	C	0.1562	0.09574	0	0.1667	0.06522	0	0.1803	0.1304	0.02857	0.125	0.1512	0.09375	0.1667	0.1919	0.0875	0.3058

SNP	Allele	ACB	Afro	Aimaras	Ancash	Arequipa	Ashaninkas	ASW	Awajun	Ayacucho	Bambui	BEB	Candoshi	CDX	CEU	Chachapoyas	CHB
rs188495261	A	0	NA	NA	NA	NA	NA	0	NA	NA	NA	0	NA	0	0	NA	0
rs563800355	T	0	NA	NA	NA	NA	NA	0	NA	NA	NA	0	NA	0	0	NA	0
rs143060022	A	0	NA	NA	NA	NA	NA	0	NA	NA	NA	0	NA	0	0	NA	0
rs527811791	C	0	NA	NA	NA	NA	NA	0	NA	NA	NA	0.005814	NA	0	0	NA	0
rs554692214	C	0	NA	NA	NA	NA	NA	0	NA	NA	NA	0	NA	0	0	NA	0
rs8129582	A	0	NA	NA	NA	NA	NA	0	NA	NA	NA	0	NA	0	0	NA	0
rs532513568	C	0	NA	NA	NA	NA	NA	0	NA	NA	NA	0	NA	0	0	NA	0
rs537357445	C	0.005208	NA	NA	NA	NA	NA	0	NA	NA	NA	0	NA	0	0	NA	0
rs538591883	C	0	NA	NA	NA	NA	NA	0	NA	NA	NA	0	NA	0	0	NA	0
rs535599066	T	0	NA	NA	NA	NA	NA	0	NA	NA	NA	0.005814	NA	0	0	NA	0
rs7275220	G	0.4792	NA	NA	NA	NA	NA	0.4426	NA	NA	0.361	0.3488	NA	0.8172	0.2374	NA	0.7136
rs199636550	G	0	NA	NA	NA	NA	NA	0	NA	NA	NA	0	NA	0	0.005051	NA	0
rs117696554	A	0	NA	NA	NA	NA	NA	0	NA	NA	NA	0	NA	0	0.03535	NA	0
rs137871202	C	0.005208	NA	NA	NA	NA	NA	0.01639	NA	NA	NA	0	NA	0	0	NA	0
rs555056776	A	0	NA	NA	NA	NA	NA	0	NA	NA	NA	0	NA	0	0	NA	0.004854
rs553934496	T	0	NA	NA	NA	NA	NA	0	NA	NA	NA	0	NA	0	0	NA	0
rs547701911	C	0	NA	NA	NA	NA	NA	0	NA	NA	NA	0	NA	0	0.005051	NA	0
rs187460831	A	0	NA	NA	NA	NA	NA	0	NA	NA	NA	0	NA	0	0	NA	0.004854
rs422761	A	0.3073	NA	NA	NA	NA	NA	0.2377	NA	NA	NA	0.2442	NA	0.371	0.01515	NA	0.2767
rs186422083	C	0	NA	NA	NA	NA	NA	0	NA	NA	NA	0	NA	0	0	NA	0
rs544946928	T	0	NA	NA	NA	NA	NA	0	NA	NA	NA	0	NA	0	0	NA	0
rs542577849	A	0	NA	NA	NA	NA	NA	0	NA	NA	NA	0	NA	0	0	NA	0
rs561308071	T	0	NA	NA	NA	NA	NA	0	NA	NA	NA	0	NA	0	0	NA	0
rs184146774	G	0	NA	NA	NA	NA	NA	0	NA	NA	NA	0	NA	0	0	NA	0.004854
rs181446489	C	0	NA	NA	NA	NA	NA	0	NA	NA	NA	0	NA	0	0	NA	0
rs543518445	T	0	NA	NA	NA	NA	NA	0	NA	NA	NA	0	NA	0	0	NA	0
rs566732470	A	0	NA	NA	NA	NA	NA	0	NA	NA	NA	0	NA	0	0	NA	0
rs533571483	A	0	NA	NA	NA	NA	NA	0	NA	NA	NA	0.005814	NA	0	0	NA	0
rs531148133	A	0	NA	NA	NA	NA	NA	0	NA	NA	NA	0	NA	0	0	NA	0
rs113288437	G	0.1094	NA	NA	NA	NA	NA	0.09016	NA	NA	NA	0.005814	NA	0.005376	0	NA	0
rs115968373	A	0	NA	NA	NA	NA	NA	0	NA	NA	NA	0	NA	0	0	NA	0
rs140715097	T	0	NA	NA	NA	NA	NA	0	NA	NA	NA	0	NA	0	0	NA	0.004854
rs376897988	T	0	NA	NA	NA	NA	NA	0	NA	NA	NA	0	NA	0	0	NA	0
rs574759417	A	0	NA	NA	NA	NA	NA	0	NA	NA	NA	0	NA	0.005376	0	NA	0
rs528568210	A	0.005208	NA	NA	NA	NA	NA	0	NA	NA	NA	0	NA	0	0	NA	0
rs540209049	C	0	NA	NA	NA	NA	NA	0	NA	NA	NA	0	NA	0	0	NA	0
rs191595416	A	0	NA	NA	NA	NA	NA	0	NA	NA	NA	0	NA	0	0	NA	0
rs66492316	C	0.3021	NA	NA	NA	NA	NA	0.3033	NA	NA	NA	0.2326	NA	0.03763	0.4091	NA	0.009709
rs8126497	A	0.01562	NA	NA	NA	NA	NA	0.05738	NA	NA	NA	0.06395	NA	0.01613	0.2172	NA	0.06796
rs531884709	A	0	NA	NA	NA	NA	NA	0	NA	NA	NA	0	NA	0	0	NA	0
rs547558669	G	0	NA	NA	NA	NA	NA	0	NA	NA	NA	0	NA	0	0	NA	0
rs55704664	T	0.01562	NA	NA	NA	NA	NA	0.05738	NA	NA	NA	0.06395	NA	0.01613	0.2172	NA	0.06796
rs546388820	T	0	NA	NA	NA	NA	NA	0	NA	NA	NA	0	NA	0	0	NA	0
rs142103907	G	0	NA	NA	NA	NA	NA	0	NA	NA	NA	0	NA	0	0	NA	0
rs117656646	C	0.005208	NA	NA	NA	NA	NA	0.008197	NA	NA	NA	0	NA	0	0.0202	NA	0

SNP	Allele	ACB	Afro	Aimaras	Ancash	Arequipa	Ashaninkas	ASW	Awajun	Ayacucho	Bambui	BEB	Candoshi	CDX	CEU	Chachapoyas	CHB
rs139258152	T	0.005208	NA	NA	NA	NA	NA	0.008197	NA	NA	NA	0	NA	0	0	NA	0
rs150382508	C	0.02604	NA	NA	NA	NA	NA	0.03279	NA	NA	NA	0	NA	0	0	NA	0
rs544308006	T	0	NA	NA	NA	NA	NA	0	NA	NA	NA	0	NA	0	0	NA	0
rs551100726	T	0	NA	NA	NA	NA	NA	0	NA	NA	NA	0	NA	0	0	NA	0
rs527435310	A	0	NA	NA	NA	NA	NA	0	NA	NA	NA	0	NA	0.005376	0	NA	0
rs552725417	A	0.005208	NA	NA	NA	NA	NA	0	NA	NA	NA	0	NA	0	0	NA	0
rs549038772	T	0	NA	NA	NA	NA	NA	0	NA	NA	NA	0	NA	0	0	NA	0
rs562574628	G	0	NA	NA	NA	NA	NA	0	NA	NA	NA	0.005814	NA	0	0	NA	0
rs8129713	C	0.04688	0.234	0.1667	0.2083	0.1739	0.05714	0.08197	0.1304	0.2429	0.1915	0.06395	0.1875	0.01613	0.2172	0.1625	0.06796
rs183385542	C	0	NA	NA	NA	NA	NA	0	NA	NA	NA	0	NA	0	0	NA	0
rs553596811	G	0	NA	NA	NA	NA	NA	0	NA	NA	NA	0	NA	0	0	NA	0
rs150473664	C	0	NA	NA	NA	NA	NA	0	NA	NA	NA	0	NA	0	0	NA	0
rs146120690	A	0	NA	NA	NA	NA	NA	0	NA	NA	NA	0	NA	0.005376	0	NA	0.009709
rs527261679	A	0	NA	NA	NA	NA	NA	0	NA	NA	NA	0	NA	0	0	NA	0
rs570544092	T	0	NA	NA	NA	NA	NA	0	NA	NA	NA	0	NA	0	0	NA	0
rs392370	C	0.3802	NA	NA	NA	NA	NA	0.3525	NA	NA	NA	0.2326	NA	0.3011	0.2374	NA	0.335
rs150875127	C	0.4844	NA	NA	NA	NA	NA	0.5	NA	NA	NA	0.5988	NA	0.2581	0.7323	NA	0.3107
rs536039173	T	0	NA	NA	NA	NA	NA	0	NA	NA	NA	0.01163	NA	0	0	NA	0
rs192019778	A	0	NA	NA	NA	NA	NA	0	NA	NA	NA	0	NA	0	0	NA	0
SNP	A1	MAF	MAF	MAF	MAF	MAF	MAF	MAF	MAF	MAF	MAF	MAF	MAF	MAF	MAF	MAF	MAF
rs537728755	A	0.01562	NA	NA	NA	NA	NA	0.01639	NA	NA	NA	0	NA	0	0	NA	0
rs55964536	T	0.1406	NA	NA	NA	NA	NA	0.2049	NA	NA	NA	0.3256	NA	0.005376	0.5051	NA	0.004854
rs373311004	C	0	NA	NA	NA	NA	NA	0	NA	NA	NA	0	NA	0.005376	0	NA	0
rs575968857	T	0	NA	NA	NA	NA	NA	0	NA	NA	NA	0	NA	0	0	NA	0
rs548402221	G	0	NA	NA	NA	NA	NA	0	NA	NA	NA	0	NA	0	0.0202	NA	0
rs140037718	C	0	NA	NA	NA	NA	NA	0	NA	NA	NA	0	NA	0	0	NA	0
rs75373173	C	0.01042	0	0	0	0.02174	0	0.02459	0	0.01429	0.04993	0.02907	0	0	0.08586	0.01282	0
rs568619174	C	0	NA	NA	NA	NA	NA	0	NA	NA	NA	0	NA	0	0	NA	0
rs187175193	T	0	NA	NA	NA	NA	NA	0	NA	NA	NA	0	NA	0	0	NA	0
rs193122175	T	0	NA	NA	NA	NA	NA	0.008197	NA	NA	NA	0	NA	0	0	NA	0
rs117898838	A	0.01042	NA	NA	NA	NA	NA	0	NA	NA	NA	0	NA	0	0.0101	NA	0
rs537989379	A	0	NA	NA	NA	NA	NA	0	NA	NA	NA	0	NA	0	0	NA	0
rs570302987	A	0	NA	NA	NA	NA	NA	0	NA	NA	NA	0	NA	0	0	NA	0
rs190970011	T	0	NA	NA	NA	NA	NA	0	NA	NA	NA	0	NA	0	0	NA	0.004854
rs556994660	C	0	NA	NA	NA	NA	NA	0	NA	NA	NA	0	NA	0.005376	0	NA	0
rs527380714	C	0	NA	NA	NA	NA	NA	0	NA	NA	NA	0	NA	0	0.005051	NA	0
rs536026892	T	0	NA	NA	NA	NA	NA	0	NA	NA	NA	0.005814	NA	0	0	NA	0
rs535103772	T	0	NA	NA	NA	NA	NA	0	NA	NA	NA	0	NA	0	0.005051	NA	0
rs191587330	C	0	NA	NA	NA	NA	NA	0	NA	NA	NA	0	NA	0	0	NA	0
rs553617111	T	0	NA	NA	NA	NA	NA	0	NA	NA	NA	0	NA	0	0	NA	0
rs182838808	A	0	NA	NA	NA	NA	NA	0	NA	NA	NA	0	NA	0	0	NA	0
rs78503214	T	0.02604	NA	NA	NA	NA	NA	0.02459	NA	NA	NA	0	NA	0	0	NA	0
rs142425263	A	0	NA	NA	NA	NA	NA	0	NA	NA	NA	0	NA	0	0	NA	0.004854
rs573213706	A	0	NA	NA	NA	NA	NA	0	NA	NA	NA	0	NA	0	0	NA	0
rs73357663	C	0.01562	NA	NA	NA	NA	NA	0	NA	NA	NA	0	NA	0	0	NA	0

SNP	Allele	ACB	Afro	Aimaras	Ancash	Arequipa	Ashaninkas	ASW	Awajun	Ayacucho	Bambui	BEB	Candoshi	CDX	CEU	Chachapoyas	CHB
rs563670115	C	0	NA	NA	NA	NA	NA	0	NA	NA	NA	0	NA	0	0	NA	0
rs111220509	G	0.4531	NA	NA	NA	NA	NA	0.5	NA	NA	NA	0.593	NA	0.2473	0.7475	NA	0.3107
rs186605876	T	0	NA	NA	NA	NA	NA	0	NA	NA	NA	0	NA	0	0	NA	0
rs373611430	G	0	NA	NA	NA	NA	NA	0	NA	NA	NA	0	NA	0.005376	0	NA	0
rs2094881	C	0.4583	0.4468	0.1562	0.06944	0.2609	0.01429	0.5	0.2391	0.1286	0.7202	0.593	0.125	0.2473	0.7475	0.2	0.3107
rs73372166	A	0.25	NA	NA	NA	NA	NA	0.1885	NA	NA	NA	0.09302	NA	0.02151	0.1313	NA	0.06311
rs76135088	G	0.05208	0.05319	0	0	0	0	0.03279	0	0	0.01664	0.005814	0	0.005376	0	0	0
rs548979551	G	0	NA	NA	NA	NA	NA	0	NA	NA	NA	0	NA	0	0	NA	0
rs540046047	C	0	NA	NA	NA	NA	NA	0	NA	NA	NA	0	NA	0.005376	0	NA	0
rs2410429	A	0.2344	NA	NA	NA	NA	NA	0.2131	NA	NA	NA	0.2907	NA	0.6989	0.2525	NA	0.5874
rs532950488	A	0	NA	NA	NA	NA	NA	0	NA	NA	NA	0	NA	0	0	NA	0
rs535531364	C	0.005208	NA	NA	NA	NA	NA	0	NA	NA	NA	0	NA	0	0	NA	0
rs550091217	T	0	NA	NA	NA	NA	NA	0	NA	NA	NA	0	NA	0	0	NA	0
rs62217527	T	0.01562	NA	NA	NA	NA	NA	0.02459	NA	NA	NA	0.005814	NA	0	0.1364	NA	0.004854
rs80275470	A	0.005208	NA	NA	NA	NA	NA	0	NA	NA	NA	0.005814	NA	0	0.0303	NA	0
rs545165511	A	0	NA	NA	NA	NA	NA	0	NA	NA	NA	0	NA	0	0	NA	0
rs144318842	A	0	NA	NA	NA	NA	NA	0	NA	NA	NA	0	NA	0	0	NA	0
rs200395836	T	0	NA	NA	NA	NA	NA	0	NA	NA	NA	0	NA	0	0	NA	0
rs548552862	C	0	NA	NA	NA	NA	NA	0	NA	NA	NA	0	NA	0	0	NA	0
rs577632959	A	0	NA	NA	NA	NA	NA	0	NA	NA	NA	0	NA	0	0.005051	NA	0
rs567258163	A	0	NA	NA	NA	NA	NA	0	NA	NA	NA	0	NA	0	0	NA	0
rs561789442	A	0	NA	NA	NA	NA	NA	0	NA	NA	NA	0	NA	0	0	NA	0
rs538588854	A	0	NA	NA	NA	NA	NA	0	NA	NA	NA	0	NA	0	0	NA	0
rs115429336	A	0.01562	NA	NA	NA	NA	NA	0.01639	NA	NA	NA	0	NA	0	0	NA	0
rs537412369	T	0	NA	NA	NA	NA	NA	0	NA	NA	NA	0	NA	0	0	NA	0.004854
rs371531071	T	0	NA	NA	NA	NA	NA	0	NA	NA	NA	0	NA	0	0	NA	0
rs183141812	A	0	NA	NA	NA	NA	NA	0	NA	NA	NA	0	NA	0	0	NA	0
rs189546747	T	0	NA	NA	NA	NA	NA	0	NA	NA	NA	0	NA	0	0	NA	0
rs3787950	C	0.1875	0.08511	0	0.01429	0.06818	0	0.1803	0	0.02857	0.1154	0.25	0	0.1774	0.09091	0	0.1165
rs549067055	A	0	NA	NA	NA	NA	NA	0	NA	NA	NA	0	NA	0	0	NA	0
rs576955441	A	0	NA	NA	NA	NA	NA	0	NA	NA	NA	0	NA	0	0	NA	0
rs8134203	T	0.4688	NA	NA	NA	NA	NA	0.4918	NA	NA	NA	0.593	NA	0.2473	0.7475	NA	0.3058
rs532216261	C	0	NA	NA	NA	NA	NA	0	NA	NA	NA	0	NA	0	0	NA	0.004854
rs557766326	T	0	NA	NA	NA	NA	NA	0	NA	NA	NA	0	NA	0	0	NA	0
rs546531769	G	0	NA	NA	NA	NA	NA	0	NA	NA	NA	0	NA	0	0.005051	NA	0
rs571993620	A	0	NA	NA	NA	NA	NA	0	NA	NA	NA	0	NA	0	0	NA	0
rs374261644	C	0	NA	NA	NA	NA	NA	0	NA	NA	NA	0	NA	0	0	NA	0
rs564210410	A	0.02083	NA	NA	NA	NA	NA	0	NA	NA	NA	0	NA	0	0	NA	0
rs562387776	C	0	NA	NA	NA	NA	NA	0	NA	NA	NA	0	NA	0.005376	0	NA	0
rs562587205	T	0	NA	NA	NA	NA	NA	0	NA	NA	NA	0	NA	0	0	NA	0
rs542538936	T	0	NA	NA	NA	NA	NA	0	NA	NA	NA	0	NA	0	0	NA	0
rs143818732	C	0	NA	NA	NA	NA	NA	0	NA	NA	NA	0.01163	NA	0	0.0101	NA	0
rs183650725	A	0	NA	NA	NA	NA	NA	0	NA	NA	NA	0	NA	0	0	NA	0
rs531439352	A	0	NA	NA	NA	NA	NA	0	NA	NA	NA	0	NA	0	0	NA	0
rs531183955	T	0	NA	NA	NA	NA	NA	0	NA	NA	NA	0.005814	NA	0	0	NA	0.004854

SNP	Allele	ACB	Afro	Aimaras	Ancash	Arequipa	Ashaninkas	ASW	Awajun	Ayacucho	Bambui	BEB	Candoshi	CDX	CEU	Chachapoyas	CHB
rs144192191	GGTGA	0.3073	NA	NA	NA	NA	NA	0.2705	NA	NA	NA	0.2616	NA	0.2419	0.2475	NA	0.3058
rs149708827	T	0	NA	NA	NA	NA	NA	0	NA	NA	NA	0	NA	0.005376	0	NA	0.01456
rs552635816	G	0	NA	NA	NA	NA	NA	0	NA	NA	NA	0	NA	0	0	NA	0
rs539520259	T	0	NA	NA	NA	NA	NA	0	NA	NA	NA	0	NA	0	0	NA	0
rs376235035	T	0	NA	NA	NA	NA	NA	0	NA	NA	NA	0	NA	0	0	NA	0.004854
rs142303004	T	0	NA	NA	NA	NA	NA	0	NA	NA	NA	0	NA	0	0	NA	0
rs546232086	G	0	NA	NA	NA	NA	NA	0	NA	NA	NA	0	NA	0	0	NA	0
rs549832756	C	0	NA	NA	NA	NA	NA	0	NA	NA	NA	0	NA	0	0	NA	0
rs75655573	G	0	0	0	0	0	0	0	0	0	0.003814	0	0	0	0	0	0
rs553167698	T	0	NA	NA	NA	NA	NA	0	NA	NA	NA	0	NA	0	0	NA	0.004854
rs190685013	A	0	NA	NA	NA	NA	NA	0.01639	NA	NA	NA	0	NA	0	0	NA	0
rs559934921	G	0	NA	NA	NA	NA	NA	0	NA	NA	NA	0	NA	0	0	NA	0
rs190899605	C	0	NA	NA	NA	NA	NA	0	NA	NA	NA	0.005814	NA	0.005376	0	NA	0
rs391099	C	0.3802	NA	NA	NA	NA	NA	0.3525	NA	NA	NA	0.2326	NA	0.3011	0.2374	NA	0.335
rs372422423	A	0.03125	NA	NA	NA	NA	NA	0.008197	NA	NA	NA	0	NA	0	0	NA	0
rs77014365	A	0	NA	NA	NA	NA	NA	0	NA	NA	NA	0	NA	0	0.005051	NA	0
rs562157895	A	0	NA	NA	NA	NA	NA	0	NA	NA	NA	0.01744	NA	0	0	NA	0
rs187290362	G	0	NA	NA	NA	NA	NA	0.01639	NA	NA	NA	0	NA	0	0	NA	0
rs182543256	A	0	NA	NA	NA	NA	NA	0	NA	NA	NA	0	NA	0	0	NA	0
rs563186329	C	0	NA	NA	NA	NA	NA	0	NA	NA	NA	0	NA	0	0	NA	0
rs569026066	T	0	NA	NA	NA	NA	NA	0	NA	NA	NA	0	NA	0	0	NA	0
rs116511699	T	0	NA	NA	NA	NA	NA	0.01639	NA	NA	NA	0	NA	0	0	NA	0
rs148499598	C	0	NA	NA	NA	NA	NA	0	NA	NA	NA	0	NA	0	0	NA	0
rs552256264	T	0	NA	NA	NA	NA	NA	0.008197	NA	NA	NA	0	NA	0	0	NA	0
rs562776963	A	0	NA	NA	NA	NA	NA	0	NA	NA	NA	0	NA	0	0	NA	0
rs79243099	A	0.04688	NA	NA	NA	NA	NA	0.02459	NA	NA	NA	0	NA	0	0	NA	0
rs79397218	A	0	NA	NA	NA	NA	NA	0	NA	NA	NA	0.01744	NA	0	0	NA	0
rs138094318	T	0	NA	NA	NA	NA	NA	0	NA	NA	NA	0	NA	0	0	NA	0
rs567275769	T	0	NA	NA	NA	NA	NA	0	NA	NA	NA	0	NA	0	0	NA	0
rs544297320	T	0	NA	NA	NA	NA	NA	0	NA	NA	NA	0.005814	NA	0	0	NA	0
rs561404433	G	0	NA	NA	NA	NA	NA	0	NA	NA	NA	0	NA	0	0	NA	0
rs573923452	A	0	NA	NA	NA	NA	NA	0	NA	NA	NA	0	NA	0.005376	0	NA	0
rs539858204	A	0.005208	NA	NA	NA	NA	NA	0	NA	NA	NA	0	NA	0	0	NA	0
rs9305745	T	0.3594	NA	NA	NA	NA	NA	0.418	NA	NA	NA	0.3023	NA	0.1935	0.2525	NA	0.3398
rs546510868	T	0	NA	NA	NA	NA	NA	0	NA	NA	NA	0	NA	0	0	NA	0
rs183418223	A	0.005208	NA	NA	NA	NA	NA	0	NA	NA	NA	0	NA	0	0	NA	0
rs573692135	T	0	NA	NA	NA	NA	NA	0	NA	NA	NA	0	NA	0	0	NA	0
rs570126729	A	0	NA	NA	NA	NA	NA	0	NA	NA	NA	0	NA	0	0	NA	0
rs542946711	G	0	NA	NA	NA	NA	NA	0	NA	NA	NA	0.02907	NA	0	0	NA	0
rs551277697	G	0	NA	NA	NA	NA	NA	0	NA	NA	NA	0	NA	0	0	NA	0
rs73230088	A	0.03125	NA	NA	NA	NA	NA	0.09016	NA	NA	NA	0.1163	NA	0.1129	0.1768	NA	0.06311
rs548025657	A	0	NA	NA	NA	NA	NA	0	NA	NA	NA	0	NA	0	0	NA	0
rs140428704	T	0	NA	NA	NA	NA	NA	0	NA	NA	NA	0	NA	0	0	NA	0
rs184149889	C	0	NA	NA	NA	NA	NA	0	NA	NA	NA	0	NA	0	0	NA	0
rs116568213	G	0.005208	NA	NA	NA	NA	NA	0.03279	NA	NA	NA	0	NA	0	0	NA	0

SNP	Allele	ACB	Afro	Aimaras	Ancash	Arequipa	Ashaninkas	ASW	Awajun	Ayacucho	Bambui	BEB	Candoshi	CDX	CEU	Chachapoyas	CHB
rs187742976	A	0	NA	NA	NA	NA	NA	0	NA	NA	NA	0	NA	0	0	NA	0
rs555769183	C	0	NA	NA	NA	NA	NA	0	NA	NA	NA	0	NA	0	0	NA	0
rs12627374	T	0.005208	0	0	0	0	0	0.01639	0	0	0.0003467	0.1744	0	0.1774	0	0	0.1359
rs565973484	A	0	NA	NA	NA	NA	NA	0	NA	NA	NA	0	NA	0	0	NA	0
rs547257017	C	0	NA	NA	NA	NA	NA	0	NA	NA	NA	0	NA	0	0	NA	0
rs568737536	A	0	NA	NA	NA	NA	NA	0	NA	NA	NA	0	NA	0	0	NA	0
rs541239376	T	0	NA	NA	NA	NA	NA	0	NA	NA	NA	0	NA	0	0.005051	NA	0
rs557500448	A	0	NA	NA	NA	NA	NA	0	NA	NA	NA	0	NA	0	0	NA	0
rs185166990	A	0	NA	NA	NA	NA	NA	0	NA	NA	NA	0	NA	0.01613	0	NA	0
rs541215881	A	0	NA	NA	NA	NA	NA	0	NA	NA	NA	0	NA	0	0	NA	0
rs138995130	A	0	NA	NA	NA	NA	NA	0	NA	NA	NA	0	NA	0	0	NA	0
rs462448	T	0.005208	NA	NA	NA	NA	NA	0.02459	NA	NA	NA	0.0814	NA	0.4032	0.02525	NA	0.2718
rs569303172	T	0	NA	NA	NA	NA	NA	0	NA	NA	NA	0	NA	0	0	NA	0
rs564691729	C	0	NA	NA	NA	NA	NA	0	NA	NA	NA	0	NA	0	0	NA	0
rs187452528	C	0	NA	NA	NA	NA	NA	0	NA	NA	NA	0	NA	0	0	NA	0
rs4818242	T	0	NA	NA	NA	NA	NA	0.008197	NA	NA	NA	0	NA	0	0	NA	0
rs183016576	T	0	NA	NA	NA	NA	NA	0	NA	NA	NA	0	NA	0	0	NA	0
rs539934676	A	0	NA	NA	NA	NA	NA	0	NA	NA	NA	0	NA	0	0	NA	0
rs184876485	G	0	NA	NA	NA	NA	NA	0	NA	NA	NA	0	NA	0	0	NA	0
rs549676267	T	0	NA	NA	NA	NA	NA	0	NA	NA	NA	0	NA	0	0	NA	0
rs150633108	T	0	NA	NA	NA	NA	NA	0	NA	NA	NA	0.04651	NA	0	0	NA	0
rs185103560	A	0	NA	NA	NA	NA	NA	0	NA	NA	NA	0	NA	0	0	NA	0
rs532655542	G	0	NA	NA	NA	NA	NA	0	NA	NA	NA	0	NA	0	0	NA	0
rs180831387	A	0	NA	NA	NA	NA	NA	0	NA	NA	NA	0	NA	0	0	NA	0.004854
rs569577322	T	0	NA	NA	NA	NA	NA	0	NA	NA	NA	0	NA	0.005376	0	NA	0
rs144154504	C	0	NA	NA	NA	NA	NA	0	NA	NA	NA	0	NA	0	0	NA	0
rs556937466	T	0	NA	NA	NA	NA	NA	0	NA	NA	NA	0	NA	0	0	NA	0
rs115129572	T	0	0.02128	0	0	0	0	0.008197	0	0	0.003814	0	0	0	0	0	0
rs578015897	T	0	NA	NA	NA	NA	NA	0	NA	NA	NA	0	NA	0	0	NA	0
rs2298661	A	0.2865	NA	NA	NA	NA	NA	0.2869	NA	NA	NA	0.3256	NA	0.3495	0.2222	NA	0.4223
rs557088853	T	0	NA	NA	NA	NA	NA	0	NA	NA	NA	0.005814	NA	0	0	NA	0
rs141301979	C	0	NA	NA	NA	NA	NA	0.008197	NA	NA	NA	0	NA	0	0	NA	0
rs8131649	T	0.4844	0.5319	0.8438	0.8472	0.7391	0.8714	0.459	0.7609	0.8429	0.268	0.407	0.875	0.7527	0.2525	0.7875	0.6942
rs539860607	G	0.005208	NA	NA	NA	NA	NA	0	NA	NA	NA	0	NA	0	0	NA	0
rs4816720	C	0.1927	NA	NA	NA	NA	NA	0.1885	NA	NA	NA	0.0814	NA	0.4516	0.0303	NA	0.2767
rs11702475	T	0.1719	NA	NA	NA	NA	NA	0.2049	NA	NA	NA	0.3953	NA	0.01075	0.5051	NA	0.004854
rs150205743	A	0.01042	NA	NA	NA	NA	NA	0	NA	NA	NA	0	NA	0	0	NA	0
rs8127674	G	0.3281	0.1915	0.03125	0.1528	0.2174	0.1286	0.2541	0.2174	0.1	0.3707	0.2151	0.1562	0.03763	0.3889	0.0875	0.009709
rs190134102	A	0	NA	NA	NA	NA	NA	0.008197	NA	NA	NA	0	NA	0	0	NA	0
rs141324737	T	0	NA	NA	NA	NA	NA	0.01639	NA	NA	NA	0	NA	0	0	NA	0
rs560898303	CA	0	NA	NA	NA	NA	NA	0	NA	NA	NA	0	NA	0	0	NA	0
rs552105586	T	0	NA	NA	NA	NA	NA	0	NA	NA	NA	0	NA	0	0	NA	0
rs146052428	T	0.005208	NA	NA	NA	NA	NA	0.008197	NA	NA	NA	0	NA	0	0	NA	0
rs188241223	T	0	NA	NA	NA	NA	NA	0	NA	NA	NA	0	NA	0	0	NA	0
rs547639377	G	0	NA	NA	NA	NA	NA	0	NA	NA	NA	0.005814	NA	0	0	NA	0

SNP	Allele	ACB	Afro	Aimaras	Ancash	Arequipa	Ashaninkas	ASW	Awajun	Ayacucho	Bambui	BEB	Candoshi	CDX	CEU	Chachapoyas	CHB
rs420737	G	0.3021	NA	NA	NA	NA	NA	0.2295	NA	NA	NA	0.2442	NA	0.3925	0.01515	NA	0.2767
rs534978728	G	0	NA	NA	NA	NA	NA	0	NA	NA	NA	0	NA	0	0	NA	0
rs369948528	T	0	NA	NA	NA	NA	NA	0	NA	NA	NA	0	NA	0	0	NA	0
rs533867263	A	0	NA	NA	NA	NA	NA	0.008197	NA	NA	NA	0	NA	0	0	NA	0
rs555525436	G	0	NA	NA	NA	NA	NA	0	NA	NA	NA	0.005814	NA	0	0	NA	0
rs531121505	T	0	NA	NA	NA	NA	NA	0	NA	NA	NA	0	NA	0	0	NA	0.004854
rs376143876	A	0	NA	NA	NA	NA	NA	0	NA	NA	NA	0	NA	0	0	NA	0
rs554986236	T	0	NA	NA	NA	NA	NA	0	NA	NA	NA	0	NA	0	0	NA	0.004854
rs66575656	T	0.2135	NA	NA	NA	NA	NA	0.1967	NA	NA	NA	0.2558	NA	0.2366	0.2475	NA	0.3058
rs542746297	T	0	NA	NA	NA	NA	NA	0	NA	NA	NA	0.005814	NA	0	0	NA	0
rs567175997	G	0	NA	NA	NA	NA	NA	0	NA	NA	NA	0	NA	0	0	NA	0.004854
rs578020496	C	0	NA	NA	NA	NA	NA	0	NA	NA	NA	0	NA	0	0	NA	0
rs543607180	C	0	NA	NA	NA	NA	NA	0	NA	NA	NA	0	NA	0	0	NA	0
rs115849825	A	0.005208	NA	NA	NA	NA	NA	0	NA	NA	NA	0	NA	0	0	NA	0
rs542333608	T	0	NA	NA	NA	NA	NA	0	NA	NA	NA	0	NA	0	0	NA	0
rs34983238	C	0.02083	NA	NA	NA	NA	NA	0.04098	NA	NA	NA	0.05233	NA	0	0.101	NA	0
rs372733383	T	0	NA	NA	NA	NA	NA	0	NA	NA	NA	0	NA	0	0	NA	0
rs555134126	T	0	NA	NA	NA	NA	NA	0	NA	NA	NA	0	NA	0	0	NA	0
rs533043037	A	0	NA	NA	NA	NA	NA	0	NA	NA	NA	0	NA	0	0	NA	0
rs73372168	A	0.01562	NA	NA	NA	NA	NA	0	NA	NA	NA	0	NA	0	0	NA	0
rs150715708	T	0	NA	NA	NA	NA	NA	0	NA	NA	NA	0	NA	0	0	NA	0
rs145024812	A	0.02604	NA	NA	NA	NA	NA	0.03279	NA	NA	NA	0	NA	0	0	NA	0
rs557592331	C	0.005208	NA	NA	NA	NA	NA	0	NA	NA	NA	0	NA	0	0	NA	0
rs567380110	T	0	NA	NA	NA	NA	NA	0	NA	NA	NA	0	NA	0.005376	0	NA	0
rs560408119	A	0	NA	NA	NA	NA	NA	0	NA	NA	NA	0	NA	0	0	NA	0
rs117432315	A	0	NA	NA	NA	NA	NA	0	NA	NA	NA	0	NA	0	0	NA	0.01456
rs370654859	C	0.02604	NA	NA	NA	NA	NA	0.03279	NA	NA	NA	0	NA	0	0	NA	0
rs139181746	C	0	NA	NA	NA	NA	NA	0	NA	NA	NA	0	NA	0	0	NA	0.004854
rs554175681	G	0	NA	NA	NA	NA	NA	0	NA	NA	NA	0	NA	0	0	NA	0
rs28548447	A	0.2188	NA	NA	NA	NA	NA	0.2049	NA	NA	NA	0.2442	NA	0.2366	0.298	NA	0.301
rs191526916	T	0	NA	NA	NA	NA	NA	0	NA	NA	NA	0	NA	0	0	NA	0
rs192959767	T	0	NA	NA	NA	NA	NA	0	NA	NA	NA	0	NA	0	0	NA	0
rs534192391	T	0	NA	NA	NA	NA	NA	0	NA	NA	NA	0	NA	0	0	NA	0
rs571211518	C	0	NA	NA	NA	NA	NA	0	NA	NA	NA	0	NA	0	0	NA	0
rs562033822	A	0	NA	NA	NA	NA	NA	0	NA	NA	NA	0	NA	0	0	NA	0
rs142194573	AG	0	NA	NA	NA	NA	NA	0	NA	NA	NA	0	NA	0	0	NA	0
rs149695119	T	0.1562	NA	NA	NA	NA	NA	0.09016	NA	NA	NA	0.005814	NA	0	0	NA	0
rs143270468	A	0	NA	NA	NA	NA	NA	0	NA	NA	NA	0.005814	NA	0	0	NA	0
rs534423780	A	0	NA	NA	NA	NA	NA	0	NA	NA	NA	0	NA	0	0	NA	0
rs535545388	G	0	NA	NA	NA	NA	NA	0	NA	NA	NA	0.005814	NA	0	0	NA	0
rs528206157	T	0	NA	NA	NA	NA	NA	0	NA	NA	NA	0	NA	0	0	NA	0
rs112985398	C	0.01562	NA	NA	NA	NA	NA	0.008197	NA	NA	NA	0	NA	0	0	NA	0
rs77511690	A	0.05729	NA	NA	NA	NA	NA	0.01639	NA	NA	NA	0	NA	0	0	NA	0
rs538161583	A	0	NA	NA	NA	NA	NA	0	NA	NA	NA	0	NA	0	0	NA	0
rs552591104	C	0	NA	NA	NA	NA	NA	0	NA	NA	NA	0	NA	0	0	NA	0

SNP	Allele	ACB	Afro	Aimaras	Ancash	Arequipa	Ashaninkas	ASW	Awajun	Ayacucho	Bambui	BEB	Candoshi	CDX	CEU	Chachapoyas	CHB
rs557224603	A	0	NA	NA	NA	NA	NA	0	NA	NA	NA	0	NA	0	0	NA	0
rs545825254	G	0	NA	NA	NA	NA	NA	0	NA	NA	NA	0	NA	0	0	NA	0
rs185312677	T	0	NA	NA	NA	NA	NA	0	NA	NA	NA	0	NA	0	0	NA	0
rs423596	T	0.01562	NA	NA	NA	NA	NA	0.008197	NA	NA	NA	0.1453	NA	0.2742	0.03535	NA	0.2913
rs541726793	A	0	NA	NA	NA	NA	NA	0	NA	NA	NA	0	NA	0	0	NA	0
rs62217529	T	0	NA	NA	NA	NA	NA	0	NA	NA	NA	0	NA	0	0	NA	0
rs528452414	C	0	NA	NA	NA	NA	NA	0	NA	NA	NA	0	NA	0	0	NA	0
rs143167369	T	0.005208	NA	NA	NA	NA	NA	0	NA	NA	NA	0	NA	0	0	NA	0
rs138164084	T	0	NA	NA	NA	NA	NA	0	NA	NA	NA	0	NA	0	0	NA	0
rs62217531	T	0.3438	0.2021	0.1562	0.1389	0.1087	0.1143	0.3689	0.1957	0.1286	0.4122	0.3895	0.0625	0.01075	0.5	0.175	0.004854
rs528205318	G	0	NA	NA	NA	NA	NA	0	NA	NA	NA	0	NA	0	0	NA	0
rs554097496	A	0	NA	NA	NA	NA	NA	0	NA	NA	NA	0	NA	0	0	NA	0
rs61728255	A	0.005208	NA	NA	NA	NA	NA	0.02459	NA	NA	NA	0.0814	NA	0.4032	0.02525	NA	0.2524
rs538022064	C	0	NA	NA	NA	NA	NA	0	NA	NA	NA	0	NA	0	0	NA	0
rs9980225	C	0.01042	NA	NA	NA	NA	NA	0.04098	NA	NA	NA	0.09302	NA	0.02151	0.1313	NA	0.06796
rs542530281	C	0	NA	NA	NA	NA	NA	0	NA	NA	NA	0	NA	0	0	NA	0
rs542407537	A	0	NA	NA	NA	NA	NA	0	NA	NA	NA	0	NA	0	0	NA	0
rs548552823	A	0	NA	NA	NA	NA	NA	0	NA	NA	NA	0	NA	0.01075	0	NA	0
rs565903681	C	0	NA	NA	NA	NA	NA	0	NA	NA	NA	0	NA	0	0	NA	0
rs201109436	G	0	NA	NA	NA	NA	NA	0	NA	NA	NA	0	NA	0	0	NA	0
rs577393745	T	0	NA	NA	NA	NA	NA	0	NA	NA	NA	0	NA	0	0	NA	0.004854
rs185086499	T	0	NA	NA	NA	NA	NA	0	NA	NA	NA	0	NA	0	0	NA	0.004854
rs141230106	T	0	NA	NA	NA	NA	NA	0	NA	NA	NA	0	NA	0.01075	0	NA	0.004854
rs531548362	C	0	NA	NA	NA	NA	NA	0	NA	NA	NA	0	NA	0	0	NA	0
rs150445636	A	0	NA	NA	NA	NA	NA	0	NA	NA	NA	0	NA	0	0	NA	0
rs139015396	T	0.02604	NA	NA	NA	NA	NA	0.03279	NA	NA	NA	0	NA	0	0	NA	0
rs183722985	A	0	NA	NA	NA	NA	NA	0	NA	NA	NA	0	NA	0	0	NA	0
rs543734531	A	0	NA	NA	NA	NA	NA	0.008197	NA	NA	NA	0	NA	0	0	NA	0
rs553875292	T	0.005208	NA	NA	NA	NA	NA	0	NA	NA	NA	0	NA	0	0	NA	0
rs55760462	C	0.2448	0.1596	0.09375	0.1389	0.1087	0.1286	0.2295	0.2391	0.07143	0.2514	0.1453	0.1562	0.03763	0.2374	0.0875	0.009709
rs61735790	C	0.01042	NA	NA	NA	NA	NA	0	NA	NA	NA	0	NA	0	0	NA	0
rs566918037	G	0	NA	NA	NA	NA	NA	0	NA	NA	NA	0	NA	0	0.005051	NA	0
rs555820997	G	0	NA	NA	NA	NA	NA	0.008197	NA	NA	NA	0	NA	0	0	NA	0
rs573493693	A	0	NA	NA	NA	NA	NA	0	NA	NA	NA	0	NA	0	0	NA	0
rs914184	A	0	NA	NA	NA	NA	NA	0	NA	NA	NA	0.005814	NA	0.05914	0	NA	0.03883
rs539019494	C	0	NA	NA	NA	NA	NA	0	NA	NA	NA	0	NA	0	0	NA	0
rs544036587	T	0	NA	NA	NA	NA	NA	0	NA	NA	NA	0	NA	0	0	NA	0
rs565896303	A	0	NA	NA	NA	NA	NA	0	NA	NA	NA	0	NA	0	0	NA	0
rs192793047	C	0	NA	NA	NA	NA	NA	0	NA	NA	NA	0	NA	0	0	NA	0
rs546790067	C	0	NA	NA	NA	NA	NA	0	NA	NA	NA	0	NA	0.01613	0	NA	0
rs553672299	A	0	NA	NA	NA	NA	NA	0	NA	NA	NA	0	NA	0	0	NA	0
rs570629130	A	0	NA	NA	NA	NA	NA	0	NA	NA	NA	0	NA	0	0	NA	0
rs117562633	C	0	NA	NA	NA	NA	NA	0	NA	NA	NA	0	NA	0	0	NA	0.01942
rs395584	C	0.3021	0.3298	0.375	0.5	0.4565	0.4714	0.2295	0.587	0.4857	0.1176	0.2442	0.4375	0.3871	0.01515	0.4	0.2767
rs143597099	A	NA	NA	NA	NA	NA	NA	NA	NA	NA	NA	NA	NA	NA	NA	NA	NA

SNP	Allele	ACB	Afro	Aimaras	Ancash	Arequipa	Ashaninkas	ASW	Awajun	Ayacucho	Bambui	BEB	Candoshi	CDX	CEU	Chachapoyas	CHB
rs543168691	C	0	NA	NA	NA	NA	NA	0	NA	NA	NA	0	NA	0	0	NA	0
rs530689404	T	0	NA	NA	NA	NA	NA	0	NA	NA	NA	0	NA	0	0	NA	0
rs76833541	A	0.005208	NA	NA	NA	NA	NA	0.02459	NA	NA	NA	0.02907	NA	0	0.08586	NA	0
rs150907799	T	0.005208	NA	NA	NA	NA	NA	0.01639	NA	NA	NA	0.005814	NA	0	0	NA	0
rs554550449	A	0	NA	NA	NA	NA	NA	0	NA	NA	NA	0	NA	0	0	NA	0
rs566085188	A	0	NA	NA	NA	NA	NA	0	NA	NA	NA	0	NA	0	0	NA	0
rs553431242	G	0	NA	NA	NA	NA	NA	0	NA	NA	NA	0	NA	0	0	NA	0
rs551442787	A	0	NA	NA	NA	NA	NA	0	NA	NA	NA	0	NA	0	0	NA	0
rs551677463	C	0	NA	NA	NA	NA	NA	0	NA	NA	NA	0	NA	0	0	NA	0
rs549801931	T	0	NA	NA	NA	NA	NA	0	NA	NA	NA	0	NA	0	0	NA	0
rs141864728	C	0.005208	NA	NA	NA	NA	NA	0.01639	NA	NA	NA	0	NA	0	0	NA	0
rs373480225	T	0	NA	NA	NA	NA	NA	0	NA	NA	NA	0	NA	0	0	NA	0
rs73903405	A	0.06771	NA	NA	NA	NA	NA	0.04918	NA	NA	NA	0	NA	0	0	NA	0
rs567039032	A	0	NA	NA	NA	NA	NA	0	NA	NA	NA	0	NA	0	0	NA	0
rs2070792	A	0.4427	0.3298	0	0	0.1522	0	0.3525	0.04348	0	0.3717	0.2616	0.0625	0.2419	0.298	0.0375	0.2961
rs552550910	A	0	NA	NA	NA	NA	NA	0	NA	NA	NA	0	NA	0.01075	0	NA	0
rs535778149	A	0	NA	NA	NA	NA	NA	0	NA	NA	NA	0	NA	0	0.005051	NA	0
rs201984814	A	0	NA	NA	NA	NA	NA	0	NA	NA	NA	0	NA	0	0.005051	NA	0
rs544847277	A	0	NA	NA	NA	NA	NA	0	NA	NA	NA	0	NA	0	0	NA	0
rs2070793	G	0.4427	NA	NA	NA	NA	NA	0.3525	NA	NA	NA	0.2616	NA	0.2419	0.298	NA	0.2961
rs191345780	A	0	NA	NA	NA	NA	NA	0	NA	NA	NA	0	NA	0	0	NA	0
rs9980693	A	0.03125	NA	NA	NA	NA	NA	0.09016	NA	NA	NA	0.1163	NA	0.1129	0.1768	NA	0.06311
rs146865760	T	0	NA	NA	NA	NA	NA	0	NA	NA	NA	0	NA	0	0.0202	NA	0
rs527898967	G	0	NA	NA	NA	NA	NA	0	NA	NA	NA	0	NA	0	0	NA	0
rs150389990	T	NA	NA	NA	NA	NA	NA	NA	NA	NA	NA	NA	NA	NA	NA	NA	NA
rs558492934	T	0	NA	NA	NA	NA	NA	0	NA	NA	NA	0	NA	0	0	NA	0
rs533513206	C	0	NA	NA	NA	NA	NA	0	NA	NA	NA	0	NA	0	0	NA	0
rs183758144	A	0.005208	NA	NA	NA	NA	NA	0	NA	NA	NA	0	NA	0	0.005051	NA	0
rs377403765	A	0.005208	NA	NA	NA	NA	NA	0	NA	NA	NA	0	NA	0	0	NA	0
rs543740889	C	0	NA	NA	NA	NA	NA	0	NA	NA	NA	0	NA	0	0	NA	0
rs563188125	A	0	NA	NA	NA	NA	NA	0	NA	NA	NA	0	NA	0	0	NA	0
rs536677167	G	0	NA	NA	NA	NA	NA	0	NA	NA	NA	0	NA	0	0	NA	0
rs541782164	T	0	NA	NA	NA	NA	NA	0	NA	NA	NA	0.02326	NA	0	0	NA	0
rs138161288	T	0	NA	NA	NA	NA	NA	0	NA	NA	NA	0	NA	0	0.005051	NA	0
rs541499151	A	0	NA	NA	NA	NA	NA	0	NA	NA	NA	0.005814	NA	0	0	NA	0
rs543339705	C	0	NA	NA	NA	NA	NA	0	NA	NA	NA	0	NA	0	0	NA	0
rs77599791	G	0	NA	NA	NA	NA	NA	0.02459	NA	NA	NA	0	NA	0	0	NA	0
rs549745562	G	0	NA	NA	NA	NA	NA	0	NA	NA	NA	0	NA	0.005376	0	NA	0
rs75317729	C	0.005208	NA	NA	NA	NA	NA	0	NA	NA	NA	0	NA	0	0	NA	0
rs139661079	A	0.01562	NA	NA	NA	NA	NA	0.008197	NA	NA	NA	0	NA	0	0	NA	0
rs572699613	A	0.005208	NA	NA	NA	NA	NA	0	NA	NA	NA	0	NA	0	0	NA	0
rs536023174	T	0	NA	NA	NA	NA	NA	0	NA	NA	NA	0	NA	0	0	NA	0
rs415731	A	0.1927	NA	NA	NA	NA	NA	0.2787	NA	NA	0.3236	0.2151	NA	0.2312	0.3434	NA	0.3107
rs3761374	C	0.01562	0.01087	0	0.02778	0.02174	0	0.08197	0	0.02857	0.1287	0.1221	0	0.1129	0.1768	0.0375	0.06311
rs147233451	T	0	NA	NA	NA	NA	NA	0	NA	NA	NA	0	NA	0	0	NA	0

SNP	Allele	ACB	Afro	Aimaras	Ancash	Arequipa	Ashaninkas	ASW	Awajun	Ayacucho	Bambui	BEB	Candoshi	CDX	CEU	Chachapoyas	CHB
rs143595083	T	0.02604	NA	NA	NA	NA	NA	0.03279	NA	NA	NA	0	NA	0	0	NA	0
rs565237319	C	0	NA	NA	NA	NA	NA	0	NA	NA	NA	0	NA	0	0	NA	0
rs576510757	G	0	NA	NA	NA	NA	NA	0.01639	NA	NA	NA	0	NA	0	0	NA	0
rs535235411	A	0	NA	NA	NA	NA	NA	0	NA	NA	NA	0	NA	0	0	NA	0
rs190265904	A	0	NA	NA	NA	NA	NA	0	NA	NA	NA	0	NA	0	0	NA	0.004854
rs149527323	A	0	NA	NA	NA	NA	NA	0	NA	NA	NA	0	NA	0	0	NA	0
rs544141276	A	0	NA	NA	NA	NA	NA	0	NA	NA	NA	0.005814	NA	0	0	NA	0
rs186097675	T	0	NA	NA	NA	NA	NA	0	NA	NA	NA	0	NA	0	0	NA	0
rs547168890	A	0	NA	NA	NA	NA	NA	0	NA	NA	NA	0.005814	NA	0	0	NA	0
rs138928680	GAGAA	0	NA	NA	NA	NA	NA	0	NA	NA	NA	0	NA	0	0	NA	0
rs567676569	T	0	NA	NA	NA	NA	NA	0	NA	NA	NA	0.01163	NA	0	0	NA	0
rs530701233	A	0	NA	NA	NA	NA	NA	0	NA	NA	NA	0	NA	0	0	NA	0
rs562332833	T	0	NA	NA	NA	NA	NA	0	NA	NA	NA	0	NA	0	0	NA	0
rs531545827	T	0	NA	NA	NA	NA	NA	0	NA	NA	NA	0	NA	0	0	NA	0
rs530584676	T	0	NA	NA	NA	NA	NA	0	NA	NA	NA	0	NA	0	0	NA	0
rs185416185	T	0	NA	NA	NA	NA	NA	0	NA	NA	NA	0	NA	0	0	NA	0
rs429442	T	0.3385	NA	NA	NA	NA	NA	0.3115	NA	NA	NA	0.2326	NA	0.3011	0.2273	NA	0.3252
rs79065748	A	0.03646	NA	NA	NA	NA	NA	0.02459	NA	NA	NA	0	NA	0	0	NA	0
rs575760704	C	0	NA	NA	NA	NA	NA	0	NA	NA	NA	0	NA	0	0	NA	0
rs875393	A	0	NA	NA	NA	NA	NA	0.01639	NA	NA	NA	0.05233	NA	0.1075	0.04545	NA	0.233
rs148901354	T	0	NA	NA	NA	NA	NA	0	NA	NA	NA	0	NA	0	0	NA	0
rs561279116	G	0	NA	NA	NA	NA	NA	0	NA	NA	NA	0	NA	0	0	NA	0
rs138661151	A	0	NA	NA	NA	NA	NA	0.01639	NA	NA	NA	0	NA	0	0	NA	0
rs9975014	G	0.2188	NA	NA	NA	NA	NA	0.2131	NA	NA	NA	0.2209	NA	0.2419	0.2323	NA	0.3058
rs573564326	A	0	NA	NA	NA	NA	NA	0.008197	NA	NA	NA	0	NA	0	0	NA	0.004854
rs144276163	G	0	NA	NA	NA	NA	NA	0	NA	NA	NA	0	NA	0	0	NA	0
rs142750000	T	0	NA	NA	NA	NA	NA	0	NA	NA	NA	0	NA	0	0	NA	0
rs143672898	T	0	NA	NA	NA	NA	NA	0	NA	NA	NA	0	NA	0	0.005051	NA	0
rs560182574	C	0	NA	NA	NA	NA	NA	0	NA	NA	NA	0	NA	0	0	NA	0
rs534312135	T	0	NA	NA	NA	NA	NA	0	NA	NA	NA	0	NA	0	0	NA	0
rs147986574	A	0	NA	NA	NA	NA	NA	0.01639	NA	NA	NA	0	NA	0	0	NA	0
rs181874957	T	0	NA	NA	NA	NA	NA	0	NA	NA	NA	0	NA	0	0	NA	0
rs574957059	A	0	NA	NA	NA	NA	NA	0	NA	NA	NA	0	NA	0	0	NA	0
rs185018051	C	0	NA	NA	NA	NA	NA	0	NA	NA	NA	0	NA	0	0.005051	NA	0
rs564806692	A	0	NA	NA	NA	NA	NA	0	NA	NA	NA	0.005814	NA	0	0	NA	0
rs556079659	C	0	NA	NA	NA	NA	NA	0	NA	NA	NA	0	NA	0	0	NA	0
rs79617378	C	0.005208	0	0	0	0	0	0	0	0	0.006246	0	0	0	0.02525	0	0
rs189436980	G	0	NA	NA	NA	NA	NA	0	NA	NA	NA	0	NA	0	0	NA	0
rs187078345	A	0.02604	NA	NA	NA	NA	NA	0.03279	NA	NA	NA	0	NA	0	0	NA	0
rs534885992	A	0	NA	NA	NA	NA	NA	0	NA	NA	NA	0	NA	0	0	NA	0.004854
rs187143520	C	0	NA	NA	NA	NA	NA	0	NA	NA	NA	0	NA	0	0	NA	0
rs535174935	T	0	NA	NA	NA	NA	NA	0	NA	NA	NA	0	NA	0	0	NA	0
rs528681261	T	0	NA	NA	NA	NA	NA	0	NA	NA	NA	0	NA	0	0	NA	0
rs563352095	T	0	NA	NA	NA	NA	NA	0	NA	NA	NA	0	NA	0	0	NA	0
rs117688613	G	0	NA	NA	NA	NA	NA	0	NA	NA	NA	0	NA	0	0.005051	NA	0

SNP	Allele	ACB	Afro	Aimaras	Ancash	Arequipa	Ashaninkas	ASW	Awajun	Ayacucho	Bambui	BEB	Candoshi	CDX	CEU	Chachapoyas	CHB
rs550253934	G	0	NA	NA	NA	NA	NA	0	NA	NA	NA	0	NA	0	0	NA	0
rs181162134	C	0	NA	NA	NA	NA	NA	0	NA	NA	NA	0	NA	0	0	NA	0
rs192026800	A	0	NA	NA	NA	NA	NA	0	NA	NA	NA	0	NA	0	0	NA	0.004854
rs552303675	T	0	NA	NA	NA	NA	NA	0	NA	NA	NA	0	NA	0	0	NA	0
rs147147950	A	0	NA	NA	NA	NA	NA	0	NA	NA	NA	0	NA	0	0	NA	0
rs76314085	G	0	NA	NA	NA	NA	NA	0	NA	NA	NA	0	NA	0	0	NA	0
rs567504414	T	0	NA	NA	NA	NA	NA	0	NA	NA	NA	0	NA	0	0	NA	0
rs563853944	C	0.005208	NA	NA	NA	NA	NA	0	NA	NA	NA	0	NA	0	0.005051	NA	0
rs578103765	T	0	NA	NA	NA	NA	NA	0	NA	NA	NA	0	NA	0	0	NA	0
rs574238899	G	0	NA	NA	NA	NA	NA	0	NA	NA	NA	0	NA	0	0	NA	0
rs2838040	G	0.4583	NA	NA	NA	NA	NA	0.459	NA	NA	NA	0.3023	NA	0.1882	0.2525	NA	0.335
rs537876986	A	0	NA	NA	NA	NA	NA	0	NA	NA	NA	0	NA	0	0.005051	NA	0
rs550222907	T	0	NA	NA	NA	NA	NA	0	NA	NA	NA	0	NA	0	0	NA	0
rs556260609	A	0	NA	NA	NA	NA	NA	0	NA	NA	NA	0	NA	0	0	NA	0
rs142296178	A	0	NA	NA	NA	NA	NA	0	NA	NA	NA	0	NA	0	0	NA	0
rs577285910	A	0	NA	NA	NA	NA	NA	0	NA	NA	NA	0	NA	0	0	NA	0
rs566925007	A	0	NA	NA	NA	NA	NA	0	NA	NA	NA	0	NA	0	0	NA	0
rs457909	G	0	NA	NA	NA	NA	NA	0	NA	NA	NA	0.005814	NA	0.04301	0	NA	0.01456
rs562479511	T	0	NA	NA	NA	NA	NA	0	NA	NA	NA	0	NA	0	0	NA	0
rs2298663	T	0.4531	NA	NA	NA	NA	NA	0.4918	NA	NA	NA	0.5872	NA	0.2473	0.7475	NA	0.3107
rs2070787	G	0.3073	NA	NA	NA	NA	NA	0.2705	NA	NA	NA	0.2558	NA	0.2419	0.298	NA	0.2961
rs573615979	C	0	NA	NA	NA	NA	NA	0	NA	NA	NA	0	NA	0	0	NA	0
rs542031508	C	0	NA	NA	NA	NA	NA	0	NA	NA	NA	0	NA	0	0	NA	0.004854
rs555600211	A	0	NA	NA	NA	NA	NA	0	NA	NA	NA	0	NA	0.005376	0	NA	0
rs528451616	A	0.01042	NA	NA	NA	NA	NA	0.01639	NA	NA	NA	0.005814	NA	0	0	NA	0
rs378501	G	0	NA	NA	NA	NA	NA	0	NA	NA	NA	0.005814	NA	0.172	0	NA	0.03883
rs140408843	A	0	NA	NA	NA	NA	NA	0	NA	NA	NA	0	NA	0	0	NA	0
rs186280517	C	0	NA	NA	NA	NA	NA	0	NA	NA	NA	0	NA	0	0	NA	0.004854
rs148561341	T	0.005208	NA	NA	NA	NA	NA	0	NA	NA	NA	0	NA	0	0	NA	0
rs568162250	A	0	NA	NA	NA	NA	NA	0	NA	NA	NA	0	NA	0	0	NA	0
rs561709263	C	0	NA	NA	NA	NA	NA	0	NA	NA	NA	0	NA	0	0	NA	0
rs569583276	A	0	NA	NA	NA	NA	NA	0	NA	NA	NA	0	NA	0	0	NA	0
rs572164476	A	0	NA	NA	NA	NA	NA	0	NA	NA	NA	0	NA	0	0	NA	0
rs560575827	T	0	NA	NA	NA	NA	NA	0	NA	NA	NA	0	NA	0	0	NA	0
rs111220492	G	0.224	NA	NA	NA	NA	NA	0.2541	NA	NA	NA	0.3314	NA	0.01075	0.5051	NA	0.004854
rs182231135	C	0	NA	NA	NA	NA	NA	0	NA	NA	NA	0	NA	0	0	NA	0
rs531884512	T	0.005208	NA	NA	NA	NA	NA	0	NA	NA	NA	0	NA	0	0	NA	0
rs573986970	A	0.005208	NA	NA	NA	NA	NA	0	NA	NA	NA	0	NA	0	0	NA	0
rs564705715	A	0	NA	NA	NA	NA	NA	0	NA	NA	NA	0	NA	0	0	NA	0
rs201661208	A	0	NA	NA	NA	NA	NA	0	NA	NA	NA	0	NA	0	0	NA	0.004854
rs530540353	C	0	NA	NA	NA	NA	NA	0	NA	NA	NA	0	NA	0.005376	0	NA	0
rs148155433	A	0	NA	NA	NA	NA	NA	0	NA	NA	NA	0	NA	0	0	NA	0
rs558834486	G	0	NA	NA	NA	NA	NA	0	NA	NA	NA	0	NA	0	0	NA	0
rs186275240	A	0	NA	NA	NA	NA	NA	0	NA	NA	NA	0	NA	0	0	NA	0
rs531418415	A	0	NA	NA	NA	NA	NA	0	NA	NA	NA	0	NA	0	0	NA	0

SNP	Allele	ACB	Afro	Aimaras	Ancash	Arequipa	Ashaninkas	ASW	Awajun	Ayacucho	Bambui	BEB	Candoshi	CDX	CEU	Chachapoyas	CHB
rs531609155	G	0	NA	NA	NA	NA	NA	0	NA	NA	NA	0	NA	0	0	NA	0
rs376752614	A	0	NA	NA	NA	NA	NA	0	NA	NA	NA	0.005814	NA	0	0	NA	0
rs139144487	A	0	NA	NA	NA	NA	NA	0	NA	NA	NA	0	NA	0	0	NA	0
rs531754656	A	0	NA	NA	NA	NA	NA	0	NA	NA	NA	0	NA	0	0	NA	0
rs554702767	A	0	NA	NA	NA	NA	NA	0.008197	NA	NA	NA	0	NA	0	0	NA	0
rs456016	T	0.01562	NA	NA	NA	NA	NA	0.02459	NA	NA	NA	0.0814	NA	0.4032	0.02525	NA	0.267
rs144364265	T	0	NA	NA	NA	NA	NA	0	NA	NA	NA	0	NA	0	0	NA	0
rs113773731	C	0.03646	NA	NA	NA	NA	NA	0.008197	NA	NA	NA	0	NA	0	0	NA	0
rs142261174	T	0.005208	NA	NA	NA	NA	NA	0.008197	NA	NA	NA	0	NA	0	0	NA	0
rs563595464	T	0	NA	NA	NA	NA	NA	0.008197	NA	NA	NA	0	NA	0	0	NA	0
rs531013422	T	0	NA	NA	NA	NA	NA	0	NA	NA	NA	0	NA	0	0	NA	0
rs569886879	G	0	NA	NA	NA	NA	NA	0	NA	NA	NA	0	NA	0	0	NA	0
rs541977145	G	0.005208	NA	NA	NA	NA	NA	0	NA	NA	NA	0	NA	0	0	NA	0
rs190906841	T	0	NA	NA	NA	NA	NA	0	NA	NA	NA	0	NA	0	0	NA	0.004854
rs139222305	T	0	NA	NA	NA	NA	NA	0	NA	NA	NA	0.005814	NA	0	0.0202	NA	0
rs527242422	T	0	NA	NA	NA	NA	NA	0	NA	NA	NA	0	NA	0	0	NA	0
rs364289	A	0.3958	NA	NA	NA	NA	NA	0.3361	NA	NA	NA	0.2384	NA	0.3065	0.2273	NA	0.3252
rs371053759	T	0.005208	NA	NA	NA	NA	NA	0	NA	NA	NA	0	NA	0	0.005051	NA	0
rs146116431	T	0	NA	NA	NA	NA	NA	0	NA	NA	NA	0	NA	0	0	NA	0
rs928871	C	0.3958	NA	NA	NA	NA	NA	0.377	NA	NA	NA	0.6919	NA	0.8065	0.7374	NA	0.6456
rs532335955	T	0	NA	NA	NA	NA	NA	0	NA	NA	NA	0	NA	0	0	NA	0
rs113928389	A	0.02604	NA	NA	NA	NA	NA	0.03279	NA	NA	NA	0	NA	0	0	NA	0
rs147138431	A	0.01562	NA	NA	NA	NA	NA	0.008197	NA	NA	NA	0	NA	0	0.005051	NA	0
rs4283504	T	0.04167	NA	NA	NA	NA	NA	0.04098	NA	NA	NA	0.1221	NA	0.2312	0.09596	NA	0.233
rs145297649	G	0.005208	NA	NA	NA	NA	NA	0	NA	NA	NA	0	NA	0	0	NA	0
rs146957681	T	0	NA	NA	NA	NA	NA	0	NA	NA	NA	0	NA	0	0	NA	0
rs562177468	G	0	NA	NA	NA	NA	NA	0	NA	NA	NA	0	NA	0	0	NA	0
rs576030223	A	0	NA	NA	NA	NA	NA	0	NA	NA	NA	0	NA	0	0	NA	0
rs190385097	A	0	NA	NA	NA	NA	NA	0	NA	NA	NA	0	NA	0	0	NA	0
rs7283324	T	0.3802	NA	NA	NA	NA	NA	0.4344	NA	NA	NA	0.3256	NA	0.3495	0.2071	NA	0.4272
rs118108663	T	0	NA	NA	NA	NA	NA	0	NA	NA	NA	0	NA	0	0	NA	0.01942
rs61735789	A	0	NA	NA	NA	NA	NA	0	NA	NA	NA	0	NA	0	0.01515	NA	0
rs113564116	C	0	NA	NA	NA	NA	NA	0	NA	NA	NA	0	NA	0	0.0202	NA	0.004854
rs2298659	A	0.2031	NA	NA	NA	NA	NA	0.2213	NA	NA	NA	0.25	NA	0.172	0.2172	NA	0.2961
rs150581606	A	0	NA	NA	NA	NA	NA	0	NA	NA	NA	0	NA	0	0	NA	0
rs116170128	C	0.1198	NA	NA	NA	NA	NA	0.08197	NA	NA	NA	0.005814	NA	0.005376	0.0404	NA	0
rs143291395	A	0	NA	NA	NA	NA	NA	0	NA	NA	NA	0	NA	0	0	NA	0
rs112753686	T	0.005208	NA	NA	NA	NA	NA	0.008197	NA	NA	NA	0	NA	0	0	NA	0
rs75603675	A	0.3021	NA	NA	NA	NA	NA	0.2951	NA	NA	NA	0.2267	NA	0.03763	0.399	NA	0.009709
rs553516093	A	0	NA	NA	NA	NA	NA	0	NA	NA	NA	0	NA	0	0	NA	0
rs143460343	A	0	NA	NA	NA	NA	NA	0	NA	NA	NA	0	NA	0	0	NA	0
rs184365262	T	0	NA	NA	NA	NA	NA	0	NA	NA	NA	0	NA	0	0	NA	0
rs571074719	T	0.01042	NA	NA	NA	NA	NA	0	NA	NA	NA	0	NA	0	0	NA	0
rs115975538	A	0.09375	NA	NA	NA	NA	NA	0.03279	NA	NA	NA	0.005814	NA	0.005376	0	NA	0
rs147465180	A	0	NA	NA	NA	NA	NA	0	NA	NA	NA	0	NA	0	0	NA	0

SNP	Allele	ACB	Afro	Aimaras	Ancash	Arequipa	Ashaninkas	ASW	Awajun	Ayacucho	Bambui	BEB	Candoshi	CDX	CEU	Chachapoyas	CHB
rs548349124	G	0	NA	NA	NA	NA	NA	0.008197	NA	NA	NA	0	NA	0	0	NA	0
rs139467735	T	0.07292	NA	NA	NA	NA	NA	0.03279	NA	NA	NA	0.005814	NA	0.005376	0	NA	0
rs576042280	T	0	NA	NA	NA	NA	NA	0	NA	NA	NA	0	NA	0	0	NA	0
rs544083065	A	0	NA	NA	NA	NA	NA	0	NA	NA	NA	0	NA	0	0	NA	0
rs551244742	A	0	NA	NA	NA	NA	NA	0	NA	NA	NA	0	NA	0	0	NA	0
rs149076631	T	0.005208	NA	NA	NA	NA	NA	0	NA	NA	NA	0	NA	0	0	NA	0
rs35041537	T	0.1667	0.1383	0.1562	0.1389	0.1304	0.1143	0.1803	0.1957	0.1286	0.3838	0.3779	0.0625	0.01075	0.4798	0.175	0.004854
rs56218846	A	0.3281	NA	NA	NA	NA	NA	0.2541	NA	NA	NA	0.2151	NA	0.03763	0.3889	NA	0.009709
rs527693716	T	0	NA	NA	NA	NA	NA	0	NA	NA	NA	0	NA	0.01075	0	NA	0
rs189298425	A	0	NA	NA	NA	NA	NA	0	NA	NA	NA	0	NA	0	0	NA	0
rs562750287	A	0	NA	NA	NA	NA	NA	0	NA	NA	NA	0	NA	0	0	NA	0
rs570413089	A	0	NA	NA	NA	NA	NA	0	NA	NA	NA	0	NA	0	0	NA	0
rs111383922	T	0.005208	NA	NA	NA	NA	NA	0	NA	NA	NA	0	NA	0	0	NA	0
rs559699558	A	0	NA	NA	NA	NA	NA	0	NA	NA	NA	0	NA	0	0	NA	0
rs542060428	T	0	NA	NA	NA	NA	NA	0	NA	NA	NA	0	NA	0.005376	0	NA	0
rs565142477	T	0	NA	NA	NA	NA	NA	0	NA	NA	NA	0	NA	0.005376	0	NA	0
rs548742822	C	0	NA	NA	NA	NA	NA	0	NA	NA	NA	0	NA	0	0	NA	0
rs148304071	T	0	NA	NA	NA	NA	NA	0	NA	NA	NA	0	NA	0	0	NA	0.03883
rs455045	C	0.2344	NA	NA	NA	NA	NA	0.3689	NA	NA	NA	0.3372	NA	0.3387	0.5202	NA	0.3738
rs570441750	G	0	NA	NA	NA	NA	NA	0	NA	NA	NA	0	NA	0	0	NA	0
rs9974589	A	0.3021	NA	NA	NA	NA	NA	0.3033	NA	NA	NA	0.4186	NA	0.4086	0.4899	NA	0.2767
rs2104810	A	0.4688	NA	NA	NA	NA	NA	0.5	NA	NA	NA	0.5872	NA	0.2473	0.7475	NA	0.3058
rs147711290	C	NA	NA	NA	NA	NA	NA	NA	NA	NA	NA	NA	NA	NA	NA	NA	NA
rs555056677	A	0	NA	NA	NA	NA	NA	0	NA	NA	NA	0	NA	0	0	NA	0
rs9974933	G	0.2188	NA	NA	NA	NA	NA	0.2131	NA	NA	NA	0.2209	NA	0.2419	0.2374	NA	0.3058
rs544221548	A	0	NA	NA	NA	NA	NA	0	NA	NA	NA	0	NA	0	0	NA	0
rs147827602	T	0	NA	NA	NA	NA	NA	0.008197	NA	NA	NA	0	NA	0	0	NA	0
rs462321	T	0.3906	NA	NA	NA	NA	NA	0.4344	NA	NA	NA	0.4012	NA	0.7527	0.197	NA	0.6942
rs142659685	T	0	NA	NA	NA	NA	NA	0	NA	NA	NA	0.01163	NA	0.07527	0	NA	0.0534
rs150838246	C	0	NA	NA	NA	NA	NA	0	NA	NA	NA	0	NA	0	0	NA	0.004854
rs112132031	C	0.1771	NA	NA	NA	NA	NA	0.1393	NA	NA	NA	0.08721	NA	0.4032	0.02525	NA	0.267
rs9974995	T	0.2188	0.3587	0.06667	0.1111	0.2391	0.05714	0.2131	0.1739	0.2571	0.2993	0.2209	0.3125	0.2419	0.2323	0.175	0.3058
rs557494549	TCAGGGAGTGCAGAGCAGGAGGGAC	0	NA	NA	NA	NA	NA	0	NA	NA	NA	0	NA	0	0	NA	0
rs186929947	G	0	NA	NA	NA	NA	NA	0	NA	NA	NA	0	NA	0	0.005051	NA	0
rs148509204	T	0.01562	NA	NA	NA	NA	NA	0	NA	NA	NA	0	NA	0	0	NA	0
rs202094412	A	0	NA	NA	NA	NA	NA	0	NA	NA	NA	0	NA	0	0	NA	0
rs575981127	A	0	NA	NA	NA	NA	NA	0	NA	NA	NA	0.01163	NA	0	0	NA	0
rs546775580	T	0	NA	NA	NA	NA	NA	0	NA	NA	NA	0	NA	0	0	NA	0
rs185188053	T	0	NA	NA	NA	NA	NA	0	NA	NA	NA	0	NA	0	0	NA	0
rs577381045	A	0	NA	NA	NA	NA	NA	0	NA	NA	NA	0	NA	0	0	NA	0
rs62217525	T	0.005208	NA	NA	NA	NA	NA	0.008197	NA	NA	NA	0	NA	0	0.04545	NA	0
rs561043908	A	0.005208	NA	NA	NA	NA	NA	0	NA	NA	NA	0	NA	0	0	NA	0
rs367879274	T	0	NA	NA	NA	NA	NA	0	NA	NA	NA	0	NA	0	0	NA	0
rs568167564	G	0	NA	NA	NA	NA	NA	0	NA	NA	NA	0	NA	0	0	NA	0
rs563633617	A	0	NA	NA	NA	NA	NA	0	NA	NA	NA	0	NA	0	0	NA	0

SNP	Allele	ACB	Afro	Aimaras	Ancash	Arequipa	Ashaninkas	ASW	Awajun	Ayacucho	Bambui	BEB	Candoshi	CDX	CEU	Chachapoyas	CHB
rs537935645	T	0	NA	NA	NA	NA	NA	0	NA	NA	NA	0	NA	0	0	NA	0
rs9985159	C	0.4792	0.6064	0.9375	0.6944	0.8043	0.8286	0.5246	0.7391	0.6857	0.7967	0.6802	0.6562	0.6505	0.7778	0.75	0.5777
rs61735795	A	NA	NA	NA	NA	NA	NA	NA	NA	NA	NA	NA	NA	NA	NA	NA	NA
rs557712450	C	0	NA	NA	NA	NA	NA	0	NA	NA	NA	0	NA	0	0	NA	0
rs139305247	T	0	NA	NA	NA	NA	NA	0.01639	NA	NA	NA	0	NA	0	0.0101	NA	0
rs548653178	A	0	NA	NA	NA	NA	NA	0	NA	NA	NA	0	NA	0	0	NA	0
rs146385718	C	0	NA	NA	NA	NA	NA	0	NA	NA	NA	0	NA	0	0	NA	0
rs28360562	C	0.02083	NA	NA	NA	NA	NA	0.04098	NA	NA	NA	0.05233	NA	0	0.1061	NA	0
rs9983330	G	0.2812	NA	NA	NA	NA	NA	0.2951	NA	NA	NA	0.2965	NA	0.3333	0.2323	NA	0.4175
rs539864066	G	0	NA	NA	NA	NA	NA	0.008197	NA	NA	NA	0.005814	NA	0	0	NA	0
rs562014334	A	0	NA	NA	NA	NA	NA	0	NA	NA	NA	0	NA	0	0	NA	0
rs118134524	A	0	NA	NA	NA	NA	NA	0	NA	NA	NA	0	NA	0.03226	0	NA	0
rs574811036	A	0	NA	NA	NA	NA	NA	0	NA	NA	NA	0	NA	0	0	NA	0
rs115720411	A	0.1927	NA	NA	NA	NA	NA	0.1393	NA	NA	NA	0.005814	NA	0	0	NA	0
rs368812287	T	0	NA	NA	NA	NA	NA	0	NA	NA	NA	0	NA	0	0	NA	0
rs2410428	T	0.09896	NA	NA	NA	NA	NA	0.1311	NA	NA	NA	0.2209	NA	0.2419	0.2323	NA	0.3107
rs577480005	A	0.005208	NA	NA	NA	NA	NA	0.008197	NA	NA	NA	0	NA	0	0	NA	0
rs543815381	T	0	NA	NA	NA	NA	NA	0	NA	NA	NA	0	NA	0	0	NA	0
rs142834250	G	0	NA	NA	NA	NA	NA	0	NA	NA	NA	0	NA	0	0	NA	0
rs576292358	A	0	NA	NA	NA	NA	NA	0	NA	NA	NA	0	NA	0	0	NA	0
rs141603473	A	0.01562	NA	NA	NA	NA	NA	0.008197	NA	NA	NA	0	NA	0.005376	0	NA	0
rs143523726	G	0	NA	NA	NA	NA	NA	0	NA	NA	NA	0	NA	0	0	NA	0
rs375408	G	0.01562	NA	NA	NA	NA	NA	0.02459	NA	NA	NA	0.06977	NA	0.414	0.0303	NA	0.267
rs370347248	C	0.005208	NA	NA	NA	NA	NA	0	NA	NA	NA	0	NA	0	0	NA	0
rs539123545	C	0	NA	NA	NA	NA	NA	0	NA	NA	NA	0.005814	NA	0	0	NA	0
rs4303794	C	0.3021	NA	NA	NA	NA	NA	0.3033	NA	NA	NA	0.2326	NA	0.04301	0.4091	NA	0.009709
rs151035593	T	0.01042	NA	NA	NA	NA	NA	0.01639	NA	NA	NA	0	NA	0	0	NA	0
rs370896169	C	0	NA	NA	NA	NA	NA	0	NA	NA	NA	0	NA	0	0	NA	0.004854
rs575167859	T	0	NA	NA	NA	NA	NA	0	NA	NA	NA	0	NA	0	0	NA	0
rs35050484	A	0.005208	NA	NA	NA	NA	NA	0.01639	NA	NA	NA	0	NA	0	0.01515	NA	0
rs56136037	T	0	NA	NA	NA	NA	NA	0.008197	NA	NA	NA	0.01163	NA	0	0.0303	NA	0
rs539767738	A	0	NA	NA	NA	NA	NA	0	NA	NA	NA	0.005814	NA	0	0	NA	0
rs533229030	G	0	NA	NA	NA	NA	NA	0	NA	NA	NA	0	NA	0	0	NA	0
rs465576	C	0.151	NA	NA	NA	NA	NA	0.1066	NA	NA	NA	0.08721	NA	0.4032	0.02525	NA	0.267
rs570494222	T	0	NA	NA	NA	NA	NA	0	NA	NA	NA	0	NA	0	0	NA	0
rs4818240	T	0.1875	NA	NA	NA	NA	NA	0.1885	NA	NA	NA	0.0814	NA	0.4516	0.0303	NA	0.2767
rs9636988	C	0.2188	0.3723	0.0625	0.1111	0.2391	0.05714	0.2131	0.1739	0.2571	0.3034	0.2209	0.3125	0.2419	0.2424	0.1875	0.3058
rs541887371	T	0	NA	NA	NA	NA	NA	0	NA	NA	NA	0	NA	0	0	NA	0
rs537787922	A	0	NA	NA	NA	NA	NA	0	NA	NA	NA	0	NA	0	0	NA	0.004854
rs139829932	T	0.01042	NA	NA	NA	NA	NA	0	NA	NA	NA	0	NA	0	0	NA	0
rs200615061	C	0	NA	NA	NA	NA	NA	0	NA	NA	NA	0	NA	0	0.0101	NA	0
rs3787947	T	0.4062	NA	NA	NA	NA	NA	0.4508	NA	NA	NA	0.3023	NA	0.1935	0.2626	NA	0.3544
rs402197	T	0.01562	NA	NA	NA	NA	NA	0.02459	NA	NA	NA	0.06977	NA	0.414	0.0303	NA	0.267
rs562203987	T	0	NA	NA	NA	NA	NA	0	NA	NA	NA	0	NA	0	0	NA	0
rs141764184	A	0	NA	NA	NA	NA	NA	0	NA	NA	NA	0.005814	NA	0.03763	0	NA	0.004854

SNP	Allele	ACB	Afro	Aimaras	Ancash	Arequipa	Ashaninkas	ASW	Awajun	Ayacucho	Bambui	BEB	Candoshi	CDX	CEU	Chachapoyas	CHB
rs116577479	A	0.03646	NA	NA	NA	NA	NA	0.05738	NA	NA	NA	0	NA	0	0	NA	0
rs118133613	A	0	NA	NA	NA	NA	NA	0.01639	NA	NA	NA	0	NA	0	0.005051	NA	0
rs118108194	G	0	NA	NA	NA	NA	NA	0	NA	NA	NA	0	NA	0	0.0101	NA	0
rs138763189	A	0.005208	NA	NA	NA	NA	NA	0	NA	NA	NA	0	NA	0	0	NA	0
rs138365638	TGG	0.03125	NA	NA	NA	NA	NA	0.04098	NA	NA	NA	0.06977	NA	0.4086	0.0303	NA	0.2718
rs551442751	T	0	NA	NA	NA	NA	NA	0	NA	NA	NA	0	NA	0.005376	0	NA	0
rs539019775	T	0	NA	NA	NA	NA	NA	0	NA	NA	NA	0	NA	0	0	NA	0
rs112657409	T	0.07812	0.03191	0	0	0	0	0.07377	0	0	0.02184	0.01744	0	0.1237	0	0	0.06311
rs577689706	A	0	NA	NA	NA	NA	NA	0	NA	NA	NA	0	NA	0	0	NA	0
rs147349930	T	0.02604	NA	NA	NA	NA	NA	0.03279	NA	NA	NA	0	NA	0	0	NA	0
rs557531433	T	0	NA	NA	NA	NA	NA	0	NA	NA	NA	0	NA	0	0	NA	0
rs144359794	T	0	NA	NA	NA	NA	NA	0	NA	NA	NA	0	NA	0	0	NA	0
rs187426170	T	0	NA	NA	NA	NA	NA	0.008197	NA	NA	NA	0	NA	0	0	NA	0
rs199575615	G	0	NA	NA	NA	NA	NA	0	NA	NA	NA	0	NA	0.005376	0	NA	0.004854
rs529737187	A	0	NA	NA	NA	NA	NA	0	NA	NA	NA	0	NA	0	0	NA	0
rs576447019	A	0	NA	NA	NA	NA	NA	0	NA	NA	NA	0	NA	0	0	NA	0
rs145900878	T	0.02083	NA	NA	NA	NA	NA	0.04918	NA	NA	NA	0	NA	0	0	NA	0
rs9977234	T	0.09375	NA	NA	NA	NA	NA	0.1148	NA	NA	NA	0.2151	NA	0.2419	0.2172	NA	0.3107
rs540024706	C	0	NA	NA	NA	NA	NA	0	NA	NA	NA	0.005814	NA	0	0	NA	0
rs528640390	G	0.005208	NA	NA	NA	NA	NA	0	NA	NA	NA	0	NA	0	0	NA	0
rs546337899	G	0	NA	NA	NA	NA	NA	0.008197	NA	NA	NA	0	NA	0	0	NA	0.01456
rs141323355	A	0	NA	NA	NA	NA	NA	0	NA	NA	NA	0	NA	0	0	NA	0
rs386519	G	0	NA	NA	NA	NA	NA	0.008197	NA	NA	NA	0	NA	0	0	NA	0
rs561327241	T	0	NA	NA	NA	NA	NA	0	NA	NA	NA	0.02326	NA	0	0	NA	0
rs572351113	A	0	NA	NA	NA	NA	NA	0	NA	NA	NA	0	NA	0	0	NA	0
rs189425119	A	0	NA	NA	NA	NA	NA	0	NA	NA	NA	0	NA	0	0	NA	0.004854
rs566485026	G	0	NA	NA	NA	NA	NA	0	NA	NA	NA	0	NA	0	0	NA	0
rs2298665	C	0	NA	NA	NA	NA	NA	0	NA	NA	NA	0	NA	0	0	NA	0.004854
rs142769034	T	0	NA	NA	NA	NA	NA	0	NA	NA	NA	0.005814	NA	0	0.02525	NA	0
rs74564819	C	0	NA	NA	NA	NA	NA	0	NA	NA	NA	0	NA	0.01075	0	NA	0
rs2298857	A	0.3646	0.3936	0.0625	0.09722	0.2174	0.05714	0.3197	0.1304	0.2571	0.3027	0.2267	0.25	0.2527	0.2222	0.1625	0.3107
rs545232590	T	0	NA	NA	NA	NA	NA	0	NA	NA	NA	0	NA	0	0	NA	0
rs529218967	G	0	NA	NA	NA	NA	NA	0	NA	NA	NA	0	NA	0	0	NA	0
rs528337775	T	0	NA	NA	NA	NA	NA	0	NA	NA	NA	0	NA	0	0	NA	0
rs563980567	A	0	NA	NA	NA	NA	NA	0	NA	NA	NA	0	NA	0	0	NA	0
rs534913861	T	0	NA	NA	NA	NA	NA	0	NA	NA	NA	0	NA	0	0	NA	0
rs535348902	A	0	NA	NA	NA	NA	NA	0	NA	NA	NA	0	NA	0	0	NA	0
rs555405939	C	0	NA	NA	NA	NA	NA	0	NA	NA	NA	0	NA	0	0	NA	0
rs187037274	A	0.02083	NA	NA	NA	NA	NA	0.02459	NA	NA	NA	0	NA	0	0	NA	0
rs141027872	T	0.005208	NA	NA	NA	NA	NA	0	NA	NA	NA	0	NA	0	0	NA	0
rs191394761	T	0	NA	NA	NA	NA	NA	0	NA	NA	NA	0	NA	0	0	NA	0
rs540964406	G	0	NA	NA	NA	NA	NA	0	NA	NA	NA	0	NA	0	0	NA	0
rs544440280	A	0	NA	NA	NA	NA	NA	0	NA	NA	NA	0	NA	0.005376	0	NA	0
rs146845793	C	0	NA	NA	NA	NA	NA	0	NA	NA	NA	0	NA	0	0	NA	0
rs372665499	T	0	NA	NA	NA	NA	NA	0	NA	NA	NA	0	NA	0	0	NA	0

SNP	Allele	ACB	Afro	Aimaras	Ancash	Arequipa	Ashaninkas	ASW	Awajun	Ayacucho	Bambui	BEB	Candoshi	CDX	CEU	Chachapoyas	CHB
rs538276300	A	0	NA	NA	NA	NA	NA	0	NA	NA	NA	0	NA	0	0	NA	0
rs113562865	T	0.1406	NA	NA	NA	NA	NA	0.08197	NA	NA	NA	0.005814	NA	0	0	NA	0
rs571417292	A	0	NA	NA	NA	NA	NA	0	NA	NA	NA	0	NA	0	0	NA	0
rs542865477	A	0	NA	NA	NA	NA	NA	0	NA	NA	NA	0	NA	0	0	NA	0
rs544110139	G	0	NA	NA	NA	NA	NA	0	NA	NA	NA	0	NA	0	0	NA	0
rs142914234	C	0	NA	NA	NA	NA	NA	0	NA	NA	NA	0	NA	0	0	NA	0
rs114549926	A	0.03125	NA	NA	NA	NA	NA	0.03279	NA	NA	NA	0	NA	0	0	NA	0
rs549289994	C	0	NA	NA	NA	NA	NA	0	NA	NA	NA	0	NA	0	0	NA	0
rs555327583	A	0	NA	NA	NA	NA	NA	0	NA	NA	NA	0	NA	0	0	NA	0
rs111220533	G	0.2604	NA	NA	NA	NA	NA	0.3033	NA	NA	NA	0.4128	NA	0.2419	0.4646	NA	0.3058
rs553400003	A	0	NA	NA	NA	NA	NA	0	NA	NA	NA	0.005814	NA	0	0	NA	0
rs530306757	A	0	NA	NA	NA	NA	NA	0	NA	NA	NA	0	NA	0	0	NA	0
rs561412657	G	0	NA	NA	NA	NA	NA	0	NA	NA	NA	0	NA	0	0	NA	0
rs565690211	G	0.005208	NA	NA	NA	NA	NA	0	NA	NA	NA	0	NA	0	0	NA	0
rs137929897	T	0	NA	NA	NA	NA	NA	0	NA	NA	NA	0	NA	0	0	NA	0
rs144458055	A	0.005376	NA	NA	NA	NA	NA	0.01639	NA	NA	NA	0.005814	NA	0	0	NA	0.009709
rs141722242	A	0	NA	NA	NA	NA	NA	0	NA	NA	NA	0	NA	0	0	NA	0
rs565720190	A	0	NA	NA	NA	NA	NA	0	NA	NA	NA	0	NA	0	0	NA	0
rs530051850	C	0	NA	NA	NA	NA	NA	0	NA	NA	NA	0	NA	0	0	NA	0
rs115967323	T	0	NA	NA	NA	NA	NA	0	NA	NA	NA	0	NA	0	0	NA	0.004854

Table S4-A – TMPRSS2 allele frequencies continuation. Bold = functionally relevant SNPs found in our databases; NA = missing data.

SNP	Allele	Chileans	Chopccas	CHS	CLM	Cusco	ESN	FIN	GBR	GIH	GWD	IBS	Iquitos	ITU	Jacarus	JPT	KHV
**rs456298**	**T**	**0.2778**	**NA**	**0.69**	**0.31**	**NA**	**0.41**	**0.24**	**0.14**	**0.21**	**0.39**	**0.11**	**NA**	**0.34**	**NA**	**0.53**	**0.71**
**rs12329760**	**T**	**0.5**	**0**	**0.3762**	**0.2021**	**0.01389**	**0.2626**	**0.3636**	**0.2088**	**0.1408**	**0.3097**	**0.1776**	**0.09615**	**0.1863**	**0.08824**	**0.3942**	**0.3232**
**rs2276205**	**G**	**NA**	**0**	**0.181**	**0.04787**	**0.01389**	**0.1263**	**0.1616**	**0.06593**	**0.01456**	**0.1372**	**0.06542**	**0**	**0.04412**	**0.02941**	**0.2308**	**0.1313**
**rs383510**	**T**	**0.3571**	**NA**	**0.3762**	**0.367**	**NA**	**0.3687**	**0.4242**	**0.5275**	**0.5**	**0.3186**	**0.5047**	**NA**	**0.4853**	**NA**	**0.2933**	**0.4242**
**rs2070788**	**G**	**0.4**	**0.7647**	**0.3762**	**0.4309**	**0.6389**	**0.3081**	**0.4495**	**0.4451**	**0.4806**	**0.208**	**0.5**	**0.6346**	**0.5098**	**0.7941**	**0.2981**	**0.4293**
rs149270377	A	NA	NA	0	0	NA	0.005051	0	0	0	0.00885	0	NA	0	NA	0	0
rs75833467	A	NA	NA	0	0.05319	NA	0	0.0303	0.08242	0.04369	0.004425	0.05607	NA	0.004902	NA	0	0
rs569429288	A	NA	NA	0	0	NA	0	0	0	0	0	0	NA	0	NA	0	0
rs554852594	C	NA	NA	0	0	NA	0	0	0	0	0	0	NA	0	NA	0	0
rs537997349	G	NA	NA	0	0.01064	NA	0.0101	0.005051	0	0	0.04867	0.004673	NA	0.004902	NA	0.004808	0.01515
rs569432639	C	NA	NA	0	0	NA	0	0	0	0	0	0	NA	0	NA	0	0
rs535019926	C	NA	NA	0	0	NA	0.005051	0	0	0	0	0	NA	0	NA	0	0
rs368646465	T	NA	NA	0	0	NA	0	0	0	0	0	0	NA	0	NA	0	0.0101
rs9305744	A	0.5	NA	0.419	0.2447	NA	0.4091	0.3434	0.2143	0.2039	0.3938	0.1729	NA	0.2206	NA	0.4183	0.3586
rs148910500	T	NA	NA	0	0	NA	0	0	0	0	0	0	NA	0	NA	0	0
rs142710225	T	NA	NA	0	0	NA	0.005051	0	0	0	0.02655	0	NA	0	NA	0	0
rs368735421	T	NA	NA	0	0	NA	0	0	0	0	0	0	NA	0	NA	0	0
rs140458174	A	NA	NA	0.004762	0	NA	0	0	0	0	0	0	NA	0	NA	0.02885	0
rs532232636	T	NA	NA	0	0	NA	0	0	0	0	0	0.004673	NA	0	NA	0	0
rs381179	T	0	NA	0	0	NA	0	0	0	0	0	0	NA	0	NA	0	0.01515

SNP	Allele	Chileans	Chopccas	CHS	CLM	Cusco	ESN	FIN	GBR	GIH	GWD	IBS	Iquitos	ITU	Jacarus	JPT	KHV
rs572410855	A	NA	NA	0	0.005319	NA	0	0	0	0	0	0	NA	0	NA	0	0
rs111620846	A	NA	NA	0	0	NA	0.0202	0	0	0	0.0177	0	NA	0	NA	0	0
rs377496737	T	NA	NA	0	0	NA	0	0	0	0	0.02212	0	NA	0	NA	0	0
rs533552237	A	NA	NA	0	0	NA	0	0	0	0	0	0	NA	0	NA	0	0
rs76315847	A	NA	0	0	0	0	0.0101	0	0	0	0	0	0	0	0	0	0
rs377348479	A	NA	NA	0	0	NA	0	0	0	0	0	0	NA	0	NA	0	0.005051
rs180818774	C	NA	NA	0	0	NA	0.005051	0	0	0	0	0	NA	0	NA	0	0
rs462471	A	0.2778	0.7353	0.6714	0.3138	0.5556	0.4141	0.2374	0.1374	0.2136	0.3894	0.1121	0.5577	0.3431	0.6765	0.5288	0.7071
rs554357472	A	NA	NA	0	0	NA	0	0	0.01099	0	0	0	NA	0	NA	0	0
rs117652812	T	NA	NA	0.0381	0	NA	0	0.005051	0	0	0	0	NA	0	NA	0.03365	0.0101
rs56097233	CAG	NA	NA	0.7476	0.4202	NA	0.4242	0.4394	0.2308	0.2767	0.4558	0.229	NA	0.3333	NA	0.6827	0.7071
rs573178525	A	NA	NA	0	0	NA	0	0	0	0	0	0	NA	0	NA	0	0
rs186168224	T	NA	NA	0	0	NA	0	0	0	0	0	0	NA	0	NA	0	0
rs551103429	T	NA	NA	0	0	NA	0	0	0	0	0	0	NA	0	NA	0	0
rs462574	A	0.3	0.7353	0.6333	0.2074	0.5556	0.1869	0.04545	0.01648	0.1748	0.1858	0.01869	0.4615	0.2843	0.6176	0.4712	0.6818
rs543681861	C	NA	NA	0	0	NA	0	0	0	0	0.02212	0.004673	NA	0	NA	0	0
rs557632131	T	NA	NA	0.009524	0	NA	0.005051	0	0	0	0	0	NA	0	NA	0	0
rs186425543	G	NA	NA	0.004762	0	NA	0	0	0	0	0	0	NA	0	NA	0	0
rs542228195	C	NA	NA	0	0.01064	NA	0	0	0	0	0	0	NA	0	NA	0	0
rs144052153	G	NA	NA	0	0	NA	0	0	0	0	0	0	NA	0	NA	0.004808	0
rs374377315	T	NA	NA	0	0	NA	0	0	0	0	0	0	NA	0	NA	0	0
rs73357664	A	NA	NA	0	0	NA	0.0404	0	0	0	0.04425	0	NA	0	NA	0	0
rs373196115	A	NA	NA	0	0	NA	0	0	0.005495	0	0	0	NA	0	NA	0	0
rs181107522	A	NA	NA	0	0	NA	0.005051	0	0	0	0	0	NA	0	NA	0	0
rs569716669	C	NA	NA	0	0	NA	0	0	0	0	0	0	NA	0.004902	NA	0	0
rs535834505	G	NA	NA	0	0	NA	0	0	0	0	0	0	NA	0	NA	0	0
rs28707508	A	0.5	NA	0.01429	0.3138	NA	0.2828	0.3586	0.4011	0.1748	0.2434	0.3598	NA	0.1618	NA	0.01923	0.01515
rs458280	T	0.3889	NA	0.3762	0.1596	NA	0	0.04545	0.01099	0.06796	0	0.004673	NA	0.09314	NA	0.2933	0.4141
rs567208488	CAG	NA	NA	0	0	NA	0	0	0	0	0	0	NA	0.009804	NA	0	0
rs186510586	C	NA	NA	0	0	NA	0	0	0	0	0	0	NA	0	NA	0	0
rs140532244	C	NA	NA	0	0	NA	0	0	0	0	0	0	NA	0	NA	0	0
rs193019598	A	NA	NA	0	0	NA	0	0	0	0	0	0	NA	0	NA	0	0
rs533708336	A	NA	NA	0	0	NA	0	0	0	0	0	0	NA	0	NA	0	0
rs188198121	A	NA	NA	0	0	NA	0	0	0.005495	0	0	0.004673	NA	0	NA	0	0
rs142119028	T	NA	NA	0	0	NA	0.005051	0	0	0	0	0	NA	0	NA	0	0
rs141128014	G	NA	NA	0	0	NA	0	0	0	0	0	0	NA	0	NA	0	0
rs2838039	C	0.5	NA	0.3714	0.2234	NA	0.404	0.3939	0.2198	0.1456	0.4381	0.2056	NA	0.2206	NA	0.3942	0.3131
rs558683527	C	NA	NA	0	0	NA	0	0	0	0	0	0	NA	0	NA	0	0
rs545307151	G	NA	NA	0	0	NA	0	0	0	0	0	0	NA	0	NA	0	0
rs9976780	C	0.4375	NA	0.7905	0.3936	NA	0.4242	0.4394	0.2308	0.2087	0.4779	0.2103	NA	0.299	NA	0.6923	0.7525
rs553788077	A	NA	NA	0.004762	0	NA	0	0	0	0	0	0	NA	0	NA	0	0
rs539639766	C	NA	NA	0	0	NA	0	0	0	0	0	0.004673	NA	0	NA	0	0
rs543253134	A	NA	NA	0	0	NA	0	0	0	0	0.01327	0	NA	0	NA	0	0
rs79971314	A	NA	NA	0	0.01064	NA	0.2424	0	0	0	0.1504	0.009346	NA	0	NA	0	0
rs148719900	T	NA	NA	0	0	NA	0.005051	0	0	0	0.0177	0	NA	0	NA	0	0

SNP	Allele	Chileans	Chopccas	CHS	CLM	Cusco	ESN	FIN	GBR	GIH	GWD	IBS	Iquitos	ITU	Jacarus	JPT	KHV
rs144948620	A	NA	NA	0	0	NA	0	0.02525	0.02198	0.004854	0	0.004673	NA	0	NA	0	0
rs536525396	A	NA	NA	0	0	NA	0	0	0	0	0.00885	0	NA	0	NA	0	0
rs548341299	C	NA	NA	0	0	NA	0	0	0	0	0	0	NA	0	NA	0	0.005051
rs139816990	A	NA	NA	0	0	NA	0.0202	0	0	0	0.0177	0	NA	0	NA	0	0
rs536147878	A	NA	NA	0	0	NA	0	0	0	0	0	0	NA	0	NA	0	0
rs564793560	G	NA	NA	0	0	NA	0.005051	0	0	0.004854	0.004425	0	NA	0	NA	0	0
rs192011400	T	NA	NA	0	0	NA	0	0	0	0	0	0	NA	0	NA	0	0
rs531024324	A	NA	NA	0.004762	0	NA	0	0	0.005495	0.004854	0.02212	0	NA	0	NA	0.004808	0
rs192666663	A	NA	NA	0.009524	0	NA	0	0	0	0	0	0	NA	0	NA	0	0
rs138282462	T	NA	NA	0	0	NA	0	0	0	0	0	0	NA	0	NA	0	0
rs143109187	G	NA	NA	0.01905	0	NA	0	0	0	0	0	0	NA	0	NA	0.02404	0
rs150014829	A	NA	NA	0	0	NA	0	0	0	0	0	0	NA	0	NA	0.004808	0
rs34561135	A	NA	NA	0.004762	0.02128	NA	0	0.1212	0.05495	0.004854	0	0.02804	NA	0.01961	NA	0	0
rs11088551	G	0.5	NA	0.01905	0.3138	NA	0.303	0.404	0.4176	0.2087	0.2389	0.3832	NA	0.1765	NA	0.01442	0.0101
rs200169208	A	NA	NA	0	0	NA	0	0	0	0	0	0	NA	0	NA	0	0
rs566903241	C	NA	NA	0	0.005319	NA	0	0	0.005495	0	0	0	NA	0	NA	0	0
rs146132415	A	NA	NA	0	0	NA	0	0	0	0	0	0	NA	0	NA	0	0
rs61735791	T	NA	NA	0	0.005319	NA	0	0	0.005495	0	0	0	NA	0	NA	0.004808	0
rs571587918	C	NA	NA	0	0	NA	0	0	0	0	0.004425	0	NA	0	NA	0	0
rs78459594	C	NA	0	NA	NA	0	NA	NA	NA	NA	NA	NA	0	NA	0	NA	NA
rs389001	G	0.5909	NA	0.2714	0.1915	NA	0.3384	0.04545	0.01099	0.2524	0.3142	0.01402	NA	0.299	NA	0.3269	0.3485
rs200164183	T	NA	NA	0.004762	0	NA	0	0	0	0	0	0	NA	0	NA	0	0
rs548354821	C	NA	NA	0	0	NA	0	0	0	0	0	0	NA	0	NA	0	0
rs2187238	C	0.5	0.02941	0.05238	0.1649	0.2639	0.03535	0.1212	0.2253	0.1796	0.0531	0.2196	0.25	0.08824	0.08824	0.05769	0.005051
rs556456416	C	NA	NA	0	0	NA	0	0	0	0	0	0	NA	0	NA	0	0
rs181414852	G	NA	NA	0	0.005319	NA	0	0	0	0	0	0	NA	0	NA	0	0
rs553787982	A	NA	NA	0	0	NA	0	0	0	0	0	0	NA	0	NA	0	0
rs417888	A	0.4	NA	0.4381	0.4787	NA	0.1111	0.4394	0.5275	0.5	0.06195	0.4907	NA	0.4853	NA	0.3462	0.4747
rs189793246	G	NA	NA	0.009524	0	NA	0	0	0	0	0	0	NA	0	NA	0.004808	0.0101
rs556371948	T	NA	NA	0	0.005319	NA	0	0	0	0	0	0	NA	0	NA	0	0
rs546273967	T	NA	NA	0	0	NA	0	0	0.005495	0	0	0	NA	0	NA	0	0
rs76000363	A	0.5	NA	0.04762	0.1011	NA	0.08081	0.1919	0.1209	0.03883	0.08407	0.08879	NA	0.05882	NA	0.0625	0.02525
rs557639119	A	NA	NA	0	0	NA	0.01515	0.01515	0.005495	0	0.0177	0	NA	0.009804	NA	0	0
rs563507800	A	NA	NA	0	0	NA	0	0	0	0.004854	0	0	NA	0	NA	0	0
rs148125094	T	NA	NA	NA	NA	NA	NA	NA	NA	NA	NA	NA	NA	NA	NA	NA	NA
rs73372161	A	NA	NA	0	0.005319	NA	0.1869	0	0	0	0.1195	0.009346	NA	0	NA	0	0
rs555995855	C	NA	NA	0	0	NA	0	0	0	0.0534	0	0	NA	0.06863	NA	0	0
rs11281229	TCCAGG	NA	NA	0.01905	0.3298	NA	0.3232	0.3838	0.4011	0.1845	0.2566	0.3832	NA	0.1618	NA	0.01923	0.01515
rs559098267	T	NA	NA	0	0	NA	0	0	0	0	0	0	NA	0	NA	0	0
rs191693032	G	NA	NA	0.004762	0	NA	0	0	0	0	0	0	NA	0	NA	0	0
rs187648498	C	0.5	NA	0	0	NA	0	0	0	0	0	0	NA	0	NA	0	0
rs541109823	T	NA	NA	0	0	NA	0	0	0	0	0	0.004673	NA	0	NA	0	0
rs559668949	G	NA	NA	0	0	NA	0	0	0	0	0	0	NA	0	NA	0	0
rs113670863	T	NA	NA	0	0	NA	0	0	0	0	0	0	NA	0	NA	0	0
rs545494331	C	NA	NA	0	0	NA	0	0	0	0	0	0	NA	0	NA	0	0.005051

SNP	Allele	Chileans	Chopccas	CHS	CLM	Cusco	ESN	FIN	GBR	GIH	GWD	IBS	Iquitos	ITU	Jacarus	JPT	KHV
rs528691645	A	NA	NA	0	0	NA	0	0	0	0	0	0	NA	0	NA	0	0
rs558543141	T	NA	NA	0	0	NA	0	0	0	0	0	0	NA	0	NA	0	0.005051
rs145877432	A	NA	NA	0	0	NA	0.005051	0	0	0	0.00885	0	NA	0	NA	0	0
rs531615786	C	NA	NA	0	0	NA	0	0	0	0	0	0	NA	0.004902	NA	0	0
rs570264808	A	NA	NA	0	0	NA	0.005051	0	0	0	0	0	NA	0	NA	0.009615	0
rs371012018	A	NA	NA	0	0	NA	0	0	0	0	0	0	NA	0	NA	0	0
rs534326511	T	NA	NA	0	0	NA	0	0	0	0	0	0	NA	0	NA	0	0
rs200744510	C	NA	NA	NA	NA	NA	NA	NA	NA	NA	NA	NA	NA	NA	NA	NA	NA
rs77996454	A	NA	NA	0	0.01596	NA	0.05051	0	0	0	0.06637	0	NA	0	NA	0	0
rs140259165	T	NA	NA	0	0	NA	0	0	0	0	0	0	NA	0	NA	0.004808	0
rs58978895	T	NA	NA	0	0.005319	NA	0.2778	0	0	0	0.1283	0.004673	NA	0	NA	0	0.005051
rs564981706	T	NA	NA	0	0	NA	0	0	0	0	0	0	NA	0	NA	0	0
rs149173609	T	NA	NA	0.004762	0	NA	0	0	0	0	0	0	NA	0	NA	0.004808	0
rs559934709	T	NA	NA	0	0	NA	0	0	0	0	0.00885	0	NA	0	NA	0	0
rs569899862	A	NA	NA	0	0.005319	NA	0	0	0	0	0	0	NA	0	NA	0	0
rs540844961	G	NA	NA	0	0	NA	0	0	0	0	0	0	NA	0	NA	0	0
rs189582211	A	NA	NA	0	0	NA	0.005051	0	0	0	0	0	NA	0	NA	0	0
rs553097317	T	NA	NA	0	0	NA	0	0	0	0	0	0	NA	0	NA	0	0
rs563693995	G	NA	NA	0	0	NA	0	0	0	0	0	0	NA	0	NA	0	0
rs2298658	T	NA	NA	0	0	NA	0	0	0	0	0	0	NA	0	NA	0.004808	0
rs532882572	T	NA	NA	0	0	NA	0	0	0	0	0.00885	0	NA	0	NA	0	0
rs145347702	C	NA	NA	0	0	NA	0.005051	0	0	0	0	0	NA	0	NA	0	0
rs148988435	A	NA	NA	0	0	NA	0	0	0	0	0	0	NA	0	NA	0	0
rs561557938	A	NA	NA	0	0	NA	0	0	0	0	0	0	NA	0	NA	0	0
rs140230703	G	NA	NA	0	0	NA	0.005051	0	0	0	0	0	NA	0	NA	0	0
rs141232947	G	NA	NA	0	0	NA	0	0	0	0	0	0	NA	0	NA	0	0
rs61735794	T	NA	NA	0	0.02128	NA	0	0.005051	0.02747	0.009709	0	0.02804	NA	0	NA	0	0
rs117888036	A	NA	NA	0	0	NA	0	0	0	0	0	0	NA	0	NA	0	0
rs538864203	A	NA	NA	0	0	NA	0	0	0	0	0	0	NA	0	NA	0	0
rs540165785	A	NA	NA	0	0	NA	0	0	0	0	0	0.004673	NA	0	NA	0	0
rs79517809	C	NA	NA	0	0	NA	0	0	0	0	0.00885	0	NA	0	NA	0	0
rs144988776	T	NA	NA	0	0	NA	0	0.02525	0.02198	0.004854	0	0.004673	NA	0	NA	0	0
rs565946159	C	NA	NA	0	0	NA	0	0	0	0	0	0	NA	0	NA	0	0
rs574894406	G	NA	NA	0	0	NA	0	0	0	0.004854	0	0	NA	0	NA	0	0
rs145570856	A	NA	NA	0	0.005319	NA	0.0404	0	0	0	0.03097	0	NA	0	NA	0	0
rs146142989	A	NA	NA	0	0	NA	0	0	0	0	0	0	NA	0	NA	0	0
rs556966925	C	NA	NA	0	0	NA	0.005051	0	0	0	0	0	NA	0	NA	0	0
rs573704321	T	NA	NA	0	0	NA	0	0	0	0	0	0	NA	0.004902	NA	0	0
rs376074355	T	NA	NA	0	0	NA	0	0	0	0	0	0	NA	0.009804	NA	0	0
rs61325328	G	NA	NA	0	0	NA	0	0	0	0	0	0	NA	0	NA	0	0
rs9979311	G	0.5	NA	0.3762	0.2287	NA	0.399	0.3939	0.2198	0.1456	0.4513	0.2056	NA	0.2206	NA	0.3942	0.3182
rs538655114	A	NA	NA	0	0	NA	0	0	0	0	0	0	NA	0	NA	0	0
rs150066796	A	NA	NA	0	0	NA	0	0	0.005495	0	0	0	NA	0	NA	0	0
rs201093031	G	NA	NA	0.009524	0	NA	0	0	0	0	0	0	NA	0	NA	0	0
rs378616	C	0.1667	NA	0.3286	0.2287	NA	0.2778	0.3788	0.2637	0.2427	0.3628	0.2804	NA	0.2304	NA	0.3173	0.4242

SNP	Allele	Chileans	Chopccas	CHS	CLM	Cusco	ESN	FIN	GBR	GIH	GWD	IBS	Iquitos	ITU	Jacarus	JPT	KHV
rs573152591	A	NA	NA	0	0	NA	0.005051	0	0	0	0	0	NA	0	NA	0	0
rs4818241	A	0.4	NA	0.4143	0.1649	NA	0.0101	0.04545	0.01099	0.06311	0.01327	0.004673	NA	0.07843	NA	0.2981	0.4394
rs189181802	T	NA	NA	0	0	NA	0	0	0	0	0	0	NA	0	NA	0	0
rs566323147	A	NA	NA	0	0	NA	0	0	0	0	0	0	NA	0.004902	NA	0	0
rs184164037	C	NA	NA	0	0	NA	0.01515	0	0	0	0.004425	0	NA	0	NA	0	0
rs77675406	A	0.5	0	0.05238	0.1011	0	0.07071	0.1919	0.1209	0.03883	0.07965	0.08411	0.09615	0.05882	0.05882	0.0625	0.02525
rs528276471	G	NA	NA	0	0	NA	0	0	0	0	0	0	NA	0	NA	0	0
rs552912416	T	NA	NA	0	0	NA	0	0.005051	0	0	0	0	NA	0	NA	0	0
rs572279833	A	NA	NA	0	0	NA	0	0	0	0	0	0	NA	0	NA	0	0.005051
rs559830930	G	NA	NA	0	0.005319	NA	0	0	0	0	0	0	NA	0	NA	0	0
rs2298660	T	0.5	NA	0.2619	0.1702	NA	0.4747	0.3333	0.1868	0.1359	0.354	0.1402	NA	0.1716	NA	0.3173	0.1919
rs527684898	G	NA	NA	0	0.005319	NA	0	0	0	0	0	0	NA	0	NA	0	0
rs181332845	G	NA	NA	0	0	NA	0.01515	0	0	0	0	0	NA	0	NA	0	0
rs182175876	G	NA	NA	0	0	NA	0	0	0	0	0	0	NA	0	NA	0	0
rs539218228	A	NA	NA	0	0	NA	0.005051	0	0	0	0	0	NA	0	NA	0	0
rs116865960	A	NA	NA	0	0.01064	NA	0.01515	0.0202	0.02747	0	0	0.01402	NA	0	NA	0	0
rs183398094	C	NA	NA	0	0	NA	0	0	0	0	0	0	NA	0	NA	0	0
rs567340892	A	NA	NA	0	0	NA	0	0	0	0	0.009259	0	NA	0	NA	0	0
rs190125027	T	NA	NA	0	0	NA	0	0	0	0	0	0	NA	0	NA	0	0
rs75430506	A	NA	NA	0.009524	0	NA	0	0	0	0	0	0	NA	0	NA	0.03846	0.005051
rs138812485	A	NA	NA	0	0	NA	0	0	0	0	0	0	NA	0	NA	0	0
rs56066678	T	0.6667	0.1765	0.2095	0.2606	0.2917	0.4192	0.1465	0.2527	0.2864	0.4248	0.271	0.1731	0.2598	0.2647	0.3029	0.2323
rs561063944	A	NA	NA	0	0	NA	0	0	0	0	0	0	NA	0	NA	0	0
rs17854725	G	0.5	NA	0.1095	0.3617	NA	0.4192	0.5606	0.5604	0.5049	0.2522	0.5467	NA	0.4559	NA	0.1923	0.08586
rs533050705	A	NA	NA	0	0.005319	NA	0	0	0	0	0	0	NA	0	NA	0	0
rs545910743	C	NA	NA	0	0	NA	0	0	0	0	0	0	NA	0	NA	0	0
rs149021153	G	NA	NA	0	0	NA	0.0101	0	0	0	0	0	NA	0	NA	0	0
rs371583288	A	NA	NA	0	0	NA	0	0	0	0	0	0	NA	0	NA	0	0
rs552174617	C	NA	NA	0	0	NA	0	0	0	0	0	0	NA	0	NA	0	0
rs144157441	A	NA	NA	0	0	NA	0.005051	0	0	0	0.0354	0	NA	0	NA	0	0
rs559811756	A	NA	NA	0	0	NA	0.0101	0	0	0	0.004425	0	NA	0	NA	0	0
rs536642840	A	NA	NA	0	0	NA	0	0	0	0	0	0	NA	0	NA	0	0
rs572530227	T	NA	NA	0	0	NA	0	0	0	0	0	0	NA	0	NA	0	0
rs551045243	A	NA	NA	0	0	NA	0	0	0	0	0	0	NA	0	NA	0	0
rs374886738	C	NA	NA	0	0	NA	0	0	0	0	0.00885	0	NA	0	NA	0	0
rs557714443	A	NA	NA	0	0	NA	0	0	0	0	0	0	NA	0	NA	0	0
rs402303	C	0.625	0.2647	0.3333	0.5798	0.4167	0.3535	0.5505	0.7637	0.7282	0.4071	0.7617	0.4231	0.6667	0.2941	0.4087	0.298
rs544822322	C	NA	NA	0	0	NA	0.005051	0	0	0	0	0	NA	0	NA	0	0
rs186429734	A	NA	NA	0	0	NA	0	0	0	0	0	0	NA	0	NA	0	0
rs138056981	T	NA	NA	0	0	NA	0	0	0	0	0	0	NA	0	NA	0	0
rs79468500	G	NA	NA	0	0.02128	NA	0.0202	0.005051	0.02747	0.02427	0.04867	0.02336	NA	0.004902	NA	0	0
rs2156301	T	0.4	NA	0.4143	0.1649	NA	0.0101	0.04545	0.01099	0.06311	0.02655	0.004673	NA	0.07843	NA	0.2981	0.4394
rs191228684	T	NA	NA	0	0	NA	0	0	0	0	0	0	NA	0	NA	0	0
rs563812695	A	NA	NA	0	0	NA	0	0	0	0	0	0	NA	0	NA	0	0
rs8128074	T	0.1667	0.05882	0.2143	0.117	0.1389	0.04545	0.1364	0.1264	0.1359	0.04425	0.1308	0.14	0.1127	0.1765	0.274	0.2929

SNP	Allele	Chileans	Chopccas	CHS	CLM	Cusco	ESN	FIN	GBR	GIH	GWD	IBS	Iquitos	ITU	Jacarus	JPT	KHV
rs560788815	C	NA	NA	0	0	NA	0	0	0	0	0	0	NA	0	NA	0	0
rs183851466	T	NA	NA	0	0.005319	NA	0	0	0	0	0	0	NA	0	NA	0	0
rs151152524	C	NA	NA	0	0	NA	0	0	0.005495	0	0	0.009346	NA	0	NA	0	0
rs113506821	T	NA	0.02941	0	0.04787	0.04167	0	0.07576	0.02198	0.04369	0	0.05607	0.1154	0	0.02941	0.004808	0
rs530527676	A	NA	NA	0	0	NA	0	0	0	0	0	0	NA	0	NA	0	0.005051
rs550252022	C	NA	NA	0	0	NA	0.005051	0	0	0	0	0	NA	0	NA	0	0
rs386638	C	0.4	NA	0.5095	0.1649	NA	0.0101	0.05051	0.01099	0.06796	0.01327	0.004673	NA	0.07843	NA	0.4567	0.5152
rs148136016	G	NA	NA	0	0	NA	0	0	0	0	0	0	NA	0	NA	0	0
rs114848359	T	NA	NA	0	0	NA	0.005051	0	0	0	0	0	NA	0	NA	0	0
rs530009764	C	NA	NA	0	0	NA	0	0.005051	0	0	0	0	NA	0	NA	0	0
rs371965055	T	NA	NA	0	0	NA	0	0	0	0.09709	0	0	NA	0.05882	NA	0	0
rs183984610	A	NA	NA	0	0	NA	0	0	0.005495	0	0	0.004673	NA	0	NA	0	0
rs527305819	A	NA	NA	0	0	NA	0	0	0	0	0	0	NA	0	NA	0	0
rs181461974	T	NA	NA	0	0.01064	NA	0.0101	0	0	0	0	0	NA	0	NA	0	0
rs566457884	T	NA	NA	0	0	NA	0	0	0	0	0	0	NA	0	NA	0	0
rs540070564	A	NA	NA	0	0	NA	0	0	0	0	0	0	NA	0	NA	0	0
rs563555305	T	NA	NA	0	0	NA	0	0	0	0	0	0	NA	0	NA	0	0
rs151189718	T	NA	NA	0	0	NA	0	0	0	0	0	0.004673	NA	0	NA	0	0
rs569681994	C	NA	NA	0	0	NA	0	0	0	0	0	0.004673	NA	0	NA	0	0
rs143097165	A	NA	NA	0	0	NA	0.0101	0	0	0	0.004425	0	NA	0	NA	0	0
rs541200538	G	NA	NA	0.004762	0	NA	0	0	0	0	0	0	NA	0	NA	0	0
rs11701576	G	NA	NA	0.3381	0.1383	NA	0.08081	0.07071	0.1044	0.1311	0.1637	0.1262	NA	0.2304	NA	0.2933	0.2374
rs17001042	A	NA	NA	0	0.005319	NA	0.1515	0	0	0	0.1195	0.004673	NA	0	NA	0	0
rs558449333	T	NA	NA	0	0	NA	0	0	0	0	0	0	NA	0.004902	NA	0	0
rs569866330	A	NA	NA	0	0	NA	0	0.005051	0	0	0	0	NA	0	NA	0	0
rs573234046	T	NA	NA	0	0	NA	0.005051	0	0	0	0	0	NA	0	NA	0	0
rs114363287	T	NA	NA	0	0	NA	0.0101	0	0	0	0	0	NA	0	NA	0	0
rs55896064	A	0.5	NA	0.05238	0.1011	NA	0.07071	0.1919	0.1209	0.03883	0.07965	0.08879	NA	0.05882	NA	0.0625	0.02525
rs547961708	T	NA	NA	0	0	NA	0	0	0	0	0.004425	0	NA	0	NA	0	0
rs542712985	G	NA	NA	0	0	NA	0	0	0	0	0	0	NA	0	NA	0	0
rs117941520	A	NA	NA	0	0	NA	0	0.0202	0.01648	0	0	0.03738	NA	0	NA	0	0
rs73903404	G	NA	NA	0	0	NA	0.08586	0	0	0	0.02212	0	NA	0	NA	0	0
rs551817968	T	NA	NA	0.004762	0	NA	0	0	0	0	0	0	NA	0	NA	0	0
rs550208798	T	NA	NA	0	0	NA	0	0	0	0	0	0	NA	0	NA	0	0
rs542471574	A	NA	NA	0	0	NA	0	0	0	0	0	0	NA	0.004902	NA	0	0
rs145841350	T	NA	NA	0.004762	0	NA	0	0	0	0	0	0	NA	0	NA	0	0
rs7278627	A	NA	0	0	0.005319	0	0.1919	0	0	0	0.1239	0.009346	0	0	0	0	0
rs185555838	A	NA	NA	0	0	NA	0.005051	0	0	0	0	0	NA	0	NA	0	0
rs374666536	T	NA	NA	0	0	NA	0	0	0	0	0	0	NA	0	NA	0	0
rs7278739	A	NA	NA	0	0.01064	NA	0.2677	0	0	0	0.2434	0.009346	NA	0.009804	NA	0	0
rs555667242	C	NA	NA	0	0	NA	0	0	0	0	0	0	NA	0	NA	0	0
rs7364083	G	0.3889	NA	0.3762	0.4202	NA	0.1313	0.4495	0.4505	0.4806	0.1062	0.4907	NA	0.5098	NA	0.2981	0.4293
rs527284833	G	NA	NA	0	0	NA	0.0101	0	0	0	0.004425	0	NA	0	NA	0	0
rs139458208	C	NA	NA	0	0	NA	0	0	0	0	0	0	NA	0	NA	0	0
rs139001727	G	NA	NA	0	0.01064	NA	0	0	0	0	0	0	NA	0	NA	0	0

SNP	Allele	Chileans	Chopccas	CHS	CLM	Cusco	ESN	FIN	GBR	GIH	GWD	IBS	Iquitos	ITU	Jacarus	JPT	KHV
rs4290734	G	0.5	NA	0	0.3457	NA	0.05556	0.4141	0.511	0.5049	0.04867	0.5187	NA	0.4461	NA	0.004808	0.01515
rs2156300	G	0.4	NA	0.4143	0.1649	NA	0.0101	0.04545	0.01099	0.06311	0.01327	0.004673	NA	0.07843	NA	0.2981	0.4394
rs555328523	T	NA	NA	0.02381	0	NA	0	0	0	0	0	0	NA	0	NA	0	0.005051
rs575168295	G	NA	NA	0	0	NA	0	0	0	0	0	0	NA	0	NA	0	0
rs145355824	A	NA	NA	0	0	NA	0	0	0	0	0	0.004673	NA	0	NA	0	0
rs375827195	G	NA	NA	0	0	NA	0	0	0	0	0	0	NA	0	NA	0	0.0101
rs533805020	T	NA	NA	0	0	NA	0	0	0	0	0	0	NA	0.004902	NA	0	0
rs536311940	G	NA	NA	0	0	NA	0	0	0	0	0	0	NA	0	NA	0	0
rs371046741	A	NA	NA	0	0	NA	0.005051	0	0	0	0.02212	0.004673	NA	0	NA	0	0
rs2838042	C	0.5	0.02941	0.2619	0.1383	0.01389	0.2121	0.3182	0.2308	0.2913	0.2876	0.2009	0.1154	0.348	0.05882	0.2404	0.2626
rs192334131	A	NA	NA	0	0	NA	0	0.005051	0	0	0	0	NA	0	NA	0	0
rs557822084	T	NA	NA	0.004762	0	NA	0	0	0	0	0	0	NA	0	NA	0	0.005051
rs566208761	C	NA	NA	0	0	NA	0	0	0	0	0	0	NA	0	NA	0	0
rs546335233	A	NA	NA	0	0	NA	0	0	0	0.004854	0	0	NA	0.004902	NA	0	0
rs184767756	C	NA	NA	0	0	NA	0	0	0.005495	0	0	0	NA	0	NA	0	0
rs528259213	A	NA	NA	0	0	NA	0	0	0	0	0	0	NA	0	NA	0	0.005051
rs115265507	A	NA	NA	0	0.01596	NA	0.005051	0	0	0	0	0	NA	0	NA	0	0
rs200072801	G	NA	NA	0	0	NA	0	0	0	0	0	0	NA	0	NA	0.01442	0
rs138765307	T	NA	NA	0	0	NA	0	0	0	0	0	0	NA	0	NA	0	0
rs532005855	C	NA	NA	0	0	NA	0	0	0	0	0	0	NA	0	NA	0	0.005051
rs2838043	T	0.5	NA	0.05238	0.1702	NA	0.0303	0.1212	0.2253	0.1699	0.0354	0.2243	NA	0.08824	NA	0.05769	0.005051
rs115596471	A	NA	NA	0	0	NA	0.09091	0	0	0	0.07522	0	NA	0	NA	0	0
rs574538362	T	NA	NA	0	0	NA	0	0	0	0	0	0	NA	0	NA	0	0
rs190618812	A	NA	NA	0.004762	0	NA	0	0	0	0	0	0	NA	0	NA	0.004808	0.01515
rs141583878	A	NA	NA	0	0	NA	0	0	0	0	0	0	NA	0	NA	0	0
rs571805265	T	NA	NA	0	0	NA	0	0	0	0	0	0	NA	0	NA	0	0
rs371497524	A	NA	NA	0	0	NA	0	0	0	0	0	0	NA	0	NA	0	0
rs540908584	T	NA	NA	0	0	NA	0	0	0	0	0	0	NA	0	NA	0	0
rs73357642	A	NA	NA	0	0.005319	NA	0.2778	0	0	0	0.2345	0.004673	NA	0	NA	0	0.005051
rs192854367	A	NA	NA	0	0	NA	0	0	0	0	0	0	NA	0	NA	0	0
rs371180501	G	NA	NA	0	0	NA	0.0101	0	0	0	0.0177	0	NA	0	NA	0	0
rs467375	A	NA	0.02941	0	0.2606	0.06944	0.09091	0.404	0.4341	0.4126	0.04867	0.486	0.1346	0.4167	0.02941	0.004808	0.01515
rs562045100	A	NA	NA	0	0	NA	0	0	0	0	0	0	NA	0	NA	0	0
rs34205539	AT	NA	NA	0	0.07979	NA	0.03535	0.09596	0.1099	0.1068	0.05752	0.09813	NA	0.07353	NA	0	0
rs186418926	A	NA	NA	0	0	NA	0	0.0101	0	0	0	0	NA	0	NA	0	0
rs539528622	T	NA	NA	0	0	NA	0	0	0	0	0	0	NA	0	NA	0	0
rs145292327	G	NA	NA	0	0	NA	0	0	0	0	0	0	NA	0	NA	0	0
rs372405355	C	NA	NA	0	0	NA	0	0	0	0	0	0	NA	0	NA	0	0.005051
rs544417878	T	NA	NA	0	0	NA	0	0	0	0	0	0	NA	0	NA	0	0
rs559363556	A	NA	NA	0	0	NA	0	0	0	0	0	0	NA	0	NA	0	0
rs570467504	C	NA	NA	0	0	NA	0	0	0	0	0	0	NA	0	NA	0	0
rs148701953	C	NA	NA	0	0	NA	0	0	0	0	0	0	NA	0	NA	0	0
rs569988060	A	NA	NA	0	0	NA	0	0	0	0	0	0	NA	0	NA	0	0
rs182254633	G	NA	NA	0	0	NA	0.005051	0	0	0	0	0	NA	0	NA	0	0
rs563305848	C	NA	NA	0	0	NA	0	0	0	0.004854	0	0	NA	0.004902	NA	0	0

SNP	Allele	Chileans	Chopccas	CHS	CLM	Cusco	ESN	FIN	GBR	GIH	GWD	IBS	Iquitos	ITU	Jacarus	JPT	KHV
rs528477904	C	NA	NA	0	0	NA	0	0	0.005495	0	0	0	NA	0	NA	0	0
rs550874821	G	NA	NA	0	0	NA	0	0	0	0	0	0	NA	0	NA	0	0
rs73372193	C	NA	NA	0	0.005319	NA	0.2778	0	0	0	0.146	0.004673	NA	0	NA	0	0.005051
rs552607028	A	NA	NA	0	0	NA	0	0	0	0	0	0	NA	0.009804	NA	0	0
rs553861191	T	NA	NA	0	0	NA	0	0	0	0	0	0	NA	0	NA	0	0
rs147977027	C	NA	NA	0	0	NA	0	0	0	0	0	0	NA	0	NA	0.004808	0
rs533445403	T	NA	NA	0	0	NA	0	0	0	0	0	0	NA	0	NA	0	0
rs578005531	T	NA	NA	0	0	NA	0	0	0	0.004854	0	0	NA	0	NA	0	0
rs140605461	A	NA	NA	0	0	NA	0	0	0	0	0	0	NA	0	NA	0.01442	0
rs544023509	A	NA	NA	0	0	NA	0.005051	0	0	0	0	0	NA	0	NA	0	0
rs61735792	A	NA	NA	0	0.01596	NA	0	0.005051	0.01099	0.004854	0	0.02336	NA	0	NA	0	0
rs568256706	G	NA	NA	0	0	NA	0	0	0	0	0	0	NA	0	NA	0	0
rs8131648	T	0.4286	NA	0.7952	0.3936	NA	0.4949	0.399	0.2253	0.2427	0.385	0.1916	NA	0.3088	NA	0.6779	0.7778
rs527964326	C	NA	NA	0	0	NA	0	0	0	0	0.004425	0	NA	0	NA	0	0
rs191697229	A	NA	NA	0	0	NA	0.005051	0	0	0	0.02655	0	NA	0	NA	0	0
rs147359020	A	NA	NA	0	0	NA	0	0	0.005495	0.04369	0	0	NA	0.04902	NA	0	0.005051
rs532085624	T	NA	NA	0	0	NA	0	0	0	0	0	0	NA	0.009804	NA	0	0
rs577753477	A	NA	NA	0	0	NA	0	0	0	0	0	0	NA	0	NA	0	0
rs181091055	C	NA	NA	0	0	NA	0	0	0	0	0	0	NA	0	NA	0	0
rs546447704	T	NA	NA	0	0	NA	0	0	0	0	0	0	NA	0	NA	0	0
rs575335056	CA	NA	NA	0	0	NA	0	0	0	0	0	0	NA	0.004902	NA	0	0
rs536763630	A	NA	NA	0	0	NA	0	0	0	0	0.004425	0	NA	0	NA	0	0
rs184500277	T	NA	NA	0.004762	0	NA	0	0	0	0	0	0	NA	0	NA	0	0.0101
rs569292495	G	NA	NA	0	0	NA	0	0	0	0.004854	0	0	NA	0	NA	0	0
rs73372163	A	0.5	NA	0.05238	0.1064	NA	0.2879	0.1919	0.1209	0.03883	0.2655	0.09813	NA	0.05882	NA	0.0625	0.02525
rs554443322	C	NA	NA	0	0	NA	0	0	0	0	0.004425	0	NA	0	NA	0	0
rs568346732	T	NA	NA	0	0	NA	0	0	0	0	0	0	NA	0.004902	NA	0	0
rs114641598	C	NA	NA	0	0	NA	0.005051	0	0	0	0.0177	0	NA	0	NA	0	0
rs549459413	A	NA	NA	0	0	NA	0	0	0	0	0	0	NA	0	NA	0	0
rs184380117	A	NA	NA	0	0	NA	0	0	0	0	0	0	NA	0	NA	0	0.005051
rs576181035	G	NA	NA	0	0	NA	0	0	0	0	0.004425	0	NA	0	NA	0	0
rs201679623	C	NA	NA	0.01429	0	NA	0	0	0	0	0	0	NA	0	NA	0	0.005051
rs568846685	A	NA	NA	0	0	NA	0.005051	0	0	0	0	0	NA	0	NA	0	0
rs548718178	C	NA	NA	0	0	NA	0.005051	0	0	0	0	0	NA	0	NA	0	0
rs537584838	G	NA	NA	0	0	NA	0	0	0	0	0	0	NA	0	NA	0	0
rs569259137	A	NA	NA	0	0	NA	0	0	0	0.004854	0	0	NA	0	NA	0	0
rs367885466	T	NA	NA	0	0	NA	0	0	0	0	0	0	NA	0	NA	0	0
rs2410430	A	0.4	NA	0.4143	0.1649	NA	0.02525	0.04545	0.01099	0.06311	0.0177	0.004673	NA	0.07843	NA	0.2981	0.4394
rs559637785	A	NA	NA	0.004762	0	NA	0	0	0	0	0	0	NA	0	NA	0	0
rs573343651	C	NA	NA	0	0	NA	0	0	0	0	0	0	NA	0	NA	0	0
rs34769294	CA	0.5	NA	0.2762	0.2606	NA	0.1919	0.1515	0.2363	0.2864	0.1903	0.2664	NA	0.25	NA	0.3365	0.2727
rs7364088	A	0.5	NA	0.419	0.2553	NA	0.3081	0.3485	0.2363	0.2039	0.3407	0.2196	NA	0.2255	NA	0.4183	0.3586
rs551432766	T	NA	NA	0	0	NA	0	0	0	0	0	0	NA	0	NA	0	0
rs565299100	G	NA	NA	0	0	NA	0	0	0	0	0	0.004673	NA	0	NA	0	0
rs538252674	C	NA	NA	0	0	NA	0	0.005051	0	0	0	0	NA	0	NA	0	0

SNP	Allele	Chileans	Chopccas	CHS	CLM	Cusco	ESN	FIN	GBR	GIH	GWD	IBS	Iquitos	ITU	Jacarus	JPT	KHV
rs368994585	A	NA	NA	0	0	NA	0	0	0	0	0	0	NA	0	NA	0	0
rs192709500	A	NA	NA	0.004762	0	NA	0.005051	0	0	0	0	0	NA	0	NA	0	0
rs192955773	C	NA	NA	0	0	NA	0.0101	0	0	0	0.004425	0	NA	0	NA	0	0
rs371020678	T	NA	NA	0	0	NA	0	0	0	0	0	0	NA	0.009804	NA	0	0
rs556435793	A	NA	NA	0	0	NA	0	0	0	0	0	0	NA	0.004902	NA	0	0
rs549990870	G	NA	NA	0	0	NA	0	0	0	0.004854	0	0	NA	0	NA	0	0
rs146723217	T	NA	NA	0	0.01064	NA	0.0202	0	0	0	0.03982	0	NA	0	NA	0	0
rs145738510	C	NA	NA	0	0	NA	0	0	0	0	0	0	NA	0	NA	0	0
rs114844880	C	NA	NA	0	0	NA	0.0101	0	0	0	0	0	NA	0	NA	0	0
rs370641046	T	NA	NA	0	0	NA	0	0	0	0	0	0	NA	0.009804	NA	0	0
rs528452128	GTGTTT	NA	NA	0	0	NA	0	0	0	0	0	0	NA	0	NA	0	0
rs11910678	C	NA	0	0.1	0.02128	0	0.1768	0	0	0	0.1504	0	0	0	0	0.06731	0.101
rs140625413	C	NA	NA	0	0	NA	0	0	0	0	0	0	NA	0	NA	0	0
rs142444476	T	NA	NA	0	0	NA	0.005051	0	0	0	0	0	NA	0	NA	0	0
rs545149163	A	NA	NA	0	0	NA	0	0	0	0	0	0	NA	0	NA	0	0
rs149798653	C	NA	NA	0	0	NA	0.005051	0	0	0	0.04425	0	NA	0	NA	0	0
rs527416684	A	NA	NA	0	0	NA	0	0	0	0.01942	0	0	NA	0.03431	NA	0	0
rs189067157	A	NA	NA	0	0	NA	0	0	0	0	0	0	NA	0	NA	0.009615	0
rs527489879	C	NA	NA	0	0	NA	0	0	0	0	0	0	NA	0	NA	0	0.0101
rs558824762	T	NA	NA	0	0	NA	0	0	0	0	0.05752	0	NA	0	NA	0	0
rs370927492	T	NA	NA	0	0	NA	0	0	0	0	0	0	NA	0	NA	0	0
rs1003030	G	NA	NA	0.3381	0.1383	NA	0.08081	0.07071	0.1044	0.1311	0.1637	0.1262	NA	0.2304	NA	0.2933	0.2374
rs543908797	A	NA	NA	0	0	NA	0	0	0	0	0.004425	0	NA	0	NA	0	0
rs567103273	G	NA	NA	0	0	NA	0	0	0	0	0	0	NA	0	NA	0	0
rs565820920	T	NA	NA	0	0	NA	0	0	0	0	0	0.004673	NA	0	NA	0	0
rs548463596	G	NA	NA	0.2429	0.3245	NA	0.3737	0.3131	0.2747	0.2087	0.4336	0.2383	NA	0.2304	NA	0.149	0.1919
rs561570726	T	NA	NA	0	0	NA	0	0	0	0	0	0	NA	0	NA	0	0
rs555911696	A	NA	NA	0	0	NA	0	0	0	0	0	0	NA	0	NA	0	0
rs561179495	T	NA	NA	0	0	NA	0	0	0	0	0	0	NA	0	NA	0	0
rs181972300	A	NA	NA	0	0	NA	0	0	0	0	0	0	NA	0	NA	0	0
rs138498737	A	NA	NA	0	0.01596	NA	0	0.005051	0.01099	0.004854	0	0.01402	NA	0	NA	0	0
rs565468881	T	NA	NA	0	0	NA	0	0	0	0	0	0	NA	0	NA	0	0
rs186734573	T	NA	NA	0	0	NA	0	0	0.005495	0	0	0	NA	0	NA	0	0
rs146132480	A	NA	NA	0	0.01064	NA	0	0.005051	0.02747	0	0	0.004673	NA	0	NA	0	0
rs559125514	C	NA	NA	0	0	NA	0	0	0	0	0	0	NA	0.004902	NA	0	0
rs527499027	T	NA	NA	0	0	NA	0	0	0	0	0	0	NA	0	NA	0	0
rs573736906	A	NA	NA	0	0	NA	0	0	0	0.01942	0	0	NA	0.02451	NA	0	0
rs573613070	G	NA	NA	0	0	NA	0	0	0	0	0	0	NA	0	NA	0	0
rs542898542	T	NA	NA	0	0	NA	0	0	0	0	0	0	NA	0	NA	0	0
rs422471	C	0.375	0.7353	0.7476	0.4202	0.5833	0.4242	0.4596	0.2473	0.2767	0.4071	0.243	0.58	0.3333	0.7059	0.6827	0.7121
rs181058683	A	NA	NA	0	0	NA	0	0	0	0.009709	0	0	NA	0	NA	0	0
rs149855493	C	NA	NA	0	0	NA	0.0101	0	0	0	0.004425	0	NA	0	NA	0	0
rs191457025	T	NA	NA	0	0	NA	0	0	0	0	0	0	NA	0	NA	0	0.0101
rs375760	T	0.6667	NA	0.2524	0.25	NA	0.1313	0.1465	0.2418	0.2816	0.0885	0.2477	NA	0.25	NA	0.3125	0.2677
rs148038688	T	NA	NA	0	0	NA	0.0101	0	0	0	0.004425	0	NA	0	NA	0	0

SNP	Allele	Chileans	Chopccas	CHS	CLM	Cusco	ESN	FIN	GBR	GIH	GWD	IBS	Iquitos	ITU	Jacarus	JPT	KHV
rs145728087	A	NA	NA	0	0	NA	0	0	0	0	0.00885	0	NA	0	NA	0	0
rs569947342	T	NA	NA	0	0	NA	0	0	0	0	0.004425	0	NA	0	NA	0	0
rs562599408	G	NA	NA	0	0	NA	0	0	0	0	0	0	NA	0	NA	0	0
rs146252393	A	NA	NA	0	0	NA	0	0	0	0	0	0	NA	0	NA	0	0
rs73372182	A	NA	NA	0	0.005319	NA	0.2879	0	0	0	0.177	0.009346	NA	0	NA	0	0
rs566012796	C	NA	NA	0	0	NA	0	0	0.005495	0	0	0	NA	0	NA	0	0
rs74423429	A	NA	NA	0	0	NA	0	0.02525	0	0.009709	0	0.03738	NA	0	NA	0	0
rs193067129	A	NA	NA	0	0	NA	0	0	0.005495	0	0	0	NA	0	NA	0	0.005051
rs572143040	C	NA	NA	0	0	NA	0	0	0	0	0	0	NA	0	NA	0	0
rs9984012	T	NA	NA	0.07619	0.09043	NA	0	0.2222	0.1209	0.09709	0.004425	0.1308	NA	0.09804	NA	0.03846	0.09596
rs141898436	C	NA	NA	0	0	NA	0.0101	0	0	0	0.01327	0	NA	0	NA	0	0
rs558272207	A	NA	NA	0	0	NA	0	0	0	0	0	0	NA	0	NA	0	0
rs146681599	A	NA	NA	0	0	NA	0	0	0	0	0	0	NA	0	NA	0	0
rs743542	A	0.5	0.2353	0.2667	0.08511	0.25	0.1162	0.1364	0.05495	0.1359	0.1106	0.0514	0.2308	0.1569	0.1176	0.2885	0.2323
rs532511763	T	NA	NA	0	0	NA	0	0	0	0	0	0	NA	0	NA	0	0
rs149424945	T	NA	NA	0.004762	0	NA	0	0	0	0	0	0	NA	0	NA	0	0
rs568605816	A	NA	NA	0	0	NA	0.005051	0	0	0	0	0	NA	0	NA	0	0
rs184859933	T	NA	NA	0	0	NA	0	0	0	0	0	0	NA	0	NA	0	0
rs530918384	GGTGA	NA	NA	0	0	NA	0	0.01515	0	0.004854	0.004425	0	NA	0.004902	NA	0	0
rs28369457	T	NA	NA	0	0	NA	0	0	0	0	0.004425	0	NA	0	NA	0	0
rs556192760	A	NA	NA	0.004762	0	NA	0	0	0	0	0	0	NA	0	NA	0	0
rs576447507	C	NA	NA	0	0	NA	0	0	0	0	0	0	NA	0	NA	0	0
rs546512831	A	NA	NA	0	0	NA	0	0	0	0	0	0	NA	0	NA	0	0
rs543066660	A	NA	NA	0	0	NA	0	0	0	0	0	0	NA	0	NA	0	0.005051
rs543404937	T	NA	NA	0	0	NA	0	0	0	0	0	0	NA	0.004902	NA	0	0
rs180826598	C	NA	NA	0	0	NA	0	0	0	0	0	0	NA	0	NA	0	0
rs368878191	T	NA	NA	0	0	NA	0	0	0	0	0	0	NA	0	NA	0	0.005051
rs369619813	G	NA	NA	0	0	NA	0	0	0	0	0	0	NA	0	NA	0	0
rs112213575	A	NA	NA	0	0	NA	0.0202	0	0	0	0.0177	0	NA	0	NA	0	0
rs548725701	A	NA	NA	0	0	NA	0	0	0	0	0	0.004673	NA	0	NA	0	0
rs569959837	A	NA	NA	0	0	NA	0	0	0	0	0	0	NA	0.009804	NA	0	0
rs554986094	A	NA	NA	0	0	NA	0	0.005051	0	0	0	0	NA	0	NA	0	0
rs456142	T	0.2778	NA	0.681	0.3138	NA	0.4141	0.2374	0.1374	0.2136	0.3894	0.1121	NA	0.3431	NA	0.5288	0.7071
rs73357644	T	NA	NA	0	0.005319	NA	0.2828	0	0	0	0.2345	0.004673	NA	0	NA	0	0.005051
rs576259678	C	NA	NA	0	0	NA	0	0	0	0.01456	0	0	NA	0.004902	NA	0	0
rs140141551	A	NA	NA	0	0.01064	NA	0	0	0.005495	0.004854	0	0.009346	NA	0	NA	0	0
rs146564124	T	NA	NA	0	0.01064	NA	0	0.01515	0.02747	0.004854	0	0.01402	NA	0.009804	NA	0	0
rs558715322	T	NA	NA	0	0	NA	0	0	0	0	0.004425	0	NA	0	NA	0	0
rs116020930	A	NA	NA	0	0	NA	0	0	0	0	0	0	NA	0	NA	0	0
rs539333191	T	NA	NA	0	0	NA	0	0	0	0	0	0	NA	0	NA	0	0
rs192259532	T	NA	NA	0	0	NA	0	0.005051	0	0	0	0.009346	NA	0	NA	0	0
rs531920590	T	NA	NA	0	0	NA	0	0	0	0	0	0	NA	0	NA	0	0
rs531300154	C	NA	NA	0	0	NA	0	0	0	0	0	0	NA	0.004902	NA	0	0
rs147945120	T	NA	NA	0	0	NA	0	0	0.005495	0	0	0	NA	0	NA	0	0
rs147934552	A	NA	NA	0	0	NA	0	0	0	0	0	0	NA	0	NA	0.004808	0

SNP	Allele	Chileans	Chopccas	CHS	CLM	Cusco	ESN	FIN	GBR	GIH	GWD	IBS	Iquitos	ITU	Jacarus	JPT	KHV
rs112980967	A	NA	NA	0	0	NA	0.0202	0	0	0	0.02212	0	NA	0	NA	0	0
rs149601802	T	NA	NA	0	0	NA	0	0	0	0	0	0	NA	0	NA	0	0
rs3761373	T	NA	NA	0.3381	0.1383	NA	0.08081	0.07071	0.1044	0.1311	0.1637	0.1262	NA	0.2304	NA	0.2933	0.2374
rs545333396	C	NA	NA	0	0	NA	0	0	0	0	0.00885	0	NA	0	NA	0	0
rs574683527	A	NA	NA	0	0	NA	0	0	0	0.01456	0	0	NA	0.004902	NA	0	0
rs189431131	C	NA	NA	0	0	NA	0	0	0	0	0	0	NA	0	NA	0	0
rs183147330	A	NA	NA	0	0	NA	0	0.005051	0	0	0	0	NA	0	NA	0	0
rs417443	A	0.4	NA	0.4333	0.1649	NA	0.0101	0.04545	0.01099	0.06311	0.01327	0.004673	NA	0.07843	NA	0.3365	0.4596
rs564403172	T	NA	NA	0	0	NA	0	0	0	0	0.004425	0	NA	0	NA	0	0
rs8134216	T	0.5714	0.08824	0.2048	0.6064	0.1944	0.4192	0.601	0.7802	0.7573	0.5487	0.8084	0.3269	0.6912	0.2059	0.3125	0.2222
rs191441931	T	NA	NA	0	0	NA	0	0	0	0	0.004425	0	NA	0	NA	0	0
rs567188556	T	NA	NA	0	0	NA	0	0	0	0	0	0	NA	0	NA	0	0
rs187662898	T	NA	NA	0	0.005319	NA	0	0	0	0	0.004425	0	NA	0	NA	0	0
rs114911304	A	NA	NA	0	0	NA	0.0202	0	0	0	0.0177	0	NA	0	NA	0	0
rs558858943	C	NA	NA	0	0	NA	0	0	0	0	0	0	NA	0	NA	0	0
rs541396507	C	NA	NA	0	0	NA	0	0	0	0	0	0	NA	0	NA	0	0
rs565142599	T	NA	NA	0	0	NA	0	0	0	0	0	0	NA	0	NA	0	0
rs146605032	A	NA	NA	0	0	NA	0	0	0	0	0	0	NA	0	NA	0	0
rs553673225	G	NA	NA	0	0	NA	0	0	0	0	0	0	NA	0	NA	0.004808	0
rs113034290	T	NA	NA	0	0	NA	0	0	0	0	0.004425	0	NA	0	NA	0	0
rs150554820	T	NA	NA	NA	NA	NA	NA	NA	NA	NA	NA	NA	NA	NA	NA	NA	NA
rs544880781	A	NA	NA	0	0	NA	0	0	0	0	0.00885	0	NA	0	NA	0	0
rs6517669	A	0.5	1	0.6905	0.7394	0.97222	0.3182	0.5859	0.7692	0.7864	0.3274	0.757	0.90385	0.7451	0.91176	0.6635	0.7475
rs550238535	A	NA	NA	0	0	NA	0	0	0	0	0	0	NA	0	NA	0	0
rs455922	T	0.4	NA	0.3762	0.1649	NA	0.005051	0.0404	0.01099	0.06796	0	0.004673	NA	0.09314	NA	0.2885	0.4141
rs374203194	A	NA	NA	0	0	NA	0	0	0	0	0	0	NA	0	NA	0	0
rs111671182	G	NA	NA	0	0	NA	0.005051	0	0	0	0.02655	0	NA	0	NA	0	0
rs181044447	A	NA	NA	0	0	NA	0	0	0	0	0	0	NA	0	NA	0	0
rs573737830	A	NA	NA	0	0	NA	0	0	0	0	0	0	NA	0	NA	0	0
rs181778800	A	NA	NA	0	0	NA	0	0	0	0	0	0	NA	0	NA	0	0
rs577217013	C	NA	NA	0	0	NA	0	0	0	0.004854	0	0	NA	0	NA	0	0
rs563948676	A	NA	NA	0	0	NA	0	0	0	0	0	0	NA	0	NA	0	0
rs141888586	T	NA	NA	0	0.0266	NA	0.1566	0	0	0	0.1372	0.004673	NA	0	NA	0	0
rs146797606	C	NA	NA	0	0	NA	0.0101	0	0	0	0.004425	0	NA	0	NA	0	0
rs544037190	C	NA	NA	0	0	NA	0	0	0	0.004854	0	0	NA	0	NA	0	0
rs535041396	T	NA	NA	0	0	NA	0	0	0	0	0	0	NA	0	NA	0.004808	0
rs534591508	T	NA	NA	0	0	NA	0	0	0	0	0	0	NA	0	NA	0	0
rs547758146	A	NA	NA	0	0	NA	0	0	0.005495	0.004854	0	0	NA	0	NA	0	0
rs529424146	A	NA	NA	0	0	NA	0	0	0	0	0	0	NA	0	NA	0	0.005051
rs563946938	T	NA	NA	0	0	NA	0	0	0	0	0.00885	0	NA	0	NA	0	0
rs545056304	A	NA	NA	0	0	NA	0	0	0	0.004854	0	0	NA	0	NA	0	0
rs12481984	C	0.5	NA	0.01429	0.3138	NA	0.2828	0.404	0.4066	0.1942	0.2478	0.3785	NA	0.1667	NA	0.01923	0.01515
rs538226704	T	NA	NA	0	0	NA	0	0	0	0	0	0	NA	0	NA	0	0
rs548678297	G	NA	NA	0	0	NA	0	0	0	0	0	0	NA	0	NA	0	0
rs149676870	T	0.5	NA	0	0.02128	NA	0	0	0	0	0	0	NA	0	NA	0	0

SNP	Allele	Chileans	Chopccas	CHS	CLM	Cusco	ESN	FIN	GBR	GIH	GWD	IBS	Iquitos	ITU	Jacarus	JPT	KHV
rs78217567	C	NA	NA	0	0.02128	NA	0	0.05556	0.02747	0.004854	0.004425	0.02804	NA	0.004902	NA	0	0.005051
rs558791980	A	NA	NA	0	0	NA	0	0	0	0	0	0	NA	0	NA	0	0
rs34256269	A	NA	NA	0.07619	0.09574	NA	0	0.2222	0.1209	0.1068	0.004425	0.1308	NA	0.1078	NA	0.04327	0.08081
rs74749793	T	NA	0	0.3048	0.133	0.01389	0.08081	0.07071	0.1044	0.1311	0.1637	0.1262	0	0.2206	0	0.2885	0.2121
rs193253965	C	NA	NA	0	0	NA	0	0	0	0	0	0	NA	0	NA	0	0
rs373622147	C	NA	NA	0	0	NA	0	0	0	0	0	0	NA	0.02941	NA	0	0.005051
rs559691670	A	NA	NA	0	0	NA	0	0	0	0	0	0	NA	0	NA	0	0
rs534317621	A	NA	NA	0	0	NA	0	0	0	0	0	0	NA	0.004902	NA	0	0
rs539981354	G	NA	NA	0	0	NA	0	0	0	0	0	0	NA	0	NA	0	0
rs115266855	G	NA	NA	0	0	NA	0.05556	0	0	0	0.01327	0	NA	0	NA	0	0
rs149109132	A	NA	NA	0	0	NA	0.0101	0	0	0	0.00885	0	NA	0	NA	0	0
rs557656729	A	NA	NA	0	0	NA	0	0	0	0	0	0	NA	0.004902	NA	0	0
rs57161767	A	NA	NA	0	0	NA	0.04545	0	0	0	0.00885	0	NA	0	NA	0	0
rs574708698	T	NA	NA	0	0	NA	0	0	0	0	0	0	NA	0	NA	0	0
rs4303795	G	0.5	NA	0.01905	0.3138	NA	0.303	0.404	0.4121	0.2087	0.2434	0.3832	NA	0.1765	NA	0.01442	0.0101
rs145662876	T	NA	NA	0	0	NA	0	0	0	0	0	0	NA	0	NA	0.01923	0.02525
rs566146941	A	NA	NA	0	0	NA	0	0	0	0	0	0	NA	0	NA	0	0
rs563595766	T	NA	NA	0	0	NA	0.005051	0	0	0	0	0	NA	0	NA	0	0
rs572507505	T	NA	NA	0.004762	0	NA	0	0	0	0	0	0	NA	0	NA	0	0
rs557606231	G	NA	NA	0	0	NA	0.005051	0	0	0	0	0	NA	0	NA	0	0
rs11911394	T	0.5	1	0.6905	0.7394	0.97222	0.3232	0.5859	0.7692	0.7864	0.3274	0.757	0.90385	0.7451	0.91176	0.6635	0.7475
rs76973757	A	NA	NA	0	0.01596	NA	0.05051	0	0	0	0.06637	0	NA	0	NA	0	0
rs573484758	G	NA	NA	0	0	NA	0	0	0	0	0	0	NA	0	NA	0	0
rs530471976	T	NA	NA	0	0	NA	0	0	0	0	0	0	NA	0	NA	0	0
rs372286621	A	NA	NA	0.004762	0	NA	0	0	0	0	0	0	NA	0	NA	0	0
rs4818239	C	0.5	NA	0	0.3777	NA	0.2172	0.4899	0.5055	0.5146	0.2743	0.5327	NA	0.4706	NA	0.004808	0.01515
rs186484871	C	NA	NA	0	0	NA	0	0	0.01099	0	0	0	NA	0	NA	0	0
rs542575868	A	NA	NA	0	0	NA	0	0	0	0	0	0	NA	0.004902	NA	0	0
rs111572592	A	NA	NA	0	0.005319	NA	0	0.02525	0.01648	0.009709	0	0.009346	NA	0.009804	NA	0	0
rs144576889	G	NA	NA	0	0	NA	0.0101	0	0	0	0.004425	0	NA	0	NA	0	0
rs572265353	A	NA	NA	0	0	NA	0	0	0	0.004854	0	0	NA	0.004902	NA	0	0
rs191368876	C	NA	NA	0.004762	0	NA	0	0	0	0	0	0	NA	0	NA	0	0
rs535472251	CAAAAAAAAAA	NA	NA	0.3095	0.2766	NA	0.3434	0.4293	0.2637	0.2379	0.2301	0.3505	NA	0.2353	NA	0.2692	0.3838
rs141788162	A	NA	NA	0	0	NA	0.005051	0.0101	0	0	0.004425	0	NA	0	NA	0	0
rs56695953	A	0.5	NA	0.05238	0.1649	NA	0.02525	0.1212	0.2253	0.1699	0.0354	0.2243	NA	0.08824	NA	0.05769	0.005051
rs548680244	T	NA	NA	0	0	NA	0	0	0	0	0.004425	0	NA	0	NA	0	0
rs430915	A	0.4	0.8235	0.4333	0.4787	0.6806	0.1212	0.4394	0.5275	0.5	0.06637	0.4907	0.7308	0.4853	0.6471	0.351	0.4848
rs564795317	A	NA	NA	0	0	NA	0	0	0	0.004854	0	0	NA	0	NA	0	0
rs577731955	C	NA	NA	0	0	NA	0	0	0	0	0	0.004673	NA	0	NA	0	0
rs369342013	A	NA	NA	0	0	NA	0	0	0	0	0	0	NA	0	NA	0	0
rs542402063	A	NA	NA	0	0	NA	0	0	0	0	0	0	NA	0	NA	0.004808	0
rs556381293	C	NA	NA	0	0	NA	0.0101	0	0	0	0	0	NA	0	NA	0	0
rs534924497	T	NA	NA	0	0.005319	NA	0	0	0	0	0	0	NA	0	NA	0	0
rs554462605	A	NA	NA	0	0	NA	0	0	0	0	0	0	NA	0	NA	0	0
rs80027429	A	NA	NA	0	0	NA	0	0	0.005495	0.009709	0	0.004673	NA	0.004902	NA	0	0

SNP	Allele	Chileans	Chopccas	CHS	CLM	Cusco	ESN	FIN	GBR	GIH	GWD	IBS	Iquitos	ITU	Jacarus	JPT	KHV
rs561192574	T	NA	NA	0	0	NA	0	0	0	0	0	0	NA	0	NA	0	0
rs455281	G	0.3	NA	0.6333	0.2074	NA	0.2374	0.04545	0.01648	0.1796	0.1504	0.01869	NA	0.2941	NA	0.4663	0.6768
rs9975782	A	NA	NA	0	0	NA	0	0	0	0	0	0	NA	0	NA	0	0
rs569362310	G	NA	NA	0	0	NA	0	0	0	0.004854	0	0	NA	0	NA	0	0
rs536278410	C	NA	NA	0	0	NA	0	0	0	0	0	0	NA	0	NA	0	0
rs415918	C	0.375	NA	0.7476	0.4202	NA	0.4242	0.4394	0.2308	0.2767	0.4071	0.2336	NA	0.3333	NA	0.7019	0.7121
rs554890599	A	NA	NA	0	0	NA	0	0	0	0	0.004425	0	NA	0	NA	0	0
rs530721239	G	NA	NA	0	0	NA	0	0	0	0	0	0	NA	0	NA	0	0
rs552589240	G	NA	NA	0	0.005319	NA	0	0	0	0	0	0	NA	0	NA	0	0
rs545504807	C	NA	NA	0	0	NA	0	0	0	0	0.004425	0	NA	0	NA	0	0
rs28403625	A	NA	NA	0	0	NA	0	0	0	0	0	0	NA	0	NA	0	0.005051
rs532159311	C	NA	NA	0	0	NA	0	0	0	0	0	0	NA	0	NA	0	0
rs543578752	G	NA	NA	0	0	NA	0	0	0	0	0	0	NA	0	NA	0	0
rs576215861	A	NA	NA	0	0	NA	0	0	0	0	0	0	NA	0.004902	NA	0	0
rs199824558	A	NA	NA	0	0	NA	0	0	0	0	0	0	NA	0.01471	NA	0	0
rs562000328	C	NA	NA	0	0	NA	0	0	0	0	0	0	NA	0	NA	0	0
rs559698510	T	NA	NA	0	0	NA	0	0	0	0	0.004425	0	NA	0	NA	0	0
rs75168613	A	NA	NA	0	0.03723	NA	0.1061	0	0	0	0.1681	0.02336	NA	0.009804	NA	0	0
rs543344542	T	NA	NA	0	0	NA	0	0	0	0	0	0	NA	0	NA	0	0
rs570889046	A	NA	NA	0	0	NA	0.01515	0	0	0	0	0	NA	0	NA	0	0
rs548267325	A	NA	NA	0	0	NA	0	0.01515	0	0	0	0	NA	0	NA	0	0
rs35899679	A	0.5	NA	0	0.3138	NA	0.101	0.3939	0.5165	0.4369	0.04867	0.486	NA	0.4069	NA	0.004808	0.01515
rs561575371	A	NA	NA	0	0	NA	0	0	0	0	0	0	NA	0.004902	NA	0	0
rs577554253	A	NA	NA	0	0	NA	0	0	0	0	0	0	NA	0	NA	0	0
rs535407146	A	NA	NA	0	0	NA	0	0	0	0	0	0	NA	0	NA	0	0
rs149024920	G	NA	NA	0	0	NA	0.005051	0	0	0	0.02655	0	NA	0	NA	0	0
rs112209215	A	NA	NA	0	0	NA	0.0303	0	0	0	0.0177	0	NA	0	NA	0	0
rs183405398	G	NA	NA	0	0	NA	0	0	0	0	0	0	NA	0	NA	0	0
rs374510753	T	NA	NA	0	0	NA	0	0	0	0	0	0	NA	0.004902	NA	0	0
rs75200570	C	NA	NA	0.04286	0.03723	NA	0.07576	0	0.02198	0.01942	0.1681	0.01869	NA	0.03922	NA	0.08173	0.09596
rs149275684	T	NA	NA	0	0	NA	0	0	0	0	0	0	NA	0	NA	0.01442	0
rs547544037	A	NA	NA	0	0	NA	0	0	0	0	0	0	NA	0.004902	NA	0	0
rs553755767	A	NA	NA	0	0	NA	0	0	0	0.01942	0	0	NA	0	NA	0	0
rs76855393	T	NA	NA	0.004762	0	NA	0	0	0	0	0	0	NA	0	NA	0	0.005051
rs118028230	C	NA	NA	NA	NA	NA	NA	NA	NA	NA	NA	NA	NA	NA	NA	NA	NA
rs184764113	A	NA	NA	0	0	NA	0	0	0	0	0	0	NA	0	NA	0	0
rs541351488	A	NA	NA	0	0	NA	0	0	0	0	0	0	NA	0	NA	0	0
rs185078457	T	NA	NA	0	0	NA	0	0.005051	0	0	0	0	NA	0	NA	0	0
rs542961770	T	NA	NA	0	0	NA	0	0	0	0	0	0	NA	0.004902	NA	0	0
rs562131885	A	NA	NA	0	0.005319	NA	0.03535	0	0	0	0.04425	0	NA	0	NA	0	0
rs536399456	A	NA	NA	0	0	NA	0.005051	0	0	0	0	0	NA	0	NA	0	0
rs547523412	A	NA	NA	0	0	NA	0.005051	0	0	0	0	0	NA	0	NA	0	0
rs534034788	A	NA	NA	0	0	NA	0	0	0	0	0	0	NA	0.004902	NA	0	0
rs544784789	G	NA	NA	0	0	NA	0	0	0	0	0	0	NA	0	NA	0	0
rs540987630	T	NA	NA	0	0	NA	0	0	0	0.004854	0	0	NA	0	NA	0	0

SNP	Allele	Chileans	Chopccas	CHS	CLM	Cusco	ESN	FIN	GBR	GIH	GWD	IBS	Iquitos	ITU	Jacarus	JPT	KHV
rs141478137	G	NA	NA	0	0	NA	0	0	0	0	0	0	NA	0	NA	0.01442	0.0101
rs5844077	G	0.1667	NA	0.3143	0.1968	NA	0.09091	0.3586	0.2527	0.2282	0.07522	0.257	NA	0.2059	NA	0.3173	0.3737
rs551681725	G	NA	NA	0	0	NA	0	0	0	0	0	0	NA	0	NA	0	0
rs548592256	C	NA	NA	0	0	NA	0	0	0	0	0	0	NA	0	NA	0	0.0101
rs568689321	A	NA	NA	0	0	NA	0	0	0	0	0	0	NA	0	NA	0	0
rs555302570	A	NA	NA	0	0	NA	0	0	0	0	0	0	NA	0	NA	0	0
rs537212420	G	NA	NA	0	0	NA	0	0	0	0	0	0	NA	0	NA	0	0
rs547186777	C	NA	NA	0	0	NA	0	0	0	0	0	0	NA	0	NA	0	0
rs532627816	A	NA	NA	0	0	NA	0	0	0	0	0.00885	0	NA	0	NA	0	0
rs150314077	A	NA	NA	0	0	NA	0.03535	0	0	0	0.01327	0	NA	0	NA	0	0
rs544983081	A	NA	NA	0	0	NA	0	0	0	0	0	0	NA	0	NA	0	0
rs140121827	T	NA	NA	0.004762	0	NA	0	0	0	0	0	0	NA	0	NA	0.004808	0
rs74605993	T	NA	NA	0	0	NA	0.02525	0	0	0	0.0177	0	NA	0	NA	0	0
rs7279603	C	0.5	0	0.2048	0.3404	0.1111	0.4646	0.2172	0.3462	0.3155	0.5088	0.3411	0.05769	0.2696	0	0.2837	0.2121
rs535611212	G	NA	NA	0	0	NA	0.005051	0	0	0	0.01327	0	NA	0	NA	0	0
rs180792570	T	NA	NA	0	0	NA	0.005051	0	0	0	0	0	NA	0	NA	0	0
rs180784757	A	NA	NA	0	0	NA	0.0101	0	0	0	0.004425	0	NA	0	NA	0	0
rs532756922	A	NA	NA	0	0.005319	NA	0	0	0	0	0	0	NA	0	NA	0	0
rs138651919	A	NA	NA	0	0	NA	0	0	0.02198	0	0	0	NA	0	NA	0	0
rs549212787	G	NA	NA	0	0	NA	0	0	0	0.004854	0	0	NA	0	NA	0	0
rs538863731	C	NA	NA	0	0	NA	0	0	0	0	0.004425	0	NA	0	NA	0	0
rs538803792	T	NA	NA	0	0	NA	0	0	0	0	0	0	NA	0	NA	0	0
rs574890845	A	NA	NA	0	0	NA	0	0.005051	0	0	0	0	NA	0	NA	0	0
rs546165791	T	NA	NA	0	0.005319	NA	0	0	0	0	0	0	NA	0	NA	0	0
rs554530868	G	NA	NA	0	0.03191	NA	0	0.06566	0.05495	0.02427	0	0.02336	NA	0.004902	NA	0	0
rs79512425	C	NA	NA	0	0	NA	0	0	0	0	0	0	NA	0	NA	0.004808	0
rs542395007	T	NA	NA	0.004762	0	NA	0	0	0.005495	0.004854	0.02212	0	NA	0	NA	0.004808	0
rs556249043	A	NA	NA	0	0	NA	0	0	0	0	0	0	NA	0	NA	0	0
rs529550811	A	NA	NA	0	0	NA	0	0	0	0	0	0	NA	0	NA	0	0
rs533931558	A	NA	NA	0	0.005319	NA	0	0	0	0	0	0	NA	0	NA	0	0
rs565902164	AACC	NA	NA	0	0	NA	0	0	0	0.009709	0	0	NA	0.009804	NA	0	0
rs530084541	G	NA	NA	0	0	NA	0	0	0	0	0	0	NA	0	NA	0	0
rs549593213	T	NA	NA	0	0	NA	0	0	0	0	0	0	NA	0	NA	0	0
rs189570078	C	NA	NA	0	0	NA	0	0	0	0	0	0	NA	0	NA	0	0
rs190682490	T	NA	NA	0	0	NA	0	0	0.005495	0	0	0	NA	0	NA	0	0
rs577570888	T	NA	NA	0	0	NA	0	0	0	0	0	0	NA	0	NA	0	0
rs376403654	A	NA	NA	0	0	NA	0.005051	0	0	0	0	0	NA	0	NA	0	0
rs576016640	T	NA	NA	0	0	NA	0	0	0	0	0	0	NA	0	NA	0	0
rs567552202	C	NA	NA	0	0	NA	0	0	0	0	0	0	NA	0	NA	0	0
rs140793040	A	NA	NA	0	0.005319	NA	0	0	0	0.009709	0	0.004673	NA	0	NA	0	0
rs146445857	TGCC	NA	NA	0.03333	0	NA	0	0	0	0	0	0	NA	0	NA	0.02885	0.005051
rs547401659	T	NA	NA	0	0.005319	NA	0	0	0	0	0	0	NA	0	NA	0	0
rs571396442	T	NA	NA	0	0	NA	0	0	0	0	0	0	NA	0	NA	0	0
rs185406661	G	NA	NA	0	0	NA	0	0	0.005495	0	0	0	NA	0	NA	0	0
rs2070789	T	0.5	NA	0.419	0.234	NA	0.4293	0.3333	0.2088	0.2039	0.4115	0.1682	NA	0.2206	NA	0.4183	0.3586

SNP	Allele	Chileans	Chopccas	CHS	CLM	Cusco	ESN	FIN	GBR	GIH	GWD	IBS	Iquitos	ITU	Jacarus	JPT	KHV
rs7277080	T	0.5	NA	0.009524	0.2926	NA	0.298	0.3687	0.3791	0.2087	0.2389	0.3178	NA	0.1667	NA	0.01442	0.01515
rs183385443	A	NA	NA	0	0	NA	0	0	0	0	0	0	NA	0	NA	0	0
rs181592444	T	NA	NA	0	0	NA	0	0	0	0	0	0	NA	0	NA	0	0.0303
rs189832305	T	NA	NA	0.01429	0	NA	0	0	0	0	0	0	NA	0	NA	0	0.01515
rs734056	A	0.5	0.08824	0	0.3511	0.1389	0.1768	0.4141	0.511	0.5049	0.1903	0.528	0.2885	0.451	0.02941	0.004808	0.01515
rs561819256	G	NA	NA	0	0	NA	0	0	0	0	0	0	NA	0	NA	0	0
rs2070786	C	0.5	0	0.2048	0.3351	0.1111	0.2929	0.2172	0.3462	0.3155	0.4071	0.3318	0.04	0.2696	0	0.2837	0.2121
rs541155775	C	NA	NA	0	0	NA	0	0	0	0	0	0	NA	0	NA	0	0.005051
rs544315388	A	NA	NA	0	0	NA	0.005051	0	0	0	0	0	NA	0	NA	0	0
rs191763249	G	NA	NA	0	0	NA	0	0	0	0	0	0	NA	0	NA	0	0
rs137962614	T	NA	NA	0	0	NA	0	0	0	0	0	0	NA	0	NA	0	0
rs464431	A	0.4	NA	0.3762	0.1649	NA	0.0101	0.04545	0.01099	0.06796	0.02655	0.004673	NA	0.09314	NA	0.2933	0.4141
rs147054075	A	NA	NA	0	0	NA	0	0	0	0.009709	0	0	NA	0	NA	0	0
rs190516010	A	NA	NA	0	0	NA	0	0	0	0	0	0	NA	0	NA	0	0
rs542265380	T	NA	NA	0	0	NA	0	0	0	0	0	0	NA	0	NA	0	0
rs188263047	A	NA	NA	0	0	NA	0	0	0	0	0	0	NA	0	NA	0	0
rs143148203	G	NA	NA	0	0	NA	0	0	0	0	0.004425	0	NA	0	NA	0	0
rs557853844	A	NA	NA	0	0	NA	0	0	0	0	0	0	NA	0	NA	0	0
rs571275590	G	NA	NA	0	0	NA	0	0	0	0	0	0	NA	0	NA	0	0
rs562173027	C	NA	NA	0	0	NA	0	0	0	0.004854	0	0	NA	0	NA	0	0
rs530253044	T	NA	NA	0	0	NA	0	0	0	0	0	0	NA	0.004902	NA	0	0
rs532694622	C	NA	NA	0	0	NA	0	0	0	0	0	0	NA	0	NA	0	0
rs75756279	T	NA	NA	0	0	NA	0	0	0.005495	0.009709	0	0.004673	NA	0.004902	NA	0	0
rs570835062	T	NA	NA	0	0	NA	0	0	0	0	0	0	NA	0	NA	0	0
rs530942388	G	NA	NA	0	0	NA	0.005051	0	0	0	0.02212	0	NA	0	NA	0	0
rs575313753	G	NA	NA	0	0	NA	0	0	0	0	0.004425	0	NA	0	NA	0	0
rs537370123	T	NA	NA	0	0	NA	0	0	0	0	0	0	NA	0	NA	0	0
rs185946013	A	NA	NA	0	0.005319	NA	0	0	0	0	0	0	NA	0	NA	0	0
rs142518005	T	NA	NA	0	0	NA	0.0101	0	0	0	0	0	NA	0	NA	0	0
rs558104085	A	NA	NA	0	0	NA	0	0	0	0	0	0	NA	0	NA	0	0
rs144800717	T	NA	NA	0	0	NA	0	0	0	0	0	0	NA	0	NA	0	0
rs57474639	T	0.5	0	0.05238	0.1011	0	0.07576	0.1919	0.1209	0.03883	0.115	0.08879	0.09615	0.05882	0.05882	0.0625	0.02525
rs538139969	T	NA	NA	0	0	NA	0	0	0	0	0	0.004673	NA	0	NA	0	0
rs567302726	A	NA	NA	0	0	NA	0	0	0	0.01456	0	0	NA	0.02451	NA	0	0
rs187052796	T	NA	NA	0	0	NA	0.0101	0	0	0	0.004425	0	NA	0	NA	0	0
rs551164492	G	NA	NA	0	0.005319	NA	0	0	0	0	0	0.009346	NA	0.004902	NA	0	0.005051
rs143562195	A	NA	NA	0	0	NA	0.0101	0	0	0	0.00885	0	NA	0	NA	0	0
rs11088550	A	0.5	NA	0	0.09574	NA	0	0.1616	0.09341	0.009709	0.004425	0.07477	NA	0.009804	NA	0	0.005051
rs151338439	A	NA	NA	0	0	NA	0	0	0	0	0	0	NA	0	NA	0	0
rs185726773	A	NA	NA	0	0	NA	0	0	0	0	0	0	NA	0	NA	0	0
rs531410451	A	NA	NA	0	0	NA	0	0	0	0	0	0	NA	0.009804	NA	0	0
rs2257202	C	0.6667	NA	0.2143	0.25	NA	0.09596	0.1465	0.2473	0.2864	0.1239	0.257	NA	0.25	NA	0.3029	0.2273
rs117827300	A	NA	NA	0	0	NA	0	0.0101	0	0	0	0	NA	0	NA	0.01442	0
rs187208295	C	NA	NA	0.02857	0	NA	0	0	0	0	0	0	NA	0	NA	0.009615	0.0101
rs61735793	A	NA	0	0	0.005319	0	0	0	0.01099	0	0	0.004673	0	0	0	0	0

SNP	Allele	Chileans	Chopccas	CHS	CLM	Cusco	ESN	FIN	GBR	GIH	GWD	IBS	Iquitos	ITU	Jacarus	JPT	KHV
rs143712818	T	NA	NA	0	0	NA	0.0101	0	0	0	0.004425	0	NA	0	NA	0	0
rs542575245	T	NA	NA	0	0	NA	0	0	0	0	0	0	NA	0	NA	0	0
rs551797163	C	NA	NA	0	0	NA	0	0	0.005495	0	0	0	NA	0	NA	0	0
rs571050655	C	NA	NA	0	0	NA	0	0	0	0	0.00885	0	NA	0	NA	0	0
rs34624090	AC	NA	NA	0	0.2606	NA	0.09596	0.4091	0.4341	0.4126	0.0531	0.486	NA	0.4118	NA	0.004808	0.01515
rs147099383	C	NA	NA	0.1286	0.04787	NA	0.0404	0.01515	0.02198	0.09223	0.02212	0.04673	NA	0.1176	NA	0.1058	0.1566
rs58146697	C	NA	0	0.0381	0.03191	0	0.3182	0.03535	0.005495	0.02913	0.2566	0.004673	0	0.07843	0	0.08173	0.06566
rs139700775	C	NA	NA	0	0	NA	0	0.0101	0	0	0	0.004673	NA	0	NA	0	0
rs560183935	C	NA	NA	0	0	NA	0.0101	0	0	0	0.004425	0	NA	0	NA	0	0
rs182330267	A	NA	NA	0	0.01064	NA	0.0101	0	0	0	0	0	NA	0	NA	0	0
rs565879862	C	NA	NA	0.004762	0	NA	0	0	0	0	0	0	NA	0	NA	0	0
rs188500480	T	NA	NA	0.004762	0	NA	0	0	0	0	0	0	NA	0	NA	0	0
rs533556786	G	NA	NA	0	0	NA	0	0	0	0	0	0	NA	0	NA	0	0
rs551106657	T	NA	NA	0	0	NA	0	0	0	0	0	0	NA	0	NA	0	0
rs116606027	A	NA	NA	0	0	NA	0.0101	0	0	0	0.004425	0	NA	0	NA	0	0
rs573715927	A	NA	NA	0	0	NA	0	0	0	0	0	0	NA	0	NA	0	0
rs145026350	T	NA	NA	0	0	NA	0	0	0	0	0	0	NA	0	NA	0.004808	0
rs915823	C	0.5	0	0.2619	0.133	0.01389	0.1212	0.3535	0.1868	0.04369	0.07965	0.1262	0.09615	0.07843	0.08824	0.2981	0.1919
rs188495261	A	NA	NA	0.004762	0	NA	0	0	0	0	0	0	NA	0	NA	0	0
rs563800355	T	NA	NA	0	0	NA	0	0	0	0.01456	0	0	NA	0.004902	NA	0	0
rs143060022	A	NA	NA	0	0	NA	0	0	0	0	0	0	NA	0	NA	0	0
rs527811791	C	NA	NA	0	0	NA	0	0	0	0	0	0	NA	0	NA	0	0
rs554692214	C	NA	NA	0	0	NA	0	0	0	0	0	0	NA	0	NA	0	0.005051
rs8129582	A	NA	NA	0	0	NA	0	0	0	0	0	0	NA	0	NA	0	0
rs532513568	C	NA	NA	0	0	NA	0	0	0	0	0	0	NA	0	NA	0	0
rs537357445	C	NA	NA	0	0	NA	0	0	0	0	0	0	NA	0	NA	0	0
rs538591883	C	NA	NA	0	0	NA	0	0	0	0	0	0	NA	0	NA	0	0
rs535599066	T	NA	NA	0	0	NA	0	0	0	0	0	0	NA	0	NA	0	0
rs7275220	G	0.5	NA	0.7429	0.4149	NA	0.5455	0.2273	0.2418	0.3301	0.5575	0.271	NA	0.3333	NA	0.7308	0.7525
rs199636550	G	NA	NA	0	0	NA	0	0	0	0	0	0	NA	0	NA	0.004808	0
rs117696554	A	NA	NA	0	0.02128	NA	0	0.005051	0.02747	0.009709	0.00885	0.02336	NA	0	NA	0	0
rs137871202	C	NA	NA	0	0	NA	0.0101	0	0	0	0	0	NA	0	NA	0	0
rs555056776	A	NA	NA	0	0	NA	0	0	0	0	0	0	NA	0	NA	0	0
rs553934496	T	NA	NA	0.004762	0	NA	0	0	0	0	0	0	NA	0	NA	0	0
rs547701911	C	NA	NA	0	0	NA	0	0	0	0	0	0	NA	0	NA	0	0
rs187460831	A	NA	NA	0	0	NA	0	0	0	0	0	0	NA	0	NA	0	0
rs422761	A	0.5909	NA	0.2762	0.1915	NA	0.3384	0.04545	0.01099	0.2524	0.3142	0.01402	NA	0.299	NA	0.3269	0.3434
rs186422083	C	NA	NA	0	0	NA	0	0	0	0	0	0	NA	0	NA	0.004808	0
rs544946928	T	NA	NA	0	0	NA	0	0	0	0.004854	0	0	NA	0.03431	NA	0	0
rs542577849	A	NA	NA	0	0	NA	0	0.005051	0	0	0	0	NA	0	NA	0	0
rs561308071	T	NA	NA	0	0	NA	0	0	0	0	0	0	NA	0.004902	NA	0	0
rs184146774	G	NA	NA	0	0	NA	0	0	0	0	0	0	NA	0	NA	0	0
rs181446489	C	NA	NA	0	0	NA	0	0	0	0	0	0	NA	0	NA	0	0
rs543518445	T	NA	NA	0	0	NA	0	0	0	0	0	0	NA	0	NA	0	0
rs566732470	A	NA	NA	0.004762	0	NA	0	0	0	0	0.004425	0	NA	0	NA	0	0

SNP	Allele	Chileans	Chopccas	CHS	CLM	Cusco	ESN	FIN	GBR	GIH	GWD	IBS	Iquitos	ITU	Jacarus	JPT	KHV
rs533571483	A	NA	NA	0	0	NA	0	0	0	0	0	0	NA	0	NA	0	0
rs531148133	A	NA	NA	0	0	NA	0	0	0	0	0	0	NA	0	NA	0	0.005051
rs113288437	G	NA	NA	0	0.03723	NA	0.1111	0	0	0	0.1947	0.02336	NA	0.009804	NA	0	0
rs115968373	A	NA	NA	0	0	NA	0	0	0	0	0	0	NA	0	NA	0	0
rs140715097	T	NA	NA	0	0	NA	0	0	0	0	0	0	NA	0	NA	0.01923	0.0101
rs376897988	T	NA	NA	0	0	NA	0	0	0	0	0	0	NA	0	NA	0	0.005051
rs574759417	A	NA	NA	0.009524	0	NA	0	0	0	0	0	0	NA	0	NA	0	0
rs528568210	A	NA	NA	0	0	NA	0	0	0	0	0	0	NA	0	NA	0	0
rs540209049	C	NA	NA	0	0	NA	0	0	0	0	0	0	NA	0	NA	0	0
rs191595416	A	NA	NA	0	0.005319	NA	0	0	0	0	0	0	NA	0	NA	0	0
rs66492316	C	NA	NA	0.01905	0.3138	NA	0.303	0.404	0.4176	0.2087	0.2389	0.3832	NA	0.1765	NA	0.01442	0.0101
rs8126497	A	0.5	NA	0.05714	0.1649	NA	0	0.1212	0.2308	0.1699	0.03097	0.215	NA	0.08824	NA	0.05769	0.005051
rs531884709	A	NA	NA	0	0	NA	0	0	0	0	0	0	NA	0	NA	0	0
rs547558669	G	NA	NA	0	0	NA	0	0	0	0	0	0.004673	NA	0	NA	0	0
rs55704664	T	0.5	NA	0.05714	0.1649	NA	0	0.1212	0.2308	0.1699	0.03097	0.2196	NA	0.08824	NA	0.05769	0.005051
rs546388820	T	NA	NA	0	0	NA	0	0	0	0	0.004425	0	NA	0	NA	0	0
rs142103907	G	NA	NA	0	0	NA	0	0	0	0	0	0	NA	0	NA	0	0
rs117656646	C	NA	NA	0	0.005319	NA	0.005051	0	0.01648	0	0	0.009346	NA	0	NA	0	0
rs139258152	T	NA	NA	0	0	NA	0	0	0	0	0	0	NA	0	NA	0	0
rs150382508	C	NA	NA	0	0	NA	0.0101	0	0	0	0.004425	0	NA	0	NA	0	0
rs544308006	T	NA	NA	0	0	NA	0	0	0	0.004854	0	0	NA	0	NA	0	0
rs551100726	T	NA	NA	0	0	NA	0	0	0	0	0	0	NA	0	NA	0	0
rs527435310	A	NA	NA	0	0	NA	0	0	0	0	0	0	NA	0	NA	0	0
rs552725417	A	NA	NA	0	0	NA	0	0	0	0	0	0	NA	0	NA	0	0
rs549038772	T	NA	NA	0	0	NA	0	0	0	0	0	0	NA	0.004902	NA	0	0
rs562574628	G	NA	NA	0	0	NA	0	0	0	0	0	0	NA	0	NA	0	0
rs8129713	C	0.5	0.02941	0.05238	0.1649	0.2639	0.0303	0.1212	0.2253	0.1796	0.0354	0.2196	0.25	0.09314	0.09375	0.05769	0.005051
rs183385542	C	NA	NA	0	0	NA	0	0.0101	0	0	0	0	NA	0	NA	0	0
rs553596811	G	NA	NA	0	0	NA	0	0	0	0	0	0	NA	0	NA	0	0
rs150473664	C	NA	NA	0	0	NA	0	0	0	0	0	0	NA	0	NA	0.02404	0
rs146120690	A	NA	NA	0.02857	0	NA	0	0	0	0	0	0	NA	0	NA	0.009615	0.005051
rs527261679	A	NA	NA	0	0	NA	0	0	0	0	0	0	NA	0	NA	0	0.005051
rs570544092	T	NA	NA	0	0	NA	0	0	0	0	0	0.004673	NA	0	NA	0	0
rs392370	C	0.6667	NA	0.2714	0.266	NA	0.4596	0.1515	0.2418	0.2816	0.4115	0.271	NA	0.2451	NA	0.3462	0.2778
rs150875127	C	NA	NA	0.2143	0.5372	NA	0.4091	0.5808	0.7582	0.7573	0.5354	0.7617	NA	0.7059	NA	0.2885	0.2525
rs536039173	T	NA	NA	0	0	NA	0	0	0	0.02913	0	0	NA	0.03922	NA	0	0
rs192019778	A	NA	NA	0	0	NA	0	0.005051	0	0	0	0	NA	0	NA	0	0
SNP	A1	NA	MAF	MAF	MAF	MAF	MAF	MAF	MAF	MAF	MAF	MAF	MAF	MAF	MAF	MAF	MAF
rs537728755	A	NA	NA	0	0	NA	0.005051	0	0	0	0.03982	0	NA	0	NA	0	0
rs55964536	T	NA	NA	0	0.3032	NA	0.09596	0.4293	0.5055	0.4612	0.04867	0.5	NA	0.4167	NA	0.004808	0.01515
rs373311004	C	NA	NA	0	0	NA	0	0	0	0	0	0	NA	0	NA	0	0.005051
rs575968857	T	NA	NA	0	0	NA	0	0	0	0	0	0.004673	NA	0	NA	0	0
rs548402221	G	NA	NA	0	0.01596	NA	0	0.005051	0.01099	0.004854	0	0.01402	NA	0	NA	0	0
rs140037718	C	NA	NA	0	0	NA	0	0	0	0	0	0	NA	0	NA	0	0
rs75373173	C	NA	0	0.004762	0.01596	0.01389	0	0.1465	0.1099	0.02427	0	0.08879	0	0.01471	0.02941	0	0

SNP	Allele	Chileans	Chopccas	CHS	CLM	Cusco	ESN	FIN	GBR	GIH	GWD	IBS	Iquitos	ITU	Jacarus	JPT	KHV
rs568619174	C	NA	NA	0	0	NA	0	0	0	0.004854	0	0	NA	0	NA	0	0
rs187175193	T	NA	NA	0	0.01596	NA	0	0	0	0	0	0	NA	0	NA	0	0
rs193122175	T	NA	NA	0	0.005319	NA	0	0	0	0	0	0.004673	NA	0	NA	0	0
rs117898838	A	NA	NA	0.004762	0	NA	0.005051	0	0	0	0.02655	0	NA	0	NA	0.02885	0.0101
rs537989379	A	NA	NA	0	0	NA	0	0	0	0	0	0	NA	0	NA	0	0
rs570302987	A	NA	NA	0	0	NA	0	0	0.005495	0	0	0	NA	0	NA	0	0
rs190970011	T	NA	NA	0	0	NA	0	0	0	0	0	0	NA	0	NA	0	0
rs556994660	C	NA	NA	0	0	NA	0	0	0	0	0	0	NA	0	NA	0	0
rs527380714	C	NA	NA	0	0	NA	0	0	0	0	0	0	NA	0	NA	0	0
rs536026892	T	NA	NA	0	0	NA	0	0	0	0	0	0	NA	0	NA	0	0
rs535103772	T	NA	NA	0	0	NA	0	0	0	0	0	0	NA	0	NA	0	0
rs191587330	C	NA	NA	0	0	NA	0	0	0	0	0	0	NA	0	NA	0.004808	0
rs553617111	T	NA	NA	0	0	NA	0	0	0	0	0.00885	0	NA	0	NA	0	0
rs182838808	A	NA	NA	0	0	NA	0	0	0	0	0	0	NA	0	NA	0	0
rs78503214	T	NA	NA	0	0.01064	NA	0.0202	0	0	0	0.0177	0	NA	0	NA	0	0
rs142425263	A	NA	NA	0	0	NA	0	0	0	0	0	0	NA	0	NA	0	0
rs573213706	A	NA	NA	0	0	NA	0	0.0101	0	0	0	0	NA	0	NA	0	0
rs73357663	C	NA	NA	0	0	NA	0.01515	0	0	0	0.00885	0.004673	NA	0	NA	0	0
rs563670115	C	NA	NA	0	0	NA	0	0	0	0	0	0.01402	NA	0	NA	0	0
rs111220509	G	NA	NA	0.2048	0.6064	NA	0.3838	0.6111	0.7912	0.7718	0.5044	0.8131	NA	0.6961	NA	0.2885	0.2273
rs186605876	T	NA	NA	0	0	NA	0	0	0	0	0	0	NA	0	NA	0	0
rs373611430	G	NA	NA	0	0	NA	0	0	0	0	0	0	NA	0	NA	0	0.0101
rs2094881	C	0.5	0.02941	0.2048	0.6011	0.1667	0.404	0.6162	0.7802	0.7573	0.5	0.8084	0.2115	0.6912	0.2059	0.2885	0.2273
rs73372166	A	0.5	NA	0.05238	0.1064	NA	0.2879	0.1919	0.1209	0.04369	0.2655	0.09813	NA	0.06373	NA	0.0625	0.0303
rs76135088	G	NA	0	0	0	0	0.06061	0	0	0	0.09735	0	0	0.009804	0	0	0
rs548979551	G	NA	NA	0	0.005319	NA	0	0	0.01099	0	0	0	NA	0	NA	0	0
rs540046047	C	NA	NA	0	0	NA	0	0	0	0	0	0	NA	0	NA	0	0
rs2410429	A	0.5	NA	0.6238	0.4149	NA	0.2172	0.1919	0.2637	0.3495	0.3186	0.271	NA	0.3382	NA	0.601	0.6667
rs532950488	A	NA	NA	0	0	NA	0	0	0	0	0	0	NA	0	NA	0	0
rs535531364	C	NA	NA	0	0	NA	0.01515	0	0	0	0.00885	0	NA	0	NA	0	0
rs550091217	T	NA	NA	0	0	NA	0	0	0	0	0	0	NA	0.004902	NA	0	0
rs62217527	T	NA	NA	0	0.06915	NA	0.0202	0.05051	0.1044	0.06311	0.0354	0.1308	NA	0.02451	NA	0	0
rs80275470	A	NA	NA	0	0.01064	NA	0	0.0404	0.03846	0.009709	0	0.04206	NA	0.004902	NA	0	0
rs545165511	A	NA	NA	0	0	NA	0	0	0	0	0	0	NA	0	NA	0	0
rs144318842	A	NA	NA	0	0	NA	0.005051	0	0	0	0.00885	0	NA	0	NA	0	0
rs200395836	T	NA	NA	0	0	NA	0	0	0	0	0	0	NA	0	NA	0	0
rs548552862	C	NA	NA	0	0	NA	0	0	0	0	0	0	NA	0	NA	0	0
rs577632959	A	NA	NA	0	0	NA	0	0	0	0	0	0	NA	0	NA	0	0
rs567258163	A	NA	NA	0	0	NA	0	0	0	0.02427	0	0	NA	0.01471	NA	0	0
rs561789442	A	NA	NA	0	0	NA	0	0	0	0	0	0	NA	0	NA	0	0.005051
rs538588854	A	NA	NA	0	0	NA	0	0	0	0	0	0	NA	0	NA	0	0.005051
rs115429336	A	NA	NA	0	0	NA	0.0303	0	0	0	0.02212	0	NA	0	NA	0	0
rs537412369	T	NA	NA	0.004762	0	NA	0	0	0	0	0	0	NA	0	NA	0	0
rs371531071	T	NA	NA	0	0	NA	0	0	0	0	0	0	NA	0	NA	0	0.005051
rs183141812	A	NA	NA	0	0	NA	0	0	0.005495	0	0	0.004673	NA	0	NA	0	0

SNP	Allele	Chileans	Chopccas	CHS	CLM	Cusco	ESN	FIN	GBR	GIH	GWD	IBS	Iquitos	ITU	Jacarus	JPT	KHV
rs189546747	T	NA	NA	0	0.005319	NA	0	0	0	0	0	0	NA	0	NA	0	0
rs3787950	C	NA	0	0.1381	0.04255	0	0.197	0.03535	0.07143	0.2816	0.1858	0.1121	0	0.2549	0	0.1346	0.2121
rs549067055	A	NA	NA	0	0	NA	0	0	0	0	0	0	NA	0.004902	NA	0	0
rs576955441	A	NA	NA	0	0	NA	0	0	0	0	0	0	NA	0	NA	0	0.005051
rs8134203	T	0.5714	NA	0.2048	0.6064	NA	0.4192	0.601	0.7747	0.7573	0.5487	0.8084	NA	0.6912	NA	0.3125	0.2222
rs532216261	C	NA	NA	0	0	NA	0	0	0	0	0	0	NA	0	NA	0	0
rs557766326	T	NA	NA	0	0	NA	0	0	0	0	0	0	NA	0	NA	0	0
rs546531769	G	NA	NA	0	0	NA	0	0	0	0	0	0	NA	0	NA	0	0
rs571993620	A	NA	NA	0	0	NA	0	0	0	0	0.004425	0	NA	0	NA	0	0
rs374261644	C	NA	NA	0	0	NA	0	0	0	0	0	0	NA	0	NA	0	0
rs564210410	A	NA	NA	0	0	NA	0.005051	0	0	0	0	0	NA	0	NA	0	0
rs562387776	C	NA	NA	0	0	NA	0	0	0	0	0	0	NA	0	NA	0	0
rs562587205	T	NA	NA	0	0	NA	0	0	0	0	0	0	NA	0.004902	NA	0	0
rs542538936	T	NA	NA	0	0.005319	NA	0	0	0	0	0	0	NA	0	NA	0	0
rs143818732	C	NA	NA	0	0	NA	0	0	0.005495	0.009709	0	0.004673	NA	0	NA	0	0
rs183650725	A	NA	NA	0.004762	0	NA	0	0	0	0	0	0	NA	0	NA	0	0
rs531439352	A	NA	NA	0	0	NA	0	0	0	0	0	0.004673	NA	0	NA	0	0
rs531183955	T	NA	NA	0	0	NA	0	0	0	0	0	0	NA	0	NA	0	0
rs144192191	GGTGA	NA	NA	0.2238	0.2979	NA	0.2778	0.1818	0.2692	0.3058	0.4204	0.3084	NA	0.2745	NA	0.2837	0.2121
rs149708827	T	NA	NA	0.004762	0	NA	0	0	0	0	0	0	NA	0	NA	0.009615	0.01515
rs552635816	G	NA	NA	0	0	NA	0	0	0	0	0	0	NA	0	NA	0	0
rs539520259	T	NA	NA	0	0	NA	0	0	0	0	0	0	NA	0.004902	NA	0	0
rs376235035	T	NA	NA	0	0	NA	0	0.0101	0	0	0	0	NA	0	NA	0	0
rs142303004	T	NA	NA	0	0	NA	0.005051	0	0	0	0	0	NA	0	NA	0	0
rs546232086	G	NA	NA	0	0	NA	0	0	0	0	0	0	NA	0	NA	0	0
rs549832756	C	NA	NA	0	0	NA	0	0	0	0	0	0	NA	0	NA	0.004808	0
rs75655573	G	NA	0	0	0	0	0	0	0	0	0	0	0	0	0	0	0
rs553167698	T	NA	NA	0	0	NA	0	0	0	0	0	0	NA	0	NA	0	0
rs190685013	A	NA	NA	0	0	NA	0	0	0	0	0.02212	0	NA	0	NA	0	0
rs559934921	G	NA	NA	0	0	NA	0	0	0.005495	0	0	0	NA	0	NA	0	0
rs190899605	C	NA	NA	0	0	NA	0	0.03535	0	0	0	0	NA	0.01471	NA	0	0
rs391099	C	0.6667	NA	0.2714	0.266	NA	0.4596	0.1515	0.2418	0.2816	0.4159	0.271	NA	0.2451	NA	0.3462	0.2778
rs372422423	A	NA	NA	0	0	NA	0.01515	0	0	0	0.00885	0	NA	0	NA	0	0
rs77014365	A	NA	NA	0	0	NA	0	0	0	0	0	0	NA	0	NA	0	0
rs562157895	A	NA	NA	0	0	NA	0	0	0	0	0	0	NA	0	NA	0	0
rs187290362	G	NA	NA	0	0.005319	NA	0	0	0	0	0	0.004673	NA	0	NA	0	0
rs182543256	A	NA	NA	0	0.005319	NA	0	0	0	0	0.01327	0.004673	NA	0	NA	0	0
rs563186329	C	NA	NA	0	0.005319	NA	0	0	0	0	0	0	NA	0	NA	0.009615	0
rs569026066	T	NA	NA	0	0	NA	0.005051	0	0	0	0	0	NA	0	NA	0	0
rs116511699	T	NA	NA	0	0	NA	0.0101	0	0	0	0	0	NA	0	NA	0	0
rs148499598	C	NA	NA	0	0	NA	0	0	0.005495	0	0	0	NA	0	NA	0	0
rs552256264	T	NA	NA	0	0	NA	0	0	0	0	0	0	NA	0	NA	0	0
rs562776963	A	NA	NA	0	0	NA	0.005051	0	0	0	0	0	NA	0	NA	0	0
rs79243099	A	NA	NA	0	0	NA	0.03535	0	0	0	0.09292	0.004673	NA	0	NA	0	0
rs79397218	A	NA	NA	0	0	NA	0	0	0	0	0	0	NA	0.01471	NA	0	0

SNP	Allele	Chileans	Chopccas	CHS	CLM	Cusco	ESN	FIN	GBR	GIH	GWD	IBS	Iquitos	ITU	Jacarus	JPT	KHV
rs138094318	T	NA	NA	0	0	NA	0	0	0	0	0	0	NA	0	NA	0.004808	0
rs567275769	T	NA	NA	0	0	NA	0	0	0	0	0.004425	0	NA	0	NA	0	0
rs544297320	T	NA	NA	0	0	NA	0	0	0	0	0	0	NA	0	NA	0	0
rs561404433	G	NA	NA	0	0	NA	0	0	0	0	0	0	NA	0.004902	NA	0	0
rs573923452	A	NA	NA	0	0	NA	0	0	0	0	0	0	NA	0	NA	0	0
rs539858204	A	NA	NA	0	0	NA	0	0	0	0	0	0	NA	0	NA	0	0
rs9305745	T	0.5	NA	0.2905	0.25	NA	0.3535	0.4091	0.2308	0.2136	0.3496	0.229	NA	0.2549	NA	0.3077	0.2475
rs546510868	T	NA	NA	0	0	NA	0	0	0	0	0	0.004673	NA	0	NA	0	0
rs183418223	A	NA	NA	0	0	NA	0.005051	0	0	0	0	0	NA	0	NA	0	0
rs573692135	T	NA	NA	0	0	NA	0	0	0	0	0	0	NA	0	NA	0	0
rs570126729	A	NA	NA	0	0	NA	0	0	0	0	0	0	NA	0.004902	NA	0	0
rs542946711	G	NA	NA	0	0	NA	0	0	0	0.01942	0	0	NA	0.02941	NA	0	0
rs551277697	G	NA	NA	0	0.01064	NA	0	0	0	0	0	0	NA	0	NA	0	0
rs73230088	A	NA	NA	0.07619	0.07979	NA	0.005051	0.2222	0.1209	0.09223	0.00885	0.1262	NA	0.09314	NA	0.03846	0.08081
rs548025657	A	NA	NA	0	0	NA	0	0	0	0	0	0	NA	0	NA	0	0
rs140428704	T	NA	NA	0	0	NA	0	0	0	0	0	0	NA	0	NA	0	0
rs184149889	C	NA	NA	0	0	NA	0	0	0	0	0	0	NA	0	NA	0	0
rs116568213	G	NA	NA	0	0.005319	NA	0.0303	0	0	0	0.04867	0	NA	0	NA	0	0
rs187742976	A	NA	NA	0	0.005319	NA	0	0	0	0	0	0	NA	0	NA	0	0
rs555769183	C	NA	NA	0	0	NA	0	0	0	0	0	0	NA	0	NA	0	0
rs12627374	T	NA	0	0.1619	0.01596	0	0	0.005051	0	0.08252	0	0	0	0.1275	0	0.1106	0.1667
rs565973484	A	NA	NA	0	0	NA	0	0	0	0	0	0	NA	0	NA	0	0
rs547257017	C	NA	NA	0	0	NA	0	0	0	0	0	0	NA	0	NA	0	0
rs568737536	A	NA	NA	0	0	NA	0	0	0	0	0	0	NA	0	NA	0	0
rs541239376	T	NA	NA	0	0	NA	0	0	0	0	0	0	NA	0	NA	0	0
rs557500448	A	NA	NA	0	0	NA	0	0	0	0	0	0	NA	0	NA	0	0.005051
rs185166990	A	NA	NA	0.004762	0	NA	0	0	0	0	0	0	NA	0	NA	0.009615	0.005051
rs541215881	A	NA	NA	0	0	NA	0	0	0	0	0	0	NA	0	NA	0	0.005051
rs138995130	A	NA	NA	0	0	NA	0	0	0	0	0	0	NA	0	NA	0.004808	0
rs462448	T	0.4	NA	0.3762	0.1649	NA	0.005051	0.0404	0.01099	0.06796	0	0.004673	NA	0.09314	NA	0.2933	0.4141
rs569303172	T	NA	NA	0	0	NA	0	0	0	0	0	0	NA	0.009804	NA	0	0
rs564691729	C	NA	NA	0	0	NA	0.005051	0	0	0	0	0	NA	0	NA	0	0
rs187452528	C	NA	NA	0	0	NA	0	0	0	0	0	0	NA	0	NA	0	0
rs4818242	T	0.5	NA	0	0.03191	NA	0	0	0	0	0	0	NA	0	NA	0	0
rs183016576	T	NA	NA	0	0	NA	0	0	0	0	0	0	NA	0	NA	0.004808	0
rs539934676	A	NA	NA	0	0.005319	NA	0	0	0	0	0	0	NA	0	NA	0	0
rs184876485	G	NA	NA	0	0	NA	0	0	0	0	0	0	NA	0	NA	0	0
rs549676267	T	NA	NA	0	0	NA	0	0	0	0.004854	0	0	NA	0	NA	0	0
rs150633108	T	NA	NA	0	0	NA	0	0	0.005495	0.03883	0	0	NA	0.04902	NA	0	0.005051
rs185103560	A	NA	NA	0	0	NA	0	0	0	0	0	0	NA	0	NA	0.004808	0
rs532655542	G	NA	NA	0.004762	0	NA	0	0	0	0	0	0	NA	0	NA	0	0
rs180831387	A	NA	NA	0	0	NA	0	0	0	0	0	0	NA	0	NA	0	0
rs569577322	T	0.5	NA	0	0	NA	0	0	0	0	0	0	NA	0	NA	0	0
rs144154504	C	NA	NA	0	0	NA	0	0	0.01099	0	0	0.004673	NA	0	NA	0	0
rs556937466	T	NA	NA	0	0	NA	0	0	0	0	0	0	NA	0	NA	0.004808	0

SNP	Allele	Chileans	Chopccas	CHS	CLM	Cusco	ESN	FIN	GBR	GIH	GWD	IBS	Iquitos	ITU	Jacarus	JPT	KHV
rs115129572	T	NA	0	0	0	0	0.0202	0	0	0	0.03982	0	0	0	0	0	0
rs578015897	T	NA	NA	0	0	NA	0	0	0	0	0	0	NA	0	NA	0	0
rs2298661	A	0.5	NA	0.4238	0.2128	NA	0.2323	0.3434	0.2088	0.1748	0.2212	0.1776	NA	0.2206	NA	0.4183	0.3535
rs557088853	T	NA	NA	0	0	NA	0	0	0	0	0	0	NA	0	NA	0	0
rs141301979	C	NA	NA	0	0	NA	0	0	0	0	0	0	NA	0	NA	0	0
rs8131649	T	0.4286	0.91176	0.7952	0.3936	0.8056	0.4949	0.399	0.2198	0.2427	0.385	0.1916	0.6731	0.3088	0.7941	0.6875	0.7778
rs539860607	G	NA	NA	0.009524	0	NA	0	0	0	0	0	0	NA	0	NA	0	0
rs4816720	C	0.375	NA	0.4143	0.1702	NA	0.2576	0.04545	0.01099	0.06311	0.2168	0.009346	NA	0.07843	NA	0.2981	0.4444
rs11702475	T	0.5	NA	0	0.3511	NA	0.1061	0.4141	0.5275	0.5049	0.09735	0.5187	NA	0.4412	NA	0.004808	0.0202
rs150205743	A	NA	NA	0	0	NA	0.005051	0	0	0	0.03982	0	NA	0	NA	0	0
rs8127674	G	0.5	0.02941	0.01905	0.3298	0.09722	0.3232	0.3838	0.4011	0.1845	0.2566	0.3832	0.1346	0.1618	0.2353	0.01923	0.01515
rs190134102	A	NA	NA	0	0	NA	0	0	0	0	0	0	NA	0	NA	0	0
rs141324737	T	NA	NA	0	0	NA	0	0	0	0	0	0	NA	0	NA	0	0
rs560898303	CA	NA	NA	0	0	NA	0	0	0	0	0	0	NA	0.009804	NA	0	0
rs552105586	T	NA	NA	0	0	NA	0	0	0	0	0	0	NA	0	NA	0	0.005051
rs146052428	T	NA	NA	0	0	NA	0.0202	0	0	0	0.0177	0	NA	0	NA	0	0
rs188241223	T	NA	NA	0.004762	0	NA	0	0	0	0	0	0	NA	0	NA	0	0
rs547639377	G	NA	NA	0	0	NA	0	0	0	0	0	0	NA	0	NA	0	0
rs420737	G	0.5455	NA	0.3048	0.1915	NA	0.3333	0.04545	0.01099	0.2524	0.3097	0.01402	NA	0.299	NA	0.3317	0.3586
rs534978728	G	NA	NA	0	0	NA	0	0	0	0	0	0	NA	0	NA	0	0
rs369948528	T	NA	NA	0	0	NA	0	0	0	0	0	0	NA	0	NA	0	0
rs533867263	A	NA	NA	0	0	NA	0.01515	0	0	0	0.00885	0	NA	0	NA	0	0
rs555525436	G	NA	NA	0	0	NA	0	0	0	0	0	0	NA	0	NA	0	0
rs531121505	T	NA	NA	0	0	NA	0	0	0	0	0	0	NA	0	NA	0	0
rs376143876	A	NA	NA	0	0	NA	0	0	0	0	0	0	NA	0	NA	0	0.0101
rs554986236	T	NA	NA	0	0	NA	0	0	0	0	0	0	NA	0	NA	0	0
rs66575656	T	0.5	NA	0.2048	0.2606	NA	0.202	0.1818	0.2747	0.2913	0.2788	0.2804	NA	0.2598	NA	0.2837	0.2121
rs542746297	T	NA	NA	0	0	NA	0	0	0	0	0	0	NA	0	NA	0	0
rs567175997	G	NA	NA	0	0	NA	0	0	0	0	0	0	NA	0	NA	0	0
rs578020496	C	NA	NA	0	0	NA	0	0	0	0.004854	0	0	NA	0.009804	NA	0	0
rs543607180	C	NA	NA	0	0	NA	0	0	0	0.004854	0	0	NA	0	NA	0	0
rs115849825	A	NA	NA	0	0	NA	0.01515	0	0	0	0	0	NA	0	NA	0	0
rs542333608	T	NA	NA	0	0	NA	0	0	0	0	0	0	NA	0	NA	0	0
rs34983238	C	0.5	NA	0	0.07979	NA	0.03535	0.09596	0.1099	0.1068	0.06637	0.09813	NA	0.07353	NA	0	0
rs372733383	T	NA	NA	0	0	NA	0	0	0	0	0.00885	0	NA	0	NA	0	0
rs555134126	T	NA	NA	0	0	NA	0	0	0	0	0	0	NA	0.009804	NA	0	0.005051
rs533043037	A	NA	NA	0	0	NA	0	0	0	0	0	0	NA	0	NA	0	0
rs73372168	A	NA	NA	0	0	NA	0.0202	0	0	0	0.004425	0	NA	0	NA	0	0
rs150715708	T	NA	NA	0	0	NA	0	0	0	0	0	0	NA	0	NA	0	0
rs145024812	A	NA	NA	0	0	NA	0.0101	0	0	0	0.004425	0	NA	0	NA	0	0
rs557592331	C	NA	NA	0	0	NA	0	0	0	0.004854	0	0	NA	0.009804	NA	0	0
rs567380110	T	NA	NA	0	0	NA	0	0	0	0	0	0	NA	0	NA	0	0
rs560408119	A	NA	NA	0	0	NA	0	0	0	0	0	0	NA	0	NA	0	0
rs117432315	A	NA	NA	0.009524	0	NA	0	0	0	0	0	0	NA	0	NA	0.009615	0
rs370654859	C	NA	NA	0	0	NA	0.0101	0	0	0	0.00885	0	NA	0	NA	0	0

SNP	Allele	Chileans	Chopccas	CHS	CLM	Cusco	ESN	FIN	GBR	GIH	GWD	IBS	Iquitos	ITU	Jacarus	JPT	KHV
rs139181746	C	NA	NA	0	0	NA	0	0	0	0	0	0	NA	0	NA	0	0
rs554175681	G	NA	NA	0	0	NA	0	0.01515	0	0	0	0	NA	0	NA	0	0
rs28548447	A	0.5	NA	0.2048	0.3245	NA	0.202	0.2071	0.3352	0.3155	0.2832	0.3271	NA	0.2598	NA	0.2837	0.2121
rs191526916	T	NA	NA	0	0	NA	0	0.02525	0	0	0	0	NA	0	NA	0	0
rs192959767	T	NA	NA	0	0	NA	0	0.005051	0	0	0	0	NA	0	NA	0	0
rs534192391	T	NA	NA	0	0	NA	0	0	0	0	0	0	NA	0	NA	0	0
rs571211518	C	NA	NA	0	0	NA	0	0	0	0	0	0	NA	0	NA	0	0
rs562033822	A	NA	NA	0	0	NA	0	0	0	0	0	0	NA	0	NA	0.004808	0
rs142194573	AG	NA	NA	0.004762	0	NA	0	0	0	0	0	0	NA	0	NA	0.02404	0
rs149695119	T	NA	NA	0	0.005319	NA	0.2121	0	0	0	0.1504	0.009346	NA	0	NA	0	0
rs143270468	A	NA	NA	0	0.005319	NA	0	0.005051	0.005495	0	0	0.01402	NA	0	NA	0	0
rs534423780	A	NA	NA	0	0	NA	0	0	0	0	0	0	NA	0	NA	0	0
rs535545388	G	NA	NA	0	0	NA	0	0	0	0	0	0	NA	0	NA	0	0
rs528206157	T	NA	NA	0	0	NA	0	0	0	0.02913	0	0	NA	0	NA	0	0
rs112985398	C	NA	NA	0	0	NA	0.03535	0	0	0	0.04425	0	NA	0	NA	0	0
rs77511690	A	NA	NA	0	0	NA	0.0404	0	0	0	0	0	NA	0	NA	0	0
rs538161583	A	NA	NA	0	0	NA	0	0	0	0.009709	0	0	NA	0	NA	0.004808	0
rs552591104	C	NA	NA	0	0	NA	0	0	0	0	0	0.004673	NA	0	NA	0	0
rs557224603	A	NA	NA	0	0	NA	0	0	0	0	0	0	NA	0	NA	0	0.005051
rs545825254	G	NA	NA	0	0	NA	0	0	0	0	0	0	NA	0	NA	0	0
rs185312677	T	NA	NA	0	0	NA	0	0	0	0	0	0	NA	0	NA	0	0
rs423596	T	NA	NA	0.2143	0.0266	NA	0	0.0303	0.02747	0.1796	0	0.03738	NA	0.1961	NA	0.2308	0.2424
rs541726793	A	NA	NA	0	0	NA	0	0	0	0	0	0	NA	0	NA	0	0
rs62217529	T	NA	NA	0	0	NA	0	0.005051	0.005495	0	0	0	NA	0	NA	0	0
rs528452414	C	NA	NA	0	0.01064	NA	0	0.01515	0	0	0	0.004673	NA	0	NA	0	0
rs143167369	T	NA	NA	0	0	NA	0.005051	0	0	0	0	0	NA	0	NA	0	0
rs138164084	T	NA	NA	0	0	NA	0	0	0	0	0.004425	0	NA	0	NA	0	0
rs62217531	T	0.5	0.08824	0	0.3245	0.125	0.3535	0.4141	0.5165	0.4369	0.3053	0.5047	0.25	0.4069	0.02941	0.004808	0.02525
rs528205318	G	NA	NA	0	0	NA	0	0	0	0	0	0	NA	0	NA	0	0
rs554097496	A	NA	NA	0	0	NA	0.005051	0	0	0	0	0	NA	0	NA	0	0
rs61728255	A	0.5	NA	0.3619	0.1649	NA	0.005051	0.04545	0.01099	0.06796	0	0.004673	NA	0.09314	NA	0.2885	0.4091
rs538022064	C	NA	NA	0	0	NA	0	0	0	0	0	0	NA	0	NA	0	0
rs9980225	C	0.5	NA	0.05238	0.09574	NA	0	0.2071	0.1264	0.03398	0.004425	0.08411	NA	0.05392	NA	0.07212	0.02525
rs542530281	C	NA	NA	0	0	NA	0	0	0.005495	0	0	0	NA	0	NA	0	0
rs542407537	A	NA	NA	0	0	NA	0	0	0.005495	0	0	0	NA	0	NA	0	0
rs548552823	A	NA	NA	0	0	NA	0	0	0	0	0	0	NA	0	NA	0	0
rs565903681	C	NA	NA	0	0	NA	0	0	0	0	0	0	NA	0	NA	0	0
rs201109436	G	NA	NA	0	0	NA	0	0	0	0	0	0	NA	0	NA	0	0
rs577393745	T	NA	NA	0	0	NA	0	0	0	0	0	0	NA	0	NA	0	0
rs185086499	T	NA	NA	0	0	NA	0	0	0	0	0	0	NA	0	NA	0	0
rs141230106	T	NA	NA	0	0	NA	0	0	0	0	0	0	NA	0	NA	0.009615	0
rs531548362	C	NA	NA	0	0	NA	0	0	0	0	0	0	NA	0	NA	0	0
rs150445636	A	0.5	NA	0	0	NA	0	0	0	0	0	0	NA	0	NA	0	0
rs139015396	T	NA	NA	0	0	NA	0.0101	0	0	0	0.004425	0	NA	0	NA	0	0
rs183722985	A	NA	NA	0	0	NA	0.005051	0	0	0	0	0	NA	0	NA	0	0

SNP	Allele	Chileans	Chopccas	CHS	CLM	Cusco	ESN	FIN	GBR	GIH	GWD	IBS	Iquitos	ITU	Jacarus	JPT	KHV
rs543734531	A	NA	NA	0	0	NA	0	0	0	0	0	0	NA	0	NA	0	0
rs553875292	T	NA	NA	0	0	NA	0.005051	0	0	0	0	0	NA	0	NA	0	0
rs55760462	C	0.5	0.02941	0.004762	0.2128	0.09722	0.2222	0.2576	0.2582	0.08738	0.1726	0.2056	0.1538	0.08824	0.2059	0.01442	0.01515
rs61735790	C	NA	NA	0	0	NA	0.0101	0	0	0	0	0	NA	0	NA	0	0
rs566918037	G	NA	NA	0	0	NA	0	0	0	0	0	0	NA	0	NA	0	0
rs555820997	G	NA	NA	0	0	NA	0.005051	0	0	0	0	0	NA	0	NA	0	0
rs573493693	A	NA	NA	0	0	NA	0	0	0	0.004854	0	0	NA	0	NA	0	0
rs914184	A	NA	NA	0.04762	0	NA	0	0	0	0	0	0	NA	0	NA	0.03365	0.04545
rs539019494	C	NA	NA	0	0	NA	0	0	0	0	0	0	NA	0	NA	0	0
rs544036587	T	NA	NA	0	0	NA	0.005051	0	0	0	0	0	NA	0	NA	0	0
rs565896303	A	NA	NA	0.004762	0	NA	0	0	0	0	0	0	NA	0	NA	0	0
rs192793047	C	NA	NA	0	0	NA	0	0	0.005495	0	0	0	NA	0	NA	0	0
rs546790067	C	NA	NA	0	0	NA	0	0	0	0	0	0	NA	0	NA	0	0.005051
rs553672299	A	NA	NA	0	0	NA	0	0	0	0	0	0	NA	0	NA	0	0
rs570629130	A	NA	NA	0	0	NA	0	0	0	0	0	0	NA	0	NA	0	0
rs117562633	C	NA	NA	0.009524	0	NA	0	0	0	0	0	0	NA	0	NA	0	0.005051
rs395584	C	0.6364	0.8824	0.3095	0.1915	0.4306	0.3333	0.04545	0.01099	0.2427	0.3097	0.01402	0.3654	0.299	0.5	0.3317	0.3586
rs143597099	A	NA	NA	NA	NA	NA	NA	NA	NA	NA	NA	NA	NA	NA	NA	NA	NA
rs543168691	C	NA	NA	0	0	NA	0.005051	0	0	0	0	0	NA	0	NA	0	0
rs530689404	T	NA	NA	0	0	NA	0	0	0	0	0	0	NA	0	NA	0	0
rs76833541	A	NA	NA	0.004762	0.01596	NA	0	0.1263	0.1044	0.02427	0	0.08411	NA	0.01471	NA	0	0
rs150907799	T	NA	NA	0	0	NA	0	0	0	0	0.05752	0	NA	0	NA	0	0
rs554550449	A	NA	NA	0	0	NA	0	0	0	0	0	0	NA	0	NA	0	0
rs566085188	A	NA	NA	0	0	NA	0.005051	0	0	0	0	0	NA	0	NA	0	0
rs553431242	G	NA	NA	0	0	NA	0	0	0	0	0	0	NA	0	NA	0	0
rs551442787	A	NA	NA	0	0	NA	0.005051	0	0	0	0	0	NA	0	NA	0	0
rs551677463	C	NA	NA	0.004762	0	NA	0	0	0	0	0	0	NA	0	NA	0	0
rs549801931	T	NA	NA	0	0	NA	0	0	0	0	0	0.004673	NA	0	NA	0	0
rs141864728	C	NA	NA	0	0	NA	0.01515	0	0	0	0	0	NA	0	NA	0	0
rs373480225	T	NA	NA	0	0	NA	0	0	0	0	0	0	NA	0	NA	0	0.0101
rs73903405	A	NA	NA	0	0	NA	0.09596	0	0	0	0.02655	0	NA	0	NA	0	0
rs567039032	A	NA	NA	0	0	NA	0	0	0	0	0	0	NA	0	NA	0	0
rs2070792	A	0.5	0	0.2048	0.3404	0.1111	0.4646	0.2172	0.3462	0.3155	0.5044	0.3411	0.03846	0.2696	0	0.2837	0.2121
rs552550910	A	NA	NA	0.004762	0	NA	0	0	0	0	0	0	NA	0	NA	0	0
rs535778149	A	NA	NA	0	0	NA	0	0	0	0	0	0	NA	0	NA	0	0
rs201984814	A	NA	NA	0	0	NA	0	0	0	0	0	0	NA	0	NA	0	0
rs544847277	A	NA	NA	0	0	NA	0	0	0	0	0	0	NA	0	NA	0	0
rs2070793	G	0.5	NA	0.2048	0.3404	NA	0.4646	0.2172	0.3462	0.3155	0.5044	0.3411	NA	0.2696	NA	0.2837	0.2121
rs191345780	A	NA	NA	0	0.005319	NA	0	0	0	0	0	0	NA	0	NA	0	0
rs9980693	A	NA	NA	0.07619	0.09043	NA	0.005051	0.2222	0.1209	0.09709	0.00885	0.1308	NA	0.09804	NA	0.03846	0.08081
rs146865760	T	NA	NA	0	0	NA	0	0.02525	0.02198	0.004854	0	0.004673	NA	0	NA	0	0
rs527898967	G	NA	NA	0	0	NA	0	0.005051	0	0	0	0	NA	0	NA	0	0
rs150389990	T	NA	NA	NA	NA	NA	NA	NA	NA	NA	NA	NA	NA	NA	NA	NA	NA
rs558492934	T	NA	NA	0	0	NA	0	0	0	0.004854	0	0	NA	0	NA	0	0
rs533513206	C	NA	NA	0	0	NA	0	0	0	0	0	0.004673	NA	0	NA	0	0

SNP	Allele	Chileans	Chopccas	CHS	CLM	Cusco	ESN	FIN	GBR	GIH	GWD	IBS	Iquitos	ITU	Jacarus	JPT	KHV
rs183758144	A	NA	NA	0	0	NA	0	0.005051	0.005495	0	0	0	NA	0	NA	0	0
rs377403765	A	NA	NA	0	0	NA	0	0	0	0	0	0	NA	0	NA	0	0
rs543740889	C	NA	NA	0	0	NA	0	0	0	0	0	0	NA	0	NA	0	0.005051
rs563188125	A	NA	NA	0	0	NA	0	0	0	0	0	0	NA	0	NA	0	0
rs536677167	G	NA	NA	0	0	NA	0	0	0	0	0.00885	0	NA	0	NA	0	0
rs541782164	T	NA	NA	0	0	NA	0	0	0	0.01456	0	0	NA	0.004902	NA	0	0
rs138161288	T	NA	NA	0	0	NA	0	0	0	0	0	0	NA	0	NA	0	0
rs541499151	A	NA	NA	0	0	NA	0	0	0	0	0	0	NA	0	NA	0	0
rs543339705	C	NA	NA	0	0	NA	0	0	0	0	0.00885	0	NA	0	NA	0	0
rs77599791	G	NA	NA	0	0	NA	0.005051	0	0	0	0.05752	0	NA	0	NA	0	0
rs549745562	G	NA	NA	0	0	NA	0	0	0	0	0	0	NA	0	NA	0	0
rs75317729	C	NA	NA	0	0	NA	0.005051	0	0	0	0	0	NA	0	NA	0	0
rs139661079	A	NA	NA	0	0	NA	0.005051	0	0	0	0.004425	0	NA	0	NA	0	0
rs572699613	A	NA	NA	0	0	NA	0	0	0	0	0	0	NA	0	NA	0	0
rs536023174	T	NA	NA	0	0	NA	0	0	0	0	0	0	NA	0.004902	NA	0	0
rs415731	A	0.2857	NA	0.3	0.2979	NA	0.2273	0.2929	0.3846	0.3107	0.2389	0.4159	NA	0.1961	NA	0.2981	0.3182
rs3761374	C	NA	0	0.07619	0.09043	0.02778	0	0.2222	0.1209	0.1068	0.004425	0.1308	0.08	0.1078	0	0.03846	0.08081
rs147233451	T	NA	NA	0	0	NA	0	0	0	0	0.004425	0	NA	0	NA	0	0
rs143595083	T	NA	NA	0	0	NA	0.0101	0	0	0	0.004425	0	NA	0	NA	0	0
rs565237319	C	NA	NA	0	0	NA	0	0	0	0	0	0	NA	0	NA	0	0
rs576510757	G	NA	NA	0	0	NA	0	0	0	0	0	0	NA	0	NA	0	0
rs535235411	A	NA	NA	0	0	NA	0	0	0	0	0	0	NA	0	NA	0	0
rs190265904	A	NA	NA	0	0	NA	0	0	0	0	0	0	NA	0	NA	0	0
rs149527323	A	NA	NA	0	0	NA	0	0	0	0	0.00885	0.004673	NA	0	NA	0	0
rs544141276	A	NA	NA	0	0	NA	0	0	0	0	0	0	NA	0	NA	0	0
rs186097675	T	NA	NA	0	0.0266	NA	0	0	0	0	0	0	NA	0	NA	0	0
rs547168890	A	NA	NA	0	0	NA	0	0	0	0	0	0	NA	0.009804	NA	0	0
rs138928680	GAGAA	NA	NA	0	0.005319	NA	0	0	0	0	0.0177	0.004673	NA	0	NA	0	0
rs567676569	T	NA	NA	0.004762	0	NA	0	0	0	0	0	0	NA	0	NA	0	0
rs530701233	A	NA	NA	0	0	NA	0	0	0	0	0	0	NA	0	NA	0	0
rs562332833	T	NA	NA	0	0	NA	0	0	0	0	0	0	NA	0	NA	0	0
rs531545827	T	NA	NA	0	0	NA	0	0	0	0	0.00885	0	NA	0	NA	0	0
rs530584676	T	NA	NA	0	0	NA	0.005051	0	0	0	0	0	NA	0	NA	0	0
rs185416185	T	NA	NA	0	0.01064	NA	0	0.0101	0	0	0	0.004673	NA	0	NA	0	0.0101
rs429442	T	0.6667	NA	0.2524	0.2553	NA	0.3788	0.1465	0.2418	0.2864	0.3142	0.2523	NA	0.25	NA	0.3173	0.2778
rs79065748	A	NA	NA	0	0	NA	0.03535	0	0	0	0.05752	0	NA	0	NA	0	0
rs575760704	C	NA	NA	0	0	NA	0	0	0	0.004854	0	0	NA	0	NA	0	0
rs875393	A	NA	NA	0.181	0.02128	NA	0	0.1313	0.05495	0.004854	0	0.03738	NA	0.01961	NA	0.226	0.1313
rs148901354	T	NA	NA	0	0	NA	0	0	0	0	0	0	NA	0	NA	0	0
rs561279116	G	NA	NA	0	0	NA	0	0	0	0	0.004425	0	NA	0	NA	0	0
rs138661151	A	NA	NA	0	0	NA	0.0101	0	0	0	0.05752	0	NA	0	NA	0	0
rs9975014	G	0.6667	NA	0.2095	0.2766	NA	0.2172	0.1768	0.2747	0.2913	0.3407	0.285	NA	0.2696	NA	0.3029	0.2222
rs573564326	A	NA	NA	0	0	NA	0.005051	0	0	0.004854	0.004425	0.004673	NA	0.004902	NA	0	0
rs144276163	G	NA	NA	0	0	NA	0	0	0	0	0	0	NA	0	NA	0	0
rs142750000	T	NA	NA	0	0	NA	0	0	0.005495	0.004854	0	0	NA	0	NA	0	0

SNP	Allele	Chileans	Chopccas	CHS	CLM	Cusco	ESN	FIN	GBR	GIH	GWD	IBS	Iquitos	ITU	Jacarus	JPT	KHV
rs143672898	T	NA	NA	0	0.005319	NA	0	0	0	0.004854	0	0.004673	NA	0	NA	0	0
rs560182574	C	NA	NA	0	0	NA	0	0	0	0	0	0	NA	0	NA	0	0
rs534312135	T	NA	NA	0	0	NA	0	0	0	0	0	0.004673	NA	0	NA	0	0
rs147986574	A	NA	NA	0	0	NA	0	0	0	0	0	0	NA	0	NA	0	0
rs181874957	T	NA	NA	0	0	NA	0	0	0	0	0	0	NA	0	NA	0	0
rs574957059	A	NA	NA	0	0	NA	0	0	0	0	0.004425	0	NA	0	NA	0	0
rs185018051	C	NA	NA	0	0	NA	0	0	0	0	0	0	NA	0	NA	0	0
rs564806692	A	NA	NA	0	0	NA	0	0	0	0.004854	0	0	NA	0	NA	0	0
rs556079659	C	NA	NA	0	0	NA	0	0	0.005495	0	0	0	NA	0	NA	0	0
rs79617378	C	NA	0	0	0.01064	0	0	0.01515	0.02747	0.004854	0	0.009346	0	0	0	0	0
rs189436980	G	NA	NA	0	0	NA	0.005051	0	0	0	0	0	NA	0	NA	0	0
rs187078345	A	NA	NA	0	0	NA	0.0101	0	0	0	0.004425	0	NA	0	NA	0	0
rs534885992	A	NA	NA	0	0	NA	0	0	0	0	0	0	NA	0	NA	0	0
rs187143520	C	NA	NA	0	0	NA	0	0	0	0	0.004425	0	NA	0	NA	0	0
rs535174935	T	NA	NA	0	0.005319	NA	0	0	0	0	0	0	NA	0	NA	0	0
rs528681261	T	NA	NA	0	0	NA	0	0.005051	0	0	0	0	NA	0	NA	0	0
rs563352095	T	NA	NA	0	0	NA	0	0	0	0	0	0	NA	0	NA	0	0
rs117688613	G	NA	NA	0	0	NA	0	0	0	0	0	0	NA	0	NA	0	0
rs550253934	G	NA	NA	0	0	NA	0	0	0	0	0	0	NA	0.004902	NA	0	0
rs181162134	C	NA	NA	0	0	NA	0	0	0	0	0	0	NA	0	NA	0	0
rs192026800	A	NA	NA	0.009524	0	NA	0	0	0	0	0	0	NA	0	NA	0	0.0101
rs552303675	T	NA	NA	0	0	NA	0	0	0	0	0	0	NA	0	NA	0	0
rs147147950	A	NA	NA	0	0	NA	0	0	0	0	0	0	NA	0	NA	0.009615	0
rs76314085	G	NA	NA	0.009524	0	NA	0	0	0	0	0	0	NA	0	NA	0.004808	0
rs567504414	T	NA	NA	0.009524	0	NA	0	0	0	0	0	0	NA	0	NA	0	0.005051
rs563853944	C	NA	NA	0	0	NA	0	0.005051	0.005495	0	0	0	NA	0	NA	0	0
rs578103765	T	NA	NA	0	0	NA	0	0	0	0	0	0	NA	0	NA	0	0
rs574238899	G	NA	NA	0	0.005319	NA	0	0	0.005495	0	0	0	NA	0	NA	0	0
rs2838040	G	0.5	NA	0.2952	0.2553	NA	0.4545	0.4091	0.2308	0.2136	0.5841	0.2383	NA	0.2647	NA	0.3077	0.2374
rs537876986	A	NA	NA	0	0	NA	0	0	0	0	0	0	NA	0	NA	0	0
rs550222907	T	NA	NA	0	0	NA	0	0	0	0	0	0	NA	0	NA	0	0
rs556260609	A	NA	NA	0	0	NA	0	0	0	0	0	0	NA	0	NA	0	0
rs142296178	A	NA	NA	0	0	NA	0	0	0	0	0	0	NA	0	NA	0	0
rs577285910	A	NA	NA	0	0	NA	0	0	0	0	0	0	NA	0	NA	0	0.005051
rs566925007	A	NA	NA	0	0	NA	0	0	0	0	0	0	NA	0	NA	0	0
rs457909	G	0	NA	0.02857	0	NA	0	0	0	0	0	0	NA	0	NA	0.01923	0.05051
rs562479511	T	NA	NA	0	0	NA	0	0	0	0	0	0	NA	0.004902	NA	0	0
rs2298663	T	0.5	NA	0.2048	0.6011	NA	0.3838	0.6162	0.7802	0.7573	0.4823	0.8037	NA	0.6814	NA	0.2885	0.2273
rs2070787	G	0.3333	NA	0.2095	0.3351	NA	0.2727	0.2172	0.3516	0.3155	0.385	0.3318	NA	0.2696	NA	0.2837	0.2121
rs573615979	C	NA	NA	0	0	NA	0	0	0	0	0	0	NA	0	NA	0	0
rs542031508	C	NA	NA	0	0	NA	0	0	0	0	0	0	NA	0	NA	0	0
rs555600211	A	NA	NA	0	0	NA	0	0	0.005495	0	0	0	NA	0	NA	0	0
rs528451616	A	NA	NA	0	0	NA	0	0	0	0.01456	0	0	NA	0.004902	NA	0	0
rs378501	G	NA	NA	0.08571	0	NA	0	0	0	0	0	0	NA	0	NA	0.009615	0.1212
rs140408843	A	NA	NA	0	0	NA	0	0	0	0	0	0	NA	0	NA	0.01442	0

SNP	Allele	Chileans	Chopccas	CHS	CLM	Cusco	ESN	FIN	GBR	GIH	GWD	IBS	Iquitos	ITU	Jacarus	JPT	KHV
rs186280517	C	NA	NA	0.004762	0	NA	0	0	0	0	0	0	NA	0	NA	0	0
rs148561341	T	NA	NA	0	0	NA	0	0	0	0	0	0	NA	0	NA	0.004808	0.005051
rs568162250	A	NA	NA	0	0	NA	0	0	0	0	0	0	NA	0	NA	0	0
rs561709263	C	NA	NA	0	0	NA	0	0	0.005495	0	0	0	NA	0	NA	0	0
rs569583276	A	NA	NA	0.004762	0	NA	0	0	0	0	0	0	NA	0.004902	NA	0	0
rs572164476	A	NA	NA	0	0	NA	0	0	0	0	0	0	NA	0	NA	0	0
rs560575827	T	NA	NA	0.004762	0	NA	0	0	0	0	0	0	NA	0	NA	0	0
rs111220492	G	NA	NA	0	0.3457	NA	0.1919	0.4293	0.5055	0.4612	0.2434	0.5234	NA	0.4265	NA	0.004808	0.01515
rs182231135	C	NA	NA	0	0.005319	NA	0	0	0	0	0	0	NA	0	NA	0	0
rs531884512	T	NA	NA	0	0	NA	0	0	0	0	0	0	NA	0	NA	0	0
rs573986970	A	NA	NA	0	0	NA	0	0	0	0	0	0	NA	0	NA	0	0
rs564705715	A	NA	NA	0	0	NA	0.005051	0	0	0	0	0	NA	0	NA	0	0
rs201661208	A	NA	NA	0	0	NA	0	0	0	0	0	0	NA	0	NA	0	0
rs530540353	C	NA	NA	0	0	NA	0	0	0	0	0	0	NA	0	NA	0	0
rs148155433	A	NA	NA	0	0	NA	0	0	0	0	0	0	NA	0	NA	0	0
rs558834486	G	NA	NA	0	0	NA	0.005051	0	0	0	0	0	NA	0	NA	0	0
rs186275240	A	NA	NA	0	0.01596	NA	0	0	0	0	0	0	NA	0	NA	0	0
rs531418415	A	NA	NA	0	0	NA	0	0	0.005495	0	0	0	NA	0	NA	0	0
rs531609155	G	NA	NA	0	0	NA	0	0	0	0	0	0	NA	0	NA	0	0
rs376752614	A	NA	NA	0	0	NA	0	0	0	0	0	0	NA	0.004902	NA	0	0
rs139144487	A	NA	NA	0	0	NA	0	0	0	0	0	0	NA	0	NA	0	0
rs531754656	A	NA	NA	0	0	NA	0	0	0	0	0	0	NA	0	NA	0	0
rs554702767	A	NA	NA	0	0	NA	0	0	0	0	0	0	NA	0	NA	0	0
rs456016	T	0.4	NA	0.3762	0.1649	NA	0.0101	0.0404	0.01099	0.06796	0.02655	0.004673	NA	0.09314	NA	0.2933	0.4141
rs144364265	T	NA	NA	0.004762	0	NA	0	0	0	0	0	0	NA	0	NA	0	0
rs113773731	C	NA	NA	0	0	NA	0.02525	0	0	0	0.00885	0	NA	0	NA	0	0
rs142261174	T	NA	NA	0	0	NA	0.005051	0	0	0	0	0	NA	0	NA	0	0
rs563595464	T	NA	NA	0	0	NA	0.005051	0	0	0	0	0	NA	0	NA	0	0
rs531013422	T	NA	NA	0	0	NA	0	0	0	0	0.004425	0	NA	0	NA	0	0
rs569886879	G	NA	NA	0	0	NA	0	0	0	0	0.00885	0	NA	0	NA	0	0
rs541977145	G	NA	NA	0	0	NA	0	0	0	0	0	0	NA	0	NA	0	0
rs190906841	T	NA	NA	0	0	NA	0	0	0	0	0	0	NA	0	NA	0	0
rs139222305	T	NA	NA	0	0.01596	NA	0	0.01515	0.02198	0.01456	0	0.01869	NA	0	NA	0	0
rs527242422	T	NA	NA	0	0	NA	0	0	0	0.009709	0	0	NA	0	NA	0	0
rs364289	A	0.5	NA	0.2524	0.2553	NA	0.4646	0.1414	0.2198	0.2864	0.4823	0.257	NA	0.2598	NA	0.3125	0.2727
rs371053759	T	NA	NA	0	0	NA	0.0101	0	0	0	0.004425	0	NA	0	NA	0	0
rs146116431	T	NA	NA	0	0	NA	0	0	0	0	0	0	NA	0	NA	0.004808	0
rs928871	C	0.5	NA	0.6905	0.7394	NA	0.3182	0.5859	0.7692	0.7864	0.3274	0.757	NA	0.7451	NA	0.6635	0.7475
rs532335955	T	NA	NA	0.004762	0	NA	0	0	0	0	0	0	NA	0	NA	0	0.005051
rs113928389	A	NA	NA	0	0	NA	0.0101	0	0	0	0.004425	0	NA	0	NA	0	0
rs147138431	A	NA	NA	0	0.01064	NA	0	0.01515	0	0	0.0177	0.004673	NA	0	NA	0	0
rs4283504	T	0.1667	NA	0.2381	0.117	NA	0.02525	0.1364	0.1264	0.1359	0.0531	0.1308	NA	0.1127	NA	0.2788	0.2929
rs145297649	G	NA	NA	0	0	NA	0.005051	0	0	0	0	0	NA	0	NA	0	0
rs146957681	T	NA	NA	0	0	NA	0	0	0	0	0	0	NA	0	NA	0	0
rs562177468	G	NA	NA	0	0	NA	0	0	0	0	0	0	NA	0.004902	NA	0	0

SNP	Allele	Chileans	Chopccas	CHS	CLM	Cusco	ESN	FIN	GBR	GIH	GWD	IBS	Iquitos	ITU	Jacarus	JPT	KHV
rs576030223	A	NA	NA	0	0	NA	0	0	0	0	0	0	NA	0	NA	0	0
rs190385097	A	NA	NA	0	0	NA	0	0	0	0	0.004425	0	NA	0	NA	0	0
rs7283324	T	0.5	NA	0.419	0.234	NA	0.4242	0.3333	0.1978	0.2039	0.4027	0.1682	NA	0.2255	NA	0.4183	0.3586
rs118108663	T	NA	NA	0.01905	0	NA	0	0	0	0	0	0	NA	0	NA	0.02885	0.0101
rs61735789	A	NA	NA	0	0.005319	NA	0	0.005051	0.005495	0	0	0.02336	NA	0	NA	0	0
rs113564116	C	NA	NA	0	0.005319	NA	0	0.005051	0.01099	0	0	0.01402	NA	0	NA	0	0
rs2298659	A	0.5	NA	0.2619	0.1862	NA	0.1717	0.3384	0.2143	0.1602	0.1637	0.1636	NA	0.1765	NA	0.3221	0.1818
rs150581606	A	NA	NA	0	0	NA	0	0	0	0	0	0	NA	0	NA	0.004808	0
rs116170128	C	NA	NA	0	0.04255	NA	0.1263	0.005051	0.02747	0.02427	0.1372	0.03271	NA	0.004902	NA	0	0
rs143291395	A	NA	NA	0.004762	0	NA	0	0	0	0	0	0	NA	0	NA	0	0
rs112753686	T	NA	NA	0	0	NA	0.0202	0	0	0	0.0177	0	NA	0	NA	0	0
rs75603675	A	0.5	NA	0.01429	0.3138	NA	0.303	0.404	0.4066	0.2087	0.2434	0.3785	NA	0.1765	NA	0.01442	0.0101
rs553516093	A	NA	NA	0	0	NA	0	0	0	0.004854	0	0	NA	0	NA	0	0
rs143460343	A	NA	NA	0	0	NA	0.005051	0	0	0	0.00885	0	NA	0	NA	0	0
rs184365262	T	NA	NA	0	0	NA	0	0.0101	0	0	0	0	NA	0	NA	0	0
rs571074719	T	NA	NA	0	0	NA	0.005051	0	0	0	0	0	NA	0	NA	0	0
rs115975538	A	NA	NA	0	0	NA	0.06061	0	0	0	0	0	NA	0.009804	NA	0	0
rs147465180	A	NA	NA	0	0	NA	0	0	0	0	0	0	NA	0	NA	0	0
rs548349124	G	NA	NA	0	0	NA	0	0	0	0	0	0	NA	0	NA	0	0
rs139467735	T	NA	NA	0	0.005319	NA	0.07071	0	0	0	0.1504	0.009346	NA	0.009804	NA	0	0
rs576042280	T	NA	NA	0	0	NA	0	0	0	0	0	0	NA	0.004902	NA	0	0
rs544083065	A	NA	NA	0	0	NA	0	0	0	0	0	0	NA	0	NA	0	0
rs551244742	A	NA	NA	0	0	NA	0	0	0	0	0	0.004673	NA	0	NA	0	0
rs149076631	T	0.5	NA	0	0	NA	0.0101	0	0	0	0.00885	0	NA	0	NA	0	0
rs35041537	T	0.5	0.08824	0	0.3138	0.125	0.101	0.3939	0.5165	0.4369	0.04867	0.486	0.25	0.4069	0.02941	0.004808	0.01515
rs56218846	A	0.5	NA	0.01905	0.3298	NA	0.3232	0.3838	0.4011	0.1845	0.2566	0.3832	NA	0.1618	NA	0.01923	0.01515
rs527693716	T	NA	NA	0	0	NA	0	0	0	0	0	0	NA	0	NA	0	0
rs189298425	A	NA	NA	0	0	NA	0	0	0	0	0	0	NA	0	NA	0.004808	0
rs562750287	A	NA	NA	0	0	NA	0	0	0	0	0	0	NA	0	NA	0	0
rs570413089	A	NA	NA	0.004762	0	NA	0	0	0	0	0	0	NA	0	NA	0	0
rs111383922	T	NA	NA	0	0	NA	0	0	0	0	0.004425	0	NA	0	NA	0	0
rs559699558	A	NA	NA	0	0	NA	0	0	0	0	0	0	NA	0	NA	0.004808	0
rs542060428	T	NA	NA	0	0	NA	0	0	0	0	0	0	NA	0	NA	0	0
rs565142477	T	NA	NA	0	0	NA	0	0	0	0	0	0	NA	0	NA	0	0
rs548742822	C	NA	NA	0	0	NA	0	0	0	0	0	0	NA	0	NA	0	0
rs148304071	T	NA	NA	0.004762	0	NA	0	0	0	0	0	0	NA	0	NA	0.004808	0
rs455045	C	0.2857	NA	0.3762	0.3883	NA	0.2525	0.5152	0.5055	0.4175	0.2522	0.5467	NA	0.3039	NA	0.3413	0.399
rs570441750	G	NA	NA	0	0	NA	0	0	0	0.004854	0	0	NA	0	NA	0	0
rs9974589	A	0.4	NA	0.3762	0.4309	NA	0.3182	0.4495	0.4451	0.4806	0.2212	0.5	NA	0.5098	NA	0.2981	0.4293
rs2104810	A	0.5	NA	0.2048	0.6011	NA	0.4192	0.601	0.7802	0.7573	0.5487	0.8084	NA	0.6912	NA	0.2885	0.2222
rs147711290	C	NA	NA	NA	NA	NA	NA	NA	NA	NA	NA	NA	NA	NA	NA	NA	NA
rs555056677	A	NA	NA	0	0	NA	0	0	0	0	0	0	NA	0	NA	0	0
rs9974933	G	0.6667	NA	0.2095	0.2766	NA	0.2172	0.1768	0.2747	0.2864	0.3407	0.285	NA	0.2696	NA	0.3029	0.2222
rs544221548	A	NA	NA	0	0	NA	0	0	0	0	0	0	NA	0	NA	0	0
rs147827602	T	NA	NA	0	0	NA	0	0	0	0	0	0	NA	0	NA	0	0

SNP	Allele	Chileans	Chopccas	CHS	CLM	Cusco	ESN	FIN	GBR	GIH	GWD	IBS	Iquitos	ITU	Jacarus	JPT	KHV
rs462321	T	0.5	NA	0.7905	0.3777	NA	0.4141	0.3687	0.1868	0.2427	0.4115	0.1495	NA	0.3137	NA	0.7115	0.7576
rs142659685	T	NA	NA	0.07143	0	NA	0.005051	0	0	0	0	0	NA	0	NA	0.04808	0.06061
rs150838246	C	NA	NA	0	0	NA	0	0	0	0	0	0	NA	0	NA	0	0
rs112132031	C	0.4	NA	0.3762	0.1702	NA	0.2121	0.0404	0.01099	0.06796	0.1504	0.01402	NA	0.09314	NA	0.2933	0.4141
rs9974995	T	0.6667	0.1765	0.2095	0.2766	0.2917	0.2172	0.1768	0.2747	0.2864	0.3407	0.285	0.1731	0.2696	0.2647	0.3029	0.2222
rs557494549	TCAGGGAGTGCAGAGCAGGAGGGAC	NA	NA	0	0	NA	0.005051	0	0	0	0.00885	0	NA	0	NA	0	0
rs186929947	G	NA	NA	0	0	NA	0	0	0	0	0	0	NA	0	NA	0	0
rs148509204	T	NA	NA	0	0	NA	0.005051	0	0	0	0.01327	0	NA	0	NA	0	0
rs202094412	A	NA	NA	0.004762	0	NA	0	0	0	0	0	0	NA	0	NA	0.004808	0
rs575981127	A	NA	NA	0	0	NA	0	0	0	0	0	0	NA	0	NA	0	0
rs546775580	T	NA	NA	0	0	NA	0	0	0	0	0	0	NA	0	NA	0.004808	0
rs185188053	T	NA	NA	0	0.01064	NA	0	0	0	0	0	0	NA	0	NA	0	0
rs577381045	A	NA	NA	0.004762	0	NA	0	0	0	0	0	0	NA	0	NA	0	0
rs62217525	T	NA	NA	0	0.03723	NA	0	0.03535	0.06044	0.01942	0	0.07009	NA	0.01961	NA	0	0
rs561043908	A	NA	NA	0	0	NA	0	0	0	0	0	0	NA	0	NA	0	0
rs367879274	T	NA	NA	0	0	NA	0	0	0	0	0	0	NA	0	NA	0	0
rs568167564	G	NA	NA	0	0	NA	0	0	0	0	0	0	NA	0	NA	0	0
rs563633617	A	NA	NA	0	0	NA	0	0	0	0	0.004425	0	NA	0	NA	0	0
rs537935645	T	NA	NA	0.004762	0	NA	0	0	0	0	0	0	NA	0	NA	0	0
rs9985159	C	0.5	0.7647	0.5762	0.766	0.7222	0.4293	0.6616	0.7912	0.8252	0.5796	0.8178	0.6538	0.7892	0.8235	0.5769	0.6364
rs61735795	A	NA	NA	NA	NA	NA	NA	NA	NA	NA	NA	NA	NA	NA	NA	NA	NA
rs557712450	C	NA	NA	0	0	NA	0	0.0101	0	0	0	0	NA	0	NA	0	0
rs139305247	T	0.5	NA	0	0.03723	NA	0	0.07576	0.01648	0.004854	0	0.01402	NA	0	NA	0	0
rs548653178	A	NA	NA	0	0	NA	0	0	0	0	0	0	NA	0	NA	0	0
rs146385718	C	NA	NA	0	0	NA	0.005051	0	0	0	0.02655	0	NA	0	NA	0	0
rs28360562	C	0.5	NA	0	0.08511	NA	0.03535	0.09596	0.1044	0.1068	0.06195	0.09813	NA	0.07353	NA	0	0
rs9983330	G	0.5	NA	0.4048	0.2021	NA	0.2626	0.3586	0.2033	0.1408	0.2522	0.1776	NA	0.1863	NA	0.399	0.3586
rs539864066	G	NA	NA	0	0	NA	0.01515	0	0	0	0	0	NA	0.004902	NA	0	0
rs562014334	A	NA	NA	0	0	NA	0	0	0	0	0	0	NA	0	NA	0	0
rs118134524	A	NA	NA	0.004762	0	NA	0	0	0	0	0	0	NA	0	NA	0.004808	0.01515
rs574811036	A	NA	NA	0	0	NA	0	0	0	0	0.004425	0	NA	0	NA	0	0
rs115720411	A	NA	NA	0	0.005319	NA	0.2828	0	0	0	0.177	0.009346	NA	0	NA	0	0
rs368812287	T	NA	NA	0	0	NA	0.005051	0	0	0	0	0	NA	0	NA	0	0
rs2410428	T	0.6667	NA	0.2095	0.2606	NA	0.08081	0.1768	0.2692	0.2913	0.1504	0.2804	NA	0.2647	NA	0.3029	0.2222
rs577480005	A	NA	NA	0	0	NA	0.005051	0	0	0	0	0	NA	0	NA	0	0
rs543815381	T	NA	NA	0	0	NA	0	0	0	0	0	0	NA	0	NA	0	0
rs142834250	G	NA	NA	0	0	NA	0	0	0	0	0	0	NA	0	NA	0.02885	0
rs576292358	A	NA	NA	0	0	NA	0	0	0	0	0	0	NA	0	NA	0	0
rs141603473	A	NA	NA	0	0	NA	0.02525	0	0	0	0.03982	0	NA	0	NA	0	0
rs143523726	G	NA	NA	0	0	NA	0	0	0	0	0	0	NA	0	NA	0	0
rs375408	G	0.4	NA	0.3762	0.1649	NA	0.0101	0.04545	0.01099	0.06311	0.01327	0.004673	NA	0.08333	NA	0.2981	0.399
rs370347248	C	NA	NA	0	0	NA	0	0	0	0	0	0	NA	0	NA	0	0
rs539123545	C	NA	NA	0	0	NA	0	0	0	0	0	0	NA	0	NA	0	0
rs4303794	C	0.5	NA	0.01905	0.3138	NA	0.303	0.404	0.4176	0.2087	0.2389	0.3832	NA	0.1765	NA	0.01442	0.0101
rs151035593	T	NA	NA	0	0	NA	0.02525	0	0	0	0.004425	0	NA	0	NA	0	0

SNP	Allele	Chileans	Chopccas	CHS	CLM	Cusco	ESN	FIN	GBR	GIH	GWD	IBS	Iquitos	ITU	Jacarus	JPT	KHV
rs370896169	C	NA	NA	0	0	NA	0	0	0	0	0	0	NA	0	NA	0	0
rs575167859	T	NA	NA	0	0	NA	0	0	0	0.004854	0	0	NA	0	NA	0	0
rs35050484	A	0.5	NA	0	0.0266	NA	0	0.0404	0.06044	0.01456	0	0.03738	NA	0	NA	0	0
rs56136037	T	NA	NA	0	0	NA	0	0.05556	0.05495	0.01942	0	0.03738	NA	0	NA	0	0
rs539767738	A	NA	NA	0	0	NA	0	0	0	0	0	0	NA	0	NA	0	0
rs533229030	G	NA	NA	0	0	NA	0	0	0	0	0	0	NA	0	NA	0.004808	0
rs465576	C	0.4	NA	0.3762	0.1702	NA	0.202	0.0404	0.01099	0.06796	0.146	0.01402	NA	0.09314	NA	0.2933	0.4141
rs570494222	T	NA	NA	0	0	NA	0	0	0	0	0.004425	0	NA	0	NA	0	0
rs4818240	T	0.4	NA	0.4143	0.1702	NA	0.2576	0.04545	0.01099	0.06311	0.2168	0.009346	NA	0.07843	NA	0.2981	0.4444
rs9636988	C	0.6667	0.1765	0.2095	0.2766	0.2917	0.2172	0.1818	0.2747	0.2816	0.3407	0.285	0.1731	0.2647	0.2647	0.3029	0.2172
rs541887371	T	NA	NA	0	0	NA	0	0	0	0	0	0	NA	0	NA	0	0.005051
rs537787922	A	NA	NA	0	0	NA	0	0	0	0	0	0	NA	0	NA	0	0
rs139829932	T	NA	NA	0	0	NA	0.005051	0	0	0	0.0177	0	NA	0	NA	0	0
rs200615061	C	NA	NA	0	0	NA	0	0	0	0	0	0.004673	NA	0.004902	NA	0	0
rs3787947	T	0.5	NA	0.3048	0.2553	NA	0.4091	0.4192	0.2308	0.2136	0.3938	0.2383	NA	0.25	NA	0.3365	0.2475
rs402197	T	0.4	NA	0.3762	0.1649	NA	0.01515	0.04545	0.01099	0.06311	0.02212	0.004673	NA	0.08333	NA	0.2981	0.399
rs562203987	T	NA	NA	0.004762	0	NA	0	0	0	0	0	0	NA	0	NA	0	0
rs141764184	A	NA	NA	0.02381	0	NA	0	0	0	0	0	0	NA	0	NA	0.009615	0.0101
rs116577479	A	NA	NA	0	0.01596	NA	0.05051	0	0	0	0.06637	0	NA	0	NA	0	0
rs118133613	A	NA	NA	0	0	NA	0	0.005051	0	0	0	0.004673	NA	0	NA	0	0
rs118108194	G	NA	NA	0	0	NA	0	0.005051	0.02198	0.004854	0	0.004673	NA	0.004902	NA	0	0
rs138763189	A	NA	NA	0	0	NA	0.005051	0	0	0	0.004425	0	NA	0	NA	0	0
rs138365638	TGG	NA	NA	0.381	0.1649	NA	0.02525	0.04545	0.01099	0.06311	0.02212	0.004673	NA	0.08824	NA	0.2933	0.404
rs551442751	T	NA	NA	0	0	NA	0	0	0	0	0	0	NA	0	NA	0	0
rs539019775	T	NA	NA	0	0	NA	0	0	0	0	0.004425	0	NA	0	NA	0	0
rs112657409	T	NA	0	0.1	0.005319	0	0.1212	0	0	0	0.08407	0	0	0	0	0.06731	0.101
rs577689706	A	NA	NA	0.004762	0	NA	0	0	0	0	0	0	NA	0	NA	0	0
rs147349930	T	NA	NA	0	0	NA	0.0101	0	0	0	0.004425	0	NA	0	NA	0	0
rs557531433	T	NA	NA	0	0	NA	0	0	0	0	0	0.004673	NA	0	NA	0	0
rs144359794	T	NA	NA	0	0	NA	0	0	0	0	0	0	NA	0	NA	0.004808	0
rs187426170	T	NA	NA	0	0	NA	0	0	0.01099	0	0	0.004673	NA	0.004902	NA	0	0
rs199575615	G	NA	NA	0	0	NA	0	0	0	0	0	0	NA	0	NA	0	0
rs529737187	A	NA	NA	0	0	NA	0	0	0	0	0	0	NA	0	NA	0	0
rs576447019	A	NA	NA	0.004762	0	NA	0	0	0	0	0	0	NA	0	NA	0	0
rs145900878	T	NA	NA	0	0	NA	0.05556	0	0	0	0.03982	0	NA	0	NA	0	0
rs9977234	T	0.6667	NA	0.2095	0.2447	NA	0.07071	0.1465	0.2527	0.2816	0.09735	0.257	NA	0.25	NA	0.3029	0.2273
rs540024706	C	NA	NA	0	0	NA	0	0	0	0.009709	0	0	NA	0.009804	NA	0	0
rs528640390	G	NA	NA	0	0.005319	NA	0	0	0	0	0.02655	0	NA	0	NA	0	0
rs546337899	G	NA	NA	0.004762	0.005319	NA	0	0	0.01099	0.004854	0	0.004673	NA	0	NA	0.01923	0.01515
rs141323355	A	NA	NA	0	0	NA	0	0	0	0	0	0	NA	0	NA	0	0
rs386519	G	NA	NA	0	0	NA	0.005051	0	0.005495	0	0	0	NA	0	NA	0	0
rs561327241	T	NA	NA	0	0	NA	0	0	0	0.01456	0	0	NA	0.004902	NA	0	0
rs572351113	A	NA	NA	0	0	NA	0	0	0	0	0	0	NA	0	NA	0	0
rs189425119	A	NA	NA	0	0	NA	0	0	0	0	0	0	NA	0	NA	0	0
rs566485026	G	NA	NA	0	0	NA	0	0	0	0	0	0	NA	0.004902	NA	0	0

SNP	Allele	Chileans	Chopccas	CHS	CLM	Cusco	ESN	FIN	GBR	GIH	GWD	IBS	Iquitos	ITU	Jacarus	JPT	KHV
rs2298665	C	NA	NA	0.004762	0	NA	0	0	0	0	0	0	NA	0	NA	0	0.005051
rs142769034	T	NA	NA	0.004762	0.01596	NA	0	0.01515	0.02198	0.01456	0	0.01869	NA	0	NA	0	0
rs74564819	C	NA	NA	0	0	NA	0	0	0	0	0	0	NA	0	NA	0.009615	0
rs2298857	A	0.6667	0.1765	0.219	0.2606	0.2917	0.4141	0.1465	0.2473	0.2864	0.4779	0.271	0.1731	0.2598	0.2647	0.3029	0.2323
rs545232590	T	NA	NA	0	0	NA	0	0	0	0	0	0	NA	0	NA	0	0.005051
rs529218967	G	NA	NA	0	0	NA	0	0	0	0	0	0	NA	0	NA	0	0
rs528337775	T	NA	NA	0	0	NA	0	0	0	0.004854	0	0	NA	0.004902	NA	0	0
rs563980567	A	NA	NA	0	0	NA	0.005051	0	0	0	0	0	NA	0	NA	0	0
rs534913861	T	NA	NA	0	0	NA	0	0	0	0	0.004425	0	NA	0	NA	0	0
rs535348902	A	NA	NA	0	0	NA	0	0	0	0	0	0	NA	0.004902	NA	0	0
rs555405939	C	NA	NA	0	0	NA	0	0	0	0	0	0	NA	0	NA	0	0.005051
rs187037274	A	NA	NA	0	0	NA	0.0101	0	0	0	0.004425	0	NA	0	NA	0	0
rs141027872	T	NA	NA	0	0	NA	0	0	0	0	0	0	NA	0	NA	0	0
rs191394761	T	NA	NA	0	0.03191	NA	0	0	0	0	0	0.01402	NA	0	NA	0	0
rs540964406	G	NA	NA	0	0	NA	0	0	0.005495	0	0	0	NA	0	NA	0	0
rs544440280	A	NA	NA	0	0	NA	0	0	0	0	0	0	NA	0	NA	0	0.0101
rs146845793	C	NA	NA	0.004762	0	NA	0	0	0	0	0	0	NA	0	NA	0	0
rs372665499	T	NA	NA	0	0	NA	0.005051	0	0	0	0	0	NA	0	NA	0	0
rs538276300	A	NA	NA	0	0	NA	0	0	0	0	0.004425	0	NA	0	NA	0	0
rs113562865	T	NA	NA	0	0.005319	NA	0.1869	0	0	0	0.1195	0.009346	NA	0	NA	0	0
rs571417292	A	NA	NA	0	0	NA	0	0	0	0	0.004425	0	NA	0	NA	0	0
rs542865477	A	NA	NA	0	0	NA	0	0	0	0	0	0	NA	0	NA	0	0
rs544110139	G	NA	NA	0	0	NA	0	0	0	0	0	0	NA	0	NA	0	0
rs142914234	C	NA	NA	0	0	NA	0.005051	0	0	0	0.00885	0	NA	0	NA	0	0
rs114549926	A	NA	NA	0	0	NA	0.0101	0	0	0	0.00885	0	NA	0	NA	0	0
rs549289994	C	NA	NA	0	0	NA	0	0	0	0	0.00885	0	NA	0	NA	0	0
rs555327583	A	NA	NA	0	0	NA	0	0	0	0.004854	0	0	NA	0	NA	0	0
rs111220533	G	NA	NA	0.2048	0.4202	NA	0.2626	0.3889	0.544	0.4903	0.3584	0.5187	NA	0.4559	NA	0.2837	0.2172
rs553400003	A	NA	NA	0	0	NA	0	0	0	0.01456	0	0	NA	0	NA	0	0
rs530306757	A	NA	NA	0	0	NA	0	0	0	0	0	0	NA	0	NA	0	0
rs561412657	G	NA	NA	0.004762	0	NA	0	0	0	0	0	0	NA	0.009804	NA	0	0
rs565690211	G	NA	NA	0	0	NA	0	0	0	0	0	0	NA	0	NA	0	0
rs137929897	T	NA	NA	0	0	NA	0.005051	0	0	0	0	0	NA	0	NA	0	0
rs144458055	A	NA	NA	0.004762	0	NA	0	0	0.01099	0.004854	0.009174	0.004673	NA	0.009804	NA	0.009615	0.0303
rs141722242	A	NA	NA	0	0	NA	0	0	0	0	0	0	NA	0	NA	0	0
rs565720190	A	NA	NA	0	0	NA	0	0	0	0	0	0	NA	0	NA	0	0
rs530051850	C	NA	NA	0	0	NA	0	0	0	0	0	0.004673	NA	0	NA	0	0
rs115967323	T	NA	NA	0	0	NA	0	0	0	0	0	0	NA	0	NA	0	0

Table S4-A – TMPRSS2 allele frequencies continuation. Bold = functionally relevant SNPs found in our databases; NA = missing data.

SNP	Allele	Lamas	Lambayeque	Lima	LWK	Matses	Moche	Moquegua	MSL	MXL	PEL	Pelotas	PJL	Puno	PUR	Qeros	Quechuas
**rs456298**	**T**	**NA**	**NA**	**NA**	**0.32**	**NA**	**NA**	**NA**	**0.43**	**0.38**	**0.51**	**0.20**	**0.30**	**NA**	**0.25**	**NA**	**NA**
**rs12329760**	**T**	**0.04762**	**0.04167**	**0.01786**	**0.2879**	**0.04545**	**0.01667**	**0.01724**	**0.3118**	**0.1797**	**0.06471**	**0.206**	**0.2396**	**0**	**0.1683**	**0**	**0.04167**

SNP	Allele	Lamas	Lambayeque	Lima	LWK	Matses	Moche	Moquegua	MSL	MXL	PEL	Pelotas	PJL	Puno	PUR	Qeros	Quechuas
**rs2276205**	**G**	**0.02381**	**0**	**0.01786**	**0.197**	**0**	**0.01667**	**0.01724**	**0.07647**	**0.0625**	**0.01765**	**0.09104**	**0.05729**	**0**	**0.05288**	**0**	**0**
**rs383510**	**T**	**NA**	**NA**	**NA**	**0.2424**	**NA**	**NA**	**NA**	**0.3118**	**0.4375**	**0.3529**	**NA**	**0.375**	**NA**	**0.3894**	**NA**	**NA**
**rs2070788**	**G**	**0.5476**	**0.75**	**0.6071**	**0.1768**	**0.7727**	**0.7667**	**0.7069**	**0.2941**	**0.4688**	**0.6412**	**0.428**	**0.4531**	**0.8125**	**0.4471**	**0.91667**	**0.6875**
rs149270377	A	NA	NA	NA	0.0303	NA	NA	NA	0	0	0	NA	0	NA	0	NA	NA
rs75833467	A	NA	NA	NA	0	NA	NA	NA	0	0.0625	0.01765	NA	0.01562	NA	0.08173	NA	NA
rs569429288	A	NA	NA	NA	0	NA	NA	NA	0	0	0	NA	0.01562	NA	0	NA	NA
rs554852594	C	NA	NA	NA	0	NA	NA	NA	0	0	0	NA	0	NA	0	NA	NA
rs537997349	G	NA	NA	NA	0.01515	NA	NA	NA	0.01176	0	0.005882	NA	0	NA	0.004808	NA	NA
rs569432639	C	NA	NA	NA	0	NA	NA	NA	0	0	0	NA	0	NA	0	NA	NA
rs535019926	C	NA	NA	NA	0	NA	NA	NA	0	0	0	NA	0	NA	0	NA	NA
rs368646465	T	NA	NA	NA	0	NA	NA	NA	0	0	0	NA	0.02083	NA	0	NA	NA
rs9305744	A	NA	NA	NA	0.3737	NA	NA	NA	0.4294	0.3359	0.2588	0.2367	0.2604	NA	0.1875	NA	NA
rs148910500	T	NA	NA	NA	0.005051	NA	NA	NA	0	0	0	NA	0	NA	0	NA	NA
rs142710225	T	NA	NA	NA	0.02525	NA	NA	NA	0.01765	0	0	NA	0	NA	0	NA	NA
rs368735421	T	NA	NA	NA	0	NA	NA	NA	0	0	0	NA	0	NA	0.01442	NA	NA
rs140458174	A	NA	NA	NA	0	NA	NA	NA	0	0	0	NA	0	NA	0	NA	NA
rs532232636	T	NA	NA	NA	0	NA	NA	NA	0	0	0	NA	0	NA	0	NA	NA
rs381179	T	NA	NA	NA	0	NA	NA	NA	0	0	0	NA	0	NA	0	NA	NA
rs572410855	A	NA	NA	NA	0	NA	NA	NA	0	0	0	NA	0	NA	0	NA	NA
rs111620846	A	NA	NA	NA	0.0202	NA	NA	NA	0.005882	0	0	NA	0	NA	0	NA	NA
rs377496737	T	NA	NA	NA	0	NA	NA	NA	0	0	0	NA	0	NA	0	NA	NA
rs533552237	A	NA	NA	NA	0.005051	NA	NA	NA	0	0	0	NA	0	NA	0.004808	NA	NA
rs76315847	A	0	0	0	0	0	0	0	0	0	0	0.0004016	0	0	0	0	0
rs377348479	A	NA	NA	NA	0	NA	NA	NA	0	0	0	NA	0	NA	0	NA	NA
rs180818774	C	NA	NA	NA	0.0101	NA	NA	NA	0	0	0	NA	0	NA	0.009615	NA	NA
rs462471	A	0.4762	0.75	0.5	0.3131	0.7727	0.7167	0.5893	0.4294	0.3672	0.5059	0.2138	0.2969	0.6875	0.25	0.91667	0.6042
rs554357472	A	NA	NA	NA	0	NA	NA	NA	0	0	0	NA	0	NA	0	NA	NA
rs117652812	T	NA	NA	NA	0	NA	NA	NA	0	0	0	NA	0	NA	0	NA	NA
rs56097233	CAG	NA	NA	NA	0.4848	NA	NA	NA	0.4588	0.4688	0.5235	NA	0.375	NA	0.351	NA	NA
rs573178525	A	NA	NA	NA	0	NA	NA	NA	0	0	0.005882	NA	0	NA	0	NA	NA
rs186168224	T	NA	NA	NA	0.005051	NA	NA	NA	0	0	0	NA	0	NA	0	NA	NA
rs551103429	T	NA	NA	NA	0	NA	NA	NA	0	0	0	NA	0	NA	0	NA	NA
rs462574	A	0.4524	0.5833	0.4821	0.2273	0.7273	0.7167	0.569	0.1824	0.2422	0.4588	0.1061	0.2292	0.6875	0.1298	0.91667	0.5208
rs543681861	C	NA	NA	NA	0	NA	NA	NA	0.01176	0	0	NA	0	NA	0	NA	NA
rs557632131	T	NA	NA	NA	0	NA	NA	NA	0	0	0	NA	0	NA	0	NA	NA
rs186425543	G	NA	NA	NA	0	NA	NA	NA	0	0	0	NA	0	NA	0	NA	NA
rs542228195	C	NA	NA	NA	0	NA	NA	NA	0	0	0	NA	0	NA	0	NA	NA
rs144052153	G	NA	NA	NA	0	NA	NA	NA	0	0	0	NA	0	NA	0	NA	NA
rs374377315	T	NA	NA	NA	0	NA	NA	NA	0	0	0	NA	0	NA	0	NA	NA
rs73357664	A	NA	NA	NA	0.0303	NA	NA	NA	0.06471	0	0	NA	0	NA	0.004808	NA	NA
rs373196115	A	NA	NA	NA	0	NA	NA	NA	0	0	0	NA	0	NA	0	NA	NA
rs181107522	A	NA	NA	NA	0.0101	NA	NA	NA	0	0	0	NA	0	NA	0.004808	NA	NA
rs569716669	C	NA	NA	NA	0	NA	NA	NA	0	0	0	NA	0	NA	0	NA	NA
rs535834505	G	NA	NA	NA	0	NA	NA	NA	0	0	0	NA	0	NA	0	NA	NA
rs28707508	A	NA	NA	NA	0.303	NA	NA	NA	0.2176	0.2578	0.1471	NA	0.2031	NA	0.2837	NA	NA

SNP	Allele	Lamas	Lambayeque	Lima	LWK	Matses	Moche	Moquegua	MSL	MXL	PEL	Pelotas	PJL	Puno	PUR	Qeros	Quechuas
rs458280	T	NA	NA	NA	0	NA	NA	NA	0	0.2344	0.4529	NA	0.05729	NA	0.1058	NA	NA
rs567208488	CAG	NA	NA	NA	0	NA	NA	NA	0	0.007812	0	NA	0.01562	NA	0	NA	NA
rs186510586	C	NA	NA	NA	0	NA	NA	NA	0	0	0	NA	0	NA	0	NA	NA
rs140532244	C	NA	NA	NA	0	NA	NA	NA	0.005882	0	0	NA	0	NA	0	NA	NA
rs193019598	A	NA	NA	NA	0	NA	NA	NA	0	0	0	NA	0	NA	0	NA	NA
rs533708336	A	NA	NA	NA	0	NA	NA	NA	0	0	0	NA	0	NA	0	NA	NA
rs188198121	A	NA	NA	NA	0	NA	NA	NA	0	0	0	NA	0	NA	0	NA	NA
rs142119028	T	NA	NA	NA	0	NA	NA	NA	0.005882	0	0	NA	0	NA	0.004808	NA	NA
rs141128014	G	NA	NA	NA	0	NA	NA	NA	0	0	0	NA	0	NA	0	NA	NA
rs2838039	C	NA	NA	NA	0.4394	NA	NA	NA	0.4059	0.2266	0.07647	NA	0.276	NA	0.2212	NA	NA
rs558683527	C	NA	NA	NA	0	NA	NA	NA	0	0	0	NA	0	NA	0	NA	NA
rs545307151	G	NA	NA	NA	0.005051	NA	NA	NA	0	0	0	NA	0	NA	0	NA	NA
rs9976780	C	NA	NA	NA	0.4798	NA	NA	NA	0.4294	0.4453	0.5235	0.3046	0.3125	NA	0.351	NA	NA
rs553788077	A	NA	NA	NA	0	NA	NA	NA	0	0	0	NA	0	NA	0	NA	NA
rs539639766	C	NA	NA	NA	0	NA	NA	NA	0	0	0	NA	0	NA	0	NA	NA
rs543253134	A	NA	NA	NA	0.0101	NA	NA	NA	0.01765	0	0	NA	0	NA	0.004808	NA	NA
rs79971314	A	NA	NA	NA	0.1162	NA	NA	NA	0.2	0.007812	0.005882	NA	0	NA	0.05769	NA	NA
rs148719900	T	NA	NA	NA	0.0202	NA	NA	NA	0.02353	0	0	NA	0	NA	0.004808	NA	NA
rs144948620	A	NA	NA	NA	0	NA	NA	NA	0	0	0	NA	0	NA	0.004808	NA	NA
rs536525396	A	NA	NA	NA	0	NA	NA	NA	0	0	0	NA	0	NA	0	NA	NA
rs548341299	C	NA	NA	NA	0	NA	NA	NA	0	0	0	NA	0	NA	0	NA	NA
rs139816990	A	NA	NA	NA	0.0202	NA	NA	NA	0.005882	0	0	NA	0	NA	0	NA	NA
rs536147878	A	NA	NA	NA	0	NA	NA	NA	0	0	0	NA	0	NA	0	NA	NA
rs564793560	G	NA	NA	NA	0.01515	NA	NA	NA	0.02353	0	0	NA	0	NA	0.004808	NA	NA
rs192011400	T	NA	NA	NA	0.005051	NA	NA	NA	0	0	0	NA	0	NA	0	NA	NA
rs531024324	A	NA	NA	NA	0	NA	NA	NA	0.005882	0	0	NA	0	NA	0.01442	NA	NA
rs192666663	A	NA	NA	NA	0	NA	NA	NA	0	0	0	NA	0	NA	0	NA	NA
rs138282462	T	NA	NA	NA	0	NA	NA	NA	0	0	0	NA	0	NA	0	NA	NA
rs143109187	G	NA	NA	NA	0	NA	NA	NA	0	0	0	NA	0	NA	0	NA	NA
rs150014829	A	NA	NA	NA	0	NA	NA	NA	0	0	0	NA	0	NA	0	NA	NA
rs34561135	A	NA	NA	NA	0	NA	NA	NA	0	0.01562	0.005882	NA	0.04167	NA	0.009615	NA	NA
rs11088551	G	NA	NA	NA	0.2879	NA	NA	NA	0.2176	0.2812	0.1588	NA	0.2292	NA	0.3269	NA	NA
rs200169208	A	NA	NA	NA	0	NA	NA	NA	0	0.007812	0	NA	0	NA	0	NA	NA
rs566903241	C	NA	NA	NA	0	NA	NA	NA	0	0	0	NA	0	NA	0.004808	NA	NA
rs146132415	A	NA	NA	NA	0.02525	NA	NA	NA	0	0	0	NA	0	NA	0	NA	NA
rs61735791	T	NA	NA	NA	0	NA	NA	NA	0	0	0	NA	0	NA	0.004808	NA	NA
rs571587918	C	NA	NA	NA	0	NA	NA	NA	0	0.007812	0	NA	0	NA	0	NA	NA
rs78459594	C	0	0	0	NA	0	0	0	NA	NA	NA	NA	NA	0	NA	0	0
rs389001	G	NA	NA	NA	0.3081	NA	NA	NA	0.2647	0.2344	0.4118	NA	0.1719	NA	0.1346	NA	NA
rs200164183	T	NA	NA	NA	0	NA	NA	NA	0	0	0	NA	0	NA	0	NA	NA
rs548354821	C	NA	NA	NA	0	NA	NA	NA	0	0	0	NA	0	NA	0	NA	NA
rs2187238	C	0.381	0.1667	0.3214	0.05556	0.09091	0.1667	0.1207	0.02941	0.1719	0.2294	0.1669	0.1458	0.15	0.1875	0.08333	0.1667
rs556456416	C	NA	NA	NA	0	NA	NA	NA	0	0	0	NA	0.005208	NA	0	NA	NA
rs181414852	G	NA	NA	NA	0	NA	NA	NA	0	0	0	NA	0	NA	0	NA	NA
rs553787982	A	NA	NA	NA	0	NA	NA	NA	0	0	0	NA	0	NA	0	NA	NA

SNP	Allele	Lamas	Lambayeque	Lima	LWK	Matses	Moche	Moquegua	MSL	MXL	PEL	Pelotas	PJL	Puno	PUR	Qeros	Quechuas
rs417888	A	NA	NA	NA	0.1566	NA	NA	NA	0.07647	0.5312	0.6647	NA	0.3906	NA	0.4471	NA	NA
rs189793246	G	NA	NA	NA	0	NA	NA	NA	0	0	0	NA	0	NA	0	NA	NA
rs556371948	T	NA	NA	NA	0	NA	NA	NA	0	0	0	NA	0	NA	0	NA	NA
rs546273967	T	NA	NA	NA	0	NA	NA	NA	0	0	0	NA	0	NA	0	NA	NA
rs76000363	A	NA	NA	NA	0.03535	NA	NA	NA	0.1176	0.125	0.04706	NA	0.07292	NA	0.08654	NA	NA
rs557639119	A	NA	NA	NA	0.005051	NA	NA	NA	0.01765	0.007812	0	NA	0	NA	0	NA	NA
rs563507800	A	NA	NA	NA	0	NA	NA	NA	0	0	0	NA	0.005208	NA	0	NA	NA
rs148125094	T	NA	NA	NA	NA	NA	NA	NA	NA	NA	NA	NA	NA	NA	NA	NA	NA
rs73372161	A	NA	NA	NA	0.06566	NA	NA	NA	0.1765	0.007812	0.005882	0.02431	0	NA	0.05288	NA	NA
rs555995855	C	NA	NA	NA	0	NA	NA	NA	0	0	0	NA	0.03646	NA	0	NA	NA
rs11281229	TCCAGG	NA	NA	NA	0.3131	NA	NA	NA	0.2235	0.2578	0.1412	NA	0.1979	NA	0.3029	NA	NA
rs559098267	T	NA	NA	NA	0	NA	NA	NA	0.005882	0	0	NA	0	NA	0	NA	NA
rs191693032	G	NA	NA	NA	0	NA	NA	NA	0	0	0	NA	0	NA	0	NA	NA
rs187648498	C	NA	NA	NA	0	NA	NA	NA	0	0	0.005882	NA	0	NA	0.004808	NA	NA
rs541109823	T	NA	NA	NA	0	NA	NA	NA	0	0	0	NA	0	NA	0	NA	NA
rs559668949	G	NA	NA	NA	0	NA	NA	NA	0	0	0	NA	0.005208	NA	0	NA	NA
rs113670863	T	NA	NA	NA	0.005051	NA	NA	NA	0	0	0	NA	0	NA	0	NA	NA
rs545494331	C	NA	NA	NA	0	NA	NA	NA	0	0	0	NA	0	NA	0	NA	NA
rs528691645	A	NA	NA	NA	0.005051	NA	NA	NA	0.005882	0	0	NA	0	NA	0	NA	NA
rs558543141	T	NA	NA	NA	0	NA	NA	NA	0	0	0	NA	0	NA	0	NA	NA
rs145877432	A	NA	NA	NA	0.02525	NA	NA	NA	0	0	0	NA	0	NA	0	NA	NA
rs531615786	C	NA	NA	NA	0	NA	NA	NA	0	0	0	NA	0	NA	0	NA	NA
rs570264808	A	NA	NA	NA	0.0101	NA	NA	NA	0	0	0	NA	0	NA	0.004808	NA	NA
rs371012018	A	NA	NA	NA	0	NA	NA	NA	0	0	0	NA	0	NA	0	NA	NA
rs534326511	T	NA	NA	NA	0	NA	NA	NA	0	0	0	NA	0	NA	0	NA	NA
rs200744510	C	NA	NA	NA	NA	NA	NA	NA	NA	NA	NA	NA	NA	NA	NA	NA	NA
rs77996454	A	NA	NA	NA	0.04545	NA	NA	NA	0.05294	0.007812	0	0.007889	0	NA	0.01442	NA	NA
rs140259165	T	NA	NA	NA	0	NA	NA	NA	0	0	0	NA	0	NA	0	NA	NA
rs58978895	T	NA	NA	NA	0.101	NA	NA	NA	0.2294	0.007812	0.005882	NA	0	NA	0.04327	NA	NA
rs564981706	T	NA	NA	NA	0	NA	NA	NA	0.005882	0	0	NA	0	NA	0	NA	NA
rs149173609	T	NA	NA	NA	0	NA	NA	NA	0	0	0	NA	0	NA	0	NA	NA
rs559934709	T	NA	NA	NA	0	NA	NA	NA	0	0	0	NA	0	NA	0	NA	NA
rs569899862	A	NA	NA	NA	0	NA	NA	NA	0	0	0	NA	0	NA	0	NA	NA
rs540844961	G	NA	NA	NA	0	NA	NA	NA	0	0	0	NA	0	NA	0	NA	NA
rs189582211	A	NA	NA	NA	0	NA	NA	NA	0.005882	0	0	NA	0	NA	0	NA	NA
rs553097317	T	NA	NA	NA	0	NA	NA	NA	0	0	0	NA	0	NA	0	NA	NA
rs563693995	G	NA	NA	NA	0	NA	NA	NA	0	0	0	NA	0	NA	0	NA	NA
rs2298658	T	NA	NA	NA	0	NA	NA	NA	0	0.007812	0	NA	0	NA	0	NA	NA
rs532882572	T	NA	NA	NA	0	NA	NA	NA	0	0	0	NA	0	NA	0	NA	NA
rs145347702	C	NA	NA	NA	0.005051	NA	NA	NA	0	0	0	NA	0	NA	0	NA	NA
rs148988435	A	NA	NA	NA	0	NA	NA	NA	0	0	0	NA	0	NA	0	NA	NA
rs561557938	A	NA	NA	NA	0	NA	NA	NA	0	0	0	NA	0	NA	0	NA	NA
rs140230703	G	NA	NA	NA	0	NA	NA	NA	0	0	0	NA	0	NA	0	NA	NA
rs141232947	G	NA	NA	NA	0.005051	NA	NA	NA	0	0	0	NA	0	NA	0	NA	NA
rs61735794	T	NA	NA	NA	0	NA	NA	NA	0	0.007812	0	NA	0.01562	NA	0.01442	NA	NA

SNP	Allele	Lamas	Lambayeque	Lima	LWK	Matses	Moche	Moquegua	MSL	MXL	PEL	Pelotas	PJL	Puno	PUR	Qeros	Quechuas
rs117888036	A	NA	NA	NA	0	NA	NA	NA	0	0	0	NA	0	NA	0	NA	NA
rs538864203	A	NA	NA	NA	0	NA	NA	NA	0	0	0.005882	NA	0	NA	0	NA	NA
rs540165785	A	NA	NA	NA	0	NA	NA	NA	0	0	0	NA	0	NA	0	NA	NA
rs79517809	C	NA	NA	NA	0	NA	NA	NA	0	0	0	NA	0	NA	0	NA	NA
rs144988776	T	NA	NA	NA	0	NA	NA	NA	0	0	0	NA	0	NA	0.009615	NA	NA
rs565946159	C	NA	NA	NA	0	NA	NA	NA	0	0	0	NA	0	NA	0	NA	NA
rs574894406	G	NA	NA	NA	0	NA	NA	NA	0	0	0	NA	0	NA	0	NA	NA
rs145570856	A	NA	NA	NA	0.0303	NA	NA	NA	0.02353	0	0	NA	0	NA	0	NA	NA
rs146142989	A	NA	NA	NA	0.0101	NA	NA	NA	0	0	0	NA	0	NA	0	NA	NA
rs556966925	C	NA	NA	NA	0	NA	NA	NA	0.01176	0	0	NA	0	NA	0	NA	NA
rs573704321	T	NA	NA	NA	0	NA	NA	NA	0	0	0	NA	0	NA	0	NA	NA
rs376074355	T	NA	NA	NA	0	NA	NA	NA	0	0	0	NA	0	NA	0	NA	NA
rs61325328	G	NA	NA	NA	0	NA	NA	NA	0	0	0	NA	0	NA	0	NA	NA
rs9979311	G	NA	NA	NA	0.4495	NA	NA	NA	0.3941	0.2266	0.07647	NA	0.276	NA	0.2163	NA	NA
rs538655114	A	NA	NA	NA	0	NA	NA	NA	0.005882	0	0	NA	0	NA	0	NA	NA
rs150066796	A	NA	NA	NA	0	NA	NA	NA	0	0	0	NA	0	NA	0	NA	NA
rs201093031	G	NA	NA	NA	0	NA	NA	NA	0	0	0	NA	0	NA	0	NA	NA
rs378616	C	NA	NA	NA	0.2677	NA	NA	NA	0.4118	0.2891	0.1824	0.2919	0.2708	NA	0.2067	NA	NA
rs573152591	A	NA	NA	NA	0	NA	NA	NA	0	0	0	NA	0	NA	0	NA	NA
rs4818241	A	NA	NA	NA	0.0101	NA	NA	NA	0.01176	0.2188	0.4412	NA	0.03646	NA	0.1346	NA	NA
rs189181802	T	NA	NA	NA	0	NA	NA	NA	0.005882	0	0	NA	0	NA	0	NA	NA
rs566323147	A	NA	NA	NA	0	NA	NA	NA	0	0	0	NA	0	NA	0	NA	NA
rs184164037	C	NA	NA	NA	0	NA	NA	NA	0.04118	0	0	NA	0	NA	0.004808	NA	NA
rs77675406	A	0.02381	0.08333	0.01786	0.03535	0.04545	0	0.01724	0.08235	0.125	0.04706	0.08664	0.07292	0	0.08173	0	0.08333
rs528276471	G	NA	NA	NA	0.005051	NA	NA	NA	0	0	0	NA	0	NA	0	NA	NA
rs552912416	T	NA	NA	NA	0	NA	NA	NA	0	0	0	NA	0	NA	0	NA	NA
rs572279833	A	NA	NA	NA	0	NA	NA	NA	0	0	0	NA	0	NA	0	NA	NA
rs559830930	G	NA	NA	NA	0	NA	NA	NA	0	0	0.02353	NA	0	NA	0	NA	NA
rs2298660	T	NA	NA	NA	0.2778	NA	NA	NA	0.4588	0.3125	0.2588	NA	0.1719	NA	0.1875	NA	NA
rs527684898	G	NA	NA	NA	0	NA	NA	NA	0	0	0	NA	0	NA	0	NA	NA
rs181332845	G	NA	NA	NA	0	NA	NA	NA	0.005882	0	0	NA	0	NA	0.004808	NA	NA
rs182175876	G	NA	NA	NA	0	NA	NA	NA	0	0	0	NA	0	NA	0	NA	NA
rs539218228	A	NA	NA	NA	0	NA	NA	NA	0	0	0	NA	0	NA	0	NA	NA
rs116865960	A	NA	NA	NA	0.005051	NA	NA	NA	0	0.007812	0.01176	NA	0.01042	NA	0.02404	NA	NA
rs183398094	C	NA	NA	NA	0	NA	NA	NA	0	0	0	NA	0	NA	0.004808	NA	NA
rs567340892	A	NA	NA	NA	0	NA	NA	NA	0.005882	0	0	NA	0	NA	0	NA	NA
rs190125027	T	NA	NA	NA	0	NA	NA	NA	0	0	0	NA	0	NA	0	NA	NA
rs75430506	A	NA	NA	NA	0	NA	NA	NA	0	0	0	NA	0	NA	0	NA	NA
rs138812485	A	NA	NA	NA	0	NA	NA	NA	0	0	0	NA	0	NA	0	NA	NA
rs56066678	T	0.3095	0.2083	0.2679	0.3687	0	0.1333	0.2414	0.4529	0.2031	0.2471	0.2951	0.2917	0.15	0.3077	0.08333	0.25
rs561063944	A	NA	NA	NA	0	NA	NA	NA	0.005882	0	0	NA	0	NA	0	NA	NA
rs17854725	G	NA	NA	NA	0.2374	NA	NA	NA	0.3706	0.4766	0.4118	NA	0.4531	NA	0.4135	NA	NA
rs533050705	A	NA	NA	NA	0	NA	NA	NA	0	0	0	NA	0	NA	0	NA	NA
rs545910743	C	NA	NA	NA	0	NA	NA	NA	0	0	0	NA	0	NA	0	NA	NA
rs149021153	G	NA	NA	NA	0.0404	NA	NA	NA	0.005882	0	0	NA	0	NA	0.004808	NA	NA

SNP	Allele	Lamas	Lambayeque	Lima	LWK	Matses	Moche	Moquegua	MSL	MXL	PEL	Pelotas	PJL	Puno	PUR	Qeros	Quechuas
rs371583288	A	NA	NA	NA	0	NA	NA	NA	0	0	0	NA	0	NA	0	NA	NA
rs552174617	C	NA	NA	NA	0	NA	NA	NA	0	0	0	NA	0.005208	NA	0	NA	NA
rs144157441	A	NA	NA	NA	0.04545	NA	NA	NA	0.05294	0	0	NA	0	NA	0	NA	NA
rs559811756	A	NA	NA	NA	0	NA	NA	NA	0.03529	0	0	NA	0	NA	0.004808	NA	NA
rs536642840	A	NA	NA	NA	0	NA	NA	NA	0	0	0	NA	0	NA	0	NA	NA
rs572530227	T	NA	NA	NA	0	NA	NA	NA	0	0	0.005882	NA	0	NA	0	NA	NA
rs551045243	A	NA	NA	NA	0	NA	NA	NA	0	0	0	NA	0	NA	0	NA	NA
rs374886738	C	NA	NA	NA	0.0101	NA	NA	NA	0.01765	0	0	NA	0	NA	0	NA	NA
rs557714443	A	NA	NA	NA	0	NA	NA	NA	0	0	0.005882	NA	0	NA	0	NA	NA
rs402303	C	0.5	0.2917	0.4643	0.4495	0.2273	0.2667	0.3621	0.3353	0.5078	0.4706	0.6512	0.6354	0.3	0.625	0.08333	0.3542
rs544822322	C	NA	NA	NA	0	NA	NA	NA	0	0	0	NA	0	NA	0	NA	NA
rs186429734	A	NA	NA	NA	0.005051	NA	NA	NA	0	0	0	NA	0	NA	0	NA	NA
rs138056981	T	NA	NA	NA	0	NA	NA	NA	0	0	0	NA	0	NA	0.004808	NA	NA
rs79468500	G	NA	NA	NA	0.0202	NA	NA	NA	0.005882	0	0.005882	NA	0.01562	NA	0.02885	NA	NA
rs2156301	T	NA	NA	NA	0.0101	NA	NA	NA	0.01765	0.2188	0.4471	NA	0.03646	NA	0.1346	NA	NA
rs191228684	T	NA	NA	NA	0	NA	NA	NA	0	0	0	NA	0	NA	0	NA	NA
rs563812695	A	NA	NA	NA	0	NA	NA	NA	0	0	0	NA	0	NA	0	NA	NA
rs8128074	T	0.1429	0.1667	0.125	0.01515	0.09091	0.1	0.2931	0.04118	0.1641	0.1353	0.1172	0.1562	0.2875	0.07692	0.3333	0.1739
rs560788815	C	NA	NA	NA	0	NA	NA	NA	0	0	0	NA	0	NA	0	NA	NA
rs183851466	T	NA	NA	NA	0	NA	NA	NA	0	0	0	NA	0	NA	0	NA	NA
rs151152524	C	NA	NA	NA	0.01515	NA	NA	NA	0.005882	0	0	NA	0	NA	0	NA	NA
rs113506821	T	0.09524	0.08333	0.05357	0	0.04545	0.03333	0.03448	0	0.09375	0.08235	0.03285	0.02604	0.0625	0.02404	0	0.0625
rs530527676	A	NA	NA	NA	0	NA	NA	NA	0	0	0	NA	0	NA	0	NA	NA
rs550252022	C	NA	NA	NA	0	NA	NA	NA	0	0	0	NA	0	NA	0	NA	NA
rs386638	C	NA	NA	NA	0.02525	NA	NA	NA	0.01176	0.2344	0.4471	NA	0.05729	NA	0.1346	NA	NA
rs148136016	G	NA	NA	NA	0	NA	NA	NA	0	0	0	NA	0	NA	0	NA	NA
rs114848359	T	NA	NA	NA	0.0101	NA	NA	NA	0	0	0	NA	0	NA	0.009615	NA	NA
rs530009764	C	NA	NA	NA	0	NA	NA	NA	0	0	0	NA	0	NA	0	NA	NA
rs371965055	T	NA	NA	NA	0	NA	NA	NA	0	0	0	NA	0.06771	NA	0	NA	NA
rs183984610	A	NA	NA	NA	0	NA	NA	NA	0	0	0	NA	0	NA	0	NA	NA
rs527305819	A	NA	NA	NA	0	NA	NA	NA	0	0	0	NA	0	NA	0	NA	NA
rs181461974	T	NA	NA	NA	0.0101	NA	NA	NA	0	0	0	NA	0	NA	0	NA	NA
rs566457884	T	NA	NA	NA	0	NA	NA	NA	0.005882	0	0	NA	0	NA	0	NA	NA
rs540070564	A	NA	NA	NA	0	NA	NA	NA	0	0	0.005882	NA	0	NA	0	NA	NA
rs563555305	T	NA	NA	NA	0	NA	NA	NA	0	0.007812	0	NA	0	NA	0	NA	NA
rs151189718	T	NA	NA	NA	0.01515	NA	NA	NA	0	0.007812	0	NA	0	NA	0	NA	NA
rs569681994	C	NA	NA	NA	0	NA	NA	NA	0	0	0	NA	0	NA	0	NA	NA
rs143097165	A	NA	NA	NA	0	NA	NA	NA	0.03529	0	0	NA	0	NA	0.004808	NA	NA
rs541200538	G	NA	NA	NA	0	NA	NA	NA	0	0	0	NA	0	NA	0	NA	NA
rs11701576	G	NA	NA	NA	0.1212	NA	NA	NA	0.1824	0.05469	0.02941	NA	0.224	NA	0.1346	NA	NA
rs17001042	A	NA	NA	NA	0.05556	NA	NA	NA	0.1294	0.007812	0	NA	0	NA	0.03365	NA	NA
rs558449333	T	NA	NA	NA	0	NA	NA	NA	0	0	0	NA	0.005208	NA	0	NA	NA
rs569866330	A	NA	NA	NA	0	NA	NA	NA	0	0	0	NA	0	NA	0	NA	NA
rs573234046	T	NA	NA	NA	0	NA	NA	NA	0	0	0	NA	0	NA	0	NA	NA
rs114363287	T	NA	NA	NA	0.005051	NA	NA	NA	0.005882	0	0	NA	0	NA	0	NA	NA

SNP	Allele	Lamas	Lambayeque	Lima	LWK	Matses	Moche	Moquegua	MSL	MXL	PEL	Pelotas	PJL	Puno	PUR	Qeros	Quechuas
rs55896064	A	NA	NA	NA	0.03535	NA	NA	NA	0.08824	0.125	0.04706	NA	0.07292	NA	0.08173	NA	NA
rs547961708	T	NA	NA	NA	0	NA	NA	NA	0	0	0	NA	0	NA	0	NA	NA
rs542712985	G	NA	NA	NA	0	NA	NA	NA	0.005882	0	0	NA	0	NA	0	NA	NA
rs117941520	A	NA	NA	NA	0	NA	NA	NA	0	0.007812	0.005882	NA	0.005208	NA	0.004808	NA	NA
rs73903404	G	NA	NA	NA	0.02525	NA	NA	NA	0.05882	0	0	NA	0	NA	0.01442	NA	NA
rs551817968	T	NA	NA	NA	0	NA	NA	NA	0	0	0	NA	0	NA	0	NA	NA
rs550208798	T	NA	NA	NA	0	NA	NA	NA	0	0	0	NA	0	NA	0	NA	NA
rs542471574	A	NA	NA	NA	0.005051	NA	NA	NA	0	0	0	NA	0.02083	NA	0	NA	NA
rs145841350	T	NA	NA	NA	0	NA	NA	NA	0	0	0	NA	0	NA	0	NA	NA
rs7278627	A	0	0.04167	0	0.06061	0	0	0	0.1765	0	0.005882	0.02331	0	0	0.03846	0	0
rs185555838	A	NA	NA	NA	0.005051	NA	NA	NA	0	0	0	NA	0	NA	0	NA	NA
rs374666536	T	NA	NA	NA	0	NA	NA	NA	0	0	0	NA	0	NA	0	NA	NA
rs7278739	A	NA	NA	NA	0.1919	NA	NA	NA	0.3176	0.007812	0.01176	NA	0	NA	0.07692	NA	NA
rs555667242	C	NA	NA	NA	0.005051	NA	NA	NA	0	0	0	NA	0	NA	0	NA	NA
rs7364083	G	NA	NA	NA	0.1465	NA	NA	NA	0.1294	0.4688	0.6471	NA	0.4479	NA	0.4038	NA	NA
rs527284833	G	NA	NA	NA	0	NA	NA	NA	0.03529	0	0	NA	0	NA	0.004808	NA	NA
rs139458208	C	NA	NA	NA	0	NA	NA	NA	0	0	0	NA	0	NA	0	NA	NA
rs139001727	G	NA	NA	NA	0	NA	NA	NA	0	0.007812	0	NA	0	NA	0	NA	NA
rs4290734	G	NA	NA	NA	0.06566	NA	NA	NA	0.03529	0.3438	0.2294	NA	0.4062	NA	0.3269	NA	NA
rs2156300	G	NA	NA	NA	0.0101	NA	NA	NA	0.01176	0.2188	0.4471	NA	0.03646	NA	0.1346	NA	NA
rs555328523	T	NA	NA	NA	0	NA	NA	NA	0	0	0	NA	0	NA	0	NA	NA
rs575168295	G	NA	NA	NA	0	NA	NA	NA	0	0	0	NA	0	NA	0	NA	NA
rs145355824	A	NA	NA	NA	0	NA	NA	NA	0	0	0	NA	0	NA	0	NA	NA
rs375827195	G	NA	NA	NA	0	NA	NA	NA	0	0	0	NA	0.02083	NA	0	NA	NA
rs533805020	T	NA	NA	NA	0	NA	NA	NA	0	0	0	NA	0	NA	0	NA	NA
rs536311940	G	NA	NA	NA	0	NA	NA	NA	0.005882	0	0	NA	0	NA	0	NA	NA
rs371046741	A	NA	NA	NA	0.005051	NA	NA	NA	0.01765	0	0	NA	0	NA	0	NA	NA
rs2838042	C	0.02381	0.08333	0.03571	0.1566	0.04545	0.01667	0.05172	0.2294	0.1484	0.06471	0.1893	0.3333	0.0375	0.1346	0	0.0625
rs192334131	A	NA	NA	NA	0	NA	NA	NA	0	0	0	NA	0.01042	NA	0	NA	NA
rs557822084	T	NA	NA	NA	0	NA	NA	NA	0	0	0	NA	0	NA	0	NA	NA
rs566208761	C	NA	NA	NA	0	NA	NA	NA	0	0	0	NA	0.005208	NA	0	NA	NA
rs546335233	A	NA	NA	NA	0	NA	NA	NA	0	0	0	NA	0	NA	0	NA	NA
rs184767756	C	NA	NA	NA	0	NA	NA	NA	0	0	0	NA	0	NA	0	NA	NA
rs528259213	A	NA	NA	NA	0	NA	NA	NA	0	0	0	NA	0	NA	0	NA	NA
rs115265507	A	NA	NA	NA	0.04545	NA	NA	NA	0.01176	0	0	NA	0	NA	0.004808	NA	NA
rs200072801	G	NA	NA	NA	0	NA	NA	NA	0	0	0	NA	0	NA	0	NA	NA
rs138765307	T	NA	NA	NA	0	NA	NA	NA	0	0.007812	0	NA	0	NA	0	NA	NA
rs532005855	C	NA	NA	NA	0	NA	NA	NA	0	0	0	NA	0	NA	0	NA	NA
rs2838043	T	NA	NA	NA	0.0303	NA	NA	NA	0.02941	0.1719	0.2294	NA	0.1354	NA	0.1875	NA	NA
rs115596471	A	NA	NA	NA	0.05051	NA	NA	NA	0.05882	0.007812	0	NA	0	NA	0.009615	NA	NA
rs574538362	T	NA	NA	NA	0	NA	NA	NA	0	0	0	NA	0	NA	0	NA	NA
rs190618812	A	NA	NA	NA	0	NA	NA	NA	0	0	0	NA	0	NA	0	NA	NA
rs141583878	A	NA	NA	NA	0	NA	NA	NA	0	0	0	NA	0	NA	0	NA	NA
rs571805265	T	NA	NA	NA	0	NA	NA	NA	0	0	0	NA	0	NA	0	NA	NA
rs371497524	A	NA	NA	NA	0	NA	NA	NA	0	0	0	NA	0	NA	0	NA	NA

SNP	Allele	Lamas	Lambayeque	Lima	LWK	Matses	Moche	Moquegua	MSL	MXL	PEL	Pelotas	PJL	Puno	PUR	Qeros	Quechuas
rs540908584	T	NA	NA	NA	0	NA	NA	NA	0	0	0.01176	NA	0	NA	0	NA	NA
rs73357642	A	NA	NA	NA	0.1465	NA	NA	NA	0.2588	0.007812	0	NA	0	NA	0.03846	NA	NA
rs192854367	A	NA	NA	NA	0	NA	NA	NA	0	0	0	NA	0	NA	0.004808	NA	NA
rs371180501	G	NA	NA	NA	0	NA	NA	NA	0.02353	0	0.005882	NA	0	NA	0	NA	NA
rs467375	A	0.09524	0	0.125	0.07071	0.04545	0.05	0.1379	0.05294	0.2344	0.1765	0.3419	0.3958	0.125	0.274	0	0.125
rs562045100	A	NA	NA	NA	0	NA	NA	NA	0	0	0	NA	0	NA	0	NA	NA
rs34205539	AT	NA	NA	NA	0.03535	NA	NA	NA	0.02941	0.02344	0.01176	NA	0.125	NA	0.03846	NA	NA
rs186418926	A	NA	NA	NA	0	NA	NA	NA	0	0	0	NA	0	NA	0	NA	NA
rs539528622	T	NA	NA	NA	0	NA	NA	NA	0	0	0	NA	0	NA	0	NA	NA
rs145292327	G	NA	NA	NA	0.005051	NA	NA	NA	0	0	0	NA	0	NA	0	NA	NA
rs372405355	C	NA	NA	NA	0	NA	NA	NA	0	0	0	NA	0	NA	0	NA	NA
rs544417878	T	NA	NA	NA	0	NA	NA	NA	0	0	0	NA	0	NA	0	NA	NA
rs559363556	A	NA	NA	NA	0	NA	NA	NA	0	0	0	NA	0	NA	0	NA	NA
rs570467504	C	NA	NA	NA	0	NA	NA	NA	0	0	0	NA	0	NA	0	NA	NA
rs148701953	C	NA	NA	NA	0.005051	NA	NA	NA	0	0	0	NA	0	NA	0	NA	NA
rs569988060	A	NA	NA	NA	0	NA	NA	NA	0	0	0	NA	0	NA	0.004808	NA	NA
rs182254633	G	NA	NA	NA	0.005051	NA	NA	NA	0.005882	0	0	NA	0	NA	0	NA	NA
rs563305848	C	NA	NA	NA	0	NA	NA	NA	0	0	0	NA	0	NA	0	NA	NA
rs528477904	C	NA	NA	NA	0	NA	NA	NA	0	0	0	NA	0	NA	0	NA	NA
rs550874821	G	NA	NA	NA	0	NA	NA	NA	0.005882	0	0	NA	0	NA	0	NA	NA
rs73372193	C	NA	NA	NA	0.1111	NA	NA	NA	0.2471	0.007812	0.005882	NA	0	NA	0.06731	NA	NA
rs552607028	A	NA	NA	NA	0	NA	NA	NA	0	0	0	NA	0	NA	0	NA	NA
rs553861191	T	NA	NA	NA	0	NA	NA	NA	0	0	0	NA	0	NA	0	NA	NA
rs147977027	C	NA	NA	NA	0	NA	NA	NA	0	0	0	NA	0	NA	0	NA	NA
rs533445403	T	NA	NA	NA	0	NA	NA	NA	0.005882	0	0	NA	0	NA	0	NA	NA
rs578005531	T	NA	NA	NA	0	NA	NA	NA	0	0	0	NA	0	NA	0	NA	NA
rs140605461	A	NA	NA	NA	0	NA	NA	NA	0	0	0	NA	0	NA	0	NA	NA
rs544023509	A	NA	NA	NA	0	NA	NA	NA	0	0	0	NA	0	NA	0	NA	NA
rs61735792	A	NA	NA	NA	0	NA	NA	NA	0	0	0	NA	0	NA	0.009615	NA	NA
rs568256706	G	NA	NA	NA	0	NA	NA	NA	0	0	0	NA	0	NA	0	NA	NA
rs8131648	T	NA	NA	NA	0.4091	NA	NA	NA	0.4706	0.5312	0.6882	NA	0.3177	NA	0.3173	NA	NA
rs527964326	C	NA	NA	NA	0	NA	NA	NA	0	0	0	NA	0	NA	0	NA	NA
rs191697229	A	NA	NA	NA	0.02525	NA	NA	NA	0.02353	0	0	NA	0	NA	0	NA	NA
rs147359020	A	NA	NA	NA	0	NA	NA	NA	0	0	0	NA	0.03646	NA	0.009615	NA	NA
rs532085624	T	NA	NA	NA	0	NA	NA	NA	0	0	0	NA	0	NA	0	NA	NA
rs577753477	A	NA	NA	NA	0	NA	NA	NA	0	0	0	NA	0	NA	0	NA	NA
rs181091055	C	NA	NA	NA	0	NA	NA	NA	0	0	0	NA	0	NA	0	NA	NA
rs546447704	T	NA	NA	NA	0	NA	NA	NA	0	0	0	NA	0	NA	0	NA	NA
rs575335056	CA	NA	NA	NA	0	NA	NA	NA	0	0	0	NA	0.01562	NA	0	NA	NA
rs536763630	A	NA	NA	NA	0	NA	NA	NA	0	0	0	NA	0	NA	0	NA	NA
rs184500277	T	NA	NA	NA	0	NA	NA	NA	0	0	0	NA	0	NA	0	NA	NA
rs569292495	G	NA	NA	NA	0	NA	NA	NA	0	0	0	NA	0	NA	0.01442	NA	NA
rs73372163	A	NA	NA	NA	0.1717	NA	NA	NA	0.3353	0.1328	0.05294	NA	0.06771	NA	0.1394	NA	NA
rs554443322	C	NA	NA	NA	0	NA	NA	NA	0	0	0	NA	0	NA	0	NA	NA
rs568346732	T	NA	NA	NA	0	NA	NA	NA	0	0	0	NA	0	NA	0	NA	NA

SNP	Allele	Lamas	Lambayeque	Lima	LWK	Matses	Moche	Moquegua	MSL	MXL	PEL	Pelotas	PJL	Puno	PUR	Qeros	Quechuas
rs114641598	C	NA	NA	NA	0.02525	NA	NA	NA	0	0	0	NA	0	NA	0	NA	NA
rs549459413	A	NA	NA	NA	0	NA	NA	NA	0	0	0	NA	0.005208	NA	0	NA	NA
rs184380117	A	NA	NA	NA	0	NA	NA	NA	0	0	0	NA	0	NA	0	NA	NA
rs576181035	G	NA	NA	NA	0	NA	NA	NA	0	0	0	NA	0	NA	0	NA	NA
rs201679623	C	NA	NA	NA	0	NA	NA	NA	0	0	0	NA	0	NA	0	NA	NA
rs568846685	A	NA	NA	NA	0	NA	NA	NA	0	0	0	NA	0	NA	0	NA	NA
rs548718178	C	NA	NA	NA	0	NA	NA	NA	0.01765	0	0	NA	0	NA	0	NA	NA
rs537584838	G	NA	NA	NA	0	NA	NA	NA	0	0	0.005882	NA	0	NA	0	NA	NA
rs569259137	A	NA	NA	NA	0	NA	NA	NA	0	0	0	NA	0	NA	0	NA	NA
rs367885466	T	NA	NA	NA	0	NA	NA	NA	0.005882	0	0	NA	0	NA	0.004808	NA	NA
rs2410430	A	NA	NA	NA	0.0202	NA	NA	NA	0.01765	0.2188	0.4471	NA	0.03646	NA	0.1346	NA	NA
rs559637785	A	NA	NA	NA	0	NA	NA	NA	0	0	0	NA	0	NA	0	NA	NA
rs573343651	C	NA	NA	NA	0	NA	NA	NA	0	0	0	NA	0	NA	0	NA	NA
rs34769294	CA	NA	NA	NA	0.2121	NA	NA	NA	0.1235	0.2031	0.2471	NA	0.2969	NA	0.3077	NA	NA
rs7364088	A	NA	NA	NA	0.298	NA	NA	NA	0.3412	0.3281	0.2588	NA	0.2656	NA	0.1875	NA	NA
rs551432766	T	NA	NA	NA	0	NA	NA	NA	0	0	0	NA	0	NA	0	NA	NA
rs565299100	G	NA	NA	NA	0	NA	NA	NA	0	0	0	NA	0	NA	0	NA	NA
rs538252674	C	NA	NA	NA	0	NA	NA	NA	0	0	0	NA	0	NA	0	NA	NA
rs368994585	A	NA	NA	NA	0	NA	NA	NA	0	0	0	NA	0	NA	0	NA	NA
rs192709500	A	NA	NA	NA	0	NA	NA	NA	0.005882	0	0	NA	0	NA	0	NA	NA
rs192955773	C	NA	NA	NA	0	NA	NA	NA	0.03529	0	0	NA	0	NA	0.004808	NA	NA
rs371020678	T	NA	NA	NA	0	NA	NA	NA	0	0	0	NA	0	NA	0	NA	NA
rs556435793	A	NA	NA	NA	0	NA	NA	NA	0	0	0	NA	0.005208	NA	0	NA	NA
rs549990870	G	NA	NA	NA	0	NA	NA	NA	0	0	0	NA	0	NA	0	NA	NA
rs146723217	T	NA	NA	NA	0.03535	NA	NA	NA	0.01765	0	0	NA	0	NA	0.009615	NA	NA
rs145738510	C	NA	NA	NA	0	NA	NA	NA	0	0	0	NA	0	NA	0	NA	NA
rs114844880	C	NA	NA	NA	0	NA	NA	NA	0	0	0	NA	0	NA	0	NA	NA
rs370641046	T	NA	NA	NA	0	NA	NA	NA	0	0	0	NA	0	NA	0.009615	NA	NA
rs528452128	GTGTTT	NA	NA	NA	0.0303	NA	NA	NA	0	0	0	NA	0	NA	0.009615	NA	NA
rs11910678	C	0	0	0	0.1566	0	0	0.01724	0.1294	0.01562	0	0.03081	0	0	0.02404	0	0
rs140625413	C	NA	NA	NA	0.005051	NA	NA	NA	0	0	0	NA	0	NA	0	NA	NA
rs142444476	T	NA	NA	NA	0.005051	NA	NA	NA	0	0	0	NA	0	NA	0	NA	NA
rs545149163	A	NA	NA	NA	0	NA	NA	NA	0	0	0	NA	0	NA	0	NA	NA
rs149798653	C	NA	NA	NA	0.0202	NA	NA	NA	0.02353	0	0	NA	0	NA	0	NA	NA
rs527416684	A	NA	NA	NA	0	NA	NA	NA	0	0	0	NA	0.02083	NA	0	NA	NA
rs189067157	A	NA	NA	NA	0	NA	NA	NA	0	0	0	NA	0	NA	0	NA	NA
rs527489879	C	NA	NA	NA	0	NA	NA	NA	0	0	0	NA	0	NA	0	NA	NA
rs558824762	T	NA	NA	NA	0.0101	NA	NA	NA	0.02941	0	0	NA	0	NA	0.009615	NA	NA
rs370927492	T	NA	NA	NA	0	NA	NA	NA	0	0	0	NA	0	NA	0	NA	NA
rs1003030	G	NA	NA	NA	0.1212	NA	NA	NA	0.1941	0.05469	0.02941	NA	0.224	NA	0.1346	NA	NA
rs543908797	A	NA	NA	NA	0	NA	NA	NA	0	0	0	NA	0	NA	0	NA	NA
rs567103273	G	NA	NA	NA	0.0101	NA	NA	NA	0	0	0	NA	0	NA	0	NA	NA
rs565820920	T	NA	NA	NA	0	NA	NA	NA	0	0	0	NA	0	NA	0	NA	NA
rs548463596	G	NA	NA	NA	0.4747	NA	NA	NA	0.3765	0.3516	0.3118	NA	0.2552	NA	0.2692	NA	NA
rs561570726	T	NA	NA	NA	0	NA	NA	NA	0	0	0.005882	NA	0	NA	0	NA	NA

SNP	Allele	Lamas	Lambayeque	Lima	LWK	Matses	Moche	Moquegua	MSL	MXL	PEL	Pelotas	PJL	Puno	PUR	Qeros	Quechuas
rs555911696	A	NA	NA	NA	0	NA	NA	NA	0	0	0	NA	0	NA	0	NA	NA
rs561179495	T	NA	NA	NA	0	NA	NA	NA	0	0	0	NA	0	NA	0	NA	NA
rs181972300	A	NA	NA	NA	0	NA	NA	NA	0	0	0	NA	0	NA	0	NA	NA
rs138498737	A	NA	NA	NA	0	NA	NA	NA	0	0	0	NA	0.005208	NA	0.01442	NA	NA
rs565468881	T	NA	NA	NA	0	NA	NA	NA	0.01765	0	0	NA	0	NA	0	NA	NA
rs186734573	T	NA	NA	NA	0	NA	NA	NA	0	0	0	NA	0	NA	0	NA	NA
rs146132480	A	NA	NA	NA	0	NA	NA	NA	0	0.007812	0.01176	NA	0	NA	0.009615	NA	NA
rs559125514	C	NA	NA	NA	0	NA	NA	NA	0	0	0	NA	0	NA	0	NA	NA
rs527499027	T	NA	NA	NA	0	NA	NA	NA	0	0	0	NA	0	NA	0	NA	NA
rs573736906	A	NA	NA	NA	0	NA	NA	NA	0	0	0	NA	0.03125	NA	0	NA	NA
rs573613070	G	NA	NA	NA	0	NA	NA	NA	0	0	0	NA	0	NA	0	NA	NA
rs542898542	T	NA	NA	NA	0	NA	NA	NA	0	0	0	NA	0	NA	0	NA	NA
rs422471	C	0.5	0.7083	0.5357	0.4646	0.7727	0.7333	0.6379	0.4412	0.4844	0.5294	0.3318	0.375	0.7	0.3558	0.91667	0.6458
rs181058683	A	NA	NA	NA	0	NA	NA	NA	0	0	0	NA	0.01562	NA	0	NA	NA
rs149855493	C	NA	NA	NA	0	NA	NA	NA	0.03529	0	0	NA	0	NA	0.004808	NA	NA
rs191457025	T	NA	NA	NA	0	NA	NA	NA	0	0	0	NA	0	NA	0	NA	NA
rs375760	T	NA	NA	NA	0.1515	NA	NA	NA	0.08824	0.2031	0.2353	NA	0.2812	NA	0.274	NA	NA
rs148038688	T	NA	NA	NA	0	NA	NA	NA	0.03529	0	0	NA	0	NA	0.004808	NA	NA
rs145728087	A	NA	NA	NA	0.0101	NA	NA	NA	0.01765	0	0	NA	0	NA	0	NA	NA
rs569947342	T	NA	NA	NA	0	NA	NA	NA	0	0	0	NA	0	NA	0	NA	NA
rs562599408	G	NA	NA	NA	0	NA	NA	NA	0.005882	0	0	NA	0	NA	0	NA	NA
rs146252393	A	NA	NA	NA	0.005051	NA	NA	NA	0	0	0	NA	0	NA	0	NA	NA
rs73372182	A	NA	NA	NA	0.1263	NA	NA	NA	0.2529	0.007812	0.005882	NA	0	NA	0.04808	NA	NA
rs566012796	C	NA	NA	NA	0	NA	NA	NA	0	0	0	NA	0	NA	0	NA	NA
rs74423429	A	NA	NA	NA	0	NA	NA	NA	0	0.007812	0	NA	0	NA	0.01923	NA	NA
rs193067129	A	NA	NA	NA	0	NA	NA	NA	0	0	0	NA	0	NA	0	NA	NA
rs572143040	C	NA	NA	NA	0	NA	NA	NA	0	0	0	NA	0	NA	0	NA	NA
rs9984012	T	NA	NA	NA	0.005051	NA	NA	NA	0.005882	0.08594	0.03529	NA	0.07812	NA	0.07692	NA	NA
rs141898436	C	NA	NA	NA	0.01515	NA	NA	NA	0.005882	0	0.005882	NA	0	NA	0	NA	NA
rs558272207	A	NA	NA	NA	0	NA	NA	NA	0	0	0	NA	0.01042	NA	0	NA	NA
rs146681599	A	NA	NA	NA	0.005051	NA	NA	NA	0	0	0	NA	0	NA	0	NA	NA
rs743542	A	0.4286	0.08333	0.1964	0.1364	0.1818	0.1667	0.1034	0.1882	0.1719	0.2	0.08795	0.1719	0.1625	0.05769	0.08333	0.1667
rs532511763	T	NA	NA	NA	0	NA	NA	NA	0.005882	0	0	NA	0	NA	0	NA	NA
rs149424945	T	NA	NA	NA	0	NA	NA	NA	0	0	0	NA	0	NA	0	NA	NA
rs568605816	A	NA	NA	NA	0	NA	NA	NA	0	0	0	NA	0	NA	0	NA	NA
rs184859933	T	NA	NA	NA	0	NA	NA	NA	0	0	0	NA	0	NA	0.02404	NA	NA
rs530918384	GGTGA	NA	NA	NA	0	NA	NA	NA	0.005882	0.007812	0.005882	NA	0	NA	0.004808	NA	NA
rs28369457	T	NA	NA	NA	0	NA	NA	NA	0	0	0	NA	0	NA	0	NA	NA
rs556192760	A	NA	NA	NA	0	NA	NA	NA	0	0	0	NA	0	NA	0	NA	NA
rs576447507	C	NA	NA	NA	0	NA	NA	NA	0	0.007812	0	NA	0	NA	0	NA	NA
rs546512831	A	NA	NA	NA	0	NA	NA	NA	0	0	0	NA	0	NA	0	NA	NA
rs543066660	A	NA	NA	NA	0	NA	NA	NA	0	0	0	NA	0	NA	0	NA	NA
rs543404937	T	NA	NA	NA	0	NA	NA	NA	0	0	0	NA	0	NA	0	NA	NA
rs180826598	C	NA	NA	NA	0	NA	NA	NA	0	0	0	NA	0	NA	0.009615	NA	NA
rs368878191	T	NA	NA	NA	0	NA	NA	NA	0	0	0	NA	0	NA	0	NA	NA

SNP	Allele	Lamas	Lambayeque	Lima	LWK	Matses	Moche	Moquegua	MSL	MXL	PEL	Pelotas	PJL	Puno	PUR	Qeros	Quechuas
rs369619813	G	NA	NA	NA	0	NA	NA	NA	0.005882	0	0	NA	0	NA	0	NA	NA
rs112213575	A	NA	NA	NA	0.0202	NA	NA	NA	0.01176	0	0	NA	0	NA	0	NA	NA
rs548725701	A	NA	NA	NA	0	NA	NA	NA	0	0	0	NA	0	NA	0	NA	NA
rs569959837	A	NA	NA	NA	0	NA	NA	NA	0	0	0	NA	0	NA	0	NA	NA
rs554986094	A	NA	NA	NA	0	NA	NA	NA	0	0	0	NA	0	NA	0	NA	NA
rs456142	T	NA	NA	NA	0.3182	NA	NA	NA	0.4294	0.3672	0.5059	0.2092	0.2969	NA	0.25	NA	NA
rs73357644	T	NA	NA	NA	0.1515	NA	NA	NA	0.2588	0.007812	0	NA	0	NA	0.03846	NA	NA
rs576259678	C	NA	NA	NA	0	NA	NA	NA	0	0	0	NA	0	NA	0	NA	NA
rs140141551	A	NA	NA	NA	0	NA	NA	NA	0	0.007812	0	NA	0	NA	0.01923	NA	NA
rs146564124	T	NA	NA	NA	0	NA	NA	NA	0	0	0	NA	0.005208	NA	0	NA	NA
rs558715322	T	NA	NA	NA	0	NA	NA	NA	0	0	0	NA	0	NA	0	NA	NA
rs116020930	A	NA	NA	NA	0	NA	NA	NA	0	0	0	NA	0	NA	0	NA	NA
rs539333191	T	NA	NA	NA	0	NA	NA	NA	0	0	0	NA	0	NA	0	NA	NA
rs192259532	T	NA	NA	NA	0	NA	NA	NA	0	0	0	NA	0	NA	0.004808	NA	NA
rs531920590	T	NA	NA	NA	0	NA	NA	NA	0	0	0	NA	0.005208	NA	0.009615	NA	NA
rs531300154	C	NA	NA	NA	0	NA	NA	NA	0	0	0	NA	0	NA	0	NA	NA
rs147945120	T	NA	NA	NA	0	NA	NA	NA	0	0	0	NA	0	NA	0	NA	NA
rs147934552	A	NA	NA	NA	0	NA	NA	NA	0	0	0	NA	0	NA	0	NA	NA
rs112980967	A	NA	NA	NA	0.04545	NA	NA	NA	0.01176	0	0	NA	0	NA	0.004808	NA	NA
rs149601802	T	NA	NA	NA	0	NA	NA	NA	0	0	0	NA	0	NA	0	NA	NA
rs3761373	T	NA	NA	NA	0.1212	NA	NA	NA	0.2	0.05469	0.02941	0.09298	0.224	NA	0.1346	NA	NA
rs545333396	C	NA	NA	NA	0	NA	NA	NA	0	0	0	NA	0	NA	0	NA	NA
rs574683527	A	NA	NA	NA	0	NA	NA	NA	0	0	0	NA	0.005208	NA	0	NA	NA
rs189431131	C	NA	NA	NA	0	NA	NA	NA	0	0	0	NA	0	NA	0.004808	NA	NA
rs183147330	A	NA	NA	NA	0	NA	NA	NA	0	0	0	NA	0	NA	0	NA	NA
rs417443	A	NA	NA	NA	0.0101	NA	NA	NA	0.01176	0.2266	0.4353	NA	0.05729	NA	0.1394	NA	NA
rs564403172	T	NA	NA	NA	0	NA	NA	NA	0	0	0	NA	0	NA	0	NA	NA
rs8134216	T	0.1905	0.25	0.3393	0.5404	0.2273	0.1667	0.2931	0.4471	0.4688	0.3059	0.696	0.6823	0.175	0.6587	0	0.2083
rs191441931	T	NA	NA	NA	0	NA	NA	NA	0	0	0	NA	0	NA	0	NA	NA
rs567188556	T	NA	NA	NA	0	NA	NA	NA	0	0	0	NA	0	NA	0	NA	NA
rs187662898	T	NA	NA	NA	0	NA	NA	NA	0	0.007812	0	NA	0	NA	0	NA	NA
rs114911304	A	NA	NA	NA	0.005051	NA	NA	NA	0	0	0	NA	0	NA	0.009615	NA	NA
rs558858943	C	NA	NA	NA	0	NA	NA	NA	0	0	0	NA	0	NA	0	NA	NA
rs541396507	C	NA	NA	NA	0	NA	NA	NA	0	0	0	NA	0	NA	0	NA	NA
rs565142599	T	NA	NA	NA	0	NA	NA	NA	0	0	0	NA	0	NA	0	NA	NA
rs146605032	A	NA	NA	NA	0.005051	NA	NA	NA	0	0	0	NA	0	NA	0	NA	NA
rs553673225	G	NA	NA	NA	0	NA	NA	NA	0	0	0	NA	0	NA	0	NA	NA
rs113034290	T	NA	NA	NA	0.005051	NA	NA	NA	0.005882	0	0	NA	0	NA	0.01923	NA	NA
rs150554820	T	NA	NA	NA	NA	NA	NA	NA	NA	NA	NA	NA	NA	NA	NA	NA	NA
rs544880781	A	NA	NA	NA	0	NA	NA	NA	0	0	0	NA	0	NA	0	NA	NA
rs6517669	A	0.95238	0.875	0.94643	0.3788	0.95455	0.96667	0.93103	0.3235	0.7344	0.91765	0.6948	0.6823	1	0.75	1	0.95833
rs550238535	A	NA	NA	NA	0	NA	NA	NA	0	0	0	NA	0	NA	0	NA	NA
rs455922	T	NA	NA	NA	0.005051	NA	NA	NA	0	0.2344	0.4529	NA	0.05729	NA	0.1058	NA	NA
rs374203194	A	NA	NA	NA	0.005051	NA	NA	NA	0	0	0	NA	0	NA	0	NA	NA
rs111671182	G	NA	NA	NA	0.0303	NA	NA	NA	0.02353	0	0	NA	0	NA	0	NA	NA

SNP	Allele	Lamas	Lambayeque	Lima	LWK	Matses	Moche	Moquegua	MSL	MXL	PEL	Pelotas	PJL	Puno	PUR	Qeros	Quechuas
rs181044447	A	NA	NA	NA	0	NA	NA	NA	0	0	0	NA	0	NA	0	NA	NA
rs573737830	A	NA	NA	NA	0	NA	NA	NA	0	0	0	NA	0	NA	0	NA	NA
rs181778800	A	NA	NA	NA	0	NA	NA	NA	0	0	0	NA	0	NA	0.004808	NA	NA
rs577217013	C	NA	NA	NA	0	NA	NA	NA	0	0	0	NA	0	NA	0	NA	NA
rs563948676	A	NA	NA	NA	0	NA	NA	NA	0	0	0	NA	0	NA	0	NA	NA
rs141888586	T	NA	NA	NA	0.1768	NA	NA	NA	0.08235	0.007812	0	NA	0	NA	0.009615	NA	NA
rs146797606	C	NA	NA	NA	0	NA	NA	NA	0.03529	0	0	NA	0	NA	0.004808	NA	NA
rs544037190	C	NA	NA	NA	0	NA	NA	NA	0	0	0	NA	0	NA	0	NA	NA
rs535041396	T	NA	NA	NA	0	NA	NA	NA	0	0	0	NA	0	NA	0	NA	NA
rs534591508	T	NA	NA	NA	0	NA	NA	NA	0	0	0	NA	0	NA	0	NA	NA
rs547758146	A	NA	NA	NA	0	NA	NA	NA	0	0	0	NA	0	NA	0.004808	NA	NA
rs529424146	A	NA	NA	NA	0	NA	NA	NA	0	0	0	NA	0	NA	0	NA	NA
rs563946938	T	NA	NA	NA	0	NA	NA	NA	0	0	0	NA	0	NA	0	NA	NA
rs545056304	A	NA	NA	NA	0	NA	NA	NA	0	0	0	NA	0	NA	0	NA	NA
rs12481984	C	NA	NA	NA	0.2929	NA	NA	NA	0.2176	0.2734	0.1529	NA	0.2135	NA	0.3365	NA	NA
rs538226704	T	NA	NA	NA	0	NA	NA	NA	0	0	0	NA	0	NA	0	NA	NA
rs548678297	G	NA	NA	NA	0	NA	NA	NA	0	0	0	NA	0	NA	0	NA	NA
rs149676870	T	NA	NA	NA	0	NA	NA	NA	0	0.1094	0.1765	NA	0	NA	0.004808	NA	NA
rs78217567	C	NA	NA	NA	0	NA	NA	NA	0	0.02344	0.005882	NA	0.02604	NA	0.02404	NA	NA
rs558791980	A	NA	NA	NA	0	NA	NA	NA	0	0	0	NA	0	NA	0	NA	NA
rs34256269	A	NA	NA	NA	0.005051	NA	NA	NA	0.005882	0.07812	0.03529	NA	0.08854	NA	0.07692	NA	NA
rs74749793	T	0	0.08333	0.05357	0.1263	0	0	0.01786	0.1941	0.05469	0.02941	0.09258	0.2292	0.0125	0.1346	0	0.0625
rs193253965	C	NA	NA	NA	0	NA	NA	NA	0	0	0	NA	0	NA	0	NA	NA
rs373622147	C	NA	NA	NA	0	NA	NA	NA	0	0	0	NA	0	NA	0	NA	NA
rs559691670	A	NA	NA	NA	0	NA	NA	NA	0	0	0	NA	0	NA	0	NA	NA
rs534317621	A	NA	NA	NA	0	NA	NA	NA	0	0	0	NA	0	NA	0	NA	NA
rs539981354	G	NA	NA	NA	0.005051	NA	NA	NA	0	0	0	NA	0	NA	0	NA	NA
rs115266855	G	NA	NA	NA	0.01515	NA	NA	NA	0.02353	0	0	NA	0	NA	0.01442	NA	NA
rs149109132	A	NA	NA	NA	0.005051	NA	NA	NA	0.04118	0	0	NA	0	NA	0.02404	NA	NA
rs557656729	A	NA	NA	NA	0	NA	NA	NA	0	0	0	NA	0	NA	0	NA	NA
rs57161767	A	NA	NA	NA	0.0303	NA	NA	NA	0.03529	0	0	NA	0	NA	0.004808	NA	NA
rs574708698	T	NA	NA	NA	0	NA	NA	NA	0	0	0	NA	0	NA	0	NA	NA
rs4303795	G	NA	NA	NA	0.2929	NA	NA	NA	0.2176	0.2812	0.1588	NA	0.2292	NA	0.3269	NA	NA
rs145662876	T	NA	NA	NA	0	NA	NA	NA	0	0	0	NA	0	NA	0	NA	NA
rs566146941	A	NA	NA	NA	0	NA	NA	NA	0	0	0	NA	0.005208	NA	0	NA	NA
rs563595766	T	NA	NA	NA	0	NA	NA	NA	0.005882	0	0	NA	0	NA	0	NA	NA
rs572507505	T	NA	NA	NA	0	NA	NA	NA	0	0	0	NA	0	NA	0	NA	NA
rs557606231	G	NA	NA	NA	0	NA	NA	NA	0.01765	0	0	NA	0	NA	0	NA	NA
rs11911394	T	0.95238	0.875	0.94643	0.3838	0.95455	0.96667	0.93103	0.3235	0.7344	0.91765	0.697	0.6823	1	0.75	1	0.95833
rs76973757	A	NA	NA	NA	0.05556	NA	NA	NA	0.05294	0.007812	0	NA	0	NA	0.01442	NA	NA
rs573484758	G	NA	NA	NA	0	NA	NA	NA	0	0	0	NA	0.005208	NA	0	NA	NA
rs530471976	T	NA	NA	NA	0	NA	NA	NA	0	0	0	NA	0	NA	0.004808	NA	NA
rs372286621	A	NA	NA	NA	0	NA	NA	NA	0	0	0	NA	0	NA	0	NA	NA
rs4818239	C	NA	NA	NA	0.2273	NA	NA	NA	0.2529	0.3438	0.2353	NA	0.4167	NA	0.375	NA	NA
rs186484871	C	NA	NA	NA	0	NA	NA	NA	0	0	0	NA	0	NA	0	NA	NA

SNP	Allele	Lamas	Lambayeque	Lima	LWK	Matses	Moche	Moquegua	MSL	MXL	PEL	Pelotas	PJL	Puno	PUR	Qeros	Quechuas
rs542575868	A	NA	NA	NA	0	NA	NA	NA	0	0	0	NA	0	NA	0	NA	NA
rs111572592	A	NA	NA	NA	0	NA	NA	NA	0	0	0	NA	0.005208	NA	0.01442	NA	NA
rs144576889	G	NA	NA	NA	0	NA	NA	NA	0.03529	0	0	NA	0	NA	0.004808	NA	NA
rs572265353	A	NA	NA	NA	0	NA	NA	NA	0	0	0	NA	0	NA	0	NA	NA
rs191368876	C	NA	NA	NA	0	NA	NA	NA	0	0	0	NA	0	NA	0	NA	NA
rs535472251	CAAAAAAAAAA	NA	NA	NA	0.2677	NA	NA	NA	0.3059	0.2578	0.2176	NA	0.276	NA	0.3077	NA	NA
rs141788162	A	NA	NA	NA	0	NA	NA	NA	0.005882	0	0	NA	0	NA	0.01923	NA	NA
rs56695953	A	NA	NA	NA	0.0303	NA	NA	NA	0.02941	0.1797	0.2294	NA	0.1354	NA	0.1875	NA	NA
rs548680244	T	NA	NA	NA	0	NA	NA	NA	0	0	0	NA	0	NA	0	NA	NA
rs430915	A	0.6429	0.6667	0.6786	0.1364	0.95455	0.8333	0.7069	0.07059	0.5312	0.6765	0.4307	0.3958	0.85	0.4327	0.91667	0.7083
rs564795317	A	NA	NA	NA	0	NA	NA	NA	0	0	0	NA	0	NA	0	NA	NA
rs577731955	C	NA	NA	NA	0	NA	NA	NA	0	0	0	NA	0	NA	0	NA	NA
rs369342013	A	NA	NA	NA	0	NA	NA	NA	0	0	0	NA	0	NA	0	NA	NA
rs542402063	A	NA	NA	NA	0	NA	NA	NA	0	0	0	NA	0	NA	0	NA	NA
rs556381293	C	NA	NA	NA	0	NA	NA	NA	0.005882	0	0	NA	0	NA	0	NA	NA
rs534924497	T	NA	NA	NA	0	NA	NA	NA	0	0	0	NA	0	NA	0	NA	NA
rs554462605	A	NA	NA	NA	0	NA	NA	NA	0	0	0	NA	0	NA	0	NA	NA
rs80027429	A	NA	NA	NA	0	NA	NA	NA	0	0	0	NA	0.01042	NA	0.004808	NA	NA
rs561192574	T	NA	NA	NA	0	NA	NA	NA	0	0	0	NA	0	NA	0	NA	NA
rs455281	G	NA	NA	NA	0.2222	NA	NA	NA	0.1647	0.25	0.4588	NA	0.2292	NA	0.1346	NA	NA
rs9975782	A	NA	NA	NA	0	NA	NA	NA	0	0	0	NA	0.005208	NA	0	NA	NA
rs569362310	G	NA	NA	NA	0	NA	NA	NA	0	0	0	NA	0	NA	0	NA	NA
rs536278410	C	NA	NA	NA	0	NA	NA	NA	0	0	0	NA	0.005208	NA	0	NA	NA
rs415918	C	NA	NA	NA	0.4747	NA	NA	NA	0.4412	0.4688	0.5235	NA	0.375	NA	0.351	NA	NA
rs554890599	A	NA	NA	NA	0	NA	NA	NA	0	0	0	NA	0	NA	0	NA	NA
rs530721239	G	NA	NA	NA	0	NA	NA	NA	0	0	0	NA	0	NA	0	NA	NA
rs552589240	G	NA	NA	NA	0	NA	NA	NA	0	0	0	NA	0	NA	0	NA	NA
rs545504807	C	NA	NA	NA	0	NA	NA	NA	0	0	0	NA	0	NA	0	NA	NA
rs28403625	A	NA	NA	NA	0	NA	NA	NA	0	0	0	NA	0	NA	0	NA	NA
rs532159311	C	NA	NA	NA	0	NA	NA	NA	0	0	0	NA	0	NA	0	NA	NA
rs543578752	G	NA	NA	NA	0	NA	NA	NA	0	0	0	NA	0.005208	NA	0	NA	NA
rs576215861	A	NA	NA	NA	0	NA	NA	NA	0	0	0	NA	0	NA	0.004808	NA	NA
rs199824558	A	NA	NA	NA	0	NA	NA	NA	0	0	0	NA	0.005208	NA	0	NA	NA
rs562000328	C	NA	NA	NA	0.005051	NA	NA	NA	0	0	0	NA	0	NA	0	NA	NA
rs559698510	T	NA	NA	NA	0	NA	NA	NA	0	0	0	NA	0	NA	0	NA	NA
rs75168613	A	NA	NA	NA	0.1364	NA	NA	NA	0.1765	0.007812	0.005882	NA	0.005208	NA	0.02885	NA	NA
rs543344542	T	NA	NA	NA	0	NA	NA	NA	0	0	0	NA	0.005208	NA	0	NA	NA
rs570889046	A	NA	NA	NA	0	NA	NA	NA	0.005882	0	0	NA	0	NA	0.009615	NA	NA
rs548267325	A	NA	NA	NA	0	NA	NA	NA	0	0	0	NA	0	NA	0	NA	NA
rs35899679	A	NA	NA	NA	0.1162	NA	NA	NA	0.06471	0.3047	0.2294	NA	0.3333	NA	0.3029	NA	NA
rs561575371	A	NA	NA	NA	0	NA	NA	NA	0	0	0	NA	0	NA	0	NA	NA
rs577554253	A	NA	NA	NA	0	NA	NA	NA	0	0	0	NA	0.005208	NA	0	NA	NA
rs535407146	A	NA	NA	NA	0	NA	NA	NA	0	0	0	NA	0	NA	0	NA	NA
rs149024920	G	NA	NA	NA	0.02525	NA	NA	NA	0.01765	0	0	NA	0	NA	0	NA	NA
rs112209215	A	NA	NA	NA	0.02525	NA	NA	NA	0.005882	0	0	NA	0	NA	0	NA	NA

SNP	Allele	Lamas	Lambayeque	Lima	LWK	Matses	Moche	Moquegua	MSL	MXL	PEL	Pelotas	PJL	Puno	PUR	Qeros	Quechuas
rs183405398	G	NA	NA	NA	0	NA	NA	NA	0	0	0	NA	0	NA	0	NA	NA
rs374510753	T	NA	NA	NA	0	NA	NA	NA	0	0	0	NA	0	NA	0	NA	NA
rs75200570	C	NA	NA	NA	0.1162	NA	NA	NA	0.1941	0.03125	0	NA	0.05208	NA	0.01442	NA	NA
rs149275684	T	NA	NA	NA	0	NA	NA	NA	0	0	0	NA	0	NA	0	NA	NA
rs547544037	A	NA	NA	NA	0	NA	NA	NA	0	0	0	NA	0	NA	0	NA	NA
rs553755767	A	NA	NA	NA	0	NA	NA	NA	0	0	0	NA	0	NA	0	NA	NA
rs76855393	T	NA	NA	NA	0	NA	NA	NA	0	0	0	NA	0	NA	0	NA	NA
rs118028230	C	NA	NA	NA	NA	NA	NA	NA	NA	NA	NA	NA	NA	NA	NA	NA	NA
rs184764113	A	NA	NA	NA	0	NA	NA	NA	0	0	0	NA	0	NA	0.004808	NA	NA
rs541351488	A	NA	NA	NA	0	NA	NA	NA	0	0	0	NA	0	NA	0	NA	NA
rs185078457	T	NA	NA	NA	0	NA	NA	NA	0	0	0	NA	0	NA	0	NA	NA
rs542961770	T	NA	NA	NA	0	NA	NA	NA	0	0	0	NA	0	NA	0	NA	NA
rs562131885	A	NA	NA	NA	0.02525	NA	NA	NA	0.01176	0	0	NA	0	NA	0.009615	NA	NA
rs536399456	A	NA	NA	NA	0	NA	NA	NA	0	0	0	NA	0	NA	0	NA	NA
rs547523412	A	NA	NA	NA	0	NA	NA	NA	0	0	0	NA	0	NA	0	NA	NA
rs534034788	A	NA	NA	NA	0	NA	NA	NA	0	0	0	NA	0.01562	NA	0	NA	NA
rs544784789	G	NA	NA	NA	0	NA	NA	NA	0	0	0	NA	0	NA	0	NA	NA
rs540987630	T	NA	NA	NA	0	NA	NA	NA	0	0	0	NA	0	NA	0	NA	NA
rs141478137	G	NA	NA	NA	0	NA	NA	NA	0.005882	0	0	NA	0	NA	0	NA	NA
rs5844077	G	NA	NA	NA	0.1212	NA	NA	NA	0.1353	0.2578	0.1706	NA	0.2396	NA	0.1731	NA	NA
rs551681725	G	NA	NA	NA	0	NA	NA	NA	0	0	0	NA	0	NA	0.004808	NA	NA
rs548592256	C	NA	NA	NA	0	NA	NA	NA	0	0	0	NA	0	NA	0	NA	NA
rs568689321	A	NA	NA	NA	0	NA	NA	NA	0	0	0	NA	0	NA	0	NA	NA
rs555302570	A	NA	NA	NA	0	NA	NA	NA	0	0	0	NA	0	NA	0	NA	NA
rs537212420	G	NA	NA	NA	0	NA	NA	NA	0	0	0.005882	NA	0	NA	0	NA	NA
rs547186777	C	NA	NA	NA	0.005051	NA	NA	NA	0	0	0	NA	0	NA	0	NA	NA
rs532627816	A	NA	NA	NA	0	NA	NA	NA	0	0	0	NA	0	NA	0	NA	NA
rs150314077	A	NA	NA	NA	0	NA	NA	NA	0	0	0	NA	0	NA	0.02404	NA	NA
rs544983081	A	NA	NA	NA	0	NA	NA	NA	0	0	0	NA	0	NA	0	NA	NA
rs140121827	T	NA	NA	NA	0	NA	NA	NA	0	0	0	NA	0	NA	0	NA	NA
rs74605993	T	NA	NA	NA	0.03535	NA	NA	NA	0.005882	0	0	NA	0	NA	0.004808	NA	NA
rs7279603	C	0	0.1667	0.1786	0.5	0	0.06667	0.1552	0.4824	0.1953	0.1059	0.3592	0.2865	0.025	0.4279	0	0.02083
rs535611212	G	NA	NA	NA	0	NA	NA	NA	0.01765	0	0.005882	NA	0	NA	0.004808	NA	NA
rs180792570	T	NA	NA	NA	0	NA	NA	NA	0	0	0	NA	0	NA	0	NA	NA
rs180784757	A	NA	NA	NA	0	NA	NA	NA	0.03529	0	0	NA	0	NA	0.004808	NA	NA
rs532756922	A	NA	NA	NA	0	NA	NA	NA	0	0	0	NA	0	NA	0	NA	NA
rs138651919	A	NA	NA	NA	0	NA	NA	NA	0	0	0	NA	0	NA	0	NA	NA
rs549212787	G	NA	NA	NA	0	NA	NA	NA	0	0	0	NA	0	NA	0.01442	NA	NA
rs538863731	C	NA	NA	NA	0	NA	NA	NA	0	0	0	NA	0	NA	0	NA	NA
rs538803792	T	NA	NA	NA	0	NA	NA	NA	0	0.007812	0	NA	0	NA	0	NA	NA
rs574890845	A	NA	NA	NA	0	NA	NA	NA	0	0	0	NA	0	NA	0	NA	NA
rs546165791	T	NA	NA	NA	0	NA	NA	NA	0	0.01562	0.005882	NA	0	NA	0	NA	NA
rs554530868	G	NA	NA	NA	0	NA	NA	NA	0	0.007812	0	NA	0.04167	NA	0.004808	NA	NA
rs79512425	C	NA	NA	NA	0	NA	NA	NA	0	0	0	NA	0	NA	0	NA	NA
rs542395007	T	NA	NA	NA	0	NA	NA	NA	0.005882	0	0	NA	0	NA	0.01442	NA	NA

SNP	Allele	Lamas	Lambayeque	Lima	LWK	Matses	Moche	Moquegua	MSL	MXL	PEL	Pelotas	PJL	Puno	PUR	Qeros	Quechuas
rs556249043	A	NA	NA	NA	0	NA	NA	NA	0	0	0	NA	0	NA	0	NA	NA
rs529550811	A	NA	NA	NA	0	NA	NA	NA	0	0	0	NA	0	NA	0	NA	NA
rs533931558	A	NA	NA	NA	0	NA	NA	NA	0	0	0	NA	0	NA	0	NA	NA
rs565902164	AACC	NA	NA	NA	0	NA	NA	NA	0	0	0	NA	0.01042	NA	0	NA	NA
rs530084541	G	NA	NA	NA	0.005051	NA	NA	NA	0	0	0	NA	0	NA	0	NA	NA
rs549593213	T	NA	NA	NA	0	NA	NA	NA	0	0	0	NA	0	NA	0	NA	NA
rs189570078	C	NA	NA	NA	0	NA	NA	NA	0	0.007812	0	NA	0	NA	0	NA	NA
rs190682490	T	NA	NA	NA	0	NA	NA	NA	0	0	0	NA	0	NA	0.004808	NA	NA
rs577570888	T	NA	NA	NA	0	NA	NA	NA	0	0	0	NA	0	NA	0.004808	NA	NA
rs376403654	A	NA	NA	NA	0	NA	NA	NA	0	0	0	NA	0	NA	0	NA	NA
rs576016640	T	NA	NA	NA	0	NA	NA	NA	0	0	0	NA	0	NA	0	NA	NA
rs567552202	C	NA	NA	NA	0	NA	NA	NA	0	0	0	NA	0.005208	NA	0	NA	NA
rs140793040	A	NA	NA	NA	0	NA	NA	NA	0	0	0	NA	0.005208	NA	0	NA	NA
rs146445857	TGCC	NA	NA	NA	0	NA	NA	NA	0	0	0	NA	0	NA	0	NA	NA
rs547401659	T	NA	NA	NA	0	NA	NA	NA	0	0	0	NA	0	NA	0	NA	NA
rs571396442	T	NA	NA	NA	0	NA	NA	NA	0	0	0.005882	NA	0	NA	0	NA	NA
rs185406661	G	NA	NA	NA	0	NA	NA	NA	0	0	0	NA	0	NA	0	NA	NA
rs2070789	T	NA	NA	NA	0.3889	NA	NA	NA	0.4412	0.3359	0.2588	NA	0.2604	NA	0.1875	NA	NA
rs7277080	T	NA	NA	NA	0.2576	NA	NA	NA	0.2294	0.2578	0.1353	NA	0.2135	NA	0.2981	NA	NA
rs183385443	A	NA	NA	NA	0.005051	NA	NA	NA	0	0	0	NA	0	NA	0	NA	NA
rs181592444	T	NA	NA	NA	0	NA	NA	NA	0	0	0	NA	0	NA	0	NA	NA
rs189832305	T	NA	NA	NA	0	NA	NA	NA	0	0	0	NA	0	NA	0	NA	NA
rs734056	A	0.1905	0.2083	0.2143	0.197	0.2273	0.1333	0.1379	0.2412	0.3516	0.2294	0.4071	0.401	0.15	0.3365	0	0.1042
rs561819256	G	NA	NA	NA	0	NA	NA	NA	0	0	0	NA	0	NA	0.009615	NA	NA
rs2070786	C	0	0.125	0.1786	0.4646	0	0.06667	0.1552	0.3176	0.1953	0.1	0.3382	0.2865	0.02564	0.3894	0	0.02083
rs541155775	C	NA	NA	NA	0	NA	NA	NA	0	0	0	NA	0	NA	0	NA	NA
rs544315388	A	NA	NA	NA	0	NA	NA	NA	0	0	0	NA	0	NA	0	NA	NA
rs191763249	G	NA	NA	NA	0.005051	NA	NA	NA	0	0	0	NA	0	NA	0	NA	NA
rs137962614	T	NA	NA	NA	0	NA	NA	NA	0	0	0	NA	0	NA	0	NA	NA
rs464431	A	NA	NA	NA	0.03535	NA	NA	NA	0.02353	0.2344	0.4529	NA	0.05729	NA	0.1106	NA	NA
rs147054075	A	NA	NA	NA	0.005051	NA	NA	NA	0	0	0	NA	0.01042	NA	0	NA	NA
rs190516010	A	NA	NA	NA	0	NA	NA	NA	0	0	0	NA	0	NA	0	NA	NA
rs542265380	T	NA	NA	NA	0	NA	NA	NA	0	0	0	NA	0.005208	NA	0	NA	NA
rs188263047	A	NA	NA	NA	0	NA	NA	NA	0	0	0	NA	0	NA	0	NA	NA
rs143148203	G	NA	NA	NA	0.005051	NA	NA	NA	0	0	0	NA	0	NA	0	NA	NA
rs557853844	A	NA	NA	NA	0	NA	NA	NA	0.005882	0	0	NA	0	NA	0	NA	NA
rs571275590	G	NA	NA	NA	0	NA	NA	NA	0	0	0	NA	0	NA	0	NA	NA
rs562173027	C	NA	NA	NA	0	NA	NA	NA	0	0	0	NA	0	NA	0	NA	NA
rs530253044	T	NA	NA	NA	0	NA	NA	NA	0	0	0	NA	0	NA	0	NA	NA
rs532694622	C	NA	NA	NA	0	NA	NA	NA	0	0	0	NA	0	NA	0	NA	NA
rs75756279	T	NA	NA	NA	0	NA	NA	NA	0	0	0	NA	0.01042	NA	0.004808	NA	NA
rs570835062	T	NA	NA	NA	0	NA	NA	NA	0	0	0	NA	0.005208	NA	0	NA	NA
rs530942388	G	NA	NA	NA	0	NA	NA	NA	0.01765	0	0	NA	0	NA	0	NA	NA
rs575313753	G	NA	NA	NA	0.005051	NA	NA	NA	0.005882	0	0	NA	0	NA	0	NA	NA
rs537370123	T	NA	NA	NA	0	NA	NA	NA	0	0	0.005882	NA	0	NA	0	NA	NA

SNP	Allele	Lamas	Lambayeque	Lima	LWK	Matses	Moche	Moquegua	MSL	MXL	PEL	Pelotas	PJL	Puno	PUR	Qeros	Quechuas
rs185946013	A	NA	NA	NA	0	NA	NA	NA	0	0.007812	0	NA	0	NA	0	NA	NA
rs142518005	T	NA	NA	NA	0.0303	NA	NA	NA	0.005882	0	0	NA	0	NA	0.009615	NA	NA
rs558104085	A	NA	NA	NA	0.005051	NA	NA	NA	0	0	0.005882	NA	0	NA	0	NA	NA
rs144800717	T	NA	NA	NA	0.005051	NA	NA	NA	0	0	0	NA	0	NA	0	NA	NA
rs57474639	T	0.02381	0.08333	0.01786	0.07576	0.04545	0	0.01724	0.1412	0.125	0.05294	0.08919	0.06771	0	0.08173	0	0.08696
rs538139969	T	NA	NA	NA	0	NA	NA	NA	0	0	0	NA	0	NA	0	NA	NA
rs567302726	A	NA	NA	NA	0	NA	NA	NA	0	0	0	NA	0.02604	NA	0	NA	NA
rs187052796	T	NA	NA	NA	0	NA	NA	NA	0.03529	0	0	NA	0	NA	0.004808	NA	NA
rs551164492	G	NA	NA	NA	0	NA	NA	NA	0.005882	0	0	NA	0	NA	0	NA	NA
rs143562195	A	NA	NA	NA	0	NA	NA	NA	0.01765	0	0	NA	0	NA	0	NA	NA
rs11088550	A	NA	NA	NA	0	NA	NA	NA	0	0.125	0.04706	NA	0.02604	NA	0.05288	NA	NA
rs151338439	A	NA	NA	NA	0	NA	NA	NA	0	0	0	NA	0	NA	0	NA	NA
rs185726773	A	NA	NA	NA	0	NA	NA	NA	0	0	0	NA	0	NA	0	NA	NA
rs531410451	A	NA	NA	NA	0	NA	NA	NA	0	0	0	NA	0	NA	0	NA	NA
rs2257202	C	NA	NA	NA	0.1717	NA	NA	NA	0.08235	0.2031	0.2412	NA	0.2865	NA	0.2837	NA	NA
rs117827300	A	NA	NA	NA	0	NA	NA	NA	0	0	0	NA	0	NA	0	NA	NA
rs187208295	C	NA	NA	NA	0	NA	NA	NA	0	0	0	NA	0	NA	0	NA	NA
rs61735793	A	0	0	0	0	0	0	0	0	0	0	0.00455	0.01042	0	0.004808	0	0
rs143712818	T	NA	NA	NA	0	NA	NA	NA	0.03529	0	0	NA	0	NA	0.004808	NA	NA
rs542575245	T	NA	NA	NA	0	NA	NA	NA	0	0	0.005882	NA	0	NA	0	NA	NA
rs551797163	C	NA	NA	NA	0	NA	NA	NA	0	0	0	NA	0	NA	0	NA	NA
rs571050655	C	NA	NA	NA	0	NA	NA	NA	0	0	0	NA	0	NA	0	NA	NA
rs34624090	AC	NA	NA	NA	0.07576	NA	NA	NA	0.05294	0.2344	0.1824	NA	0.3958	NA	0.274	NA	NA
rs147099383	C	NA	NA	NA	0.01515	NA	NA	NA	0.04118	0.007812	0.005882	NA	0.1042	NA	0.01442	NA	NA
rs58146697	C	0	0.04167	0	0.2576	0	0	0.03448	0.2176	0.01562	0	0.05624	0.04167	0	0.04327	0	0
rs139700775	C	NA	NA	NA	0	NA	NA	NA	0	0.007812	0.005882	NA	0	NA	0	NA	NA
rs560183935	C	NA	NA	NA	0.005051	NA	NA	NA	0.005882	0	0	NA	0	NA	0	NA	NA
rs182330267	A	NA	NA	NA	0.0101	NA	NA	NA	0.01176	0	0	NA	0	NA	0	NA	NA
rs565879862	C	NA	NA	NA	0	NA	NA	NA	0	0	0	NA	0	NA	0	NA	NA
rs188500480	T	NA	NA	NA	0	NA	NA	NA	0	0	0	NA	0	NA	0	NA	NA
rs533556786	G	NA	NA	NA	0	NA	NA	NA	0	0	0	NA	0.01562	NA	0	NA	NA
rs551106657	T	NA	NA	NA	0	NA	NA	NA	0	0	0	NA	0	NA	0	NA	NA
rs116606027	A	NA	NA	NA	0	NA	NA	NA	0.03529	0	0	NA	0	NA	0.004808	NA	NA
rs573715927	A	NA	NA	NA	0	NA	NA	NA	0	0	0	NA	0	NA	0	NA	NA
rs145026350	T	NA	NA	NA	0	NA	NA	NA	0	0	0	NA	0	NA	0	NA	NA
rs915823	C	0.04762	0.04167	0.01786	0.1162	0.04545	0.01667	0.01724	0.1412	0.1562	0.05882	0.1588	0.1406	0	0.125	0	0.04167
rs188495261	A	NA	NA	NA	0	NA	NA	NA	0	0	0	NA	0	NA	0	NA	NA
rs563800355	T	NA	NA	NA	0	NA	NA	NA	0	0	0	NA	0.005208	NA	0	NA	NA
rs143060022	A	NA	NA	NA	0	NA	NA	NA	0	0	0	NA	0	NA	0	NA	NA
rs527811791	C	NA	NA	NA	0	NA	NA	NA	0	0	0	NA	0	NA	0	NA	NA
rs554692214	C	NA	NA	NA	0	NA	NA	NA	0	0	0	NA	0	NA	0	NA	NA
rs8129582	A	NA	NA	NA	0.005051	NA	NA	NA	0	0	0	NA	0	NA	0	NA	NA
rs532513568	C	NA	NA	NA	0.005051	NA	NA	NA	0	0	0	NA	0	NA	0	NA	NA
rs537357445	C	NA	NA	NA	0	NA	NA	NA	0	0	0	NA	0	NA	0	NA	NA
rs538591883	C	NA	NA	NA	0	NA	NA	NA	0	0	0	NA	0	NA	0	NA	NA

SNP	Allele	Lamas	Lambayeque	Lima	LWK	Matses	Moche	Moquegua	MSL	MXL	PEL	Pelotas	PJL	Puno	PUR	Qeros	Quechuas
rs535599066	T	NA	NA	NA	0	NA	NA	NA	0	0	0	NA	0	NA	0	NA	NA
rs7275220	G	NA	NA	NA	0.5	NA	NA	NA	0.5647	0.4375	0.6882	0.3565	0.3594	NA	0.4615	NA	NA
rs199636550	G	NA	NA	NA	0	NA	NA	NA	0	0	0	NA	0	NA	0	NA	NA
rs117696554	A	NA	NA	NA	0	NA	NA	NA	0	0	0	NA	0.01562	NA	0.01442	NA	NA
rs137871202	C	NA	NA	NA	0.0101	NA	NA	NA	0	0	0	NA	0	NA	0	NA	NA
rs555056776	A	NA	NA	NA	0	NA	NA	NA	0	0	0	NA	0	NA	0	NA	NA
rs553934496	T	NA	NA	NA	0	NA	NA	NA	0	0	0	NA	0	NA	0	NA	NA
rs547701911	C	NA	NA	NA	0	NA	NA	NA	0	0	0	NA	0	NA	0	NA	NA
rs187460831	A	NA	NA	NA	0	NA	NA	NA	0	0	0	NA	0	NA	0	NA	NA
rs422761	A	NA	NA	NA	0.3081	NA	NA	NA	0.2647	0.2344	0.4118	NA	0.1667	NA	0.1346	NA	NA
rs186422083	C	NA	NA	NA	0	NA	NA	NA	0	0	0	NA	0	NA	0	NA	NA
rs544946928	T	NA	NA	NA	0	NA	NA	NA	0	0	0	NA	0.01562	NA	0	NA	NA
rs542577849	A	NA	NA	NA	0	NA	NA	NA	0	0	0	NA	0	NA	0	NA	NA
rs561308071	T	NA	NA	NA	0	NA	NA	NA	0	0	0	NA	0	NA	0	NA	NA
rs184146774	G	NA	NA	NA	0	NA	NA	NA	0	0	0	NA	0	NA	0	NA	NA
rs181446489	C	NA	NA	NA	0	NA	NA	NA	0	0.007812	0	NA	0	NA	0	NA	NA
rs543518445	T	NA	NA	NA	0	NA	NA	NA	0	0	0	NA	0	NA	0	NA	NA
rs566732470	A	NA	NA	NA	0	NA	NA	NA	0	0	0	NA	0	NA	0	NA	NA
rs533571483	A	NA	NA	NA	0	NA	NA	NA	0	0	0	NA	0	NA	0	NA	NA
rs531148133	A	NA	NA	NA	0	NA	NA	NA	0	0	0	NA	0	NA	0	NA	NA
rs113288437	G	NA	NA	NA	0.1616	NA	NA	NA	0.2	0.007812	0.005882	NA	0.005208	NA	0.02885	NA	NA
rs115968373	A	NA	NA	NA	0.0101	NA	NA	NA	0.005882	0	0	NA	0	NA	0	NA	NA
rs140715097	T	NA	NA	NA	0	NA	NA	NA	0	0	0	NA	0	NA	0	NA	NA
rs376897988	T	NA	NA	NA	0	NA	NA	NA	0	0	0	NA	0	NA	0	NA	NA
rs574759417	A	NA	NA	NA	0	NA	NA	NA	0	0	0	NA	0	NA	0	NA	NA
rs528568210	A	NA	NA	NA	0	NA	NA	NA	0	0	0	NA	0	NA	0	NA	NA
rs540209049	C	NA	NA	NA	0	NA	NA	NA	0.01176	0	0	NA	0	NA	0	NA	NA
rs191595416	A	NA	NA	NA	0	NA	NA	NA	0	0	0	NA	0	NA	0	NA	NA
rs66492316	C	NA	NA	NA	0.2879	NA	NA	NA	0.2176	0.2812	0.1588	NA	0.2292	NA	0.3269	NA	NA
rs8126497	A	NA	NA	NA	0	NA	NA	NA	0.01765	0.1641	0.2294	NA	0.1354	NA	0.1827	NA	NA
rs531884709	A	NA	NA	NA	0	NA	NA	NA	0	0	0	NA	0.005208	NA	0	NA	NA
rs547558669	G	NA	NA	NA	0	NA	NA	NA	0	0	0	NA	0	NA	0	NA	NA
rs55704664	T	NA	NA	NA	0	NA	NA	NA	0.01765	0.1719	0.2294	NA	0.1354	NA	0.1875	NA	NA
rs546388820	T	NA	NA	NA	0	NA	NA	NA	0	0	0	NA	0	NA	0	NA	NA
rs142103907	G	NA	NA	NA	0	NA	NA	NA	0	0	0	NA	0	NA	0	NA	NA
rs117656646	C	NA	NA	NA	0.005051	NA	NA	NA	0	0.01562	0	NA	0	NA	0.004808	NA	NA
rs139258152	T	NA	NA	NA	0	NA	NA	NA	0	0	0	NA	0	NA	0	NA	NA
rs150382508	C	NA	NA	NA	0	NA	NA	NA	0.03529	0	0	NA	0	NA	0.004808	NA	NA
rs544308006	T	NA	NA	NA	0	NA	NA	NA	0	0	0	NA	0	NA	0	NA	NA
rs551100726	T	NA	NA	NA	0	NA	NA	NA	0	0	0	NA	0	NA	0	NA	NA
rs527435310	A	NA	NA	NA	0	NA	NA	NA	0	0	0	NA	0	NA	0	NA	NA
rs552725417	A	NA	NA	NA	0	NA	NA	NA	0	0	0	NA	0	NA	0	NA	NA
rs549038772	T	NA	NA	NA	0	NA	NA	NA	0	0	0	NA	0	NA	0	NA	NA
rs562574628	G	NA	NA	NA	0	NA	NA	NA	0	0	0	NA	0	NA	0	NA	NA
rs8129713	C	0.381	0.1667	0.3214	0.0303	0.09091	0.1667	0.1207	0.02941	0.1719	0.2294	0.165	0.1458	0.15	0.1875	0.08333	0.1667

SNP	Allele	Lamas	Lambayeque	Lima	LWK	Matses	Moche	Moquegua	MSL	MXL	PEL	Pelotas	PJL	Puno	PUR	Qeros	Quechuas
rs183385542	C	NA	NA	NA	0	NA	NA	NA	0	0	0	NA	0	NA	0	NA	NA
rs553596811	G	NA	NA	NA	0	NA	NA	NA	0	0	0	NA	0	NA	0	NA	NA
rs150473664	C	NA	NA	NA	0	NA	NA	NA	0	0	0	NA	0	NA	0	NA	NA
rs146120690	A	NA	NA	NA	0	NA	NA	NA	0	0	0.005882	NA	0	NA	0	NA	NA
rs527261679	A	NA	NA	NA	0	NA	NA	NA	0	0	0	NA	0	NA	0	NA	NA
rs570544092	T	NA	NA	NA	0	NA	NA	NA	0	0	0	NA	0	NA	0	NA	NA
rs392370	C	NA	NA	NA	0.3485	NA	NA	NA	0.3765	0.2109	0.2471	NA	0.2917	NA	0.3462	NA	NA
rs150875127	C	NA	NA	NA	0.6212	NA	NA	NA	0.4176	0.4219	0.2706	NA	0.6927	NA	0.6683	NA	NA
rs536039173	T	NA	NA	NA	0	NA	NA	NA	0	0	0	NA	0.01562	NA	0	NA	NA
rs192019778	A	NA	NA	NA	0	NA	NA	NA	0	0	0	NA	0	NA	0	NA	NA
rs537728755	A	NA	NA	NA	0.005051	NA	NA	NA	0.01176	0	0	NA	0	NA	0	NA	NA
rs55964536	T	NA	NA	NA	0.09091	NA	NA	NA	0.05294	0.2891	0.2059	NA	0.3958	NA	0.3413	NA	NA
rs373311004	C	NA	NA	NA	0	NA	NA	NA	0	0	0	NA	0	NA	0	NA	NA
rs575968857	T	NA	NA	NA	0	NA	NA	NA	0	0	0	NA	0	NA	0	NA	NA
rs548402221	G	NA	NA	NA	0	NA	NA	NA	0	0	0	NA	0.005208	NA	0.009615	NA	NA
rs140037718	C	NA	NA	NA	0.0101	NA	NA	NA	0	0	0	NA	0	NA	0	NA	NA
rs75373173	C	0.02381	0	0.01786	0	0	0	0	0	0.03125	0.01176	0.07307	0.04688	0	0.05769	0	0.02083
rs568619174	C	NA	NA	NA	0	NA	NA	NA	0	0	0	NA	0	NA	0	NA	NA
rs187175193	T	NA	NA	NA	0	NA	NA	NA	0	0	0	NA	0	NA	0	NA	NA
rs193122175	T	NA	NA	NA	0	NA	NA	NA	0	0.007812	0	NA	0	NA	0	NA	NA
rs117898838	A	NA	NA	NA	0.0303	NA	NA	NA	0.02353	0	0	NA	0	NA	0.004808	NA	NA
rs537989379	A	NA	NA	NA	0	NA	NA	NA	0.005882	0	0	NA	0	NA	0	NA	NA
rs570302987	A	NA	NA	NA	0	NA	NA	NA	0	0	0	NA	0	NA	0	NA	NA
rs190970011	T	NA	NA	NA	0	NA	NA	NA	0	0	0	NA	0	NA	0	NA	NA
rs556994660	C	NA	NA	NA	0	NA	NA	NA	0	0	0	NA	0	NA	0	NA	NA
rs527380714	C	NA	NA	NA	0	NA	NA	NA	0	0	0	NA	0	NA	0	NA	NA
rs536026892	T	NA	NA	NA	0	NA	NA	NA	0	0	0	NA	0	NA	0	NA	NA
rs535103772	T	NA	NA	NA	0	NA	NA	NA	0	0	0	NA	0	NA	0	NA	NA
rs191587330	C	NA	NA	NA	0	NA	NA	NA	0	0	0	NA	0	NA	0	NA	NA
rs553617111	T	NA	NA	NA	0	NA	NA	NA	0.01765	0	0	NA	0	NA	0	NA	NA
rs182838808	A	NA	NA	NA	0	NA	NA	NA	0	0.007812	0	NA	0	NA	0	NA	NA
rs78503214	T	NA	NA	NA	0.02525	NA	NA	NA	0.04118	0.007812	0	NA	0	NA	0	NA	NA
rs142425263	A	NA	NA	NA	0	NA	NA	NA	0	0	0	NA	0	NA	0	NA	NA
rs573213706	A	NA	NA	NA	0	NA	NA	NA	0	0	0	NA	0	NA	0	NA	NA
rs73357663	C	NA	NA	NA	0.0202	NA	NA	NA	0.04118	0	0	NA	0	NA	0.004808	NA	NA
rs563670115	C	NA	NA	NA	0	NA	NA	NA	0	0	0	NA	0	NA	0	NA	NA
rs111220509	G	NA	NA	NA	0.5354	NA	NA	NA	0.4059	0.4688	0.3059	NA	0.6875	NA	0.6538	NA	NA
rs186605876	T	NA	NA	NA	0.0101	NA	NA	NA	0	0	0	NA	0	NA	0	NA	NA
rs373611430	G	NA	NA	NA	0	NA	NA	NA	0	0	0	NA	0	NA	0	NA	NA
rs2094881	C	0.09524	0.2083	0.3036	0.5455	0.04545	0.1167	0.2931	0.4059	0.4375	0.2824	0.6904	0.6875	0.15	0.6538	0	0.1875
rs73372166	A	NA	NA	NA	0.1717	NA	NA	NA	0.3353	0.125	0.05294	NA	0.07292	NA	0.1442	NA	NA
rs76135088	G	0	0.04167	0.01786	0.1061	0	0	0	0.07647	0.007812	0.005882	0.01312	0	0	0.01442	0	0
rs548979551	G	NA	NA	NA	0	NA	NA	NA	0	0	0	NA	0	NA	0	NA	NA
rs540046047	C	NA	NA	NA	0	NA	NA	NA	0	0	0	NA	0	NA	0	NA	NA
rs2410429	A	NA	NA	NA	0.3283	NA	NA	NA	0.2765	0.4141	0.6882	NA	0.3281	NA	0.4183	NA	NA

SNP	Allele	Lamas	Lambayeque	Lima	LWK	Matses	Moche	Moquegua	MSL	MXL	PEL	Pelotas	PJL	Puno	PUR	Qeros	Quechuas
rs532950488	A	NA	NA	NA	0	NA	NA	NA	0	0	0	NA	0	NA	0	NA	NA
rs535531364	C	NA	NA	NA	0	NA	NA	NA	0.01176	0	0	NA	0	NA	0	NA	NA
rs550091217	T	NA	NA	NA	0.005051	NA	NA	NA	0	0	0	NA	0	NA	0	NA	NA
rs62217527	T	NA	NA	NA	0.0202	NA	NA	NA	0.01176	0.09375	0.02353	NA	0.03646	NA	0.1058	NA	NA
rs80275470	A	NA	NA	NA	0	NA	NA	NA	0	0	0.005882	NA	0	NA	0.009615	NA	NA
rs545165511	A	NA	NA	NA	0	NA	NA	NA	0.005882	0	0	NA	0	NA	0	NA	NA
rs144318842	A	NA	NA	NA	0.02525	NA	NA	NA	0	0	0	NA	0	NA	0	NA	NA
rs200395836	T	NA	NA	NA	0	NA	NA	NA	0	0.007812	0	NA	0	NA	0	NA	NA
rs548552862	C	NA	NA	NA	0	NA	NA	NA	0	0	0	NA	0	NA	0	NA	NA
rs577632959	A	NA	NA	NA	0	NA	NA	NA	0	0	0	NA	0	NA	0	NA	NA
rs567258163	A	NA	NA	NA	0	NA	NA	NA	0	0	0	NA	0.005208	NA	0	NA	NA
rs561789442	A	NA	NA	NA	0	NA	NA	NA	0	0	0	NA	0	NA	0	NA	NA
rs538588854	A	NA	NA	NA	0	NA	NA	NA	0	0	0	NA	0	NA	0	NA	NA
rs115429336	A	NA	NA	NA	0	NA	NA	NA	0.01765	0	0	NA	0	NA	0	NA	NA
rs537412369	T	NA	NA	NA	0	NA	NA	NA	0	0	0	NA	0	NA	0	NA	NA
rs371531071	T	NA	NA	NA	0	NA	NA	NA	0	0	0	NA	0	NA	0	NA	NA
rs183141812	A	NA	NA	NA	0	NA	NA	NA	0	0	0	NA	0	NA	0	NA	NA
rs189546747	T	NA	NA	NA	0	NA	NA	NA	0	0	0	NA	0	NA	0	NA	NA
rs3787950	C	0	0.08333	0.05357	0.2323	0	0.01724	0	0.2176	0.01562	0.01765	0.1001	0.1927	0.01282	0.1394	0	0
rs549067055	A	NA	NA	NA	0	NA	NA	NA	0	0	0	NA	0	NA	0	NA	NA
rs576955441	A	NA	NA	NA	0	NA	NA	NA	0	0	0	NA	0	NA	0	NA	NA
rs8134203	T	NA	NA	NA	0.5505	NA	NA	NA	0.4412	0.4688	0.3059	NA	0.6823	NA	0.6587	NA	NA
rs532216261	C	NA	NA	NA	0	NA	NA	NA	0	0	0	NA	0	NA	0	NA	NA
rs557766326	T	NA	NA	NA	0	NA	NA	NA	0	0	0.005882	NA	0	NA	0	NA	NA
rs546531769	G	NA	NA	NA	0	NA	NA	NA	0	0	0	NA	0	NA	0	NA	NA
rs571993620	A	NA	NA	NA	0	NA	NA	NA	0	0	0	NA	0	NA	0	NA	NA
rs374261644	C	NA	NA	NA	0	NA	NA	NA	0	0	0	NA	0	NA	0	NA	NA
rs564210410	A	NA	NA	NA	0.005051	NA	NA	NA	0.01176	0	0	NA	0	NA	0	NA	NA
rs562387776	C	NA	NA	NA	0	NA	NA	NA	0	0	0	NA	0	NA	0	NA	NA
rs562587205	T	NA	NA	NA	0	NA	NA	NA	0	0	0	NA	0	NA	0	NA	NA
rs542538936	T	NA	NA	NA	0	NA	NA	NA	0	0	0	NA	0	NA	0	NA	NA
rs143818732	C	NA	NA	NA	0.005051	NA	NA	NA	0	0	0	NA	0.01042	NA	0.009615	NA	NA
rs183650725	A	NA	NA	NA	0	NA	NA	NA	0	0	0	NA	0	NA	0	NA	NA
rs531439352	A	NA	NA	NA	0	NA	NA	NA	0	0	0	NA	0	NA	0	NA	NA
rs531183955	T	NA	NA	NA	0	NA	NA	NA	0	0	0	NA	0	NA	0	NA	NA
rs144192191	GGTGA	NA	NA	NA	0.4394	NA	NA	NA	0.3471	0.1484	0.07647	NA	0.2969	NA	0.3029	NA	NA
rs149708827	T	NA	NA	NA	0	NA	NA	NA	0	0	0	NA	0	NA	0	NA	NA
rs552635816	G	NA	NA	NA	0.005051	NA	NA	NA	0	0	0	NA	0	NA	0	NA	NA
rs539520259	T	NA	NA	NA	0	NA	NA	NA	0	0	0	NA	0	NA	0	NA	NA
rs376235035	T	NA	NA	NA	0	NA	NA	NA	0	0	0	NA	0	NA	0	NA	NA
rs142303004	T	NA	NA	NA	0.01515	NA	NA	NA	0	0	0	NA	0	NA	0	NA	NA
rs546232086	G	NA	NA	NA	0	NA	NA	NA	0	0	0.005882	NA	0	NA	0	NA	NA
rs549832756	C	NA	NA	NA	0	NA	NA	NA	0	0	0	NA	0	NA	0	NA	NA
rs75655573	G	0	0	0	0.005051	0	0	0	0	0	0	0.002543	0	0	0	0	0
rs553167698	T	NA	NA	NA	0	NA	NA	NA	0	0	0	NA	0	NA	0	NA	NA

SNP	Allele	Lamas	Lambayeque	Lima	LWK	Matses	Moche	Moquegua	MSL	MXL	PEL	Pelotas	PJL	Puno	PUR	Qeros	Quechuas
rs190685013	A	NA	NA	NA	0	NA	NA	NA	0	0	0	NA	0	NA	0	NA	NA
rs559934921	G	NA	NA	NA	0	NA	NA	NA	0	0	0	NA	0	NA	0	NA	NA
rs190899605	C	NA	NA	NA	0	NA	NA	NA	0	0	0	NA	0	NA	0.004808	NA	NA
rs391099	C	NA	NA	NA	0.3788	NA	NA	NA	0.3765	0.2109	0.2471	NA	0.2917	NA	0.3558	NA	NA
rs372422423	A	NA	NA	NA	0	NA	NA	NA	0.01176	0	0	NA	0	NA	0.009615	NA	NA
rs77014365	A	NA	NA	NA	0	NA	NA	NA	0	0	0	NA	0	NA	0	NA	NA
rs562157895	A	NA	NA	NA	0	NA	NA	NA	0	0	0	NA	0.005208	NA	0	NA	NA
rs187290362	G	NA	NA	NA	0.0101	NA	NA	NA	0	0	0.005882	NA	0	NA	0	NA	NA
rs182543256	A	NA	NA	NA	0	NA	NA	NA	0.01765	0.007812	0	NA	0	NA	0	NA	NA
rs563186329	C	NA	NA	NA	0	NA	NA	NA	0	0	0	NA	0	NA	0	NA	NA
rs569026066	T	NA	NA	NA	0	NA	NA	NA	0	0	0	NA	0	NA	0	NA	NA
rs116511699	T	NA	NA	NA	0	NA	NA	NA	0	0	0	NA	0	NA	0	NA	NA
rs148499598	C	NA	NA	NA	0	NA	NA	NA	0	0	0	NA	0	NA	0	NA	NA
rs552256264	T	NA	NA	NA	0	NA	NA	NA	0	0	0	NA	0	NA	0	NA	NA
rs562776963	A	NA	NA	NA	0	NA	NA	NA	0	0	0	NA	0	NA	0	NA	NA
rs79243099	A	NA	NA	NA	0.0303	NA	NA	NA	0.05882	0.007812	0	NA	0	NA	0.009615	NA	NA
rs79397218	A	NA	NA	NA	0	NA	NA	NA	0	0	0	NA	0.005208	NA	0	NA	NA
rs138094318	T	NA	NA	NA	0	NA	NA	NA	0	0	0	NA	0	NA	0	NA	NA
rs567275769	T	NA	NA	NA	0	NA	NA	NA	0	0	0	NA	0	NA	0	NA	NA
rs544297320	T	NA	NA	NA	0	NA	NA	NA	0	0	0	NA	0	NA	0	NA	NA
rs561404433	G	NA	NA	NA	0	NA	NA	NA	0	0	0	NA	0	NA	0	NA	NA
rs573923452	A	NA	NA	NA	0	NA	NA	NA	0	0	0	NA	0	NA	0	NA	NA
rs539858204	A	NA	NA	NA	0	NA	NA	NA	0	0	0	NA	0	NA	0	NA	NA
rs9305745	T	NA	NA	NA	0.3838	NA	NA	NA	0.3941	0.2578	0.08235	NA	0.3125	NA	0.1923	NA	NA
rs546510868	T	NA	NA	NA	0	NA	NA	NA	0	0	0	NA	0	NA	0	NA	NA
rs183418223	A	NA	NA	NA	0.0101	NA	NA	NA	0.005882	0	0	NA	0	NA	0	NA	NA
rs573692135	T	NA	NA	NA	0	NA	NA	NA	0	0	0	NA	0	NA	0	NA	NA
rs570126729	A	NA	NA	NA	0	NA	NA	NA	0	0	0	NA	0	NA	0	NA	NA
rs542946711	G	NA	NA	NA	0	NA	NA	NA	0	0	0	NA	0.01042	NA	0	NA	NA
rs551277697	G	NA	NA	NA	0	NA	NA	NA	0	0	0	NA	0	NA	0	NA	NA
rs73230088	A	NA	NA	NA	0.02525	NA	NA	NA	0.02941	0.09375	0.03529	NA	0.08333	NA	0.07692	NA	NA
rs548025657	A	NA	NA	NA	0	NA	NA	NA	0	0	0.005882	NA	0	NA	0	NA	NA
rs140428704	T	NA	NA	NA	0	NA	NA	NA	0	0	0	NA	0	NA	0.01442	NA	NA
rs184149889	C	NA	NA	NA	0	NA	NA	NA	0	0	0	NA	0	NA	0.004808	NA	NA
rs116568213	G	NA	NA	NA	0.02525	NA	NA	NA	0.01176	0	0	NA	0	NA	0.009615	NA	NA
rs187742976	A	NA	NA	NA	0	NA	NA	NA	0	0	0	NA	0	NA	0	NA	NA
rs555769183	C	NA	NA	NA	0	NA	NA	NA	0	0	0.005882	NA	0	NA	0	NA	NA
rs12627374	T	0	0	0	0.01515	0	0	0	0	0	0	0.002545	0.1302	0	0	0	0
rs565973484	A	NA	NA	NA	0	NA	NA	NA	0	0	0	NA	0	NA	0.004808	NA	NA
rs547257017	C	NA	NA	NA	0	NA	NA	NA	0.005882	0	0	NA	0	NA	0	NA	NA
rs568737536	A	NA	NA	NA	0	NA	NA	NA	0	0	0	NA	0.005208	NA	0	NA	NA
rs541239376	T	NA	NA	NA	0	NA	NA	NA	0	0	0	NA	0	NA	0	NA	NA
rs557500448	A	NA	NA	NA	0	NA	NA	NA	0	0	0	NA	0	NA	0	NA	NA
rs185166990	A	NA	NA	NA	0	NA	NA	NA	0	0	0	NA	0	NA	0	NA	NA
rs541215881	A	NA	NA	NA	0	NA	NA	NA	0	0	0	NA	0	NA	0	NA	NA

SNP	Allele	Lamas	Lambayeque	Lima	LWK	Matses	Moche	Moquegua	MSL	MXL	PEL	Pelotas	PJL	Puno	PUR	Qeros	Quechuas
rs138995130	A	NA	NA	NA	0	NA	NA	NA	0	0	0	NA	0	NA	0	NA	NA
rs462448	T	NA	NA	NA	0.005051	NA	NA	NA	0	0.2344	0.4353	NA	0.05729	NA	0.1058	NA	NA
rs569303172	T	NA	NA	NA	0	NA	NA	NA	0	0	0	NA	0	NA	0	NA	NA
rs564691729	C	NA	NA	NA	0	NA	NA	NA	0	0	0	NA	0	NA	0	NA	NA
rs187452528	C	NA	NA	NA	0.005051	NA	NA	NA	0	0	0	NA	0	NA	0	NA	NA
rs4818242	T	NA	NA	NA	0	NA	NA	NA	0	0.03125	0.04706	NA	0	NA	0.01923	NA	NA
rs183016576	T	NA	NA	NA	0	NA	NA	NA	0	0	0	NA	0	NA	0	NA	NA
rs539934676	A	NA	NA	NA	0	NA	NA	NA	0	0	0	NA	0	NA	0	NA	NA
rs184876485	G	NA	NA	NA	0.005051	NA	NA	NA	0	0	0	NA	0	NA	0	NA	NA
rs549676267	T	NA	NA	NA	0	NA	NA	NA	0	0	0	NA	0	NA	0	NA	NA
rs150633108	T	NA	NA	NA	0	NA	NA	NA	0	0	0	NA	0.02083	NA	0.009615	NA	NA
rs185103560	A	NA	NA	NA	0	NA	NA	NA	0	0	0	NA	0	NA	0	NA	NA
rs532655542	G	NA	NA	NA	0	NA	NA	NA	0	0	0	NA	0	NA	0	NA	NA
rs180831387	A	NA	NA	NA	0	NA	NA	NA	0	0	0	NA	0	NA	0	NA	NA
rs569577322	T	NA	NA	NA	0	NA	NA	NA	0	0	0	NA	0	NA	0	NA	NA
rs144154504	C	NA	NA	NA	0	NA	NA	NA	0	0	0	NA	0	NA	0	NA	NA
rs556937466	T	NA	NA	NA	0	NA	NA	NA	0	0	0	NA	0	NA	0	NA	NA
rs115129572	T	0	0	0.01786	0.01515	0	0	0	0.005882	0	0.005882	0.004695	0	0	0	0	0
rs578015897	T	NA	NA	NA	0	NA	NA	NA	0	0	0	NA	0	NA	0	NA	NA
rs2298661	A	NA	NA	NA	0.2222	NA	NA	NA	0.2294	0.3125	0.2588	NA	0.25	NA	0.1538	NA	NA
rs557088853	T	NA	NA	NA	0	NA	NA	NA	0	0	0	NA	0	NA	0	NA	NA
rs141301979	C	NA	NA	NA	0	NA	NA	NA	0.005882	0	0	NA	0	NA	0.004808	NA	NA
rs8131649	T	0.8095	0.75	0.6607	0.4091	0.7727	0.8333	0.7069	0.4706	0.5312	0.6882	0.2962	0.3177	0.825	0.3173	1	0.7917
rs539860607	G	NA	NA	NA	0	NA	NA	NA	0.005882	0	0	NA	0	NA	0	NA	NA
rs4816720	C	NA	NA	NA	0.1162	NA	NA	NA	0.2294	0.2266	0.4471	NA	0.03646	NA	0.1635	NA	NA
rs11702475	T	NA	NA	NA	0.1212	NA	NA	NA	0.07059	0.3438	0.2294	NA	0.3958	NA	0.3173	NA	NA
rs150205743	A	NA	NA	NA	0.0202	NA	NA	NA	0.01765	0	0	NA	0	NA	0	NA	NA
rs8127674	G	0.119	0.08333	0.1607	0.3131	0.2727	0.15	0.1429	0.2235	0.2578	0.1412	0.3617	0.2031	0.075	0.3029	0	0.125
rs190134102	A	NA	NA	NA	0	NA	NA	NA	0.005882	0	0	NA	0	NA	0	NA	NA
rs141324737	T	NA	NA	NA	0.005051	NA	NA	NA	0	0	0	NA	0	NA	0	NA	NA
rs560898303	CA	NA	NA	NA	0.005051	NA	NA	NA	0	0	0	NA	0.005208	NA	0	NA	NA
rs552105586	T	NA	NA	NA	0	NA	NA	NA	0	0	0	NA	0	NA	0	NA	NA
rs146052428	T	NA	NA	NA	0.0202	NA	NA	NA	0.01176	0	0	NA	0	NA	0	NA	NA
rs188241223	T	NA	NA	NA	0	NA	NA	NA	0	0	0	NA	0	NA	0	NA	NA
rs547639377	G	NA	NA	NA	0	NA	NA	NA	0	0	0	NA	0	NA	0	NA	NA
rs420737	G	NA	NA	NA	0.303	NA	NA	NA	0.2588	0.2422	0.4118	NA	0.1667	NA	0.125	NA	NA
rs534978728	G	NA	NA	NA	0	NA	NA	NA	0	0	0.005882	NA	0	NA	0	NA	NA
rs369948528	T	NA	NA	NA	0	NA	NA	NA	0	0	0	NA	0.005208	NA	0	NA	NA
rs533867263	A	NA	NA	NA	0.0101	NA	NA	NA	0.005882	0	0	NA	0	NA	0	NA	NA
rs555525436	G	NA	NA	NA	0	NA	NA	NA	0	0	0	NA	0	NA	0	NA	NA
rs531121505	T	NA	NA	NA	0	NA	NA	NA	0	0	0	NA	0	NA	0	NA	NA
rs376143876	A	NA	NA	NA	0	NA	NA	NA	0	0	0	NA	0.02083	NA	0	NA	NA
rs554986236	T	NA	NA	NA	0	NA	NA	NA	0	0	0	NA	0	NA	0	NA	NA
rs66575656	T	NA	NA	NA	0.3232	NA	NA	NA	0.1765	0.1328	0.07059	NA	0.2865	NA	0.2837	NA	NA
rs542746297	T	NA	NA	NA	0	NA	NA	NA	0	0	0	NA	0	NA	0	NA	NA

SNP	Allele	Lamas	Lambayeque	Lima	LWK	Matses	Moche	Moquegua	MSL	MXL	PEL	Pelotas	PJL	Puno	PUR	Qeros	Quechuas
rs567175997	G	NA	NA	NA	0	NA	NA	NA	0	0	0	NA	0	NA	0	NA	NA
rs578020496	C	NA	NA	NA	0	NA	NA	NA	0	0	0	NA	0	NA	0	NA	NA
rs543607180	C	NA	NA	NA	0	NA	NA	NA	0	0	0	NA	0	NA	0	NA	NA
rs115849825	A	NA	NA	NA	0.0101	NA	NA	NA	0	0	0	NA	0	NA	0.004808	NA	NA
rs542333608	T	NA	NA	NA	0.005051	NA	NA	NA	0	0	0	NA	0	NA	0	NA	NA
rs34983238	C	NA	NA	NA	0.04545	NA	NA	NA	0.04706	0.02344	0.01176	NA	0.125	NA	0.03846	NA	NA
rs372733383	T	NA	NA	NA	0	NA	NA	NA	0	0	0	NA	0	NA	0	NA	NA
rs555134126	T	NA	NA	NA	0	NA	NA	NA	0	0	0	NA	0.005208	NA	0	NA	NA
rs533043037	A	NA	NA	NA	0	NA	NA	NA	0	0	0	NA	0	NA	0	NA	NA
rs73372168	A	NA	NA	NA	0	NA	NA	NA	0.02353	0	0	NA	0	NA	0	NA	NA
rs150715708	T	NA	NA	NA	0	NA	NA	NA	0	0	0	NA	0	NA	0	NA	NA
rs145024812	A	NA	NA	NA	0	NA	NA	NA	0.03529	0	0	NA	0	NA	0.004808	NA	NA
rs557592331	C	NA	NA	NA	0	NA	NA	NA	0	0	0	NA	0.01042	NA	0	NA	NA
rs567380110	T	NA	NA	NA	0	NA	NA	NA	0	0	0	NA	0	NA	0	NA	NA
rs560408119	A	NA	NA	NA	0	NA	NA	NA	0	0	0.005882	NA	0	NA	0	NA	NA
rs117432315	A	NA	NA	NA	0	NA	NA	NA	0	0	0	NA	0	NA	0	NA	NA
rs370654859	C	NA	NA	NA	0.005051	NA	NA	NA	0.03529	0.007812	0	NA	0	NA	0.004808	NA	NA
rs139181746	C	NA	NA	NA	0	NA	NA	NA	0	0	0	NA	0	NA	0	NA	NA
rs554175681	G	NA	NA	NA	0.005051	NA	NA	NA	0	0	0	NA	0	NA	0	NA	NA
rs28548447	A	NA	NA	NA	0.3283	NA	NA	NA	0.1882	0.1875	0.08235	NA	0.2969	NA	0.3558	NA	NA
rs191526916	T	NA	NA	NA	0	NA	NA	NA	0	0	0	NA	0	NA	0	NA	NA
rs192959767	T	NA	NA	NA	0	NA	NA	NA	0	0	0	NA	0	NA	0	NA	NA
rs534192391	T	NA	NA	NA	0	NA	NA	NA	0	0	0	NA	0	NA	0	NA	NA
rs571211518	C	NA	NA	NA	0	NA	NA	NA	0	0	0	NA	0	NA	0	NA	NA
rs562033822	A	NA	NA	NA	0	NA	NA	NA	0	0	0	NA	0	NA	0	NA	NA
rs142194573	AG	NA	NA	NA	0	NA	NA	NA	0	0	0	NA	0	NA	0	NA	NA
rs149695119	T	NA	NA	NA	0.09091	NA	NA	NA	0.1941	0.007812	0.005882	NA	0	NA	0.05769	NA	NA
rs143270468	A	NA	NA	NA	0	NA	NA	NA	0	0.007812	0.005882	NA	0	NA	0.01442	NA	NA
rs534423780	A	NA	NA	NA	0	NA	NA	NA	0	0	0	NA	0	NA	0.009615	NA	NA
rs535545388	G	NA	NA	NA	0	NA	NA	NA	0	0	0	NA	0.005208	NA	0	NA	NA
rs528206157	T	NA	NA	NA	0	NA	NA	NA	0	0	0	NA	0	NA	0	NA	NA
rs112985398	C	NA	NA	NA	0.05051	NA	NA	NA	0.02941	0	0	NA	0	NA	0	NA	NA
rs77511690	A	NA	NA	NA	0.02525	NA	NA	NA	0.01765	0	0	NA	0	NA	0.004808	NA	NA
rs538161583	A	NA	NA	NA	0	NA	NA	NA	0	0	0	NA	0	NA	0	NA	NA
rs552591104	C	NA	NA	NA	0	NA	NA	NA	0	0	0	NA	0	NA	0	NA	NA
rs557224603	A	NA	NA	NA	0	NA	NA	NA	0	0	0	NA	0	NA	0	NA	NA
rs545825254	G	NA	NA	NA	0	NA	NA	NA	0.005882	0	0	NA	0	NA	0	NA	NA
rs185312677	T	NA	NA	NA	0	NA	NA	NA	0	0	0	NA	0	NA	0	NA	NA
rs423596	T	NA	NA	NA	0.01515	NA	NA	NA	0	0.007812	0.01176	NA	0.1406	NA	0.03846	NA	NA
rs541726793	A	NA	NA	NA	0	NA	NA	NA	0	0	0.005882	NA	0	NA	0	NA	NA
rs62217529	T	NA	NA	NA	0	NA	NA	NA	0	0	0.005882	NA	0	NA	0	NA	NA
rs528452414	C	NA	NA	NA	0	NA	NA	NA	0	0	0	NA	0	NA	0	NA	NA
rs143167369	T	NA	NA	NA	0	NA	NA	NA	0	0	0	NA	0	NA	0	NA	NA
rs138164084	T	NA	NA	NA	0	NA	NA	NA	0	0	0	NA	0	NA	0	NA	NA
rs62217531	T	0.1905	0.125	0.1964	0.2273	0.2273	0.1333	0.1552	0.2824	0.3359	0.2294	0.4104	0.3385	0.15	0.3317	0	0.1042

SNP	Allele	Lamas	Lambayeque	Lima	LWK	Matses	Moche	Moquegua	MSL	MXL	PEL	Pelotas	PJL	Puno	PUR	Qeros	Quechuas
rs528205318	G	NA	NA	NA	0	NA	NA	NA	0	0	0	NA	0	NA	0	NA	NA
rs554097496	A	NA	NA	NA	0	NA	NA	NA	0	0	0	NA	0	NA	0	NA	NA
rs61728255	A	NA	NA	NA	0.005051	NA	NA	NA	0	0.2344	0.4471	NA	0.05729	NA	0.1106	NA	NA
rs538022064	C	NA	NA	NA	0	NA	NA	NA	0	0	0	NA	0	NA	0.004808	NA	NA
rs9980225	C	NA	NA	NA	0.005051	NA	NA	NA	0	0.1406	0.05294	NA	0.09896	NA	0.06731	NA	NA
rs542530281	C	NA	NA	NA	0	NA	NA	NA	0	0	0	NA	0	NA	0	NA	NA
rs542407537	A	NA	NA	NA	0	NA	NA	NA	0	0	0	NA	0	NA	0	NA	NA
rs548552823	A	NA	NA	NA	0	NA	NA	NA	0	0	0	NA	0	NA	0	NA	NA
rs565903681	C	NA	NA	NA	0	NA	NA	NA	0	0	0	NA	0	NA	0	NA	NA
rs201109436	G	NA	NA	NA	0.005051	NA	NA	NA	0	0	0	NA	0	NA	0	NA	NA
rs577393745	T	NA	NA	NA	0	NA	NA	NA	0	0	0	NA	0	NA	0	NA	NA
rs185086499	T	NA	NA	NA	0	NA	NA	NA	0	0	0	NA	0	NA	0	NA	NA
rs141230106	T	NA	NA	NA	0	NA	NA	NA	0	0	0	NA	0	NA	0	NA	NA
rs531548362	C	NA	NA	NA	0	NA	NA	NA	0.005882	0	0	NA	0	NA	0	NA	NA
rs150445636	A	NA	NA	NA	0	NA	NA	NA	0.01176	0	0	NA	0	NA	0	NA	NA
rs139015396	T	NA	NA	NA	0	NA	NA	NA	0.03529	0	0	NA	0	NA	0.004808	NA	NA
rs183722985	A	NA	NA	NA	0	NA	NA	NA	0.005882	0	0	NA	0	NA	0	NA	NA
rs543734531	A	NA	NA	NA	0	NA	NA	NA	0	0	0	NA	0	NA	0	NA	NA
rs553875292	T	NA	NA	NA	0	NA	NA	NA	0	0	0	NA	0	NA	0	NA	NA
rs55760462	C	0.1429	0.125	0.1071	0.2525	0.2727	0.15	0.08621	0.2294	0.2266	0.1118	0.2385	0.09375	0.05	0.25	0	0.1458
rs61735790	C	NA	NA	NA	0.0202	NA	NA	NA	0	0	0	NA	0	NA	0.01442	NA	NA
rs566918037	G	NA	NA	NA	0	NA	NA	NA	0	0	0	NA	0	NA	0	NA	NA
rs555820997	G	NA	NA	NA	0.0101	NA	NA	NA	0.005882	0	0	NA	0	NA	0	NA	NA
rs573493693	A	NA	NA	NA	0	NA	NA	NA	0	0	0	NA	0	NA	0	NA	NA
rs914184	A	NA	NA	NA	0	NA	NA	NA	0	0	0.005882	NA	0	NA	0	NA	NA
rs539019494	C	NA	NA	NA	0	NA	NA	NA	0	0	0.005882	NA	0	NA	0	NA	NA
rs544036587	T	NA	NA	NA	0	NA	NA	NA	0.01765	0	0	NA	0	NA	0	NA	NA
rs565896303	A	NA	NA	NA	0	NA	NA	NA	0	0	0	NA	0	NA	0	NA	NA
rs192793047	C	NA	NA	NA	0	NA	NA	NA	0	0	0	NA	0	NA	0	NA	NA
rs546790067	C	NA	NA	NA	0	NA	NA	NA	0	0	0	NA	0	NA	0	NA	NA
rs553672299	A	NA	NA	NA	0	NA	NA	NA	0	0	0.005882	NA	0	NA	0	NA	NA
rs570629130	A	NA	NA	NA	0	NA	NA	NA	0	0	0	NA	0	NA	0	NA	NA
rs117562633	C	NA	NA	NA	0	NA	NA	NA	0	0	0	NA	0	NA	0	NA	NA
rs395584	C	0.3095	0.4167	0.3036	0.303	0.5455	0.5833	0.3448	0.2588	0.2422	0.4118	0.1024	0.1667	0.4625	0.125	0.5833	0.4583
rs143597099	A	NA	NA	NA	NA	NA	NA	NA	NA	NA	NA	NA	NA	NA	NA	NA	NA
rs543168691	C	NA	NA	NA	0	NA	NA	NA	0	0	0	NA	0	NA	0	NA	NA
rs530689404	T	NA	NA	NA	0	NA	NA	NA	0	0	0	NA	0	NA	0	NA	NA
rs76833541	A	NA	NA	NA	0	NA	NA	NA	0	0.03125	0.005882	NA	0.04688	NA	0.05769	NA	NA
rs150907799	T	NA	NA	NA	0.0303	NA	NA	NA	0.02353	0	0	NA	0	NA	0	NA	NA
rs554550449	A	NA	NA	NA	0	NA	NA	NA	0	0	0	NA	0	NA	0	NA	NA
rs566085188	A	NA	NA	NA	0.005051	NA	NA	NA	0	0	0	NA	0	NA	0	NA	NA
rs553431242	G	NA	NA	NA	0	NA	NA	NA	0.005882	0	0	NA	0	NA	0	NA	NA
rs551442787	A	NA	NA	NA	0	NA	NA	NA	0	0	0	NA	0	NA	0	NA	NA
rs551677463	C	NA	NA	NA	0	NA	NA	NA	0	0	0	NA	0	NA	0	NA	NA
rs549801931	T	NA	NA	NA	0	NA	NA	NA	0	0	0	NA	0	NA	0	NA	NA

SNP	Allele	Lamas	Lambayeque	Lima	LWK	Matses	Moche	Moquegua	MSL	MXL	PEL	Pelotas	PJL	Puno	PUR	Qeros	Quechuas
rs141864728	C	NA	NA	NA	0.0101	NA	NA	NA	0	0	0	NA	0	NA	0	NA	NA
rs373480225	T	NA	NA	NA	0	NA	NA	NA	0	0	0	NA	0.02083	NA	0	NA	NA
rs73903405	A	NA	NA	NA	0.03535	NA	NA	NA	0.06471	0	0	NA	0	NA	0.02404	NA	NA
rs567039032	A	NA	NA	NA	0	NA	NA	NA	0	0	0	NA	0	NA	0	NA	NA
rs2070792	A	0	0.1667	0.1786	0.4949	0	0.06667	0.1552	0.4765	0.1953	0.1059	0.3573	0.2865	0.025	0.4087	0	0.02083
rs552550910	A	NA	NA	NA	0	NA	NA	NA	0	0	0	NA	0	NA	0	NA	NA
rs535778149	A	NA	NA	NA	0	NA	NA	NA	0	0	0	NA	0	NA	0	NA	NA
rs201984814	A	NA	NA	NA	0	NA	NA	NA	0	0	0	NA	0	NA	0	NA	NA
rs544847277	A	NA	NA	NA	0	NA	NA	NA	0.005882	0	0	NA	0	NA	0	NA	NA
rs2070793	G	NA	NA	NA	0.4949	NA	NA	NA	0.4765	0.1953	0.1059	NA	0.2865	NA	0.4087	NA	NA
rs191345780	A	NA	NA	NA	0	NA	NA	NA	0	0	0	NA	0	NA	0	NA	NA
rs9980693	A	NA	NA	NA	0.01515	NA	NA	NA	0.02941	0.08594	0.03529	NA	0.07812	NA	0.07692	NA	NA
rs146865760	T	NA	NA	NA	0	NA	NA	NA	0	0	0	NA	0	NA	0	NA	NA
rs527898967	G	NA	NA	NA	0	NA	NA	NA	0	0	0	NA	0	NA	0	NA	NA
rs150389990	T	NA	NA	NA	NA	NA	NA	NA	NA	NA	NA	NA	NA	NA	NA	NA	NA
rs558492934	T	NA	NA	NA	0	NA	NA	NA	0	0	0	NA	0	NA	0	NA	NA
rs533513206	C	NA	NA	NA	0	NA	NA	NA	0	0	0	NA	0	NA	0	NA	NA
rs183758144	A	NA	NA	NA	0	NA	NA	NA	0	0	0	NA	0	NA	0	NA	NA
rs377403765	A	NA	NA	NA	0.005051	NA	NA	NA	0	0	0	NA	0	NA	0.004808	NA	NA
rs543740889	C	NA	NA	NA	0	NA	NA	NA	0	0	0	NA	0	NA	0	NA	NA
rs563188125	A	NA	NA	NA	0	NA	NA	NA	0	0	0	NA	0	NA	0	NA	NA
rs536677167	G	NA	NA	NA	0	NA	NA	NA	0.01765	0	0	NA	0	NA	0	NA	NA
rs541782164	T	NA	NA	NA	0	NA	NA	NA	0	0	0	NA	0.005208	NA	0	NA	NA
rs138161288	T	NA	NA	NA	0	NA	NA	NA	0	0	0	NA	0	NA	0	NA	NA
rs541499151	A	NA	NA	NA	0	NA	NA	NA	0	0	0	NA	0	NA	0	NA	NA
rs543339705	C	NA	NA	NA	0	NA	NA	NA	0	0	0	NA	0	NA	0	NA	NA
rs77599791	G	NA	NA	NA	0.005051	NA	NA	NA	0.04118	0	0	NA	0	NA	0	NA	NA
rs549745562	G	NA	NA	NA	0	NA	NA	NA	0	0	0	NA	0	NA	0	NA	NA
rs75317729	C	NA	NA	NA	0.005051	NA	NA	NA	0.005882	0	0	NA	0	NA	0	NA	NA
rs139661079	A	NA	NA	NA	0.0101	NA	NA	NA	0.02353	0	0	NA	0	NA	0	NA	NA
rs572699613	A	NA	NA	NA	0	NA	NA	NA	0	0	0	NA	0	NA	0	NA	NA
rs536023174	T	NA	NA	NA	0	NA	NA	NA	0	0	0	NA	0	NA	0	NA	NA
rs415731	A	NA	NA	NA	0.1919	NA	NA	NA	0.2471	0.3672	0.3882	0.3203	0.3073	NA	0.3221	NA	NA
rs3761374	C	0	0.08333	0	0.005051	0	0.01667	0.1852	0.005882	0.08594	0.03529	0.1313	0.08854	0.02564	0.07692	0	0
rs147233451	T	NA	NA	NA	0	NA	NA	NA	0	0	0	NA	0	NA	0	NA	NA
rs143595083	T	NA	NA	NA	0	NA	NA	NA	0.03529	0	0	NA	0	NA	0.004808	NA	NA
rs565237319	C	NA	NA	NA	0	NA	NA	NA	0	0	0	NA	0.005208	NA	0	NA	NA
rs576510757	G	NA	NA	NA	0	NA	NA	NA	0	0	0	NA	0	NA	0	NA	NA
rs535235411	A	NA	NA	NA	0	NA	NA	NA	0	0	0	NA	0	NA	0	NA	NA
rs190265904	A	NA	NA	NA	0	NA	NA	NA	0	0	0	NA	0	NA	0	NA	NA
rs149527323	A	NA	NA	NA	0	NA	NA	NA	0	0	0	NA	0	NA	0	NA	NA
rs544141276	A	NA	NA	NA	0	NA	NA	NA	0	0	0	NA	0	NA	0	NA	NA
rs186097675	T	NA	NA	NA	0	NA	NA	NA	0	0	0	NA	0	NA	0	NA	NA
rs547168890	A	NA	NA	NA	0	NA	NA	NA	0	0	0	NA	0	NA	0	NA	NA
rs138928680	GAGAA	NA	NA	NA	0	NA	NA	NA	0.005882	0.007812	0	NA	0	NA	0	NA	NA

SNP	Allele	Lamas	Lambayeque	Lima	LWK	Matses	Moche	Moquegua	MSL	MXL	PEL	Pelotas	PJL	Puno	PUR	Qeros	Quechuas
rs567676569	T	NA	NA	NA	0	NA	NA	NA	0	0	0	NA	0	NA	0	NA	NA
rs530701233	A	NA	NA	NA	0	NA	NA	NA	0	0	0	NA	0	NA	0	NA	NA
rs562332833	T	NA	NA	NA	0	NA	NA	NA	0	0	0	NA	0.005208	NA	0	NA	NA
rs531545827	T	NA	NA	NA	0	NA	NA	NA	0	0	0	NA	0	NA	0	NA	NA
rs530584676	T	NA	NA	NA	0	NA	NA	NA	0	0	0	NA	0	NA	0	NA	NA
rs185416185	T	NA	NA	NA	0	NA	NA	NA	0	0	0	NA	0.02083	NA	0	NA	NA
rs429442	T	NA	NA	NA	0.2929	NA	NA	NA	0.3294	0.2109	0.2353	NA	0.2865	NA	0.3029	NA	NA
rs79065748	A	NA	NA	NA	0.0303	NA	NA	NA	0.02353	0.007812	0.005882	NA	0	NA	0.01442	NA	NA
rs575760704	C	NA	NA	NA	0	NA	NA	NA	0	0	0	NA	0.005208	NA	0	NA	NA
rs875393	A	NA	NA	NA	0	NA	NA	NA	0	0.02344	0.005882	NA	0.04688	NA	0.009615	NA	NA
rs148901354	T	NA	NA	NA	0	NA	NA	NA	0	0	0.01176	NA	0	NA	0	NA	NA
rs561279116	G	NA	NA	NA	0	NA	NA	NA	0.01176	0	0	NA	0	NA	0	NA	NA
rs138661151	A	NA	NA	NA	0.0202	NA	NA	NA	0.04118	0	0	NA	0	NA	0	NA	NA
rs9975014	G	NA	NA	NA	0.3333	NA	NA	NA	0.1941	0.2422	0.2529	NA	0.2917	NA	0.3125	NA	NA
rs573564326	A	NA	NA	NA	0.0101	NA	NA	NA	0	0	0.01176	NA	0	NA	0	NA	NA
rs144276163	G	NA	NA	NA	0.0101	NA	NA	NA	0	0	0	NA	0	NA	0	NA	NA
rs142750000	T	NA	NA	NA	0	NA	NA	NA	0	0	0	NA	0	NA	0.004808	NA	NA
rs143672898	T	NA	NA	NA	0	NA	NA	NA	0	0	0	NA	0.005208	NA	0	NA	NA
rs560182574	C	NA	NA	NA	0	NA	NA	NA	0.005882	0	0	NA	0	NA	0	NA	NA
rs534312135	T	NA	NA	NA	0	NA	NA	NA	0	0	0	NA	0	NA	0	NA	NA
rs147986574	A	NA	NA	NA	0.005051	NA	NA	NA	0	0	0	NA	0	NA	0	NA	NA
rs181874957	T	NA	NA	NA	0	NA	NA	NA	0	0	0	NA	0	NA	0	NA	NA
rs574957059	A	NA	NA	NA	0	NA	NA	NA	0	0	0	NA	0	NA	0	NA	NA
rs185018051	C	NA	NA	NA	0	NA	NA	NA	0	0	0	NA	0	NA	0	NA	NA
rs564806692	A	NA	NA	NA	0	NA	NA	NA	0	0	0	NA	0.01562	NA	0	NA	NA
rs556079659	C	NA	NA	NA	0	NA	NA	NA	0	0	0	NA	0	NA	0	NA	NA
rs79617378	C	0	0	0	0	0	0	0.01724	0	0.007812	0.005882	0.01285	0.01042	0	0.004808	0	0
rs189436980	G	NA	NA	NA	0.005051	NA	NA	NA	0.01765	0	0	NA	0	NA	0	NA	NA
rs187078345	A	NA	NA	NA	0	NA	NA	NA	0.03529	0	0	NA	0	NA	0.004808	NA	NA
rs534885992	A	NA	NA	NA	0	NA	NA	NA	0	0	0	NA	0	NA	0	NA	NA
rs187143520	C	NA	NA	NA	0	NA	NA	NA	0.01176	0	0	NA	0	NA	0	NA	NA
rs535174935	T	NA	NA	NA	0	NA	NA	NA	0	0	0	NA	0	NA	0	NA	NA
rs528681261	T	NA	NA	NA	0	NA	NA	NA	0	0	0	NA	0	NA	0	NA	NA
rs563352095	T	NA	NA	NA	0	NA	NA	NA	0	0	0	NA	0	NA	0	NA	NA
rs117688613	G	NA	NA	NA	0	NA	NA	NA	0	0	0	NA	0	NA	0	NA	NA
rs550253934	G	NA	NA	NA	0	NA	NA	NA	0	0	0	NA	0	NA	0	NA	NA
rs181162134	C	NA	NA	NA	0.005051	NA	NA	NA	0	0	0	NA	0	NA	0	NA	NA
rs192026800	A	NA	NA	NA	0	NA	NA	NA	0	0	0	NA	0	NA	0	NA	NA
rs552303675	T	NA	NA	NA	0	NA	NA	NA	0	0	0	NA	0	NA	0	NA	NA
rs147147950	A	NA	NA	NA	0	NA	NA	NA	0	0	0	NA	0	NA	0	NA	NA
rs76314085	G	NA	NA	NA	0.0202	NA	NA	NA	0	0	0	NA	0	NA	0	NA	NA
rs567504414	T	NA	NA	NA	0	NA	NA	NA	0	0	0.005882	NA	0	NA	0	NA	NA
rs563853944	C	NA	NA	NA	0	NA	NA	NA	0	0	0	NA	0	NA	0	NA	NA
rs578103765	T	NA	NA	NA	0.005051	NA	NA	NA	0	0	0	NA	0	NA	0	NA	NA
rs574238899	G	NA	NA	NA	0	NA	NA	NA	0	0	0	NA	0	NA	0	NA	NA

SNP	Allele	Lamas	Lambayeque	Lima	LWK	Matses	Moche	Moquegua	MSL	MXL	PEL	Pelotas	PJL	Puno	PUR	Qeros	Quechuas
rs2838040	G	NA	NA	NA	0.5303	NA	NA	NA	0.5588	0.2578	0.09412	NA	0.3073	NA	0.2356	NA	NA
rs537876986	A	NA	NA	NA	0	NA	NA	NA	0	0	0	NA	0	NA	0	NA	NA
rs550222907	T	NA	NA	NA	0	NA	NA	NA	0	0.007812	0	NA	0	NA	0	NA	NA
rs556260609	A	NA	NA	NA	0	NA	NA	NA	0	0	0	NA	0	NA	0	NA	NA
rs142296178	A	NA	NA	NA	0.0101	NA	NA	NA	0	0	0	NA	0	NA	0	NA	NA
rs577285910	A	NA	NA	NA	0	NA	NA	NA	0	0	0	NA	0	NA	0	NA	NA
rs566925007	A	NA	NA	NA	0	NA	NA	NA	0	0	0	NA	0	NA	0.004808	NA	NA
rs457909	G	NA	NA	NA	0	NA	NA	NA	0	0	0	NA	0	NA	0	NA	NA
rs562479511	T	NA	NA	NA	0	NA	NA	NA	0	0	0	NA	0	NA	0	NA	NA
rs2298663	T	NA	NA	NA	0.5253	NA	NA	NA	0.4	0.4375	0.2824	NA	0.6823	NA	0.649	NA	NA
rs2070787	G	NA	NA	NA	0.4343	NA	NA	NA	0.3	0.1953	0.1	NA	0.2917	NA	0.3702	NA	NA
rs573615979	C	NA	NA	NA	0	NA	NA	NA	0	0	0	NA	0	NA	0	NA	NA
rs542031508	C	NA	NA	NA	0	NA	NA	NA	0	0	0	NA	0	NA	0	NA	NA
rs555600211	A	NA	NA	NA	0	NA	NA	NA	0	0	0	NA	0	NA	0	NA	NA
rs528451616	A	NA	NA	NA	0	NA	NA	NA	0	0	0	NA	0	NA	0	NA	NA
rs378501	G	NA	NA	NA	0	NA	NA	NA	0	0	0	NA	0	NA	0	NA	NA
rs140408843	A	NA	NA	NA	0	NA	NA	NA	0	0	0	NA	0	NA	0	NA	NA
rs186280517	C	NA	NA	NA	0	NA	NA	NA	0	0	0	NA	0	NA	0	NA	NA
rs148561341	T	NA	NA	NA	0.005051	NA	NA	NA	0	0	0	NA	0	NA	0	NA	NA
rs568162250	A	NA	NA	NA	0	NA	NA	NA	0	0	0	NA	0	NA	0	NA	NA
rs561709263	C	NA	NA	NA	0	NA	NA	NA	0	0	0	NA	0	NA	0	NA	NA
rs569583276	A	NA	NA	NA	0	NA	NA	NA	0	0	0	NA	0	NA	0	NA	NA
rs572164476	A	NA	NA	NA	0	NA	NA	NA	0	0	0	NA	0	NA	0	NA	NA
rs560575827	T	NA	NA	NA	0	NA	NA	NA	0	0	0	NA	0	NA	0	NA	NA
rs111220492	G	NA	NA	NA	0.2071	NA	NA	NA	0.2176	0.3047	0.2118	NA	0.3958	NA	0.351	NA	NA
rs182231135	C	NA	NA	NA	0	NA	NA	NA	0	0	0	NA	0	NA	0	NA	NA
rs531884512	T	NA	NA	NA	0	NA	NA	NA	0	0	0	NA	0	NA	0	NA	NA
rs573986970	A	NA	NA	NA	0	NA	NA	NA	0	0	0	NA	0	NA	0	NA	NA
rs564705715	A	NA	NA	NA	0	NA	NA	NA	0	0	0	NA	0	NA	0	NA	NA
rs201661208	A	NA	NA	NA	0	NA	NA	NA	0	0	0	NA	0	NA	0	NA	NA
rs530540353	C	NA	NA	NA	0	NA	NA	NA	0	0	0	NA	0	NA	0	NA	NA
rs148155433	A	NA	NA	NA	0.005051	NA	NA	NA	0	0	0	NA	0	NA	0	NA	NA
rs558834486	G	NA	NA	NA	0	NA	NA	NA	0	0	0	NA	0	NA	0	NA	NA
rs186275240	A	NA	NA	NA	0	NA	NA	NA	0	0	0	NA	0	NA	0	NA	NA
rs531418415	A	NA	NA	NA	0	NA	NA	NA	0	0	0	NA	0	NA	0	NA	NA
rs531609155	G	NA	NA	NA	0	NA	NA	NA	0	0	0	NA	0	NA	0	NA	NA
rs376752614	A	NA	NA	NA	0	NA	NA	NA	0	0	0	NA	0	NA	0	NA	NA
rs139144487	A	NA	NA	NA	0.005051	NA	NA	NA	0	0	0	NA	0	NA	0	NA	NA
rs531754656	A	NA	NA	NA	0	NA	NA	NA	0.005882	0	0	NA	0	NA	0	NA	NA
rs554702767	A	NA	NA	NA	0	NA	NA	NA	0.005882	0	0	NA	0	NA	0	NA	NA
rs456016	T	NA	NA	NA	0.03535	NA	NA	NA	0.02353	0.2344	0.4529	NA	0.05729	NA	0.1058	NA	NA
rs144364265	T	NA	NA	NA	0	NA	NA	NA	0	0	0	NA	0	NA	0	NA	NA
rs113773731	C	NA	NA	NA	0.0101	NA	NA	NA	0.01176	0	0	NA	0	NA	0.009615	NA	NA
rs142261174	T	NA	NA	NA	0.005051	NA	NA	NA	0	0	0	NA	0	NA	0	NA	NA
rs563595464	T	NA	NA	NA	0	NA	NA	NA	0.005882	0	0	NA	0	NA	0	NA	NA

SNP	Allele	Lamas	Lambayeque	Lima	LWK	Matses	Moche	Moquegua	MSL	MXL	PEL	Pelotas	PJL	Puno	PUR	Qeros	Quechuas
rs531013422	T	NA	NA	NA	0	NA	NA	NA	0	0	0	NA	0	NA	0	NA	NA
rs569886879	G	NA	NA	NA	0	NA	NA	NA	0	0	0	NA	0	NA	0	NA	NA
rs541977145	G	NA	NA	NA	0	NA	NA	NA	0	0	0	NA	0	NA	0.004808	NA	NA
rs190906841	T	NA	NA	NA	0	NA	NA	NA	0	0	0	NA	0	NA	0	NA	NA
rs139222305	T	NA	NA	NA	0	NA	NA	NA	0	0	0	NA	0.005208	NA	0.004808	NA	NA
rs527242422	T	NA	NA	NA	0	NA	NA	NA	0	0	0	NA	0	NA	0	NA	NA
rs364289	A	NA	NA	NA	0.3939	NA	NA	NA	0.4824	0.2109	0.2471	NA	0.2865	NA	0.3365	NA	NA
rs371053759	T	NA	NA	NA	0.005051	NA	NA	NA	0.02353	0	0	NA	0	NA	0.01923	NA	NA
rs146116431	T	NA	NA	NA	0	NA	NA	NA	0	0	0	NA	0	NA	0	NA	NA
rs928871	C	NA	NA	NA	0.3485	NA	NA	NA	0.3235	0.7344	0.91765	NA	0.6823	NA	0.7404	NA	NA
rs532335955	T	NA	NA	NA	0	NA	NA	NA	0	0	0	NA	0	NA	0	NA	NA
rs113928389	A	NA	NA	NA	0	NA	NA	NA	0.03529	0	0	NA	0	NA	0.004808	NA	NA
rs147138431	A	NA	NA	NA	0.005051	NA	NA	NA	0.03529	0	0	NA	0	NA	0.004808	NA	NA
rs4283504	T	NA	NA	NA	0.01515	NA	NA	NA	0.04118	0.1641	0.1353	NA	0.1562	NA	0.07212	NA	NA
rs145297649	G	NA	NA	NA	0.0101	NA	NA	NA	0	0	0	NA	0	NA	0.01442	NA	NA
rs146957681	T	NA	NA	NA	0	NA	NA	NA	0	0	0	NA	0	NA	0	NA	NA
rs562177468	G	NA	NA	NA	0	NA	NA	NA	0	0	0	NA	0	NA	0	NA	NA
rs576030223	A	NA	NA	NA	0	NA	NA	NA	0	0	0	NA	0	NA	0	NA	NA
rs190385097	A	NA	NA	NA	0	NA	NA	NA	0	0	0	NA	0	NA	0	NA	NA
rs7283324	T	NA	NA	NA	0.3838	NA	NA	NA	0.4235	0.3359	0.2588	NA	0.2604	NA	0.1827	NA	NA
rs118108663	T	NA	NA	NA	0	NA	NA	NA	0	0	0	NA	0.005208	NA	0	NA	NA
rs61735789	A	NA	NA	NA	0	NA	NA	NA	0	0.01562	0.005882	NA	0	NA	0.009615	NA	NA
rs113564116	C	NA	NA	NA	0	NA	NA	NA	0	0	0	NA	0.005208	NA	0	NA	NA
rs2298659	A	NA	NA	NA	0.1465	NA	NA	NA	0.1765	0.3125	0.2529	NA	0.1823	NA	0.1587	NA	NA
rs150581606	A	NA	NA	NA	0	NA	NA	NA	0	0	0	NA	0	NA	0	NA	NA
rs116170128	C	NA	NA	NA	0.2071	NA	NA	NA	0.1	0.007812	0	NA	0.01562	NA	0.02885	NA	NA
rs143291395	A	NA	NA	NA	0	NA	NA	NA	0	0	0	NA	0	NA	0	NA	NA
rs112753686	T	NA	NA	NA	0.0202	NA	NA	NA	0.005882	0	0	NA	0	NA	0	NA	NA
rs75603675	A	NA	NA	NA	0.2778	NA	NA	NA	0.2176	0.2812	0.1529	NA	0.2292	NA	0.3269	NA	NA
rs553516093	A	NA	NA	NA	0	NA	NA	NA	0	0	0	NA	0.005208	NA	0	NA	NA
rs143460343	A	NA	NA	NA	0.0303	NA	NA	NA	0	0	0	NA	0	NA	0	NA	NA
rs184365262	T	NA	NA	NA	0	NA	NA	NA	0	0	0	NA	0	NA	0	NA	NA
rs571074719	T	NA	NA	NA	0	NA	NA	NA	0.01176	0	0	NA	0	NA	0	NA	NA
rs115975538	A	NA	NA	NA	0.04545	NA	NA	NA	0.03529	0	0	NA	0	NA	0.004808	NA	NA
rs147465180	A	NA	NA	NA	0	NA	NA	NA	0	0	0	NA	0	NA	0	NA	NA
rs548349124	G	NA	NA	NA	0	NA	NA	NA	0	0	0	NA	0	NA	0	NA	NA
rs139467735	T	NA	NA	NA	0.08586	NA	NA	NA	0.1824	0	0.005882	NA	0.005208	NA	0.01442	NA	NA
rs576042280	T	NA	NA	NA	0	NA	NA	NA	0	0	0	NA	0	NA	0	NA	NA
rs544083065	A	NA	NA	NA	0	NA	NA	NA	0	0	0	NA	0.005208	NA	0	NA	NA
rs551244742	A	NA	NA	NA	0	NA	NA	NA	0	0	0	NA	0	NA	0	NA	NA
rs149076631	T	NA	NA	NA	0.0101	NA	NA	NA	0.005882	0	0	NA	0	NA	0	NA	NA
rs35041537	T	0.1905	0.08333	0.1964	0.1465	0.2273	0.1333	0.1379	0.06471	0.3047	0.2294	0.3731	0.3333	0.15	0.3125	0	0.1042
rs56218846	A	NA	NA	NA	0.3131	NA	NA	NA	0.2235	0.2578	0.1412	NA	0.2031	NA	0.3029	NA	NA
rs527693716	T	NA	NA	NA	0	NA	NA	NA	0	0	0	NA	0	NA	0	NA	NA
rs189298425	A	NA	NA	NA	0.0101	NA	NA	NA	0	0	0	NA	0	NA	0	NA	NA

SNP	Allele	Lamas	Lambayeque	Lima	LWK	Matses	Moche	Moquegua	MSL	MXL	PEL	Pelotas	PJL	Puno	PUR	Qeros	Quechuas
rs562750287	A	NA	NA	NA	0.0101	NA	NA	NA	0	0	0	NA	0	NA	0	NA	NA
rs570413089	A	NA	NA	NA	0	NA	NA	NA	0	0	0	NA	0	NA	0	NA	NA
rs111383922	T	NA	NA	NA	0.005051	NA	NA	NA	0.005882	0	0	NA	0	NA	0.01923	NA	NA
rs559699558	A	NA	NA	NA	0	NA	NA	NA	0	0	0	NA	0.005208	NA	0	NA	NA
rs542060428	T	NA	NA	NA	0	NA	NA	NA	0	0	0	NA	0	NA	0	NA	NA
rs565142477	T	NA	NA	NA	0	NA	NA	NA	0	0	0	NA	0	NA	0	NA	NA
rs548742822	C	NA	NA	NA	0.005051	NA	NA	NA	0	0	0	NA	0	NA	0	NA	NA
rs148304071	T	NA	NA	NA	0	NA	NA	NA	0	0	0	NA	0	NA	0	NA	NA
rs455045	C	NA	NA	NA	0.2727	NA	NA	NA	0.2824	0.4531	0.4235	NA	0.3958	NA	0.4087	NA	NA
rs570441750	G	NA	NA	NA	0	NA	NA	NA	0	0	0	NA	0	NA	0	NA	NA
rs9974589	A	NA	NA	NA	0.197	NA	NA	NA	0.2706	0.4688	0.6412	NA	0.4479	NA	0.4423	NA	NA
rs2104810	A	NA	NA	NA	0.5455	NA	NA	NA	0.4353	0.4375	0.2824	NA	0.6823	NA	0.649	NA	NA
rs147711290	C	NA	NA	NA	NA	NA	NA	NA	NA	NA	NA	NA	NA	NA	NA	NA	NA
rs555056677	A	NA	NA	NA	0	NA	NA	NA	0	0	0	NA	0.005208	NA	0	NA	NA
rs9974933	G	NA	NA	NA	0.3333	NA	NA	NA	0.1941	0.2422	0.2529	NA	0.2865	NA	0.3125	NA	NA
rs544221548	A	NA	NA	NA	0	NA	NA	NA	0	0	0	NA	0.005208	NA	0	NA	NA
rs147827602	T	NA	NA	NA	0	NA	NA	NA	0.005882	0	0	NA	0	NA	0	NA	NA
rs462321	T	NA	NA	NA	0.399	NA	NA	NA	0.4353	0.5625	0.6941	NA	0.3021	NA	0.274	NA	NA
rs142659685	T	NA	NA	NA	0	NA	NA	NA	0	0	0	NA	0	NA	0.01923	NA	NA
rs150838246	C	NA	NA	NA	0	NA	NA	NA	0	0	0	NA	0	NA	0	NA	NA
rs112132031	C	NA	NA	NA	0.09596	NA	NA	NA	0.2353	0.2344	0.4588	NA	0.05729	NA	0.149	NA	NA
rs9974995	T	0.3095	0.125	0.2857	0.3232	0	0.15	0.2414	0.1941	0.2422	0.2529	0.2921	0.2865	0.15	0.3125	0.08333	0.25
rs557494549	TCAGGGAGTGCAGAGCAGGAGGGAC	NA	NA	NA	0.01515	NA	NA	NA	0	0	0	NA	0	NA	0	NA	NA
rs186929947	G	NA	NA	NA	0	NA	NA	NA	0	0	0	NA	0	NA	0	NA	NA
rs148509204	T	NA	NA	NA	0.0101	NA	NA	NA	0.02353	0	0	NA	0	NA	0.004808	NA	NA
rs202094412	A	NA	NA	NA	0	NA	NA	NA	0	0	0	NA	0	NA	0	NA	NA
rs575981127	A	NA	NA	NA	0	NA	NA	NA	0	0	0	NA	0.005208	NA	0	NA	NA
rs546775580	T	NA	NA	NA	0	NA	NA	NA	0	0	0	NA	0	NA	0	NA	NA
rs185188053	T	NA	NA	NA	0	NA	NA	NA	0	0	0	NA	0	NA	0	NA	NA
rs577381045	A	NA	NA	NA	0	NA	NA	NA	0	0	0	NA	0	NA	0	NA	NA
rs62217525	T	NA	NA	NA	0	NA	NA	NA	0	0.03906	0.02353	NA	0.03125	NA	0.05288	NA	NA
rs561043908	A	NA	NA	NA	0	NA	NA	NA	0	0	0	NA	0	NA	0	NA	NA
rs367879274	T	NA	NA	NA	0.005051	NA	NA	NA	0	0	0	NA	0	NA	0	NA	NA
rs568167564	G	NA	NA	NA	0	NA	NA	NA	0	0	0.005882	NA	0	NA	0	NA	NA
rs563633617	A	NA	NA	NA	0	NA	NA	NA	0	0	0	NA	0	NA	0	NA	NA
rs537935645	T	NA	NA	NA	0	NA	NA	NA	0	0	0	NA	0	NA	0	NA	NA
rs9985159	C	0.5476	0.7917	0.7857	0.6212	0.7727	0.8167	0.8621	0.4588	0.6719	0.7294	0.7539	0.7656	0.8375	0.7885	0.91667	0.75
rs61735795	A	NA	NA	NA	NA	NA	NA	NA	NA	NA	NA	NA	NA	NA	NA	NA	NA
rs557712450	C	NA	NA	NA	0	NA	NA	NA	0	0	0	NA	0	NA	0	NA	NA
rs139305247	T	NA	NA	NA	0	NA	NA	NA	0	0.1094	0.03529	NA	0	NA	0.004808	NA	NA
rs548653178	A	NA	NA	NA	0	NA	NA	NA	0	0	0	NA	0	NA	0	NA	NA
rs146385718	C	NA	NA	NA	0.0303	NA	NA	NA	0	0	0	NA	0	NA	0	NA	NA
rs28360562	C	NA	NA	NA	0.0404	NA	NA	NA	0.01176	0.02344	0.01176	NA	0.125	NA	0.03846	NA	NA
rs9983330	G	NA	NA	NA	0.2828	NA	NA	NA	0.2588	0.1797	0.06471	NA	0.2396	NA	0.1635	NA	NA
rs539864066	G	NA	NA	NA	0.005051	NA	NA	NA	0.005882	0	0	NA	0	NA	0	NA	NA

SNP	Allele	Lamas	Lambayeque	Lima	LWK	Matses	Moche	Moquegua	MSL	MXL	PEL	Pelotas	PJL	Puno	PUR	Qeros	Quechuas
rs562014334	A	NA	NA	NA	0.005051	NA	NA	NA	0	0	0	NA	0	NA	0	NA	NA
rs118134524	A	NA	NA	NA	0	NA	NA	NA	0	0	0	NA	0	NA	0	NA	NA
rs574811036	A	NA	NA	NA	0	NA	NA	NA	0	0	0	NA	0	NA	0	NA	NA
rs115720411	A	NA	NA	NA	0.1313	NA	NA	NA	0.2471	0.007812	0.005882	NA	0	NA	0.04808	NA	NA
rs368812287	T	NA	NA	NA	0	NA	NA	NA	0	0	0	NA	0	NA	0	NA	NA
rs2410428	T	NA	NA	NA	0.1263	NA	NA	NA	0.07059	0.2344	0.2529	NA	0.2865	NA	0.2981	NA	NA
rs577480005	A	NA	NA	NA	0.0101	NA	NA	NA	0.005882	0.007812	0	NA	0	NA	0.01442	NA	NA
rs543815381	T	NA	NA	NA	0	NA	NA	NA	0	0	0.01176	NA	0	NA	0	NA	NA
rs142834250	G	NA	NA	NA	0	NA	NA	NA	0	0	0	NA	0	NA	0	NA	NA
rs576292358	A	NA	NA	NA	0	NA	NA	NA	0	0	0	NA	0	NA	0.009615	NA	NA
rs141603473	A	NA	NA	NA	0.08081	NA	NA	NA	0.05294	0	0	NA	0	NA	0	NA	NA
rs143523726	G	NA	NA	NA	0.005051	NA	NA	NA	0	0	0	NA	0	NA	0	NA	NA
rs375408	G	NA	NA	NA	0.0101	NA	NA	NA	0.01176	0.2266	0.4471	NA	0.05729	NA	0.1154	NA	NA
rs370347248	C	NA	NA	NA	0	NA	NA	NA	0.005882	0	0	NA	0	NA	0	NA	NA
rs539123545	C	NA	NA	NA	0	NA	NA	NA	0	0	0	NA	0	NA	0	NA	NA
rs4303794	C	NA	NA	NA	0.2879	NA	NA	NA	0.2176	0.2812	0.1588	NA	0.2292	NA	0.3269	NA	NA
rs151035593	T	NA	NA	NA	0.01515	NA	NA	NA	0.01765	0	0	NA	0	NA	0	NA	NA
rs370896169	C	NA	NA	NA	0	NA	NA	NA	0	0	0	NA	0	NA	0	NA	NA
rs575167859	T	NA	NA	NA	0	NA	NA	NA	0	0	0	NA	0	NA	0.004808	NA	NA
rs35050484	A	NA	NA	NA	0	NA	NA	NA	0	0.007812	0.01765	NA	0.01042	NA	0.0625	NA	NA
rs56136037	T	NA	NA	NA	0	NA	NA	NA	0	0.007812	0.02353	NA	0.02604	NA	0.03365	NA	NA
rs539767738	A	NA	NA	NA	0	NA	NA	NA	0	0	0	NA	0	NA	0	NA	NA
rs533229030	G	NA	NA	NA	0	NA	NA	NA	0	0	0	NA	0	NA	0	NA	NA
rs465576	C	NA	NA	NA	0.09596	NA	NA	NA	0.2	0.2344	0.4588	NA	0.05729	NA	0.1442	NA	NA
rs570494222	T	NA	NA	NA	0	NA	NA	NA	0	0	0	NA	0	NA	0	NA	NA
rs4818240	T	NA	NA	NA	0.1162	NA	NA	NA	0.2235	0.2266	0.4471	NA	0.03646	NA	0.1635	NA	NA
rs9636988	C	0.3095	0.125	0.3036	0.3232	0	0.15	0.2414	0.1941	0.2422	0.2529	0.2935	0.2865	0.15	0.3125	0.08333	0.25
rs541887371	T	NA	NA	NA	0	NA	NA	NA	0	0	0	NA	0	NA	0	NA	NA
rs537787922	A	NA	NA	NA	0	NA	NA	NA	0	0	0	NA	0	NA	0	NA	NA
rs139829932	T	NA	NA	NA	0.0202	NA	NA	NA	0.02941	0.007812	0	NA	0	NA	0.004808	NA	NA
rs200615061	C	NA	NA	NA	0	NA	NA	NA	0	0	0	NA	0.01042	NA	0.004808	NA	NA
rs3787947	T	NA	NA	NA	0.4495	NA	NA	NA	0.4118	0.2578	0.08235	NA	0.3177	NA	0.2212	NA	NA
rs402197	T	NA	NA	NA	0.01515	NA	NA	NA	0.02941	0.2266	0.4471	NA	0.05729	NA	0.1346	NA	NA
rs562203987	T	NA	NA	NA	0	NA	NA	NA	0	0	0	NA	0	NA	0	NA	NA
rs141764184	A	NA	NA	NA	0	NA	NA	NA	0	0	0	NA	0	NA	0	NA	NA
rs116577479	A	NA	NA	NA	0.05556	NA	NA	NA	0.04706	0.007812	0	NA	0	NA	0.01442	NA	NA
rs118133613	A	NA	NA	NA	0	NA	NA	NA	0	0	0.005882	NA	0	NA	0	NA	NA
rs118108194	G	NA	NA	NA	0	NA	NA	NA	0	0	0	NA	0	NA	0.009615	NA	NA
rs138763189	A	NA	NA	NA	0.02525	NA	NA	NA	0.01176	0	0	NA	0	NA	0	NA	NA
rs138365638	TGG	NA	NA	NA	0.0202	NA	NA	NA	0.01176	0.2188	0.4471	NA	0.05729	NA	0.1106	NA	NA
rs551442751	T	NA	NA	NA	0	NA	NA	NA	0	0	0	NA	0	NA	0	NA	NA
rs539019775	T	NA	NA	NA	0	NA	NA	NA	0.01176	0	0	NA	0	NA	0	NA	NA
rs112657409	T	0	0	0	0.09596	0	0	0.01724	0.07059	0.007812	0	0.0205	0	0	0.009615	0	0
rs577689706	A	NA	NA	NA	0	NA	NA	NA	0	0	0	NA	0	NA	0	NA	NA
rs147349930	T	NA	NA	NA	0	NA	NA	NA	0.03529	0	0	NA	0	NA	0.004808	NA	NA

SNP	Allele	Lamas	Lambayeque	Lima	LWK	Matses	Moche	Moquegua	MSL	MXL	PEL	Pelotas	PJL	Puno	PUR	Qeros	Quechuas
rs557531433	T	NA	NA	NA	0	NA	NA	NA	0	0	0	NA	0	NA	0	NA	NA
rs144359794	T	NA	NA	NA	0	NA	NA	NA	0	0	0	NA	0	NA	0	NA	NA
rs187426170	T	NA	NA	NA	0	NA	NA	NA	0	0.007812	0	NA	0	NA	0.004808	NA	NA
rs199575615	G	NA	NA	NA	0	NA	NA	NA	0	0	0	NA	0	NA	0	NA	NA
rs529737187	A	NA	NA	NA	0	NA	NA	NA	0.01176	0	0	NA	0	NA	0	NA	NA
rs576447019	A	NA	NA	NA	0	NA	NA	NA	0	0	0	NA	0	NA	0	NA	NA
rs145900878	T	NA	NA	NA	0.06061	NA	NA	NA	0.02353	0.007812	0	NA	0	NA	0.01442	NA	NA
rs9977234	T	NA	NA	NA	0.1515	NA	NA	NA	0.06471	0.1953	0.2353	NA	0.2812	NA	0.2548	NA	NA
rs540024706	C	NA	NA	NA	0	NA	NA	NA	0	0	0	NA	0.01042	NA	0	NA	NA
rs528640390	G	NA	NA	NA	0	NA	NA	NA	0.01176	0	0	NA	0	NA	0	NA	NA
rs546337899	G	NA	NA	NA	0.01515	NA	NA	NA	0	0.007812	0	NA	0	NA	0.01442	NA	NA
rs141323355	A	NA	NA	NA	0	NA	NA	NA	0	0	0	NA	0	NA	0	NA	NA
rs386519	G	NA	NA	NA	0.0101	NA	NA	NA	0	0	0	NA	0	NA	0.004808	NA	NA
rs561327241	T	NA	NA	NA	0	NA	NA	NA	0	0	0	NA	0.005208	NA	0	NA	NA
rs572351113	A	NA	NA	NA	0	NA	NA	NA	0.005882	0	0	NA	0	NA	0	NA	NA
rs189425119	A	NA	NA	NA	0	NA	NA	NA	0	0	0	NA	0	NA	0	NA	NA
rs566485026	G	NA	NA	NA	0	NA	NA	NA	0	0	0	NA	0.005208	NA	0	NA	NA
rs2298665	C	NA	NA	NA	0	NA	NA	NA	0	0	0	NA	0	NA	0	NA	NA
rs142769034	T	NA	NA	NA	0	NA	NA	NA	0	0	0	NA	0.005208	NA	0.004808	NA	NA
rs74564819	C	NA	NA	NA	0	NA	NA	NA	0	0	0	NA	0	NA	0	NA	NA
rs2298857	A	0.3095	0.2083	0.2679	0.404	0	0.1333	0.2414	0.4706	0.2109	0.2471	0.2943	0.2917	0.15	0.3365	0.08333	0.25
rs545232590	T	NA	NA	NA	0	NA	NA	NA	0	0	0	NA	0	NA	0	NA	NA
rs529218967	G	NA	NA	NA	0	NA	NA	NA	0	0	0.005882	NA	0	NA	0	NA	NA
rs528337775	T	NA	NA	NA	0	NA	NA	NA	0	0	0	NA	0	NA	0	NA	NA
rs563980567	A	NA	NA	NA	0	NA	NA	NA	0	0	0	NA	0	NA	0	NA	NA
rs534913861	T	NA	NA	NA	0	NA	NA	NA	0	0	0	NA	0	NA	0	NA	NA
rs535348902	A	NA	NA	NA	0	NA	NA	NA	0	0	0	NA	0	NA	0	NA	NA
rs555405939	C	NA	NA	NA	0	NA	NA	NA	0	0	0	NA	0	NA	0	NA	NA
rs187037274	A	NA	NA	NA	0	NA	NA	NA	0.01765	0	0	NA	0	NA	0	NA	NA
rs141027872	T	NA	NA	NA	0.005051	NA	NA	NA	0.005882	0	0	NA	0	NA	0	NA	NA
rs191394761	T	NA	NA	NA	0	NA	NA	NA	0	0	0	NA	0.005208	NA	0.009615	NA	NA
rs540964406	G	NA	NA	NA	0	NA	NA	NA	0	0	0	NA	0	NA	0	NA	NA
rs544440280	A	NA	NA	NA	0	NA	NA	NA	0	0	0	NA	0	NA	0	NA	NA
rs146845793	C	NA	NA	NA	0	NA	NA	NA	0	0	0	NA	0	NA	0	NA	NA
rs372665499	T	NA	NA	NA	0	NA	NA	NA	0	0	0	NA	0	NA	0	NA	NA
rs538276300	A	NA	NA	NA	0	NA	NA	NA	0	0	0	NA	0	NA	0	NA	NA
rs113562865	T	NA	NA	NA	0.06566	NA	NA	NA	0.1765	0.007812	0.005882	NA	0	NA	0.05288	NA	NA
rs571417292	A	NA	NA	NA	0	NA	NA	NA	0	0	0	NA	0	NA	0	NA	NA
rs542865477	A	NA	NA	NA	0	NA	NA	NA	0	0	0	NA	0.005208	NA	0	NA	NA
rs544110139	G	NA	NA	NA	0	NA	NA	NA	0.01176	0	0	NA	0	NA	0	NA	NA
rs142914234	C	NA	NA	NA	0.0303	NA	NA	NA	0	0	0	NA	0	NA	0	NA	NA
rs114549926	A	NA	NA	NA	0.005051	NA	NA	NA	0.04118	0	0	NA	0	NA	0.02404	NA	NA
rs549289994	C	NA	NA	NA	0	NA	NA	NA	0	0	0	NA	0	NA	0	NA	NA
rs555327583	A	NA	NA	NA	0	NA	NA	NA	0	0	0	NA	0	NA	0	NA	NA
rs111220533	G	NA	NA	NA	0.3737	NA	NA	NA	0.2235	0.25	0.1471	NA	0.4167	NA	0.4375	NA	NA

SNP	Allele	Lamas	Lambayeque	Lima	LWK	Matses	Moche	Moquegua	MSL	MXL	PEL	Pelotas	PJL	Puno	PUR	Qeros	Quechuas
rs553400003	A	NA	NA	NA	0	NA	NA	NA	0	0	0	NA	0.02083	NA	0	NA	NA
rs530306757	A	NA	NA	NA	0	NA	NA	NA	0	0	0	NA	0	NA	0	NA	NA
rs561412657	G	NA	NA	NA	0	NA	NA	NA	0	0	0	NA	0	NA	0	NA	NA
rs565690211	G	NA	NA	NA	0	NA	NA	NA	0	0	0	NA	0	NA	0	NA	NA
rs137929897	T	NA	NA	NA	0.01515	NA	NA	NA	0	0	0	NA	0	NA	0	NA	NA
rs144458055	A	NA	NA	NA	0	NA	NA	NA	0	0.01562	0	NA	0.02604	NA	0.004854	NA	NA
rs141722242	A	NA	NA	NA	0.0303	NA	NA	NA	0	0	0	NA	0	NA	0.009615	NA	NA
rs565720190	A	NA	NA	NA	0	NA	NA	NA	0	0	0	NA	0.005208	NA	0	NA	NA
rs530051850	C	NA	NA	NA	0	NA	NA	NA	0	0	0	NA	0	NA	0	NA	NA
rs115967323	T	NA	NA	NA	0	NA	NA	NA	0	0	0	NA	0	NA	0	NA	NA

Table S4-A – TMPRSS2 allele frequencies continuation. Bold = functionally relevant SNPs found in our databases; NA = missing data.

SNP	Allele	Salvador	Shimaa	Shipibo	STU	Tacna	Tallanes	Trujillo	TSI	Tumbes	Uros	YRI
**rs456298**	**T**	**0.32**	**NA**	**NA**	**0.38**	**NA**	**NA**	**NA**	**0.20**	**NA**	**NA**	**0.34**
**rs12329760**	**T**	**0.2127**	**0**	**0.03125**	**0.2843**	**0.04545**	**0**	**0.0625**	**0.2009**	**0.07576**	**0**	**0.2546**
**rs2276205**	**G**	**0.1001**	**0.02174**	**0**	**0.02941**	**0.02273**	**0**	**0.04167**	**0.03738**	**0**	**0**	**0.1481**
**rs383510**	**T**	**NA**	**NA**	**NA**	**0.4363**	**NA**	**NA**	**NA**	**0.4673**	**NA**	**NA**	**0.3241**
**rs2070788**	**G**	**0.3713**	**0.8478**	**0.5625**	**0.4608**	**0.75**	**0.8**	**0.6667**	**0.4346**	**0.6667**	**0.625**	**0.3102**
rs149270377	A	NA	NA	NA	0	NA	NA	NA	0	NA	NA	0.01852
rs75833467	A	NA	NA	NA	0.009804	NA	NA	NA	0.09346	NA	NA	0
rs569429288	A	NA	NA	NA	0	NA	NA	NA	0	NA	NA	0
rs554852594	C	NA	NA	NA	0	NA	NA	NA	0	NA	NA	0.00463
rs537997349	G	NA	NA	NA	0.009804	NA	NA	NA	0.004673	NA	NA	0.01389
rs569432639	C	NA	NA	NA	0	NA	NA	NA	0	NA	NA	0.00463
rs535019926	C	NA	NA	NA	0	NA	NA	NA	0	NA	NA	0
rs368646465	T	NA	NA	NA	0	NA	NA	NA	0	NA	NA	0
rs9305744	A	0.2846	NA	NA	0.2941	NA	NA	NA	0.2243	NA	NA	0.3009
rs148910500	T	NA	NA	NA	0	NA	NA	NA	0	NA	NA	0
rs142710225	T	NA	NA	NA	0	NA	NA	NA	0	NA	NA	0.00463
rs368735421	T	NA	NA	NA	0	NA	NA	NA	0.004673	NA	NA	0
rs140458174	A	NA	NA	NA	0	NA	NA	NA	0	NA	NA	0
rs532232636	T	NA	NA	NA	0	NA	NA	NA	0	NA	NA	0
rs381179	T	NA	NA	NA	0	NA	NA	NA	0	NA	NA	0
rs572410855	A	NA	NA	NA	0	NA	NA	NA	0	NA	NA	0
rs111620846	A	NA	NA	NA	0	NA	NA	NA	0	NA	NA	0.01389
rs377496737	T	NA	NA	NA	0	NA	NA	NA	0	NA	NA	0
rs533552237	A	NA	NA	NA	0	NA	NA	NA	0	NA	NA	0
rs76315847	A	0.00382	0	0	0	0	0	0	0	0	0	0.01852
rs377348479	A	NA	NA	NA	0	NA	NA	NA	0	NA	NA	0
rs180818774	C	NA	NA	NA	0	NA	NA	NA	0	NA	NA	0.00463
rs462471	A	0.3021	0.7826	0.5	0.3627	0.6818	0.7286	0.5833	0.1963	0.5152	0.625	0.3426
rs554357472	A	NA	NA	NA	0	NA	NA	NA	0	NA	NA	0
rs117652812	T	NA	NA	NA	0	NA	NA	NA	0	NA	NA	0

SNP	Allele	Salvador	Shimaa	Shipibo	STU	Tacna	Tallanes	Trujillo	TSI	Tumbes	Uros	YRI
rs56097233	CAG	NA	NA	NA	0.402	NA	NA	NA	0.2523	NA	NA	0.4259
rs573178525	A	NA	NA	NA	0	NA	NA	NA	0	NA	NA	0
rs186168224	T	NA	NA	NA	0	NA	NA	NA	0	NA	NA	0
rs551103429	T	NA	NA	NA	0	NA	NA	NA	0.004673	NA	NA	0
rs462574	A	0.1677	0.7826	0.5	0.299	0.6667	0.7206	0.5625	0.05607	0.4545	0.625	0.1481
rs543681861	C	NA	NA	NA	0	NA	NA	NA	0	NA	NA	0
rs557632131	T	NA	NA	NA	0	NA	NA	NA	0	NA	NA	0
rs186425543	G	NA	NA	NA	0	NA	NA	NA	0	NA	NA	0
rs542228195	C	NA	NA	NA	0	NA	NA	NA	0	NA	NA	0
rs144052153	G	NA	NA	NA	0	NA	NA	NA	0	NA	NA	0
rs374377315	T	NA	NA	NA	0	NA	NA	NA	0	NA	NA	0
rs73357664	A	NA	NA	NA	0	NA	NA	NA	0	NA	NA	0.0463
rs373196115	A	NA	NA	NA	0	NA	NA	NA	0	NA	NA	0
rs181107522	A	NA	NA	NA	0	NA	NA	NA	0	NA	NA	0.00463
rs569716669	C	NA	NA	NA	0	NA	NA	NA	0.004673	NA	NA	0
rs535834505	G	NA	NA	NA	0	NA	NA	NA	0	NA	NA	0
rs28707508	A	NA	NA	NA	0.2451	NA	NA	NA	0.4112	NA	NA	0.3843
rs458280	T	NA	NA	NA	0.06863	NA	NA	NA	0.01402	NA	NA	0
rs567208488	CAG	NA	NA	NA	0.009804	NA	NA	NA	0	NA	NA	0
rs186510586	C	NA	NA	NA	0	NA	NA	NA	0.004673	NA	NA	0
rs140532244	C	NA	NA	NA	0	NA	NA	NA	0	NA	NA	0
rs193019598	A	NA	NA	NA	0	NA	NA	NA	0.004673	NA	NA	0
rs533708336	A	NA	NA	NA	0	NA	NA	NA	0	NA	NA	0.009259
rs188198121	A	NA	NA	NA	0	NA	NA	NA	0	NA	NA	0
rs142119028	T	NA	NA	NA	0	NA	NA	NA	0	NA	NA	0.009259
rs141128014	G	NA	NA	NA	0	NA	NA	NA	0	NA	NA	0.00463
rs2838039	C	NA	NA	NA	0.299	NA	NA	NA	0.215	NA	NA	0.412
rs558683527	C	NA	NA	NA	0.01471	NA	NA	NA	0	NA	NA	0
rs545307151	G	NA	NA	NA	0	NA	NA	NA	0	NA	NA	0
rs9976780	C	0.3504	NA	NA	0.3725	NA	NA	NA	0.2336	NA	NA	0.4074
rs553788077	A	NA	NA	NA	0	NA	NA	NA	0	NA	NA	0
rs539639766	C	NA	NA	NA	0	NA	NA	NA	0	NA	NA	0
rs543253134	A	NA	NA	NA	0	NA	NA	NA	0	NA	NA	0
rs79971314	A	NA	NA	NA	0.004902	NA	NA	NA	0.004673	NA	NA	0.3009
rs148719900	T	NA	NA	NA	0	NA	NA	NA	0	NA	NA	0
rs144948620	A	NA	NA	NA	0.004902	NA	NA	NA	0.02336	NA	NA	0
rs536525396	A	NA	NA	NA	0	NA	NA	NA	0	NA	NA	0.009259
rs548341299	C	NA	NA	NA	0	NA	NA	NA	0	NA	NA	0
rs139816990	A	NA	NA	NA	0.009804	NA	NA	NA	0	NA	NA	0.01389
rs536147878	A	NA	NA	NA	0	NA	NA	NA	0	NA	NA	0
rs564793560	G	NA	NA	NA	0	NA	NA	NA	0	NA	NA	0.009259
rs192011400	T	NA	NA	NA	0	NA	NA	NA	0	NA	NA	0
rs531024324	A	NA	NA	NA	0	NA	NA	NA	0.004673	NA	NA	0
rs192666663	A	NA	NA	NA	0	NA	NA	NA	0	NA	NA	0
rs138282462	T	NA	NA	NA	0	NA	NA	NA	0	NA	NA	0

SNP	Allele	Salvador	Shimaa	Shipibo	STU	Tacna	Tallanes	Trujillo	TSI	Tumbes	Uros	YRI
rs143109187	G	NA	NA	NA	0	NA	NA	NA	0	NA	NA	0
rs150014829	A	NA	NA	NA	0	NA	NA	NA	0	NA	NA	0
rs34561135	A	NA	NA	NA	0.02451	NA	NA	NA	0.02336	NA	NA	0
rs11088551	G	NA	NA	NA	0.2745	NA	NA	NA	0.4393	NA	NA	0.412
rs200169208	A	NA	NA	NA	0	NA	NA	NA	0	NA	NA	0
rs566903241	C	NA	NA	NA	0	NA	NA	NA	0	NA	NA	0
rs146132415	A	NA	NA	NA	0	NA	NA	NA	0	NA	NA	0
rs61735791	T	NA	NA	NA	0	NA	NA	NA	0	NA	NA	0
rs571587918	C	NA	NA	NA	0	NA	NA	NA	0	NA	NA	0
rs78459594	C	NA	0	0	NA	0	0	0	NA	0	0	NA
rs389001	G	NA	NA	NA	0.2402	NA	NA	NA	0.009346	NA	NA	0.2546
rs200164183	T	NA	NA	NA	0	NA	NA	NA	0	NA	NA	0
rs548354821	C	NA	NA	NA	0	NA	NA	NA	0	NA	NA	0
rs2187238	C	0.1329	0.06522	0.25	0.1324	0.1136	0.1857	0.1042	0.2103	0.197	0.3438	0.06019
rs556456416	C	NA	NA	NA	0	NA	NA	NA	0	NA	NA	0
rs181414852	G	NA	NA	NA	0	NA	NA	NA	0	NA	NA	0
rs553787982	A	NA	NA	NA	0	NA	NA	NA	0	NA	NA	0
rs417888	A	NA	NA	NA	0.4412	NA	NA	NA	0.4626	NA	NA	0.1204
rs189793246	G	NA	NA	NA	0	NA	NA	NA	0	NA	NA	0
rs556371948	T	NA	NA	NA	0	NA	NA	NA	0	NA	NA	0
rs546273967	T	NA	NA	NA	0	NA	NA	NA	0	NA	NA	0
rs76000363	A	NA	NA	NA	0.07353	NA	NA	NA	0.1355	NA	NA	0.05556
rs557639119	A	NA	NA	NA	0	NA	NA	NA	0	NA	NA	0.01389
rs563507800	A	NA	NA	NA	0	NA	NA	NA	0	NA	NA	0
rs148125094	T	NA	NA	NA	NA	NA	NA	NA	NA	NA	NA	NA
rs73372161	A	0.08173	NA	NA	0.004902	NA	NA	NA	0.004673	NA	NA	0.213
rs555995855	C	NA	NA	NA	0.06863	NA	NA	NA	0	NA	NA	0
rs11281229	TCCAGG	NA	NA	NA	0.2647	NA	NA	NA	0.4346	NA	NA	0.4028
rs559098267	T	NA	NA	NA	0	NA	NA	NA	0	NA	NA	0
rs191693032	G	NA	NA	NA	0	NA	NA	NA	0	NA	NA	0
rs187648498	C	NA	NA	NA	0	NA	NA	NA	0	NA	NA	0
rs541109823	T	NA	NA	NA	0	NA	NA	NA	0	NA	NA	0
rs559668949	G	NA	NA	NA	0	NA	NA	NA	0	NA	NA	0
rs113670863	T	NA	NA	NA	0	NA	NA	NA	0	NA	NA	0
rs545494331	C	NA	NA	NA	0	NA	NA	NA	0	NA	NA	0
rs528691645	A	NA	NA	NA	0	NA	NA	NA	0	NA	NA	0
rs558543141	T	NA	NA	NA	0	NA	NA	NA	0	NA	NA	0
rs145877432	A	NA	NA	NA	0	NA	NA	NA	0	NA	NA	0.01389
rs531615786	C	NA	NA	NA	0	NA	NA	NA	0	NA	NA	0
rs570264808	A	NA	NA	NA	0	NA	NA	NA	0	NA	NA	0.00463
rs371012018	A	NA	NA	NA	0.004902	NA	NA	NA	0	NA	NA	0
rs534326511	T	NA	NA	NA	0	NA	NA	NA	0	NA	NA	0
rs200744510	C	NA	NA	NA	NA	NA	NA	NA	NA	NA	NA	NA
rs77996454	A	0.03207	NA	NA	0	NA	NA	NA	0	NA	NA	0.0463
rs140259165	T	NA	NA	NA	0	NA	NA	NA	0	NA	NA	0

SNP	Allele	Salvador	Shimaa	Shipibo	STU	Tacna	Tallanes	Trujillo	TSI	Tumbes	Uros	YRI
rs58978895	T	NA	NA	NA	0	NA	NA	NA	0	NA	NA	0.213
rs564981706	T	NA	NA	NA	0	NA	NA	NA	0	NA	NA	0
rs149173609	T	NA	NA	NA	0	NA	NA	NA	0	NA	NA	0
rs559934709	T	NA	NA	NA	0	NA	NA	NA	0	NA	NA	0.009259
rs569899862	A	NA	NA	NA	0	NA	NA	NA	0	NA	NA	0
rs540844961	G	NA	NA	NA	0	NA	NA	NA	0.004673	NA	NA	0
rs189582211	A	NA	NA	NA	0	NA	NA	NA	0	NA	NA	0
rs553097317	T	NA	NA	NA	0	NA	NA	NA	0	NA	NA	0
rs563693995	G	NA	NA	NA	0	NA	NA	NA	0	NA	NA	0
rs2298658	T	NA	NA	NA	0	NA	NA	NA	0	NA	NA	0
rs532882572	T	NA	NA	NA	0	NA	NA	NA	0	NA	NA	0
rs145347702	C	NA	NA	NA	0	NA	NA	NA	0	NA	NA	0
rs148988435	A	NA	NA	NA	0	NA	NA	NA	0	NA	NA	0
rs561557938	A	NA	NA	NA	0	NA	NA	NA	0	NA	NA	0
rs140230703	G	NA	NA	NA	0	NA	NA	NA	0	NA	NA	0
rs141232947	G	NA	NA	NA	0	NA	NA	NA	0	NA	NA	0
rs61735794	T	NA	NA	NA	0	NA	NA	NA	0.0514	NA	NA	0
rs117888036	A	NA	NA	NA	0	NA	NA	NA	0	NA	NA	0
rs538864203	A	NA	NA	NA	0	NA	NA	NA	0	NA	NA	0
rs540165785	A	NA	NA	NA	0	NA	NA	NA	0	NA	NA	0
rs79517809	C	NA	NA	NA	0	NA	NA	NA	0	NA	NA	0.009259
rs144988776	T	NA	NA	NA	0.004902	NA	NA	NA	0.02336	NA	NA	0
rs565946159	C	NA	NA	NA	0.004902	NA	NA	NA	0	NA	NA	0
rs574894406	G	NA	NA	NA	0	NA	NA	NA	0	NA	NA	0
rs145570856	A	NA	NA	NA	0	NA	NA	NA	0	NA	NA	0.03241
rs146142989	A	NA	NA	NA	0	NA	NA	NA	0	NA	NA	0
rs556966925	C	NA	NA	NA	0	NA	NA	NA	0	NA	NA	0.00463
rs573704321	T	NA	NA	NA	0	NA	NA	NA	0	NA	NA	0
rs376074355	T	NA	NA	NA	0.004902	NA	NA	NA	0.004673	NA	NA	0
rs61325328	G	NA	NA	NA	0	NA	NA	NA	0	NA	NA	0.00463
rs9979311	G	NA	NA	NA	0.299	NA	NA	NA	0.215	NA	NA	0.4028
rs538655114	A	NA	NA	NA	0	NA	NA	NA	0	NA	NA	0
rs150066796	A	NA	NA	NA	0	NA	NA	NA	0	NA	NA	0
rs201093031	G	NA	NA	NA	0	NA	NA	NA	0	NA	NA	0
rs378616	C	0.2544	NA	NA	0.2255	NA	NA	NA	0.229	NA	NA	0.2315
rs573152591	A	NA	NA	NA	0	NA	NA	NA	0	NA	NA	0.00463
rs4818241	A	NA	NA	NA	0.07353	NA	NA	NA	0.01869	NA	NA	0
rs189181802	T	NA	NA	NA	0	NA	NA	NA	0	NA	NA	0.009259
rs566323147	A	NA	NA	NA	0	NA	NA	NA	0	NA	NA	0
rs184164037	C	NA	NA	NA	0	NA	NA	NA	0	NA	NA	0.009259
rs77675406	A	0.06684	0	0	0.07353	0.02273	0	0.04167	0.1355	0.06061	0	0.0463
rs528276471	G	NA	NA	NA	0	NA	NA	NA	0	NA	NA	0
rs552912416	T	NA	NA	NA	0	NA	NA	NA	0	NA	NA	0
rs572279833	A	NA	NA	NA	0	NA	NA	NA	0	NA	NA	0
rs559830930	G	NA	NA	NA	0	NA	NA	NA	0	NA	NA	0

SNP	Allele	Salvador	Shimaa	Shipibo	STU	Tacna	Tallanes	Trujillo	TSI	Tumbes	Uros	YRI
rs2298660	T	NA	NA	NA	0.1765	NA	NA	NA	0.1682	NA	NA	0.3889
rs527684898	G	NA	NA	NA	0	NA	NA	NA	0	NA	NA	0
rs181332845	G	NA	NA	NA	0	NA	NA	NA	0	NA	NA	0.00463
rs182175876	G	NA	NA	NA	0	NA	NA	NA	0	NA	NA	0
rs539218228	A	NA	NA	NA	0	NA	NA	NA	0	NA	NA	0
rs116865960	A	NA	NA	NA	0.004902	NA	NA	NA	0.004673	NA	NA	0.00463
rs183398094	C	NA	NA	NA	0	NA	NA	NA	0	NA	NA	0
rs567340892	A	NA	NA	NA	0	NA	NA	NA	0	NA	NA	0.00463
rs190125027	T	NA	NA	NA	0	NA	NA	NA	0	NA	NA	0
rs75430506	A	NA	NA	NA	0	NA	NA	NA	0	NA	NA	0
rs138812485	A	NA	NA	NA	0	NA	NA	NA	0	NA	NA	0
rs56066678	T	0.3476	0.06522	0.1875	0.2353	0.1818	0.1714	0.2292	0.2804	0.1818	0.375	0.4491
rs561063944	A	NA	NA	NA	0	NA	NA	NA	0	NA	NA	0
rs17854725	G	NA	NA	NA	0.4069	NA	NA	NA	0.5	NA	NA	0.3611
rs533050705	A	NA	NA	NA	0	NA	NA	NA	0	NA	NA	0
rs545910743	C	NA	NA	NA	0	NA	NA	NA	0	NA	NA	0
rs149021153	G	NA	NA	NA	0	NA	NA	NA	0	NA	NA	0.009259
rs371583288	A	NA	NA	NA	0.004902	NA	NA	NA	0	NA	NA	0
rs552174617	C	NA	NA	NA	0	NA	NA	NA	0	NA	NA	0
rs144157441	A	NA	NA	NA	0	NA	NA	NA	0	NA	NA	0.009259
rs559811756	A	NA	NA	NA	0	NA	NA	NA	0	NA	NA	0.009259
rs536642840	A	NA	NA	NA	0	NA	NA	NA	0	NA	NA	0
rs572530227	T	NA	NA	NA	0	NA	NA	NA	0	NA	NA	0
rs551045243	A	NA	NA	NA	0	NA	NA	NA	0.004673	NA	NA	0
rs374886738	C	NA	NA	NA	0	NA	NA	NA	0	NA	NA	0
rs557714443	A	NA	NA	NA	0	NA	NA	NA	0	NA	NA	0
rs402303	C	0.5291	0.1957	0.4688	0.6176	0.25	0.2429	0.3333	0.7336	0.5	0.375	0.3704
rs544822322	C	NA	NA	NA	0	NA	NA	NA	0	NA	NA	0
rs186429734	A	NA	NA	NA	0	NA	NA	NA	0	NA	NA	0
rs138056981	T	NA	NA	NA	0	NA	NA	NA	0.009346	NA	NA	0
rs79468500	G	NA	NA	NA	0.009804	NA	NA	NA	0.04673	NA	NA	0.02778
rs2156301	T	NA	NA	NA	0.07353	NA	NA	NA	0.01869	NA	NA	0.009259
rs191228684	T	NA	NA	NA	0	NA	NA	NA	0	NA	NA	0
rs563812695	A	NA	NA	NA	0	NA	NA	NA	0	NA	NA	0
rs8128074	T	0.08486	0.3261	0.125	0.152	0.25	0.1143	0.1458	0.08879	0.0303	0.0625	0.02778
rs560788815	C	NA	NA	NA	0	NA	NA	NA	0	NA	NA	0
rs183851466	T	NA	NA	NA	0	NA	NA	NA	0	NA	NA	0
rs151152524	C	NA	NA	NA	0	NA	NA	NA	0.004673	NA	NA	0
rs113506821	T	0.02217	0.06522	0.0625	0.009804	0.09091	0.02857	0.04167	0.02336	0.09091	0	0
rs530527676	A	NA	NA	NA	0	NA	NA	NA	0	NA	NA	0
rs550252022	C	NA	NA	NA	0	NA	NA	NA	0	NA	NA	0
rs386638	C	NA	NA	NA	0.07353	NA	NA	NA	0.02336	NA	NA	0
rs148136016	G	NA	NA	NA	0	NA	NA	NA	0	NA	NA	0.00463
rs114848359	T	NA	NA	NA	0	NA	NA	NA	0	NA	NA	0.00463
rs530009764	C	NA	NA	NA	0	NA	NA	NA	0	NA	NA	0

SNP	Allele	Salvador	Shimaa	Shipibo	STU	Tacna	Tallanes	Trujillo	TSI	Tumbes	Uros	YRI
rs371965055	T	NA	NA	NA	0.08333	NA	NA	NA	0	NA	NA	0
rs183984610	A	NA	NA	NA	0	NA	NA	NA	0	NA	NA	0
rs527305819	A	NA	NA	NA	0	NA	NA	NA	0	NA	NA	0
rs181461974	T	NA	NA	NA	0	NA	NA	NA	0	NA	NA	0.00463
rs566457884	T	NA	NA	NA	0	NA	NA	NA	0	NA	NA	0
rs540070564	A	NA	NA	NA	0	NA	NA	NA	0	NA	NA	0
rs563555305	T	NA	NA	NA	0	NA	NA	NA	0	NA	NA	0
rs151189718	T	NA	NA	NA	0	NA	NA	NA	0.004673	NA	NA	0
rs569681994	C	NA	NA	NA	0	NA	NA	NA	0	NA	NA	0
rs143097165	A	NA	NA	NA	0	NA	NA	NA	0	NA	NA	0.009259
rs541200538	G	NA	NA	NA	0	NA	NA	NA	0	NA	NA	0
rs11701576	G	NA	NA	NA	0.1765	NA	NA	NA	0.1262	NA	NA	0.0787
rs17001042	A	NA	NA	NA	0.004902	NA	NA	NA	0.009346	NA	NA	0.1389
rs558449333	T	NA	NA	NA	0	NA	NA	NA	0	NA	NA	0
rs569866330	A	NA	NA	NA	0	NA	NA	NA	0	NA	NA	0
rs573234046	T	NA	NA	NA	0	NA	NA	NA	0	NA	NA	0
rs114363287	T	NA	NA	NA	0	NA	NA	NA	0	NA	NA	0.00463
rs55896064	A	NA	NA	NA	0.07353	NA	NA	NA	0.1355	NA	NA	0.0463
rs547961708	T	NA	NA	NA	0	NA	NA	NA	0	NA	NA	0
rs542712985	G	NA	NA	NA	0	NA	NA	NA	0	NA	NA	0
rs117941520	A	NA	NA	NA	0	NA	NA	NA	0	NA	NA	0
rs73903404	G	NA	NA	NA	0	NA	NA	NA	0	NA	NA	0.03241
rs551817968	T	NA	NA	NA	0	NA	NA	NA	0	NA	NA	0
rs550208798	T	NA	NA	NA	0.004902	NA	NA	NA	0	NA	NA	0
rs542471574	A	NA	NA	NA	0.009804	NA	NA	NA	0	NA	NA	0
rs145841350	T	NA	NA	NA	0	NA	NA	NA	0.004673	NA	NA	0
rs7278627	A	0.07945	0	0	0.004902	0	0	0	0	0	0	0.1991
rs185555838	A	NA	NA	NA	0	NA	NA	NA	0	NA	NA	0.00463
rs374666536	T	NA	NA	NA	0.004902	NA	NA	NA	0	NA	NA	0
rs7278739	A	NA	NA	NA	0.009804	NA	NA	NA	0.004673	NA	NA	0.3102
rs555667242	C	NA	NA	NA	0	NA	NA	NA	0	NA	NA	0
rs7364083	G	NA	NA	NA	0.4461	NA	NA	NA	0.4346	NA	NA	0.1343
rs527284833	G	NA	NA	NA	0	NA	NA	NA	0	NA	NA	0.009259
rs139458208	C	NA	NA	NA	0	NA	NA	NA	0	NA	NA	0.00463
rs139001727	G	NA	NA	NA	0	NA	NA	NA	0	NA	NA	0
rs4290734	G	NA	NA	NA	0.3922	NA	NA	NA	0.486	NA	NA	0.0463
rs2156300	G	NA	NA	NA	0.07353	NA	NA	NA	0.01869	NA	NA	0
rs555328523	T	NA	NA	NA	0	NA	NA	NA	0	NA	NA	0
rs575168295	G	NA	NA	NA	0.004902	NA	NA	NA	0	NA	NA	0
rs145355824	A	NA	NA	NA	0	NA	NA	NA	0	NA	NA	0
rs375827195	G	NA	NA	NA	0	NA	NA	NA	0	NA	NA	0
rs533805020	T	NA	NA	NA	0	NA	NA	NA	0	NA	NA	0
rs536311940	G	NA	NA	NA	0	NA	NA	NA	0	NA	NA	0
rs371046741	A	NA	NA	NA	0	NA	NA	NA	0	NA	NA	0
rs2838042	C	0.1678	0	0	0.3137	0.02273	0.01429	0.1042	0.2243	0.1212	0	0.1713

SNP	Allele	Salvador	Shimaa	Shipibo	STU	Tacna	Tallanes	Trujillo	TSI	Tumbes	Uros	YRI
rs192334131	A	NA	NA	NA	0	NA	NA	NA	0	NA	NA	0
rs557822084	T	NA	NA	NA	0	NA	NA	NA	0	NA	NA	0
rs566208761	C	NA	NA	NA	0	NA	NA	NA	0	NA	NA	0
rs546335233	A	NA	NA	NA	0	NA	NA	NA	0	NA	NA	0
rs184767756	C	NA	NA	NA	0	NA	NA	NA	0	NA	NA	0
rs528259213	A	NA	NA	NA	0	NA	NA	NA	0	NA	NA	0
rs115265507	A	NA	NA	NA	0	NA	NA	NA	0	NA	NA	0.02315
rs200072801	G	NA	NA	NA	0	NA	NA	NA	0	NA	NA	0
rs138765307	T	NA	NA	NA	0	NA	NA	NA	0	NA	NA	0
rs532005855	C	NA	NA	NA	0	NA	NA	NA	0	NA	NA	0
rs2838043	T	NA	NA	NA	0.1275	NA	NA	NA	0.2103	NA	NA	0.04167
rs115596471	A	NA	NA	NA	0	NA	NA	NA	0	NA	NA	0.03241
rs574538362	T	NA	NA	NA	0	NA	NA	NA	0	NA	NA	0
rs190618812	A	NA	NA	NA	0	NA	NA	NA	0	NA	NA	0
rs141583878	A	NA	NA	NA	0	NA	NA	NA	0.004673	NA	NA	0
rs571805265	T	NA	NA	NA	0	NA	NA	NA	0	NA	NA	0
rs371497524	A	NA	NA	NA	0	NA	NA	NA	0	NA	NA	0
rs540908584	T	NA	NA	NA	0	NA	NA	NA	0	NA	NA	0
rs73357642	A	NA	NA	NA	0	NA	NA	NA	0	NA	NA	0.2083
rs192854367	A	NA	NA	NA	0	NA	NA	NA	0	NA	NA	0
rs371180501	G	NA	NA	NA	0	NA	NA	NA	0	NA	NA	0.01389
rs467375	A	0.2226	0.06522	0.0625	0.3725	0.09091	0.04286	0.125	0.4159	0.2273	0	0.09722
rs562045100	A	NA	NA	NA	0.004902	NA	NA	NA	0.004673	NA	NA	0
rs34205539	AT	NA	NA	NA	0.08333	NA	NA	NA	0.1776	NA	NA	0.06019
rs186418926	A	NA	NA	NA	0	NA	NA	NA	0	NA	NA	0
rs539528622	T	NA	NA	NA	0	NA	NA	NA	0	NA	NA	0
rs145292327	G	NA	NA	NA	0	NA	NA	NA	0	NA	NA	0
rs372405355	C	NA	NA	NA	0	NA	NA	NA	0	NA	NA	0
rs544417878	T	NA	NA	NA	0	NA	NA	NA	0	NA	NA	0
rs559363556	A	NA	NA	NA	0	NA	NA	NA	0	NA	NA	0
rs570467504	C	NA	NA	NA	0	NA	NA	NA	0	NA	NA	0
rs148701953	C	NA	NA	NA	0	NA	NA	NA	0	NA	NA	0
rs569988060	A	NA	NA	NA	0	NA	NA	NA	0	NA	NA	0
rs182254633	G	NA	NA	NA	0	NA	NA	NA	0	NA	NA	0.009259
rs563305848	C	NA	NA	NA	0.01471	NA	NA	NA	0	NA	NA	0
rs528477904	C	NA	NA	NA	0	NA	NA	NA	0	NA	NA	0
rs550874821	G	NA	NA	NA	0	NA	NA	NA	0	NA	NA	0
rs73372193	C	NA	NA	NA	0	NA	NA	NA	0.004673	NA	NA	0.2222
rs552607028	A	NA	NA	NA	0	NA	NA	NA	0	NA	NA	0
rs553861191	T	NA	NA	NA	0.004902	NA	NA	NA	0	NA	NA	0
rs147977027	C	NA	NA	NA	0	NA	NA	NA	0	NA	NA	0
rs533445403	T	NA	NA	NA	0	NA	NA	NA	0	NA	NA	0
rs578005531	T	NA	NA	NA	0	NA	NA	NA	0	NA	NA	0
rs140605461	A	NA	NA	NA	0	NA	NA	NA	0	NA	NA	0
rs544023509	A	NA	NA	NA	0	NA	NA	NA	0	NA	NA	0

SNP	Allele	Salvador	Shimaa	Shipibo	STU	Tacna	Tallanes	Trujillo	TSI	Tumbes	Uros	YRI
rs61735792	A	NA	NA	NA	0.004902	NA	NA	NA	0.02804	NA	NA	0
rs568256706	G	NA	NA	NA	0	NA	NA	NA	0	NA	NA	0
rs8131648	T	NA	NA	NA	0.3627	NA	NA	NA	0.2196	NA	NA	0.4028
rs527964326	C	NA	NA	NA	0	NA	NA	NA	0	NA	NA	0
rs191697229	A	NA	NA	NA	0	NA	NA	NA	0	NA	NA	0.00463
rs147359020	A	NA	NA	NA	0.04412	NA	NA	NA	0.009346	NA	NA	0
rs532085624	T	NA	NA	NA	0	NA	NA	NA	0	NA	NA	0
rs577753477	A	NA	NA	NA	0.004902	NA	NA	NA	0	NA	NA	0
rs181091055	C	NA	NA	NA	0	NA	NA	NA	0	NA	NA	0
rs546447704	T	NA	NA	NA	0	NA	NA	NA	0	NA	NA	0
rs575335056	CA	NA	NA	NA	0	NA	NA	NA	0	NA	NA	0
rs536763630	A	NA	NA	NA	0	NA	NA	NA	0	NA	NA	0
rs184500277	T	NA	NA	NA	0	NA	NA	NA	0	NA	NA	0
rs569292495	G	NA	NA	NA	0.004902	NA	NA	NA	0	NA	NA	0
rs73372163	A	NA	NA	NA	0.07843	NA	NA	NA	0.1402	NA	NA	0.2685
rs554443322	C	NA	NA	NA	0	NA	NA	NA	0	NA	NA	0
rs568346732	T	NA	NA	NA	0	NA	NA	NA	0	NA	NA	0
rs114641598	C	NA	NA	NA	0	NA	NA	NA	0	NA	NA	0.01852
rs549459413	A	NA	NA	NA	0	NA	NA	NA	0	NA	NA	0
rs184380117	A	NA	NA	NA	0	NA	NA	NA	0	NA	NA	0
rs576181035	G	NA	NA	NA	0	NA	NA	NA	0	NA	NA	0
rs201679623	C	NA	NA	NA	0	NA	NA	NA	0	NA	NA	0
rs568846685	A	NA	NA	NA	0.004902	NA	NA	NA	0	NA	NA	0
rs548718178	C	NA	NA	NA	0	NA	NA	NA	0	NA	NA	0
rs537584838	G	NA	NA	NA	0	NA	NA	NA	0.004673	NA	NA	0
rs569259137	A	NA	NA	NA	0	NA	NA	NA	0	NA	NA	0
rs367885466	T	NA	NA	NA	0	NA	NA	NA	0	NA	NA	0
rs2410430	A	NA	NA	NA	0.07353	NA	NA	NA	0.01869	NA	NA	0.00463
rs559637785	A	NA	NA	NA	0	NA	NA	NA	0	NA	NA	0
rs573343651	C	NA	NA	NA	0.004902	NA	NA	NA	0	NA	NA	0
rs34769294	CA	NA	NA	NA	0.2304	NA	NA	NA	0.2897	NA	NA	0.1991
rs7364088	A	NA	NA	NA	0.3039	NA	NA	NA	0.257	NA	NA	0.2778
rs551432766	T	NA	NA	NA	0.004902	NA	NA	NA	0	NA	NA	0
rs565299100	G	NA	NA	NA	0	NA	NA	NA	0	NA	NA	0
rs538252674	C	NA	NA	NA	0	NA	NA	NA	0	NA	NA	0
rs368994585	A	NA	NA	NA	0	NA	NA	NA	0	NA	NA	0
rs192709500	A	NA	NA	NA	0	NA	NA	NA	0	NA	NA	0
rs192955773	C	NA	NA	NA	0	NA	NA	NA	0	NA	NA	0.009259
rs371020678	T	NA	NA	NA	0.009804	NA	NA	NA	0	NA	NA	0
rs556435793	A	NA	NA	NA	0	NA	NA	NA	0	NA	NA	0
rs549990870	G	NA	NA	NA	0	NA	NA	NA	0.004673	NA	NA	0
rs146723217	T	NA	NA	NA	0.004902	NA	NA	NA	0	NA	NA	0.05093
rs145738510	C	NA	NA	NA	0	NA	NA	NA	0.004673	NA	NA	0
rs114844880	C	NA	NA	NA	0	NA	NA	NA	0	NA	NA	0.01852
rs370641046	T	NA	NA	NA	0.004902	NA	NA	NA	0	NA	NA	0

SNP	Allele	Salvador	Shimaa	Shipibo	STU	Tacna	Tallanes	Trujillo	TSI	Tumbes	Uros	YRI
rs528452128	GTGTTT	NA	NA	NA	0	NA	NA	NA	0	NA	NA	0.00463
rs11910678	C	0.09663	0	0	0	0	0	0.02083	0	0.01515	0	0.1389
rs140625413	C	NA	NA	NA	0	NA	NA	NA	0	NA	NA	0
rs142444476	T	NA	NA	NA	0	NA	NA	NA	0	NA	NA	0.00463
rs545149163	A	NA	NA	NA	0	NA	NA	NA	0	NA	NA	0
rs149798653	C	NA	NA	NA	0	NA	NA	NA	0	NA	NA	0
rs527416684	A	NA	NA	NA	0.02941	NA	NA	NA	0	NA	NA	0
rs189067157	A	NA	NA	NA	0	NA	NA	NA	0	NA	NA	0
rs527489879	C	NA	NA	NA	0	NA	NA	NA	0	NA	NA	0
rs558824762	T	NA	NA	NA	0	NA	NA	NA	0	NA	NA	0
rs370927492	T	NA	NA	NA	0	NA	NA	NA	0	NA	NA	0
rs1003030	G	NA	NA	NA	0.1765	NA	NA	NA	0.1262	NA	NA	0.0787
rs543908797	A	NA	NA	NA	0	NA	NA	NA	0	NA	NA	0
rs567103273	G	NA	NA	NA	0	NA	NA	NA	0	NA	NA	0
rs565820920	T	NA	NA	NA	0	NA	NA	NA	0	NA	NA	0
rs548463596	G	NA	NA	NA	0.25	NA	NA	NA	0.229	NA	NA	0.3796
rs561570726	T	NA	NA	NA	0	NA	NA	NA	0	NA	NA	0
rs555911696	A	NA	NA	NA	0	NA	NA	NA	0	NA	NA	0.009259
rs561179495	T	NA	NA	NA	0	NA	NA	NA	0	NA	NA	0
rs181972300	A	NA	NA	NA	0	NA	NA	NA	0	NA	NA	0.00463
rs138498737	A	NA	NA	NA	0	NA	NA	NA	0.03738	NA	NA	0
rs565468881	T	NA	NA	NA	0	NA	NA	NA	0	NA	NA	0
rs186734573	T	NA	NA	NA	0	NA	NA	NA	0	NA	NA	0
rs146132480	A	NA	NA	NA	0	NA	NA	NA	0	NA	NA	0.00463
rs559125514	C	NA	NA	NA	0	NA	NA	NA	0	NA	NA	0
rs527499027	T	NA	NA	NA	0	NA	NA	NA	0	NA	NA	0
rs573736906	A	NA	NA	NA	0.02941	NA	NA	NA	0	NA	NA	0
rs573613070	G	NA	NA	NA	0	NA	NA	NA	0	NA	NA	0.00463
rs542898542	T	NA	NA	NA	0	NA	NA	NA	0	NA	NA	0
rs422471	C	0.3774	0.8043	0.5312	0.402	0.7273	0.7571	0.6667	0.271	0.5303	0.625	0.412
rs181058683	A	NA	NA	NA	0	NA	NA	NA	0.004673	NA	NA	0
rs149855493	C	NA	NA	NA	0	NA	NA	NA	0	NA	NA	0.009259
rs191457025	T	NA	NA	NA	0	NA	NA	NA	0	NA	NA	0
rs375760	T	NA	NA	NA	0.2157	NA	NA	NA	0.2897	NA	NA	0.1667
rs148038688	T	NA	NA	NA	0	NA	NA	NA	0	NA	NA	0.009259
rs145728087	A	NA	NA	NA	0	NA	NA	NA	0	NA	NA	0
rs569947342	T	NA	NA	NA	0	NA	NA	NA	0	NA	NA	0
rs562599408	G	NA	NA	NA	0	NA	NA	NA	0	NA	NA	0
rs146252393	A	NA	NA	NA	0	NA	NA	NA	0	NA	NA	0
rs73372182	A	NA	NA	NA	0	NA	NA	NA	0	NA	NA	0.2269
rs566012796	C	NA	NA	NA	0	NA	NA	NA	0	NA	NA	0
rs74423429	A	NA	NA	NA	0	NA	NA	NA	0.01402	NA	NA	0
rs193067129	A	NA	NA	NA	0	NA	NA	NA	0	NA	NA	0
rs572143040	C	NA	NA	NA	0	NA	NA	NA	0	NA	NA	0
rs9984012	T	NA	NA	NA	0.05392	NA	NA	NA	0.1449	NA	NA	0

SNP	Allele	Salvador	Shimaa	Shipibo	STU	Tacna	Tallanes	Trujillo	TSI	Tumbes	Uros	YRI
rs141898436	C	NA	NA	NA	0	NA	NA	NA	0	NA	NA	0.02315
rs558272207	A	NA	NA	NA	0	NA	NA	NA	0	NA	NA	0
rs146681599	A	NA	NA	NA	0	NA	NA	NA	0	NA	NA	0
rs743542	A	0.09931	0.1304	0.4062	0.201	0.1591	0.1857	0.1042	0.03271	0.1364	0.375	0.09722
rs532511763	T	NA	NA	NA	0	NA	NA	NA	0	NA	NA	0
rs149424945	T	NA	NA	NA	0	NA	NA	NA	0	NA	NA	0
rs568605816	A	NA	NA	NA	0	NA	NA	NA	0	NA	NA	0
rs184859933	T	NA	NA	NA	0	NA	NA	NA	0	NA	NA	0
rs530918384	GGTGA	NA	NA	NA	0.009804	NA	NA	NA	0.004673	NA	NA	0
rs28369457	T	NA	NA	NA	0	NA	NA	NA	0	NA	NA	0
rs556192760	A	NA	NA	NA	0	NA	NA	NA	0	NA	NA	0
rs576447507	C	NA	NA	NA	0	NA	NA	NA	0	NA	NA	0
rs546512831	A	NA	NA	NA	0	NA	NA	NA	0	NA	NA	0
rs543066660	A	NA	NA	NA	0	NA	NA	NA	0	NA	NA	0
rs543404937	T	NA	NA	NA	0	NA	NA	NA	0	NA	NA	0
rs180826598	C	NA	NA	NA	0	NA	NA	NA	0	NA	NA	0
rs368878191	T	NA	NA	NA	0	NA	NA	NA	0	NA	NA	0
rs369619813	G	NA	NA	NA	0	NA	NA	NA	0	NA	NA	0
rs112213575	A	NA	NA	NA	0	NA	NA	NA	0	NA	NA	0.01389
rs548725701	A	NA	NA	NA	0	NA	NA	NA	0	NA	NA	0
rs569959837	A	NA	NA	NA	0	NA	NA	NA	0	NA	NA	0
rs554986094	A	NA	NA	NA	0	NA	NA	NA	0	NA	NA	0
rs456142	T	0.2987	NA	NA	0.3627	NA	NA	NA	0.1963	NA	NA	0.3426
rs73357644	T	NA	NA	NA	0	NA	NA	NA	0	NA	NA	0.2083
rs576259678	C	NA	NA	NA	0	NA	NA	NA	0	NA	NA	0
rs140141551	A	NA	NA	NA	0	NA	NA	NA	0.02336	NA	NA	0
rs146564124	T	NA	NA	NA	0	NA	NA	NA	0.03271	NA	NA	0
rs558715322	T	NA	NA	NA	0	NA	NA	NA	0	NA	NA	0
rs116020930	A	NA	NA	NA	0	NA	NA	NA	0	NA	NA	0.00463
rs539333191	T	NA	NA	NA	0	NA	NA	NA	0	NA	NA	0
rs192259532	T	NA	NA	NA	0	NA	NA	NA	0	NA	NA	0
rs531920590	T	NA	NA	NA	0	NA	NA	NA	0	NA	NA	0
rs531300154	C	NA	NA	NA	0	NA	NA	NA	0	NA	NA	0
rs147945120	T	NA	NA	NA	0	NA	NA	NA	0	NA	NA	0
rs147934552	A	NA	NA	NA	0	NA	NA	NA	0	NA	NA	0
rs112980967	A	NA	NA	NA	0	NA	NA	NA	0	NA	NA	0.02778
rs149601802	T	NA	NA	NA	0	NA	NA	NA	0.004673	NA	NA	0
rs3761373	T	0.101	NA	NA	0.1765	NA	NA	NA	0.1262	NA	NA	0.0787
rs545333396	C	NA	NA	NA	0	NA	NA	NA	0	NA	NA	0
rs574683527	A	NA	NA	NA	0	NA	NA	NA	0	NA	NA	0
rs189431131	C	NA	NA	NA	0	NA	NA	NA	0	NA	NA	0
rs183147330	A	NA	NA	NA	0	NA	NA	NA	0	NA	NA	0
rs417443	A	NA	NA	NA	0.07353	NA	NA	NA	0.01869	NA	NA	0
rs564403172	T	NA	NA	NA	0	NA	NA	NA	0	NA	NA	0
rs8134216	T	0.605	0.1522	0.3125	0.6324	0.1591	0.08571	0.3125	0.7804	0.3788	0	0.5139

SNP	Allele	Salvador	Shimaa	Shipibo	STU	Tacna	Tallanes	Trujillo	TSI	Tumbes	Uros	YRI
rs191441931	T	NA	NA	NA	0	NA	NA	NA	0	NA	NA	0
rs567188556	T	NA	NA	NA	0	NA	NA	NA	0	NA	NA	0
rs187662898	T	NA	NA	NA	0	NA	NA	NA	0	NA	NA	0
rs114911304	A	NA	NA	NA	0	NA	NA	NA	0	NA	NA	0.02315
rs558858943	C	NA	NA	NA	0	NA	NA	NA	0	NA	NA	0
rs541396507	C	NA	NA	NA	0	NA	NA	NA	0	NA	NA	0
rs565142599	T	NA	NA	NA	0	NA	NA	NA	0	NA	NA	0
rs146605032	A	NA	NA	NA	0	NA	NA	NA	0	NA	NA	0
rs553673225	G	NA	NA	NA	0	NA	NA	NA	0	NA	NA	0
rs113034290	T	NA	NA	NA	0	NA	NA	NA	0.004673	NA	NA	0
rs150554820	T	NA	NA	NA	NA	NA	NA	NA	NA	NA	NA	NA
rs544880781	A	NA	NA	NA	0	NA	NA	NA	0	NA	NA	0
rs6517669	A	0.5661	0.97826	0.96875	0.6765	0.93182	1	0.8333	0.7523	0.90909	1	0.3843
rs550238535	A	NA	NA	NA	0	NA	NA	NA	0	NA	NA	0.00463
rs455922	T	NA	NA	NA	0.07353	NA	NA	NA	0.01402	NA	NA	0
rs374203194	A	NA	NA	NA	0	NA	NA	NA	0	NA	NA	0
rs111671182	G	NA	NA	NA	0	NA	NA	NA	0	NA	NA	0.00463
rs181044447	A	NA	NA	NA	0	NA	NA	NA	0	NA	NA	0
rs573737830	A	NA	NA	NA	0	NA	NA	NA	0.004673	NA	NA	0
rs181778800	A	NA	NA	NA	0	NA	NA	NA	0	NA	NA	0
rs577217013	C	NA	NA	NA	0	NA	NA	NA	0	NA	NA	0
rs563948676	A	NA	NA	NA	0	NA	NA	NA	0	NA	NA	0
rs141888586	T	NA	NA	NA	0	NA	NA	NA	0	NA	NA	0.1481
rs146797606	C	NA	NA	NA	0	NA	NA	NA	0	NA	NA	0.009259
rs544037190	C	NA	NA	NA	0	NA	NA	NA	0	NA	NA	0
rs535041396	T	NA	NA	NA	0	NA	NA	NA	0	NA	NA	0
rs534591508	T	NA	NA	NA	0.004902	NA	NA	NA	0	NA	NA	0
rs547758146	A	NA	NA	NA	0	NA	NA	NA	0	NA	NA	0
rs529424146	A	NA	NA	NA	0	NA	NA	NA	0	NA	NA	0
rs563946938	T	NA	NA	NA	0	NA	NA	NA	0	NA	NA	0
rs545056304	A	NA	NA	NA	0	NA	NA	NA	0	NA	NA	0
rs12481984	C	NA	NA	NA	0.2647	NA	NA	NA	0.4393	NA	NA	0.3935
rs538226704	T	NA	NA	NA	0	NA	NA	NA	0	NA	NA	0
rs548678297	G	NA	NA	NA	0.004902	NA	NA	NA	0	NA	NA	0
rs149676870	T	NA	NA	NA	0	NA	NA	NA	0	NA	NA	0
rs78217567	C	NA	NA	NA	0.004902	NA	NA	NA	0.03738	NA	NA	0
rs558791980	A	NA	NA	NA	0	NA	NA	NA	0	NA	NA	0
rs34256269	A	NA	NA	NA	0.05882	NA	NA	NA	0.1449	NA	NA	0
rs74749793	T	0.1016	0	0	0.1716	0	0	0	0.1121	0.04545	0	0.07407
rs193253965	C	NA	NA	NA	0	NA	NA	NA	0	NA	NA	0
rs373622147	C	NA	NA	NA	0.009804	NA	NA	NA	0	NA	NA	0
rs559691670	A	NA	NA	NA	0	NA	NA	NA	0	NA	NA	0
rs534317621	A	NA	NA	NA	0	NA	NA	NA	0	NA	NA	0
rs539981354	G	NA	NA	NA	0	NA	NA	NA	0	NA	NA	0
rs115266855	G	NA	NA	NA	0	NA	NA	NA	0	NA	NA	0.03704

SNP	Allele	Salvador	Shimaa	Shipibo	STU	Tacna	Tallanes	Trujillo	TSI	Tumbes	Uros	YRI
rs149109132	A	NA	NA	NA	0	NA	NA	NA	0.004673	NA	NA	0.009259
rs557656729	A	NA	NA	NA	0	NA	NA	NA	0	NA	NA	0
rs57161767	A	NA	NA	NA	0	NA	NA	NA	0	NA	NA	0.02778
rs574708698	T	NA	NA	NA	0	NA	NA	NA	0	NA	NA	0
rs4303795	G	NA	NA	NA	0.2745	NA	NA	NA	0.4439	NA	NA	0.412
rs145662876	T	NA	NA	NA	0	NA	NA	NA	0	NA	NA	0
rs566146941	A	NA	NA	NA	0	NA	NA	NA	0	NA	NA	0
rs563595766	T	NA	NA	NA	0	NA	NA	NA	0	NA	NA	0
rs572507505	T	NA	NA	NA	0	NA	NA	NA	0	NA	NA	0
rs557606231	G	NA	NA	NA	0	NA	NA	NA	0	NA	NA	0.00463
rs11911394	T	0.5732	0.97826	0.96875	0.6765	0.93182	1	0.8333	0.7523	0.90909	1	0.3935
rs76973757	A	NA	NA	NA	0	NA	NA	NA	0	NA	NA	0.0463
rs573484758	G	NA	NA	NA	0	NA	NA	NA	0	NA	NA	0
rs530471976	T	NA	NA	NA	0	NA	NA	NA	0	NA	NA	0
rs372286621	A	NA	NA	NA	0	NA	NA	NA	0	NA	NA	0
rs4818239	C	NA	NA	NA	0.4118	NA	NA	NA	0.4766	NA	NA	0.2361
rs186484871	C	NA	NA	NA	0	NA	NA	NA	0	NA	NA	0
rs542575868	A	NA	NA	NA	0	NA	NA	NA	0	NA	NA	0
rs111572592	A	NA	NA	NA	0	NA	NA	NA	0.009346	NA	NA	0
rs144576889	G	NA	NA	NA	0	NA	NA	NA	0	NA	NA	0.009259
rs572265353	A	NA	NA	NA	0	NA	NA	NA	0	NA	NA	0
rs191368876	C	NA	NA	NA	0	NA	NA	NA	0	NA	NA	0
rs535472251	CAAAAAAAAAA	NA	NA	NA	0.2451	NA	NA	NA	0.3692	NA	NA	0.3009
rs141788162	A	NA	NA	NA	0	NA	NA	NA	0.004673	NA	NA	0
rs56695953	A	NA	NA	NA	0.1275	NA	NA	NA	0.2103	NA	NA	0.04167
rs548680244	T	NA	NA	NA	0	NA	NA	NA	0	NA	NA	0
rs430915	A	0.317	0.91304	0.7812	0.4314	0.7727	0.8286	0.6458	0.4673	0.7121	0.625	0.1065
rs564795317	A	NA	NA	NA	0	NA	NA	NA	0	NA	NA	0
rs577731955	C	NA	NA	NA	0	NA	NA	NA	0	NA	NA	0
rs369342013	A	NA	NA	NA	0	NA	NA	NA	0	NA	NA	0
rs542402063	A	NA	NA	NA	0	NA	NA	NA	0	NA	NA	0
rs556381293	C	NA	NA	NA	0	NA	NA	NA	0	NA	NA	0.00463
rs534924497	T	NA	NA	NA	0	NA	NA	NA	0	NA	NA	0
rs554462605	A	NA	NA	NA	0	NA	NA	NA	0	NA	NA	0
rs80027429	A	NA	NA	NA	0.009804	NA	NA	NA	0.02336	NA	NA	0
rs561192574	T	NA	NA	NA	0	NA	NA	NA	0	NA	NA	0
rs455281	G	NA	NA	NA	0.3039	NA	NA	NA	0.0514	NA	NA	0.2269
rs9975782	A	NA	NA	NA	0	NA	NA	NA	0	NA	NA	0
rs569362310	G	NA	NA	NA	0	NA	NA	NA	0	NA	NA	0
rs536278410	C	NA	NA	NA	0	NA	NA	NA	0	NA	NA	0
rs415918	C	NA	NA	NA	0.3971	NA	NA	NA	0.2523	NA	NA	0.4074
rs554890599	A	NA	NA	NA	0	NA	NA	NA	0	NA	NA	0
rs530721239	G	NA	NA	NA	0	NA	NA	NA	0	NA	NA	0
rs552589240	G	NA	NA	NA	0	NA	NA	NA	0	NA	NA	0
rs545504807	C	NA	NA	NA	0	NA	NA	NA	0	NA	NA	0

SNP	Allele	Salvador	Shimaa	Shipibo	STU	Tacna	Tallanes	Trujillo	TSI	Tumbes	Uros	YRI
rs28403625	A	NA	NA	NA	0	NA	NA	NA	0	NA	NA	0
rs532159311	C	NA	NA	NA	0	NA	NA	NA	0	NA	NA	0.00463
rs543578752	G	NA	NA	NA	0	NA	NA	NA	0	NA	NA	0
rs576215861	A	NA	NA	NA	0	NA	NA	NA	0	NA	NA	0
rs199824558	A	NA	NA	NA	0.004902	NA	NA	NA	0	NA	NA	0
rs562000328	C	NA	NA	NA	0	NA	NA	NA	0	NA	NA	0
rs559698510	T	NA	NA	NA	0	NA	NA	NA	0	NA	NA	0
rs75168613	A	NA	NA	NA	0.004902	NA	NA	NA	0	NA	NA	0.1296
rs543344542	T	NA	NA	NA	0	NA	NA	NA	0	NA	NA	0
rs570889046	A	NA	NA	NA	0	NA	NA	NA	0	NA	NA	0.00463
rs548267325	A	NA	NA	NA	0	NA	NA	NA	0	NA	NA	0
rs35899679	A	NA	NA	NA	0.3676	NA	NA	NA	0.4439	NA	NA	0.1065
rs561575371	A	NA	NA	NA	0	NA	NA	NA	0	NA	NA	0
rs577554253	A	NA	NA	NA	0	NA	NA	NA	0	NA	NA	0
rs535407146	A	NA	NA	NA	0.009804	NA	NA	NA	0	NA	NA	0
rs149024920	G	NA	NA	NA	0	NA	NA	NA	0	NA	NA	0.00463
rs112209215	A	NA	NA	NA	0	NA	NA	NA	0	NA	NA	0.01389
rs183405398	G	NA	NA	NA	0	NA	NA	NA	0	NA	NA	0
rs374510753	T	NA	NA	NA	0	NA	NA	NA	0	NA	NA	0
rs75200570	C	NA	NA	NA	0.03431	NA	NA	NA	0.01402	NA	NA	0.0787
rs149275684	T	NA	NA	NA	0	NA	NA	NA	0	NA	NA	0
rs547544037	A	NA	NA	NA	0.004902	NA	NA	NA	0	NA	NA	0
rs553755767	A	NA	NA	NA	0.004902	NA	NA	NA	0	NA	NA	0
rs76855393	T	NA	NA	NA	0	NA	NA	NA	0	NA	NA	0
rs118028230	C	NA	NA	NA	NA	NA	NA	NA	NA	NA	NA	NA
rs184764113	A	NA	NA	NA	0	NA	NA	NA	0	NA	NA	0
rs541351488	A	NA	NA	NA	0.009804	NA	NA	NA	0	NA	NA	0
rs185078457	T	NA	NA	NA	0	NA	NA	NA	0	NA	NA	0
rs542961770	T	NA	NA	NA	0	NA	NA	NA	0	NA	NA	0
rs562131885	A	NA	NA	NA	0	NA	NA	NA	0	NA	NA	0.009259
rs536399456	A	NA	NA	NA	0	NA	NA	NA	0	NA	NA	0
rs547523412	A	NA	NA	NA	0	NA	NA	NA	0	NA	NA	0
rs534034788	A	NA	NA	NA	0	NA	NA	NA	0	NA	NA	0
rs544784789	G	NA	NA	NA	0	NA	NA	NA	0	NA	NA	0
rs540987630	T	NA	NA	NA	0	NA	NA	NA	0	NA	NA	0
rs141478137	G	NA	NA	NA	0	NA	NA	NA	0	NA	NA	0
rs5844077	G	NA	NA	NA	0.2206	NA	NA	NA	0.2196	NA	NA	0.08333
rs551681725	G	NA	NA	NA	0	NA	NA	NA	0	NA	NA	0
rs548592256	C	NA	NA	NA	0	NA	NA	NA	0	NA	NA	0
rs568689321	A	NA	NA	NA	0	NA	NA	NA	0	NA	NA	0
rs555302570	A	NA	NA	NA	0.004902	NA	NA	NA	0	NA	NA	0
rs537212420	G	NA	NA	NA	0	NA	NA	NA	0	NA	NA	0
rs547186777	C	NA	NA	NA	0	NA	NA	NA	0	NA	NA	0
rs532627816	A	NA	NA	NA	0	NA	NA	NA	0	NA	NA	0
rs150314077	A	NA	NA	NA	0	NA	NA	NA	0	NA	NA	0.009259

SNP	Allele	Salvador	Shimaa	Shipibo	STU	Tacna	Tallanes	Trujillo	TSI	Tumbes	Uros	YRI
rs544983081	A	NA	NA	NA	0	NA	NA	NA	0	NA	NA	0
rs140121827	T	NA	NA	NA	0	NA	NA	NA	0	NA	NA	0
rs74605993	T	NA	NA	NA	0	NA	NA	NA	0	NA	NA	0.02778
rs7279603	C	0.4244	0.02174	0.03125	0.2598	0.06818	0.01429	0.1667	0.3458	0.1212	0	0.5833
rs535611212	G	NA	NA	NA	0	NA	NA	NA	0.004673	NA	NA	0.009259
rs180792570	T	NA	NA	NA	0	NA	NA	NA	0	NA	NA	0
rs180784757	A	NA	NA	NA	0.004902	NA	NA	NA	0	NA	NA	0.009259
rs532756922	A	NA	NA	NA	0	NA	NA	NA	0	NA	NA	0
rs138651919	A	NA	NA	NA	0	NA	NA	NA	0	NA	NA	0
rs549212787	G	NA	NA	NA	0.004902	NA	NA	NA	0	NA	NA	0
rs538863731	C	NA	NA	NA	0	NA	NA	NA	0	NA	NA	0
rs538803792	T	NA	NA	NA	0	NA	NA	NA	0	NA	NA	0
rs574890845	A	NA	NA	NA	0	NA	NA	NA	0	NA	NA	0
rs546165791	T	NA	NA	NA	0	NA	NA	NA	0	NA	NA	0
rs554530868	G	NA	NA	NA	0.02451	NA	NA	NA	0.04206	NA	NA	0
rs79512425	C	NA	NA	NA	0	NA	NA	NA	0	NA	NA	0
rs542395007	T	NA	NA	NA	0	NA	NA	NA	0.004673	NA	NA	0
rs556249043	A	NA	NA	NA	0	NA	NA	NA	0	NA	NA	0
rs529550811	A	NA	NA	NA	0	NA	NA	NA	0	NA	NA	0.00463
rs533931558	A	NA	NA	NA	0	NA	NA	NA	0	NA	NA	0
rs565902164	AACC	NA	NA	NA	0.01471	NA	NA	NA	0	NA	NA	0
rs530084541	G	NA	NA	NA	0	NA	NA	NA	0	NA	NA	0
rs549593213	T	NA	NA	NA	0	NA	NA	NA	0	NA	NA	0
rs189570078	C	NA	NA	NA	0	NA	NA	NA	0	NA	NA	0
rs190682490	T	NA	NA	NA	0	NA	NA	NA	0	NA	NA	0
rs577570888	T	NA	NA	NA	0	NA	NA	NA	0	NA	NA	0
rs376403654	A	NA	NA	NA	0	NA	NA	NA	0	NA	NA	0
rs576016640	T	NA	NA	NA	0	NA	NA	NA	0	NA	NA	0
rs567552202	C	NA	NA	NA	0	NA	NA	NA	0	NA	NA	0
rs140793040	A	NA	NA	NA	0	NA	NA	NA	0	NA	NA	0
rs146445857	TGCC	NA	NA	NA	0	NA	NA	NA	0	NA	NA	0
rs547401659	T	NA	NA	NA	0	NA	NA	NA	0.009346	NA	NA	0
rs571396442	T	NA	NA	NA	0	NA	NA	NA	0	NA	NA	0
rs185406661	G	NA	NA	NA	0	NA	NA	NA	0	NA	NA	0
rs2070789	T	NA	NA	NA	0.2843	NA	NA	NA	0.2243	NA	NA	0.3148
rs7277080	T	NA	NA	NA	0.2549	NA	NA	NA	0.4112	NA	NA	0.4167
rs183385443	A	NA	NA	NA	0	NA	NA	NA	0	NA	NA	0
rs181592444	T	NA	NA	NA	0	NA	NA	NA	0	NA	NA	0
rs189832305	T	NA	NA	NA	0	NA	NA	NA	0	NA	NA	0
rs734056	A	0.3205	0.1304	0.2812	0.3971	0.09091	0.07143	0.1458	0.486	0.3333	0	0.1944
rs561819256	G	NA	NA	NA	0	NA	NA	NA	0	NA	NA	0
rs2070786	C	0.3536	0.02174	0.03125	0.2549	0.09091	0.01429	0.1667	0.3458	0.1212	0	0.3981
rs541155775	C	NA	NA	NA	0	NA	NA	NA	0	NA	NA	0
rs544315388	A	NA	NA	NA	0	NA	NA	NA	0	NA	NA	0
rs191763249	G	NA	NA	NA	0	NA	NA	NA	0	NA	NA	0

SNP	Allele	Salvador	Shimaa	Shipibo	STU	Tacna	Tallanes	Trujillo	TSI	Tumbes	Uros	YRI
rs137962614	T	NA	NA	NA	0.004902	NA	NA	NA	0.009346	NA	NA	0
rs464431	A	NA	NA	NA	0.06863	NA	NA	NA	0.01402	NA	NA	0.00463
rs147054075	A	NA	NA	NA	0.004902	NA	NA	NA	0	NA	NA	0
rs190516010	A	NA	NA	NA	0	NA	NA	NA	0	NA	NA	0
rs542265380	T	NA	NA	NA	0	NA	NA	NA	0	NA	NA	0
rs188263047	A	NA	NA	NA	0.004902	NA	NA	NA	0	NA	NA	0.009259
rs143148203	G	NA	NA	NA	0	NA	NA	NA	0	NA	NA	0.00463
rs557853844	A	NA	NA	NA	0	NA	NA	NA	0	NA	NA	0
rs571275590	G	NA	NA	NA	0	NA	NA	NA	0	NA	NA	0
rs562173027	C	NA	NA	NA	0	NA	NA	NA	0	NA	NA	0
rs530253044	T	NA	NA	NA	0	NA	NA	NA	0	NA	NA	0
rs532694622	C	NA	NA	NA	0.004902	NA	NA	NA	0	NA	NA	0
rs75756279	T	NA	NA	NA	0.009804	NA	NA	NA	0.02336	NA	NA	0
rs570835062	T	NA	NA	NA	0	NA	NA	NA	0	NA	NA	0
rs530942388	G	NA	NA	NA	0	NA	NA	NA	0	NA	NA	0
rs575313753	G	NA	NA	NA	0	NA	NA	NA	0	NA	NA	0
rs537370123	T	NA	NA	NA	0	NA	NA	NA	0	NA	NA	0
rs185946013	A	NA	NA	NA	0	NA	NA	NA	0	NA	NA	0
rs142518005	T	NA	NA	NA	0	NA	NA	NA	0	NA	NA	0.00463
rs558104085	A	NA	NA	NA	0	NA	NA	NA	0	NA	NA	0
rs144800717	T	NA	NA	NA	0	NA	NA	NA	0	NA	NA	0
rs57474639	T	0.07907	0	0	0.07353	0.02273	0	0.04167	0.1355	0.0625	0	0.05556
rs538139969	T	NA	NA	NA	0	NA	NA	NA	0	NA	NA	0
rs567302726	A	NA	NA	NA	0.02451	NA	NA	NA	0	NA	NA	0
rs187052796	T	NA	NA	NA	0	NA	NA	NA	0	NA	NA	0.009259
rs551164492	G	NA	NA	NA	0.009804	NA	NA	NA	0.004673	NA	NA	0
rs143562195	A	NA	NA	NA	0	NA	NA	NA	0	NA	NA	0.00463
rs11088550	A	NA	NA	NA	0.009804	NA	NA	NA	0.07944	NA	NA	0
rs151338439	A	NA	NA	NA	0	NA	NA	NA	0	NA	NA	0
rs185726773	A	NA	NA	NA	0	NA	NA	NA	0	NA	NA	0.00463
rs531410451	A	NA	NA	NA	0	NA	NA	NA	0	NA	NA	0
rs2257202	C	NA	NA	NA	0.2304	NA	NA	NA	0.285	NA	NA	0.162
rs117827300	A	NA	NA	NA	0	NA	NA	NA	0	NA	NA	0
rs187208295	C	NA	NA	NA	0	NA	NA	NA	0	NA	NA	0
rs61735793	A	0.003438	0	0	0	0	0	0	0.004673	0	0	0
rs143712818	T	NA	NA	NA	0	NA	NA	NA	0	NA	NA	0.009259
rs542575245	T	NA	NA	NA	0	NA	NA	NA	0	NA	NA	0
rs551797163	C	NA	NA	NA	0	NA	NA	NA	0	NA	NA	0
rs571050655	C	NA	NA	NA	0	NA	NA	NA	0	NA	NA	0
rs34624090	AC	NA	NA	NA	0.3775	NA	NA	NA	0.4159	NA	NA	0.1019
rs147099383	C	NA	NA	NA	0.1814	NA	NA	NA	0.03271	NA	NA	0.02778
rs58146697	C	0.143	0	0	0.07353	0	0	0.04167	0.009346	0.01515	0	0.2269
rs139700775	C	NA	NA	NA	0	NA	NA	NA	0	NA	NA	0
rs560183935	C	NA	NA	NA	0	NA	NA	NA	0	NA	NA	0
rs182330267	A	NA	NA	NA	0	NA	NA	NA	0	NA	NA	0.00463

SNP	Allele	Salvador	Shimaa	Shipibo	STU	Tacna	Tallanes	Trujillo	TSI	Tumbes	Uros	YRI
rs565879862	C	NA	NA	NA	0	NA	NA	NA	0	NA	NA	0
rs188500480	T	NA	NA	NA	0	NA	NA	NA	0	NA	NA	0
rs533556786	G	NA	NA	NA	0.004902	NA	NA	NA	0	NA	NA	0
rs551106657	T	NA	NA	NA	0	NA	NA	NA	0	NA	NA	0
rs116606027	A	NA	NA	NA	0	NA	NA	NA	0	NA	NA	0.009259
rs573715927	A	NA	NA	NA	0.004902	NA	NA	NA	0	NA	NA	0
rs145026350	T	NA	NA	NA	0	NA	NA	NA	0	NA	NA	0
rs915823	C	0.1298	0	0	0.1225	0.02273	0	0.04167	0.1682	0.04545	0	0.1157
rs188495261	A	NA	NA	NA	0	NA	NA	NA	0	NA	NA	0
rs563800355	T	NA	NA	NA	0	NA	NA	NA	0	NA	NA	0
rs143060022	A	NA	NA	NA	0	NA	NA	NA	0.009346	NA	NA	0
rs527811791	C	NA	NA	NA	0	NA	NA	NA	0	NA	NA	0
rs554692214	C	NA	NA	NA	0	NA	NA	NA	0	NA	NA	0
rs8129582	A	NA	NA	NA	0	NA	NA	NA	0	NA	NA	0
rs532513568	C	NA	NA	NA	0	NA	NA	NA	0	NA	NA	0
rs537357445	C	NA	NA	NA	0	NA	NA	NA	0	NA	NA	0
rs538591883	C	NA	NA	NA	0.004902	NA	NA	NA	0	NA	NA	0
rs535599066	T	NA	NA	NA	0.01961	NA	NA	NA	0	NA	NA	0
rs7275220	G	0.4213	NA	NA	0.3137	NA	NA	NA	0.271	NA	NA	0.4583
rs199636550	G	NA	NA	NA	0	NA	NA	NA	0	NA	NA	0
rs117696554	A	NA	NA	NA	0	NA	NA	NA	0.04673	NA	NA	0
rs137871202	C	NA	NA	NA	0	NA	NA	NA	0	NA	NA	0.00463
rs555056776	A	NA	NA	NA	0	NA	NA	NA	0	NA	NA	0
rs553934496	T	NA	NA	NA	0	NA	NA	NA	0	NA	NA	0
rs547701911	C	NA	NA	NA	0	NA	NA	NA	0	NA	NA	0.00463
rs187460831	A	NA	NA	NA	0	NA	NA	NA	0	NA	NA	0
rs422761	A	NA	NA	NA	0.2402	NA	NA	NA	0.009346	NA	NA	0.2546
rs186422083	C	NA	NA	NA	0	NA	NA	NA	0	NA	NA	0
rs544946928	T	NA	NA	NA	0.01961	NA	NA	NA	0.004673	NA	NA	0
rs542577849	A	NA	NA	NA	0	NA	NA	NA	0	NA	NA	0
rs561308071	T	NA	NA	NA	0	NA	NA	NA	0	NA	NA	0
rs184146774	G	NA	NA	NA	0	NA	NA	NA	0	NA	NA	0
rs181446489	C	NA	NA	NA	0	NA	NA	NA	0	NA	NA	0
rs543518445	T	NA	NA	NA	0.004902	NA	NA	NA	0	NA	NA	0
rs566732470	A	NA	NA	NA	0	NA	NA	NA	0	NA	NA	0
rs533571483	A	NA	NA	NA	0	NA	NA	NA	0	NA	NA	0
rs531148133	A	NA	NA	NA	0	NA	NA	NA	0	NA	NA	0
rs113288437	G	NA	NA	NA	0.004902	NA	NA	NA	0	NA	NA	0.1343
rs115968373	A	NA	NA	NA	0	NA	NA	NA	0	NA	NA	0.00463
rs140715097	T	NA	NA	NA	0	NA	NA	NA	0	NA	NA	0
rs376897988	T	NA	NA	NA	0	NA	NA	NA	0	NA	NA	0
rs574759417	A	NA	NA	NA	0	NA	NA	NA	0	NA	NA	0
rs528568210	A	NA	NA	NA	0	NA	NA	NA	0	NA	NA	0
rs540209049	C	NA	NA	NA	0	NA	NA	NA	0	NA	NA	0
rs191595416	A	NA	NA	NA	0	NA	NA	NA	0	NA	NA	0

SNP	Allele	Salvador	Shimaa	Shipibo	STU	Tacna	Tallanes	Trujillo	TSI	Tumbes	Uros	YRI
rs66492316	C	NA	NA	NA	0.2745	NA	NA	NA	0.4393	NA	NA	0.412
rs8126497	A	NA	NA	NA	0.1225	NA	NA	NA	0.2103	NA	NA	0
rs531884709	A	NA	NA	NA	0	NA	NA	NA	0	NA	NA	0
rs547558669	G	NA	NA	NA	0	NA	NA	NA	0	NA	NA	0
rs55704664	T	NA	NA	NA	0.1275	NA	NA	NA	0.2103	NA	NA	0
rs546388820	T	NA	NA	NA	0	NA	NA	NA	0	NA	NA	0
rs142103907	G	NA	NA	NA	0	NA	NA	NA	0	NA	NA	0.009259
rs117656646	C	NA	NA	NA	0	NA	NA	NA	0.009346	NA	NA	0
rs139258152	T	NA	NA	NA	0	NA	NA	NA	0	NA	NA	0.00463
rs150382508	C	NA	NA	NA	0	NA	NA	NA	0	NA	NA	0.009259
rs544308006	T	NA	NA	NA	0	NA	NA	NA	0	NA	NA	0
rs551100726	T	NA	NA	NA	0.009804	NA	NA	NA	0	NA	NA	0
rs527435310	A	NA	NA	NA	0	NA	NA	NA	0	NA	NA	0
rs552725417	A	NA	NA	NA	0	NA	NA	NA	0	NA	NA	0
rs549038772	T	NA	NA	NA	0	NA	NA	NA	0	NA	NA	0
rs562574628	G	NA	NA	NA	0	NA	NA	NA	0	NA	NA	0
rs8129713	C	0.1283	0.06522	0.25	0.1324	0.1136	0.2	0.1042	0.2103	0.197	0.3438	0.04167
rs183385542	C	NA	NA	NA	0	NA	NA	NA	0	NA	NA	0
rs553596811	G	NA	NA	NA	0.004902	NA	NA	NA	0	NA	NA	0
rs150473664	C	NA	NA	NA	0	NA	NA	NA	0	NA	NA	0
rs146120690	A	NA	NA	NA	0	NA	NA	NA	0	NA	NA	0
rs527261679	A	NA	NA	NA	0	NA	NA	NA	0	NA	NA	0
rs570544092	T	NA	NA	NA	0	NA	NA	NA	0	NA	NA	0
rs392370	C	NA	NA	NA	0.2304	NA	NA	NA	0.2897	NA	NA	0.3935
rs150875127	C	NA	NA	NA	0.6324	NA	NA	NA	0.7664	NA	NA	0.5231
rs536039173	T	NA	NA	NA	0.01471	NA	NA	NA	0	NA	NA	0
rs192019778	A	NA	NA	NA	0	NA	NA	NA	0	NA	NA	0
SNP	A1	MAF	MAF	MAF	MAF	MAF	MAF	MAF	MAF	MAF	MAF	MAF
rs537728755	A	NA	NA	NA	0	NA	NA	NA	0	NA	NA	0.00463
rs55964536	T	NA	NA	NA	0.3775	NA	NA	NA	0.4766	NA	NA	0.1111
rs373311004	C	NA	NA	NA	0	NA	NA	NA	0	NA	NA	0
rs575968857	T	NA	NA	NA	0	NA	NA	NA	0	NA	NA	0
rs548402221	G	NA	NA	NA	0	NA	NA	NA	0.02804	NA	NA	0
rs140037718	C	NA	NA	NA	0	NA	NA	NA	0	NA	NA	0
rs75373173	C	0.03629	0	0	0.01961	0.02273	0.02857	0	0.0514	0.0303	0	0
rs568619174	C	NA	NA	NA	0	NA	NA	NA	0	NA	NA	0
rs187175193	T	NA	NA	NA	0	NA	NA	NA	0	NA	NA	0
rs193122175	T	NA	NA	NA	0	NA	NA	NA	0.004673	NA	NA	0
rs117898838	A	NA	NA	NA	0	NA	NA	NA	0	NA	NA	0.00463
rs537989379	A	NA	NA	NA	0	NA	NA	NA	0	NA	NA	0
rs570302987	A	NA	NA	NA	0	NA	NA	NA	0	NA	NA	0
rs190970011	T	NA	NA	NA	0	NA	NA	NA	0	NA	NA	0
rs556994660	C	NA	NA	NA	0	NA	NA	NA	0	NA	NA	0
rs527380714	C	NA	NA	NA	0	NA	NA	NA	0	NA	NA	0
rs536026892	T	NA	NA	NA	0	NA	NA	NA	0	NA	NA	0

SNP	Allele	Salvador	Shimaa	Shipibo	STU	Tacna	Tallanes	Trujillo	TSI	Tumbes	Uros	YRI
rs535103772	T	NA	NA	NA	0	NA	NA	NA	0	NA	NA	0
rs191587330	C	NA	NA	NA	0	NA	NA	NA	0	NA	NA	0
rs553617111	T	NA	NA	NA	0	NA	NA	NA	0	NA	NA	0
rs182838808	A	NA	NA	NA	0	NA	NA	NA	0	NA	NA	0
rs78503214	T	NA	NA	NA	0	NA	NA	NA	0	NA	NA	0.02778
rs142425263	A	NA	NA	NA	0	NA	NA	NA	0	NA	NA	0
rs573213706	A	NA	NA	NA	0	NA	NA	NA	0	NA	NA	0
rs73357663	C	NA	NA	NA	0	NA	NA	NA	0	NA	NA	0.02778
rs563670115	C	NA	NA	NA	0	NA	NA	NA	0	NA	NA	0
rs111220509	G	NA	NA	NA	0.6275	NA	NA	NA	0.7757	NA	NA	0.5
rs186605876	T	NA	NA	NA	0	NA	NA	NA	0	NA	NA	0
rs373611430	G	NA	NA	NA	0	NA	NA	NA	0	NA	NA	0
rs2094881	C	0.597	0.08696	0.09375	0.6373	0.1591	0.07143	0.3125	0.7804	0.3485	0	0.5093
rs73372166	A	NA	NA	NA	0.07843	NA	NA	NA	0.1402	NA	NA	0.2685
rs76135088	G	0.03858	0	0	0.004902	0	0	0	0	0	0	0.09259
rs548979551	G	NA	NA	NA	0	NA	NA	NA	0	NA	NA	0
rs540046047	C	NA	NA	NA	0	NA	NA	NA	0	NA	NA	0
rs2410429	A	NA	NA	NA	0.3088	NA	NA	NA	0.2991	NA	NA	0.2639
rs532950488	A	NA	NA	NA	0.004902	NA	NA	NA	0	NA	NA	0
rs535531364	C	NA	NA	NA	0	NA	NA	NA	0	NA	NA	0.009259
rs550091217	T	NA	NA	NA	0	NA	NA	NA	0	NA	NA	0
rs62217527	T	NA	NA	NA	0.04412	NA	NA	NA	0.1589	NA	NA	0.01389
rs80275470	A	NA	NA	NA	0	NA	NA	NA	0.02804	NA	NA	0
rs545165511	A	NA	NA	NA	0	NA	NA	NA	0	NA	NA	0
rs144318842	A	NA	NA	NA	0	NA	NA	NA	0	NA	NA	0.01389
rs200395836	T	NA	NA	NA	0	NA	NA	NA	0	NA	NA	0
rs548552862	C	NA	NA	NA	0	NA	NA	NA	0	NA	NA	0.00463
rs577632959	A	NA	NA	NA	0	NA	NA	NA	0	NA	NA	0
rs567258163	A	NA	NA	NA	0.02941	NA	NA	NA	0	NA	NA	0
rs561789442	A	NA	NA	NA	0	NA	NA	NA	0	NA	NA	0
rs538588854	A	NA	NA	NA	0	NA	NA	NA	0	NA	NA	0
rs115429336	A	NA	NA	NA	0	NA	NA	NA	0	NA	NA	0.0463
rs537412369	T	NA	NA	NA	0	NA	NA	NA	0	NA	NA	0
rs371531071	T	NA	NA	NA	0	NA	NA	NA	0	NA	NA	0
rs183141812	A	NA	NA	NA	0	NA	NA	NA	0.004673	NA	NA	0
rs189546747	T	NA	NA	NA	0	NA	NA	NA	0	NA	NA	0
rs3787950	C	0.1593	0	0.03125	0.299	0.02273	0	0.04348	0.07944	0.0303	0	0.3056
rs549067055	A	NA	NA	NA	0.004902	NA	NA	NA	0	NA	NA	0
rs576955441	A	NA	NA	NA	0	NA	NA	NA	0	NA	NA	0
rs8134203	T	NA	NA	NA	0.6324	NA	NA	NA	0.7804	NA	NA	0.5139
rs532216261	C	NA	NA	NA	0	NA	NA	NA	0	NA	NA	0
rs557766326	T	NA	NA	NA	0	NA	NA	NA	0	NA	NA	0
rs546531769	G	NA	NA	NA	0	NA	NA	NA	0	NA	NA	0
rs571993620	A	NA	NA	NA	0	NA	NA	NA	0	NA	NA	0
rs374261644	C	NA	NA	NA	0	NA	NA	NA	0	NA	NA	0.009259

SNP	Allele	Salvador	Shimaa	Shipibo	STU	Tacna	Tallanes	Trujillo	TSI	Tumbes	Uros	YRI
rs564210410	A	NA	NA	NA	0	NA	NA	NA	0	NA	NA	0.00463
rs562387776	C	NA	NA	NA	0	NA	NA	NA	0	NA	NA	0
rs562587205	T	NA	NA	NA	0	NA	NA	NA	0	NA	NA	0
rs542538936	T	NA	NA	NA	0	NA	NA	NA	0	NA	NA	0
rs143818732	C	NA	NA	NA	0.009804	NA	NA	NA	0.02804	NA	NA	0
rs183650725	A	NA	NA	NA	0	NA	NA	NA	0	NA	NA	0
rs531439352	A	NA	NA	NA	0	NA	NA	NA	0	NA	NA	0
rs531183955	T	NA	NA	NA	0	NA	NA	NA	0	NA	NA	0
rs144192191	GGTGA	NA	NA	NA	0.2647	NA	NA	NA	0.3037	NA	NA	0.3796
rs149708827	T	NA	NA	NA	0	NA	NA	NA	0	NA	NA	0
rs552635816	G	NA	NA	NA	0	NA	NA	NA	0	NA	NA	0
rs539520259	T	NA	NA	NA	0	NA	NA	NA	0	NA	NA	0
rs376235035	T	NA	NA	NA	0	NA	NA	NA	0	NA	NA	0
rs142303004	T	NA	NA	NA	0	NA	NA	NA	0	NA	NA	0.01389
rs546232086	G	NA	NA	NA	0	NA	NA	NA	0	NA	NA	0
rs549832756	C	NA	NA	NA	0	NA	NA	NA	0	NA	NA	0
rs75655573	G	0.00573	0	0	0	0	0	0	0	0	0	0.01389
rs553167698	T	NA	NA	NA	0	NA	NA	NA	0	NA	NA	0
rs190685013	A	NA	NA	NA	0	NA	NA	NA	0	NA	NA	0
rs559934921	G	NA	NA	NA	0	NA	NA	NA	0	NA	NA	0
rs190899605	C	NA	NA	NA	0.01961	NA	NA	NA	0	NA	NA	0
rs391099	C	NA	NA	NA	0.2304	NA	NA	NA	0.2897	NA	NA	0.4028
rs372422423	A	NA	NA	NA	0	NA	NA	NA	0	NA	NA	0.00463
rs77014365	A	NA	NA	NA	0	NA	NA	NA	0	NA	NA	0
rs562157895	A	NA	NA	NA	0	NA	NA	NA	0	NA	NA	0
rs187290362	G	NA	NA	NA	0	NA	NA	NA	0	NA	NA	0
rs182543256	A	NA	NA	NA	0	NA	NA	NA	0	NA	NA	0.00463
rs563186329	C	NA	NA	NA	0	NA	NA	NA	0.004673	NA	NA	0
rs569026066	T	NA	NA	NA	0	NA	NA	NA	0	NA	NA	0
rs116511699	T	NA	NA	NA	0	NA	NA	NA	0	NA	NA	0.01852
rs148499598	C	NA	NA	NA	0	NA	NA	NA	0.009346	NA	NA	0
rs552256264	T	NA	NA	NA	0	NA	NA	NA	0	NA	NA	0
rs562776963	A	NA	NA	NA	0	NA	NA	NA	0	NA	NA	0
rs79243099	A	NA	NA	NA	0	NA	NA	NA	0	NA	NA	0.02778
rs79397218	A	NA	NA	NA	0.009804	NA	NA	NA	0	NA	NA	0
rs138094318	T	NA	NA	NA	0	NA	NA	NA	0	NA	NA	0
rs567275769	T	NA	NA	NA	0	NA	NA	NA	0	NA	NA	0
rs544297320	T	NA	NA	NA	0	NA	NA	NA	0	NA	NA	0
rs561404433	G	NA	NA	NA	0.004902	NA	NA	NA	0	NA	NA	0
rs573923452	A	NA	NA	NA	0	NA	NA	NA	0	NA	NA	0
rs539858204	A	NA	NA	NA	0	NA	NA	NA	0	NA	NA	0
rs9305745	T	NA	NA	NA	0.3235	NA	NA	NA	0.2477	NA	NA	0.412
rs546510868	T	NA	NA	NA	0	NA	NA	NA	0	NA	NA	0
rs183418223	A	NA	NA	NA	0	NA	NA	NA	0	NA	NA	0.009259
rs573692135	T	NA	NA	NA	0.004902	NA	NA	NA	0	NA	NA	0

SNP	Allele	Salvador	Shimaa	Shipibo	STU	Tacna	Tallanes	Trujillo	TSI	Tumbes	Uros	YRI
rs570126729	A	NA	NA	NA	0	NA	NA	NA	0	NA	NA	0
rs542946711	G	NA	NA	NA	0.01961	NA	NA	NA	0.004673	NA	NA	0
rs551277697	G	NA	NA	NA	0	NA	NA	NA	0	NA	NA	0
rs73230088	A	NA	NA	NA	0.06863	NA	NA	NA	0.1402	NA	NA	0.00463
rs548025657	A	NA	NA	NA	0	NA	NA	NA	0	NA	NA	0
rs140428704	T	NA	NA	NA	0	NA	NA	NA	0.004673	NA	NA	0
rs184149889	C	NA	NA	NA	0	NA	NA	NA	0	NA	NA	0
rs116568213	G	NA	NA	NA	0	NA	NA	NA	0	NA	NA	0.01852
rs187742976	A	NA	NA	NA	0	NA	NA	NA	0	NA	NA	0
rs555769183	C	NA	NA	NA	0	NA	NA	NA	0	NA	NA	0
rs12627374	T	0.001146	0	0	0.1765	0	0	0	0.004673	0	0	0
rs565973484	A	NA	NA	NA	0	NA	NA	NA	0	NA	NA	0
rs547257017	C	NA	NA	NA	0	NA	NA	NA	0	NA	NA	0
rs568737536	A	NA	NA	NA	0	NA	NA	NA	0	NA	NA	0
rs541239376	T	NA	NA	NA	0	NA	NA	NA	0	NA	NA	0
rs557500448	A	NA	NA	NA	0	NA	NA	NA	0	NA	NA	0
rs185166990	A	NA	NA	NA	0	NA	NA	NA	0	NA	NA	0
rs541215881	A	NA	NA	NA	0	NA	NA	NA	0	NA	NA	0
rs138995130	A	NA	NA	NA	0	NA	NA	NA	0	NA	NA	0
rs462448	T	NA	NA	NA	0.07353	NA	NA	NA	0.01402	NA	NA	0
rs569303172	T	NA	NA	NA	0	NA	NA	NA	0	NA	NA	0
rs564691729	C	NA	NA	NA	0	NA	NA	NA	0	NA	NA	0
rs187452528	C	NA	NA	NA	0	NA	NA	NA	0	NA	NA	0
rs4818242	T	NA	NA	NA	0	NA	NA	NA	0	NA	NA	0
rs183016576	T	NA	NA	NA	0	NA	NA	NA	0	NA	NA	0
rs539934676	A	NA	NA	NA	0	NA	NA	NA	0	NA	NA	0
rs184876485	G	NA	NA	NA	0	NA	NA	NA	0	NA	NA	0
rs549676267	T	NA	NA	NA	0	NA	NA	NA	0	NA	NA	0
rs150633108	T	NA	NA	NA	0.04412	NA	NA	NA	0.009346	NA	NA	0
rs185103560	A	NA	NA	NA	0	NA	NA	NA	0	NA	NA	0
rs532655542	G	NA	NA	NA	0	NA	NA	NA	0	NA	NA	0
rs180831387	A	NA	NA	NA	0	NA	NA	NA	0	NA	NA	0
rs569577322	T	NA	NA	NA	0	NA	NA	NA	0	NA	NA	0
rs144154504	C	NA	NA	NA	0	NA	NA	NA	0	NA	NA	0
rs556937466	T	NA	NA	NA	0	NA	NA	NA	0	NA	NA	0
rs115129572	T	0.01186	0	0	0	0	0	0	0	0	0	0.02778
rs578015897	T	NA	NA	NA	0	NA	NA	NA	0	NA	NA	0.00463
rs2298661	A	NA	NA	NA	0.2941	NA	NA	NA	0.2009	NA	NA	0.1944
rs557088853	T	NA	NA	NA	0	NA	NA	NA	0	NA	NA	0
rs141301979	C	NA	NA	NA	0	NA	NA	NA	0	NA	NA	0.01852
rs8131649	T	0.3598	0.8478	0.6875	0.3627	0.8409	0.91429	0.6875	0.215	0.6212	1	0.3981
rs539860607	G	NA	NA	NA	0	NA	NA	NA	0	NA	NA	0
rs4816720	C	NA	NA	NA	0.07353	NA	NA	NA	0.01869	NA	NA	0.1898
rs11702475	T	NA	NA	NA	0.3922	NA	NA	NA	0.4907	NA	NA	0.1157
rs150205743	A	NA	NA	NA	0	NA	NA	NA	0	NA	NA	0

SNP	Allele	Salvador	Shimaa	Shipibo	STU	Tacna	Tallanes	Trujillo	TSI	Tumbes	Uros	YRI
rs8127674	G	0.3272	0.08696	0.2812	0.2647	0.09091	0.04286	0.125	0.4346	0.1212	0	0.4028
rs190134102	A	NA	NA	NA	0	NA	NA	NA	0	NA	NA	0
rs141324737	T	NA	NA	NA	0	NA	NA	NA	0	NA	NA	0
rs560898303	CA	NA	NA	NA	0	NA	NA	NA	0	NA	NA	0
rs552105586	T	NA	NA	NA	0	NA	NA	NA	0	NA	NA	0
rs146052428	T	NA	NA	NA	0	NA	NA	NA	0	NA	NA	0.01389
rs188241223	T	NA	NA	NA	0	NA	NA	NA	0	NA	NA	0
rs547639377	G	NA	NA	NA	0	NA	NA	NA	0	NA	NA	0
rs420737	G	NA	NA	NA	0.2108	NA	NA	NA	0.009346	NA	NA	0.2454
rs534978728	G	NA	NA	NA	0	NA	NA	NA	0	NA	NA	0
rs369948528	T	NA	NA	NA	0	NA	NA	NA	0	NA	NA	0
rs533867263	A	NA	NA	NA	0	NA	NA	NA	0	NA	NA	0.009259
rs555525436	G	NA	NA	NA	0	NA	NA	NA	0	NA	NA	0
rs531121505	T	NA	NA	NA	0	NA	NA	NA	0	NA	NA	0
rs376143876	A	NA	NA	NA	0	NA	NA	NA	0	NA	NA	0
rs554986236	T	NA	NA	NA	0	NA	NA	NA	0	NA	NA	0
rs66575656	T	NA	NA	NA	0.2451	NA	NA	NA	0.2991	NA	NA	0.2685
rs542746297	T	NA	NA	NA	0	NA	NA	NA	0	NA	NA	0
rs567175997	G	NA	NA	NA	0	NA	NA	NA	0	NA	NA	0
rs578020496	C	NA	NA	NA	0.004902	NA	NA	NA	0	NA	NA	0
rs543607180	C	NA	NA	NA	0	NA	NA	NA	0	NA	NA	0
rs115849825	A	NA	NA	NA	0	NA	NA	NA	0	NA	NA	0.00463
rs542333608	T	NA	NA	NA	0	NA	NA	NA	0	NA	NA	0
rs34983238	C	NA	NA	NA	0.08333	NA	NA	NA	0.1776	NA	NA	0.0463
rs372733383	T	NA	NA	NA	0	NA	NA	NA	0	NA	NA	0
rs555134126	T	NA	NA	NA	0	NA	NA	NA	0	NA	NA	0
rs533043037	A	NA	NA	NA	0.009804	NA	NA	NA	0	NA	NA	0
rs73372168	A	NA	NA	NA	0	NA	NA	NA	0	NA	NA	0.03241
rs150715708	T	NA	NA	NA	0	NA	NA	NA	0.004673	NA	NA	0
rs145024812	A	NA	NA	NA	0	NA	NA	NA	0	NA	NA	0.009259
rs557592331	C	NA	NA	NA	0.004902	NA	NA	NA	0	NA	NA	0
rs567380110	T	NA	NA	NA	0	NA	NA	NA	0	NA	NA	0
rs560408119	A	NA	NA	NA	0	NA	NA	NA	0	NA	NA	0
rs117432315	A	NA	NA	NA	0	NA	NA	NA	0	NA	NA	0
rs370654859	C	NA	NA	NA	0	NA	NA	NA	0	NA	NA	0.009259
rs139181746	C	NA	NA	NA	0	NA	NA	NA	0	NA	NA	0
rs554175681	G	NA	NA	NA	0	NA	NA	NA	0	NA	NA	0
rs28548447	A	NA	NA	NA	0.2549	NA	NA	NA	0.3458	NA	NA	0.2685
rs191526916	T	NA	NA	NA	0	NA	NA	NA	0	NA	NA	0
rs192959767	T	NA	NA	NA	0	NA	NA	NA	0	NA	NA	0
rs534192391	T	NA	NA	NA	0.004902	NA	NA	NA	0	NA	NA	0
rs571211518	C	NA	NA	NA	0.004902	NA	NA	NA	0	NA	NA	0
rs562033822	A	NA	NA	NA	0	NA	NA	NA	0	NA	NA	0
rs142194573	AG	NA	NA	NA	0	NA	NA	NA	0	NA	NA	0
rs149695119	T	NA	NA	NA	0.004902	NA	NA	NA	0.004673	NA	NA	0.2269

SNP	Allele	Salvador	Shimaa	Shipibo	STU	Tacna	Tallanes	Trujillo	TSI	Tumbes	Uros	YRI
rs143270468	A	NA	NA	NA	0	NA	NA	NA	0.004673	NA	NA	0
rs534423780	A	NA	NA	NA	0	NA	NA	NA	0	NA	NA	0
rs535545388	G	NA	NA	NA	0	NA	NA	NA	0	NA	NA	0
rs528206157	T	NA	NA	NA	0	NA	NA	NA	0	NA	NA	0
rs112985398	C	NA	NA	NA	0	NA	NA	NA	0	NA	NA	0.01852
rs77511690	A	NA	NA	NA	0	NA	NA	NA	0	NA	NA	0.06481
rs538161583	A	NA	NA	NA	0	NA	NA	NA	0	NA	NA	0
rs552591104	C	NA	NA	NA	0	NA	NA	NA	0	NA	NA	0
rs557224603	A	NA	NA	NA	0	NA	NA	NA	0	NA	NA	0
rs545825254	G	NA	NA	NA	0	NA	NA	NA	0	NA	NA	0
rs185312677	T	NA	NA	NA	0	NA	NA	NA	0	NA	NA	0.00463
rs423596	T	NA	NA	NA	0.1667	NA	NA	NA	0.06075	NA	NA	0
rs541726793	A	NA	NA	NA	0	NA	NA	NA	0	NA	NA	0
rs62217529	T	NA	NA	NA	0	NA	NA	NA	0	NA	NA	0
rs528452414	C	NA	NA	NA	0.004902	NA	NA	NA	0	NA	NA	0
rs143167369	T	NA	NA	NA	0	NA	NA	NA	0	NA	NA	0
rs138164084	T	NA	NA	NA	0	NA	NA	NA	0	NA	NA	0.00463
rs62217531	T	0.3724	0.1304	0.2812	0.3627	0.09091	0.07143	0.1667	0.4579	0.2727	0	0.3148
rs528205318	G	NA	NA	NA	0.004902	NA	NA	NA	0	NA	NA	0
rs554097496	A	NA	NA	NA	0	NA	NA	NA	0	NA	NA	0
rs61728255	A	NA	NA	NA	0.06863	NA	NA	NA	0.01402	NA	NA	0
rs538022064	C	NA	NA	NA	0	NA	NA	NA	0.004673	NA	NA	0
rs9980225	C	NA	NA	NA	0.08333	NA	NA	NA	0.1402	NA	NA	0
rs542530281	C	NA	NA	NA	0	NA	NA	NA	0	NA	NA	0
rs542407537	A	NA	NA	NA	0	NA	NA	NA	0	NA	NA	0
rs548552823	A	NA	NA	NA	0	NA	NA	NA	0	NA	NA	0
rs565903681	C	NA	NA	NA	0.004902	NA	NA	NA	0	NA	NA	0
rs201109436	G	NA	NA	NA	0	NA	NA	NA	0	NA	NA	0
rs577393745	T	NA	NA	NA	0	NA	NA	NA	0	NA	NA	0
rs185086499	T	NA	NA	NA	0	NA	NA	NA	0	NA	NA	0
rs141230106	T	NA	NA	NA	0.004902	NA	NA	NA	0	NA	NA	0
rs531548362	C	NA	NA	NA	0	NA	NA	NA	0	NA	NA	0
rs150445636	A	NA	NA	NA	0	NA	NA	NA	0	NA	NA	0
rs139015396	T	NA	NA	NA	0	NA	NA	NA	0	NA	NA	0.009259
rs183722985	A	NA	NA	NA	0	NA	NA	NA	0	NA	NA	0.009259
rs543734531	A	NA	NA	NA	0	NA	NA	NA	0	NA	NA	0
rs553875292	T	NA	NA	NA	0	NA	NA	NA	0	NA	NA	0.00463
rs55760462	C	0.2517	0.08696	0.3438	0.1569	0.09091	0.02857	0.08333	0.2383	0.09091	0	0.3287
rs61735790	C	NA	NA	NA	0	NA	NA	NA	0.004673	NA	NA	0.00463
rs566918037	G	NA	NA	NA	0	NA	NA	NA	0	NA	NA	0
rs555820997	G	NA	NA	NA	0	NA	NA	NA	0.004673	NA	NA	0.00463
rs573493693	A	NA	NA	NA	0	NA	NA	NA	0	NA	NA	0
rs914184	A	NA	NA	NA	0	NA	NA	NA	0	NA	NA	0
rs539019494	C	NA	NA	NA	0	NA	NA	NA	0	NA	NA	0
rs544036587	T	NA	NA	NA	0	NA	NA	NA	0	NA	NA	0

SNP	Allele	Salvador	Shimaa	Shipibo	STU	Tacna	Tallanes	Trujillo	TSI	Tumbes	Uros	YRI
rs565896303	A	NA	NA	NA	0	NA	NA	NA	0	NA	NA	0
rs192793047	C	NA	NA	NA	0	NA	NA	NA	0	NA	NA	0
rs546790067	C	NA	NA	NA	0	NA	NA	NA	0	NA	NA	0
rs553672299	A	NA	NA	NA	0	NA	NA	NA	0	NA	NA	0
rs570629130	A	NA	NA	NA	0	NA	NA	NA	0	NA	NA	0.00463
rs117562633	C	NA	NA	NA	0	NA	NA	NA	0	NA	NA	0
rs395584	C	0.2074	0.5435	0.2812	0.2108	0.5455	0.6571	0.5	0.009346	0.4848	0.5938	0.2454
rs143597099	A	NA	NA	NA	NA	NA	NA	NA	NA	NA	NA	NA
rs543168691	C	NA	NA	NA	0	NA	NA	NA	0	NA	NA	0
rs530689404	T	NA	NA	NA	0.004902	NA	NA	NA	0	NA	NA	0
rs76833541	A	NA	NA	NA	0.01471	NA	NA	NA	0.0514	NA	NA	0
rs150907799	T	NA	NA	NA	0	NA	NA	NA	0	NA	NA	0
rs554550449	A	NA	NA	NA	0.004902	NA	NA	NA	0	NA	NA	0
rs566085188	A	NA	NA	NA	0	NA	NA	NA	0	NA	NA	0
rs553431242	G	NA	NA	NA	0	NA	NA	NA	0	NA	NA	0
rs551442787	A	NA	NA	NA	0	NA	NA	NA	0	NA	NA	0
rs551677463	C	NA	NA	NA	0	NA	NA	NA	0	NA	NA	0
rs549801931	T	NA	NA	NA	0	NA	NA	NA	0	NA	NA	0
rs141864728	C	NA	NA	NA	0	NA	NA	NA	0	NA	NA	0
rs373480225	T	NA	NA	NA	0	NA	NA	NA	0	NA	NA	0
rs73903405	A	NA	NA	NA	0	NA	NA	NA	0	NA	NA	0.05093
rs567039032	A	NA	NA	NA	0.004902	NA	NA	NA	0	NA	NA	0
rs2070792	A	0.4202	0.02174	0.03125	0.2598	0.06818	0.01429	0.1667	0.3411	0.1212	0	0.5833
rs552550910	A	NA	NA	NA	0	NA	NA	NA	0	NA	NA	0
rs535778149	A	NA	NA	NA	0	NA	NA	NA	0	NA	NA	0
rs201984814	A	NA	NA	NA	0	NA	NA	NA	0	NA	NA	0
rs544847277	A	NA	NA	NA	0	NA	NA	NA	0	NA	NA	0
rs2070793	G	NA	NA	NA	0.2598	NA	NA	NA	0.3411	NA	NA	0.5833
rs191345780	A	NA	NA	NA	0	NA	NA	NA	0	NA	NA	0
rs9980693	A	NA	NA	NA	0.05392	NA	NA	NA	0.1449	NA	NA	0.00463
rs146865760	T	NA	NA	NA	0.004902	NA	NA	NA	0.02336	NA	NA	0
rs527898967	G	NA	NA	NA	0	NA	NA	NA	0	NA	NA	0
rs150389990	T	NA	NA	NA	NA	NA	NA	NA	NA	NA	NA	NA
rs558492934	T	NA	NA	NA	0	NA	NA	NA	0	NA	NA	0
rs533513206	C	NA	NA	NA	0	NA	NA	NA	0	NA	NA	0
rs183758144	A	NA	NA	NA	0	NA	NA	NA	0.004673	NA	NA	0
rs377403765	A	NA	NA	NA	0	NA	NA	NA	0	NA	NA	0
rs543740889	C	NA	NA	NA	0	NA	NA	NA	0	NA	NA	0
rs563188125	A	NA	NA	NA	0.004902	NA	NA	NA	0	NA	NA	0
rs536677167	G	NA	NA	NA	0	NA	NA	NA	0	NA	NA	0
rs541782164	T	NA	NA	NA	0.009804	NA	NA	NA	0	NA	NA	0
rs138161288	T	NA	NA	NA	0	NA	NA	NA	0	NA	NA	0
rs541499151	A	NA	NA	NA	0	NA	NA	NA	0	NA	NA	0
rs543339705	C	NA	NA	NA	0	NA	NA	NA	0	NA	NA	0
rs77599791	G	NA	NA	NA	0	NA	NA	NA	0	NA	NA	0.00463

SNP	Allele	Salvador	Shimaa	Shipibo	STU	Tacna	Tallanes	Trujillo	TSI	Tumbes	Uros	YRI
rs549745562	G	NA	NA	NA	0	NA	NA	NA	0	NA	NA	0
rs75317729	C	NA	NA	NA	0	NA	NA	NA	0	NA	NA	0.009259
rs139661079	A	NA	NA	NA	0	NA	NA	NA	0	NA	NA	0.00463
rs572699613	A	NA	NA	NA	0	NA	NA	NA	0	NA	NA	0
rs536023174	T	NA	NA	NA	0	NA	NA	NA	0	NA	NA	0
rs415731	A	0.2712	NA	NA	0.2843	NA	NA	NA	0.3084	NA	NA	0.1991
rs3761374	C	0.06475	0	0	0.05882	0	0	0.04348	0.1449	0.09677	0	0
rs147233451	T	NA	NA	NA	0	NA	NA	NA	0	NA	NA	0
rs143595083	T	NA	NA	NA	0	NA	NA	NA	0	NA	NA	0.009259
rs565237319	C	NA	NA	NA	0	NA	NA	NA	0	NA	NA	0
rs576510757	G	NA	NA	NA	0	NA	NA	NA	0	NA	NA	0
rs535235411	A	NA	NA	NA	0.004902	NA	NA	NA	0	NA	NA	0
rs190265904	A	NA	NA	NA	0	NA	NA	NA	0	NA	NA	0
rs149527323	A	NA	NA	NA	0	NA	NA	NA	0	NA	NA	0.00463
rs544141276	A	NA	NA	NA	0	NA	NA	NA	0	NA	NA	0
rs186097675	T	NA	NA	NA	0	NA	NA	NA	0	NA	NA	0
rs547168890	A	NA	NA	NA	0.004902	NA	NA	NA	0	NA	NA	0
rs138928680	GAGAA	NA	NA	NA	0	NA	NA	NA	0	NA	NA	0.00463
rs567676569	T	NA	NA	NA	0	NA	NA	NA	0	NA	NA	0
rs530701233	A	NA	NA	NA	0.004902	NA	NA	NA	0	NA	NA	0
rs562332833	T	NA	NA	NA	0	NA	NA	NA	0	NA	NA	0
rs531545827	T	NA	NA	NA	0	NA	NA	NA	0	NA	NA	0
rs530584676	T	NA	NA	NA	0	NA	NA	NA	0	NA	NA	0
rs185416185	T	NA	NA	NA	0.004902	NA	NA	NA	0	NA	NA	0
rs429442	T	NA	NA	NA	0.2304	NA	NA	NA	0.285	NA	NA	0.3565
rs79065748	A	NA	NA	NA	0	NA	NA	NA	0	NA	NA	0.06481
rs575760704	C	NA	NA	NA	0	NA	NA	NA	0	NA	NA	0
rs875393	A	NA	NA	NA	0.02941	NA	NA	NA	0.02336	NA	NA	0
rs148901354	T	NA	NA	NA	0	NA	NA	NA	0.009346	NA	NA	0
rs561279116	G	NA	NA	NA	0	NA	NA	NA	0	NA	NA	0
rs138661151	A	NA	NA	NA	0	NA	NA	NA	0	NA	NA	0.01389
rs9975014	G	NA	NA	NA	0.2353	NA	NA	NA	0.2991	NA	NA	0.2963
rs573564326	A	NA	NA	NA	0.009804	NA	NA	NA	0.009346	NA	NA	0.009259
rs144276163	G	NA	NA	NA	0	NA	NA	NA	0	NA	NA	0
rs142750000	T	NA	NA	NA	0	NA	NA	NA	0.004673	NA	NA	0
rs143672898	T	NA	NA	NA	0	NA	NA	NA	0	NA	NA	0
rs560182574	C	NA	NA	NA	0	NA	NA	NA	0	NA	NA	0
rs534312135	T	NA	NA	NA	0	NA	NA	NA	0	NA	NA	0
rs147986574	A	NA	NA	NA	0	NA	NA	NA	0	NA	NA	0
rs181874957	T	NA	NA	NA	0	NA	NA	NA	0.004673	NA	NA	0
rs574957059	A	NA	NA	NA	0	NA	NA	NA	0	NA	NA	0
rs185018051	C	NA	NA	NA	0	NA	NA	NA	0	NA	NA	0
rs564806692	A	NA	NA	NA	0	NA	NA	NA	0.004673	NA	NA	0
rs556079659	C	NA	NA	NA	0.009804	NA	NA	NA	0	NA	NA	0
rs79617378	C	0.006112	0	0	0	0	0	0	0.01402	0.01515	0	0

SNP	Allele	Salvador	Shimaa	Shipibo	STU	Tacna	Tallanes	Trujillo	TSI	Tumbes	Uros	YRI
rs189436980	G	NA	NA	NA	0	NA	NA	NA	0	NA	NA	0
rs187078345	A	NA	NA	NA	0	NA	NA	NA	0	NA	NA	0.009259
rs534885992	A	NA	NA	NA	0	NA	NA	NA	0	NA	NA	0
rs187143520	C	NA	NA	NA	0	NA	NA	NA	0	NA	NA	0.00463
rs535174935	T	NA	NA	NA	0	NA	NA	NA	0	NA	NA	0
rs528681261	T	NA	NA	NA	0	NA	NA	NA	0	NA	NA	0
rs563352095	T	NA	NA	NA	0.009804	NA	NA	NA	0	NA	NA	0
rs117688613	G	NA	NA	NA	0	NA	NA	NA	0	NA	NA	0
rs550253934	G	NA	NA	NA	0.009804	NA	NA	NA	0	NA	NA	0
rs181162134	C	NA	NA	NA	0	NA	NA	NA	0	NA	NA	0
rs192026800	A	NA	NA	NA	0	NA	NA	NA	0	NA	NA	0
rs552303675	T	NA	NA	NA	0	NA	NA	NA	0.004673	NA	NA	0
rs147147950	A	NA	NA	NA	0	NA	NA	NA	0	NA	NA	0
rs76314085	G	NA	NA	NA	0	NA	NA	NA	0	NA	NA	0
rs567504414	T	NA	NA	NA	0	NA	NA	NA	0	NA	NA	0
rs563853944	C	NA	NA	NA	0	NA	NA	NA	0.004673	NA	NA	0
rs578103765	T	NA	NA	NA	0	NA	NA	NA	0	NA	NA	0
rs574238899	G	NA	NA	NA	0	NA	NA	NA	0	NA	NA	0
rs2838040	G	NA	NA	NA	0.3284	NA	NA	NA	0.2477	NA	NA	0.5324
rs537876986	A	NA	NA	NA	0	NA	NA	NA	0.004673	NA	NA	0
rs550222907	T	NA	NA	NA	0	NA	NA	NA	0	NA	NA	0
rs556260609	A	NA	NA	NA	0	NA	NA	NA	0.004673	NA	NA	0
rs142296178	A	NA	NA	NA	0	NA	NA	NA	0	NA	NA	0
rs577285910	A	NA	NA	NA	0	NA	NA	NA	0	NA	NA	0
rs566925007	A	NA	NA	NA	0	NA	NA	NA	0	NA	NA	0
rs457909	G	NA	NA	NA	0	NA	NA	NA	0	NA	NA	0
rs562479511	T	NA	NA	NA	0	NA	NA	NA	0	NA	NA	0
rs2298663	T	NA	NA	NA	0.6373	NA	NA	NA	0.7804	NA	NA	0.4954
rs2070787	G	NA	NA	NA	0.2549	NA	NA	NA	0.3411	NA	NA	0.3843
rs573615979	C	NA	NA	NA	0	NA	NA	NA	0	NA	NA	0.01389
rs542031508	C	NA	NA	NA	0	NA	NA	NA	0	NA	NA	0
rs555600211	A	NA	NA	NA	0	NA	NA	NA	0.004673	NA	NA	0
rs528451616	A	NA	NA	NA	0.009804	NA	NA	NA	0	NA	NA	0.00463
rs378501	G	NA	NA	NA	0	NA	NA	NA	0	NA	NA	0
rs140408843	A	NA	NA	NA	0	NA	NA	NA	0	NA	NA	0
rs186280517	C	NA	NA	NA	0	NA	NA	NA	0	NA	NA	0
rs148561341	T	NA	NA	NA	0	NA	NA	NA	0	NA	NA	0
rs568162250	A	NA	NA	NA	0	NA	NA	NA	0	NA	NA	0.00463
rs561709263	C	NA	NA	NA	0	NA	NA	NA	0	NA	NA	0
rs569583276	A	NA	NA	NA	0.004902	NA	NA	NA	0	NA	NA	0.00463
rs572164476	A	NA	NA	NA	0	NA	NA	NA	0.004673	NA	NA	0
rs560575827	T	NA	NA	NA	0	NA	NA	NA	0.004673	NA	NA	0
rs111220492	G	NA	NA	NA	0.3824	NA	NA	NA	0.4813	NA	NA	0.2269
rs182231135	C	NA	NA	NA	0	NA	NA	NA	0	NA	NA	0
rs531884512	T	NA	NA	NA	0	NA	NA	NA	0	NA	NA	0

SNP	Allele	Salvador	Shimaa	Shipibo	STU	Tacna	Tallanes	Trujillo	TSI	Tumbes	Uros	YRI
rs573986970	A	NA	NA	NA	0	NA	NA	NA	0	NA	NA	0
rs564705715	A	NA	NA	NA	0	NA	NA	NA	0	NA	NA	0
rs201661208	A	NA	NA	NA	0	NA	NA	NA	0	NA	NA	0
rs530540353	C	NA	NA	NA	0	NA	NA	NA	0	NA	NA	0
rs148155433	A	NA	NA	NA	0	NA	NA	NA	0	NA	NA	0.00463
rs558834486	G	NA	NA	NA	0	NA	NA	NA	0	NA	NA	0
rs186275240	A	NA	NA	NA	0	NA	NA	NA	0	NA	NA	0
rs531418415	A	NA	NA	NA	0	NA	NA	NA	0	NA	NA	0
rs531609155	G	NA	NA	NA	0	NA	NA	NA	0	NA	NA	0.00463
rs376752614	A	NA	NA	NA	0	NA	NA	NA	0	NA	NA	0
rs139144487	A	NA	NA	NA	0	NA	NA	NA	0	NA	NA	0
rs531754656	A	NA	NA	NA	0	NA	NA	NA	0	NA	NA	0
rs554702767	A	NA	NA	NA	0	NA	NA	NA	0	NA	NA	0
rs456016	T	NA	NA	NA	0.07353	NA	NA	NA	0.01402	NA	NA	0.00463
rs144364265	T	NA	NA	NA	0	NA	NA	NA	0	NA	NA	0
rs113773731	C	NA	NA	NA	0	NA	NA	NA	0	NA	NA	0.00463
rs142261174	T	NA	NA	NA	0	NA	NA	NA	0	NA	NA	0.009259
rs563595464	T	NA	NA	NA	0	NA	NA	NA	0	NA	NA	0
rs531013422	T	NA	NA	NA	0	NA	NA	NA	0	NA	NA	0
rs569886879	G	NA	NA	NA	0	NA	NA	NA	0	NA	NA	0
rs541977145	G	NA	NA	NA	0	NA	NA	NA	0	NA	NA	0
rs190906841	T	NA	NA	NA	0	NA	NA	NA	0	NA	NA	0
rs139222305	T	NA	NA	NA	0	NA	NA	NA	0.02336	NA	NA	0
rs527242422	T	NA	NA	NA	0.004902	NA	NA	NA	0	NA	NA	0
rs364289	A	NA	NA	NA	0.2353	NA	NA	NA	0.2664	NA	NA	0.4676
rs371053759	T	NA	NA	NA	0	NA	NA	NA	0.004673	NA	NA	0
rs146116431	T	NA	NA	NA	0	NA	NA	NA	0	NA	NA	0
rs928871	C	NA	NA	NA	0.6765	NA	NA	NA	0.7523	NA	NA	0.3704
rs532335955	T	NA	NA	NA	0	NA	NA	NA	0	NA	NA	0
rs113928389	A	NA	NA	NA	0	NA	NA	NA	0	NA	NA	0.009259
rs147138431	A	NA	NA	NA	0.004902	NA	NA	NA	0	NA	NA	0
rs4283504	T	NA	NA	NA	0.152	NA	NA	NA	0.07944	NA	NA	0.03704
rs145297649	G	NA	NA	NA	0	NA	NA	NA	0	NA	NA	0.00463
rs146957681	T	NA	NA	NA	0	NA	NA	NA	0.004673	NA	NA	0
rs562177468	G	NA	NA	NA	0	NA	NA	NA	0	NA	NA	0
rs576030223	A	NA	NA	NA	0	NA	NA	NA	0	NA	NA	0.00463
rs190385097	A	NA	NA	NA	0	NA	NA	NA	0.004673	NA	NA	0
rs7283324	T	NA	NA	NA	0.2794	NA	NA	NA	0.215	NA	NA	0.3148
rs118108663	T	NA	NA	NA	0	NA	NA	NA	0	NA	NA	0
rs61735789	A	NA	NA	NA	0	NA	NA	NA	0.01402	NA	NA	0
rs113564116	C	NA	NA	NA	0	NA	NA	NA	0.004673	NA	NA	0
rs2298659	A	NA	NA	NA	0.1863	NA	NA	NA	0.2196	NA	NA	0.162
rs150581606	A	NA	NA	NA	0	NA	NA	NA	0	NA	NA	0
rs116170128	C	NA	NA	NA	0.01471	NA	NA	NA	0.04673	NA	NA	0.1481
rs143291395	A	NA	NA	NA	0	NA	NA	NA	0	NA	NA	0

SNP	Allele	Salvador	Shimaa	Shipibo	STU	Tacna	Tallanes	Trujillo	TSI	Tumbes	Uros	YRI
rs112753686	T	NA	NA	NA	0	NA	NA	NA	0	NA	NA	0.01389
rs75603675	A	NA	NA	NA	0.2745	NA	NA	NA	0.4346	NA	NA	0.412
rs553516093	A	NA	NA	NA	0	NA	NA	NA	0	NA	NA	0
rs143460343	A	NA	NA	NA	0	NA	NA	NA	0	NA	NA	0.01852
rs184365262	T	NA	NA	NA	0	NA	NA	NA	0	NA	NA	0
rs571074719	T	NA	NA	NA	0	NA	NA	NA	0	NA	NA	0.00463
rs115975538	A	NA	NA	NA	0.004902	NA	NA	NA	0	NA	NA	0.08796
rs147465180	A	NA	NA	NA	0	NA	NA	NA	0.009346	NA	NA	0
rs548349124	G	NA	NA	NA	0	NA	NA	NA	0	NA	NA	0
rs139467735	T	NA	NA	NA	0.004902	NA	NA	NA	0	NA	NA	0.0787
rs576042280	T	NA	NA	NA	0	NA	NA	NA	0	NA	NA	0
rs544083065	A	NA	NA	NA	0	NA	NA	NA	0	NA	NA	0
rs551244742	A	NA	NA	NA	0	NA	NA	NA	0	NA	NA	0
rs149076631	T	NA	NA	NA	0	NA	NA	NA	0	NA	NA	0
rs35041537	T	0.2645	0.1304	0.2812	0.3676	0.09091	0.07143	0.1458	0.4439	0.2727	0	0.1204
rs56218846	A	NA	NA	NA	0.2647	NA	NA	NA	0.4346	NA	NA	0.4028
rs527693716	T	NA	NA	NA	0	NA	NA	NA	0	NA	NA	0
rs189298425	A	NA	NA	NA	0	NA	NA	NA	0	NA	NA	0
rs562750287	A	NA	NA	NA	0	NA	NA	NA	0	NA	NA	0
rs570413089	A	NA	NA	NA	0	NA	NA	NA	0	NA	NA	0
rs111383922	T	NA	NA	NA	0	NA	NA	NA	0.004673	NA	NA	0
rs559699558	A	NA	NA	NA	0	NA	NA	NA	0	NA	NA	0
rs542060428	T	NA	NA	NA	0	NA	NA	NA	0	NA	NA	0
rs565142477	T	NA	NA	NA	0	NA	NA	NA	0	NA	NA	0
rs548742822	C	NA	NA	NA	0	NA	NA	NA	0	NA	NA	0
rs148304071	T	NA	NA	NA	0	NA	NA	NA	0	NA	NA	0
rs455045	C	NA	NA	NA	0.3431	NA	NA	NA	0.4486	NA	NA	0.2361
rs570441750	G	NA	NA	NA	0	NA	NA	NA	0	NA	NA	0
rs9974589	A	NA	NA	NA	0.4608	NA	NA	NA	0.4346	NA	NA	0.3148
rs2104810	A	NA	NA	NA	0.6324	NA	NA	NA	0.7804	NA	NA	0.5139
rs147711290	C	NA	NA	NA	NA	NA	NA	NA	NA	NA	NA	NA
rs555056677	A	NA	NA	NA	0	NA	NA	NA	0	NA	NA	0
rs9974933	G	NA	NA	NA	0.2255	NA	NA	NA	0.2944	NA	NA	0.2963
rs544221548	A	NA	NA	NA	0	NA	NA	NA	0	NA	NA	0
rs147827602	T	NA	NA	NA	0	NA	NA	NA	0.004673	NA	NA	0
rs462321	T	NA	NA	NA	0.348	NA	NA	NA	0.1822	NA	NA	0.2917
rs142659685	T	NA	NA	NA	0	NA	NA	NA	0.004673	NA	NA	0
rs150838246	C	NA	NA	NA	0	NA	NA	NA	0	NA	NA	0
rs112132031	C	NA	NA	NA	0.08333	NA	NA	NA	0.01402	NA	NA	0.213
rs9974995	T	0.2846	0.08696	0.2188	0.2255	0.2273	0.1714	0.2708	0.2944	0.1515	0.375	0.2963
rs557494549	TCAGGGAGTGCAGAGCAGGAGGGAC	NA	NA	NA	0	NA	NA	NA	0	NA	NA	0.01389
rs186929947	G	NA	NA	NA	0	NA	NA	NA	0	NA	NA	0
rs148509204	T	NA	NA	NA	0	NA	NA	NA	0	NA	NA	0
rs202094412	A	NA	NA	NA	0	NA	NA	NA	0	NA	NA	0
rs575981127	A	NA	NA	NA	0	NA	NA	NA	0	NA	NA	0

SNP	Allele	Salvador	Shimaa	Shipibo	STU	Tacna	Tallanes	Trujillo	TSI	Tumbes	Uros	YRI
rs546775580	T	NA	NA	NA	0	NA	NA	NA	0	NA	NA	0
rs185188053	T	NA	NA	NA	0	NA	NA	NA	0	NA	NA	0
rs577381045	A	NA	NA	NA	0	NA	NA	NA	0	NA	NA	0
rs62217525	T	NA	NA	NA	0.03922	NA	NA	NA	0.06075	NA	NA	0
rs561043908	A	NA	NA	NA	0	NA	NA	NA	0	NA	NA	0
rs367879274	T	NA	NA	NA	0	NA	NA	NA	0	NA	NA	0
rs568167564	G	NA	NA	NA	0	NA	NA	NA	0	NA	NA	0
rs563633617	A	NA	NA	NA	0	NA	NA	NA	0	NA	NA	0
rs537935645	T	NA	NA	NA	0	NA	NA	NA	0	NA	NA	0
rs9985159	C	0.66	0.8696	0.5938	0.7157	0.8182	0.8286	0.8333	0.7991	0.7879	0.625	0.5463
rs61735795	A	NA	NA	NA	NA	NA	NA	NA	NA	NA	NA	NA
rs557712450	C	NA	NA	NA	0	NA	NA	NA	0.004673	NA	NA	0
rs139305247	T	NA	NA	NA	0	NA	NA	NA	0.01869	NA	NA	0
rs548653178	A	NA	NA	NA	0	NA	NA	NA	0.004673	NA	NA	0
rs146385718	C	NA	NA	NA	0	NA	NA	NA	0	NA	NA	0.01852
rs28360562	C	NA	NA	NA	0.08333	NA	NA	NA	0.1776	NA	NA	0.06019
rs9983330	G	NA	NA	NA	0.2941	NA	NA	NA	0.2056	NA	NA	0.2546
rs539864066	G	NA	NA	NA	0	NA	NA	NA	0.004673	NA	NA	0.009259
rs562014334	A	NA	NA	NA	0	NA	NA	NA	0	NA	NA	0
rs118134524	A	NA	NA	NA	0	NA	NA	NA	0	NA	NA	0
rs574811036	A	NA	NA	NA	0	NA	NA	NA	0	NA	NA	0
rs115720411	A	NA	NA	NA	0	NA	NA	NA	0	NA	NA	0.2269
rs368812287	T	NA	NA	NA	0	NA	NA	NA	0	NA	NA	0
rs2410428	T	NA	NA	NA	0.2206	NA	NA	NA	0.2944	NA	NA	0.1481
rs577480005	A	NA	NA	NA	0	NA	NA	NA	0	NA	NA	0.00463
rs543815381	T	NA	NA	NA	0	NA	NA	NA	0	NA	NA	0
rs142834250	G	NA	NA	NA	0	NA	NA	NA	0	NA	NA	0
rs576292358	A	NA	NA	NA	0	NA	NA	NA	0	NA	NA	0
rs141603473	A	NA	NA	NA	0	NA	NA	NA	0	NA	NA	0.03241
rs143523726	G	NA	NA	NA	0	NA	NA	NA	0	NA	NA	0
rs375408	G	NA	NA	NA	0.08333	NA	NA	NA	0.01402	NA	NA	0
rs370347248	C	NA	NA	NA	0	NA	NA	NA	0	NA	NA	0
rs539123545	C	NA	NA	NA	0	NA	NA	NA	0	NA	NA	0
rs4303794	C	NA	NA	NA	0.2745	NA	NA	NA	0.4393	NA	NA	0.412
rs151035593	T	NA	NA	NA	0	NA	NA	NA	0	NA	NA	0.03241
rs370896169	C	NA	NA	NA	0	NA	NA	NA	0	NA	NA	0
rs575167859	T	NA	NA	NA	0	NA	NA	NA	0	NA	NA	0
rs35050484	A	NA	NA	NA	0.009804	NA	NA	NA	0.03271	NA	NA	0
rs56136037	T	NA	NA	NA	0	NA	NA	NA	0.0514	NA	NA	0
rs539767738	A	NA	NA	NA	0	NA	NA	NA	0	NA	NA	0
rs533229030	G	NA	NA	NA	0	NA	NA	NA	0	NA	NA	0
rs465576	C	NA	NA	NA	0.07843	NA	NA	NA	0.01402	NA	NA	0.2037
rs570494222	T	NA	NA	NA	0	NA	NA	NA	0	NA	NA	0
rs4818240	T	NA	NA	NA	0.07353	NA	NA	NA	0.01869	NA	NA	0.1898
rs9636988	C	0.2861	0.08696	0.2188	0.2255	0.2273	0.1714	0.2708	0.2991	0.1515	0.375	0.2963

SNP	Allele	Salvador	Shimaa	Shipibo	STU	Tacna	Tallanes	Trujillo	TSI	Tumbes	Uros	YRI
rs541887371	T	NA	NA	NA	0	NA	NA	NA	0	NA	NA	0
rs537787922	A	NA	NA	NA	0	NA	NA	NA	0	NA	NA	0
rs139829932	T	NA	NA	NA	0	NA	NA	NA	0	NA	NA	0.009259
rs200615061	C	NA	NA	NA	0	NA	NA	NA	0	NA	NA	0
rs3787947	T	NA	NA	NA	0.3186	NA	NA	NA	0.2477	NA	NA	0.412
rs402197	T	NA	NA	NA	0.08333	NA	NA	NA	0.01869	NA	NA	0.009259
rs562203987	T	NA	NA	NA	0	NA	NA	NA	0	NA	NA	0
rs141764184	A	NA	NA	NA	0	NA	NA	NA	0	NA	NA	0
rs116577479	A	NA	NA	NA	0	NA	NA	NA	0	NA	NA	0.0463
rs118133613	A	NA	NA	NA	0	NA	NA	NA	0.004673	NA	NA	0
rs118108194	G	NA	NA	NA	0	NA	NA	NA	0.02804	NA	NA	0
rs138763189	A	NA	NA	NA	0	NA	NA	NA	0	NA	NA	0.00463
rs138365638	TGG	NA	NA	NA	0.07353	NA	NA	NA	0.01402	NA	NA	0.01389
rs551442751	T	NA	NA	NA	0	NA	NA	NA	0	NA	NA	0
rs539019775	T	NA	NA	NA	0	NA	NA	NA	0	NA	NA	0
rs112657409	T	0.06011	0	0	0	0	0	0.02083	0	0	0	0.0787
rs577689706	A	NA	NA	NA	0	NA	NA	NA	0	NA	NA	0
rs147349930	T	NA	NA	NA	0	NA	NA	NA	0	NA	NA	0.009259
rs557531433	T	NA	NA	NA	0	NA	NA	NA	0.004673	NA	NA	0
rs144359794	T	NA	NA	NA	0	NA	NA	NA	0	NA	NA	0
rs187426170	T	NA	NA	NA	0	NA	NA	NA	0.004673	NA	NA	0
rs199575615	G	NA	NA	NA	0	NA	NA	NA	0	NA	NA	0
rs529737187	A	NA	NA	NA	0	NA	NA	NA	0	NA	NA	0
rs576447019	A	NA	NA	NA	0	NA	NA	NA	0	NA	NA	0
rs145900878	T	NA	NA	NA	0	NA	NA	NA	0	NA	NA	0.03704
rs9977234	T	NA	NA	NA	0.2206	NA	NA	NA	0.2804	NA	NA	0.1204
rs540024706	C	NA	NA	NA	0.004902	NA	NA	NA	0	NA	NA	0
rs528640390	G	NA	NA	NA	0	NA	NA	NA	0	NA	NA	0.00463
rs546337899	G	NA	NA	NA	0	NA	NA	NA	0.004673	NA	NA	0
rs141323355	A	NA	NA	NA	0	NA	NA	NA	0.004673	NA	NA	0
rs386519	G	NA	NA	NA	0	NA	NA	NA	0	NA	NA	0.00463
rs561327241	T	NA	NA	NA	0.009804	NA	NA	NA	0	NA	NA	0
rs572351113	A	NA	NA	NA	0	NA	NA	NA	0	NA	NA	0
rs189425119	A	NA	NA	NA	0	NA	NA	NA	0	NA	NA	0
rs566485026	G	NA	NA	NA	0.004902	NA	NA	NA	0	NA	NA	0
rs2298665	C	NA	NA	NA	0	NA	NA	NA	0	NA	NA	0
rs142769034	T	NA	NA	NA	0	NA	NA	NA	0.02336	NA	NA	0
rs74564819	C	NA	NA	NA	0	NA	NA	NA	0	NA	NA	0
rs2298857	A	0.3422	0.06522	0.1875	0.2353	0.1818	0.1714	0.1875	0.285	0.197	0.375	0.4352
rs545232590	T	NA	NA	NA	0	NA	NA	NA	0	NA	NA	0
rs529218967	G	NA	NA	NA	0	NA	NA	NA	0	NA	NA	0
rs528337775	T	NA	NA	NA	0.004902	NA	NA	NA	0	NA	NA	0
rs563980567	A	NA	NA	NA	0	NA	NA	NA	0	NA	NA	0
rs534913861	T	NA	NA	NA	0	NA	NA	NA	0	NA	NA	0.00463
rs535348902	A	NA	NA	NA	0	NA	NA	NA	0	NA	NA	0

SNP	Allele	Salvador	Shimaa	Shipibo	STU	Tacna	Tallanes	Trujillo	TSI	Tumbes	Uros	YRI
rs555405939	C	NA	NA	NA	0	NA	NA	NA	0	NA	NA	0
rs187037274	A	NA	NA	NA	0	NA	NA	NA	0	NA	NA	0.00463
rs141027872	T	NA	NA	NA	0	NA	NA	NA	0	NA	NA	0
rs191394761	T	NA	NA	NA	0	NA	NA	NA	0	NA	NA	0
rs540964406	G	NA	NA	NA	0	NA	NA	NA	0	NA	NA	0
rs544440280	A	NA	NA	NA	0	NA	NA	NA	0	NA	NA	0.00463
rs146845793	C	NA	NA	NA	0	NA	NA	NA	0	NA	NA	0
rs372665499	T	NA	NA	NA	0	NA	NA	NA	0	NA	NA	0
rs538276300	A	NA	NA	NA	0	NA	NA	NA	0	NA	NA	0
rs113562865	T	NA	NA	NA	0.004902	NA	NA	NA	0.004673	NA	NA	0.1991
rs571417292	A	NA	NA	NA	0	NA	NA	NA	0	NA	NA	0
rs542865477	A	NA	NA	NA	0	NA	NA	NA	0	NA	NA	0
rs544110139	G	NA	NA	NA	0	NA	NA	NA	0	NA	NA	0
rs142914234	C	NA	NA	NA	0	NA	NA	NA	0	NA	NA	0.01852
rs114549926	A	NA	NA	NA	0	NA	NA	NA	0.004673	NA	NA	0.009259
rs549289994	C	NA	NA	NA	0	NA	NA	NA	0	NA	NA	0
rs555327583	A	NA	NA	NA	0	NA	NA	NA	0	NA	NA	0
rs111220533	G	NA	NA	NA	0.4363	NA	NA	NA	0.4907	NA	NA	0.3009
rs553400003	A	NA	NA	NA	0.03431	NA	NA	NA	0.004673	NA	NA	0
rs530306757	A	NA	NA	NA	0.004902	NA	NA	NA	0	NA	NA	0
rs561412657	G	NA	NA	NA	0	NA	NA	NA	0	NA	NA	0
rs565690211	G	NA	NA	NA	0	NA	NA	NA	0	NA	NA	0
rs137929897	T	NA	NA	NA	0	NA	NA	NA	0	NA	NA	0
rs144458055	A	NA	NA	NA	0.009804	NA	NA	NA	0.004673	NA	NA	0.004902
rs141722242	A	NA	NA	NA	0	NA	NA	NA	0	NA	NA	0.00463
rs565720190	A	NA	NA	NA	0.004902	NA	NA	NA	0	NA	NA	0
rs530051850	C	NA	NA	NA	0	NA	NA	NA	0	NA	NA	0
rs115967323	T	NA	NA	NA	0	NA	NA	NA	0	NA	NA	0.00463
