# Supplementary material for: Human-SARS-CoV-2 interactome and human genetic diversity: TMPRSS2-rs2070788, associated with severe influenza, and its population genetics caveats in Native Americans
Source: Genet Mol Biol. 2021 Aug 25;44(1 Suppl 1):e20200484. doi: 10.1590/1678-4685-GMB-2020-0484 (PMC8387978; doi:10.1590/1678-4685-GMB-2020-0484)
Supplement: Table S4-B - [file 1415-4757-GMB-44-1-s1-e20200484-s9.pdf]

Genetics and Molecular Biology
https://doi.org/10.1590/1678-4685-GMB-2020-0484

Supplementary Material to "Human-SARS-CoV-2 interactome and human genetic diversity: *TMPRSS2*-rs2070788, associated with severe influenza, and its population genetics caveats in Native Americans"

Table S4-B – TMPRSS2 regression.

			rs2070788	rs456298	rs383510	rs2276205	rs12329760
AFR							
	Intercept		0.09531741	-1.054336	-0.21317149	-2.26775735	-1.41862404
	P-Value		0.53005373	3.55E-13	0.01404928	1.00E-54	1.12E-14
	Beta		-1.10211	0.48456	-0.56311	0.75699	0.66432
	P-Value		0.00568	0.1252	0.00457	0.01109	0.10029
	R^2		0.3306	0.13012	0.41807	0.21966	0.09815
	AICc		-14.1826	-21.88605	-24.02818	-49.91898	-22.33803
EUR							
	Intercept		-0.08610978	-0.30313117	-0.76834676	-1.99795923	-1.52512664
	P-Value		0.73063971	0.00789559	5.36E-18	6.08E-22	4.72E-09
	Beta		-0.12079	-1.13861	0.73542	-0.14339	0.51132
	P-Value		0.7582	< 0.001	< 0.001	0.65941	0.18562
	R^2		0.00545	0.73891	0.73972	0.01168	0.08217
	AICc		-7.50144	-39.39788	-33.86	-45.42372	-21.7438
NAT							
	Intercept		-0.50967505	-1.00777652	-0.37144583	-1.88758242	-0.95329427
	P-Value		6.29E-08	9.23E-13	0.00071589	1.65E-63	5.12E-28
	Beta		1.79247	0.94803	0.24465	-1.1957	-2.00514
	P-Value		< 0.001	0.26169	0.69969	0.00359	< 0.001
	R^2		0.75537	0.06746	0.01209	0.51389	0.83256
	AICc		-31.05022	-20.89499	-17.77615	-54.40628	-45.27715
EAS							
	Intercept		-0.10011063	-1.04528563	-0.30122305	-2.18543078	-1.34815305
	P-Value		0.51931724	6.74E-24	0.00130664	5.02E-72	1.28E-17
	Beta		-0.6375	1.18574	-0.48251	1.11165	1.01405
	P-Value		0.3402	0.00091	0.15722	0.00192	0.06143
	R^2		0.05536	0.36913	0.15664	0.2336	0.10463
	AICc		-8.35697	-27.5106	-19.61658	-50.96797	-22.75233
AFR+NAT							
	Intercept		-0.32106072	-1.21887598	-0.19513743	-2.03185651	-0.97790769
	P-Value		0.00051616	1.94E-13	0.05988254	7.94E-50	7.45E-19
	Beta	AFR	-0.66459	0.63745	-0.57975	0.45884	0.08227
		NAT	1.57809	1.40987	-0.15958	-1.03592	-1.97386
	P-Value		0.00132	0.03766	0.0046	0.07533	0.71335
			< 0.001	0.07363	0.75369	0.00976	< 0.001
	R^2		0.85076	0.26768	0.42266	0.57525	0.83245
	AICc		-36.04234	-20.70312	-19.41183	-53.51234	-41.92172
AFR+EUR							
	Intercept		0.61037469	0.18439457	-0.73052321	-2.40331755	-2.1134854
	P-Value		0.01651682	0.1413446	9.46E-07	8.97E-18	1.18E-09
	Beta	AFR	-1.57336	-0.74139	-0.05966	0.87858	1.26913
		EUR	-0.81163	-1.67169	0.69434	0.2095	1.04634
	P-Value		7.00E-05	< 0.001	0.75325	0.01755	0.00512
			0.0156	< 0.001	0.00013	0.56078	0.01448
	R^2		0.50138	0.87592	0.74191	0.23991	0.35685
	AICc		-15.92686	-49.17114	-29.24426	-46.7765	-24.99586
AFR+EAS							
	Intercept		0.20182169	-1.25731914	-0.11182958	-2.45981639	-1.56932582

		rs2070788	rs456298	rs383510	rs2276205	rs12329760
P-Value		0.17160744	1.93E-33	0.08578487	1.97E-98	1.79E-18
Beta	AFR	-1.25381	0.70953	-0.68489	0.94901	0.82187
	EUR	-1.01796	1.41757	-0.71587	1.3815	1.23972
P-Value		0.00071	0.00074	< 0.001	2.00E-05	0.02573
		0.05756	< 0.001	0.00038	< 0.001	0.01273
R^2		0.45293	0.62962	0.72917	0.55817	0.24977
AICc		-14.26736	-31.89502	-28.53623	-58.33904	-23.37902
**NAT+EUR**						
			-	-		
Intercept		-0.96761381	0.36934889	0.80019026	-1.41752315	-0.7313957
P-Value		1.36E-14	0.00064438	3.91E-18	5.44E-32	4.80E-08
Beta	NAT	2.21555	0.81107	0.31432	-1.66575	-2.20223
	EUR	0.73312	-1.14963	0.74262	-0.82662	-0.37316
P-Value		< 0.001	0.04843	0.32528	< 0.001	< 0.001
		1.00E-05	< 0.001	< 0.001	1.00E-05	0.04829
R^2		0.88469	0.79131	0.75953	0.74967	0.85152
AICc		-40.83882	-38.93301	-30.04975	-63.61455	-45.25398
**NAT+EAS**						
			-	-		
Intercept		-0.47971984	1.15870361	0.31503821	-1.99569722	-1.02327673
P-Value		8.46E-07	6.07E-25	0.00374905	2.13E-88	1.32E-37
Beta	NAT	1.75969	1.19229	0.1477	-1.11774	-1.9429
	EAS	-0.32461	1.2687	-0.47399	0.90996	0.659
P-Value		< 0.001	0.05512	0.80275	0.00132	< 0.001
		0.36581	1.00E-04	0.16613	0.00067	0.00763
R^2		0.76694	0.4744	0.16095	0.65926	0.86824
AICc		-28.39966	-26.73796	-14.96408	-58.08656	-47.32891
**EUR+EAS**						
			-	-		
Intercept		0.04681986	0.46140358	0.75562842	-2.22240111	-1.78849255
P-Value		0.86068709	2.77E-05	4.16E-13	3.36E-26	1.81E-11
Beta	EUR	-0.26925	-0.96175	0.72162	0.06678	0.76613
	EAS	-0.77844	0.60879	-0.0487	1.14784	1.43268
P-Value		0.50002	< 0.001	< 0.001	0.82786	0.03873
		0.26051	0.00514	0.81713	0.00366	0.00697
R^2		0.07946	0.81087	0.74087	0.23567	0.27104
AICc		-5.31844	-41.89938	-29.19952	-47.52863	-23.49095
**AFR+NAT+EUR**						
				-		
Intercept		-0.84105746	0.12001129	0.81532249	-1.08553452	-0.36125504
P-Value		0.00038664	0.39619351	2.72E-06	7.05E-09	0.10413716
Beta	AFR	-0.17153	-0.6793	0.02117	-0.4723	-0.51678
	NAT	2.08815	0.27971	0.33061	-2.01434	-2.58885
	EUR	0.6087	-1.63067	0.75766	-1.1502	-0.73541
P-Value		0.53962	5.00E-05	0.91817	0.04105	0.05343
		< 0.001	0.37272	0.35402	< 0.001	< 0.001
		0.01804	< 0.001	8.00E-05	< 0.001	0.00312
R^2		0.88752	0.88205	0.75973	0.78605	0.87203
AICc		-37.07498	-45.27769	-23.77458	-63.021	-44.42283
**AFR+NAT+EAS**						
			-	-		
Intercept		-0.23235132	1.51065748	0.05766256	-2.23574397	-1.09666949
P-Value		0.00764227	5.64E-93	0.43763248	1.08E-95	1.08E-26
Beta	AFR	-0.78024	0.95137	-0.73649	0.67791	0.21863
	NAT	1.47945	1.91035	-0.42704	-0.86413	-1.85343
	EAS	-0.60872	1.63068	-0.75766	1.15023	0.73543
P-Value		2.00E-05	< 0.001	< 0.001	0.00026	0.25729
		< 0.001	< 0.001	0.20192	0.00281	< 0.001
		0.01804	< 0.001	8.00E-05	< 0.001	0.00312
R^2		0.88752	0.88205	0.75973	0.78605	0.87203
AICc		-37.07517	-45.27772	-23.77448	-63.02135	-44.42314
**AFR+EUR+EAS**						
				-		
Intercept		1.24710167	0.39972046	0.48471817	-3.09988654	-2.95012581
P-Value		1.46E-13	0.13959343	0.10477512	6.49E-36	1.07E-23

		rs2070788	rs456298	rs383510	rs2276205	rs12329760
Beta	AFR	-2.2597	-0.95901	-0.30944	1.54206	2.07209
	EUR	-1.47945	-1.91038	0.42706	0.86414	1.85346
	EAS	-2.08819	-0.27971	-0.33061	2.01438	2.58891
P-Value		< 0.001	0.00088	0.33789	< 0.001	< 0.001
		< 0.001	< 0.001	0.20191	0.00281	< 0.001
		< 0.001	0.37272	0.35403	< 0.001	< 0.001
R^2		0.88752	0.88205	0.75973	0.78606	0.87204
AICc		-37.07562	-45.27768	-23.77454	-63.02162	-44.42394
**NAT+EUR+EAS**						
Intercept		-1.0125858	- 0.55928834	- 0.79415594	-1.55783642	-0.87804209
P-Value		4.95E-12	6.31E-10	3.56E-13	2.76E-31	2.42E-09
Beta	NAT	2.25968	0.95899	0.30944	-1.54204	-2.07206
	EUR	0.78023	-0.95137	0.73649	-0.6779	-0.21862
	EAS	0.17152	0.67931	-0.02116	0.47231	0.5168
P-Value		< 0.001	0.00088	0.33788	< 0.001	< 0.001
		2.00E-05	< 0.001	< 0.001	0.00026	0.25731
		0.53965	5.00E-05	0.91818	0.04105	0.05342
R^2		0.88752	0.88205	0.75973	0.78605	0.87203
AICc		-37.07493	-45.2777	-23.77458	-63.02115	-44.42305
**AFR+NAT+EUR+EAS**						
Intercept		14182.5351	- 544.663786	-4978.0388	-22328.9678	-28900.9709
P-Value		0.1099917	0.94305153	0.54812676	0.0354136	0.0017381
Beta	AFR	-14183.5922	544.10571	4977.24828	22327.4525	28900.1523
	NAT	-14181.2583	545.05485	4977.48302	22325.806	28897.9433
	EUR	-14182.7268	543.15256	4977.9669	22326.6651	28899.7878
	EAS	-14183.5052	544.7891	4977.271	22328.0983	28900.8863
P-Value		0.10997	0.94311	0.54819	0.03543	0.00174
		0.11002	0.94301	0.54817	0.03544	0.00174
		0.10999	0.94321	0.54813	0.03543	0.00174
		0.10997	0.94304	0.54819	0.03542	0.00174
R^2		0.90191	0.88242	0.76639	0.82868	0.92277
AICc		-34.45985	-39.44958	-15.32982	-61.78145	-47.0586
